# Mild and Chemoselective Triethylsilane-Mediated Debenzylation for Phosphate Synthesis

**DOI:** 10.1021/acs.orglett.4c04258

**Published:** 2024-12-24

**Authors:** Luke E. Hodson, Paul Joseph Tholath, Leon Jacobs, Nicole Pribut, Gouthami Pashikanti, Aletta E. van der Westhuyzen, David Laws, Dennis C. Liotta

**Affiliations:** Department of Chemistry, Emory University, 1515 Dickey Drive, Atlanta, Georgia 30322, United States

## Abstract

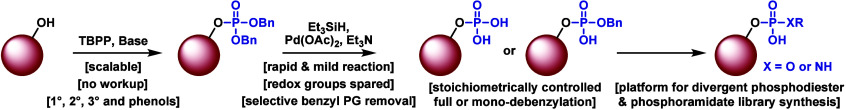

The synthetic utility of tetrabenzyl pyrophosphate for achieving chemoselective phosphorylation of phenols, as well as primary, secondary, and tertiary alcohols, is reported here. Additionally, we introduce a rapid, mild, and chemoselective debenzylation procedure, enabling access to phosphates in the presence of redox sensitive groups. Finally, stoichiometrically controlled monodebenzylation provides a versatile platform for late-stage divergent synthesis of phosphodiester and phosphoramidate chemical libraries.

Phosphorylation plays a pivotal role in nature and is profoundly implicated in human health and disease.^1^ As a regulatory mechanism, protein phosphorylation controls nearly every aspect of cellular function.^2^ The phosphate moiety has also found practical application in materials science,^3^ catalysis,^4^ agrochemicals,^5^ and medicine.^6^ While drug design opportunities incorporating a phosphate are abundant, phosph(on)ate prodrugs are also often employed to overcome the intrinsic challenges of membrane penetration, stability, and oral bioavailability.^7^ The broad importance of this functional group has led to extensive efforts to develop selective and efficient synthetic phosphorylation protocols.^8^ Nevertheless, many existing methods suffer shortcomings, such as harsh reaction conditions and difficult reactivity profiles that require multistep processing.^9^

Cannabidiol [CBD, **1** (Figure 1)] exhibits a host of beneficial therapeutic effects^10^ and recently received Food and Drug Administration orphan drug approval as Epidiolex for the treatment of refractory epilepsies and tuberous sclerosis complex.^11^ Despite the success of Epidiolex, CBD exhibits poor drug-like properties, including its marginal aqueous solubility.^12^ In our effort to develop water-soluble CBD prodrugs, we envisaged phenolic phosphate **2** (Figure 1) could emulate the prodrug character of psilocybin, providing critical solubility before cleavage by endogenous phosphatases prior to absorption.^13^ Surprisingly, other than prophetic examples in the patent literature, a viable synthetic route to **2** has not been reported. However, this can certainly be attributed to the challenging reactivity profile of CBD (Figure 1). CBD readily undergoes base-mediated aerobic oxidation to cannabidiolquinone (CBDQ),^14^ while use of Lewis acids or strongly acidic conditions leads to isomerization around the isopropylidene to other cannabinoids.^15^ The monoterpene double bonds are also highly susceptible to redox chemistry.^16^ These liabilities severely reduce available options and render most phosphorylation methods incompatible with **1** (Figure 1).

**Figure 1:**
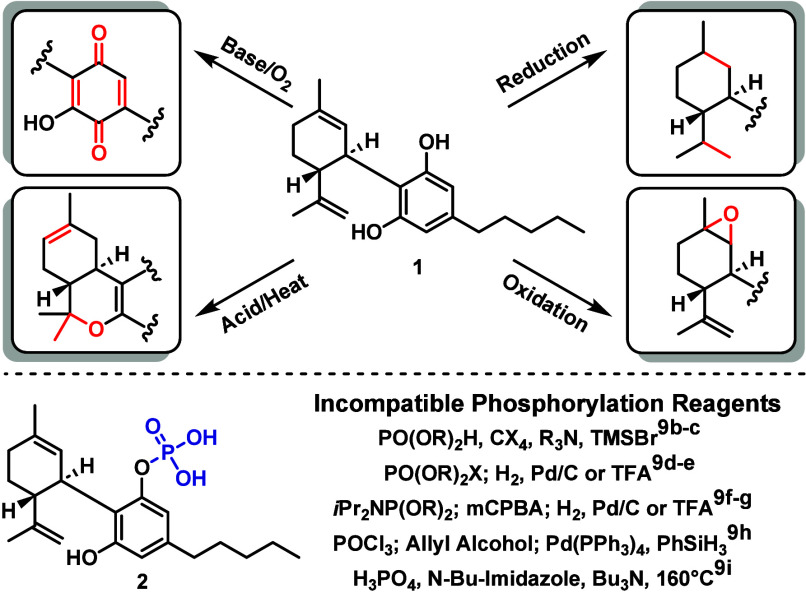
Synthetic limitations of cannabidiol and the incompatible commonly employed phosphorylation methods.

Tetrabenzyl pyrophosphate (TBPP) has been used frequently in the synthesis of numerous phosphate-containing biologically active molecules and prodrugs.^17^ This inexpensive reagent is primarily used for O- or N-phosphorylation with organolithium and other strong bases, although titanium alkoxides have also proven to be selective and effective as Lewis acid catalysts.^18^ Recently, Huters et al. utilized TBPP in the development of a scalable synthesis of foslevodopa.^19^ By employing the previously unreported use of DBU as a base, the group avoided problems associated with stronger bases, enabling isolation of the corresponding dibenzyl phosphate in excellent yield. Facing similar incompatibilities with CBD, we hypothesized that the use of these conditions could provide an accessible route to phosphate prodrug **2**. To explore the feasibility of this idea, a series of optimization studies were conducted (Table 1).

**Table 1:**
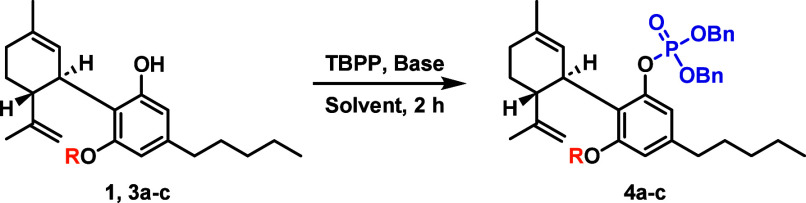
Optimization of TBPP Phosphorylation^a^

entry	R	TBPP (equiv)	base (equiv)	solvent	yield (%)^b^
1	H	1	DBU (1.2)	MeCN	ND
2	TES (**3a**)	1	DBU (1.2)	MeCN	16 (**4a**)
3	TBDMS (**3b**)	1	DBU (1.2)	MeCN	32 (**4b**)
4	TIPS (**3c**)	1	DBU (1.2)	MeCN	71 (**4c**)
5	TIPS	1.2	DBU (1)	MeCN	52
6	TIPS	1.2	DBU (1.5)	MeCN	74
7	TIPS	1.2	DBU (2)	MeCN	67
8	TIPS	2	DBU (1.5)	MeCN	66
9	TIPS	4	DBU (1.5)	MeCN	39
10	TIPS	1.2	*t*-BuOK (1.5)	MeCN	62
11	TIPS	1.2	MTBD (1.5)	MeCN	75
12	TIPS	1.2	DIPEA (1.5)	MeCN	0
13	TIPS	1.2	DMAP (1.5)	MeCN	0
14	TIPS	1.2	*n*-BuLi (1.5)^c^	THF	81
15	TIPS	1.2	*t*-BuMgCl (1.2)^c^	THF	71
16	TIPS	1.2	DBU (1.5)	THF	51
17	TIPS	1.2	DBU (1.5)	DCM	50
18	TIPS	1.2	DBU (1.5)	DMF	49
19	TIPS	1.2	DBU (1.5)	MeCN	76^d^

a Reaction conditions: **1** or **3** (0.1 mmol), TBPP in a solvent (2.0 mL), and then a base for 2 h.

b Yield determined by ^31^P qNMR of the crude mixture with triphenyl phosphate as the internal standard.

c Deprotonation first and then TBPP.

d Isolated yield on a 10 g scale.

Unsurprisingly, pilot studies using native CBD with DBU and TBPP resulted in CBDQ formation and compound degradation. Given the necessity of a protecting group, we chose to utilize silyl ethers for their compatibility, simple installation (nominal double protection), and facile removal (Table 1, entries 2–19). We observed that increasing the steric bulk of the silyl derivative progressively inhibited oxidation following deprotonation by DBU, with TIPS-CBD (**3c**) yielding the best results [**4c** (Table 1, entry 4)]. Next, the base stoichiometry was explored (Table 1, entries 5–8), with 1.5 equiv of DBU found to be optimal (74% yield), while increasing the amount of available TBPP only decreased the yield (Table 1, entries 8 and 9). Numerous other bases and catalysts were evaluated (Table 1, entries 10–15). No phosphorylation occurred with weaker bases, such as DIPEA and DMAP, whereas yields increased proportionally with base p*K*_a_ values, confirming that reactivity is driven by phenoxide formation. While *n*-BuLi provided higher yields, its widely reported use with TBPP prompted us to explore DBU as a milder, more scalable, and readily accessible alternative. Lastly, a solvent screen (Table 1, entries 16–18) revealed significantly lower performance across all alternatives compared to that with MeCN, likely due to the enhancement of DBU’s effective p*K*_a_. To demonstrate the scalability of the phosphorylation, the optimized reaction conditions were used on a 10 g preparative scale, which furnished compound **4c** in 76% yield (Table 1, entry 19). Finally, removal of the silyl ether was readily accomplished in nearly quantitative yield using TBAF and acetic acid to afford compound **5** (see the Supporting Information for complete phosphorylation optimization details).

With dibenzyl phosphate compound **5** prepared, an effective deprotection method needed to be conceived and implemented. Presenting a greater challenge, traditional hydrogenation and other unconventional methods (use of oxidants, TFA, or Lewis acids) proved to be unworkable due to the synthetic limitations of CBD (see the Supporting Information).^20^ We were drawn to the mild and chemoselective conditions reported by Coleman et al. that permitted cleavage of benzyl protecting groups using palladium acetate, triethylsilane, and Et_3_N.^21^ The procedure, based on earlier disclosures by Birkoffer^22^ and Sakaitani,^23^ allowed for selective removal of benzyl ether, ester, and carbamate protecting groups in the presence of easily reducible functional groups. While there is no literature precedent for the use of these conditions in phosphate debenzylation, we postulated that the markedly gentle conditions would be compatible with CBD. An investigation of the utility of this methodology was launched (Table 2).

**Table 2:**
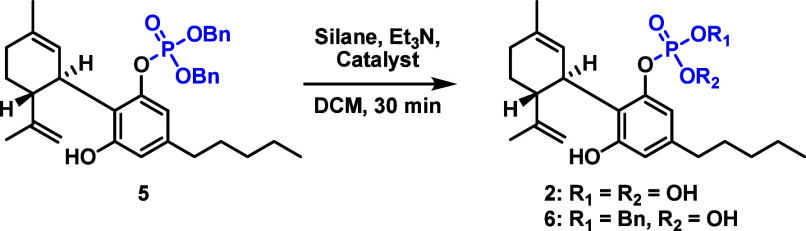
Optimization of Phosphate Benzyl Deprotection^a^

				yield (%)^b^	
entry	silane (equiv)	Et_3_N (equiv)	catalyst (mol %)	**2**	**6**
1	Et_3_SiH (2)	0.2	Pd(OAc)_2_ (5)	40	16
2	Et_3_SiH (1)	0.2	Pd(OAc)_2_ (5)	0	35
3	Et_3_SiH (1.25)	0.2	Pd(OAc)_2_ (5)	2	60
4	Et_3_SiH (1.5)	0.2	Pd(OAc)_2_ (5)	10	56
5	Et_3_SiH (2.5)	0.2	Pd(OAc)_2_ (5)	67	13
6	Et_3_SiH (4)	0.2	Pd(OAc)_2_ (5)	58	15
7	Et_3_SiH (2.5)	0	Pd(OAc)_2_ (5)	0	0
8	Et_3_SiH (2.5)	3	Pd(OAc)_2_ (5)	60	2
9	Et_3_SiH (2.5)	0.2	Pd(OAc)_2_ (50)	41	0
10	Et_3_SiH (2.5)	0.2	Pd/C (20)	0	0
11	Et_3_SiH (2.5)	0.2	no catalyst	0	0
12	PhSiH (2.5)	0.2	Pd(OAc)_2_ (5)	17	30
13	EtO_3_SiH (2.5)	0.2	Pd(OAc)_2_ (5)	54	18
14	Et_3_SiH (1.25)	0.2	Pd(OAc)_2_ (5)	ND	71^c^,^d^
15	Et_3_SiH (2.5)	0.2	Pd(OAc)_2_ (5)	78^c^	ND

a Reaction conditions: **5** (0.1 mmol), Pd(OAc)_2_ in DCM (2.0 mL), and then Et_3_N and silane for 30 min.

b Yield determined by ^31^P qNMR of the crude mixture with triphenyl phosphate as the internal standard.

c Isolated yield on a1 g scale.

d Isolated as an ammonium salt.

Initially, 2 equiv of Et_3_SiH (Table 2, entry 1) was used to minimize the potential reduction of the redox sensitive terpene alkenes. Gratifyingly, no reduction was observed, and target phosphate **2** was generated in moderate yield after 30 min. Interestingly, intermediate monodebenzylated species **6** was also detected in an ∼1:3 ratio with **2**. Controlled phosphate monodebenzylation is a challenging endeavor, with the most frequently employed methods using excess sodium iodide (or other metal halides).^24^ These conditions require higher temperatures and prolonged reaction times and often afford low yields. To our delight, reducing the amount of reductant (Table 2, entries 2–4) led to selective monodebenzylation, with minimal formation of **2**. Monodebenzylation was effective up to 1.5 equiv of Et_3_SiH, with 1.25 equiv being optimal. This result unlocked the exciting utility of a stoichiometrically controlled phosphate debenzylation reaction pathway. For complete debenzylation, doubling the molar amount of Et_3_SiH to 2.5 equiv yielded the optimal stoichiometric ratio, with larger amounts resulting in diminishing returns (Table 2, entries 5 and 6). Although it was hypothesized that excess Et_3_N could impede the progress of the reaction beyond monodebenzylation due to salt formation, the stoichiometry of Et_3_SiH proved to be the key determinant in the reaction (Table 2, entries 7 and 8). Lastly, the use of varying amounts and sources of a palladium catalyst or alternative silane reductants resulted in decreased efficiency or no reaction (Table 2, entries 9–13). Application of the optimized conditions on a 1 g scale (Table 2, entries 14 and 15) led to isolated yields of 71% for **6** and 78% for **2** in <1 h, emphasizing the simplicity, reliability, and speed of this transformation.

We conducted a comprehensive investigation of the capabilities of phosphorylation utilizing TBPP, as well as the chemoselective nature and substrate tolerance of Et_3_SiH-mediated deprotection. This assessment, exemplified by the phosphorylation and debenzylation of more than 30 compounds (Figure 2), highlights both the significant utility and reasonable limitations of these methodologies (see the Supporting Information for the complete substrate scope). First, an evaluation of both steric and electronic substituents revealed a minimal impact on the transformations (Figure 2, panel 1), as even the sterically congested propofol underwent phosphorylation to afford **8a** in moderate yield. Notably, the hydrolytic instability of **10a** necessitated omission of standard workup conditions, resulting in an improved yield and the convenience of foregoing workup when required (see the Supporting Information). Additionally, no reduction of the nitro- and chloroaromatic moieties was observed (**10b** and **11b**). While the reaction was typically more challenging, DBU was sufficient to promote phosphorylation of activated tertiary alcohol **12** (Figure 2, panel 2). In contrast, inactivated substrates, such as **13** and **14**, required stronger bases to facilitate alkoxide formation. A simple screen identified *n*-BuLi as the optimal choice, but *t*-BuMgCl was determined to be only marginally less effective. The compatibility of TBPP with a wide range of bases is highly advantageous, allowing for tailored base selection for specific scenarios (see the Supporting Information). Next, the controlled monophosphorylation of the polyphenol substrates curcumin (**15**), resveratrol (**16**), and pterostilbene (**17**) produced moderate yields and reactivity (Figure 2, panel 3) yet demonstrated an improvement over existing literature precedent.^25^ Importantly, Et_3_SiH-mediated debenzylation showed no reduction of the α,β-unsaturated diketone or internal alkene and outperformed the cited TMSBr deprotection method (see the Supporting Information for details of **16**).^26^ This methodology also provides an excellent route to phosphonate substrates (Figure 2, panel 4), enabling access to **18b**, which was previously unattainable via benzyl deprotection.^27^ Enthusiastic about the utility of stoichiometrically controlled debenzylation, we evaluated the propensity for selective and complete debenzylation in the presence of related protecting groups (Figure 2, panel 5). For compounds **20a**–**24a**, phosphorylation proceeded smoothly, and subsequent deprotections were performed using 1.25 equiv of Et_3_SiH per benzyl group. We were pleased to observe that benzyl ester (**20a**)- and Cbz (**21a**)-protected compounds readily underwent rapid global deprotection (**20c** and **21b**, respectively), providing an alternative to traditional catalytic hydrogenation (see the Supporting Information for details of **21b**). Moreover, chemoselective phosphate debenzylation was performed to isolate **20b** in good yield, underscoring the mild and selective nature of the reaction, which could also be applied to compounds **22**–**24**. For benzyl ether (**22a**) and benzylamine (**23a**) analogues, selective phosphate debenzylation was effortlessly attained, affording **22b** and **23b**, respectively. While achievable, complete deprotection of **22a** and **23a** required stringent conditions, including excess Et_3_SiH and longer reaction times, affording **22c** and **23c**, respectively. Lastly, allyl ether **24a** proved to be impervious to these conditions, offering a potentially synergistic protection strategy due to the distinct removal strategies.

**Figure 2:**
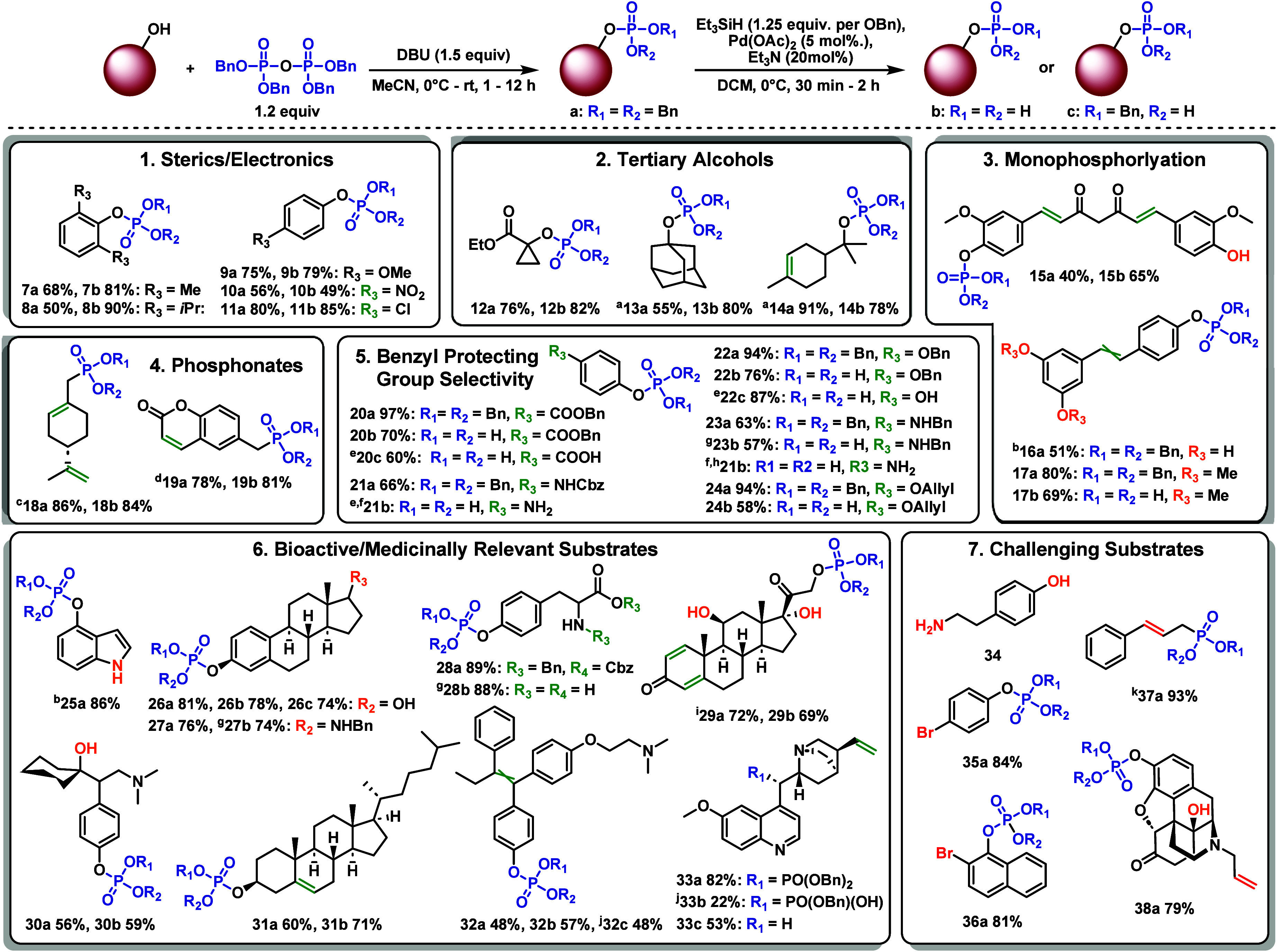
Scope of TBPP-based phosphorylation and triethylsilane-mediated debenzylation. Unless otherwise stated, condition a denotes dibenzyl phosphate, condition b denotes fully debenzylated phosphate, and condition c denotes monobenzyl phosphate derivative. Blue represents installed phosph(on)ates, green redox sensitive/benzyl protecting groups, orange competitive nucleophiles, and red problematic functionalities. ^a^*n*-BuLi was used as the base. ^b^Deprotection results in compound degradation. ^c^Synthesized from (*S*)-(−)-perillyl alcohol over two steps. ^d^Synthesized from 6-(bromomethyl)-2*H*-chromen-2-one. ^e^With 4 equiv of Et_3_SiH. ^f^Isolated as a crude Et_3_N salt. ^g^Isolated as a HCl salt. ^h^With 8 equiv of Et_3_SiH. ^i^LDA was used as the base. ^j^Isolated as an ammonium salt. ^k^Synthesized from cinnamyl bromide.

With a thorough understanding of the parameters of the phosphorylation and debenzylation reactions, we applied these methodologies to several medicinally relevant substrates (Figure 2, panel 6). Key highlights of the specific utility of TBPP phosphorylation include selectivity between phenols and secondary amines (**25a** and **27a**) and a range of hydroxyl selectivities (**26a**, **29a**, and **30a**). Importantly, the successful global deprotection of **28a** to afford phosphotyrosine (**28b**) with an excellent yield represents a new avenue for the deprotection of Cbz- and benzyl ester-protected amino acids. As with CBD, redox sensitive groups were preserved throughout and stoichiometrically controlled usage of Et_3_SiH enabled the successful synthesis of several monobenzyl phosphate analogues (**26c**, **31c**, **32c**, and **33b**). Finally, investigation of asymmetrically substituted tribenzylic phosphates, such as **33a**, allowed for only selective monodebenzylation to afford **33b**. Increasing the amount of Et_3_SiH did not allow for the isolation of the fully debenzylated product. However, these conditions notably enabled deoxygenation at this site, a transformation that typically necessitates metal catalysts or harsh reductive conditions, while sparing the vinyl alkene.^28^ This approach yielded **33c** with improved efficiency over existing methods (without further optimization), offering a mild and attractive alternative to similar deoxygenation reactions.

We encountered certain limitations with this methodology (Figure 2, panel 7), foremost being selective phosphorylation in the presence of primary amines. Although we anticipated that the oxophilic nature of the magnesium counterion would be sufficient to promote preferential O-phosphorylation in **34** using *t*-BuMgCl as a base, the reaction instead yielded an ∼1:1 mixture of O- and N-phosphorylated products. Further improvements in selectivity were deemed to be outside of the scope of this work. While phosphorylation was successful, achieving selective debenzylation proved to be challenging in the presence of certain functional groups (**35**–**38**). Despite a careful experimental setup, competitive aryl bromide hydrogenolysis in both **35a** and **36a** remained unavoidable under these conditions. While strict adherence to the stoichiometry and temperature granted access to numerous styrene derivatives, reduction was observed in **37a**. Finally, a significant disparity in reduction susceptibility was observed for allylic amines, where undesirable allylic reduction precluded the selective debenzylation of phosphate derivative **38a**. This functional group’s sensitivity was further corroborated by the rapid reduction of *N*-allylaniline (see the Supporting Information).^29^

To underscore the significance and utility of this stoichiometrically controlled deprotection methodology, we illustrated the synthesis of phosphodiester and phosphoramidate derivatives (Scheme 1). Selective monodebenzylation of **4c** provided access to **40**, which served as a versatile building block for reaction with various nucleophiles. To exemplify this, **40** was allowed to react with butanol and butylamine, using CCl_3_CN and PPh_3_, followed by desilylation to afford **41a** and **41b**. Subsequent debenzylation afforded the phosphodiester and phosphoramidate products (**42a** and **42b**, respectively) in excellent yield. The mild conditions and broad functional group tolerance of this synthetic sequence make it highly suitable for late-stage divergent synthesis. This approach facilitates the development of new phosphorus-containing drug candidates, a class that already features prominently in clinical research.^30^

**Scheme 1:**
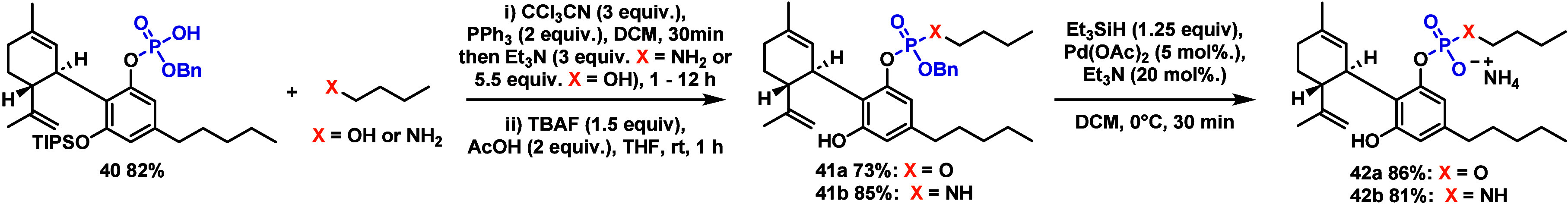
Monodebenzylation-Mediated Phosphodiester and Phosphoramidate Synthesis

In conclusion, we have comprehensively explored the boundaries of base-mediated TBPP phosphorylation, demonstrating DBU as a highly effective option while showcasing chemoselectivity through substrate-tailored base selection. Most notably, we present a stoichiometrically controlled debenzylation method, allowing for rapid deprotection under mild conditions. This approach offers broad functional group tolerance while preserving redox sensitive groups and unprecedented selectivity among various benzyl protecting groups. Finally, the broad applicability of this novel deprotection method holds great potential for late-stage divergent synthesis, enabling streamlined access to phosphodiester and phosphoramidate libraries.

## Data Availability

The data underlying this study are available in the published article and its Supporting Information.

## Supporting Information Available

NMR spectra of all new compounds (PDF)
Detailed experimental procedures, optimization details, and analytical and spectroscopic data (^1^H NMR, ^13^C NMR, ^31^P NMR, and MS) (PDF)
