# Supplementary material for: Mild and Chemoselective Triethylsilane-Mediated Debenzylation for Phosphate Synthesis
Source: Org Lett. 2024 Dec 24;27(1):246–51. doi: 10.1021/acs.orglett.4c04258 (PMC11731398; doi:10.1021/acs.orglett.4c04258)

NMR Spectra

Mild and Chemoselective Triethylsilane Mediated Debenzylation for Phosphate Synthesis

Luke E. Hodson‡, Paul Joseph Tholath‡, Leon Jacobs, Nicole Pribut, Gouthami Pashikanti, Aletta E. van der Westhuyzen, David Laws III and Dennis C. Liotta*

Department of Chemistry, Emory University, 1515 Dickey Drive, Atlanta, Georgia, 30322, United States

*Correspondence to: dliotta@emory.edu

^{1}H NMR of Compound **3a** (600 MHz, $CDCl_3$)

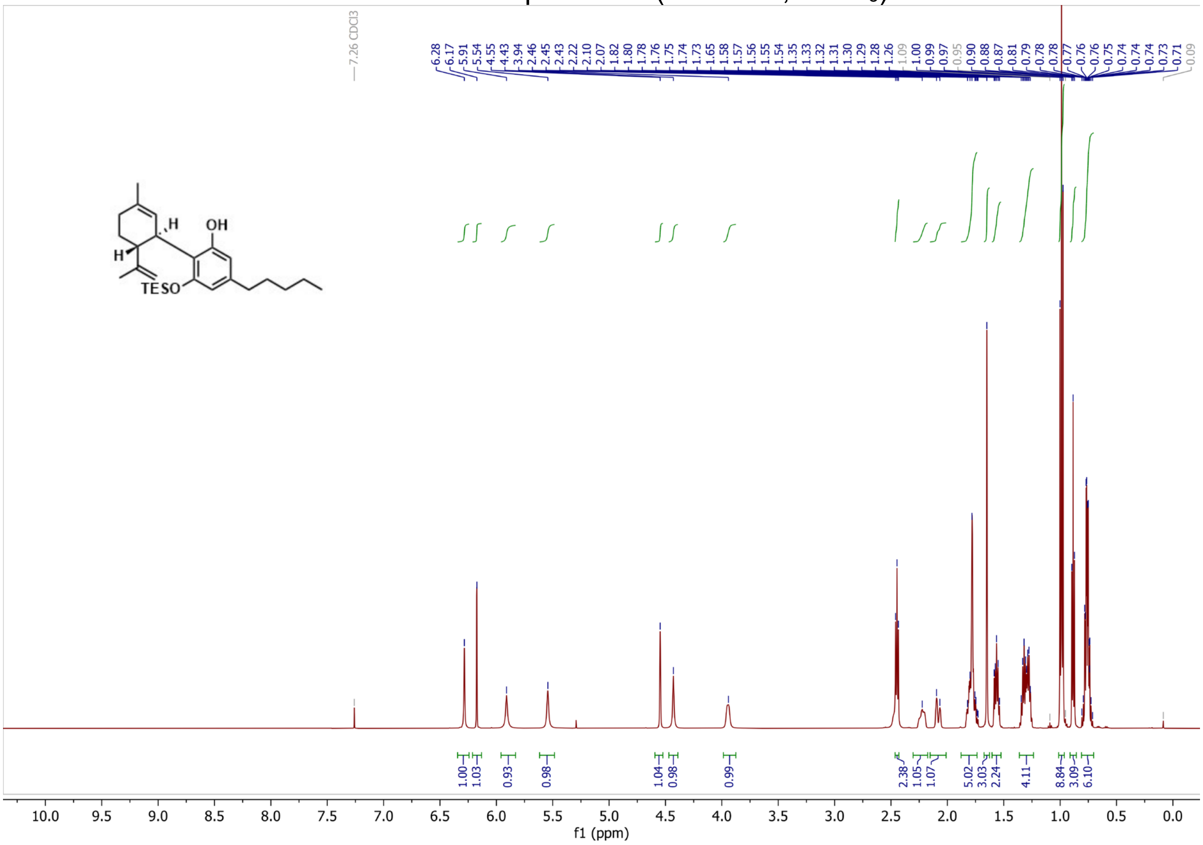

^{13}C NMR of Compound **3a** (151 MHz, $CDCl_3$)

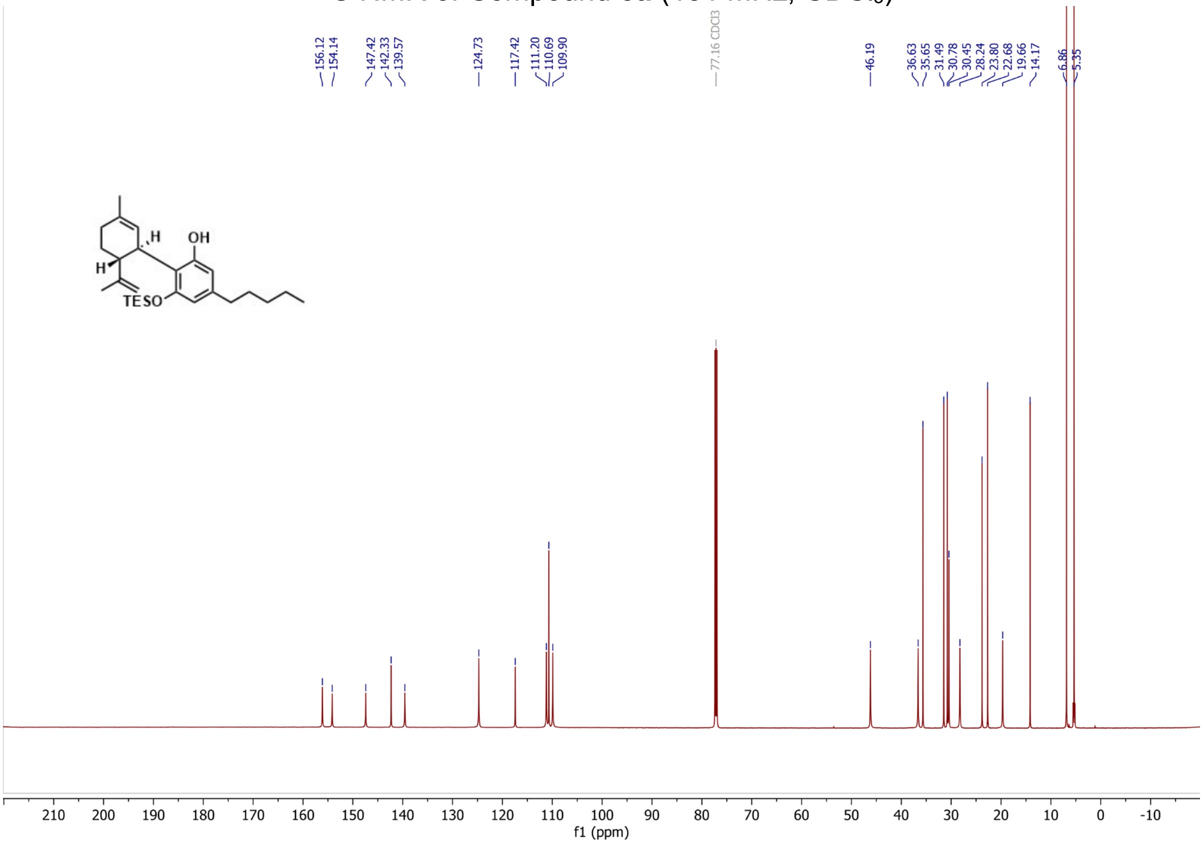

^{1}H NMR of Compound **3b** (600 MHz, $CDCl_3$)

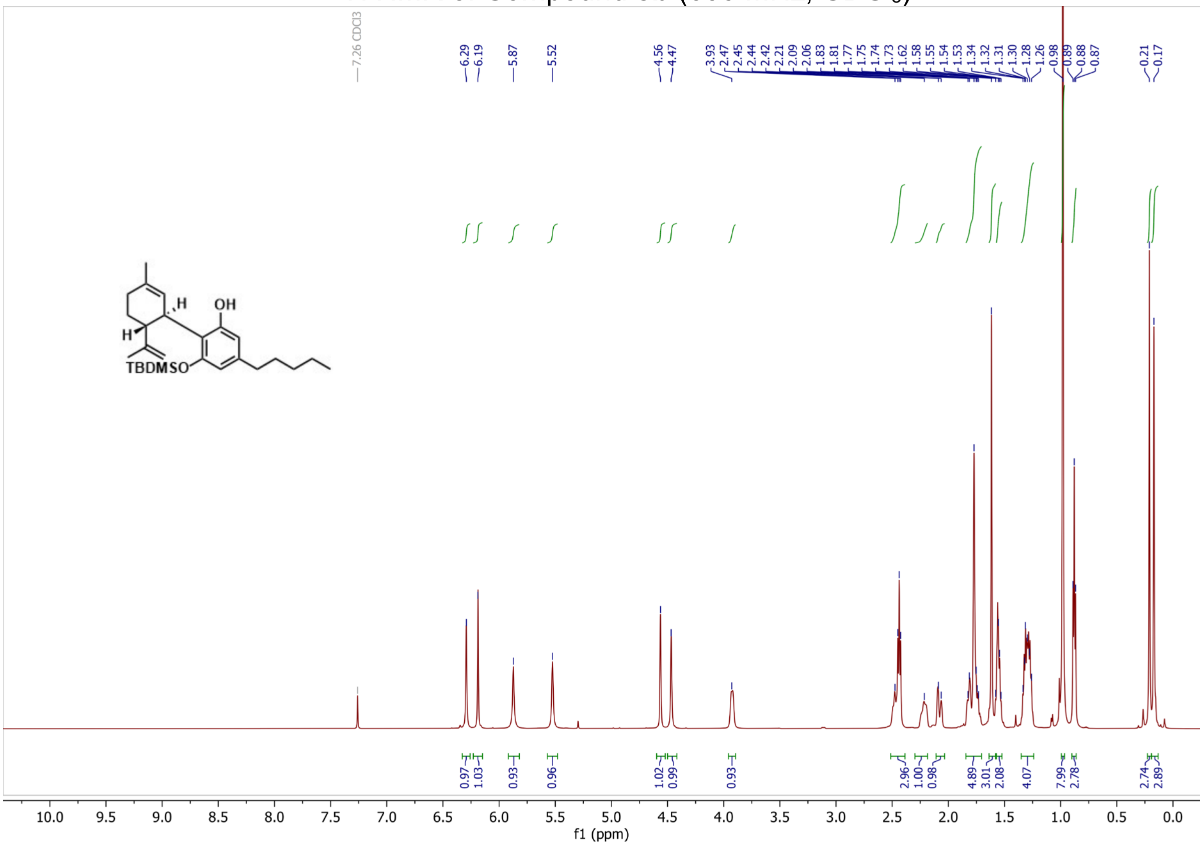

^{13}C NMR of Compound **3b** (151 MHz, $CDCl_3$)

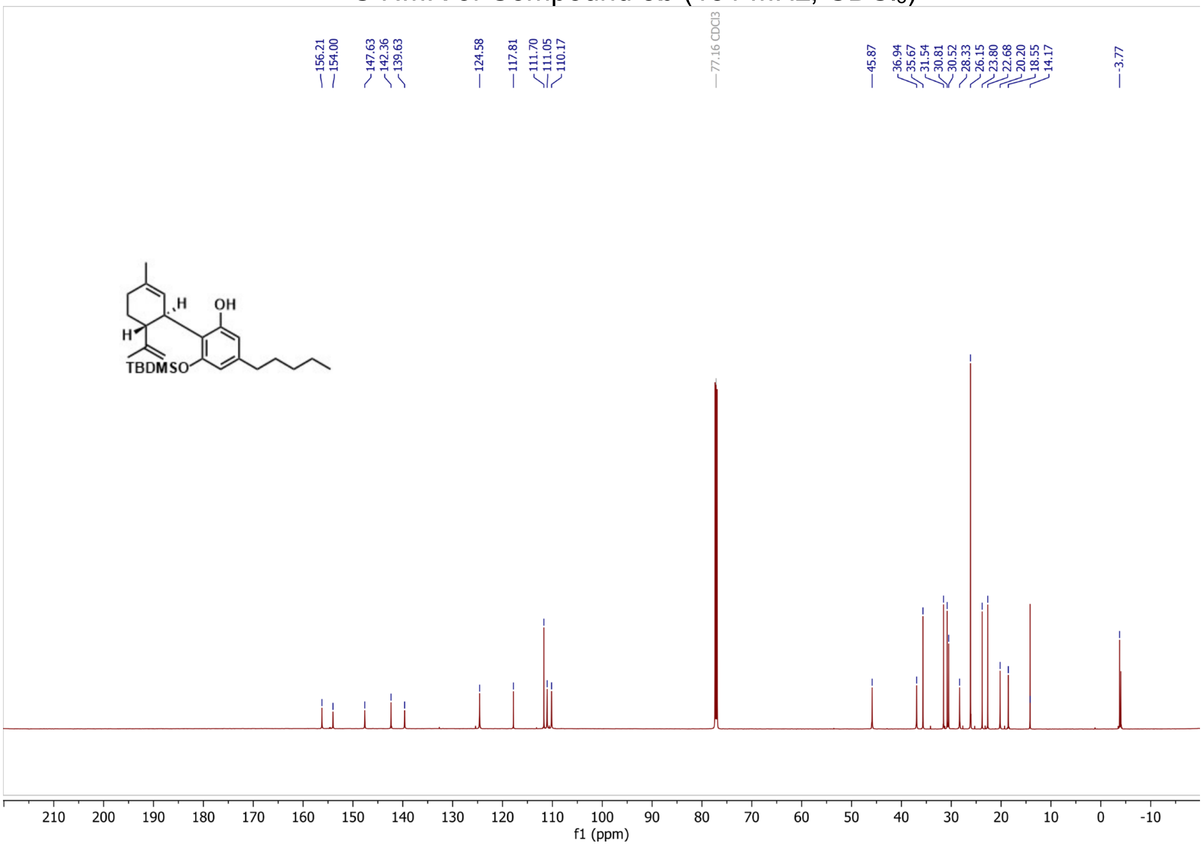

^{1}H NMR of Compound **3c** (600 MHz, $CDCl_3$)

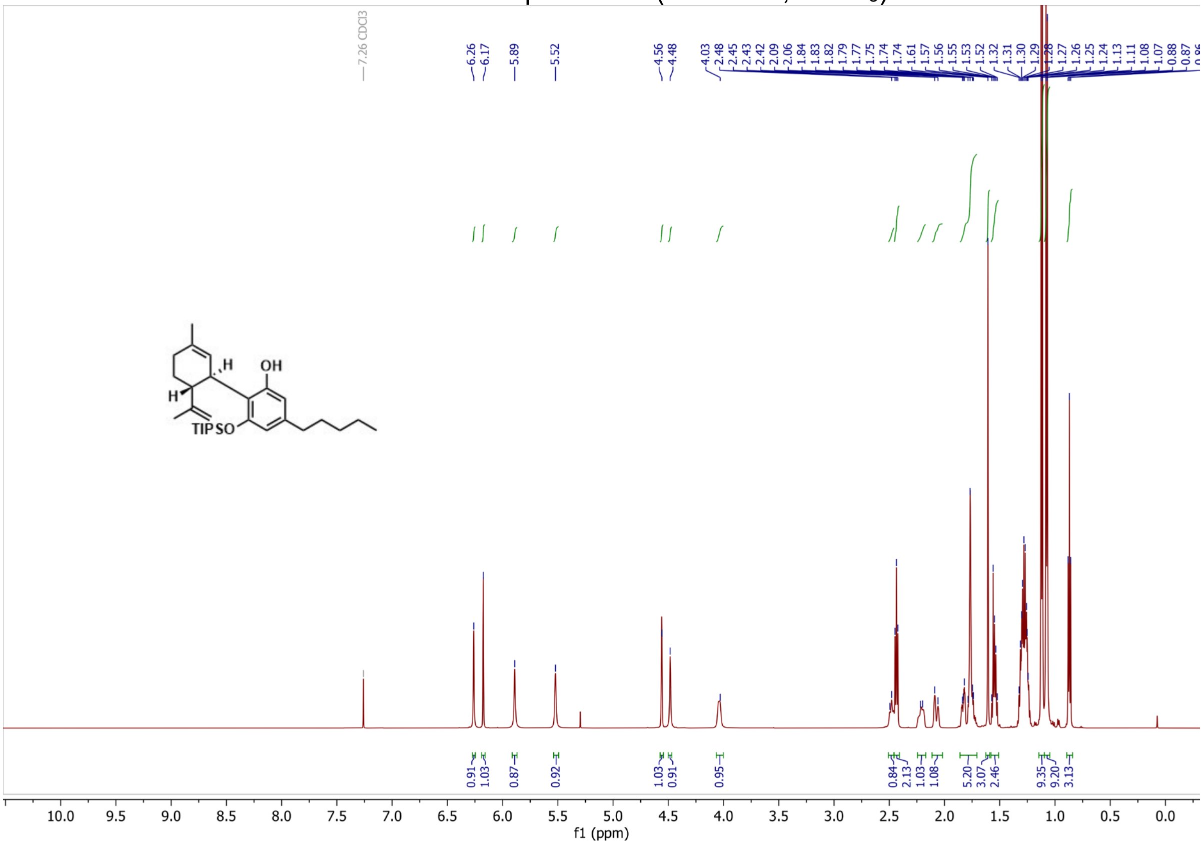

^{13}C NMR of Compound **3c** (151 MHz, $CDCl_3$)

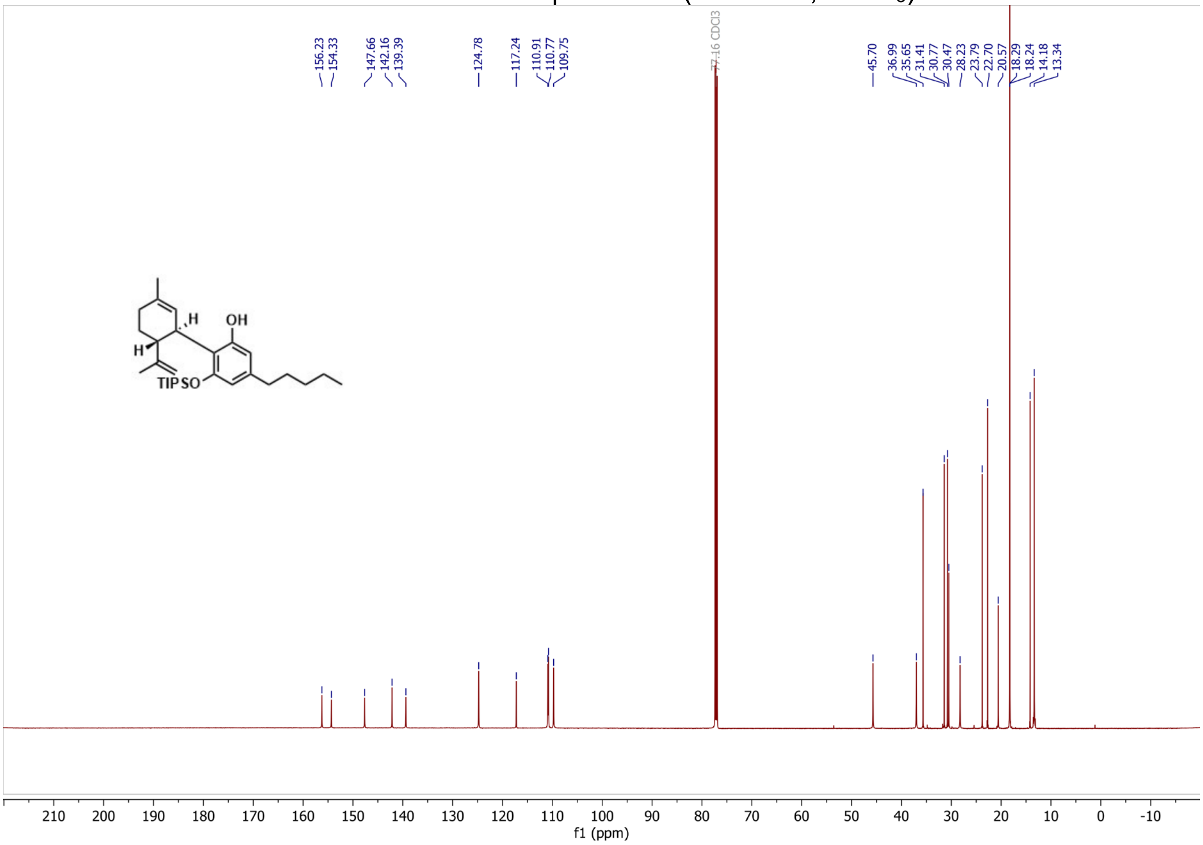

^{1}H NMR of Compound **4c** (600 MHz, $CDCl_3$)

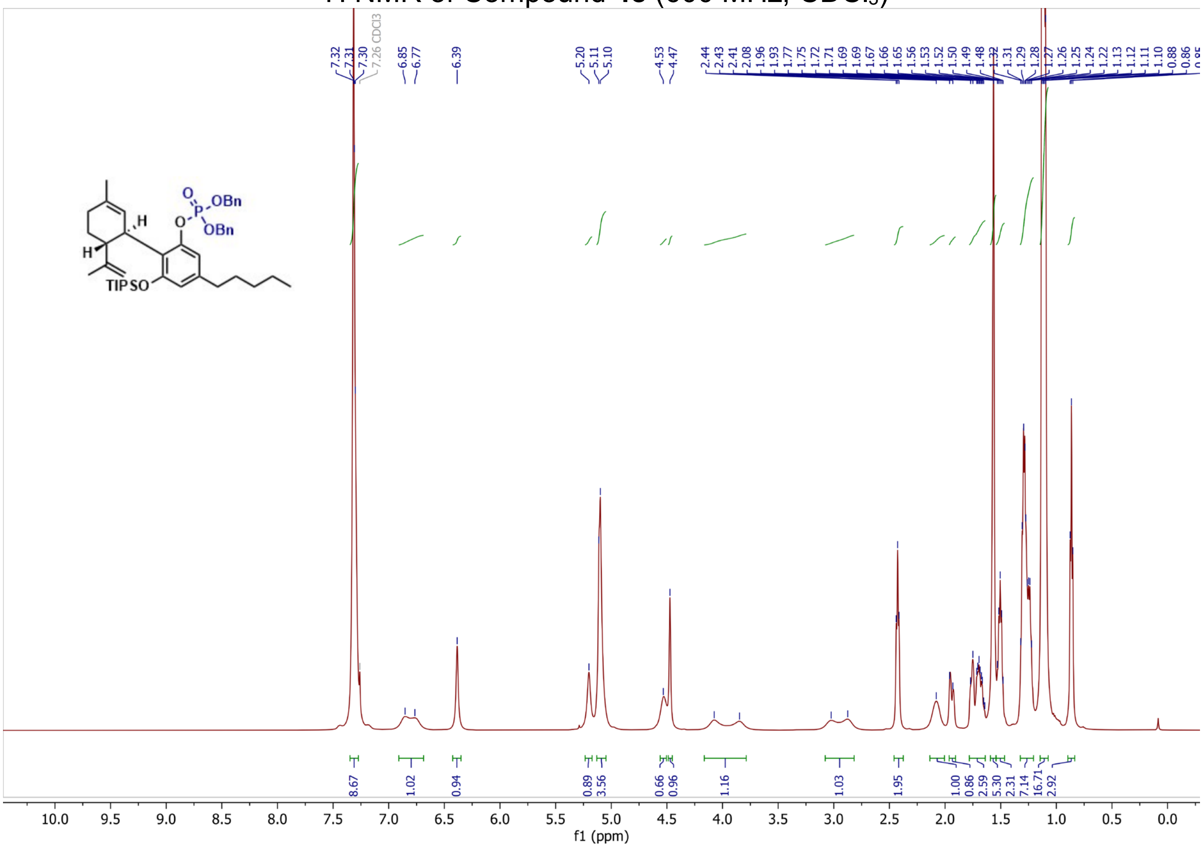

^{13}C NMR of Compound **4c** (151 MHz, $CDCl_3$)

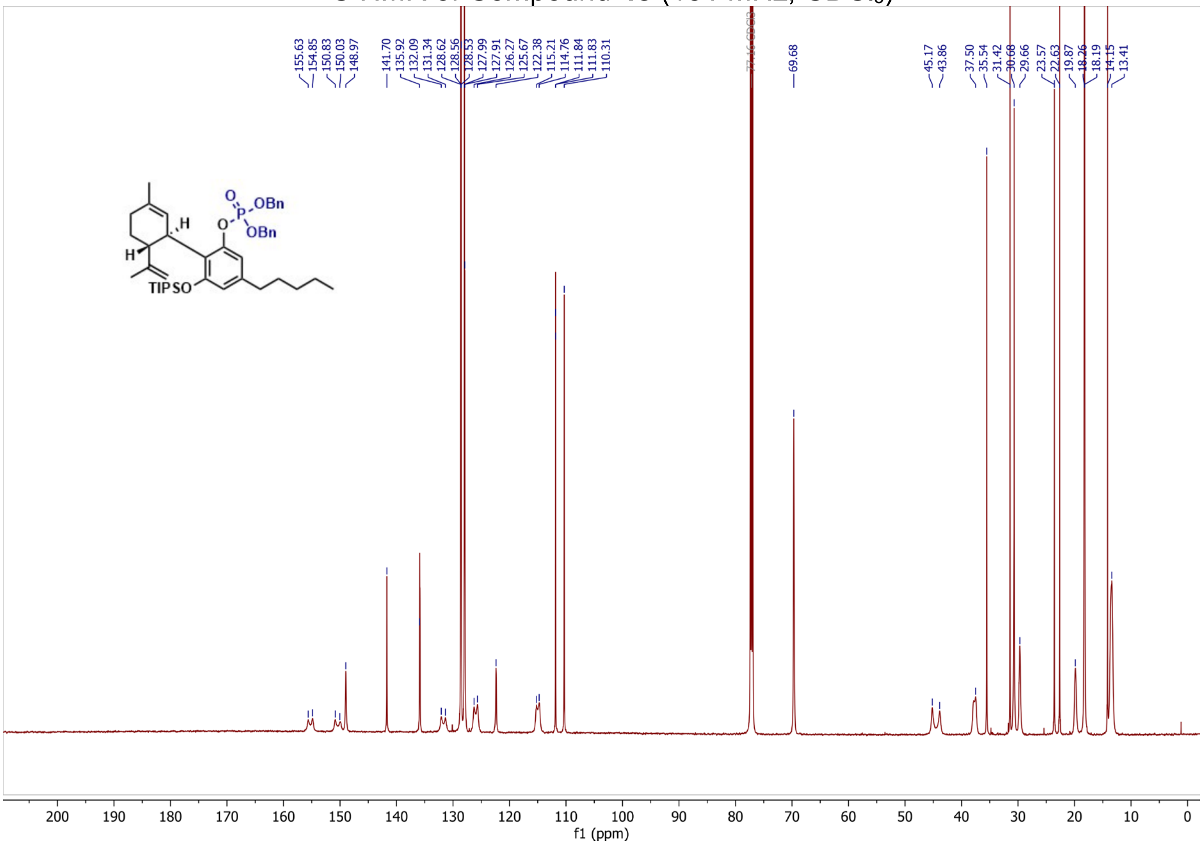

^{31}P NMR of Compound **4c** (243 MHz, $CDCl_3$)

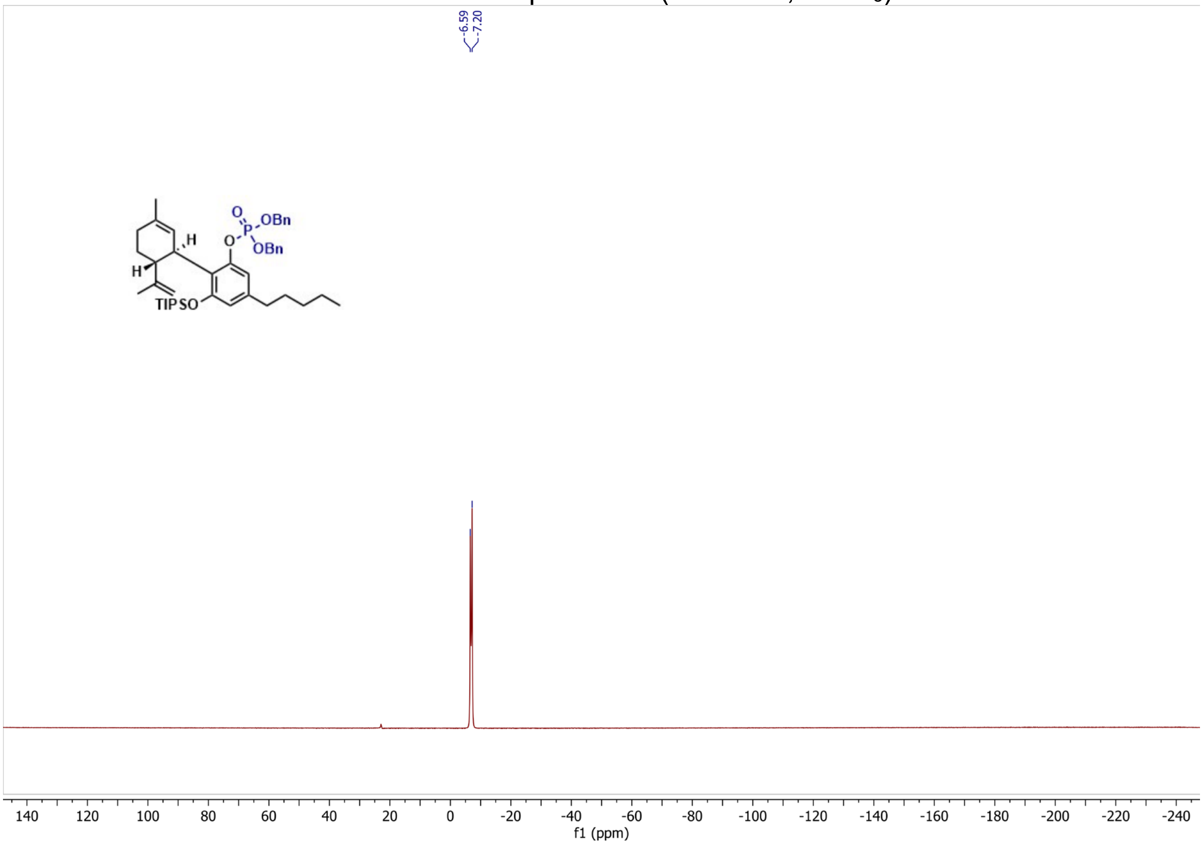

^{1}H NMR of Compound **5** (600 MHz, $CDCl_3$)

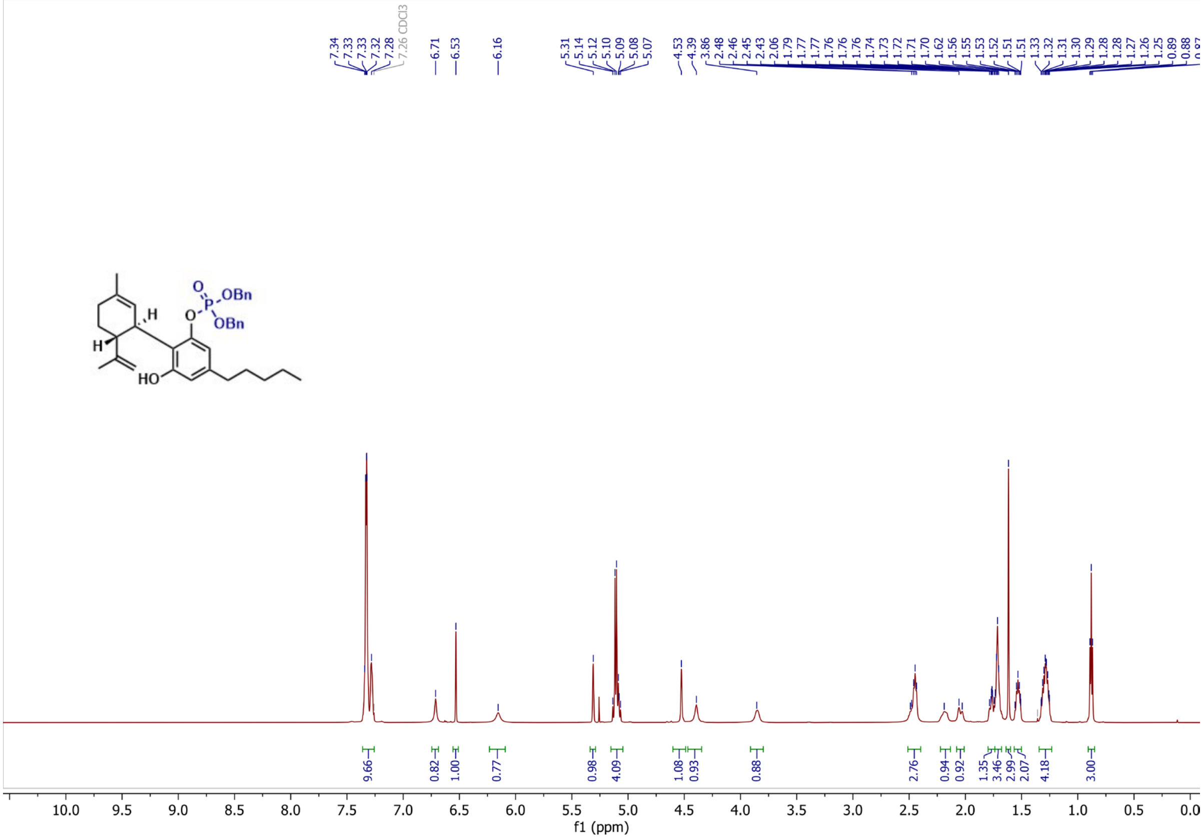

^{13}C NMR of Compound **5** (151 MHz, $CDCl_3$)

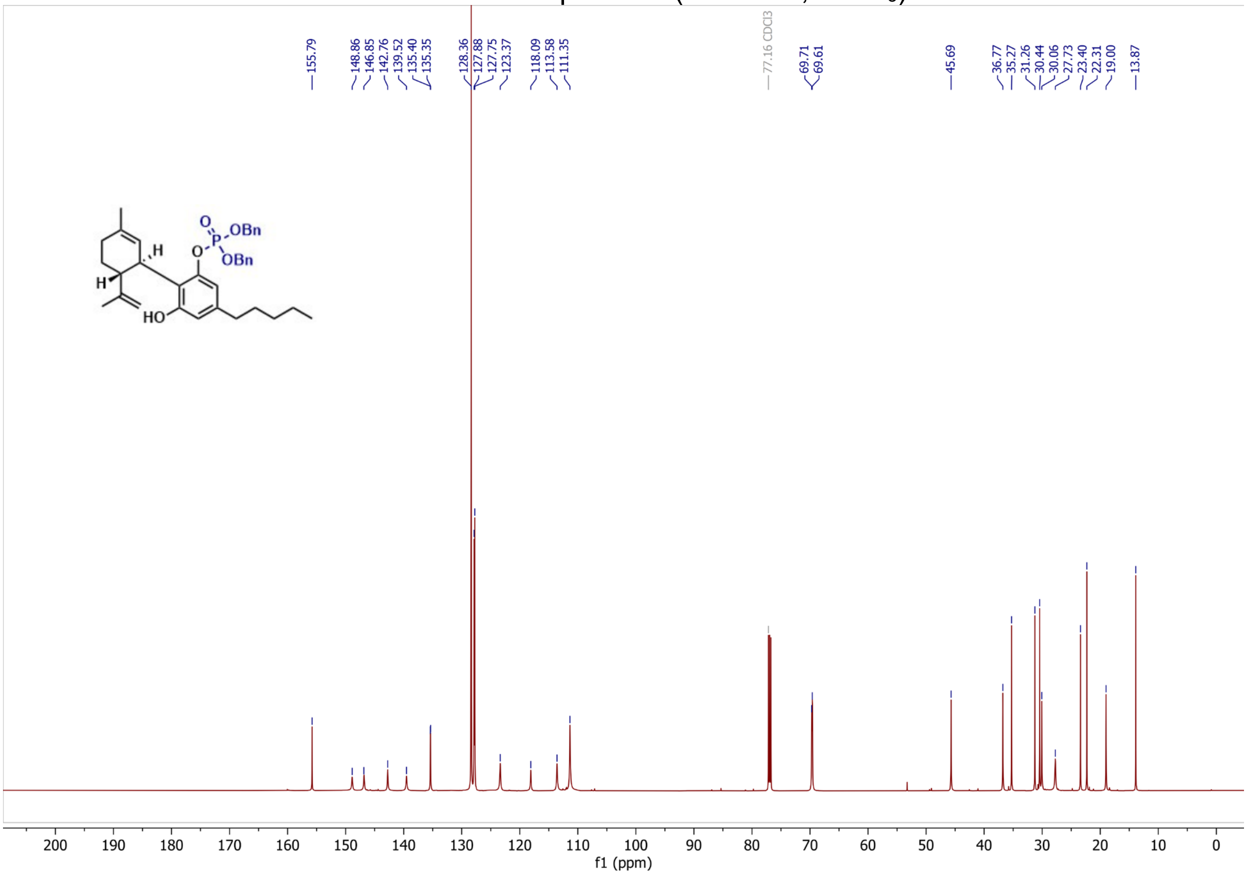

^{31}P NMR of Compound **5** (243 MHz, $CDCl_3$)

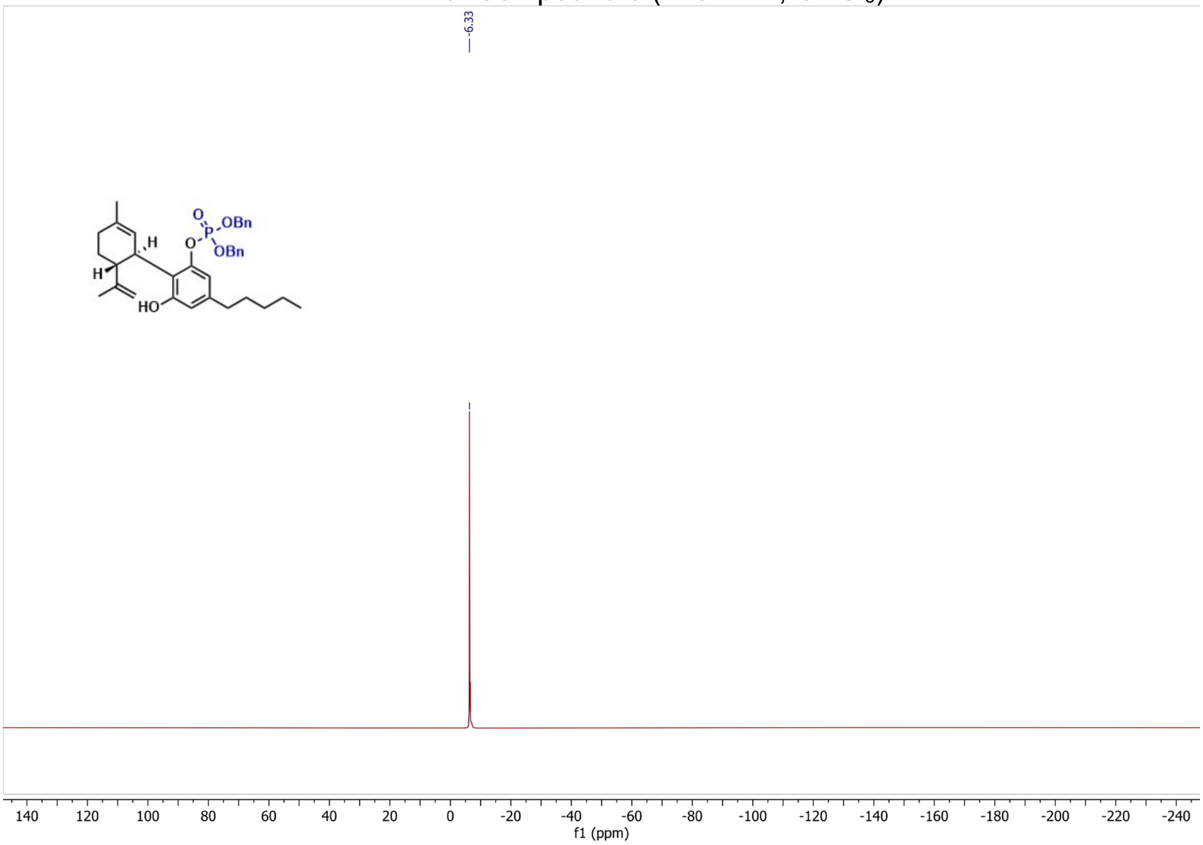

^{1}H NMR of Compound **2** (600 MHz, $CDCl_3$)

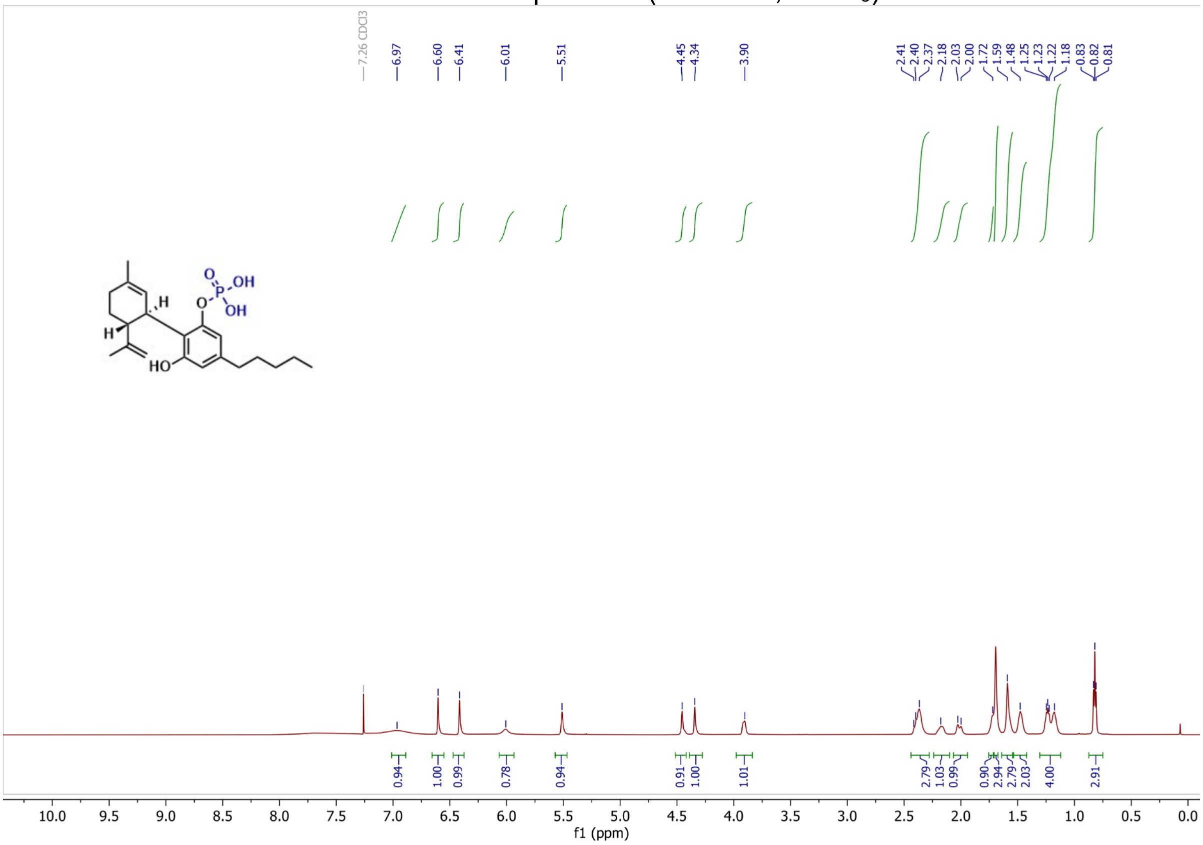

^{13}C NMR of Compound **2** (151 MHz, $CDCl_3$)

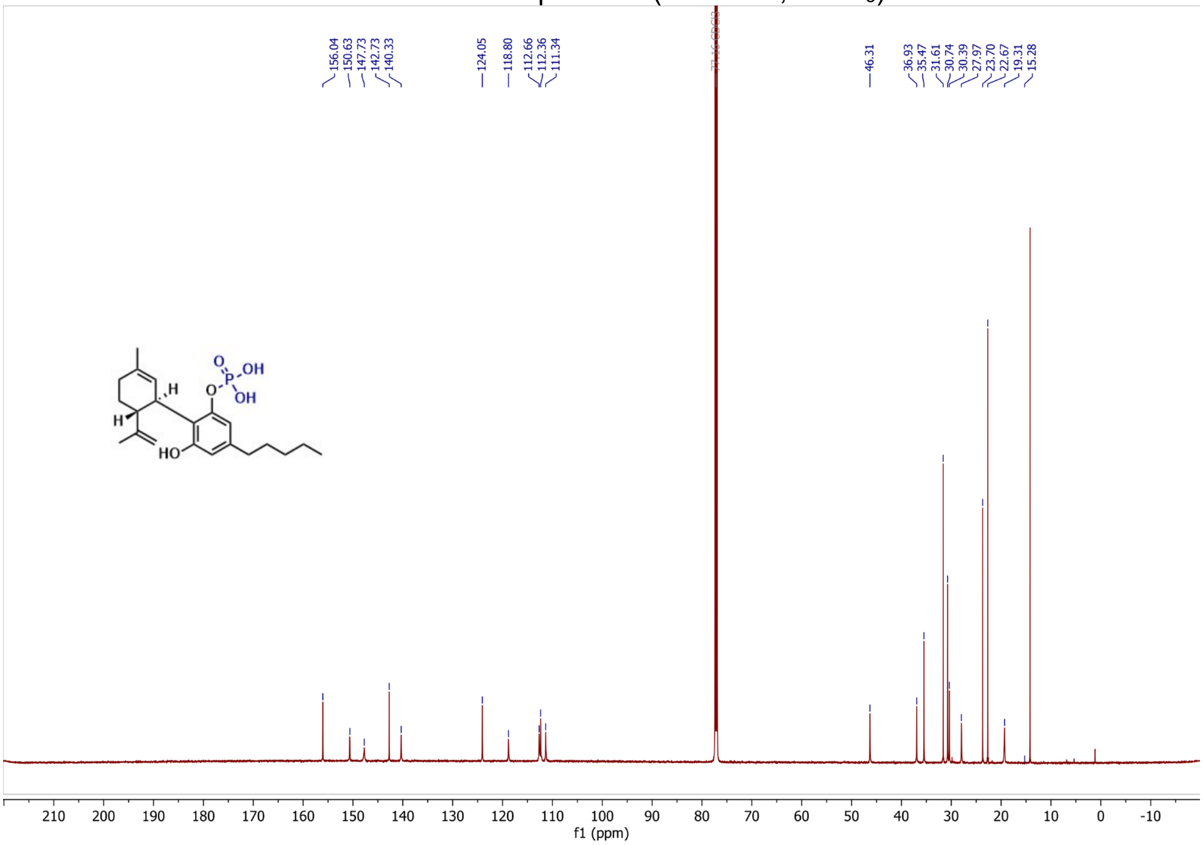

^{31}P NMR of Compound **2** (243 MHz, $CDCl_3$)

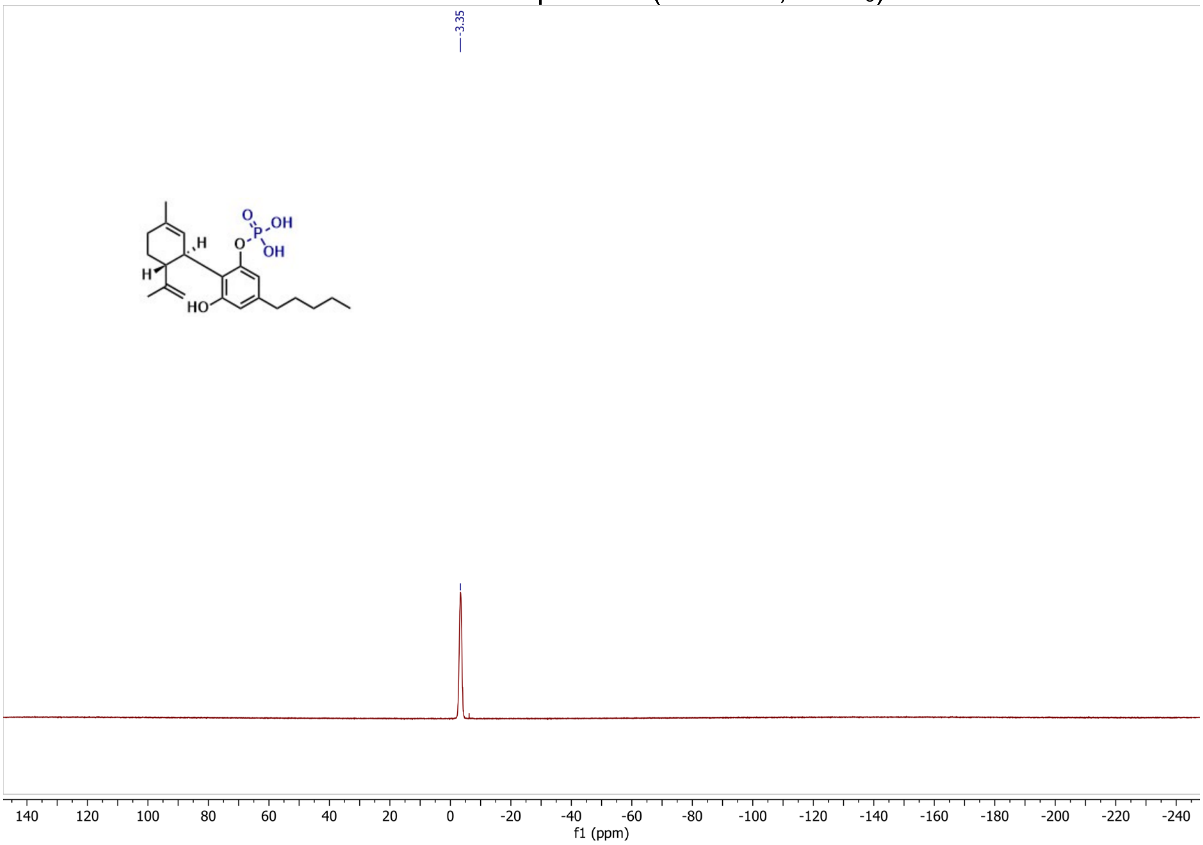

^{1}H NMR of Compound **6** (600 MHz, $CDCl_3$)

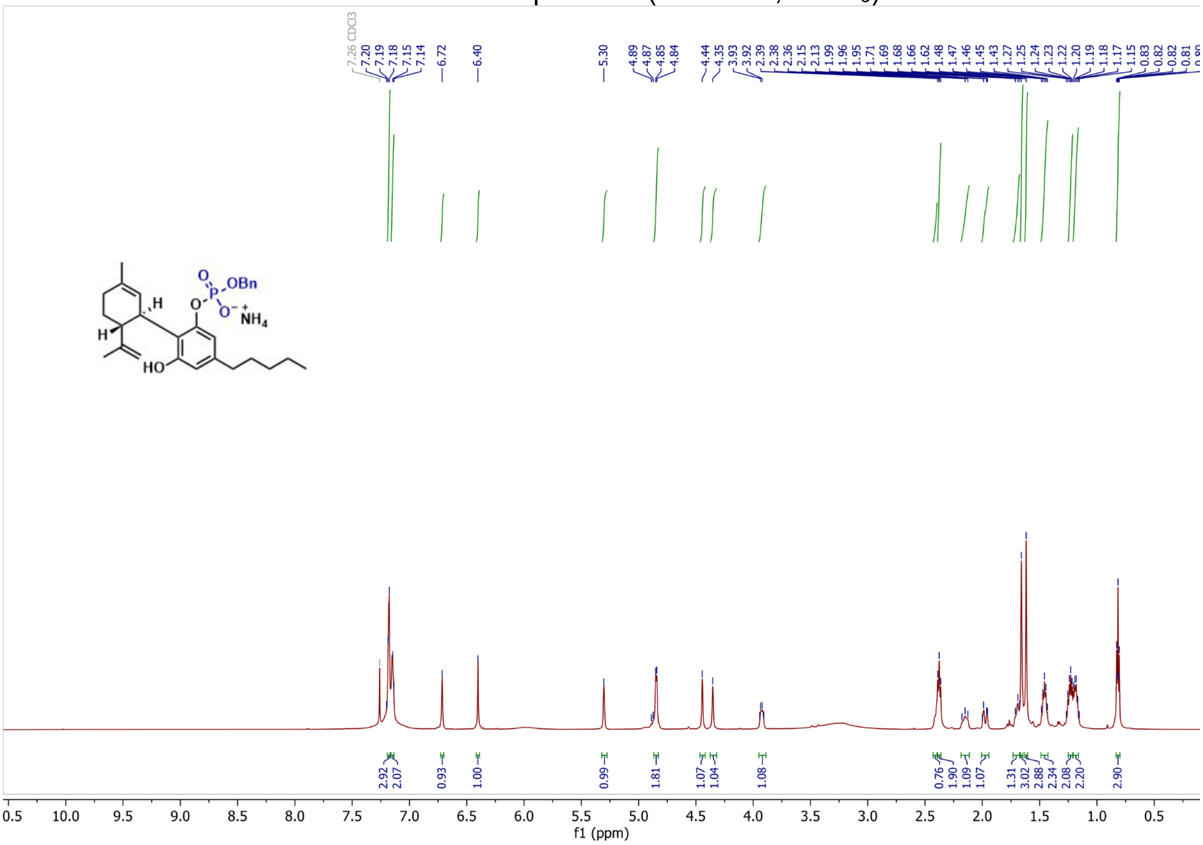

^{13}C NMR of Compound **6** (151 MHz, $CDCl_3$)

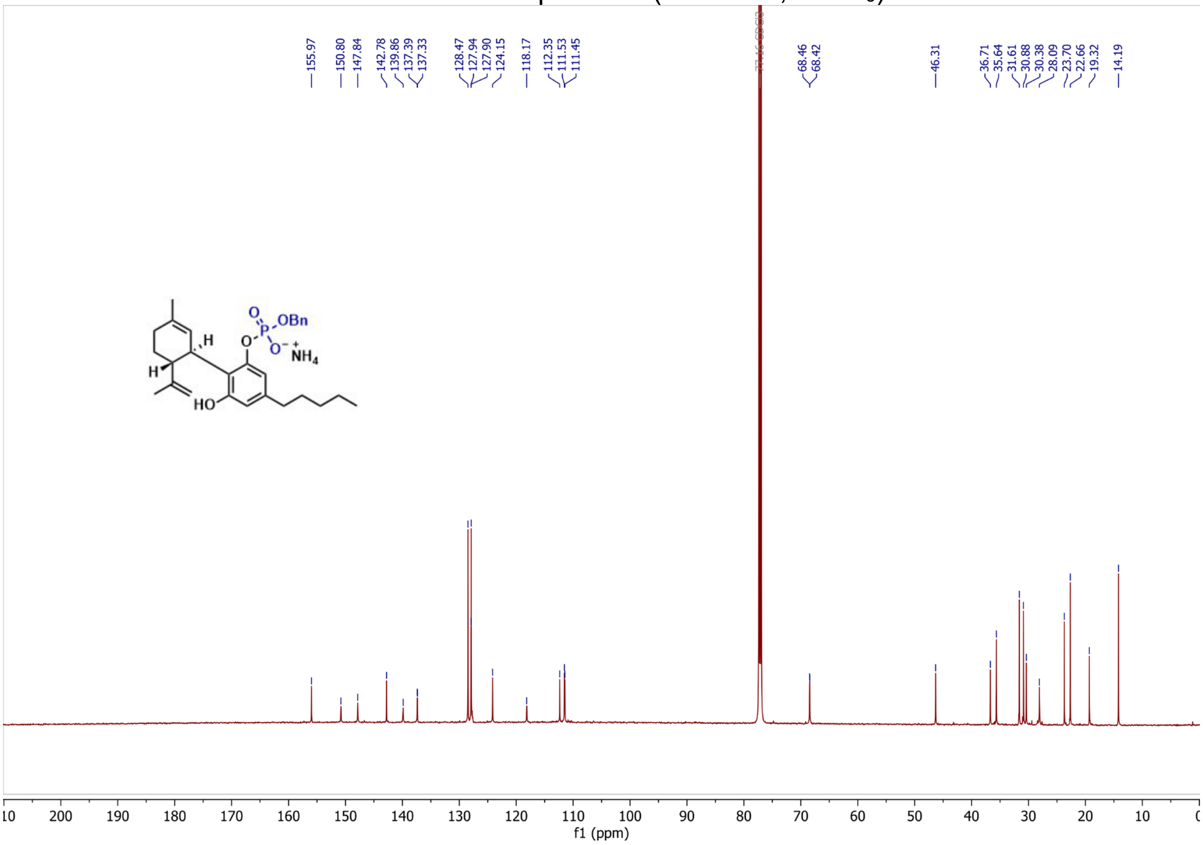

^{31}P NMR of Compound **6** (243 MHz, $CDCl_3$)

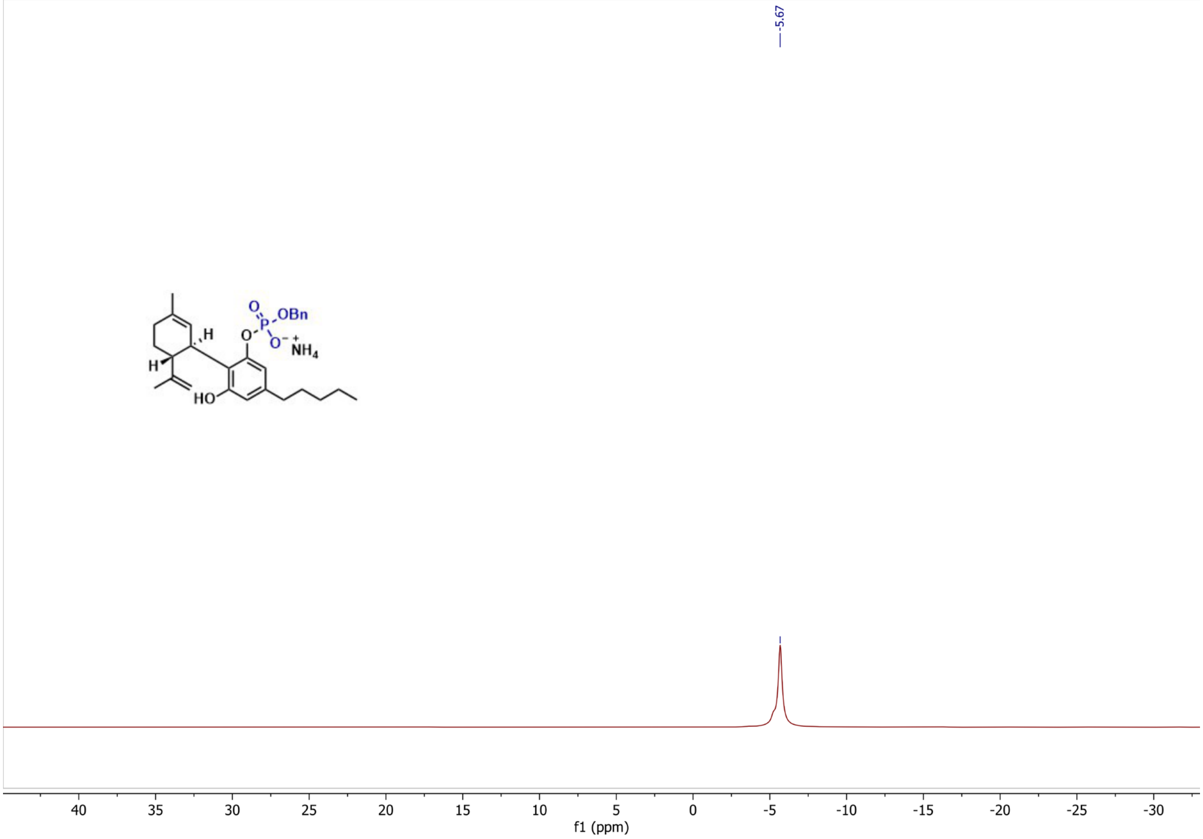

^{1}H NMR of Compound **7a** (400 MHz, $CDCl_3$)

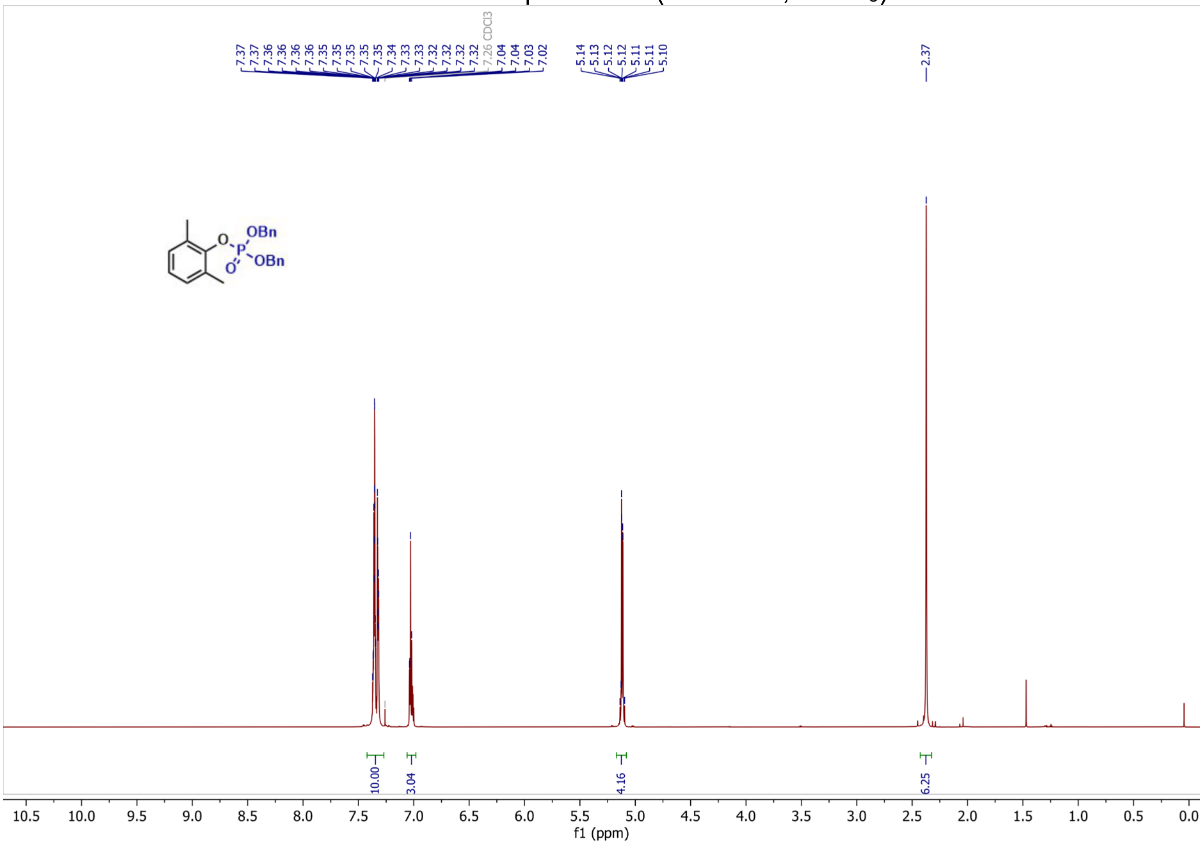

^{13}C NMR of Compound **7a** (201 MHz, $CDCl_3$)

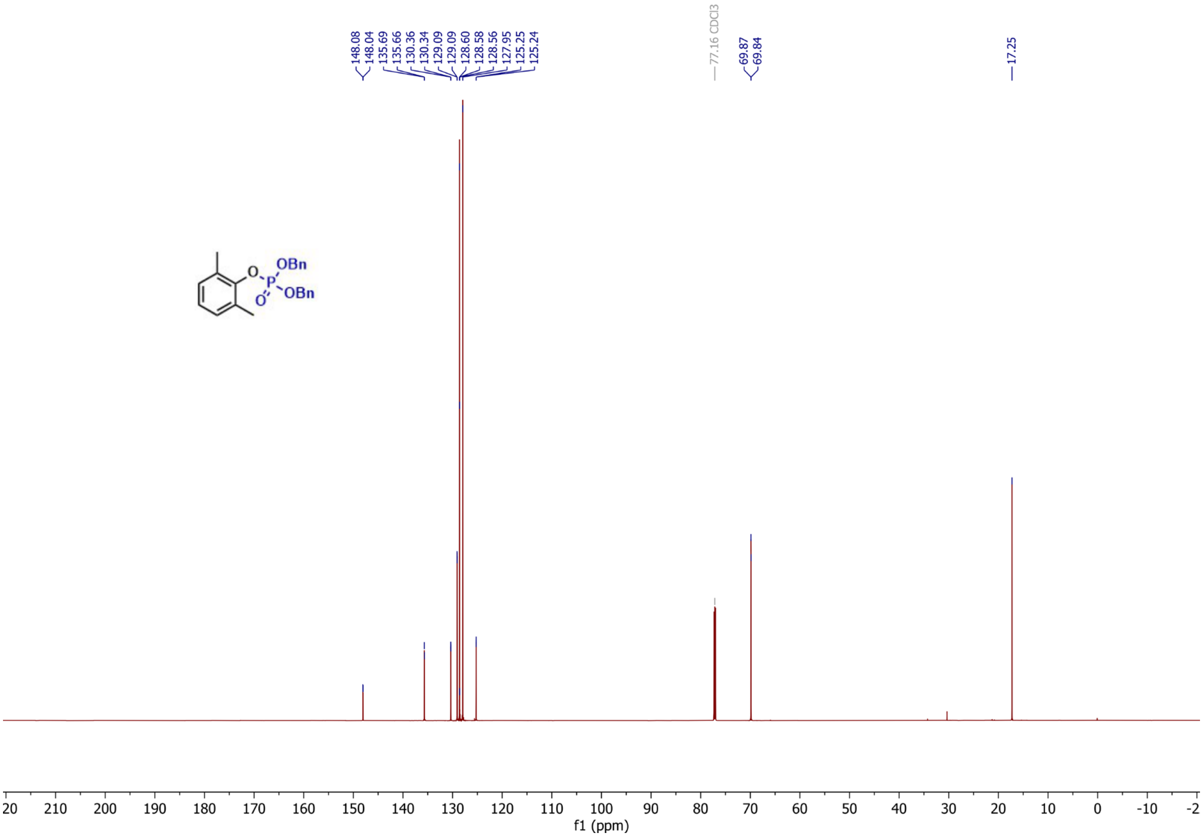

^{31}P NMR of Compound **7a** (162 MHz, $CDCl_3$)

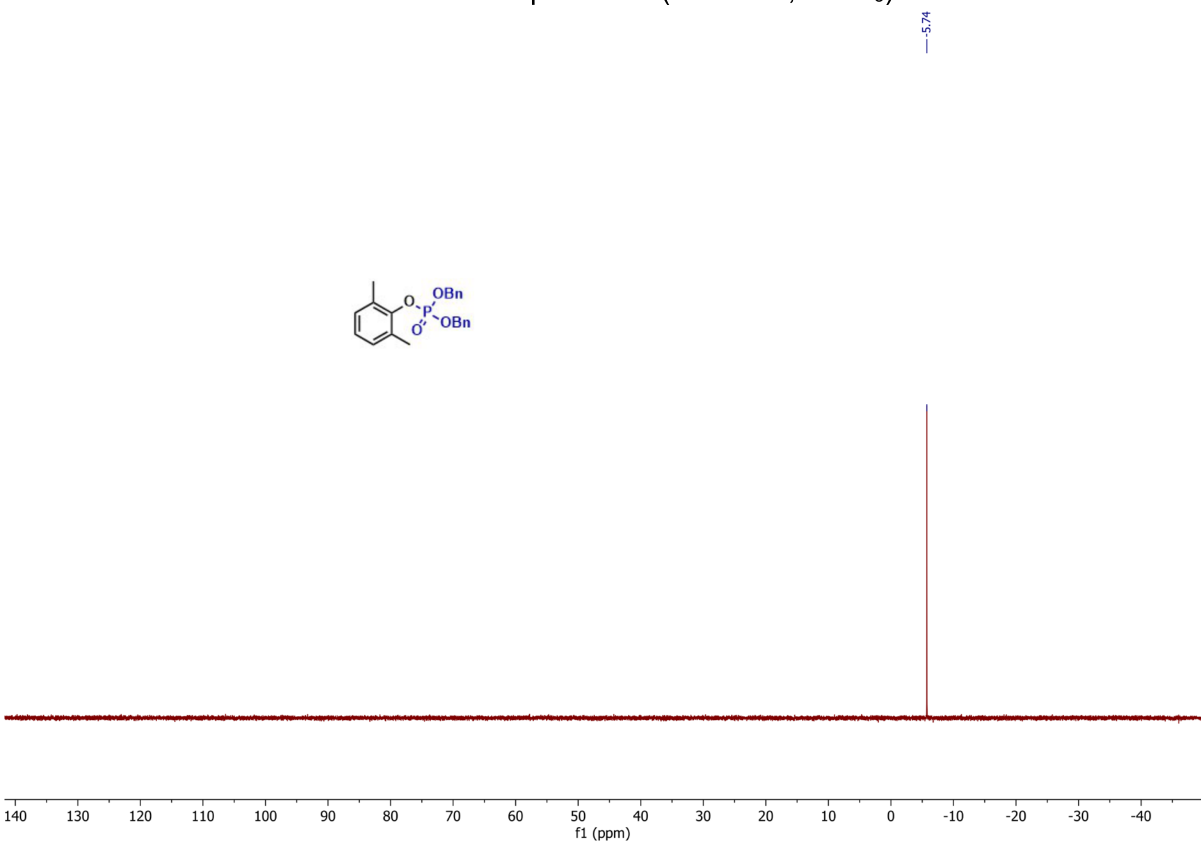

^{1}H NMR of Compound **7b** (400 MHz, DMSO-d_6)

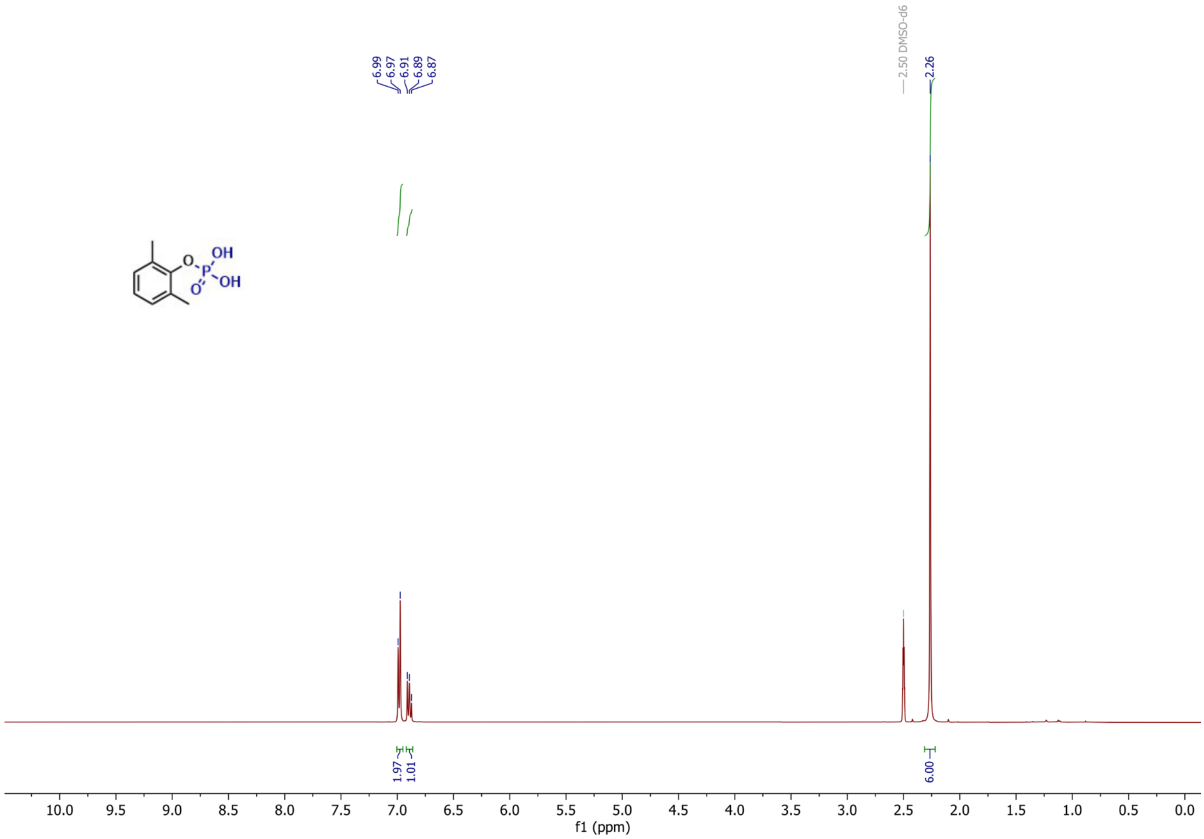

^{13}C NMR of Compound **7b** (101 MHz, DMSO-d_6)

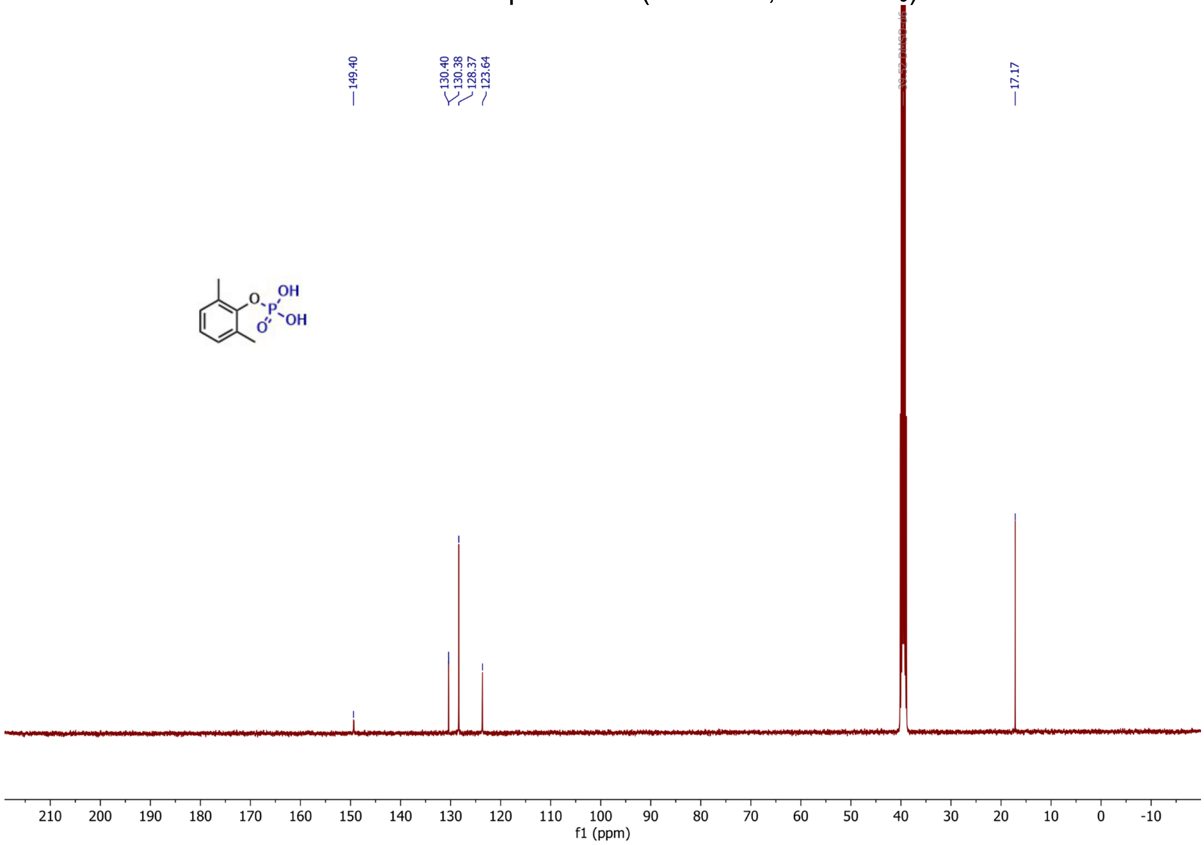

^{31}P NMR of Compound **7b** (162 MHz, DMSO-d_6)

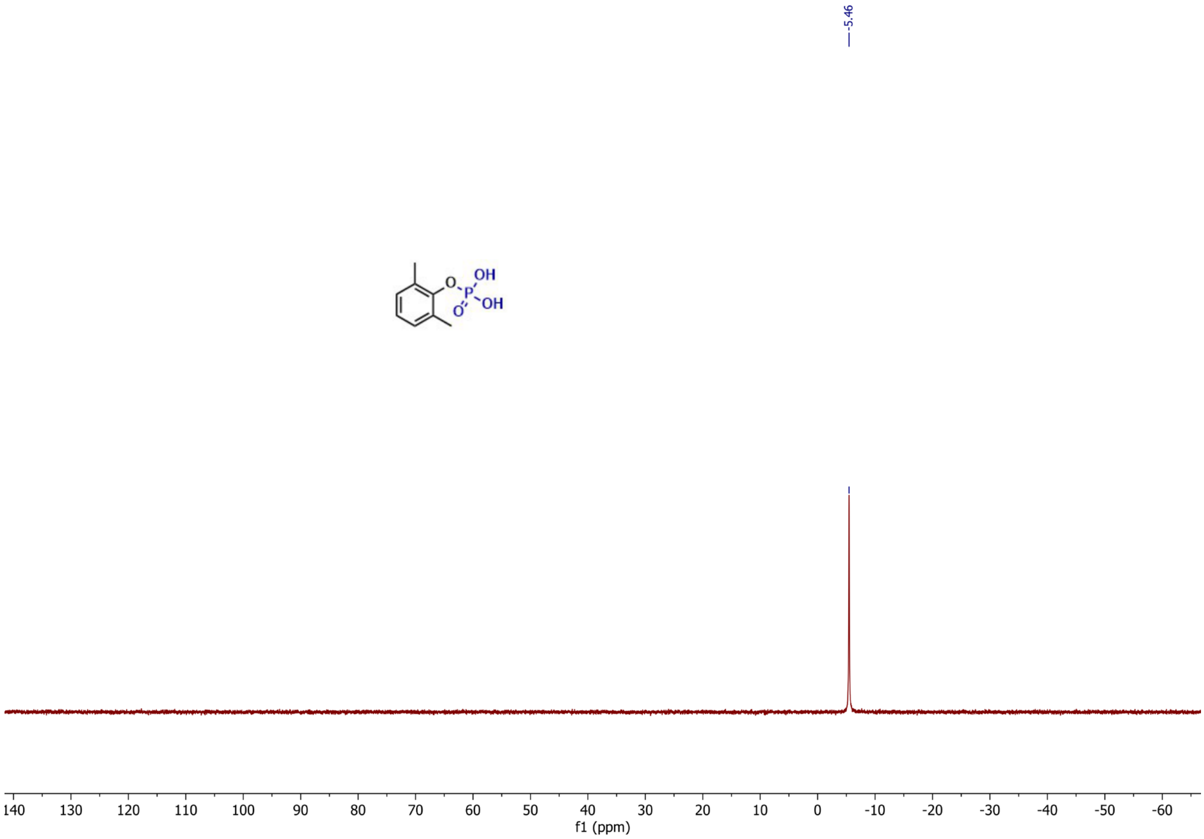

^{1}H NMR of Compound **8a** (400 MHz, $CDCl_3$)

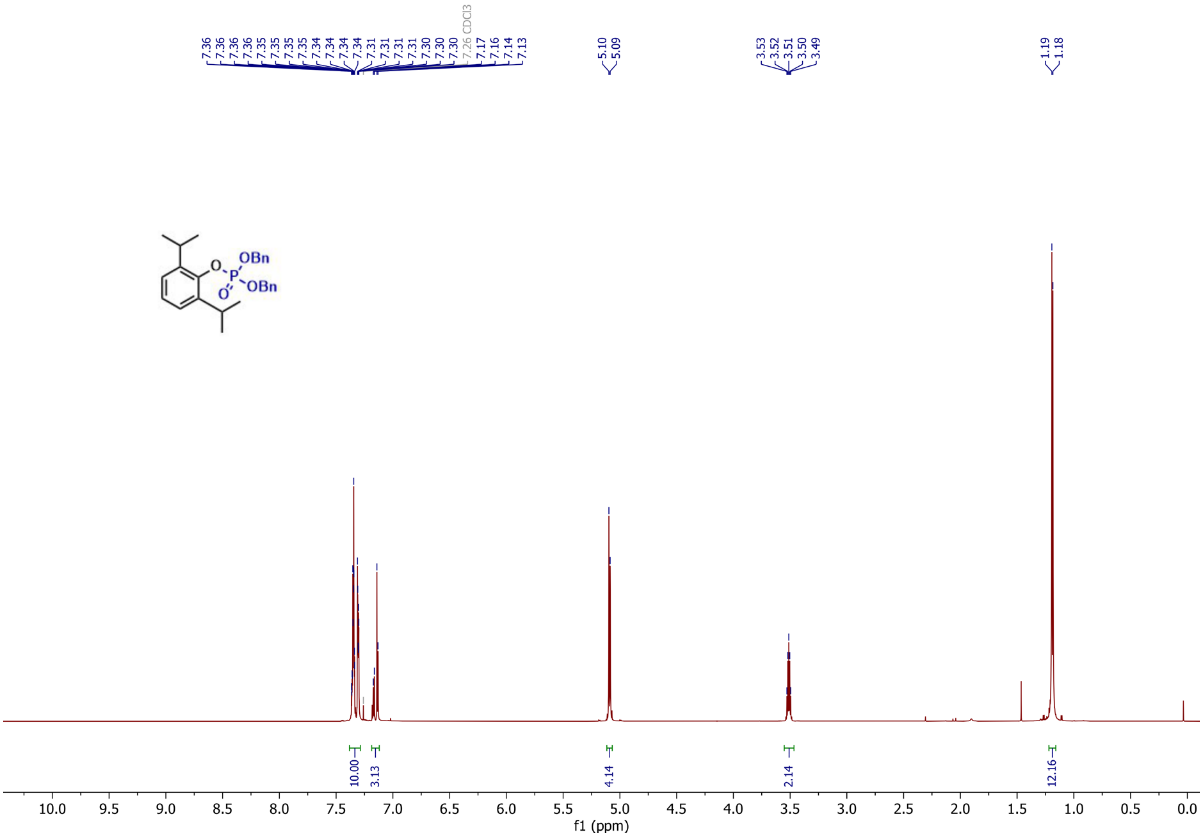

^{13}C NMR of Compound **8a** (125 MHz, $CDCl_3$)

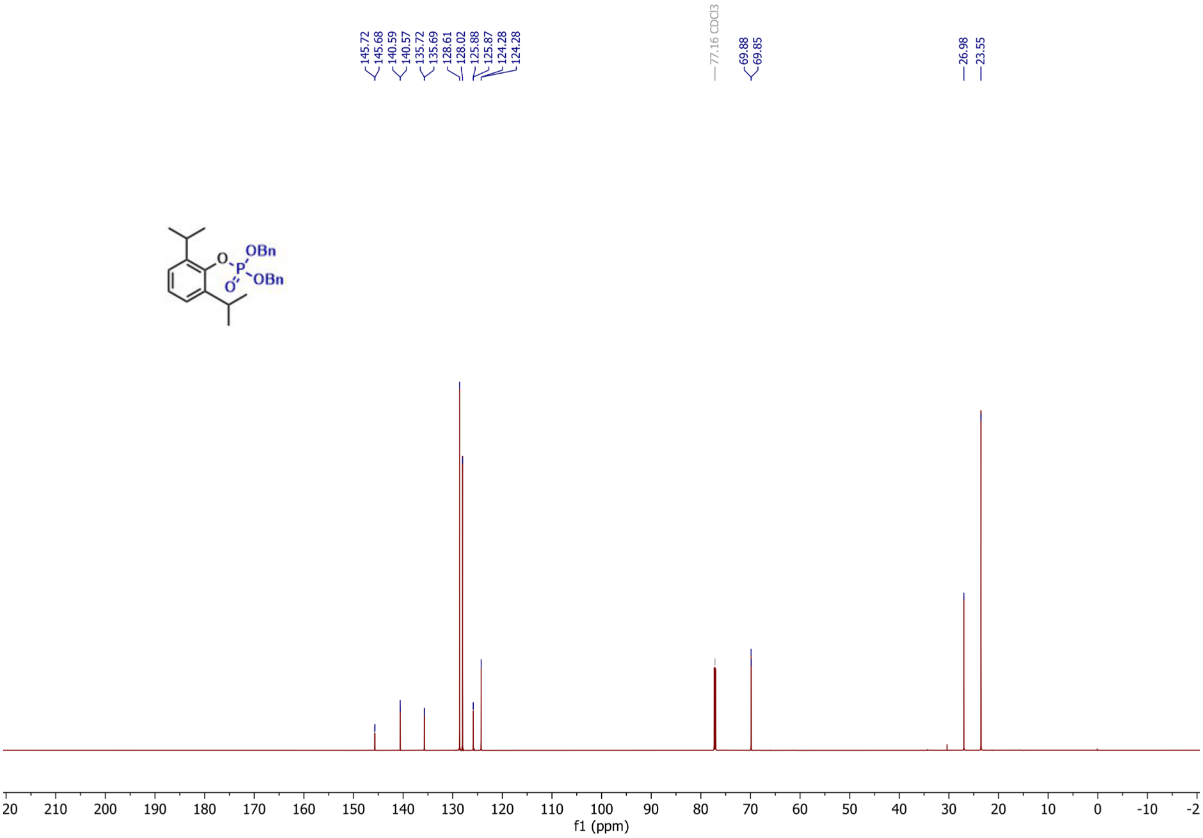

^{31}P NMR of Compound **8a** (162 MHz, $CDCl_3$)

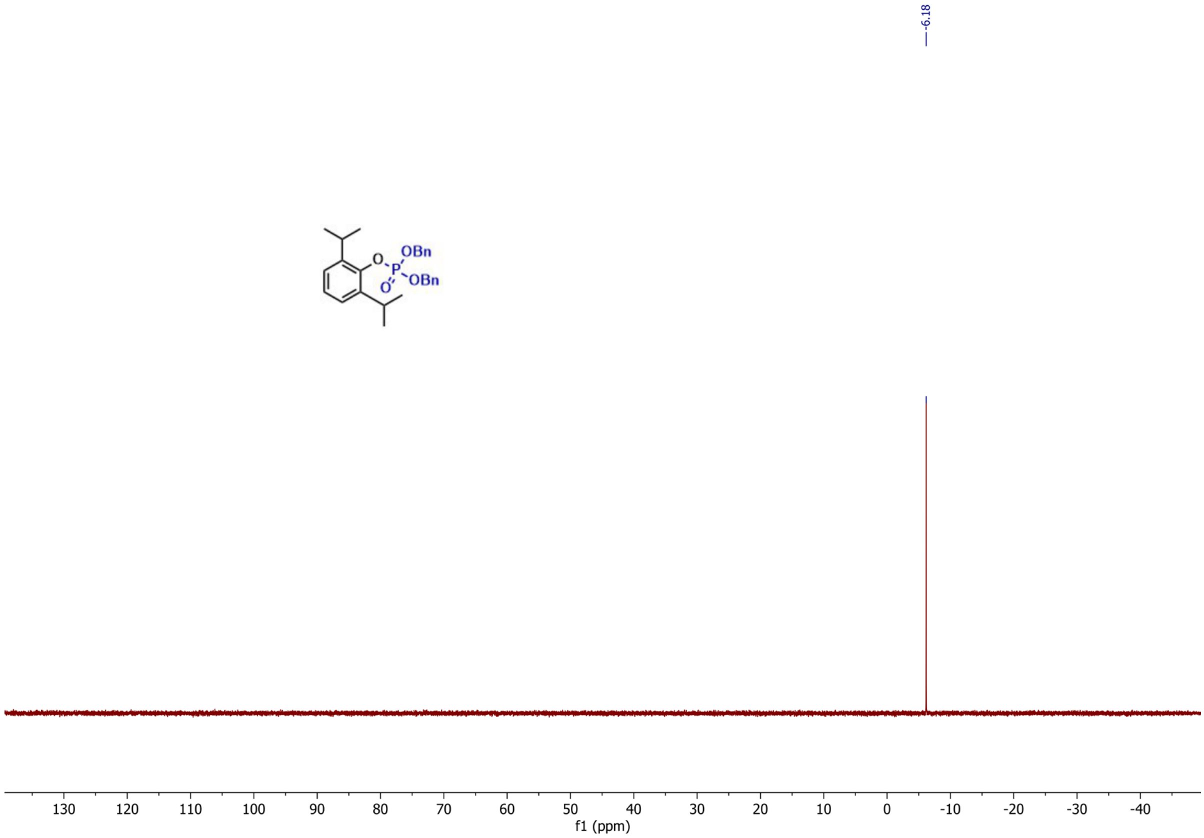

^{1}H NMR of Compound **8b** (400 MHz, DMSO-d_6)

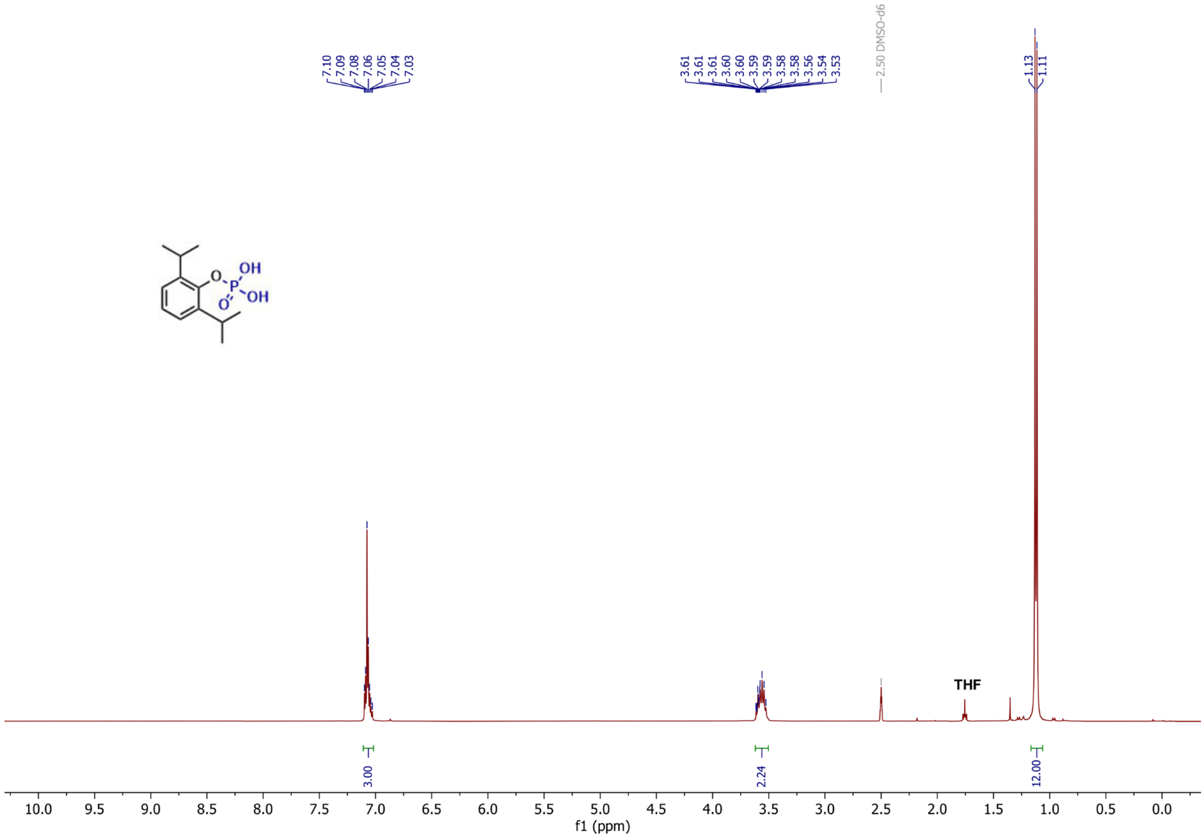

^{13}C NMR of Compound **8b** (101 MHz, DMSO-d_6)

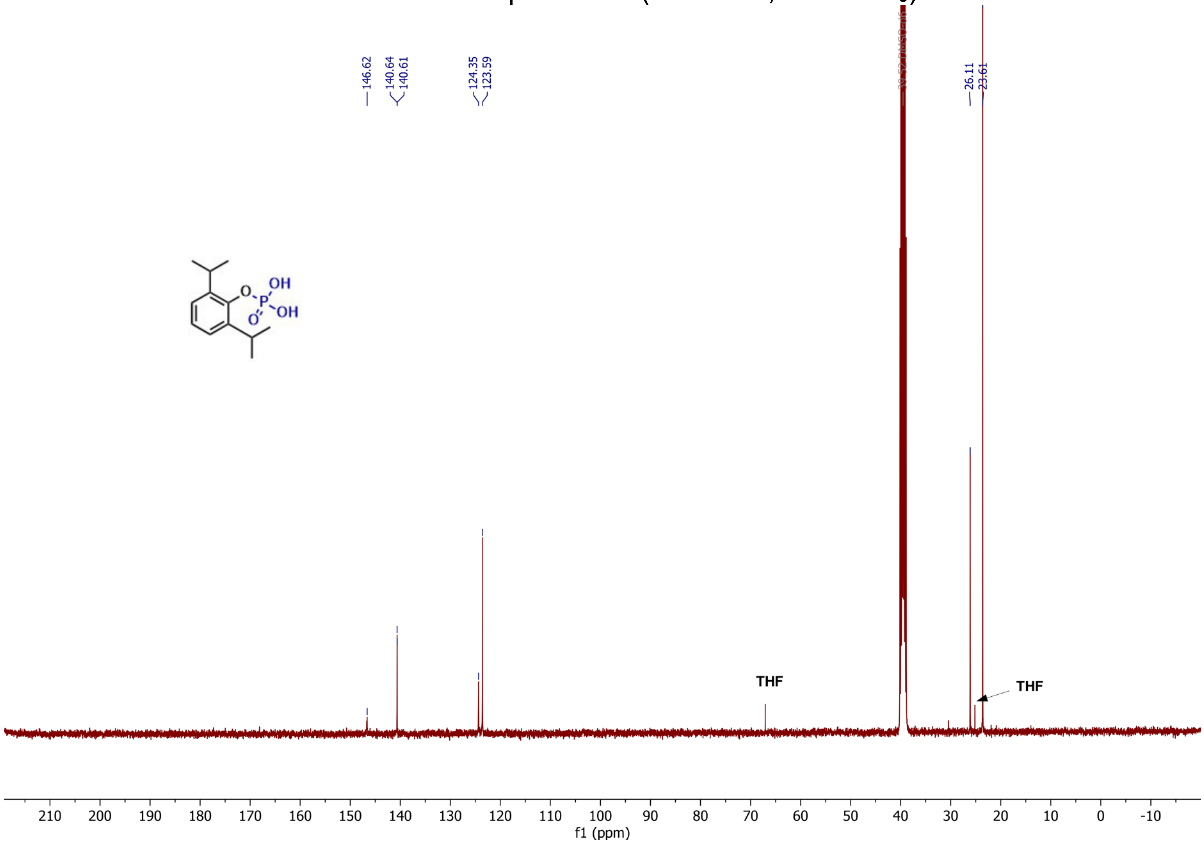

^{31}P NMR of Compound **8b** (162 MHz, DMSO-d_6)

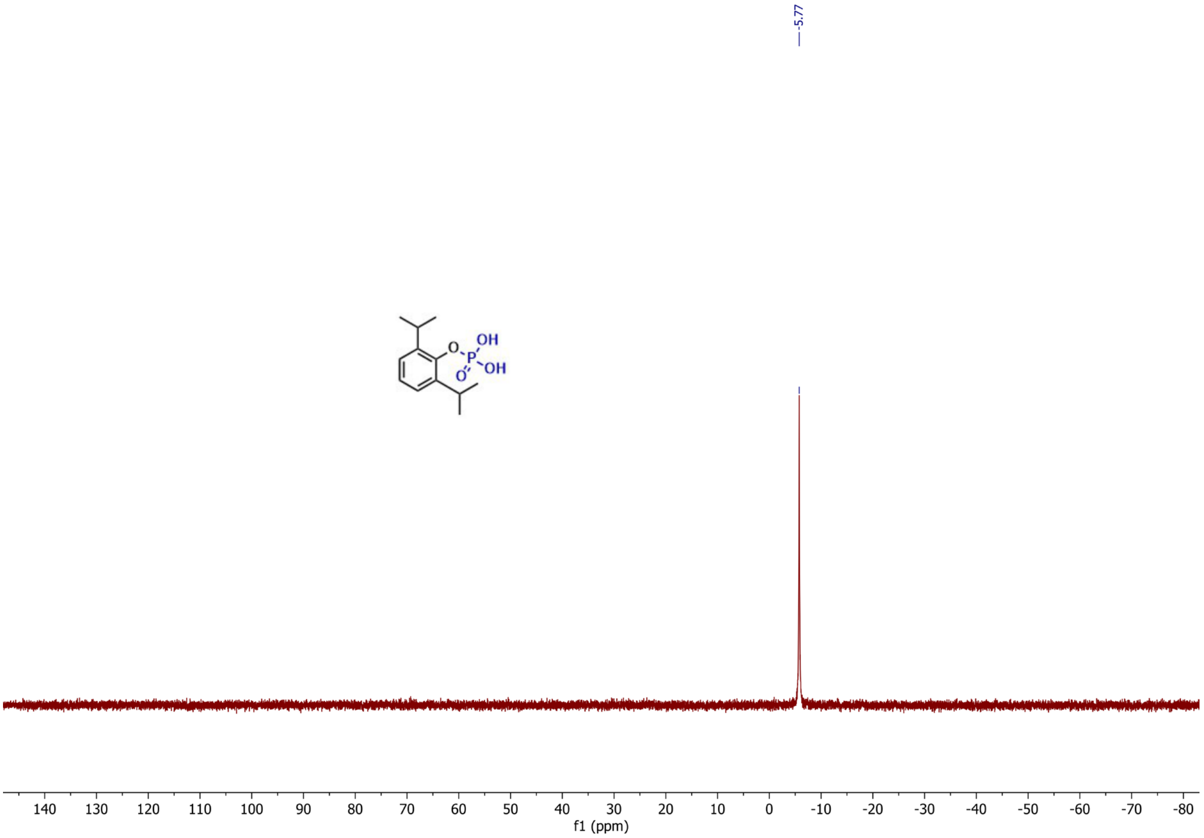

^{1}H NMR of Compound **9a** (400 MHz, $CDCl_3$)

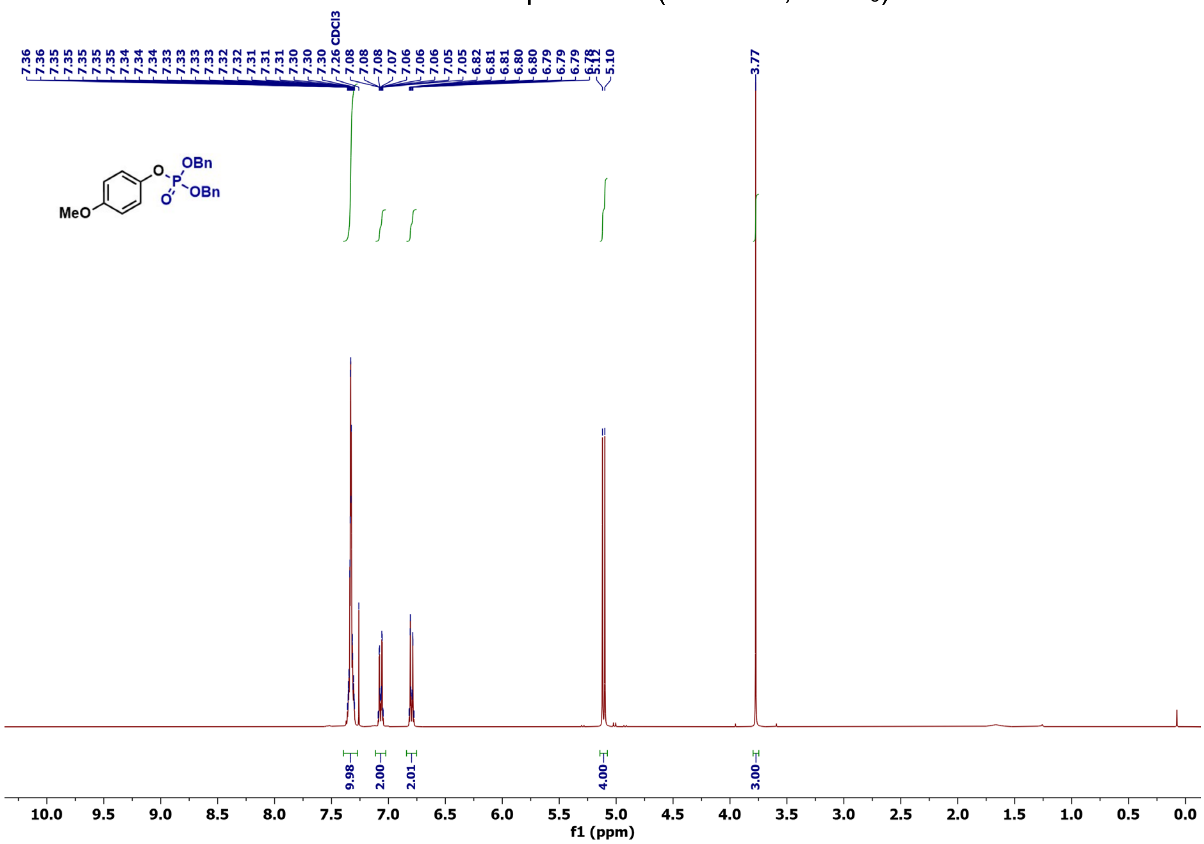

^{13}C NMR of Compound **9a** (101 MHz, $CDCl_3$)

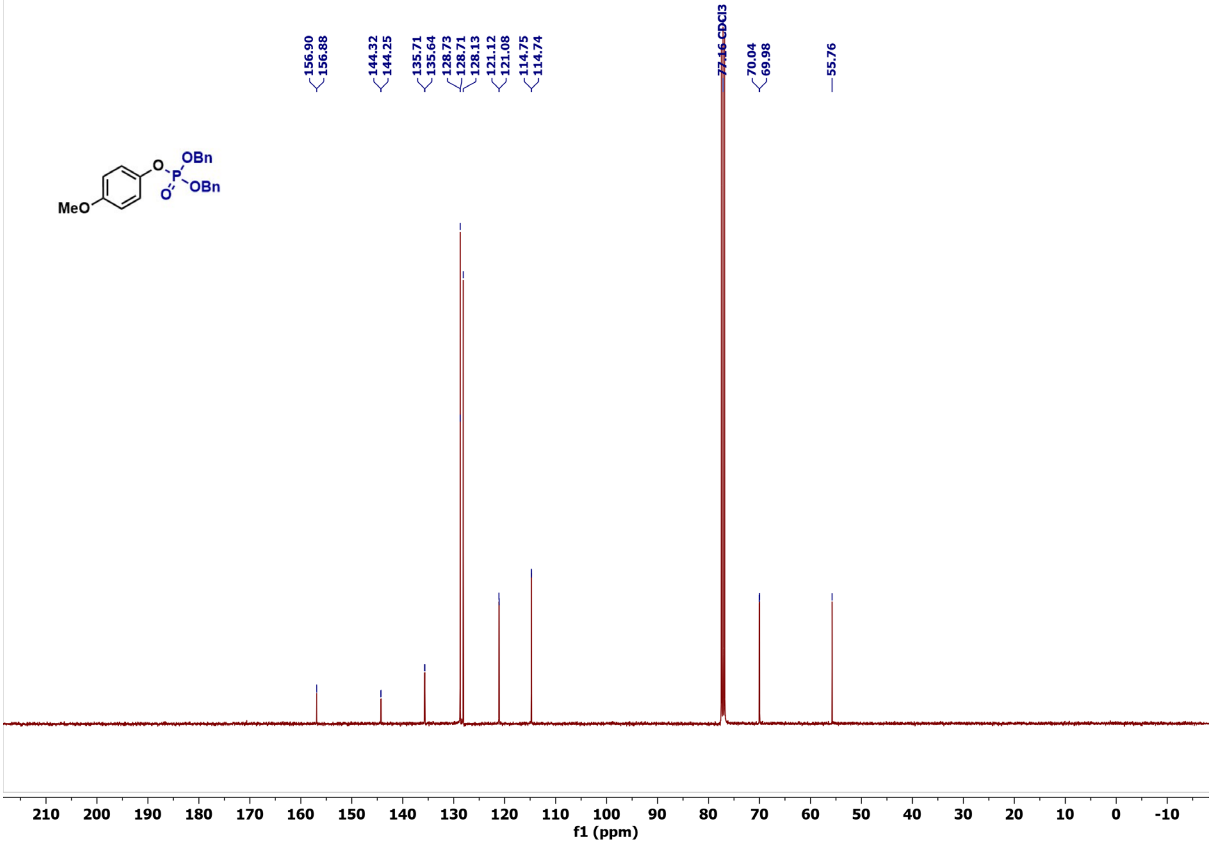

^{31}P NMR of Compound **9a** (162 MHz, $CDCl_3$)

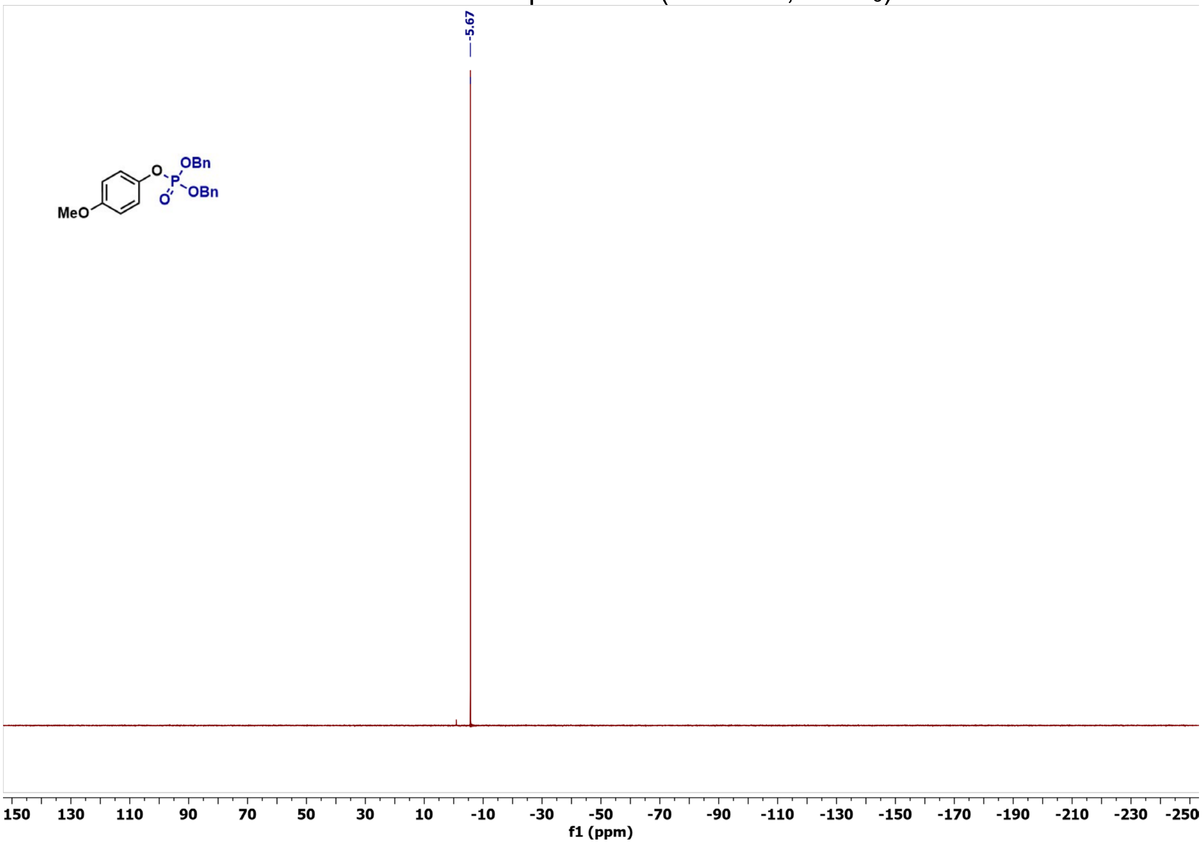

^1H NMR of Compound **9b** (400 MHz, DMSO-d_6)

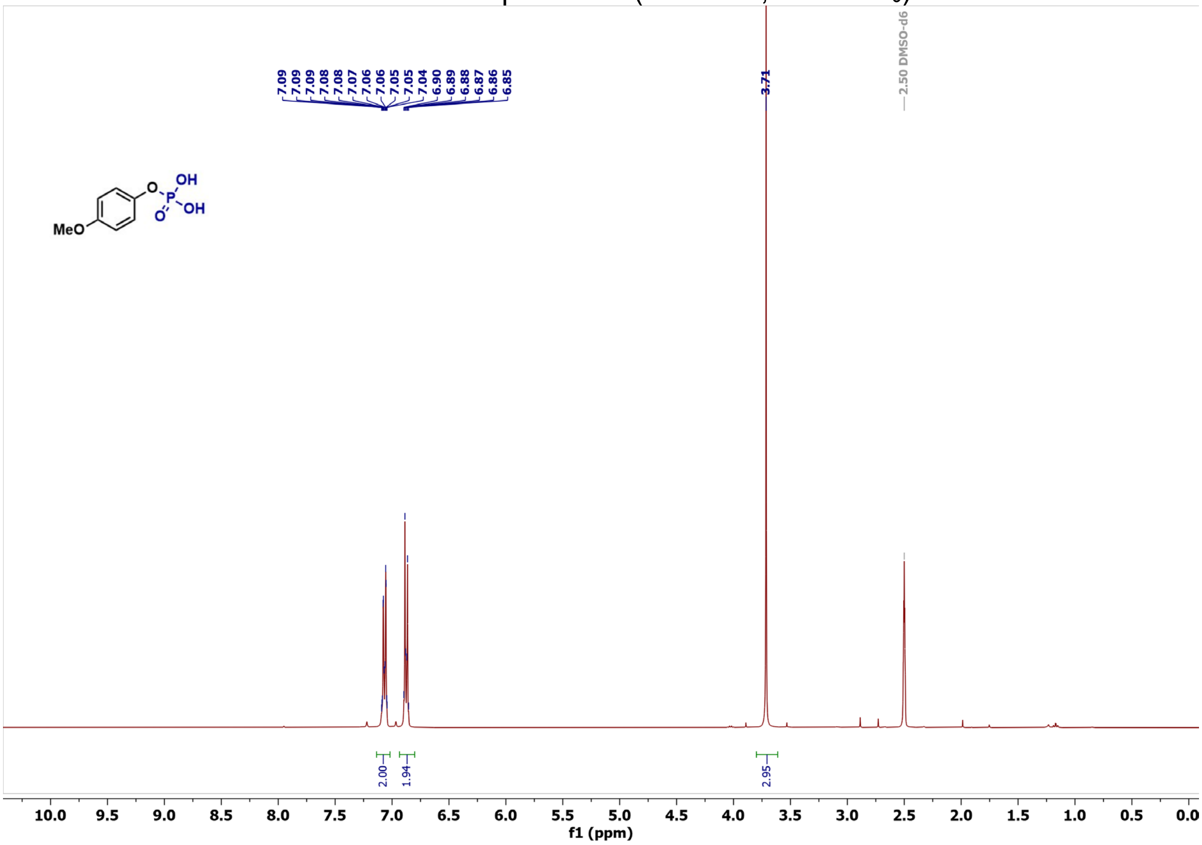

^{13}C NMR of Compound **9b** (101 MHz, DMSO-d_6)

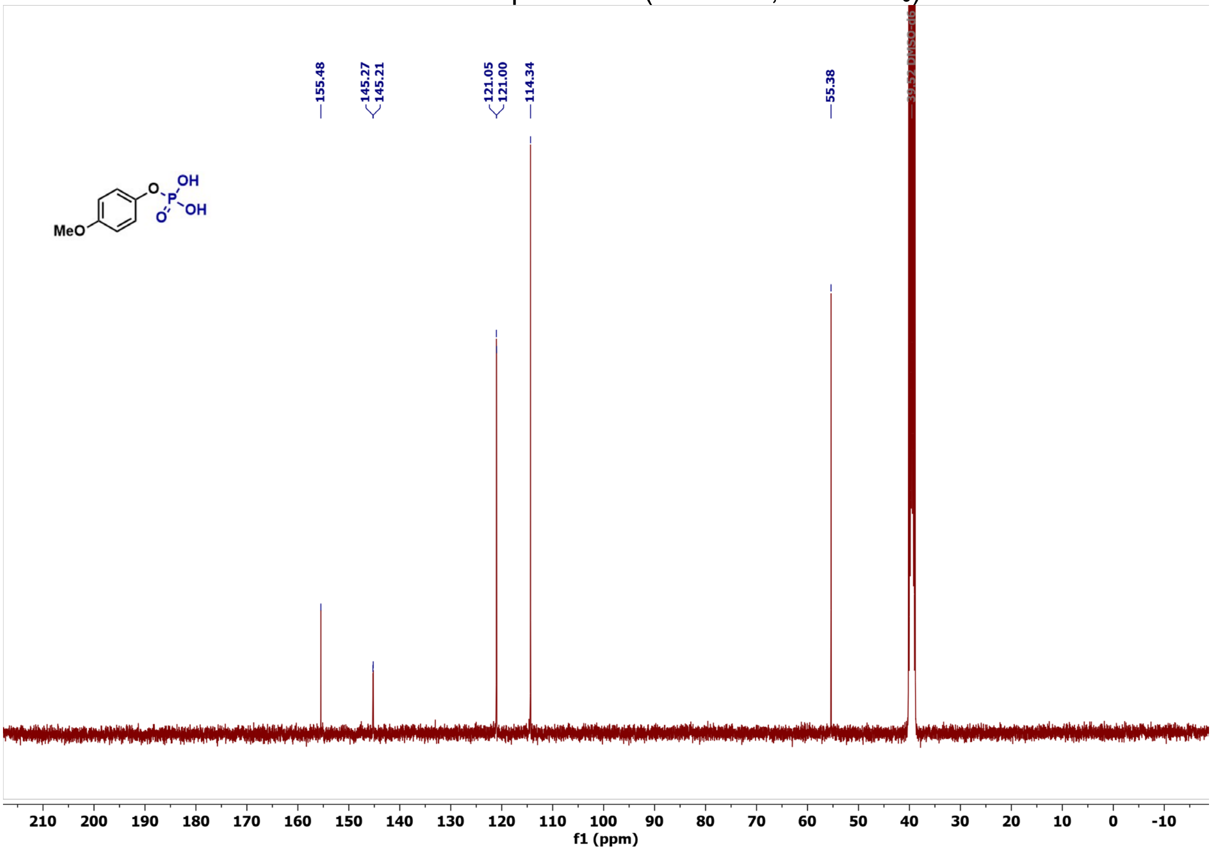

^{31}P NMR of Compound **9b** (162 MHz, DMSO-d_6)

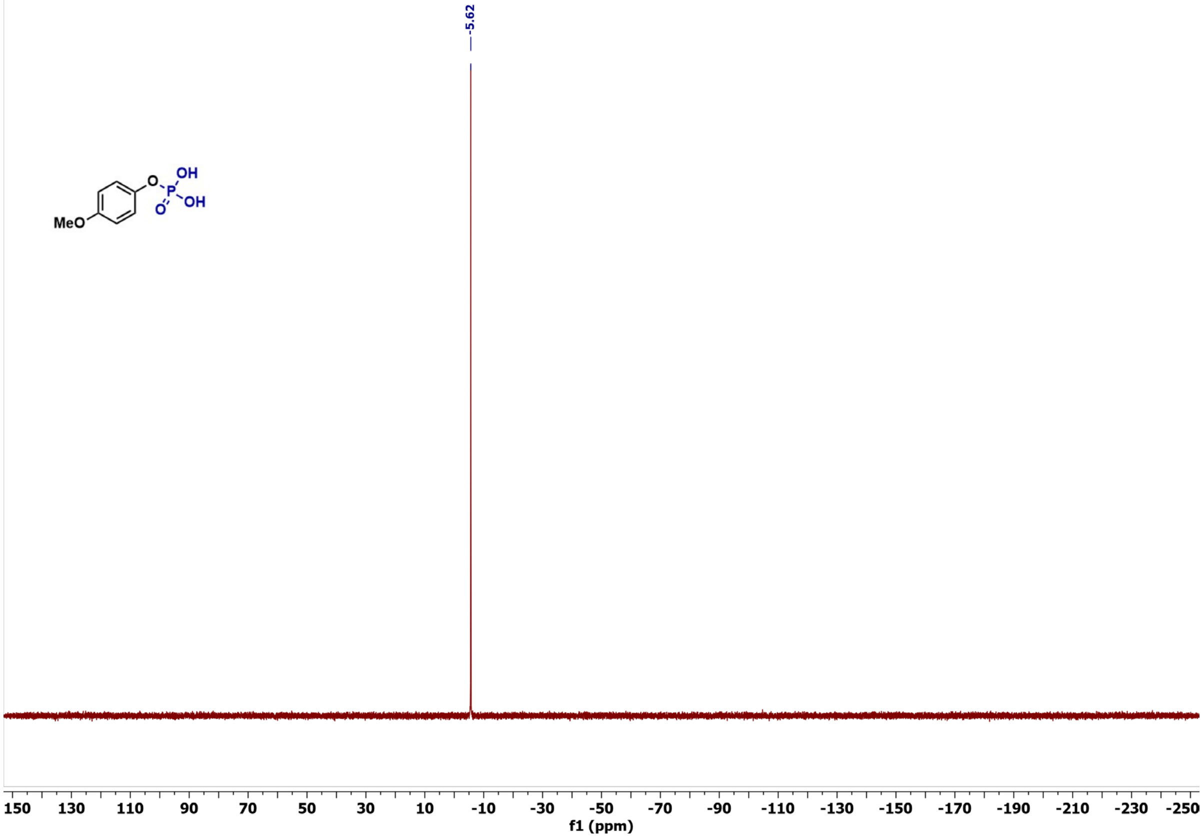

^{1}H NMR of Compound **10a** (400 MHz, $CDCl_3$)

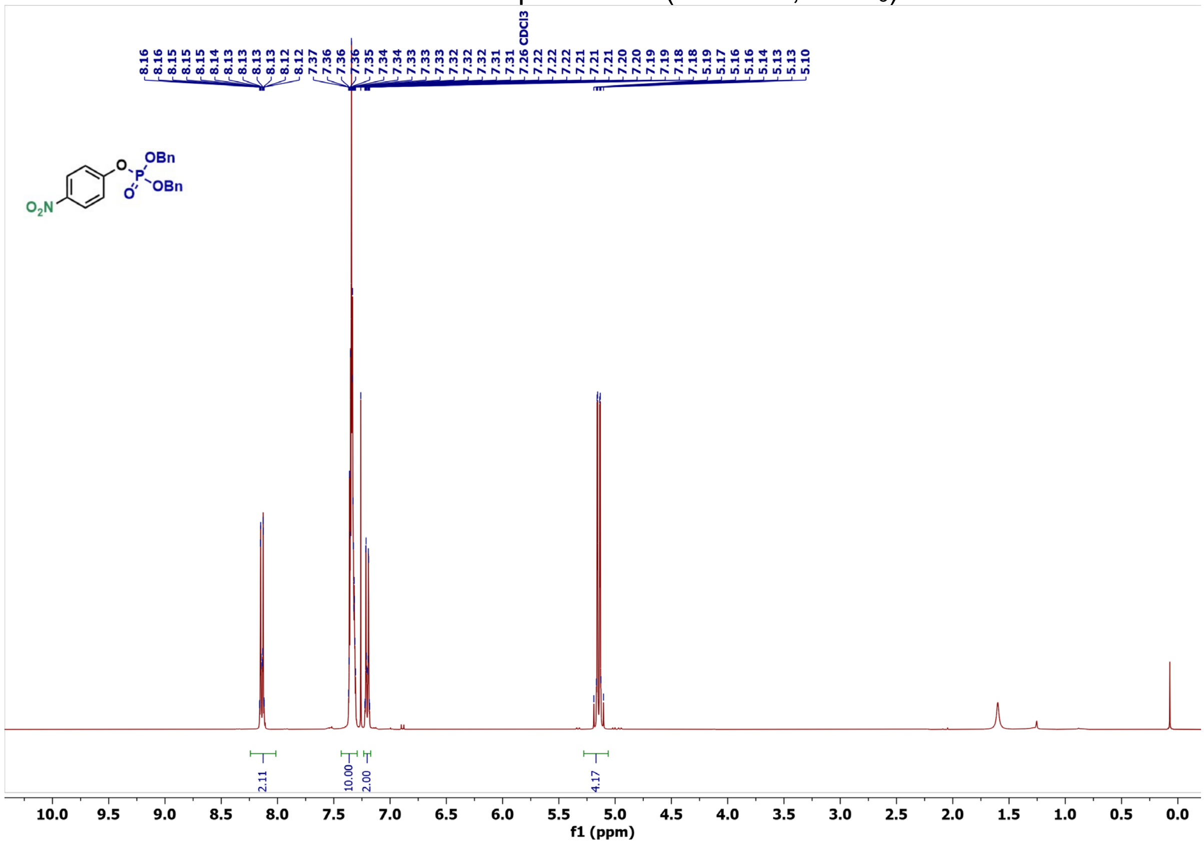

^{13}C NMR of Compound **10a** (125 MHz, $CDCl_3$)

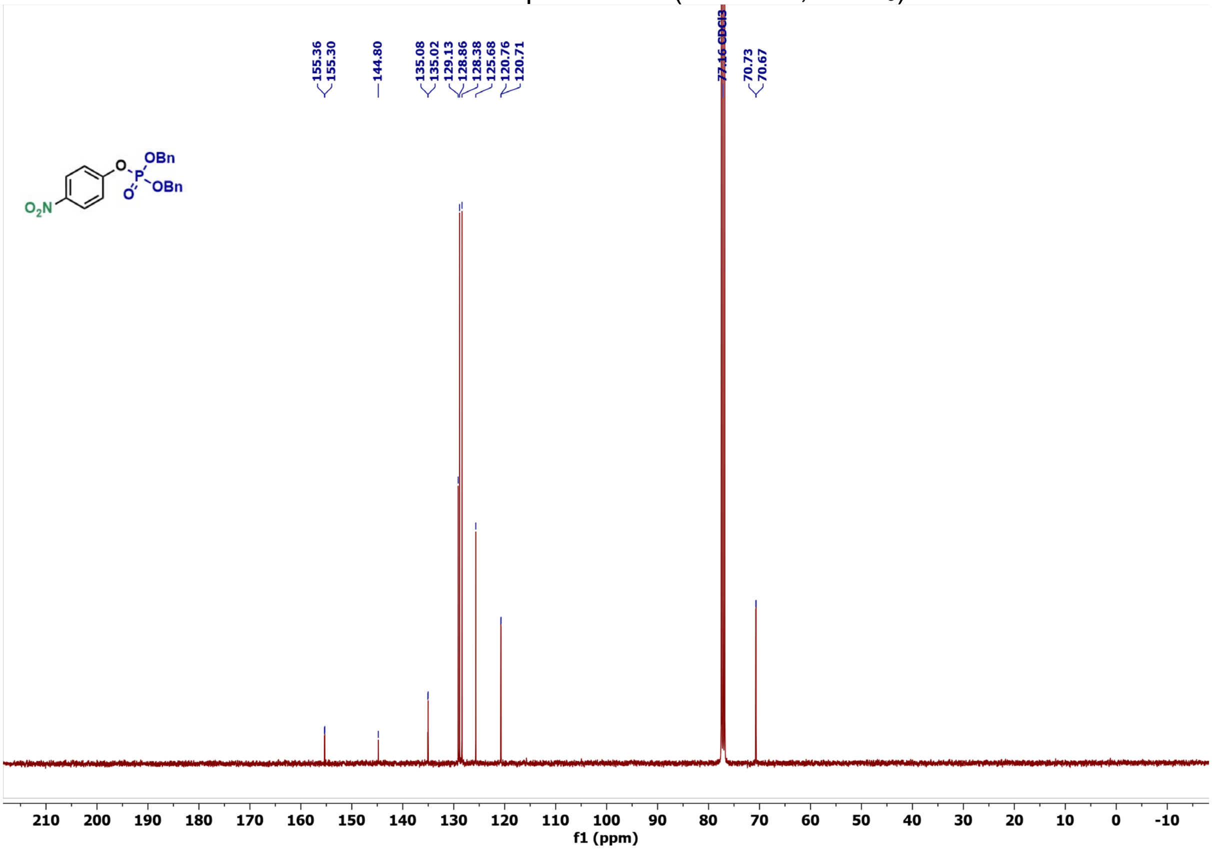

^{31}P NMR of Compound **10a** (162 MHz, $CDCl_3$)

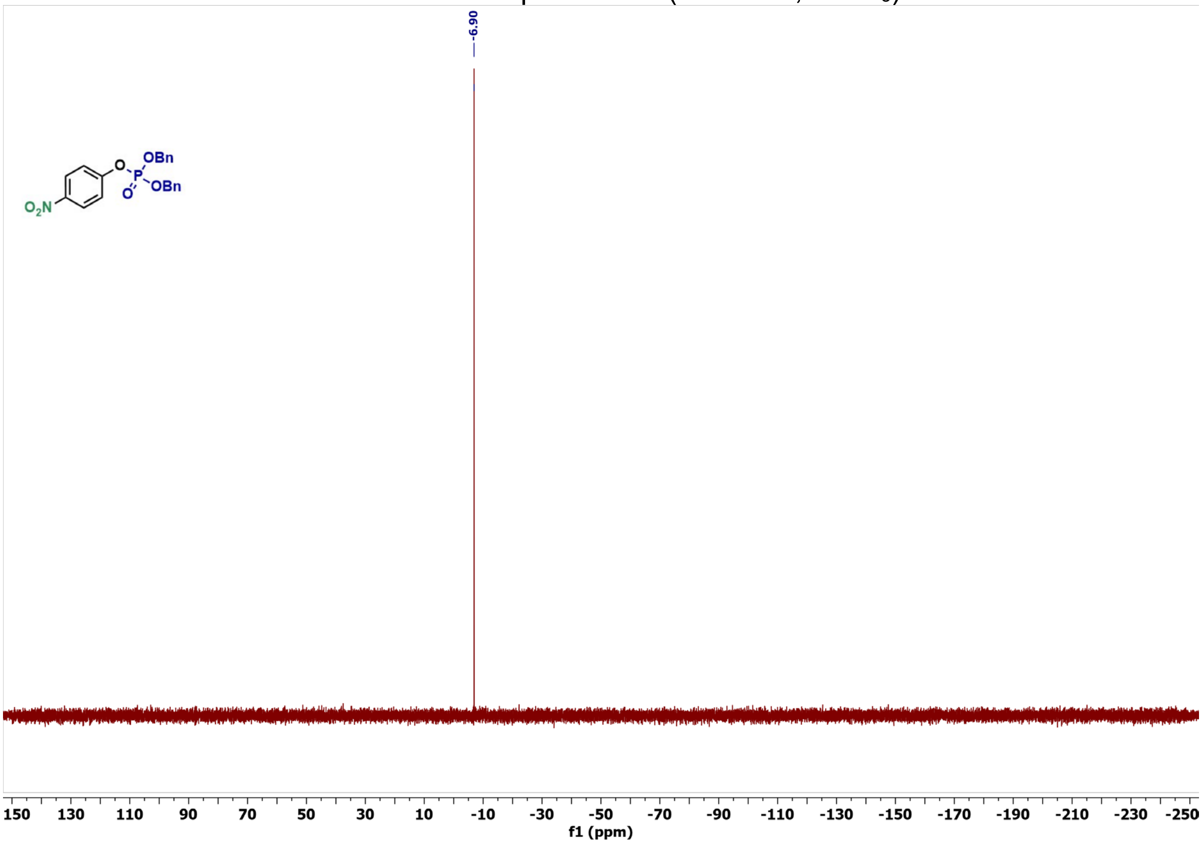

^{1}H NMR of Compound **10b** (400 MHz, DMSO-d_6)

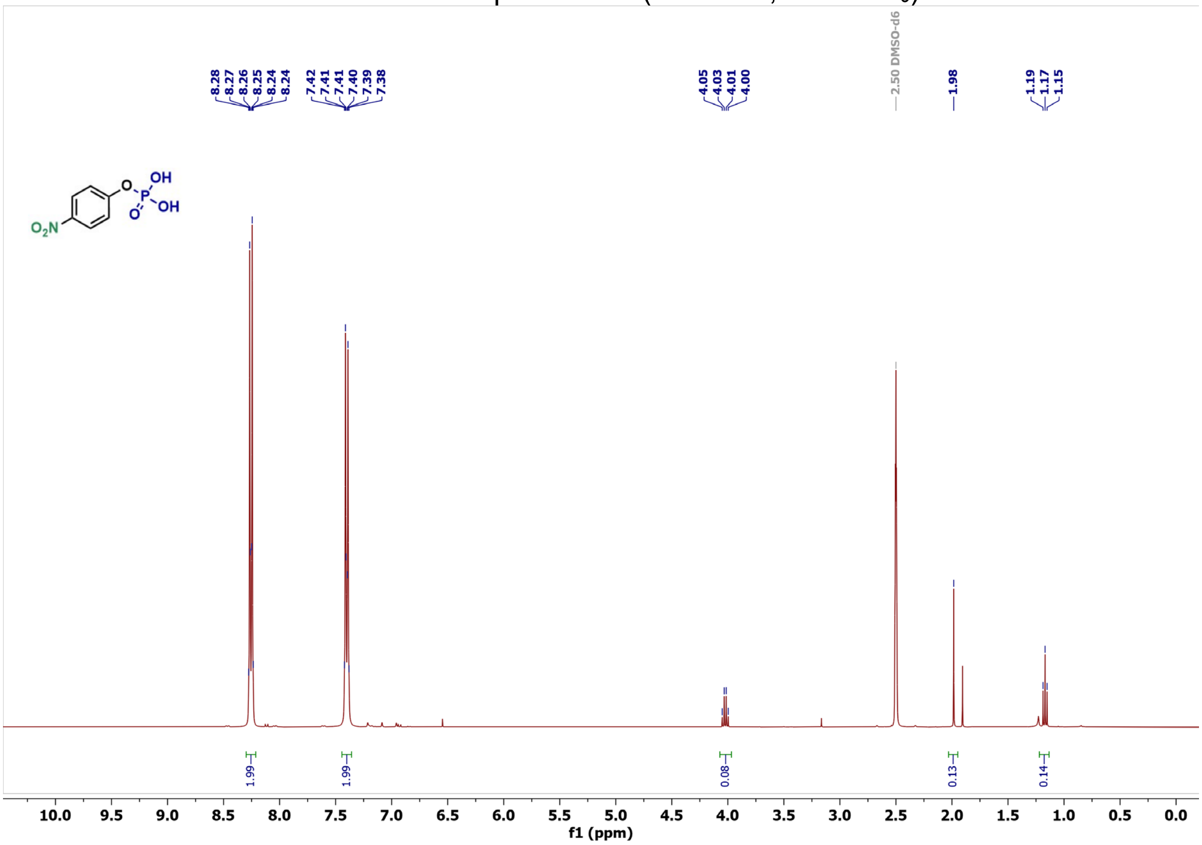

^{13}C NMR of Compound **10b** (101 MHz, DMSO-d_6)

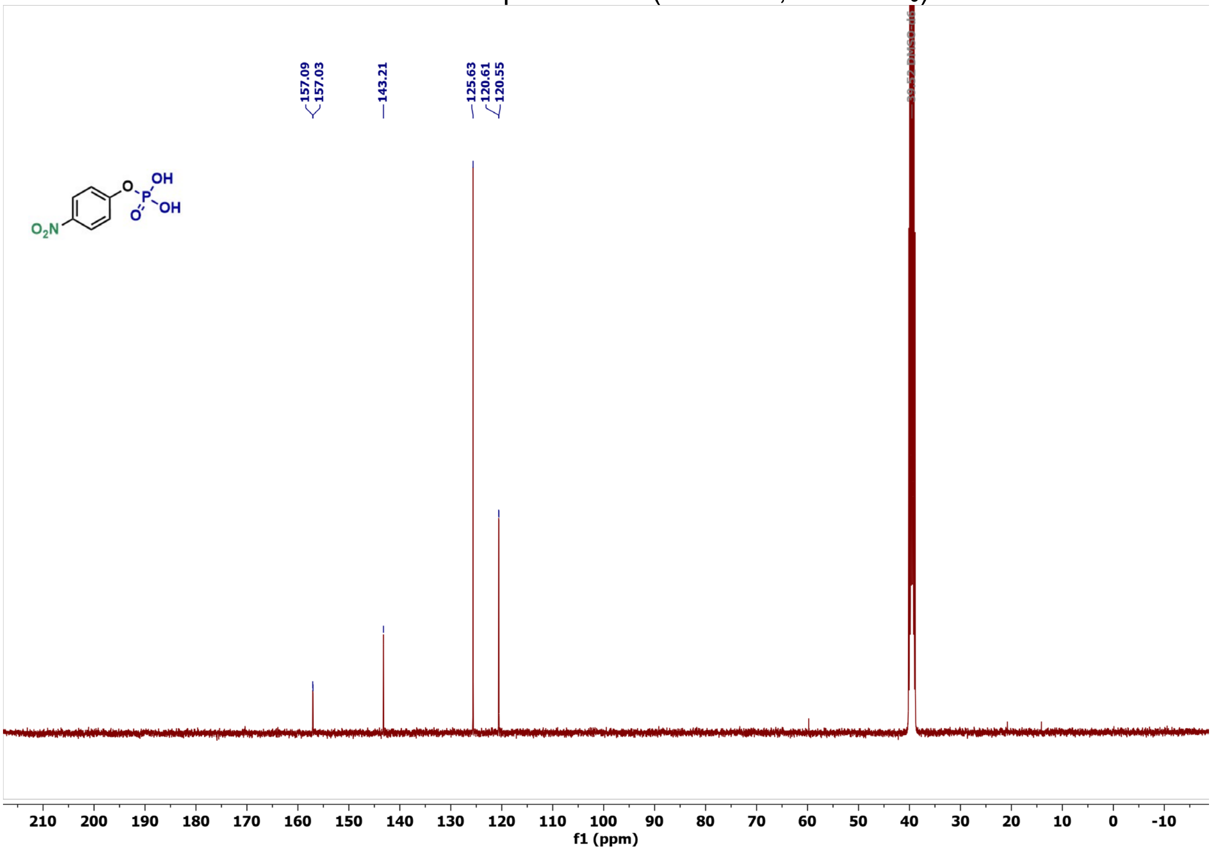

^{31}P NMR of Compound **10b** (162 MHz, DMSO-d_6)

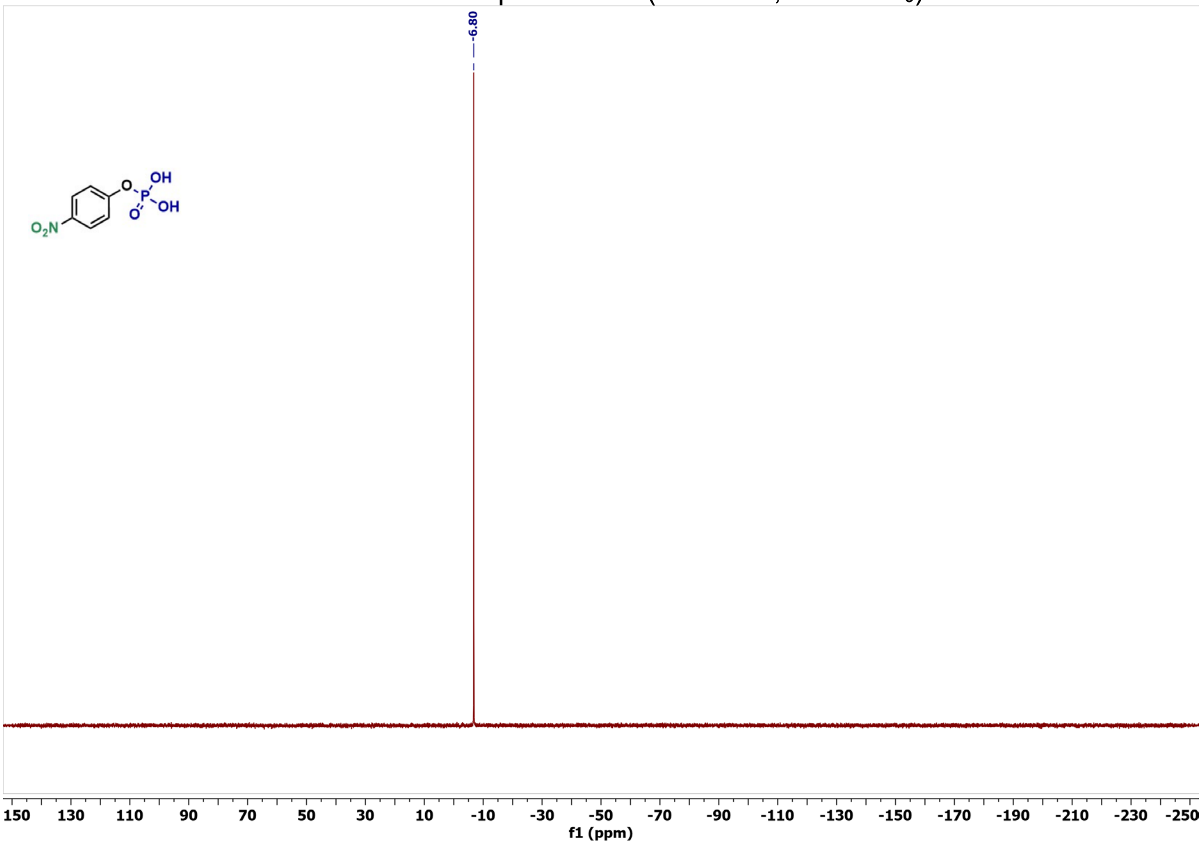

^{1}H NMR of Compound **11a** (600 MHz, MeOD)

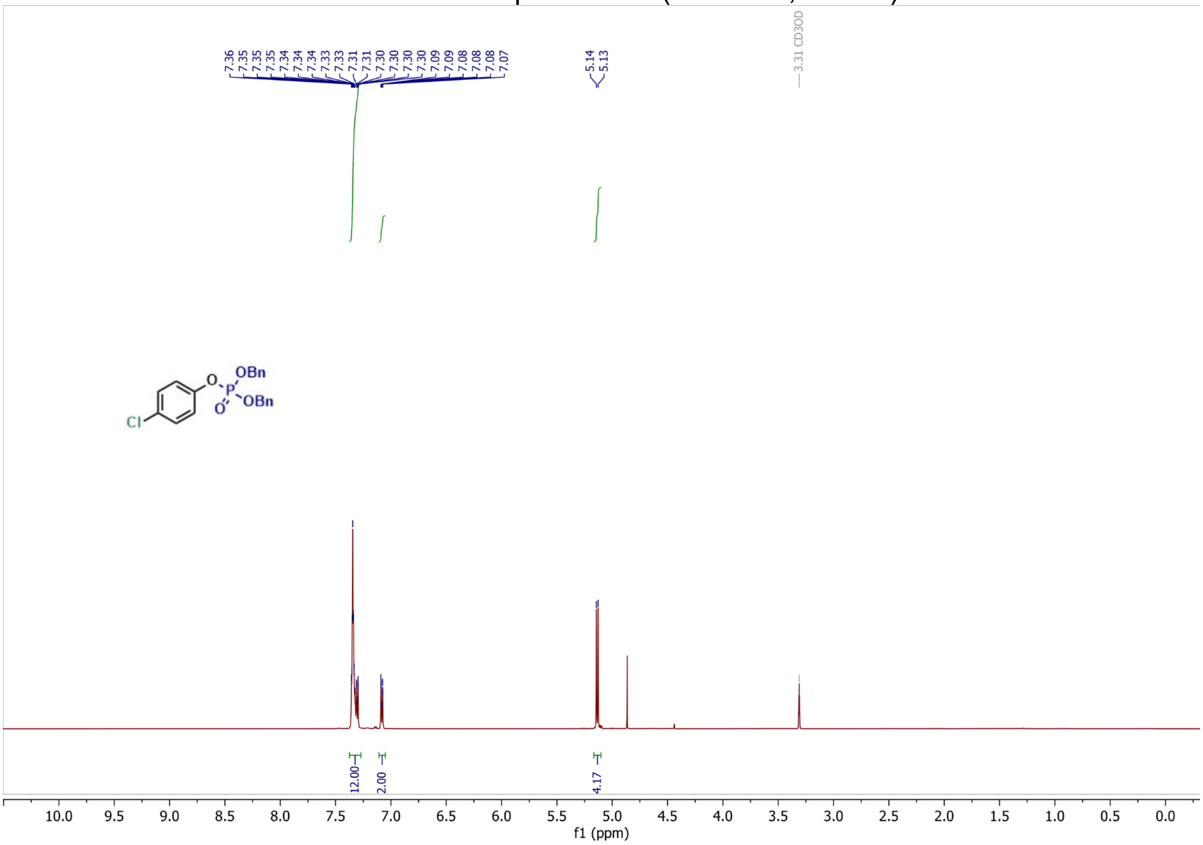

^{13}C NMR of Compound **11a** (151 MHz, MeOD)

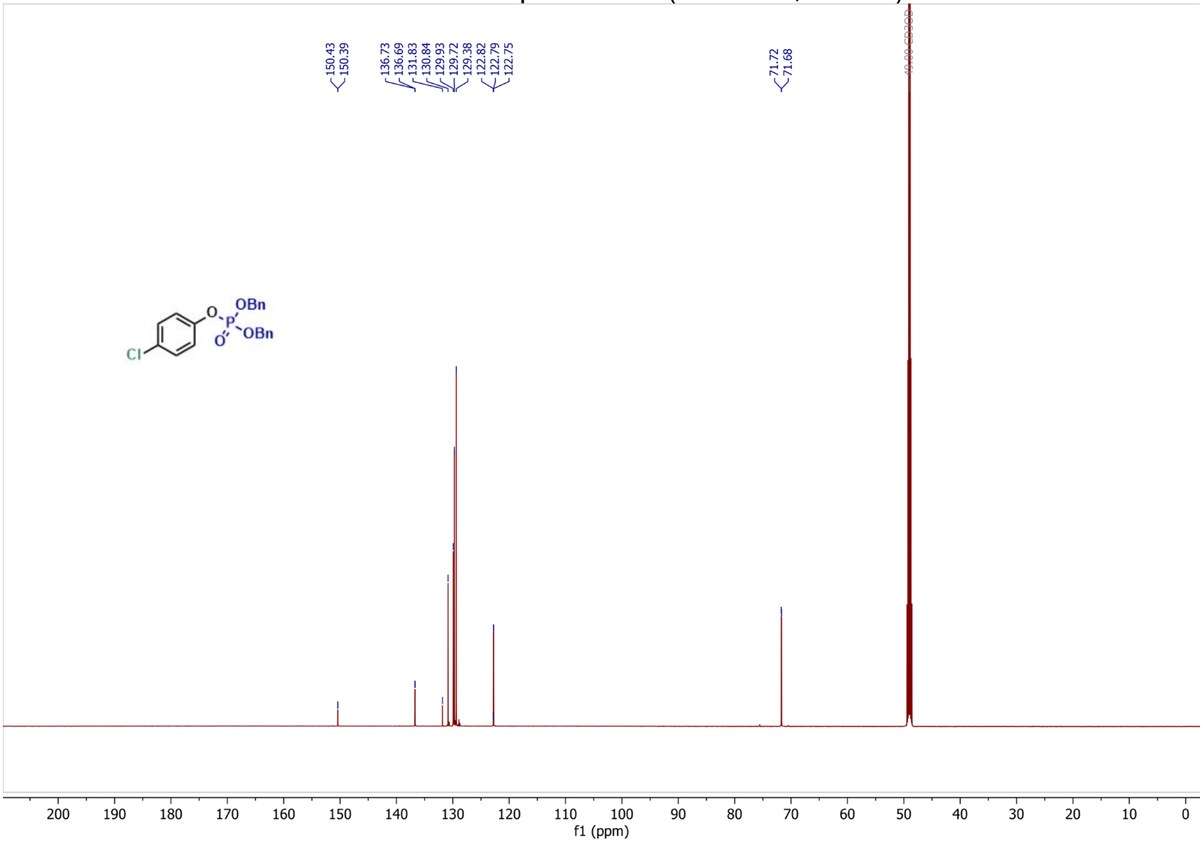

^{31}P NMR of Compound **11a** (243 MHz, $CDCl_3$)

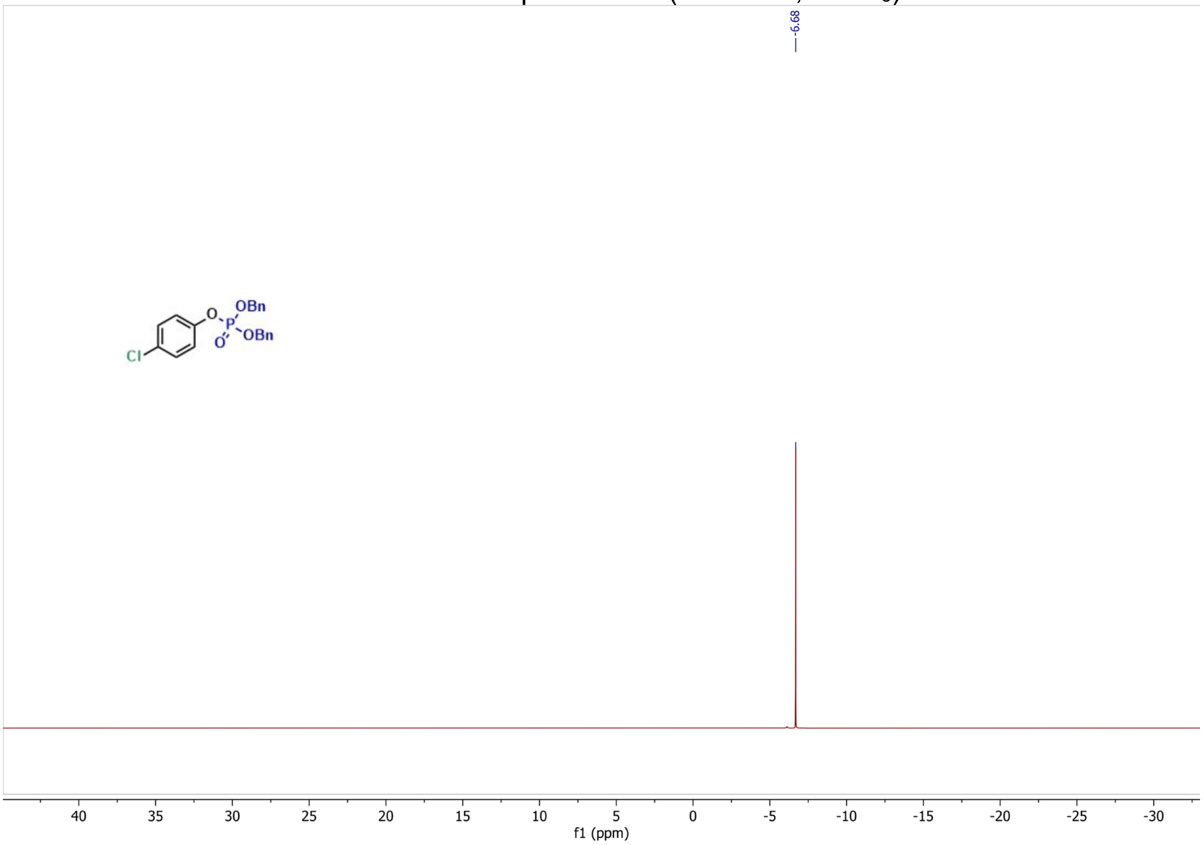

^{1}H NMR of Compound **12a** (600 MHz, $CDCl_3$)

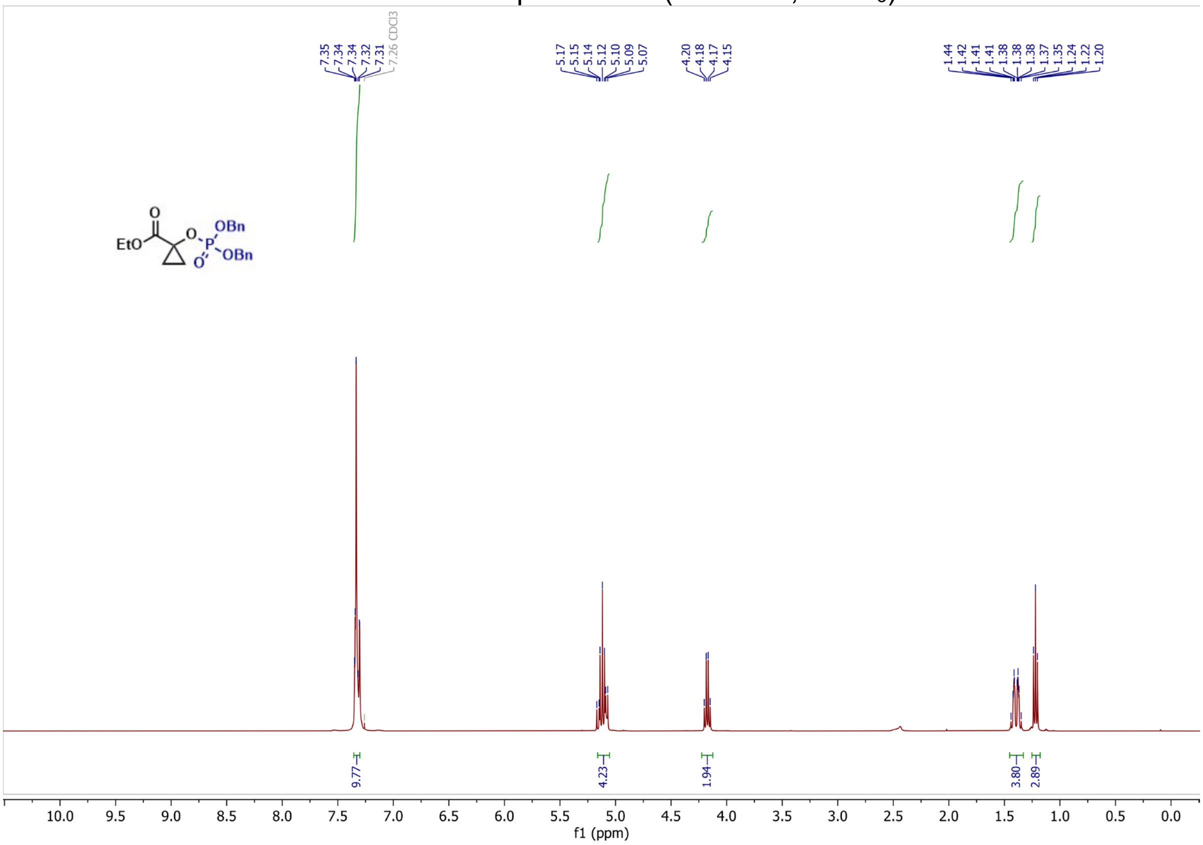

^{13}C NMR of Compound **12a** (151 MHz, $CDCl_3$)

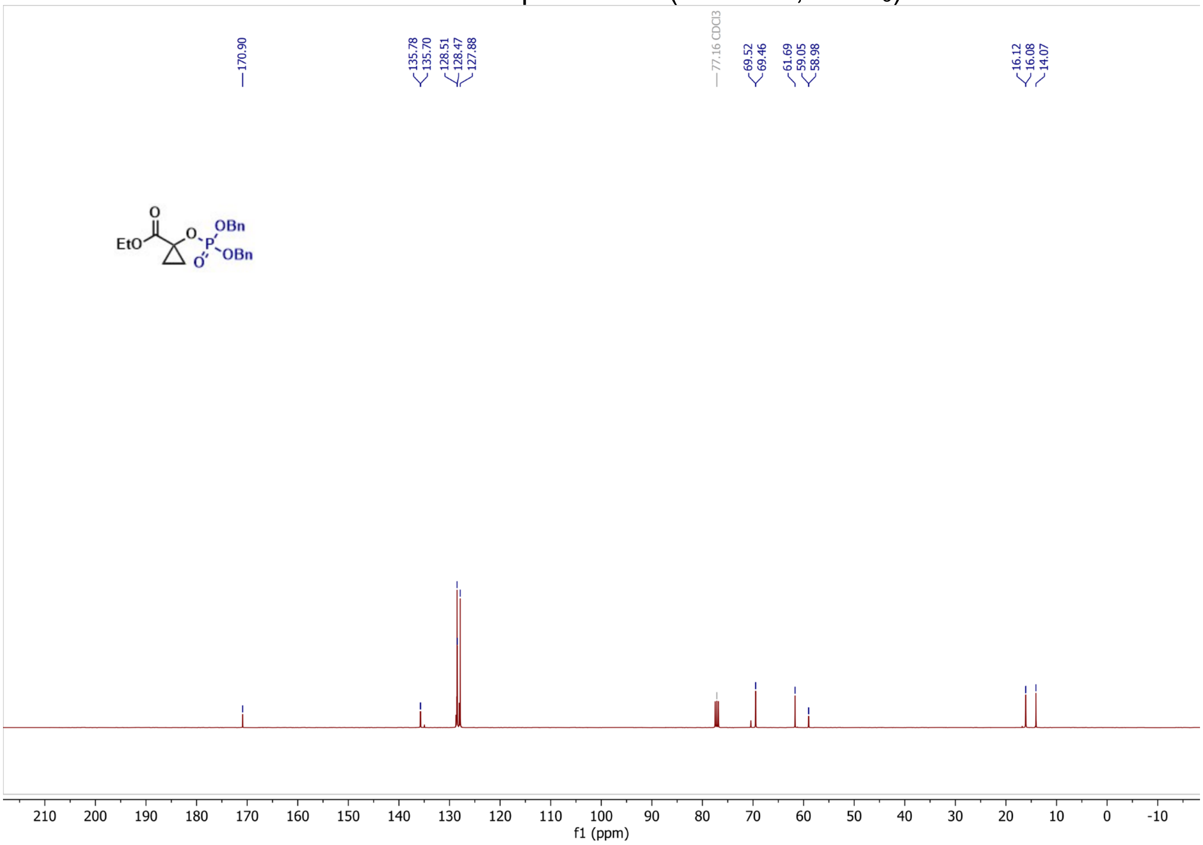

^{31}P NMR of Compound **12a** (243 MHz, $CDCl_3$)

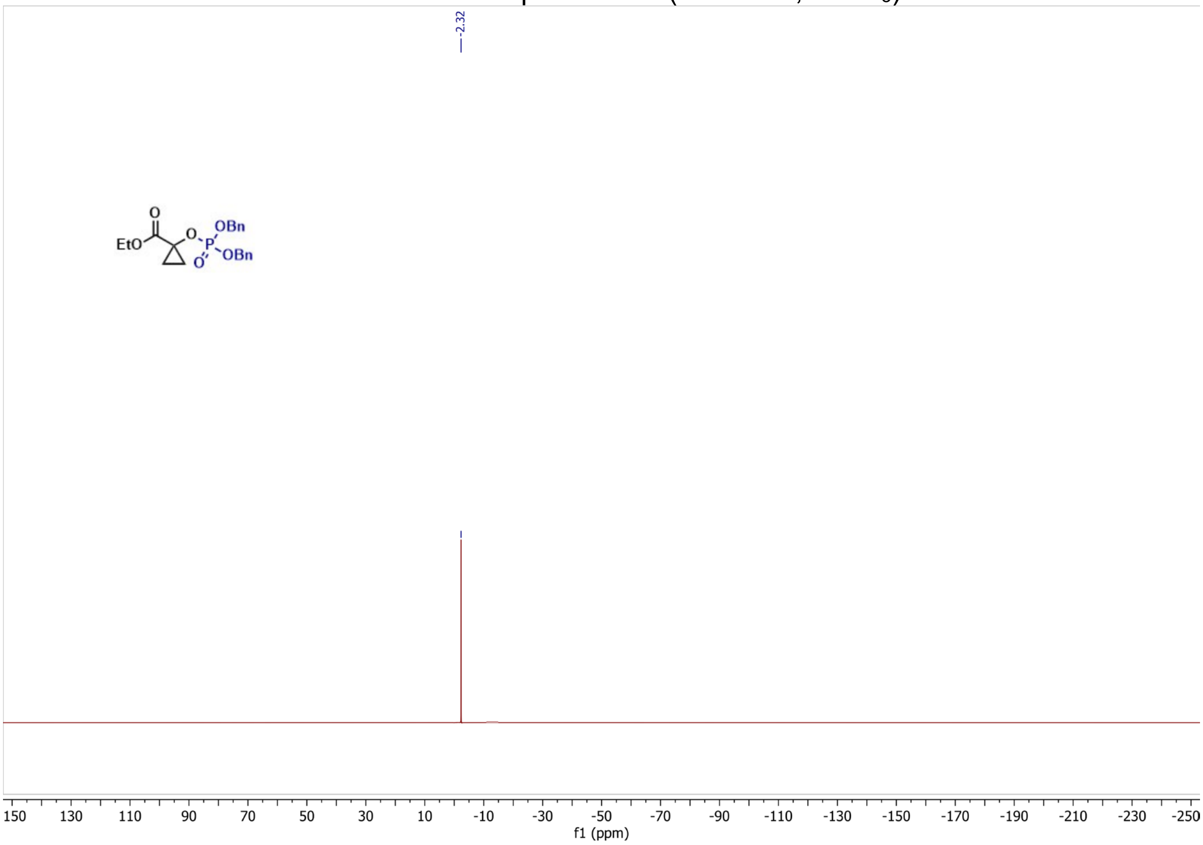

^{1}H NMR of Compound **12b** (600 MHz, $CDCl_3$)

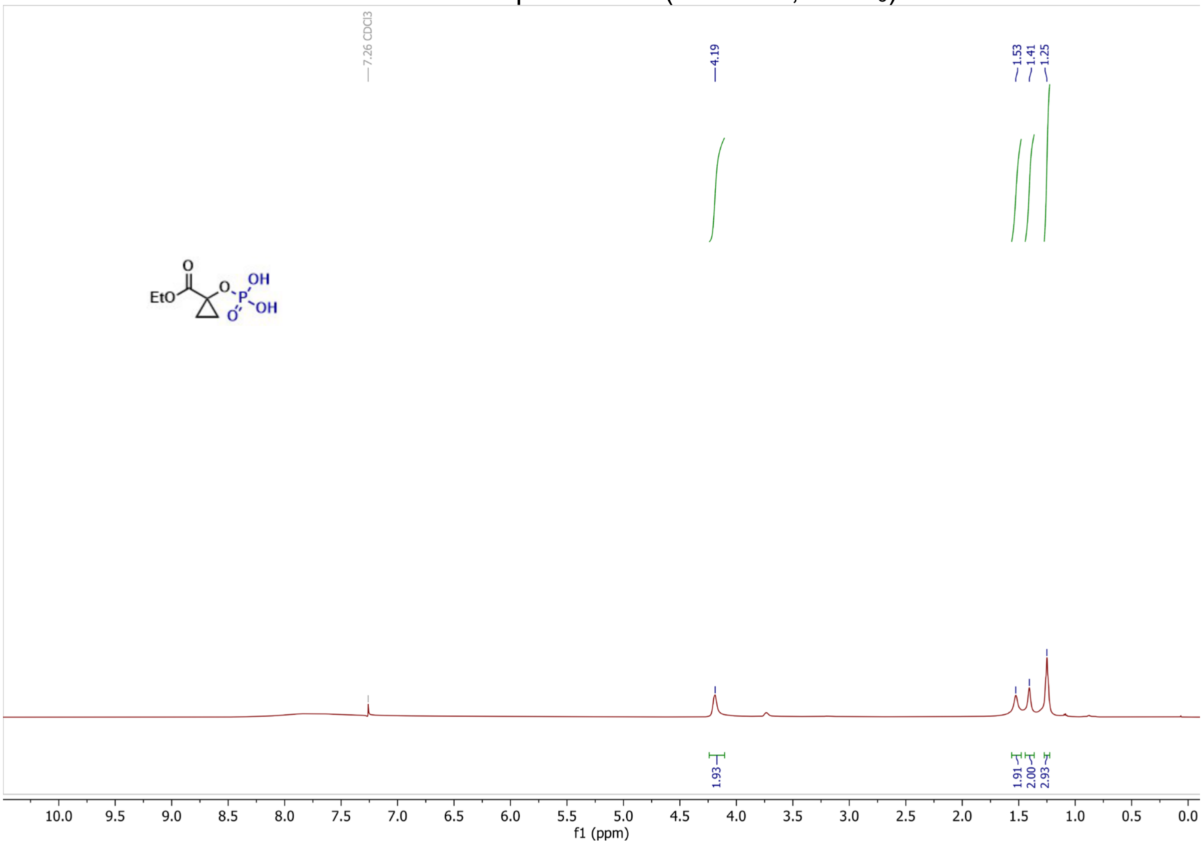

^{13}C NMR of Compound **12b** (151 MHz, $CDCl_3$)

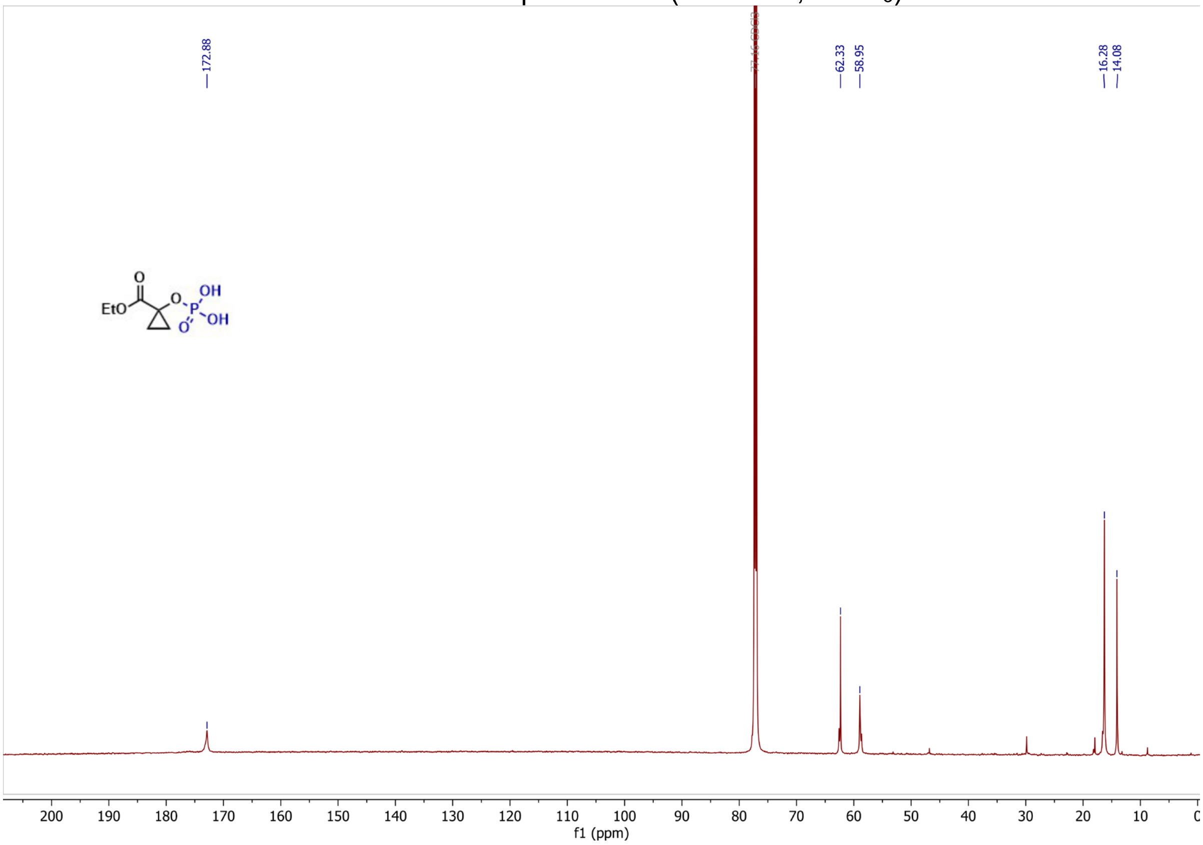

^{31}P NMR of Compound **12b** (243 MHz, $CDCl_3$)

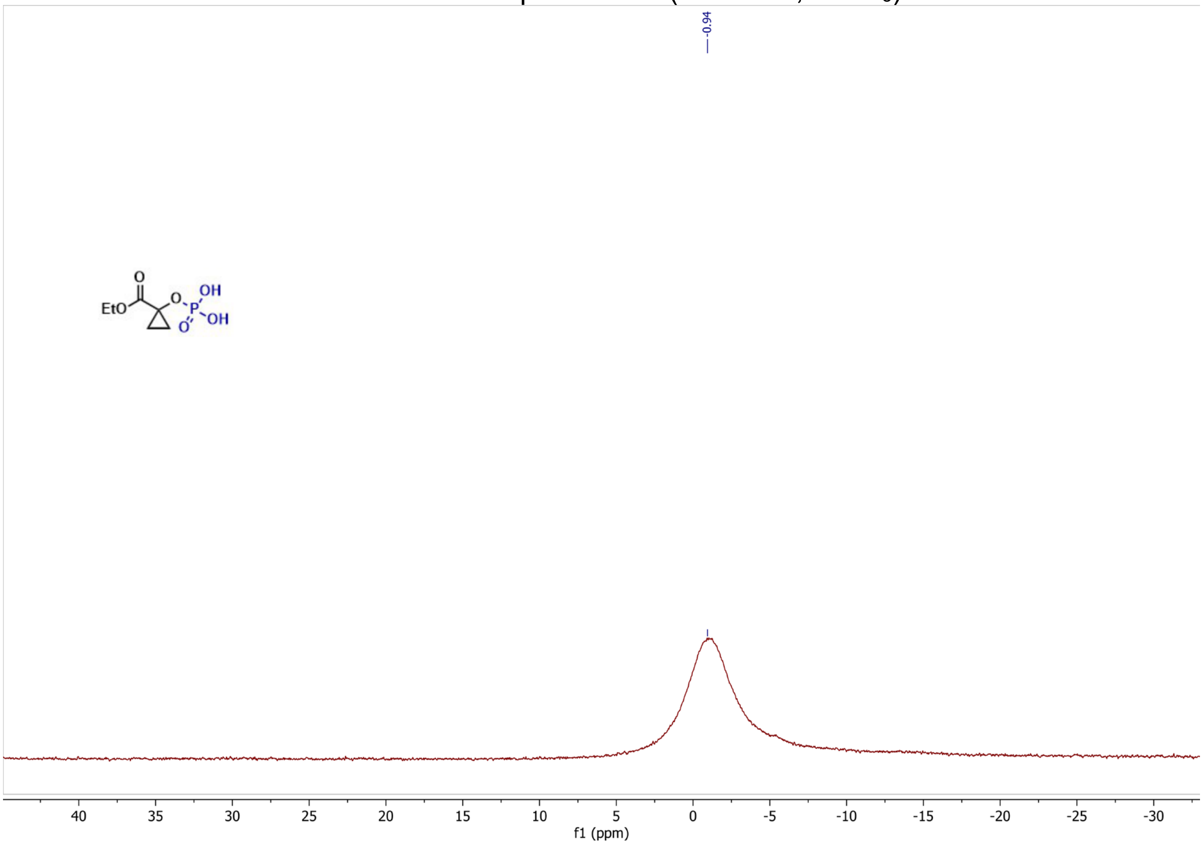

^{1}H NMR of Compound **13a** (800 MHz, $CDCl_3$)

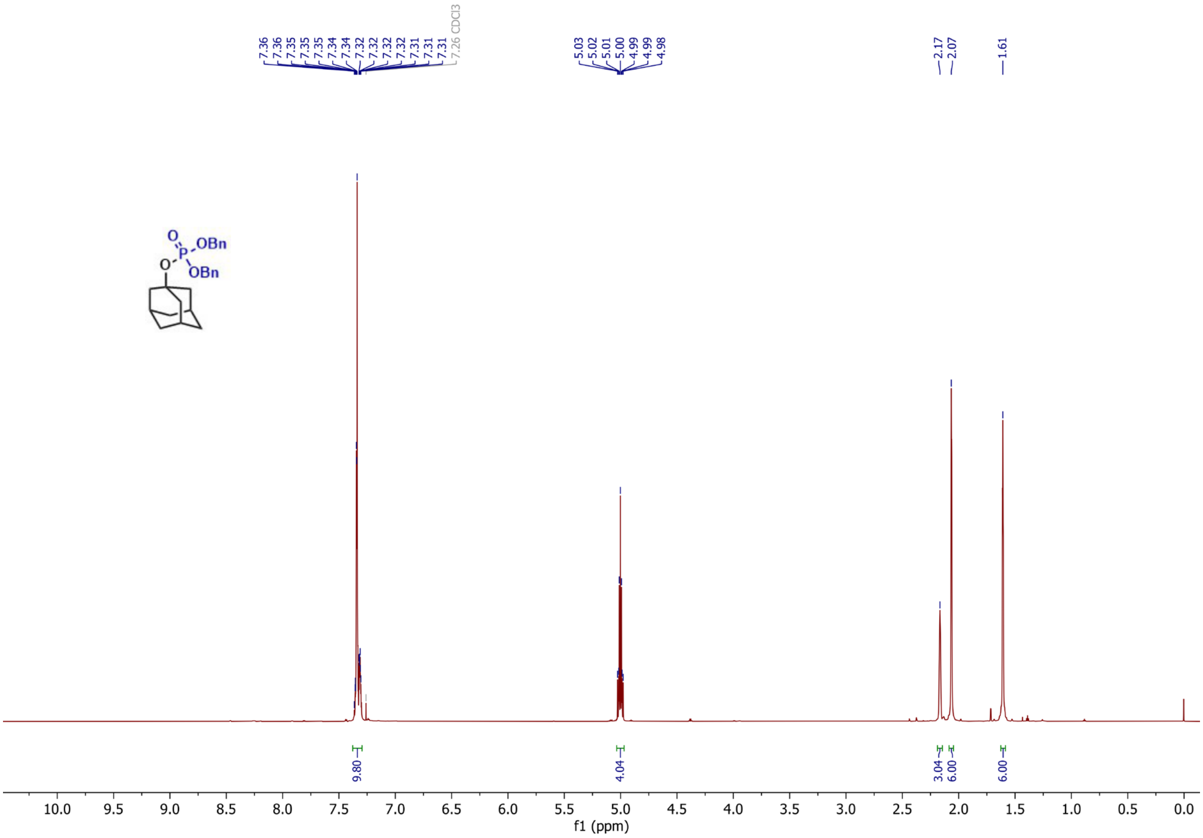

^{13}C NMR of Compound **13a** (125 MHz, $CDCl_3$)

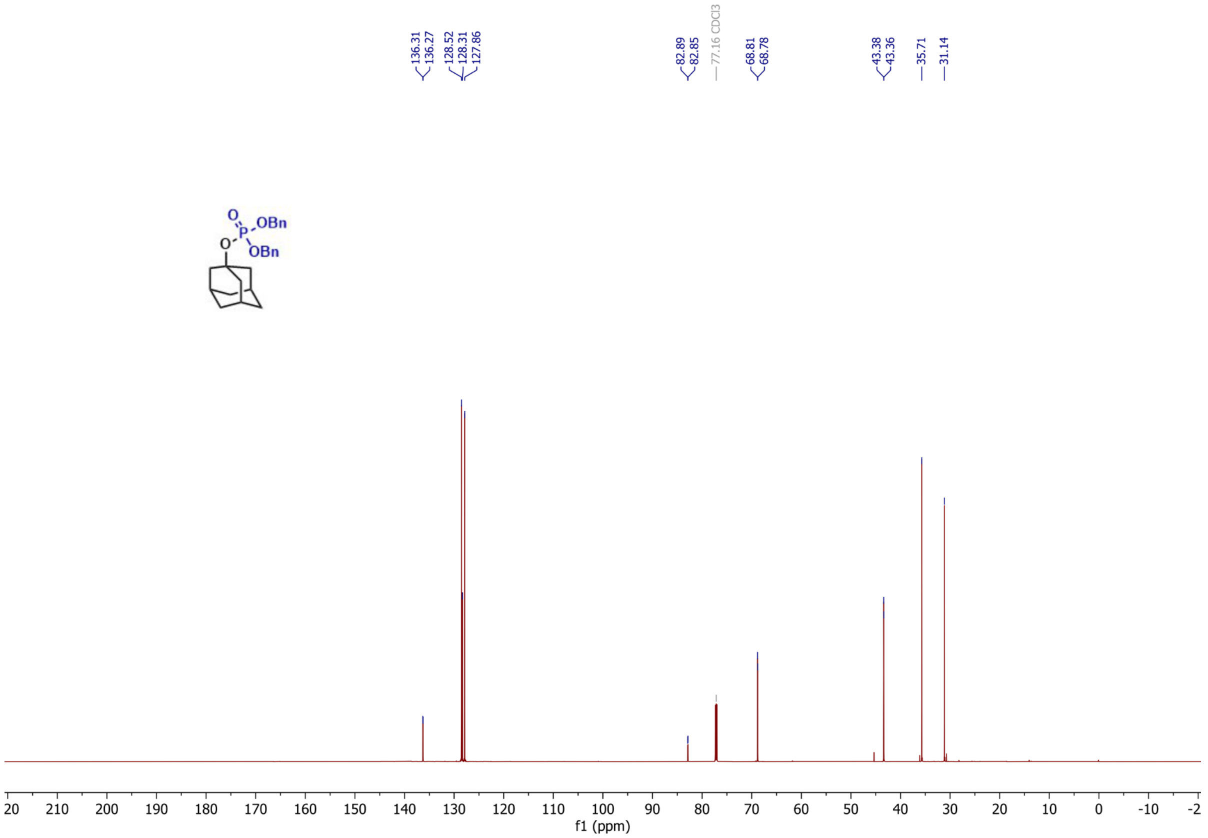

^{31}P NMR of Compound **13a** (162 MHz, $CDCl_3$)

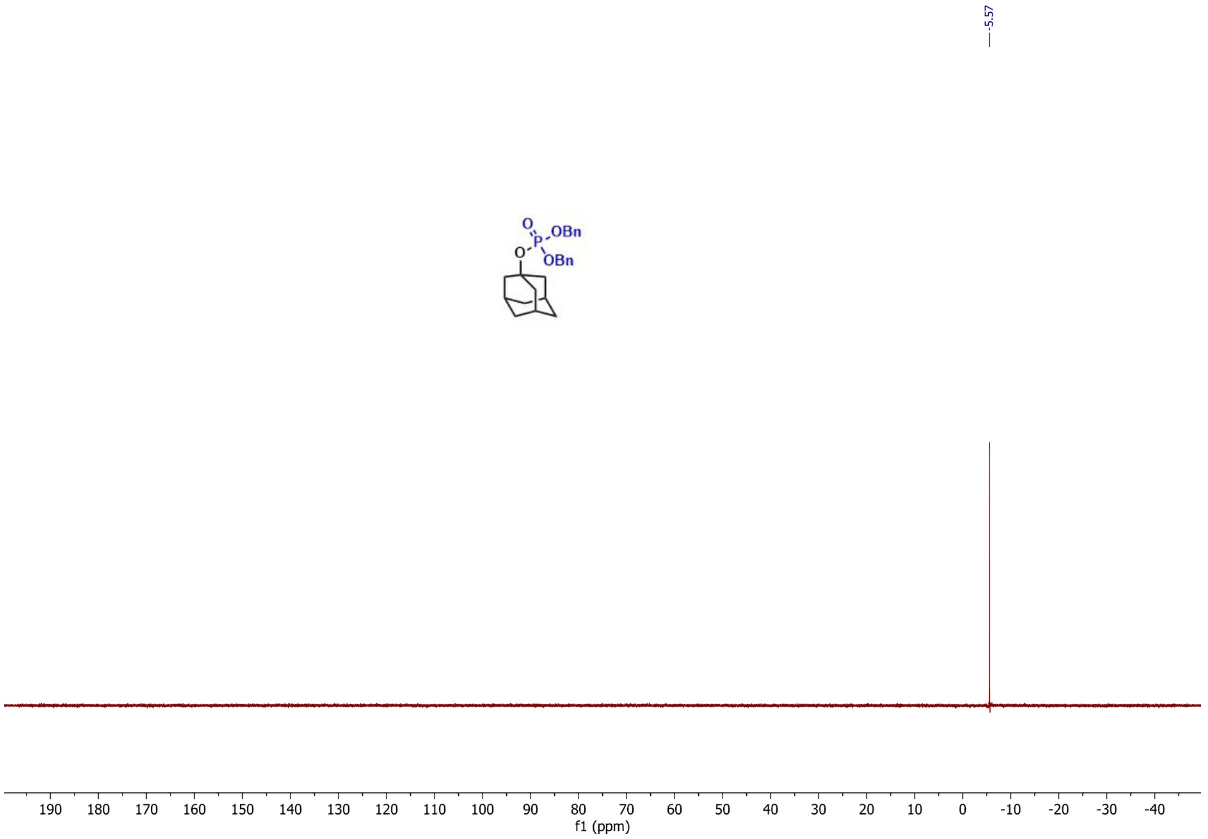

^{1}H NMR of Compound **13b** (400 MHz, DMSO-d_6)

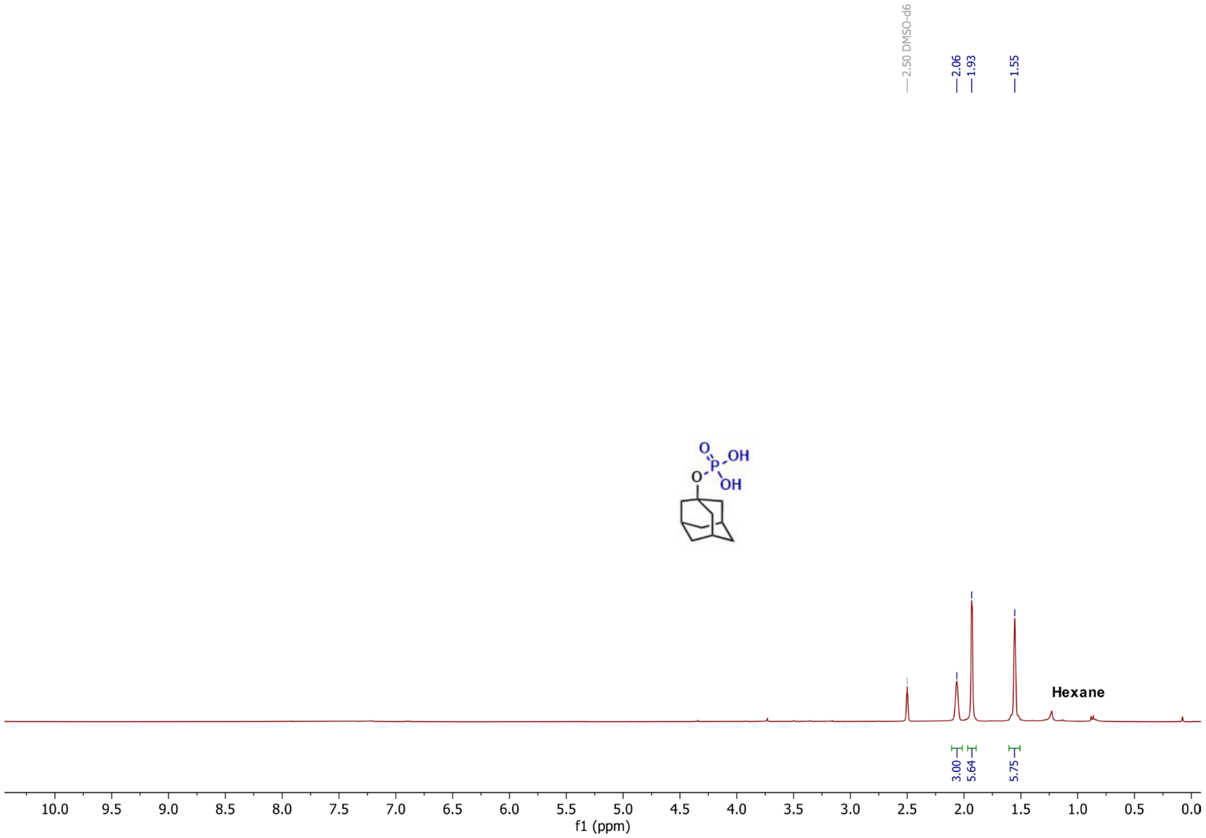

^{13}C NMR of Compound **13b** (101 MHz, DMSO-d_6)

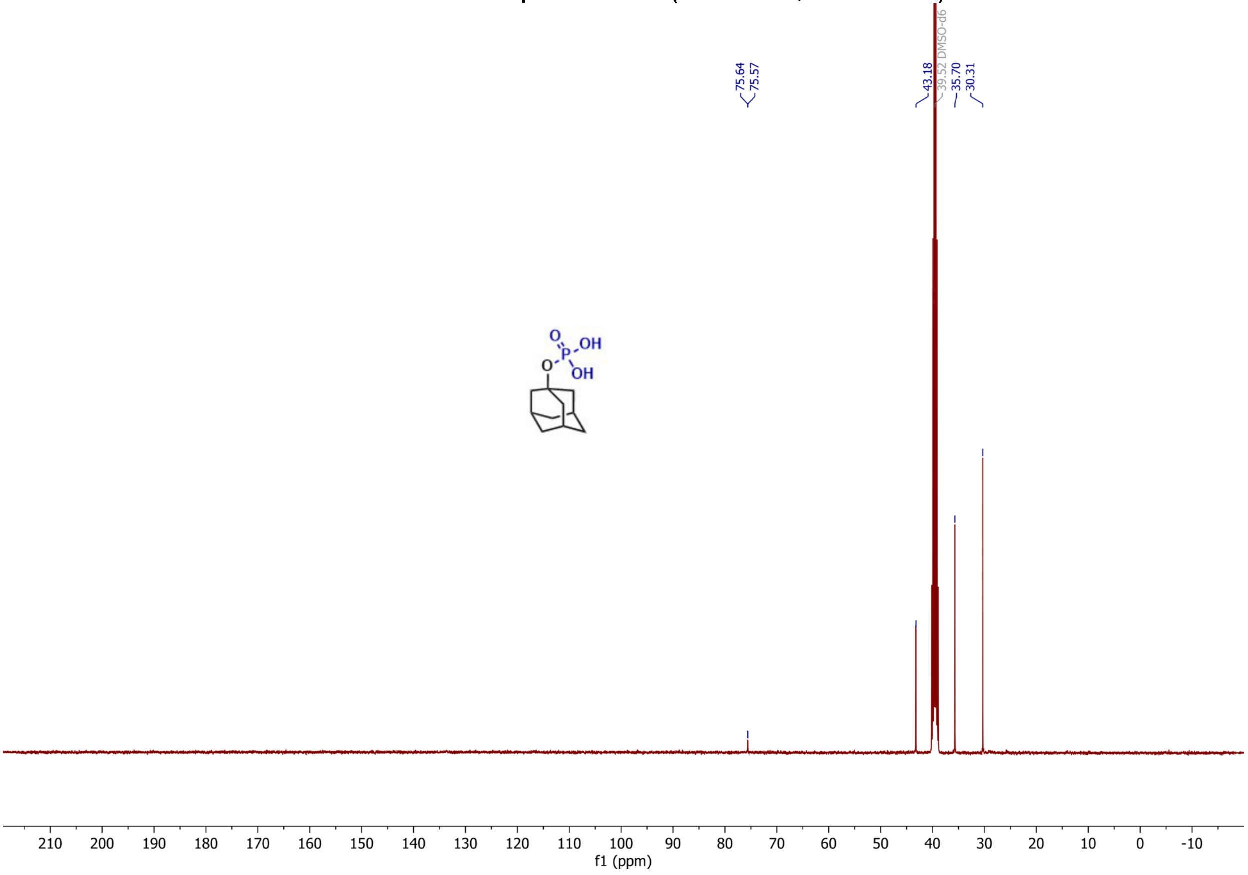

^{31}P NMR of Compound **13b** (162 MHz, DMSO-d_6)

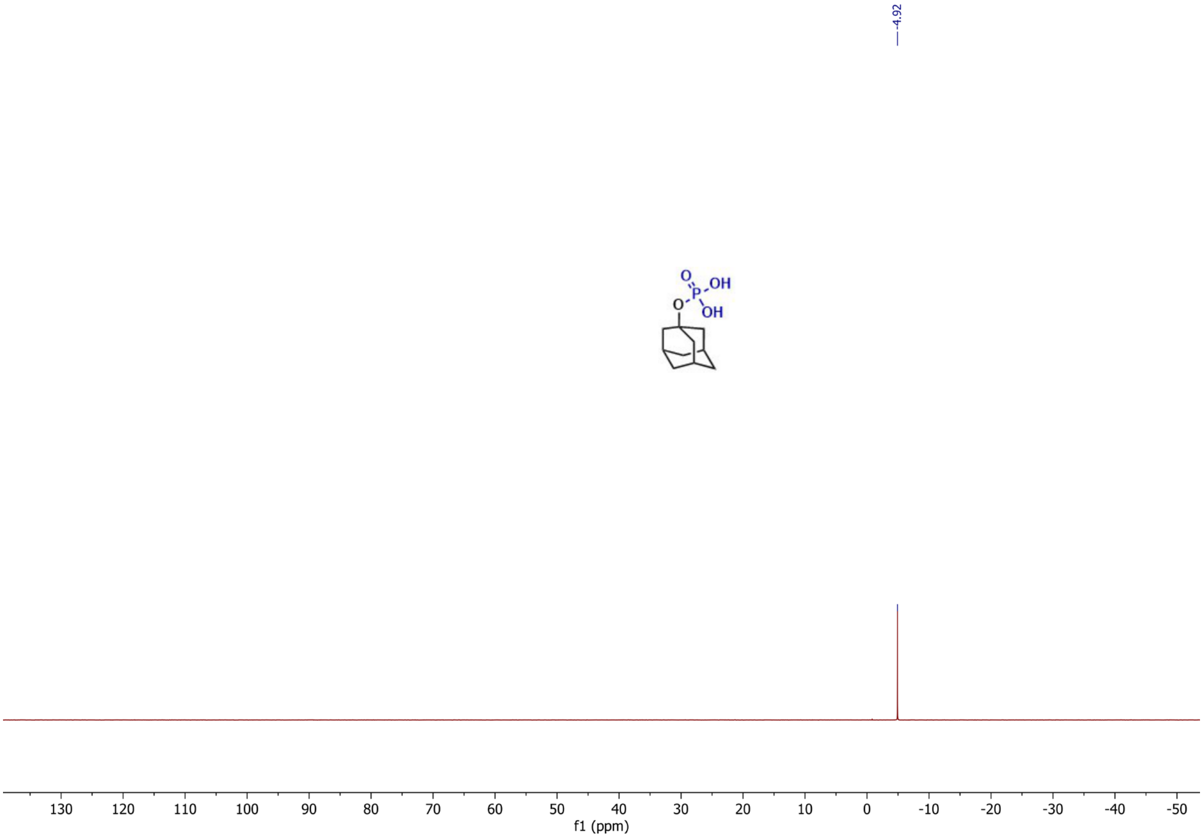

^{1}H NMR of Compound **14a** (800 MHz, $CDCl_3$)

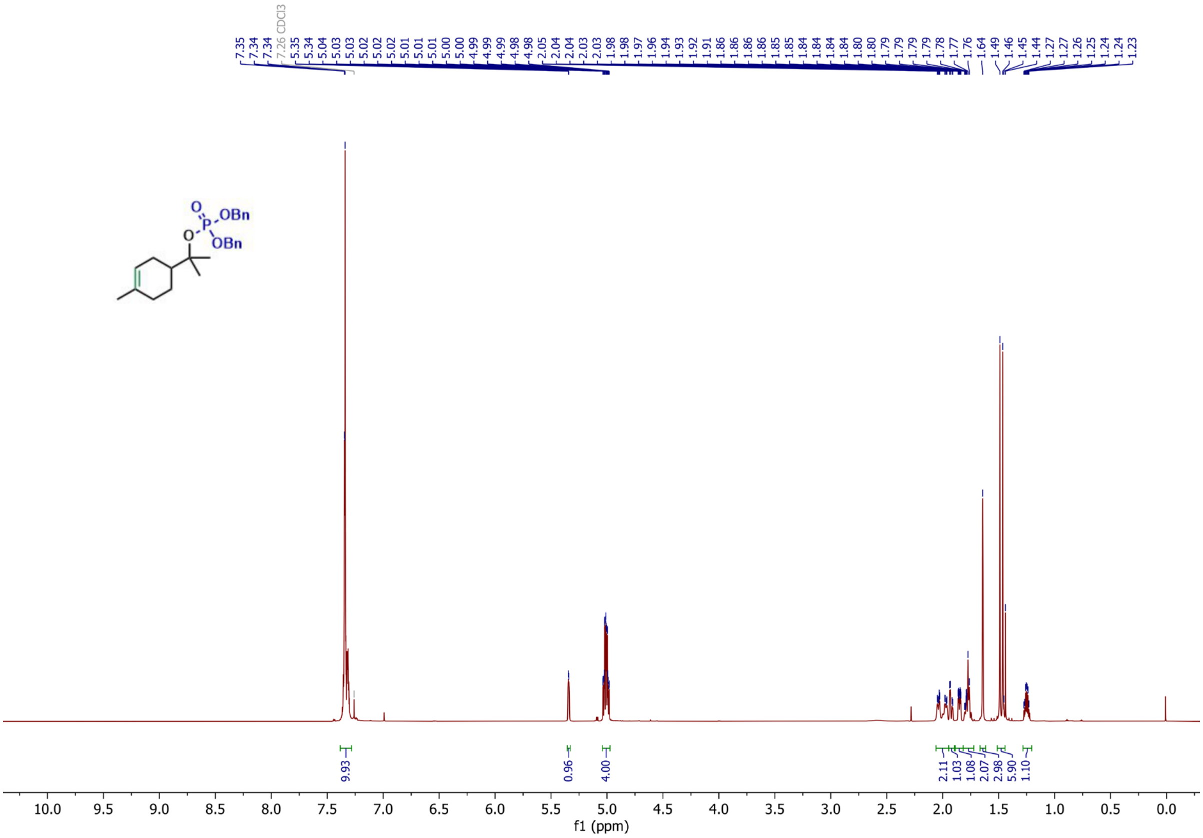

^{13}C NMR of Compound **14a** (125 MHz, $CDCl_3$)

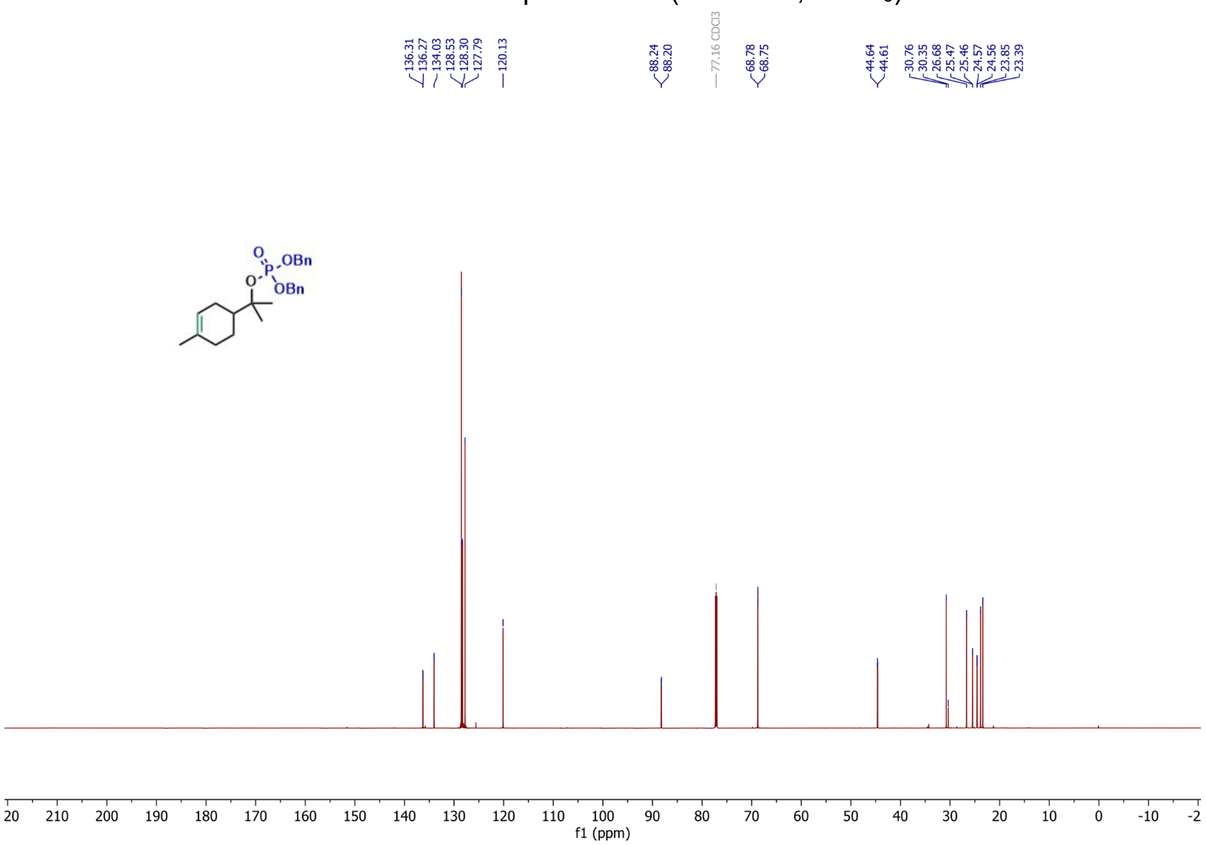

^{31}P NMR of Compound **14a** (162 MHz, $CDCl_3$)

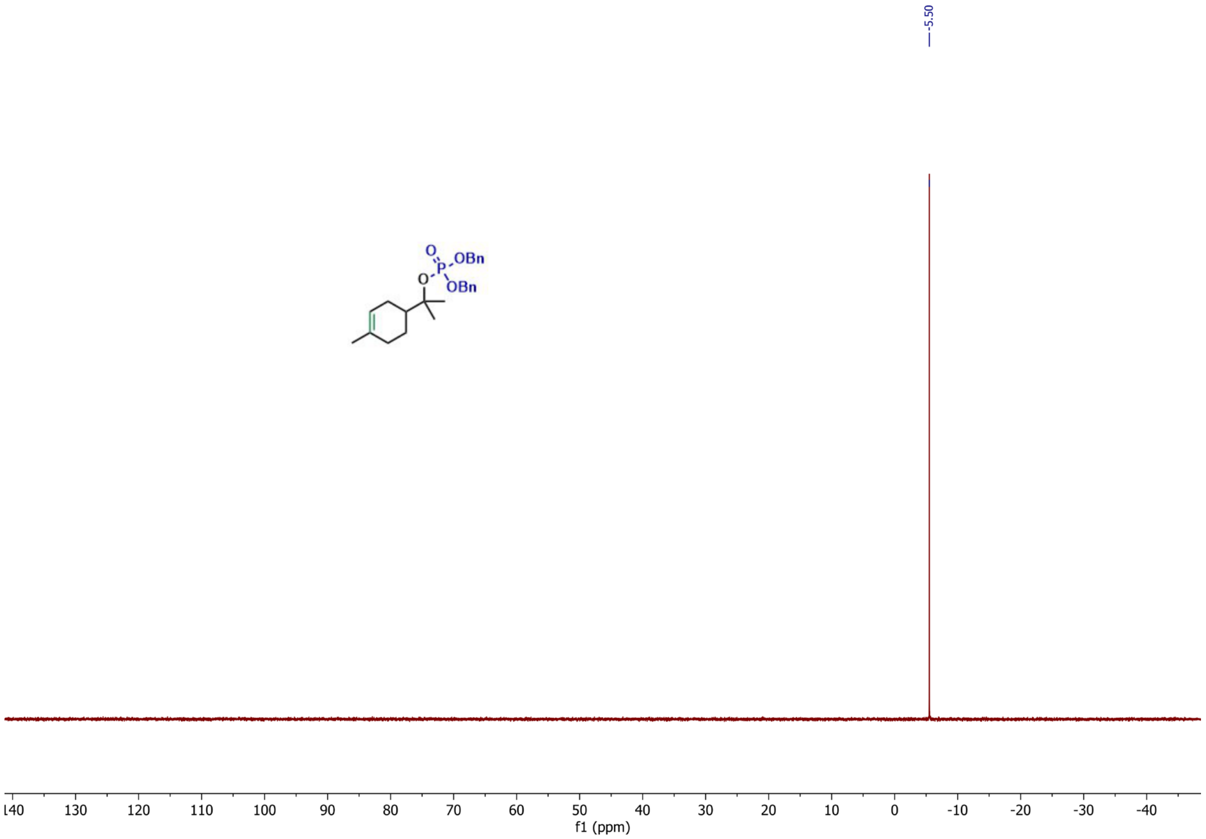

^{1}H NMR of Compound **14b** (600 MHz, $CDCl_3$)

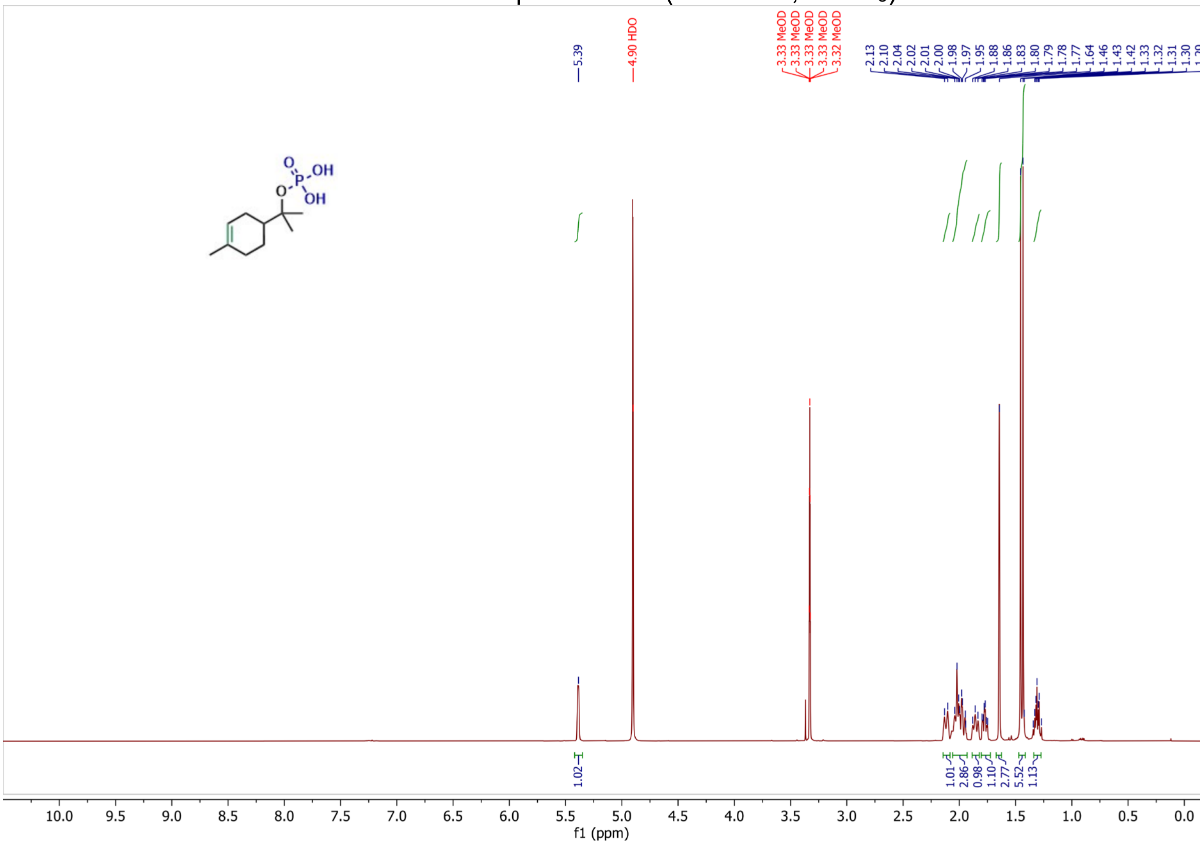

^{13}C NMR of Compound **14b** (151 MHz, $CDCl_3$)

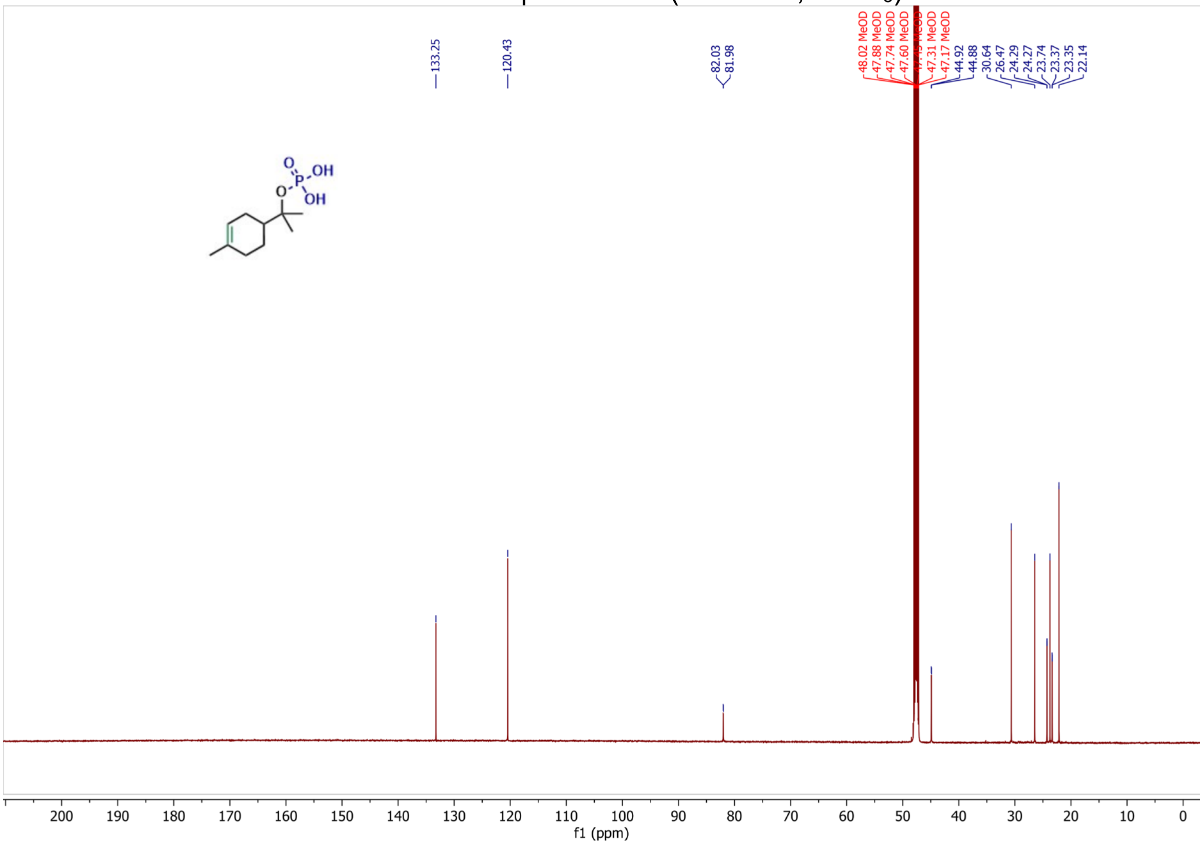

^{31}P NMR of Compound **14b** (243 MHz, $CDCl_3$)

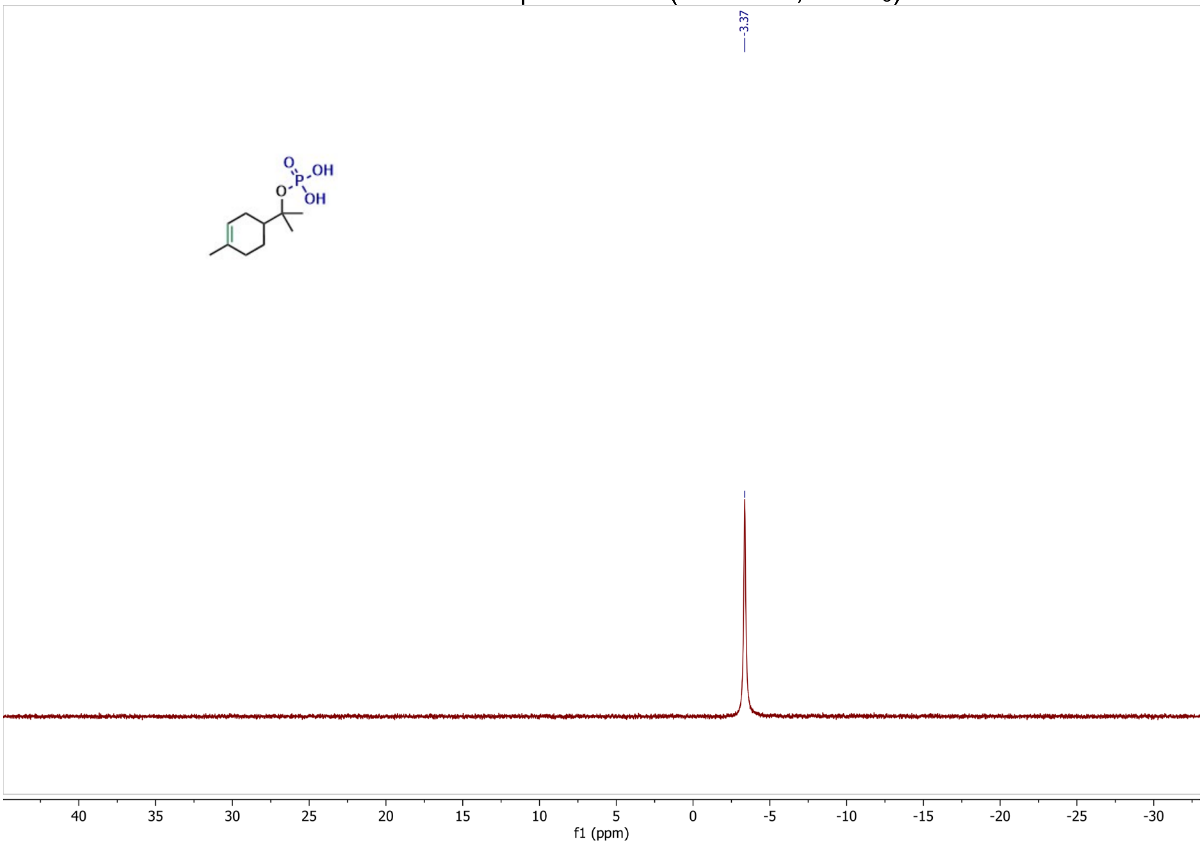

^{1}H NMR of Compound **15a** (600 MHz, DMSO-d_6)

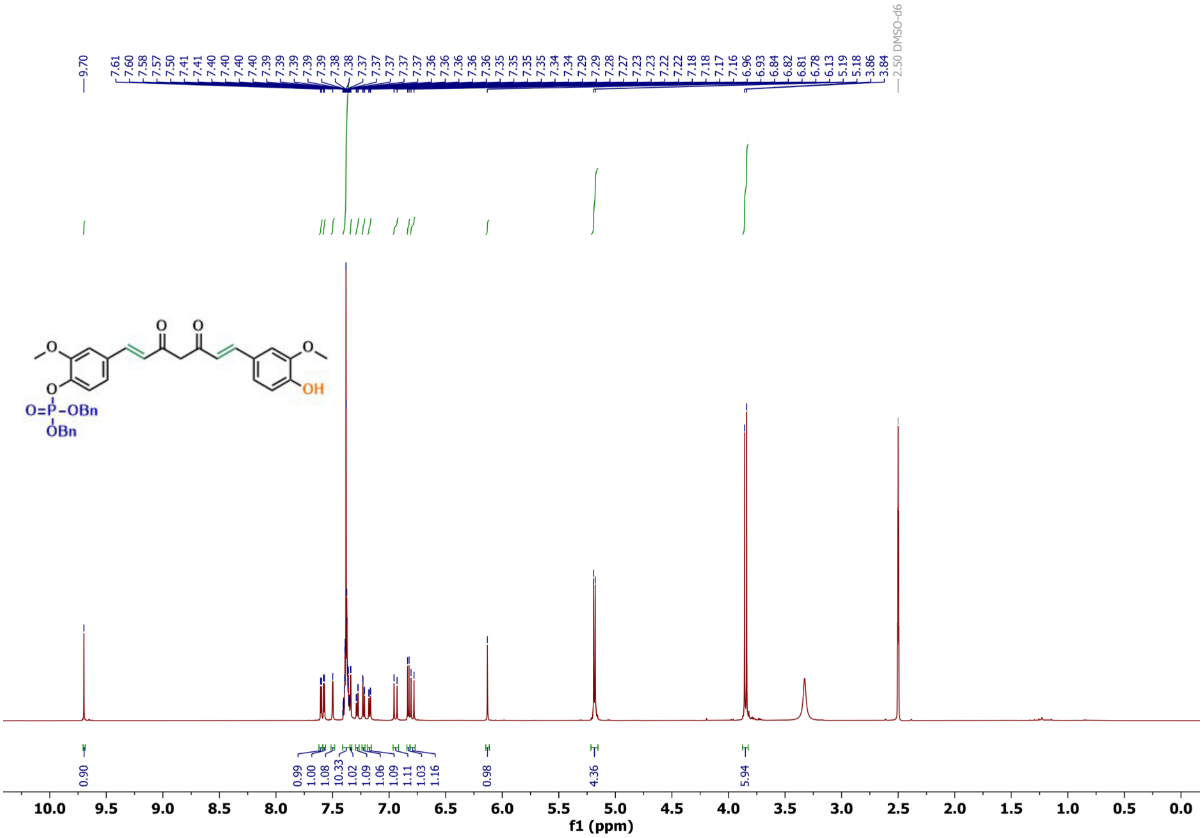

^{13}C NMR of Compound **15a** (151 MHz, DMSO-d_6)

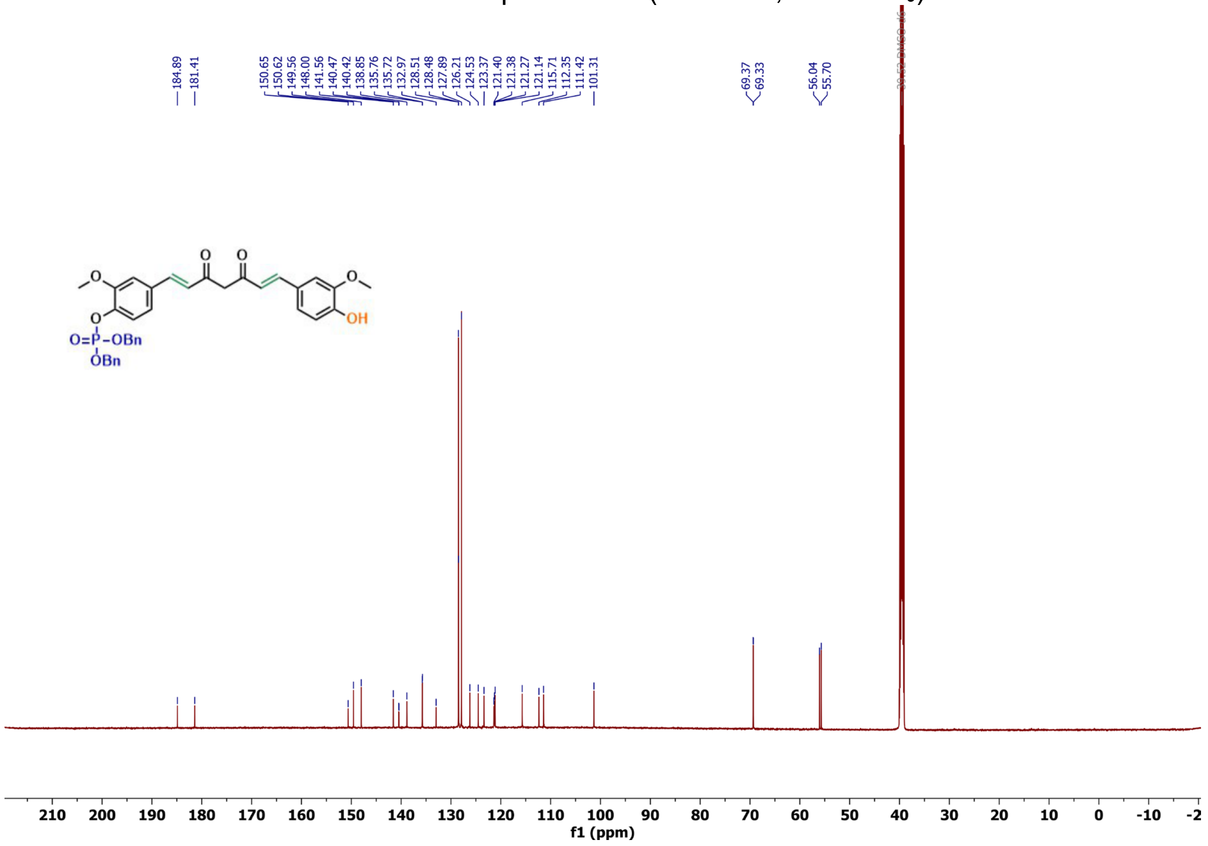

^{31}P NMR of Compound **15a** (243 MHz, DMSO-d_6)

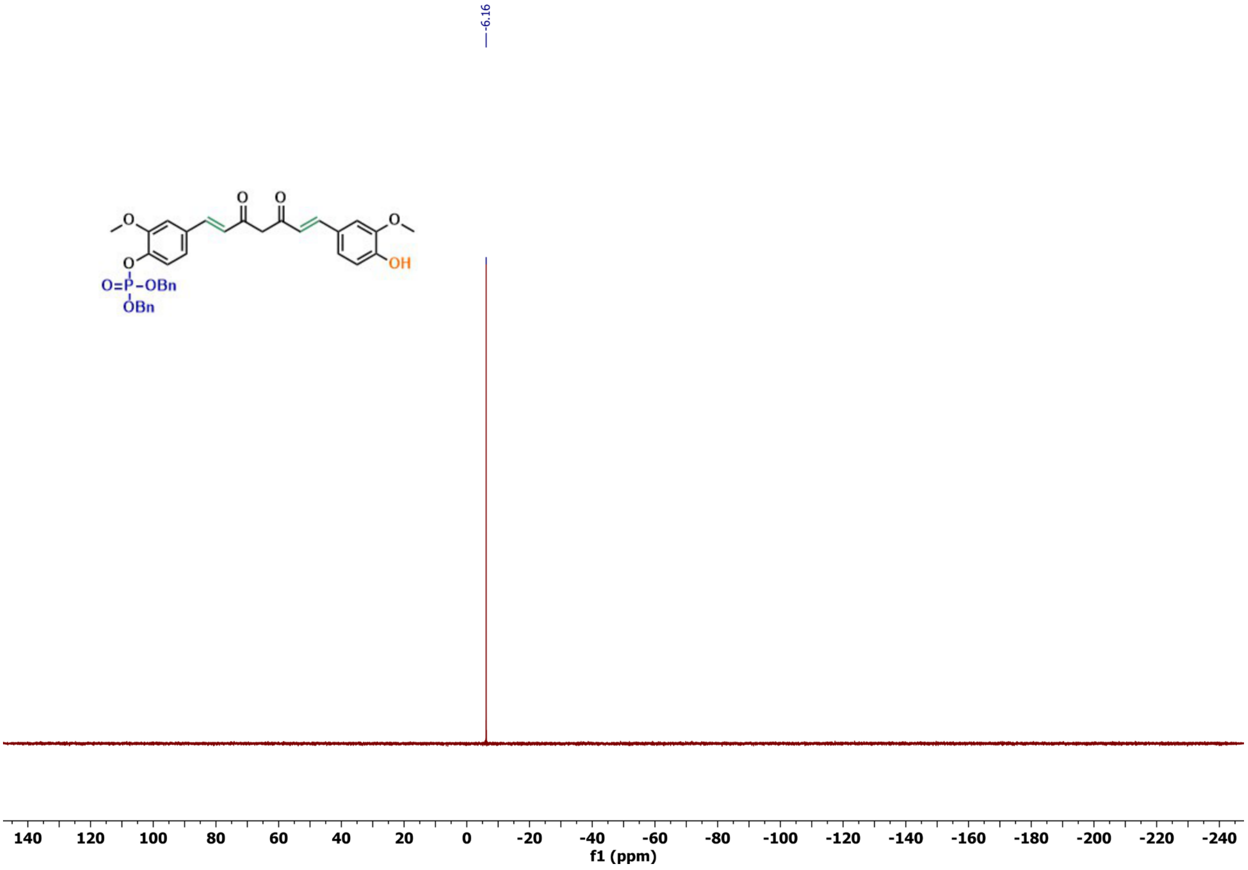

^{1}H NMR of Compound **15b** (600 MHz, MeOD)

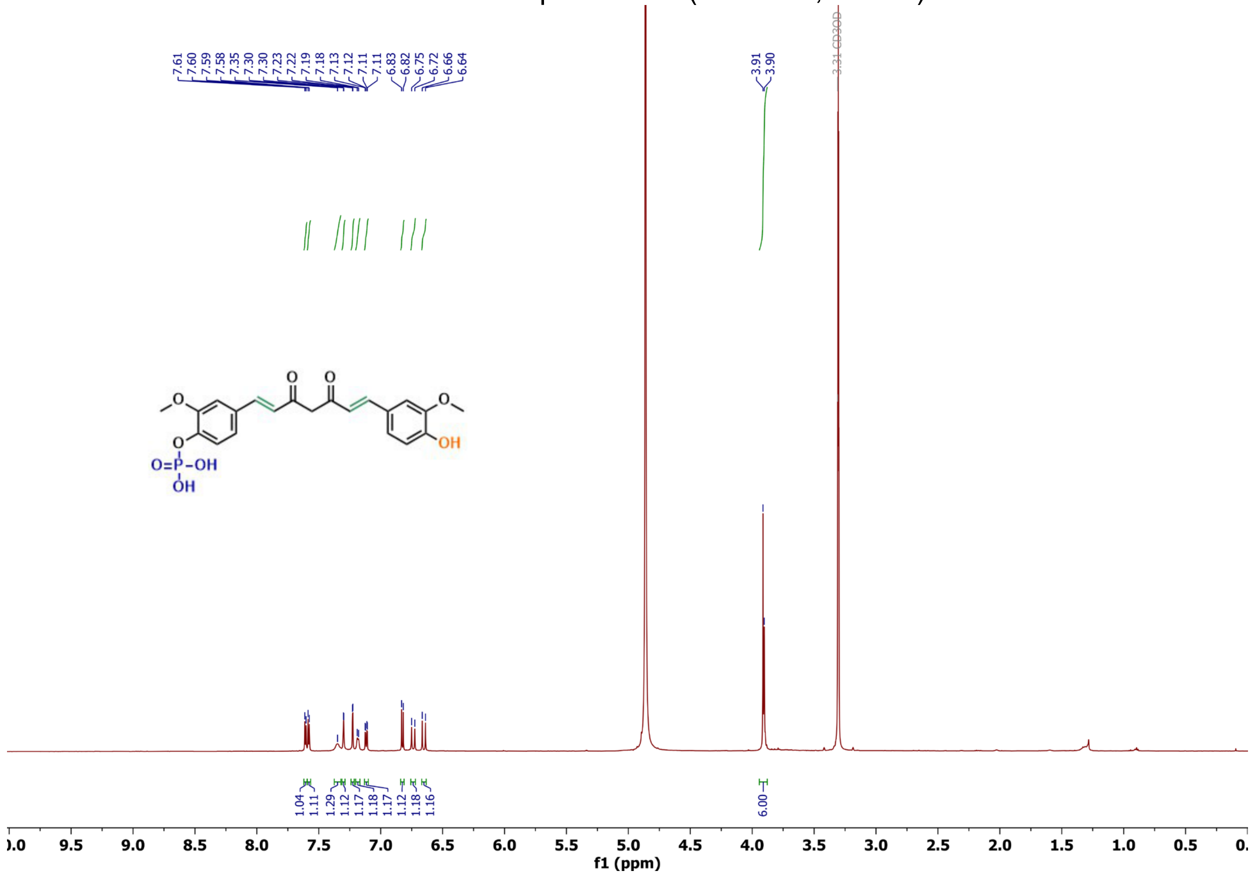

^{13}C NMR of Compound **15b** (151 MHz, MeOD)

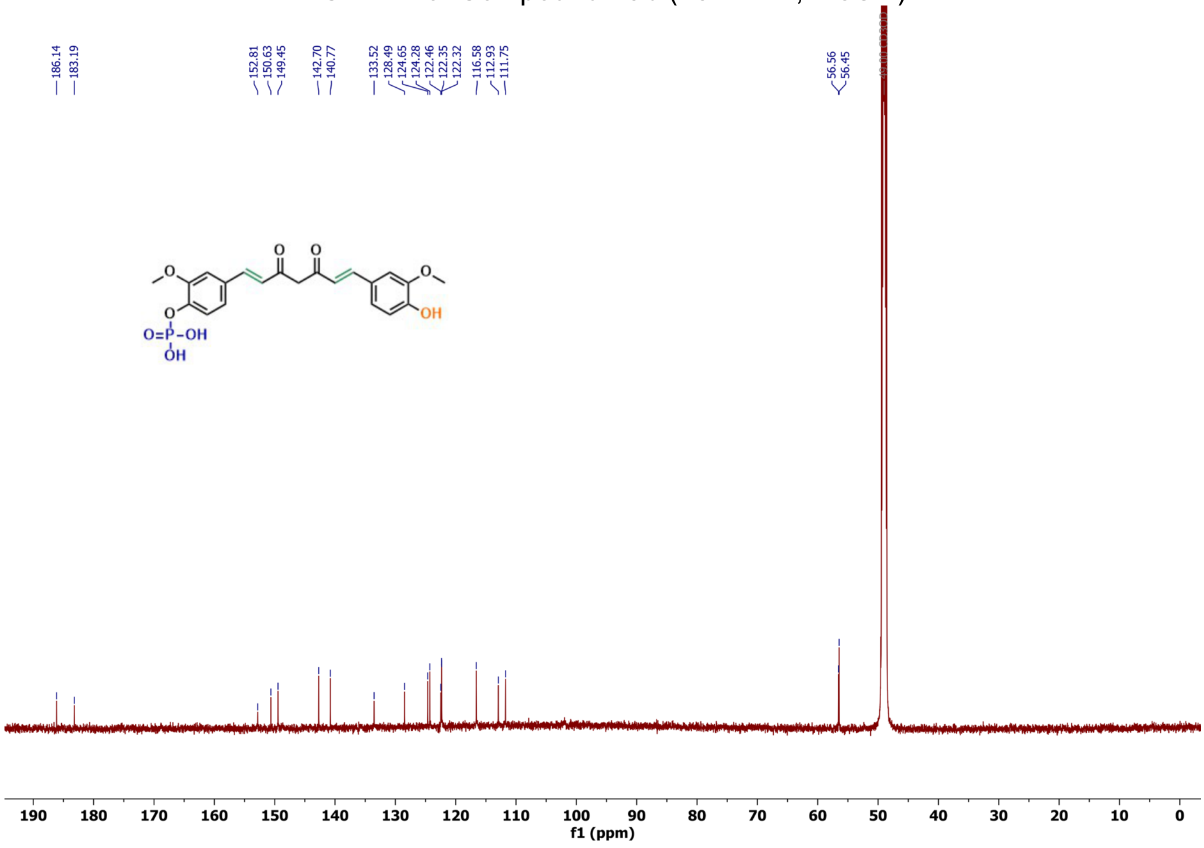

^{31}P NMR of Compound **15b** (243 MHz, MeOD)

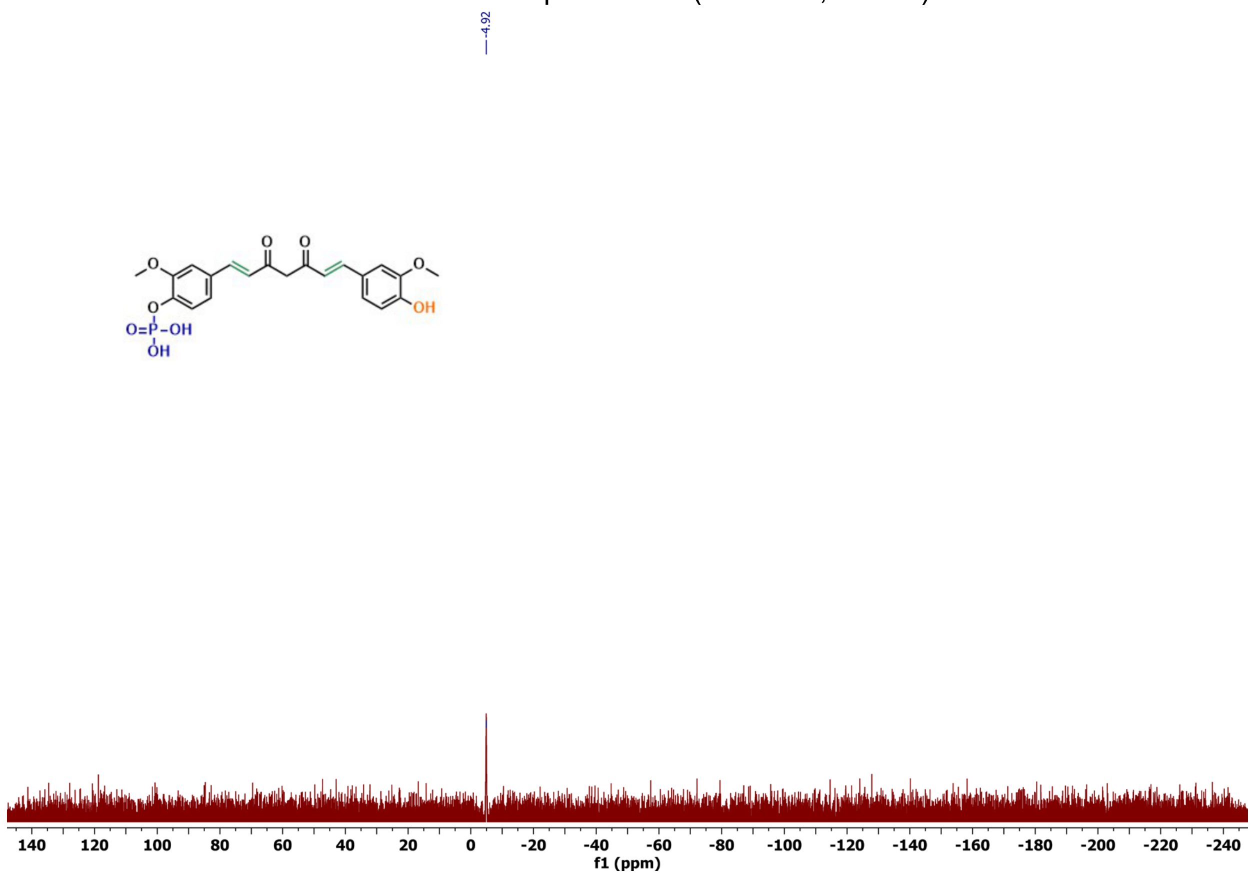

^{1}H NMR of Compound **16a** (400 MHz, $CDCl_3$)

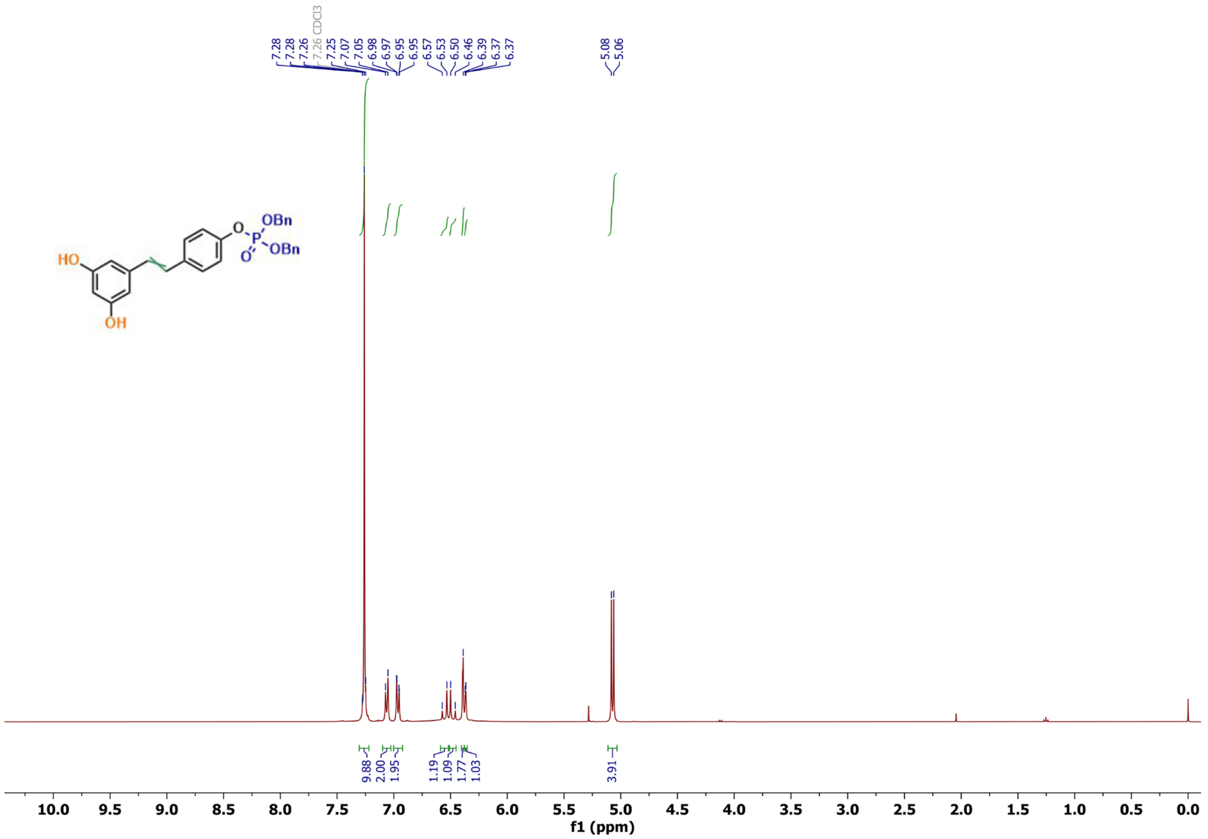

^{13}C NMR of Compound **16a** (151 MHz, DMSO-d_6)

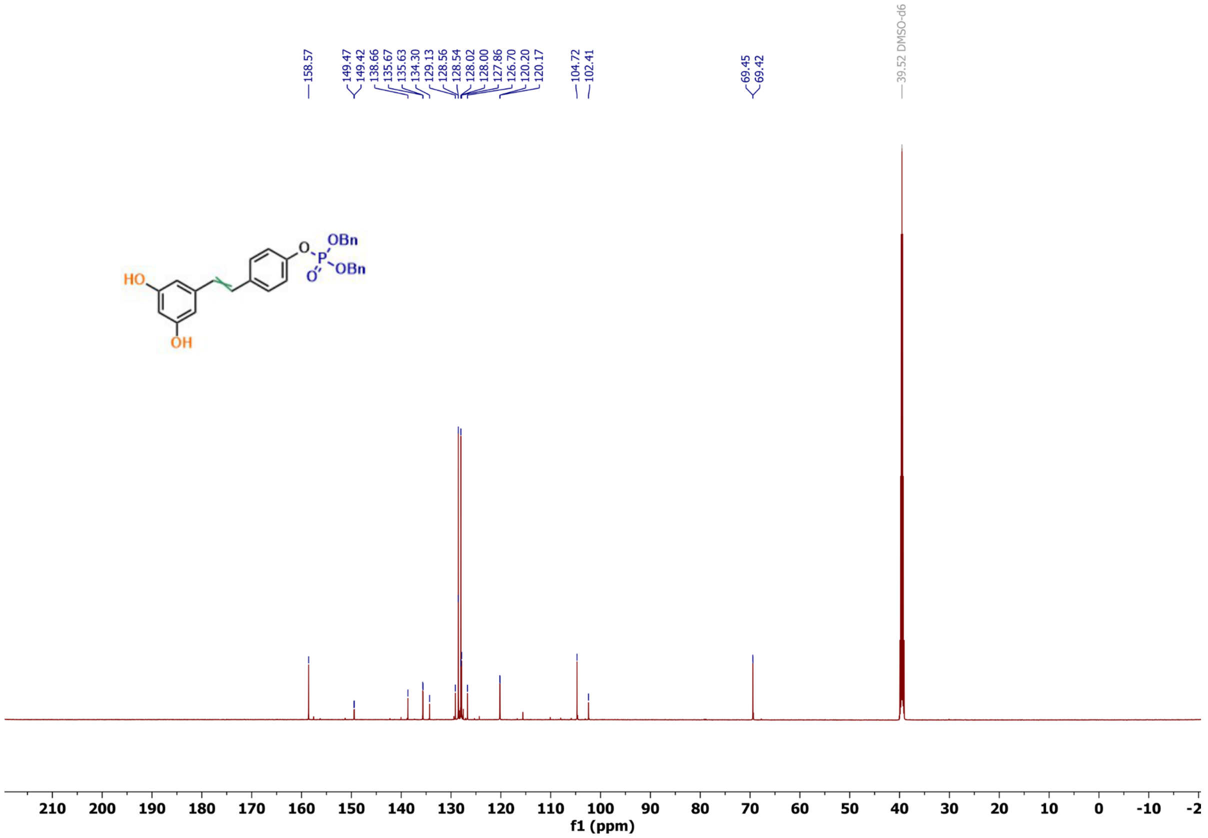

^{31}P NMR of Compound **16a** (162 MHz, $CDCl_3$)

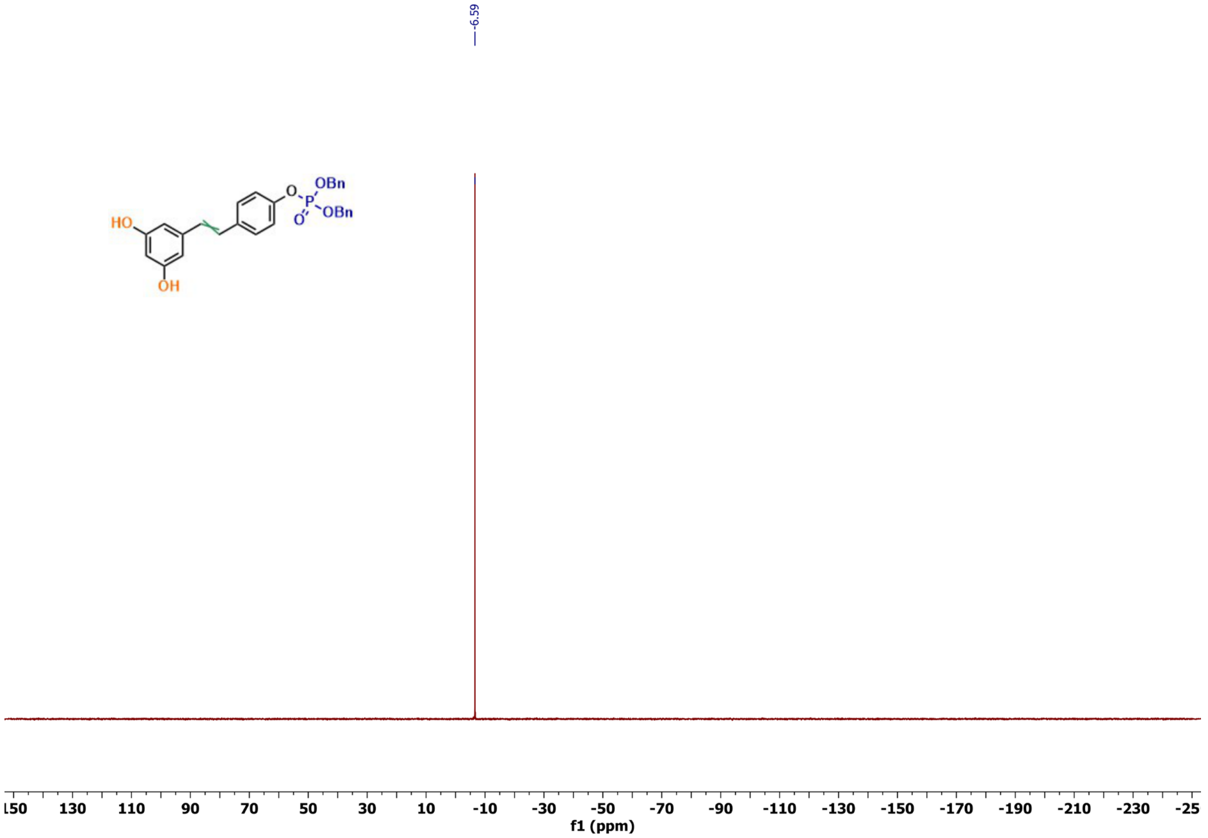

^{1}H NMR of Compound **17a** (600 MHz, $CDCl_3$)

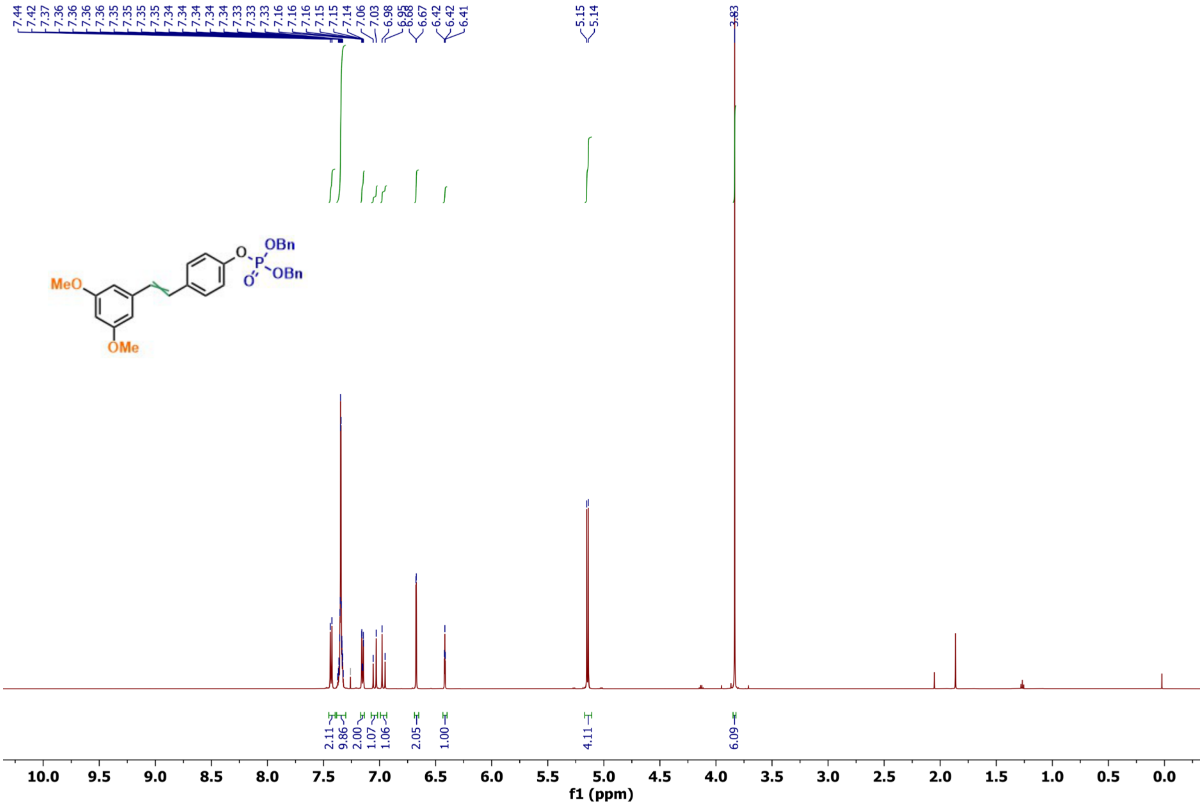

^{13}C NMR of Compound **17a** (151 MHz, $CDCl_3$)

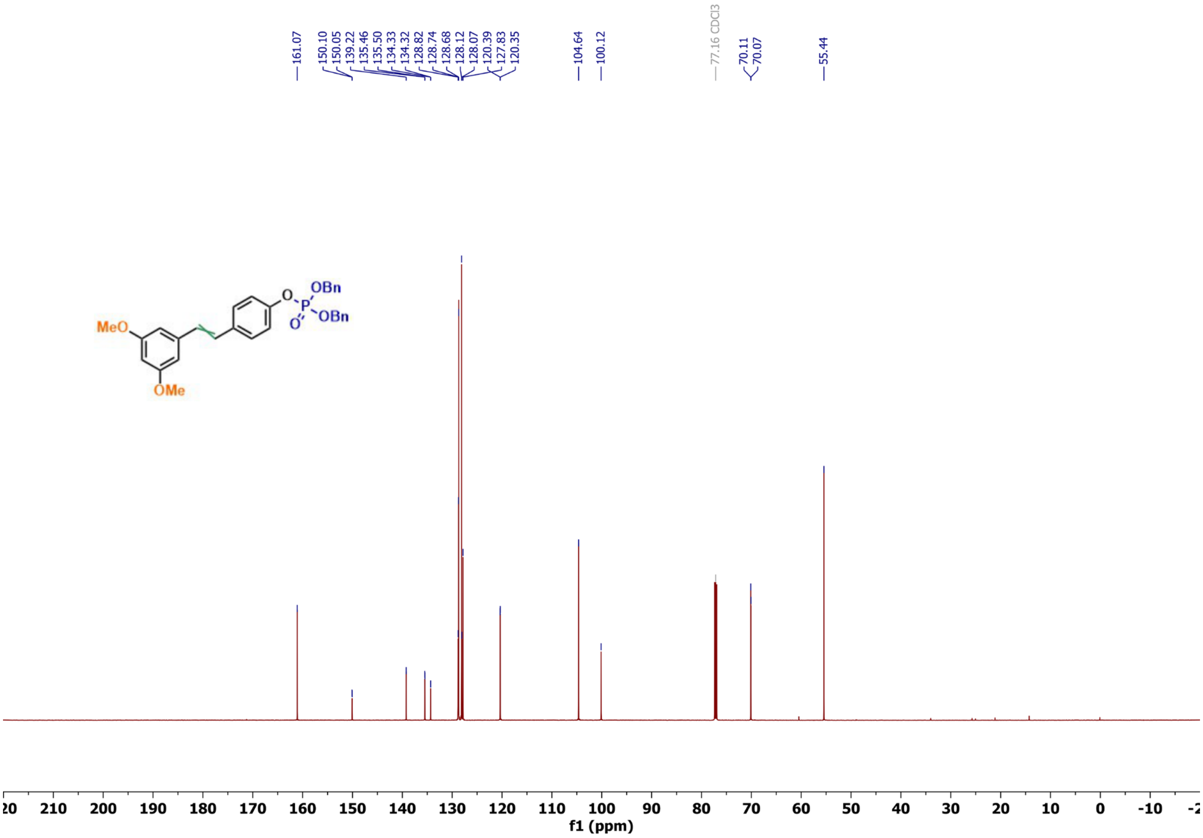

^{31}P NMR of Compound **17a** (243 MHz, $CDCl_3$)

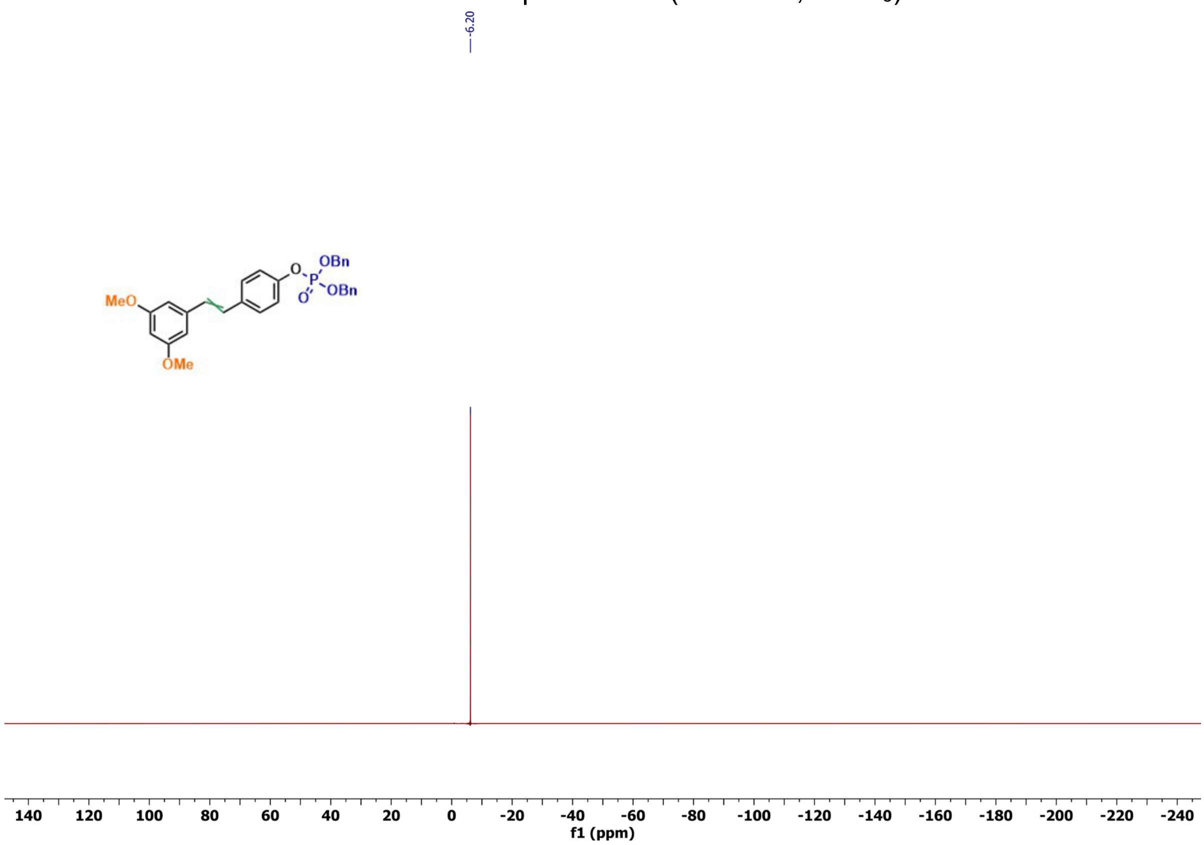

^{1}H NMR of Compound **17b** (600 MHz, $CDCl_3$)

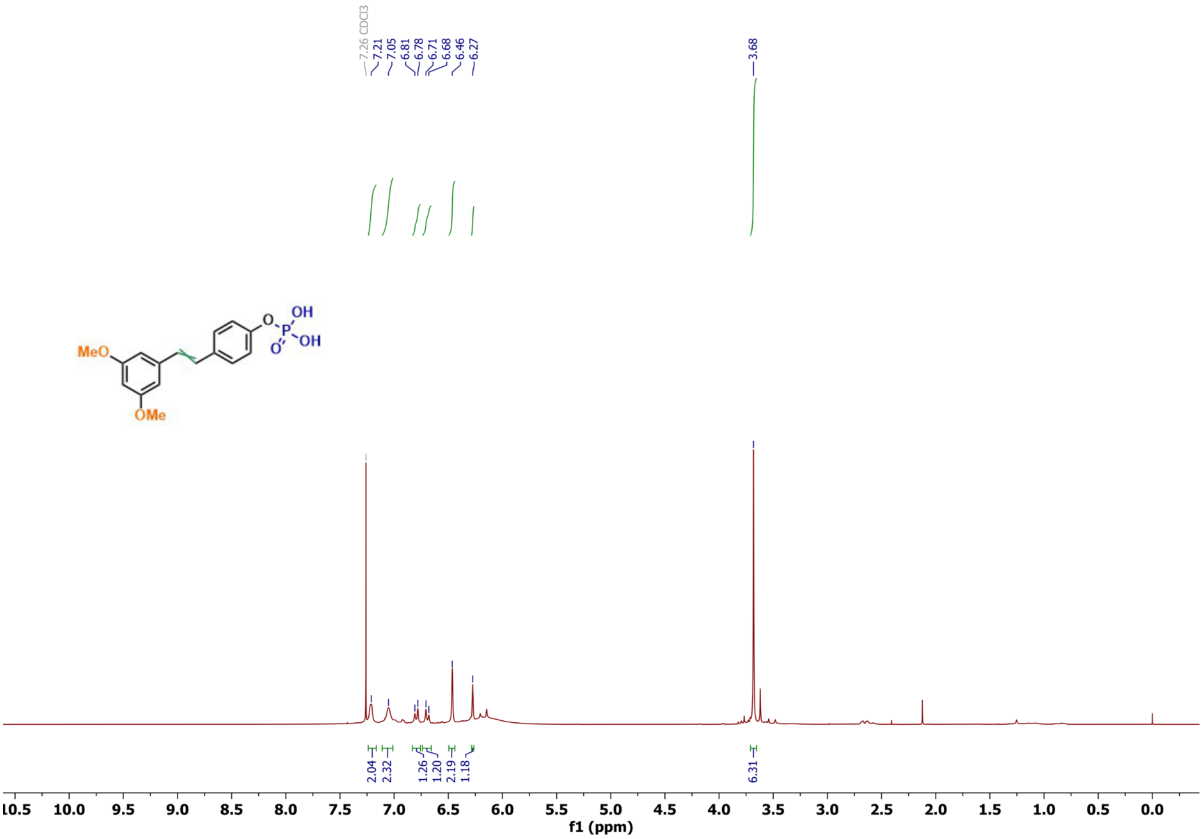

^{13}C NMR of Compound **17a** (151 MHz, $CDCl_3$)

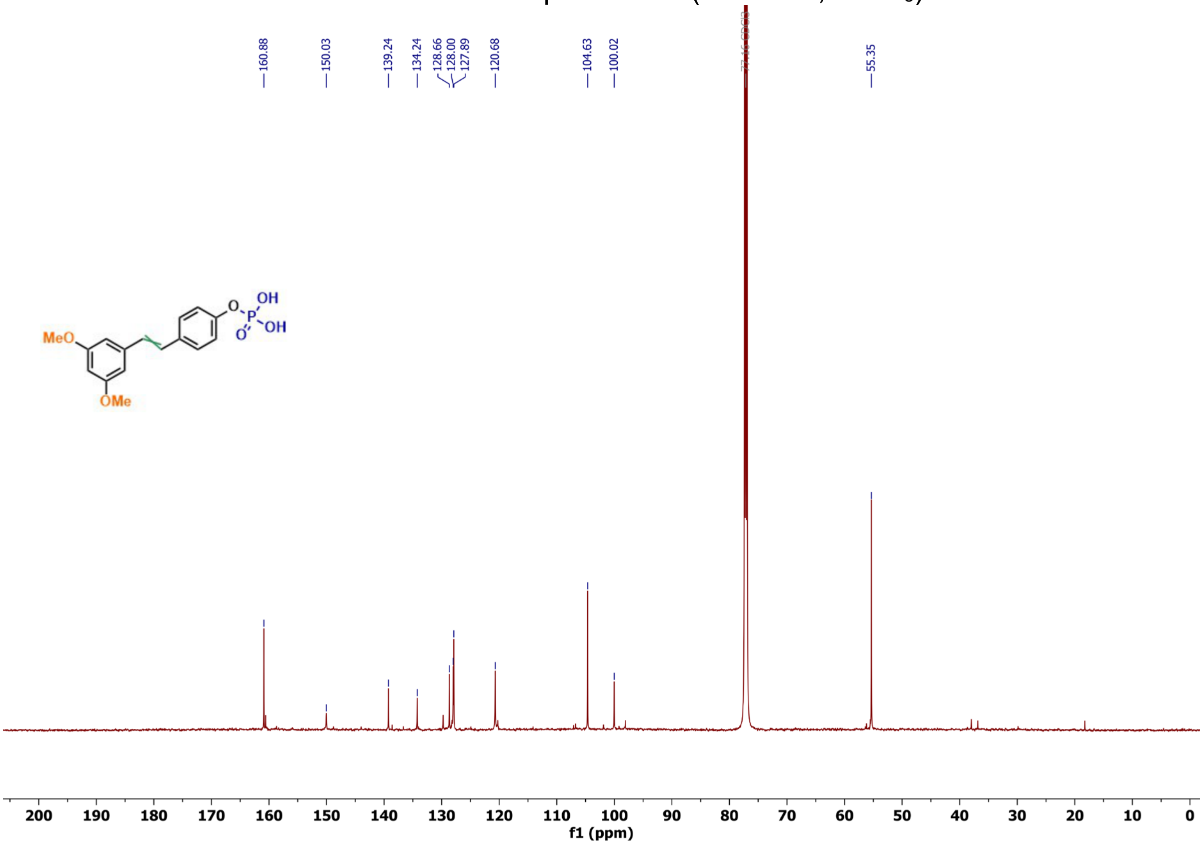

^{31}P NMR of Compound **17b** (243 MHz, $CDCl_3$)

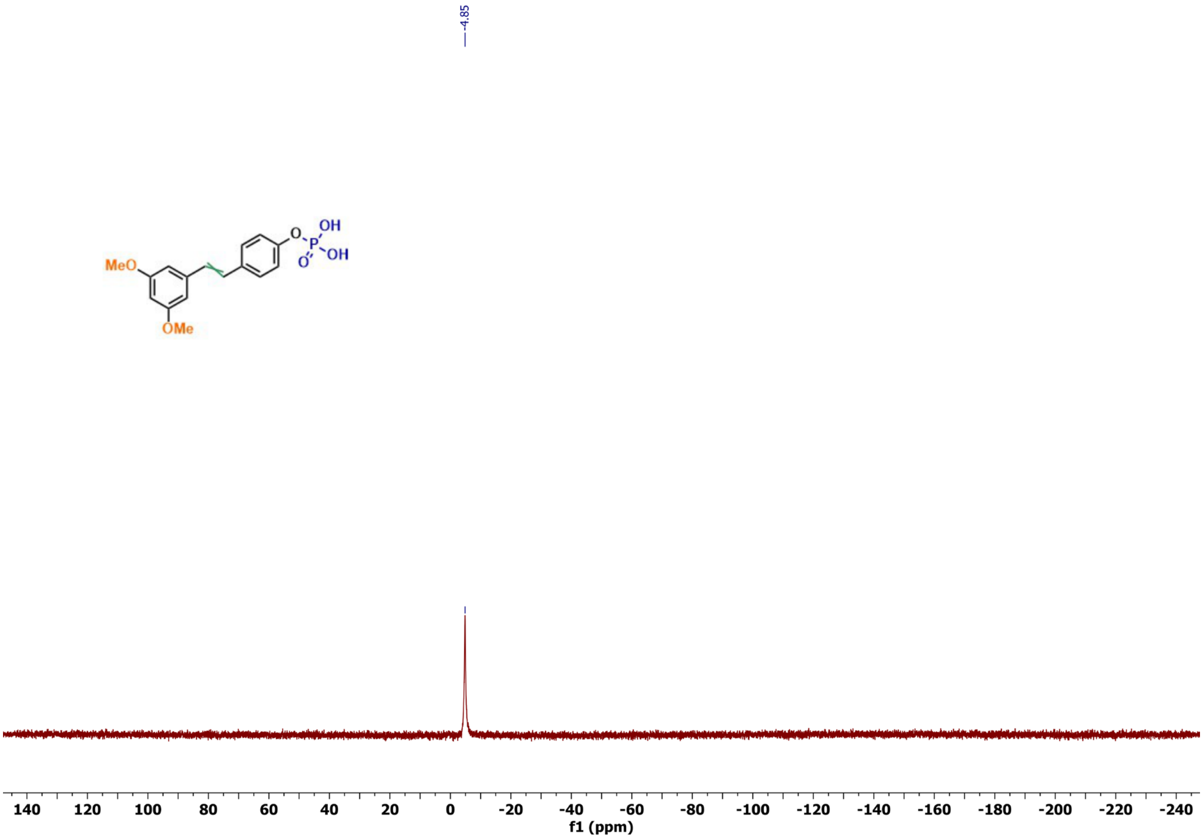

^{1}H NMR of Compound **19a** (600 MHz, $CDCl_3$)

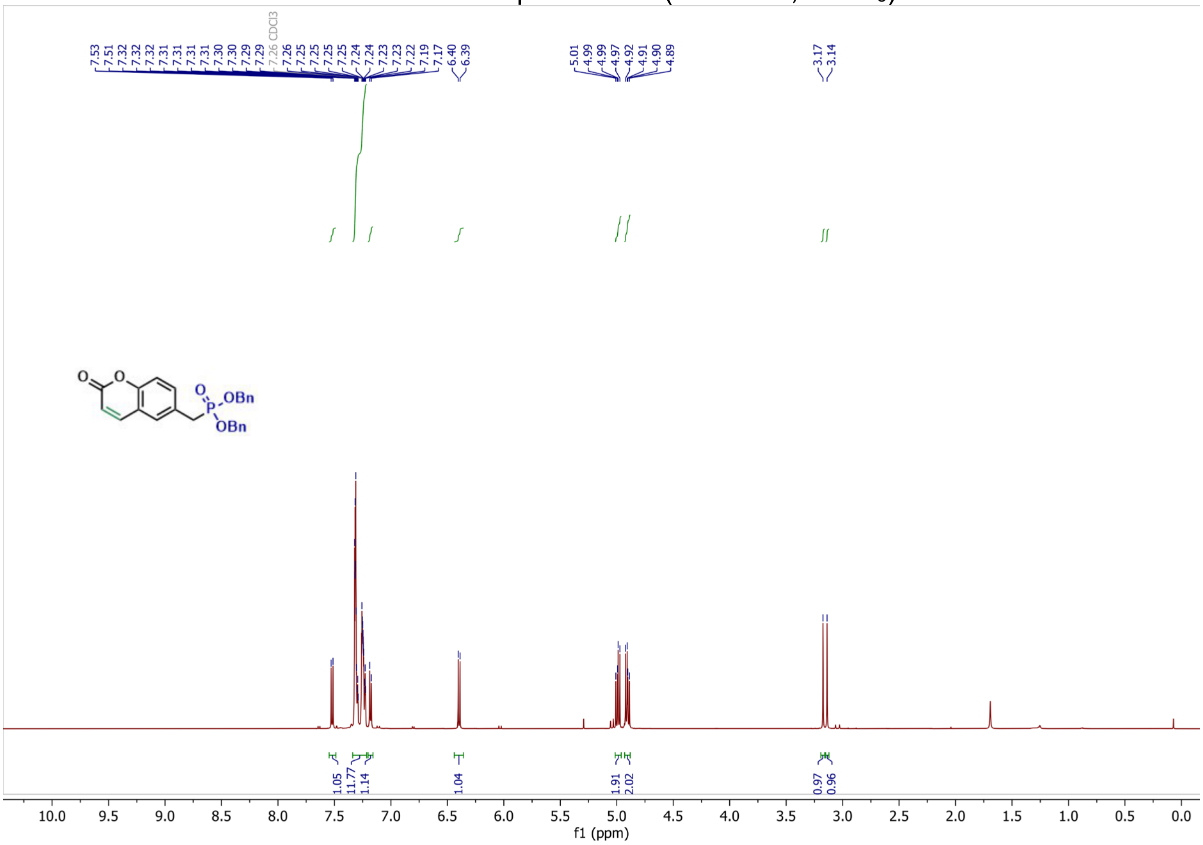

^{13}C NMR of Compound **19a** (151 MHz, $CDCl_3$)

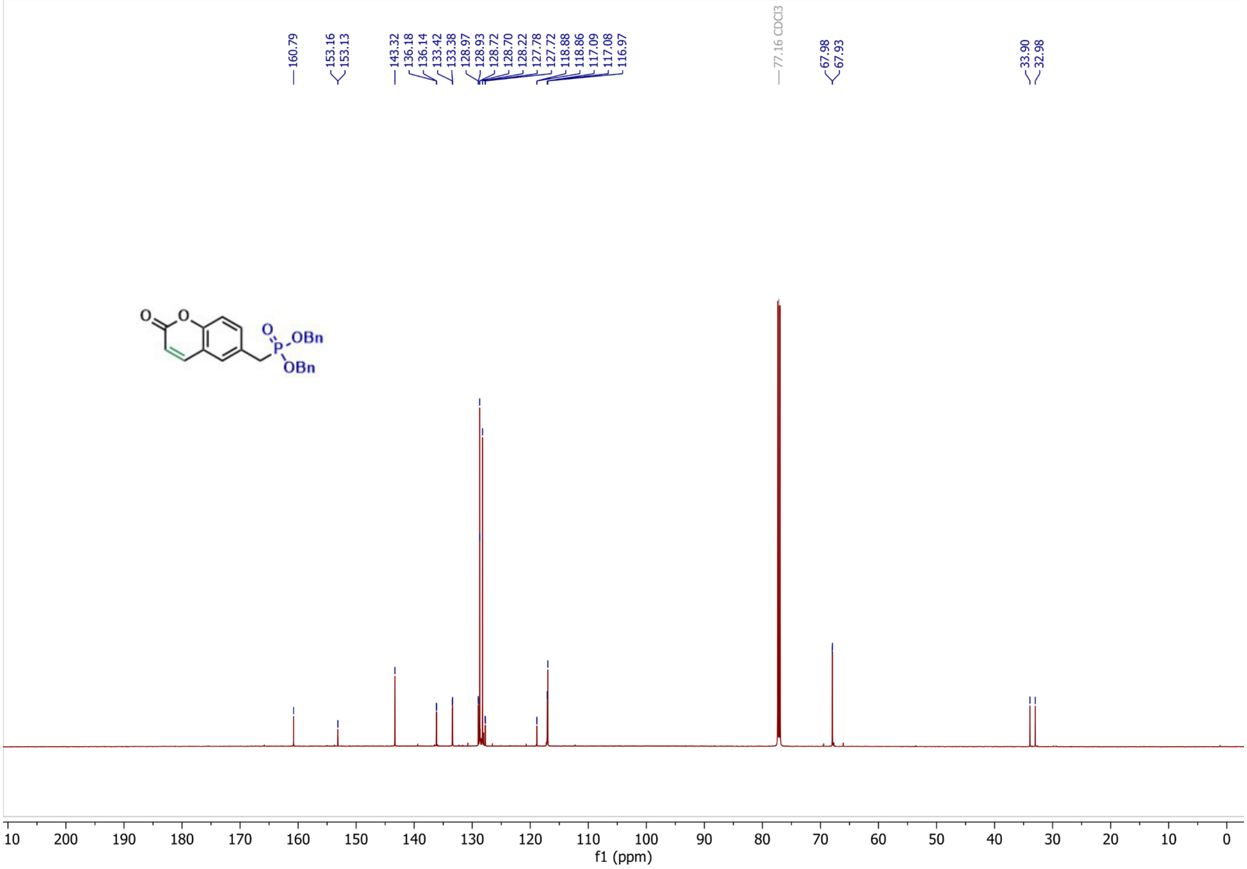

^{31}P NMR of Compound **19a** (243 MHz, CDCl$_3$)

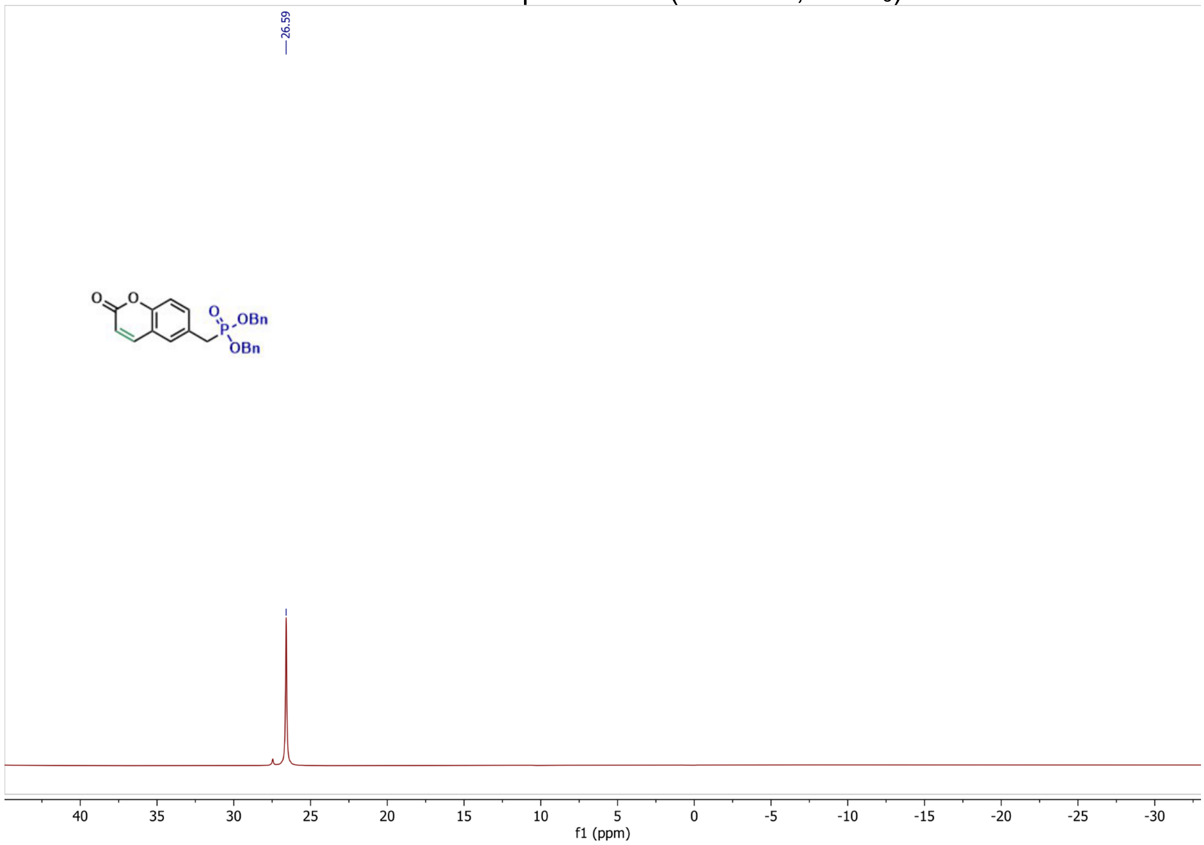

^{1}H NMR of Compound **19b** (400 MHz, CDCl$_3$)

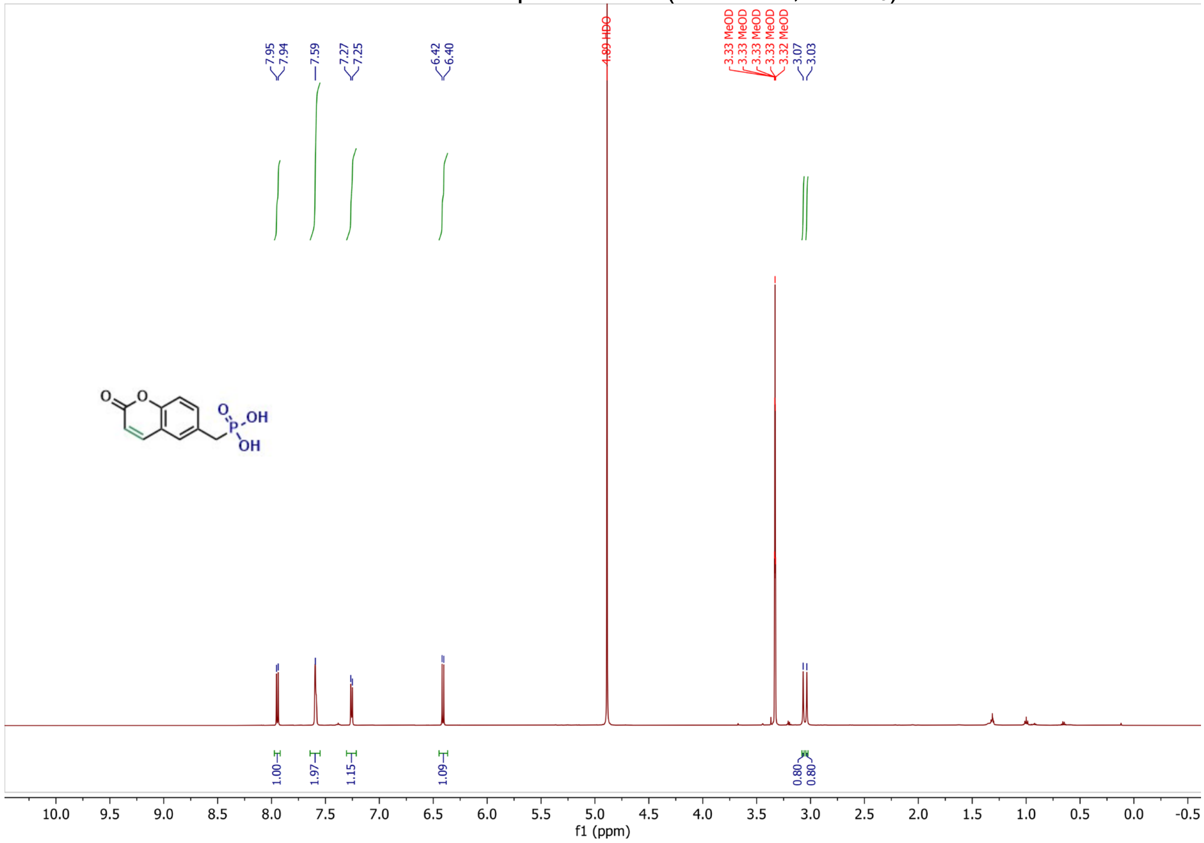

^{13}C NMR of Compound **19b** (125 MHz, $CDCl_3$)

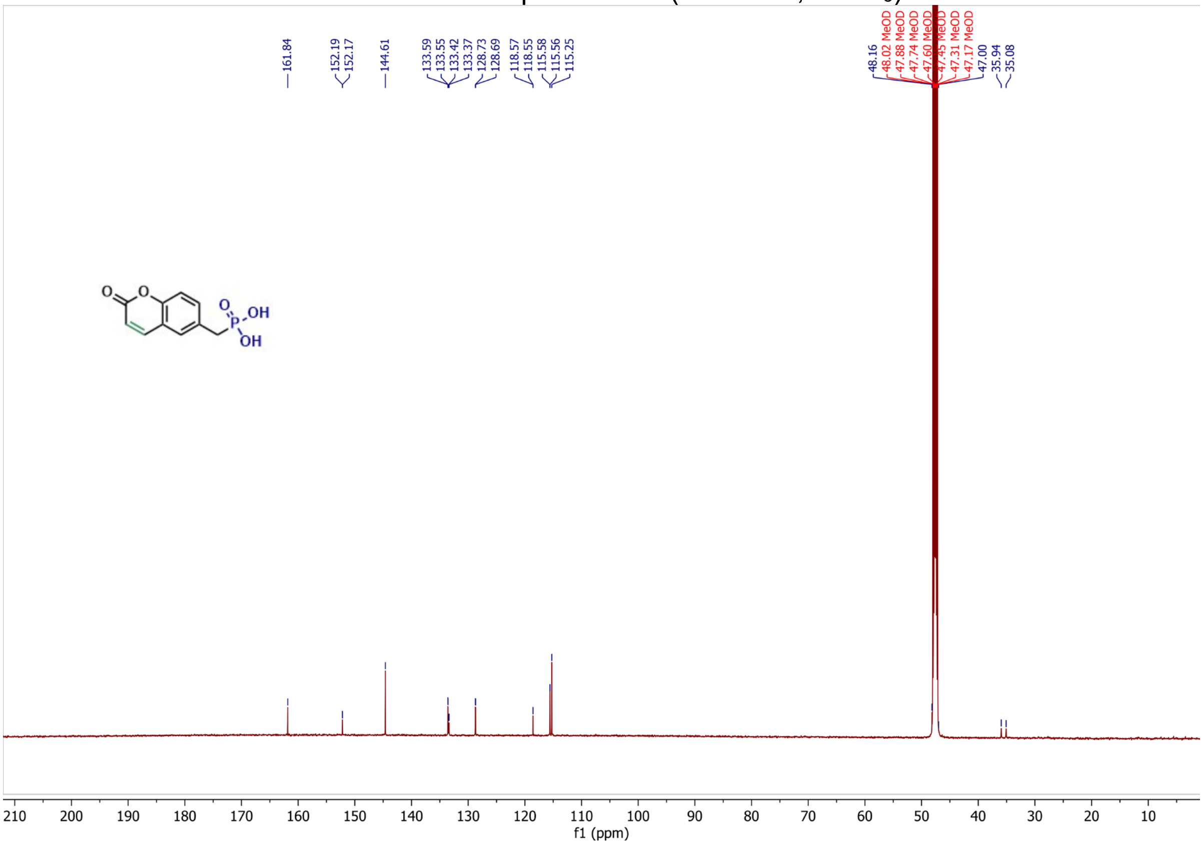

^{31}P NMR of Compound **19b** (162 MHz, $CDCl_3$)

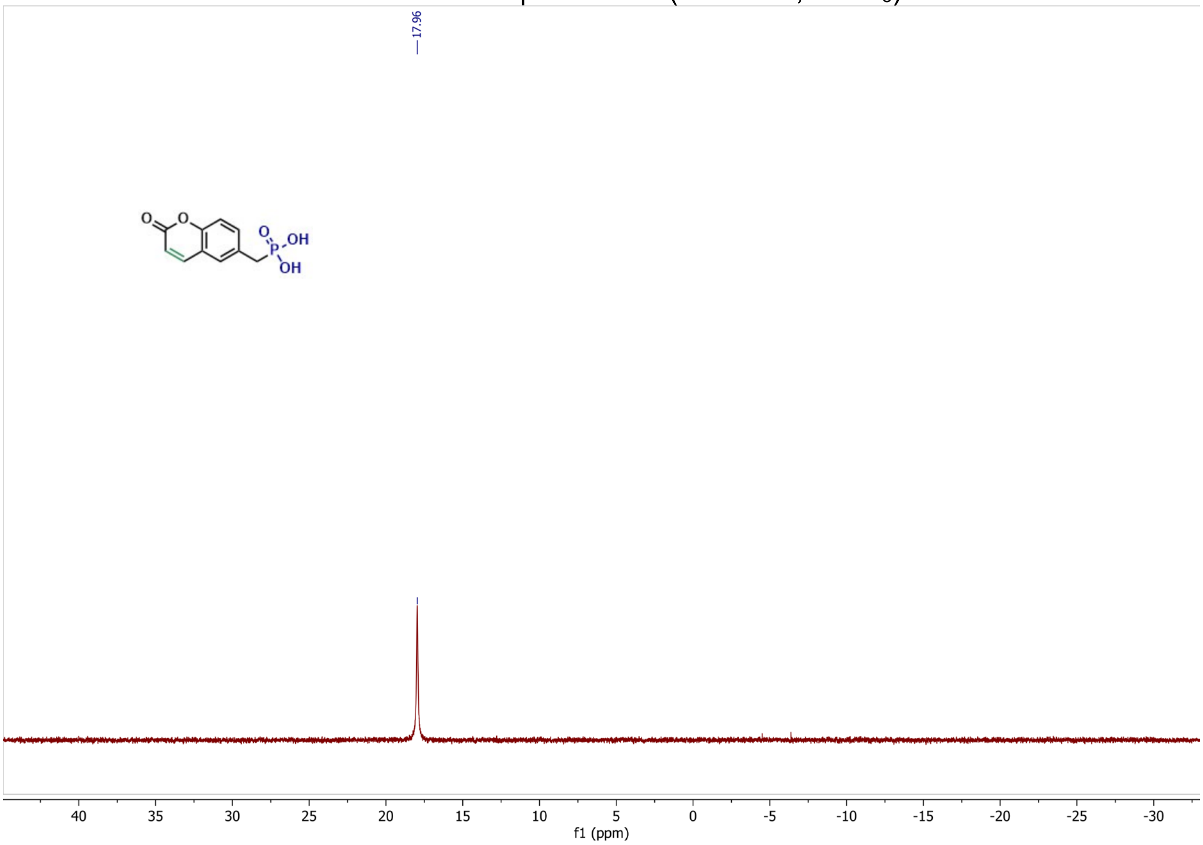

^{1}H NMR of Compound **20a** (800 MHz, $CDCl_3$)

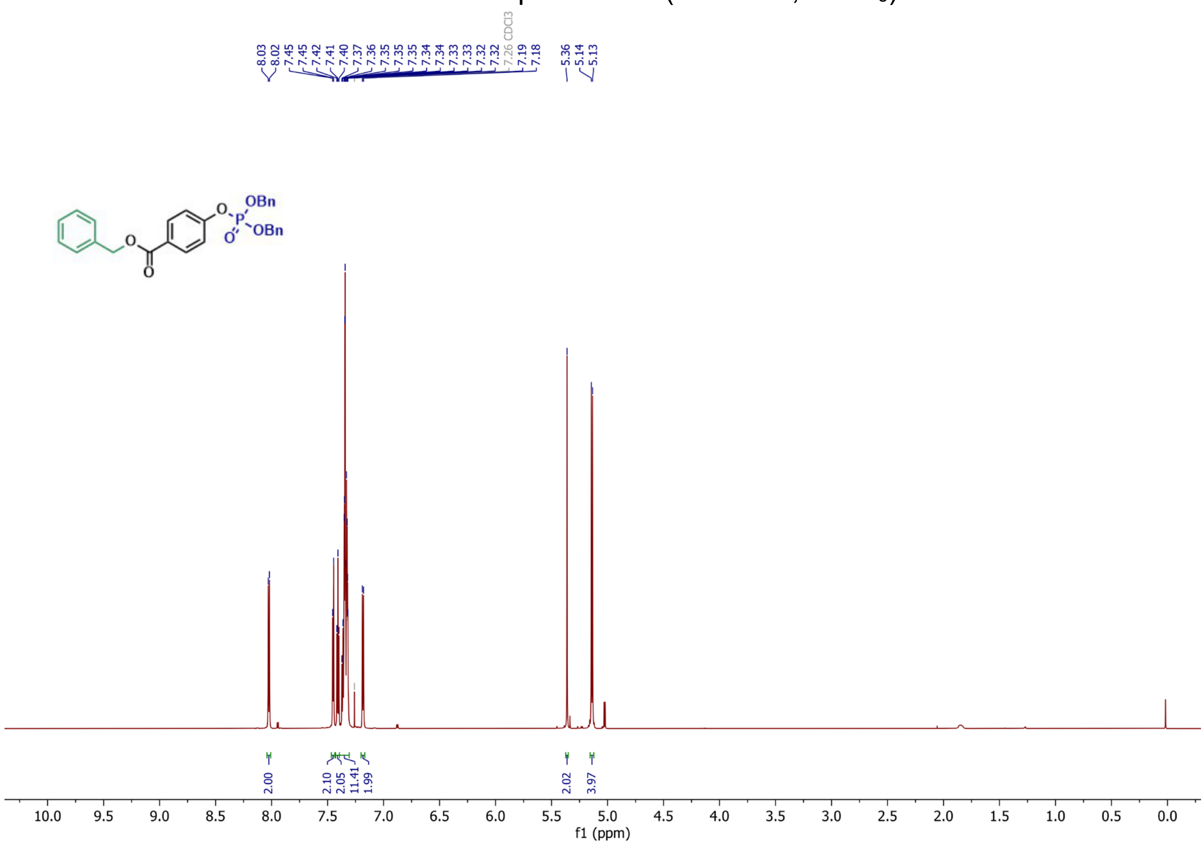

^{13}C NMR of Compound **20a** (201 MHz, $CDCl_3$)

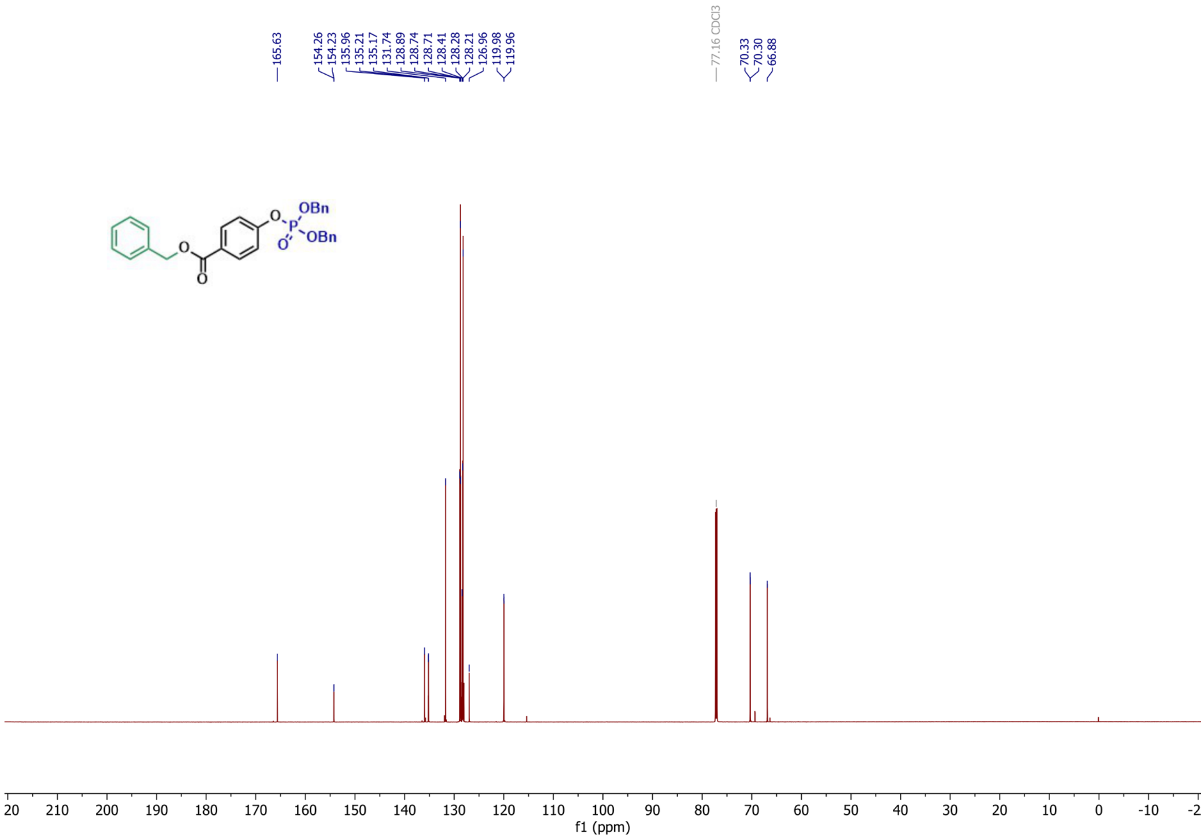

^{31}P NMR of Compound **20a** (162 MHz, $CDCl_3$)

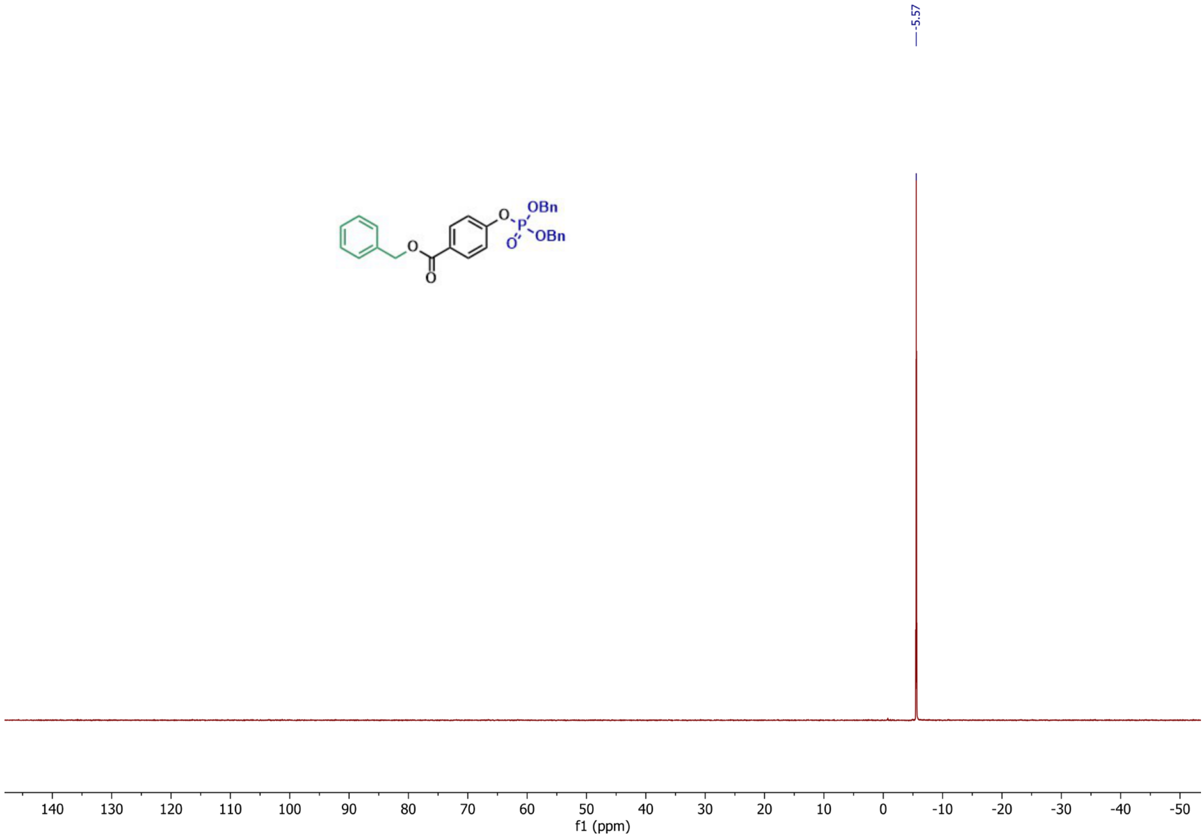

^{1}H NMR of Compound **20b** (400 MHz, acetone-d_6)

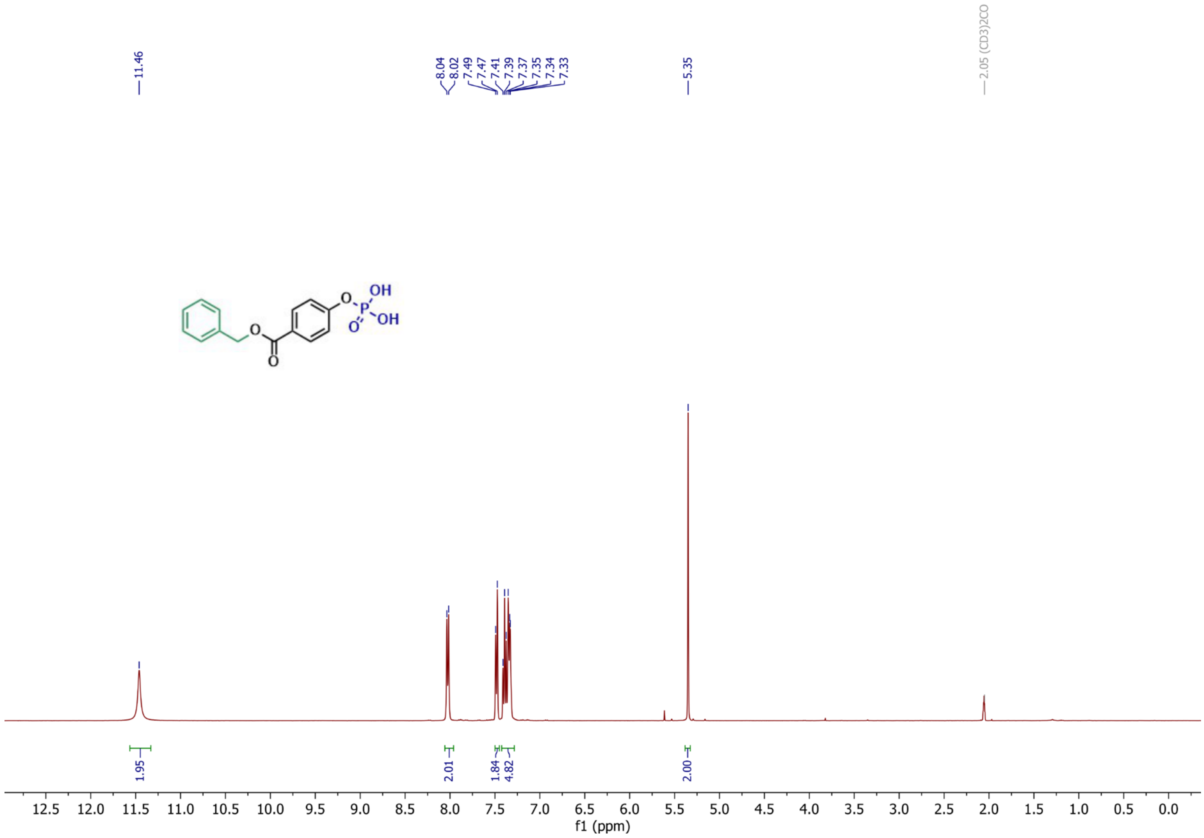

^{13}C NMR of Compound **20b** (101 MHz, acetone-d_6)

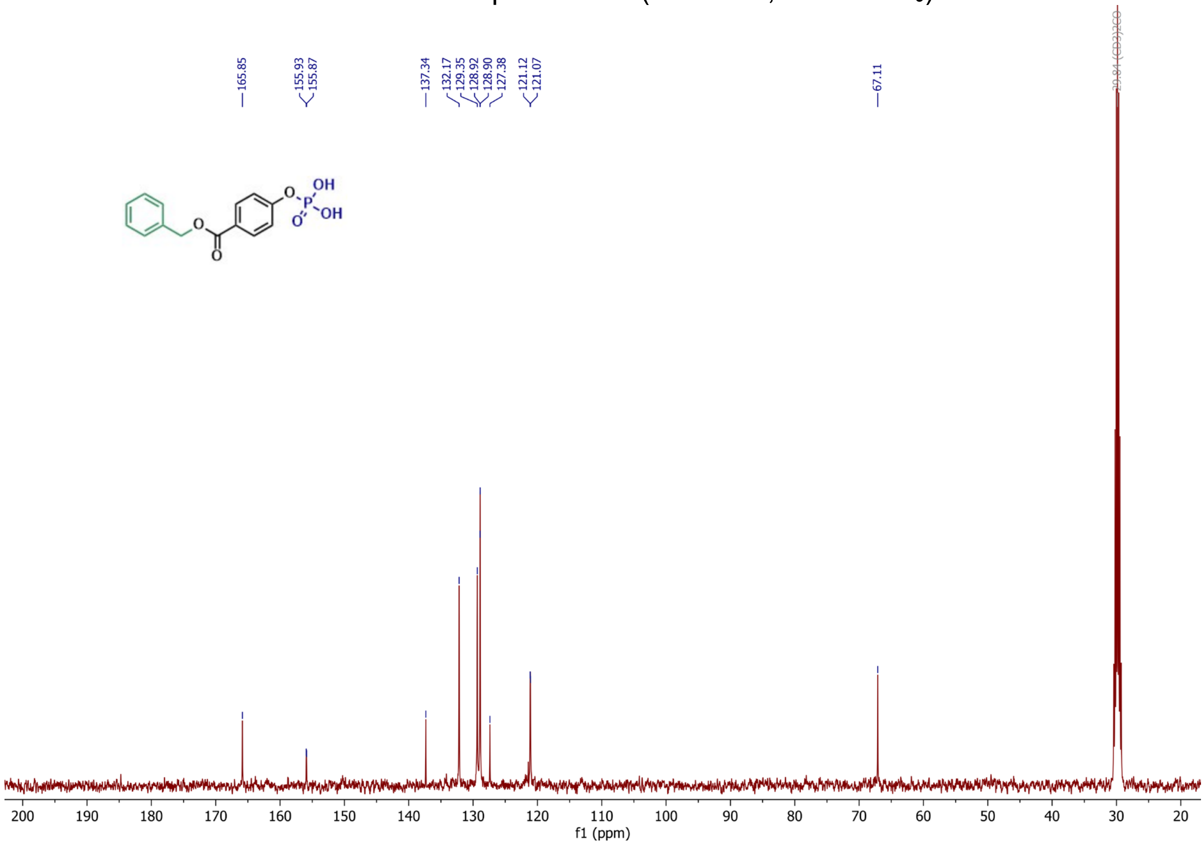

^{31}P NMR of Compound **20b** (162 MHz, acetone-d_6)

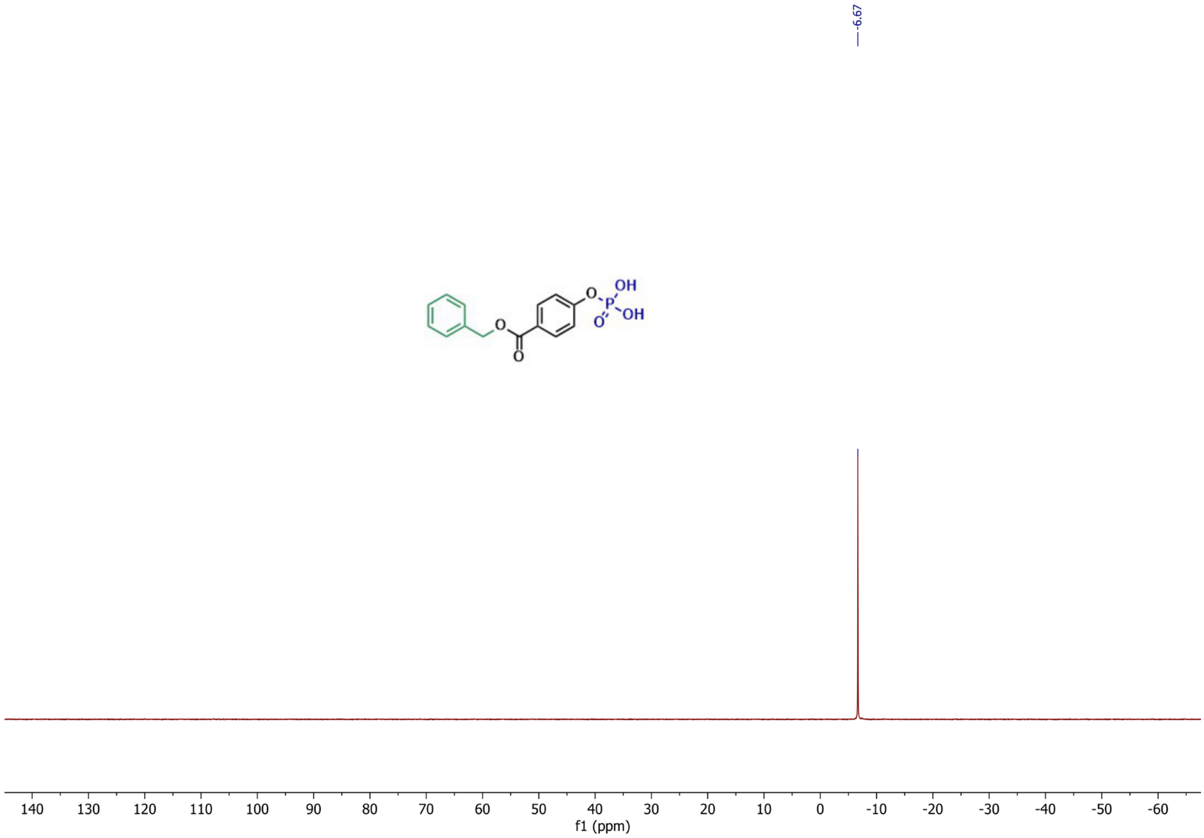

^{1}H NMR of Compound **20c** (400 MHz, DMSO-d_6)

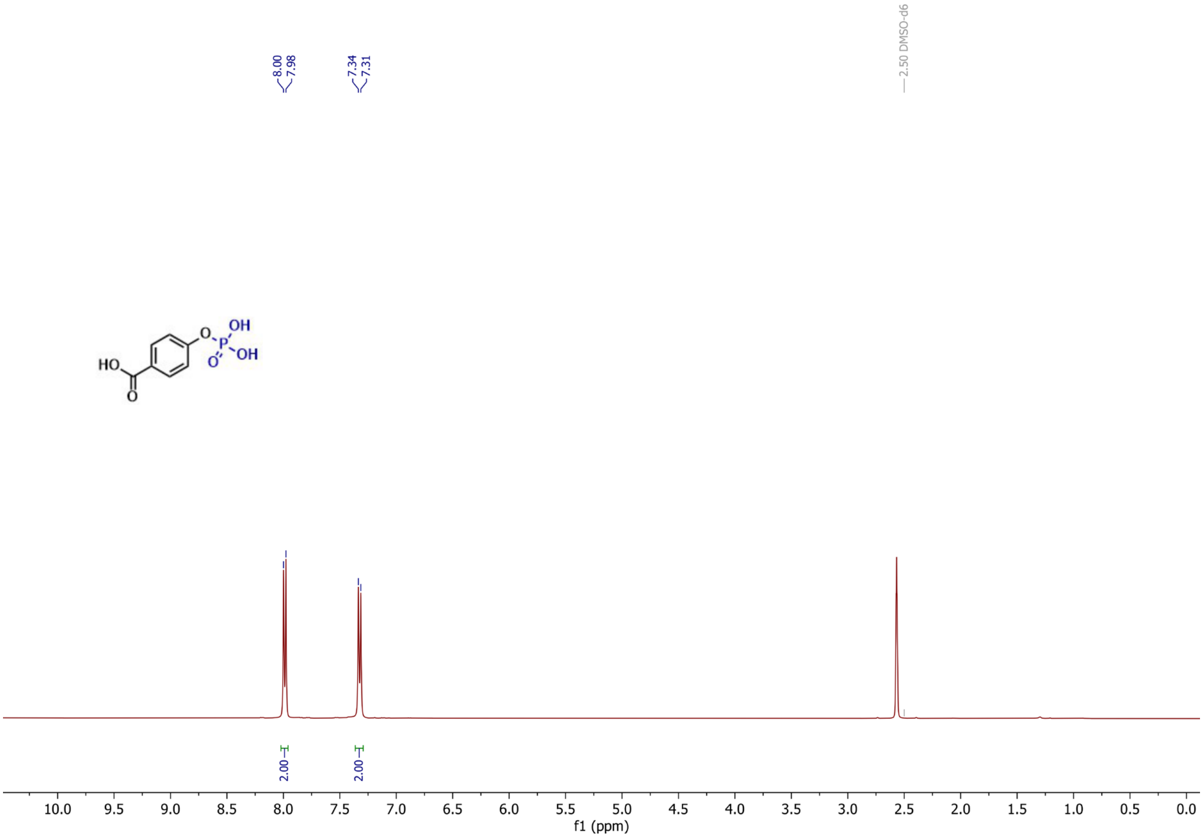

^{13}C NMR of Compound **20c** (125 MHz, DMSO-d_6)

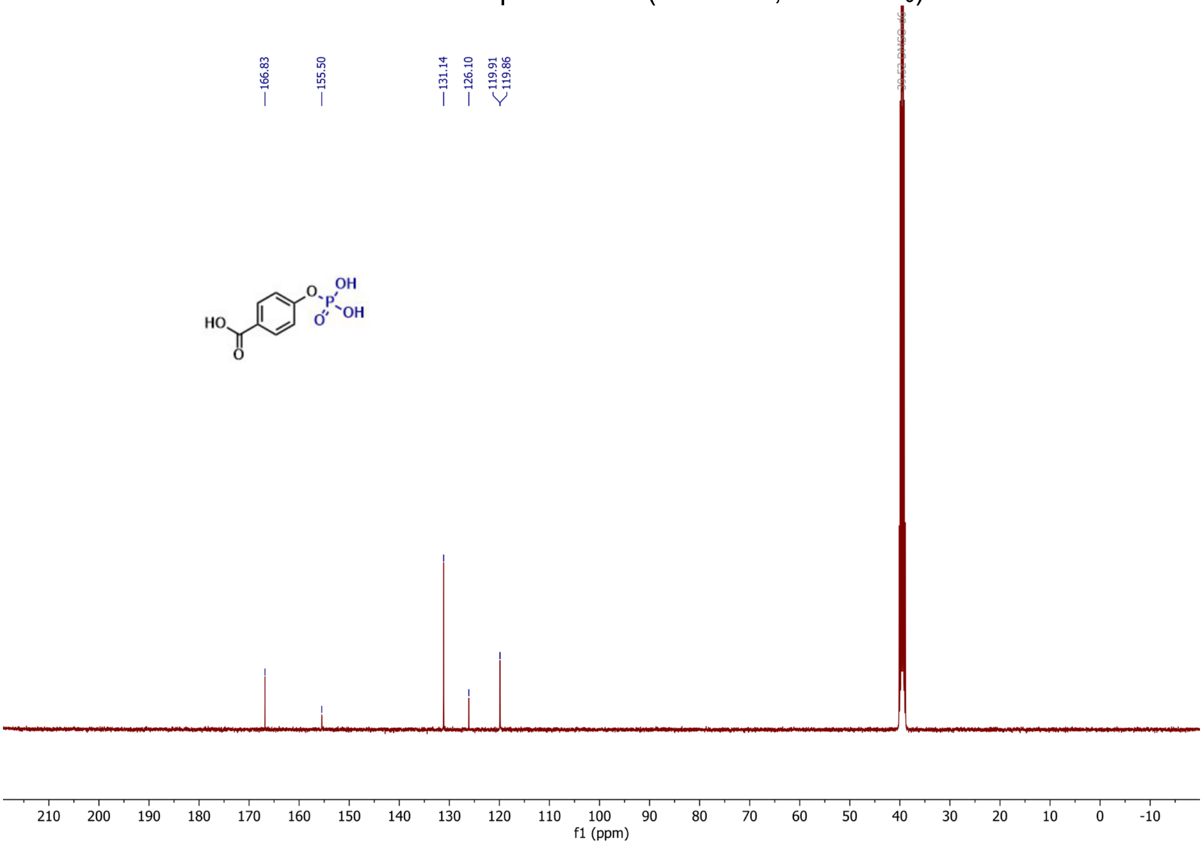

^{31}P NMR of Compound **20c** (162 MHz, DMSO-d_6)

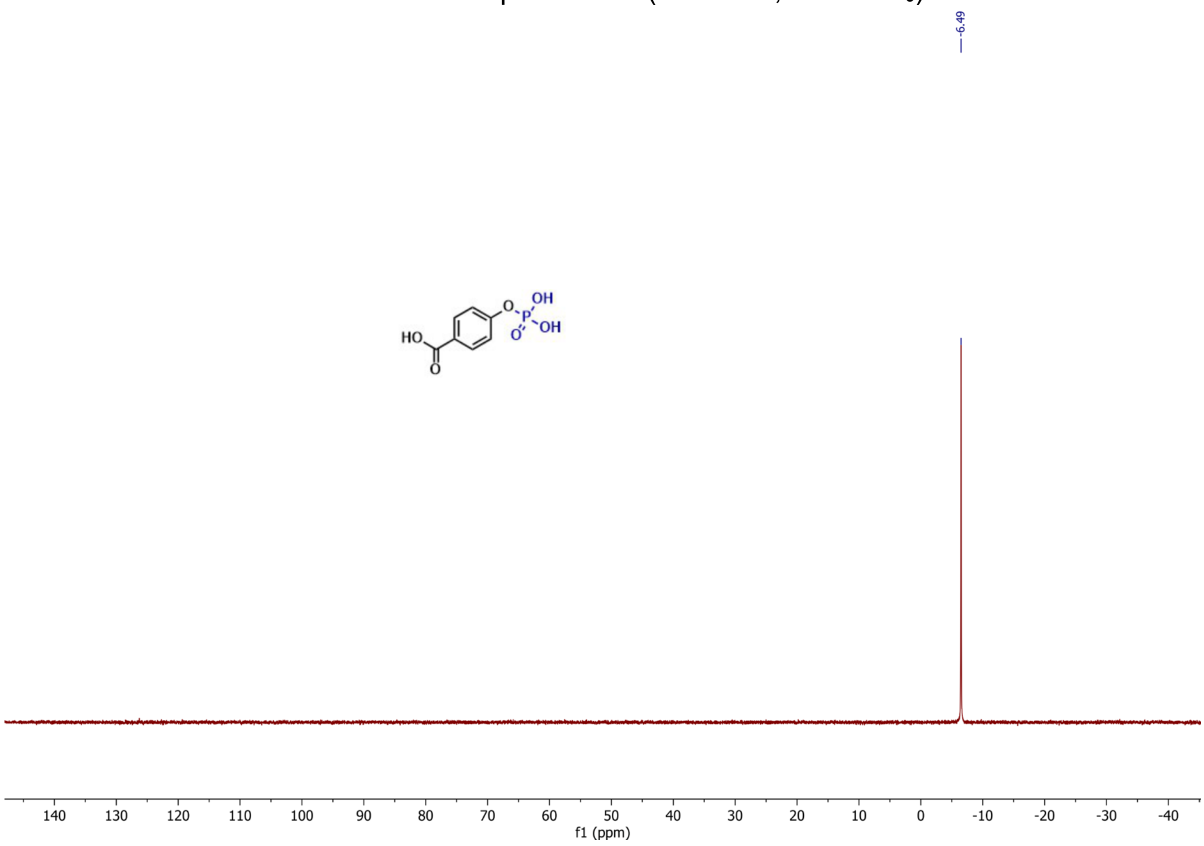

^{1}H NMR of Compound **21a** (400 MHz, $CDCl_3$)

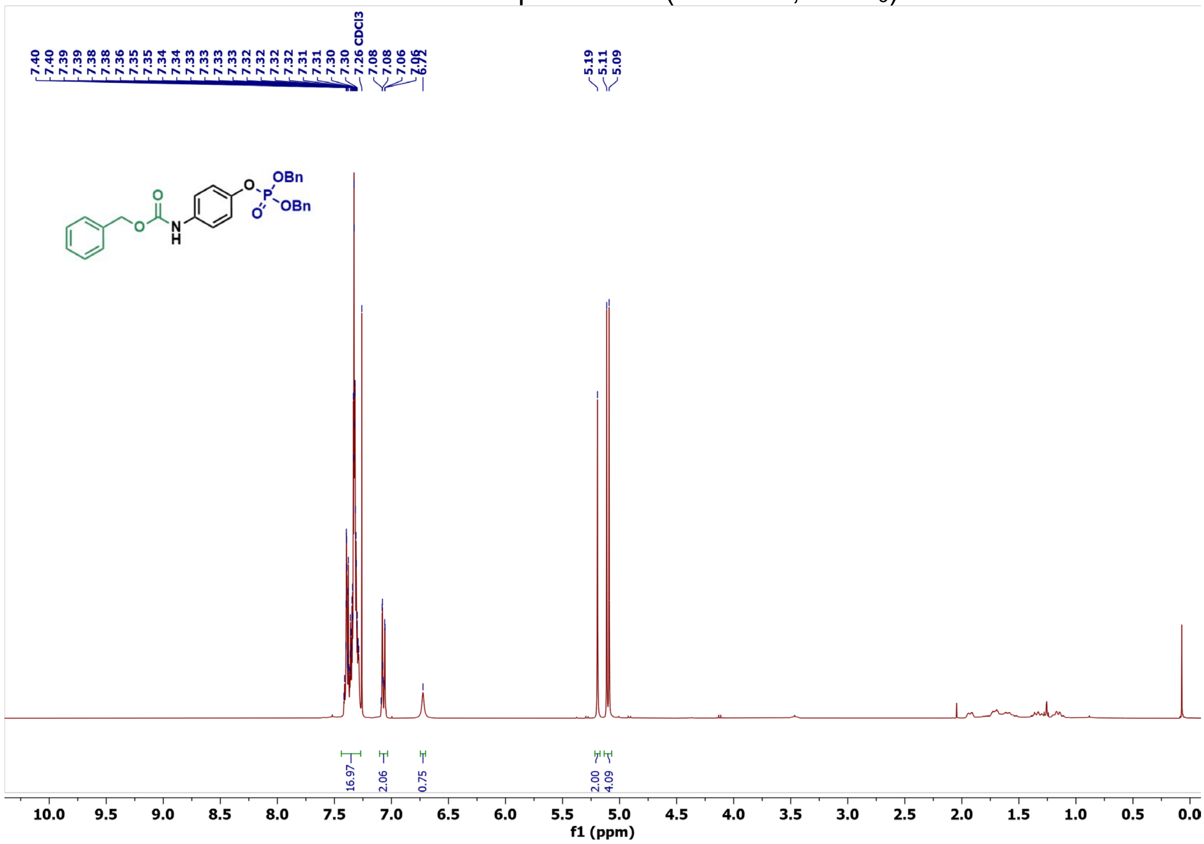

^{13}C NMR of Compound **21a** (125 MHz, $CDCl_3$)

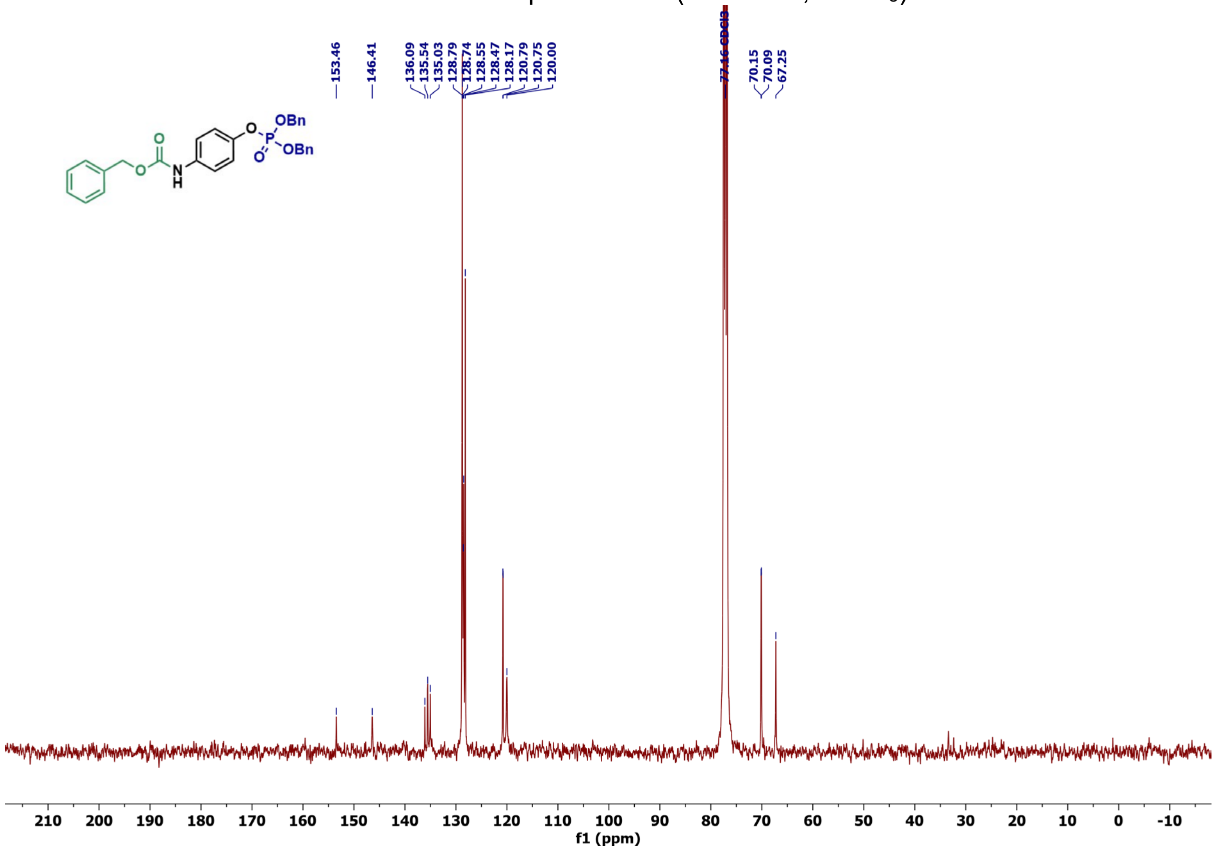

^{31}P NMR of Compound **21a** (162 MHz, $CDCl_3$)

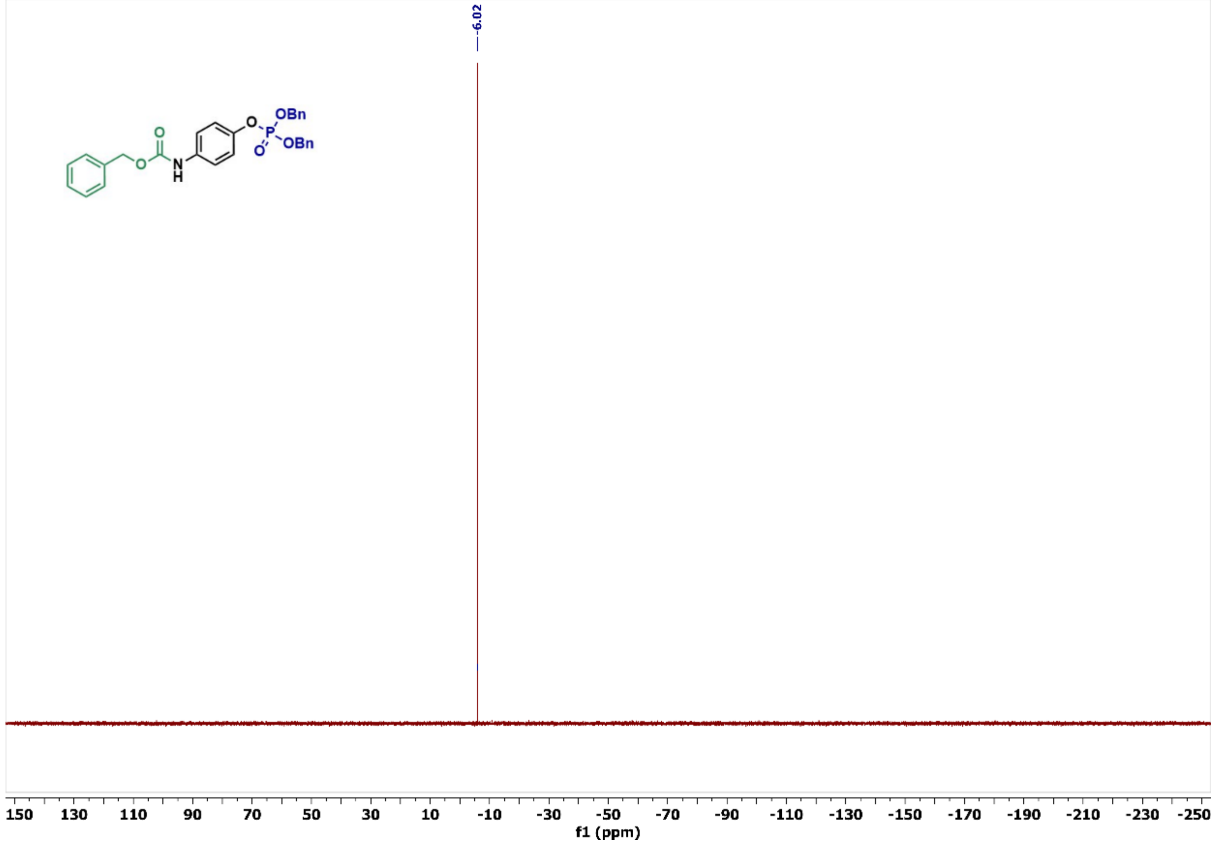

^{1}H NMR of Compound **21b** (400 MHz, DMSO-d_6)

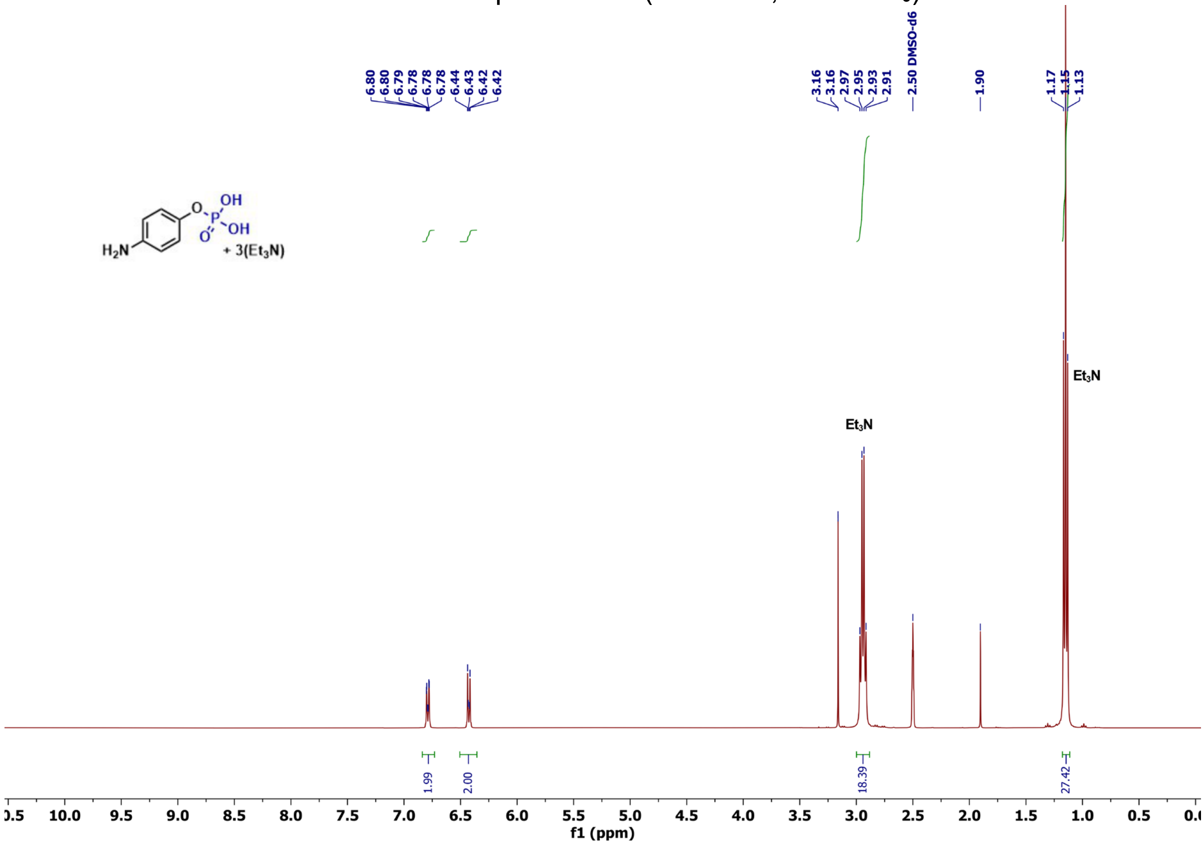

^{13}C NMR of Compound **21b** (101 MHz, DMSO-d_6)

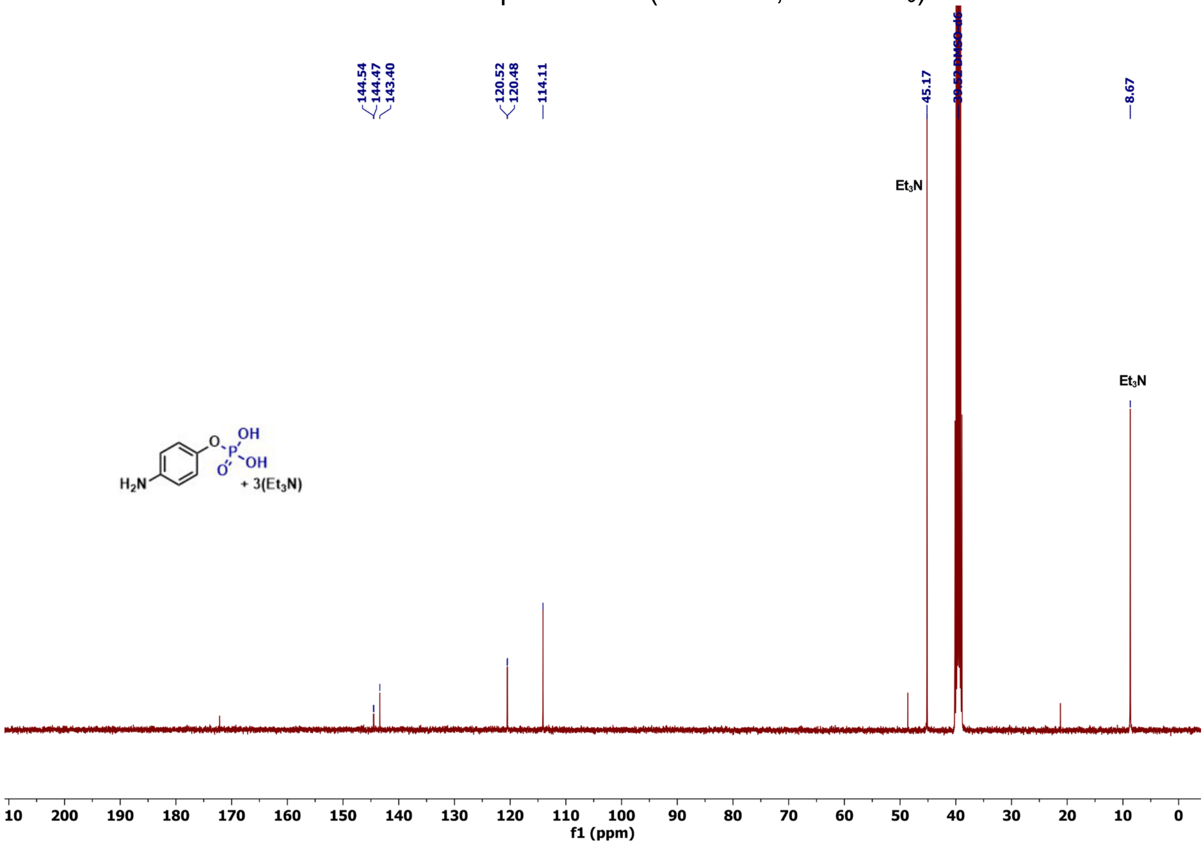

^{31}P NMR of Compound **21b** (162 MHz, DMSO-d_6)

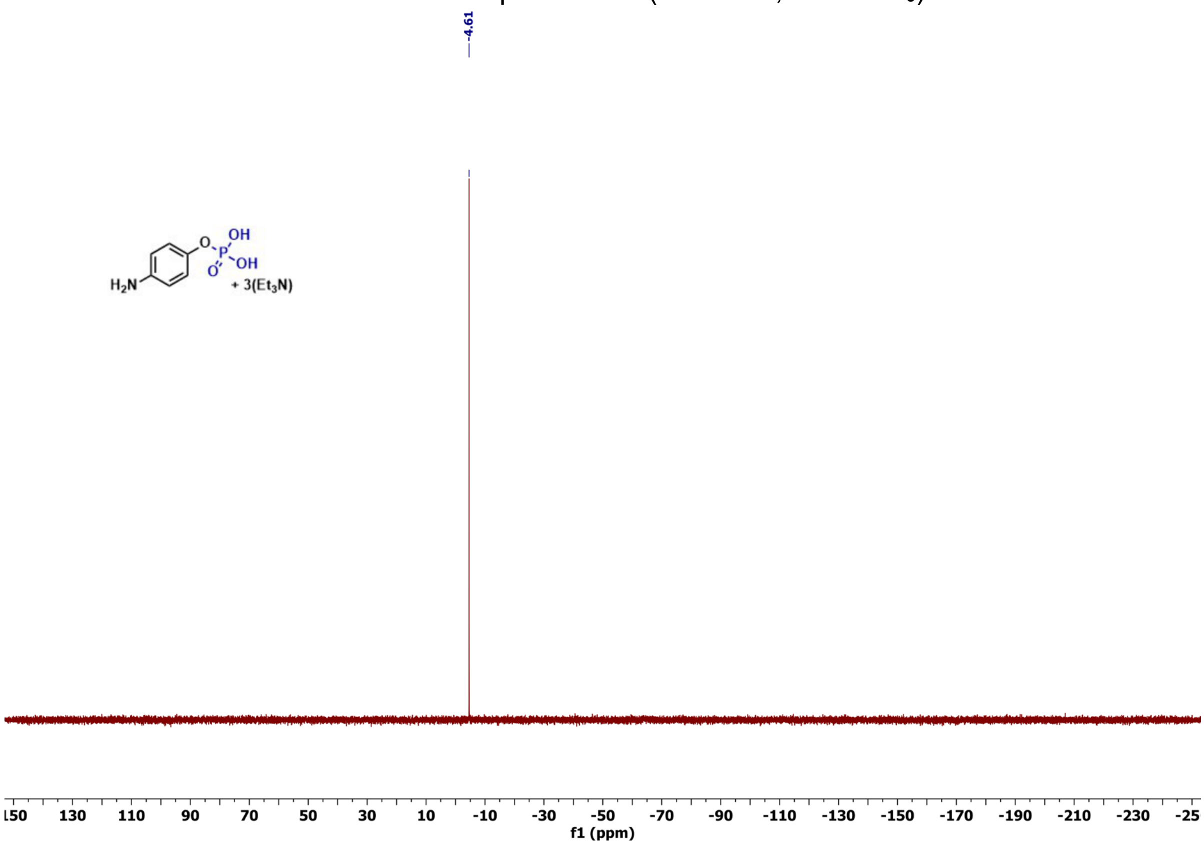

^{1}H NMR of Compound **22a** (800 MHz, $CDCl_3$)

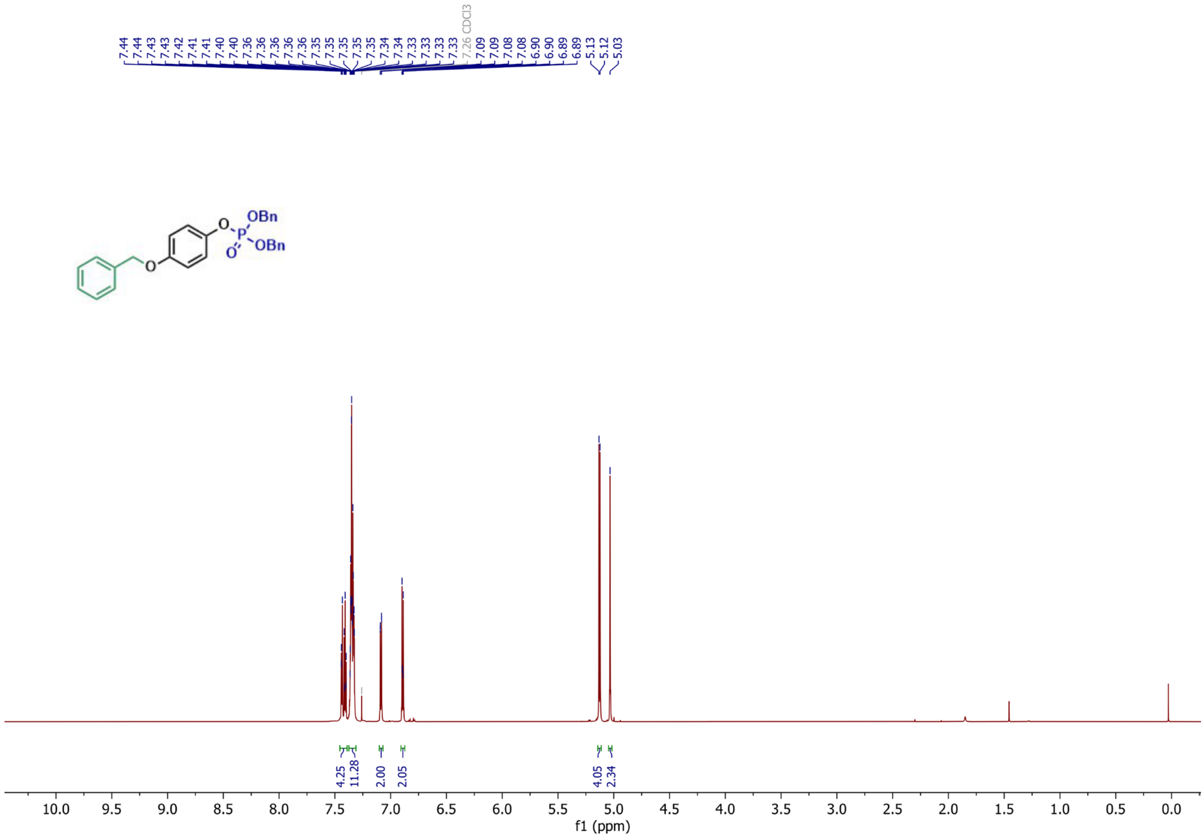

^{13}C NMR of Compound **22a** (125 MHz, $CDCl_3$)

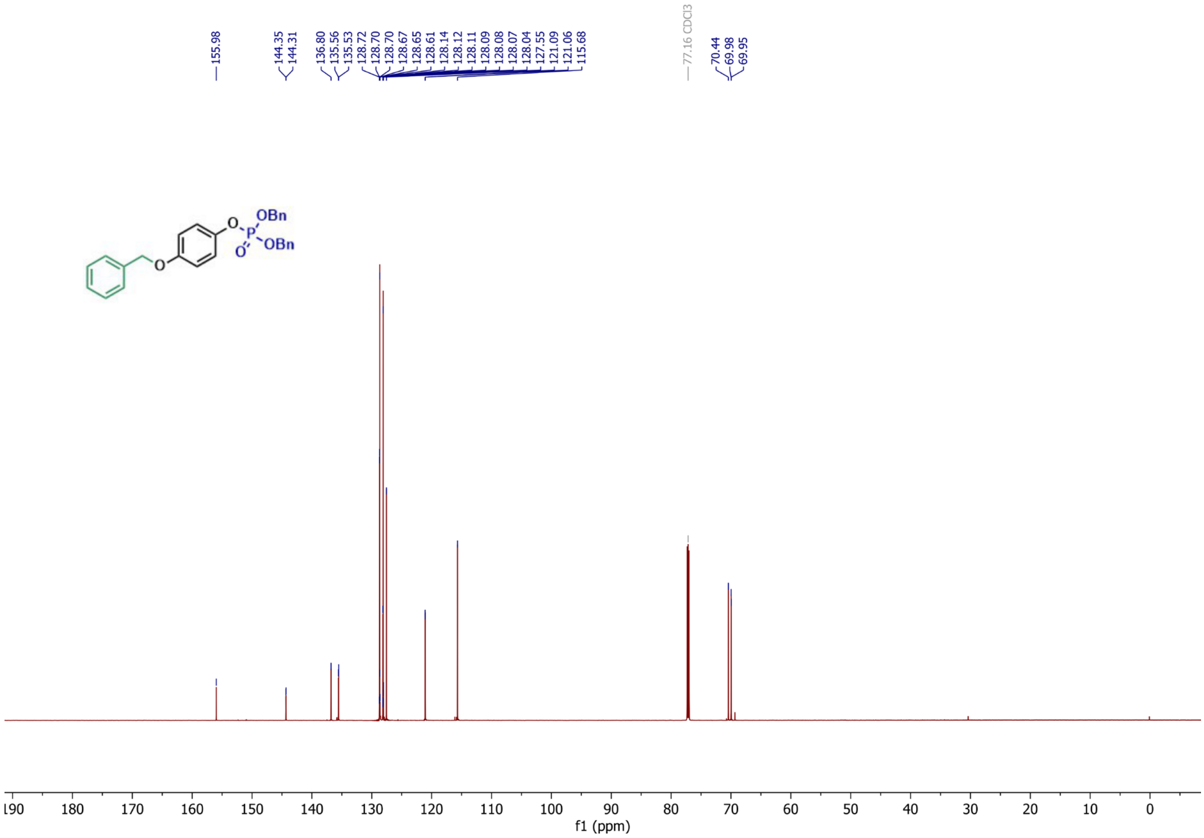

^{31}P NMR of Compound **22a** (162 MHz, $CDCl_3$)

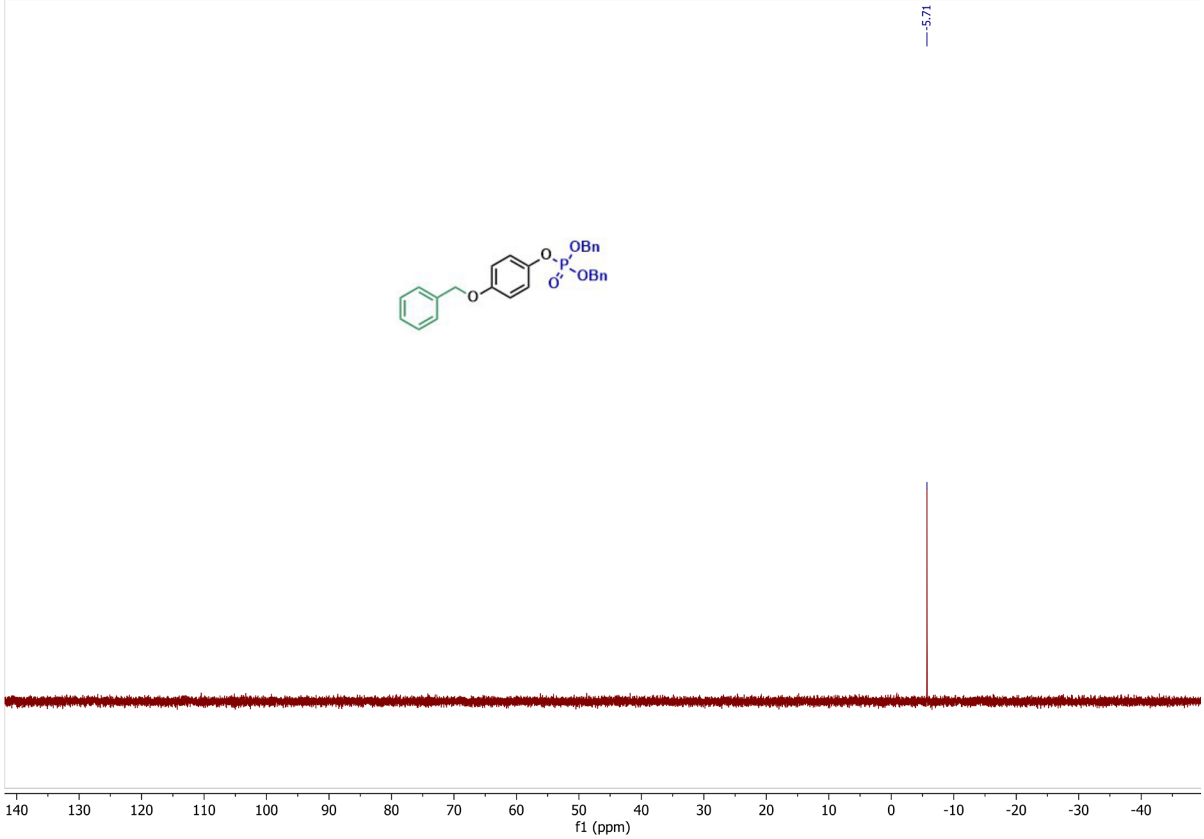

^{1}H NMR of Compound **22b** (400 MHz, DMSO-d_6)

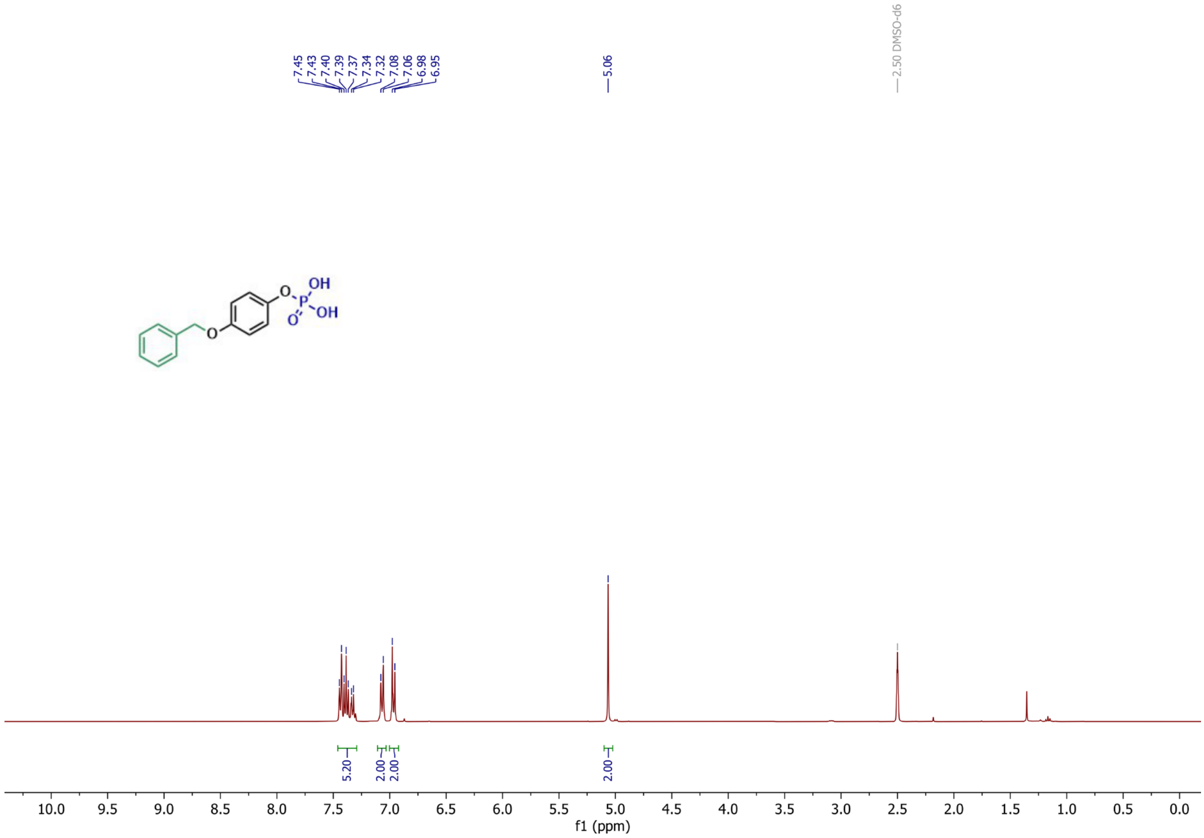

^{13}C NMR of Compound **22b** (125 MHz, DMSO-d_6)

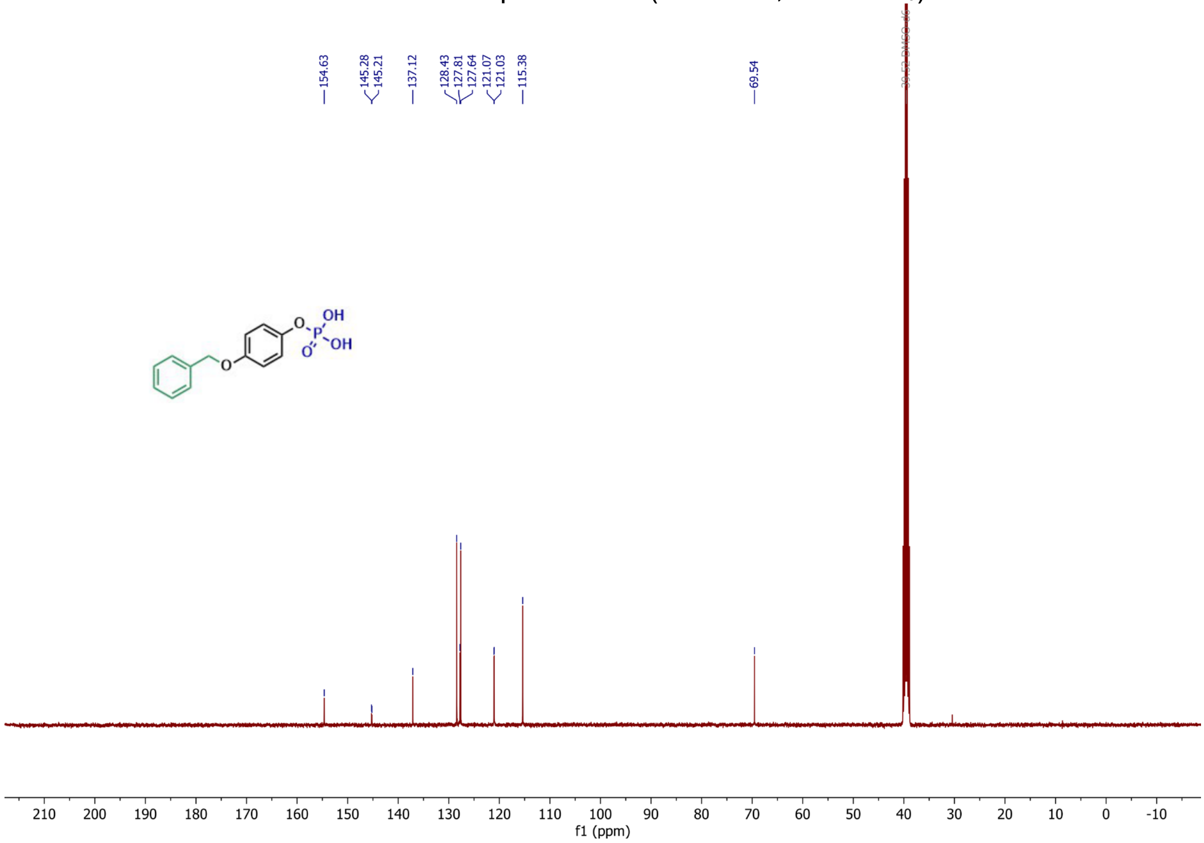

^{31}P NMR of Compound **22b** (162 MHz, DMSO-d_6)

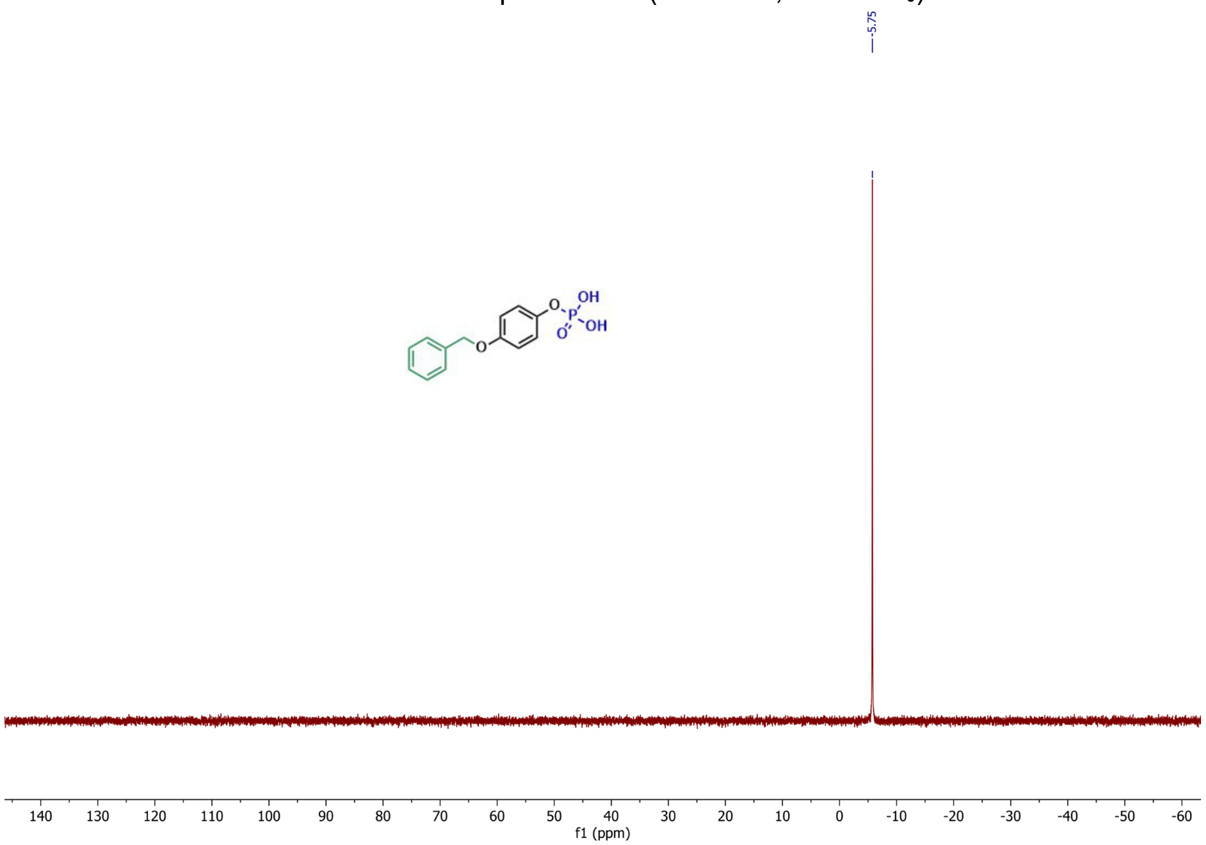

^{1}H NMR of Compound **22c** (400 MHz, DMSO-d_6)

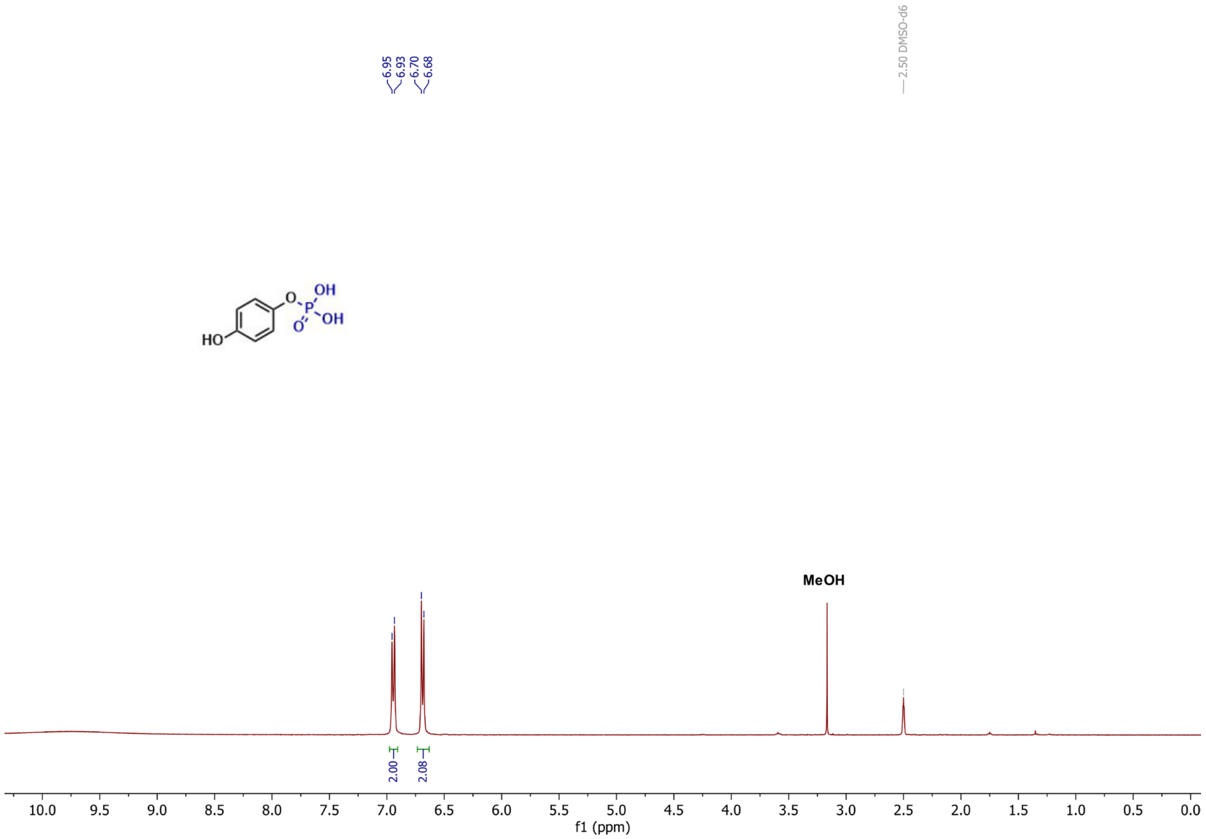

^{13}C NMR of Compound **22c** (101 MHz, DMSO-d_6)

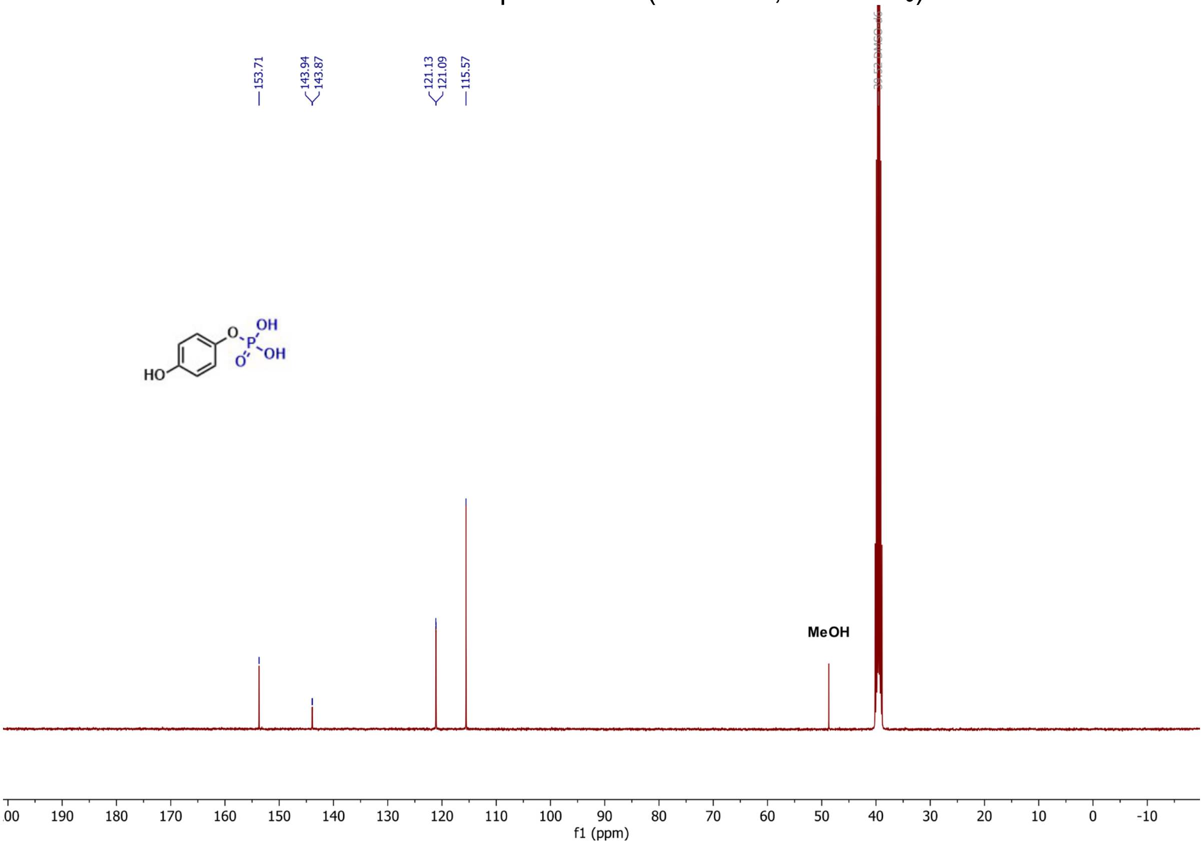

^{31}P NMR of Compound **22c** (162 MHz, DMSO-d_6)

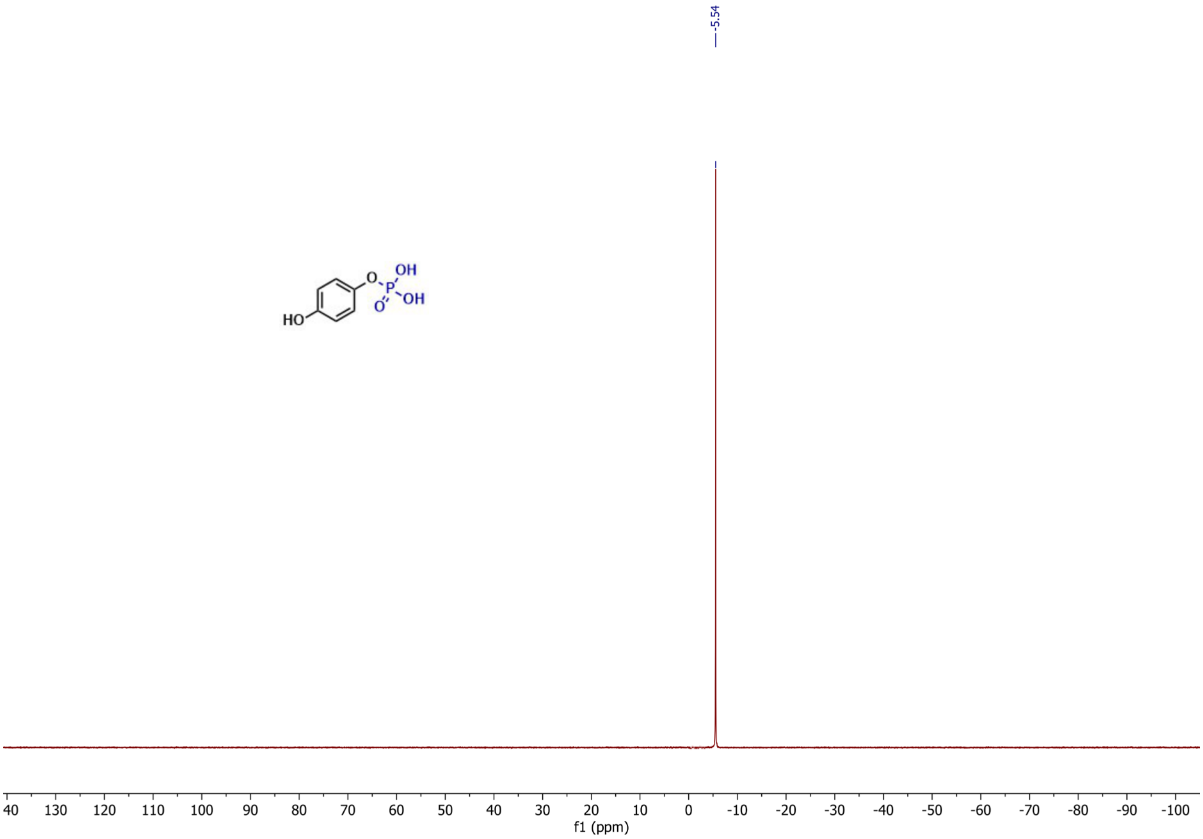

^{1}H NMR of Compound **23a** (400 MHz, $CDCl_3$)

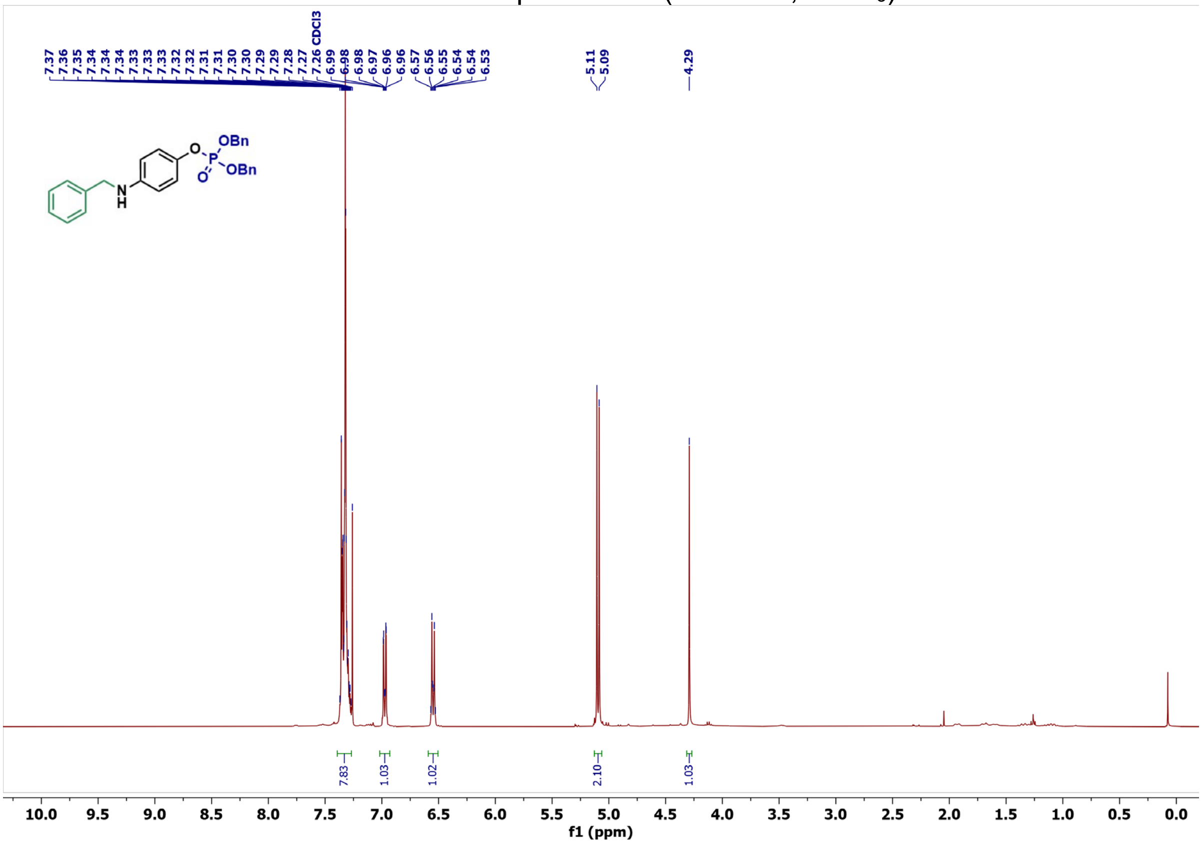

^{13}C NMR of Compound **23a** (125 MHz, $CDCl_3$)

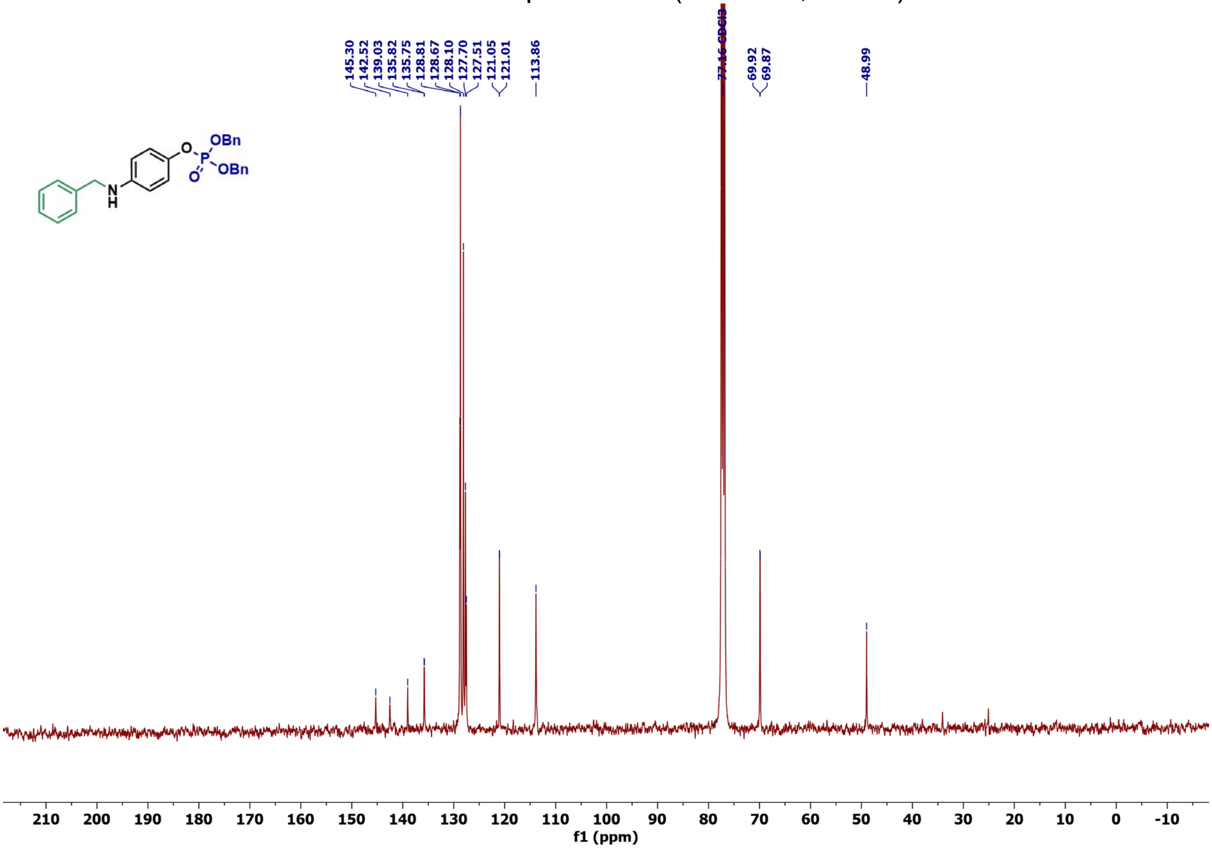

^{31}P NMR of Compound **23a** (162 MHz, $CDCl_3$)

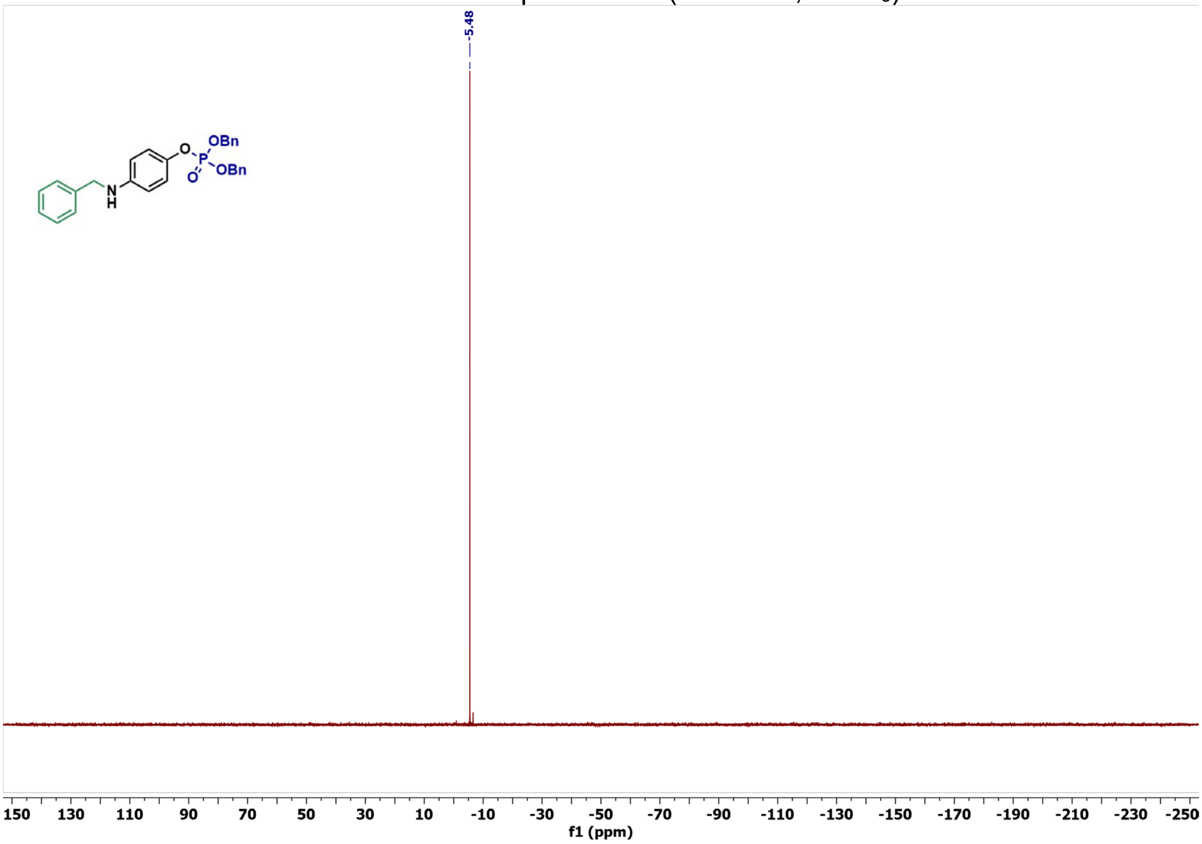

^{1}H NMR of Compound **23b** (400 MHz, DMSO-d_6)

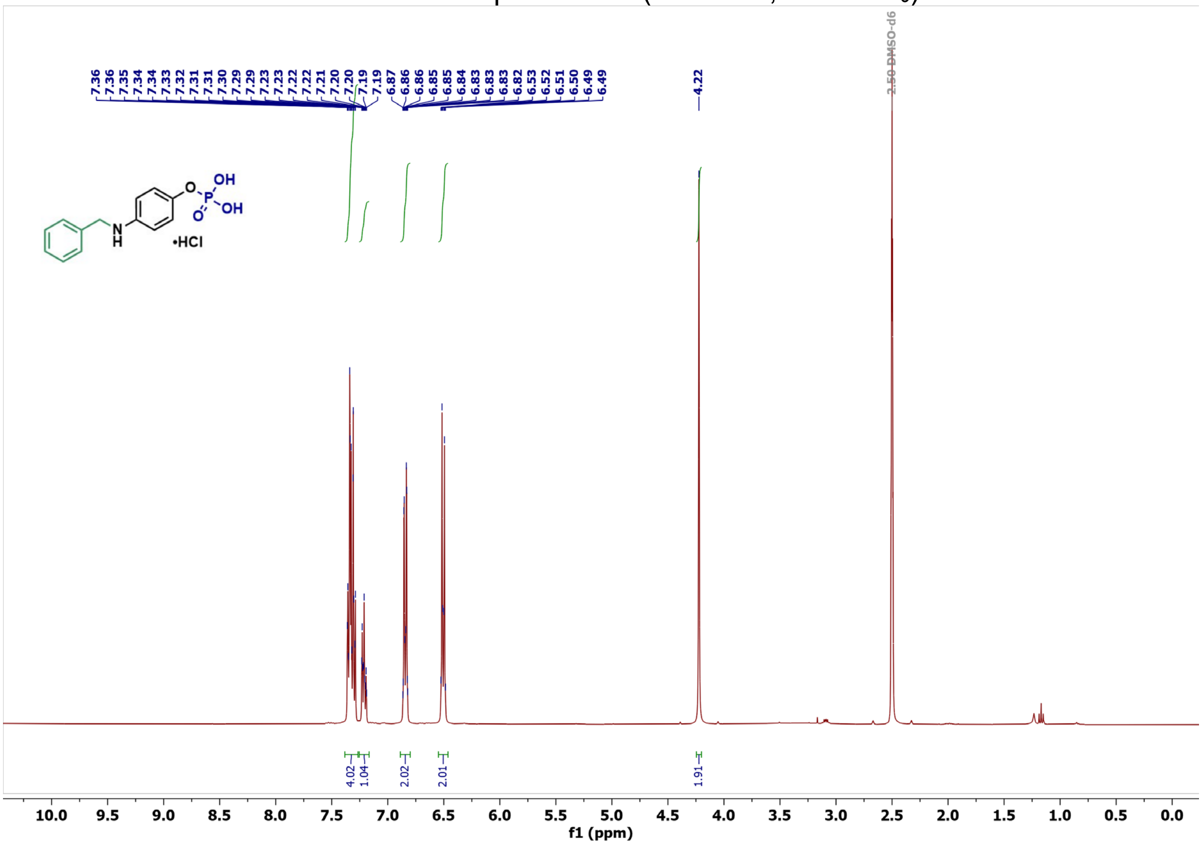

^{13}C NMR of Compound **23b** (125 MHz, DMSO-d_6)

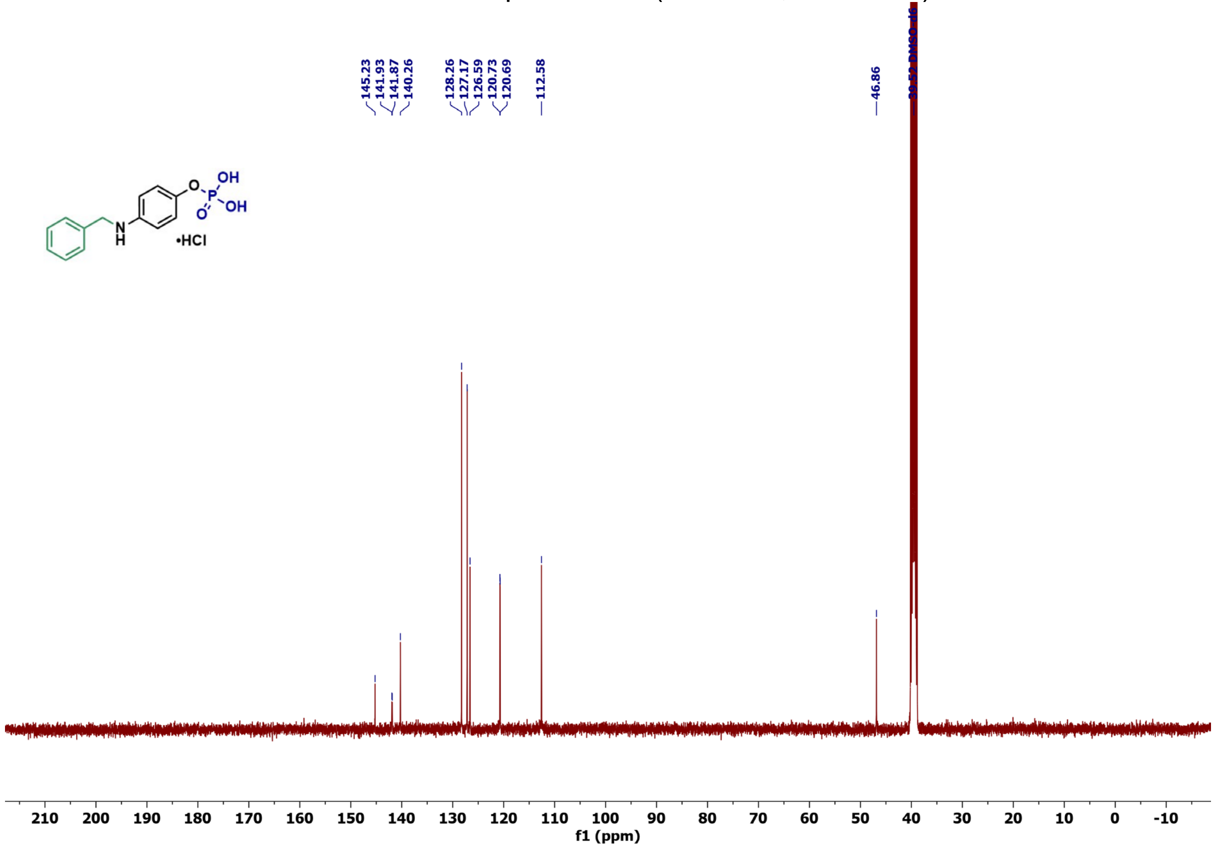

^{31}P NMR of Compound **23b** (162 MHz, DMSO-d_6)

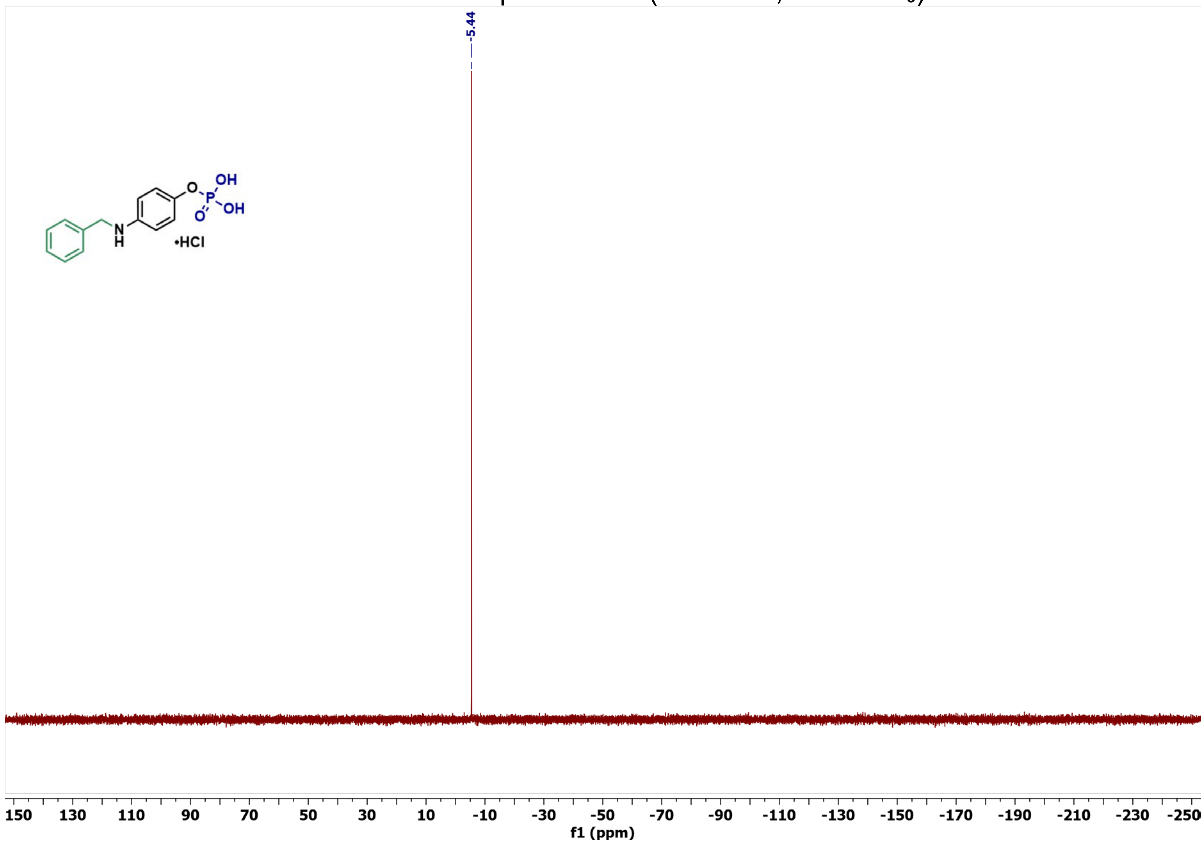

^{1}H NMR of Compound **24a** (400 MHz, $CDCl_3$)

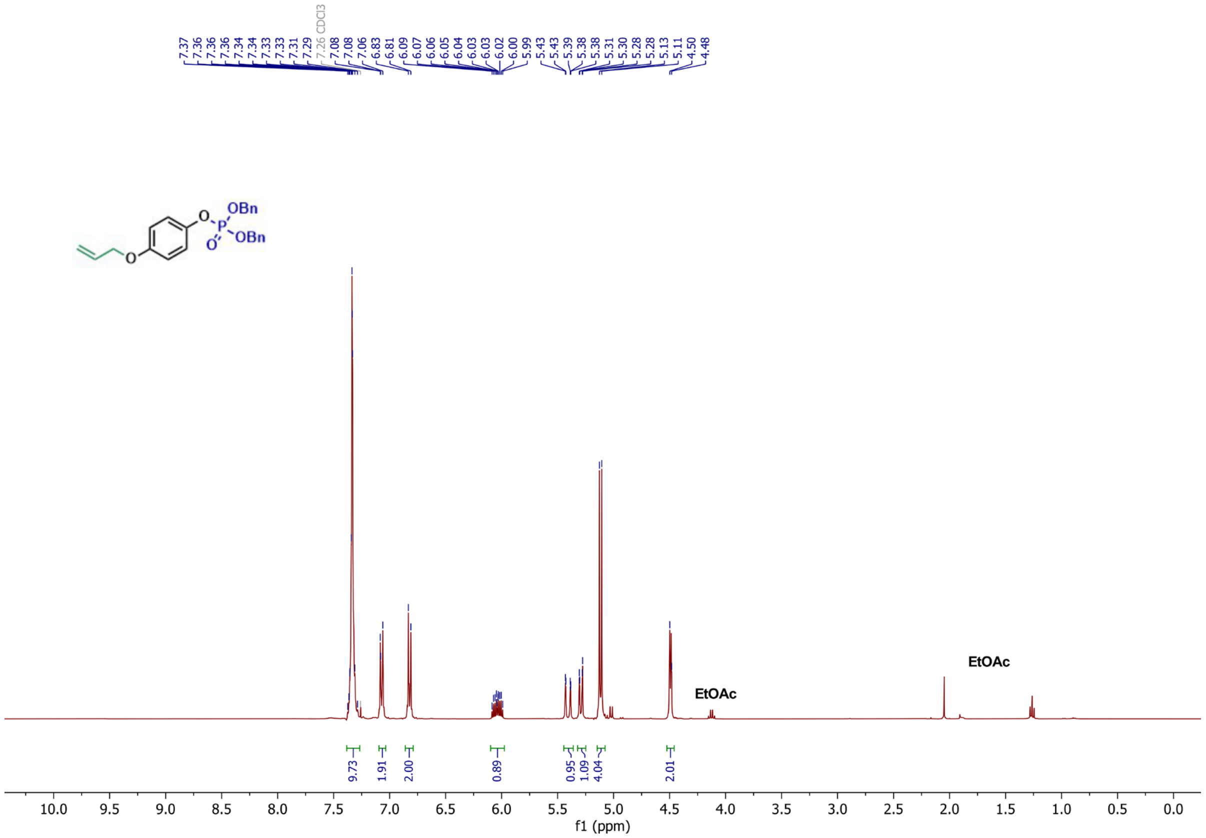

^{13}C NMR of Compound **24a** (201 MHz, $CDCl_3$)

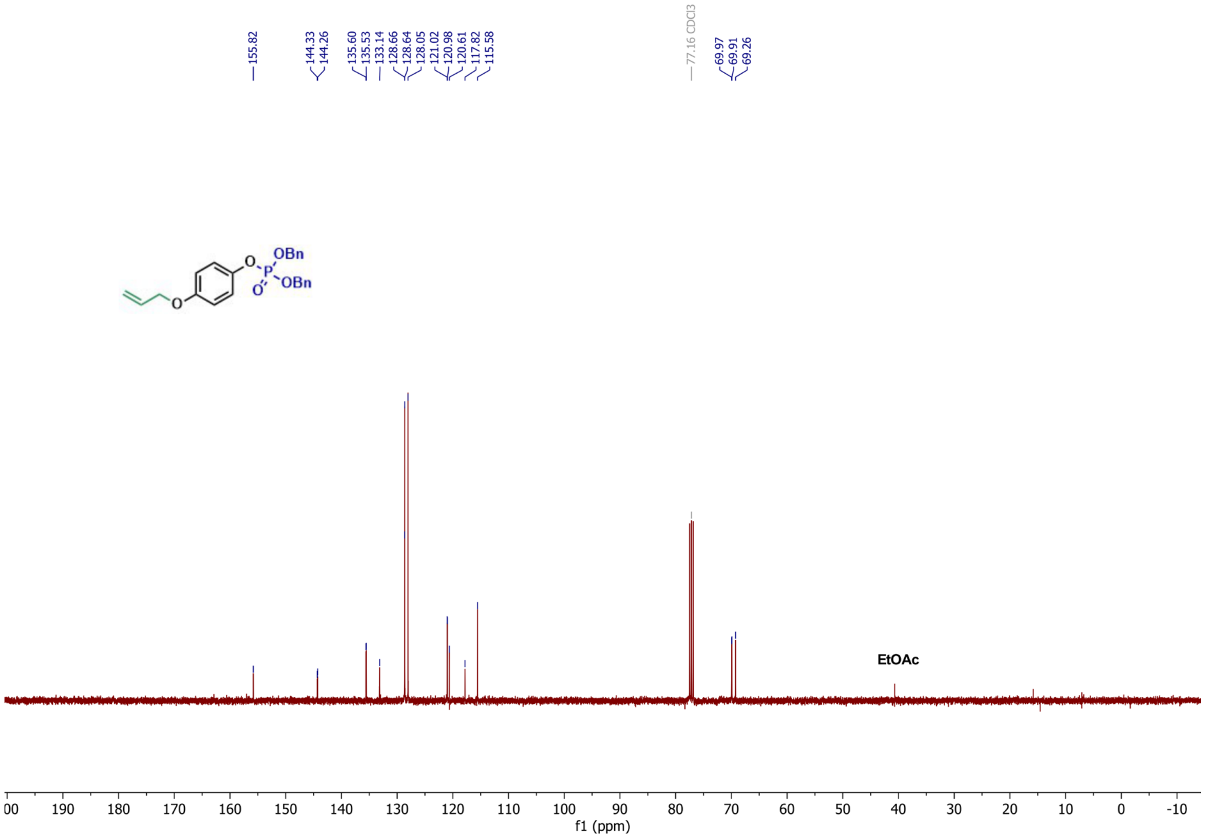

^{31}P NMR of Compound **24a** (162 MHz, $CDCl_3$)

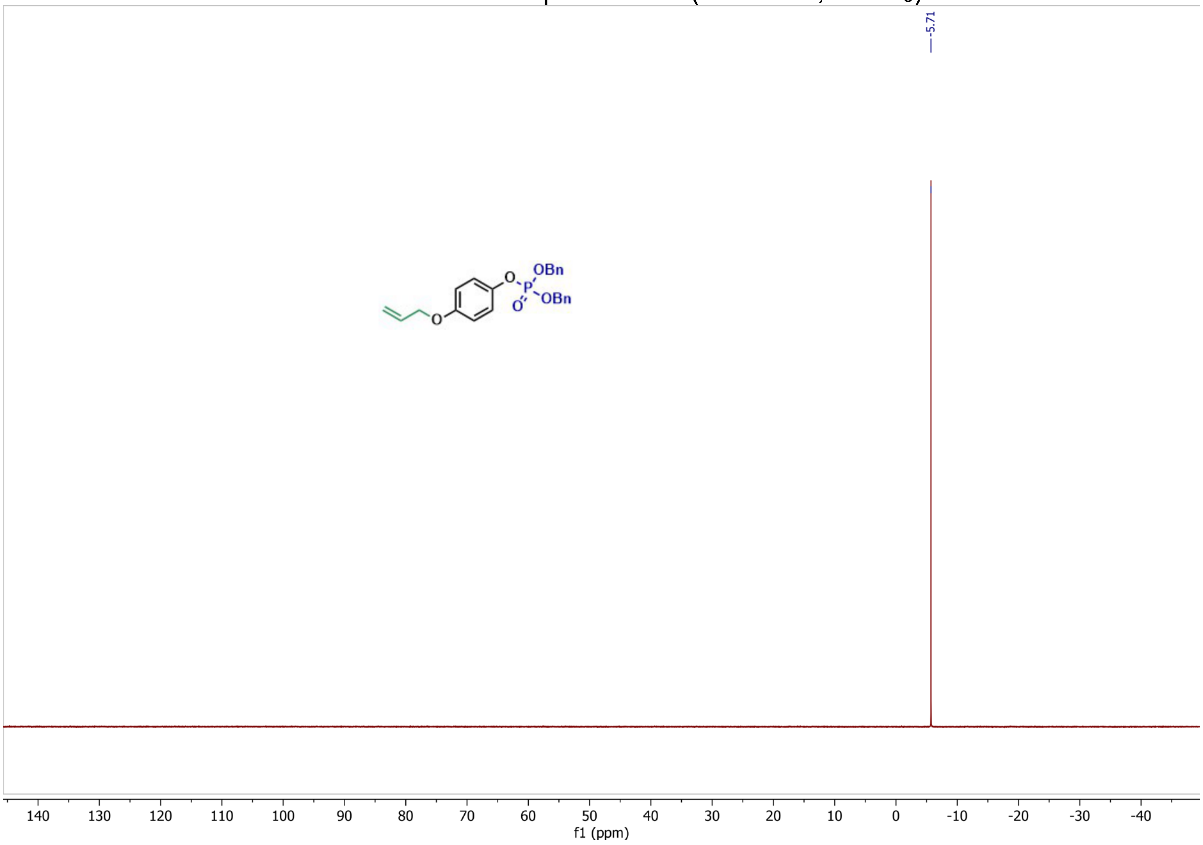

^{1}H NMR of Compound **24b** (400 MHz, DMSO-d_6)

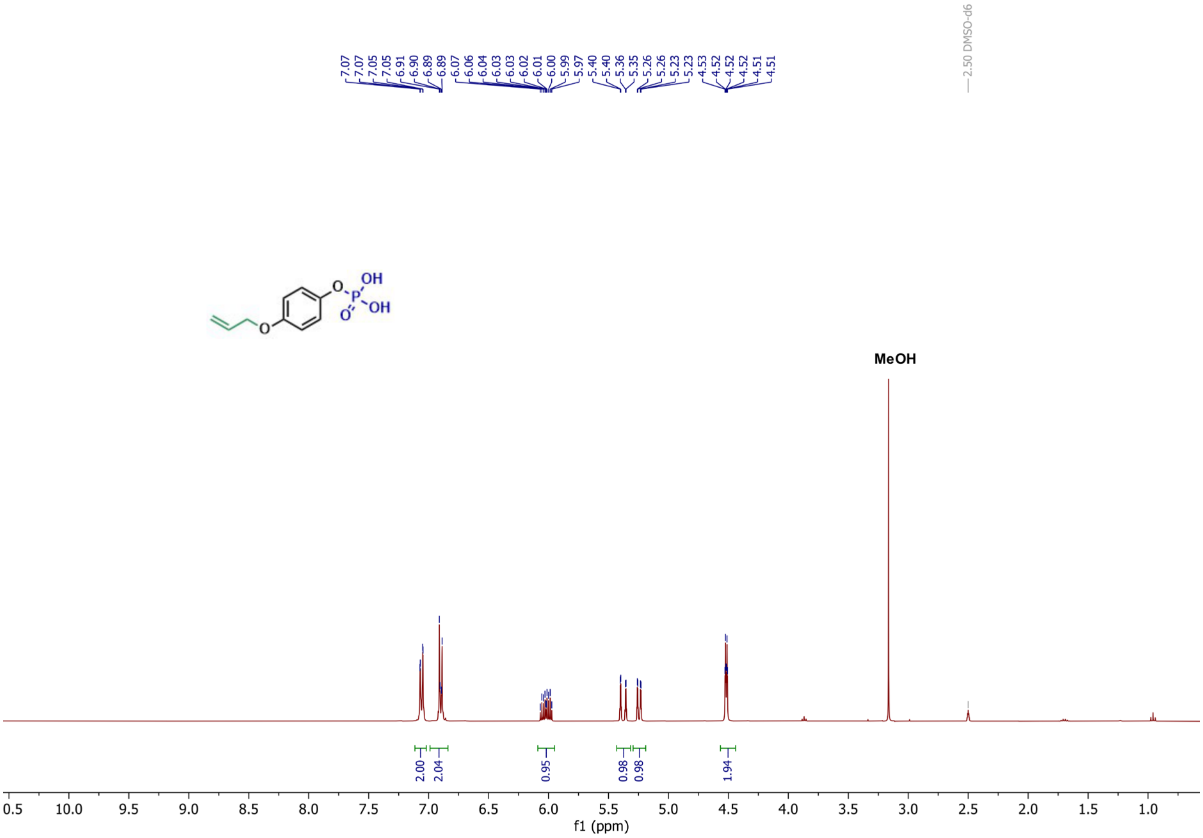

^{13}C NMR of Compound **24b** (125 MHz, DMSO-d_6)

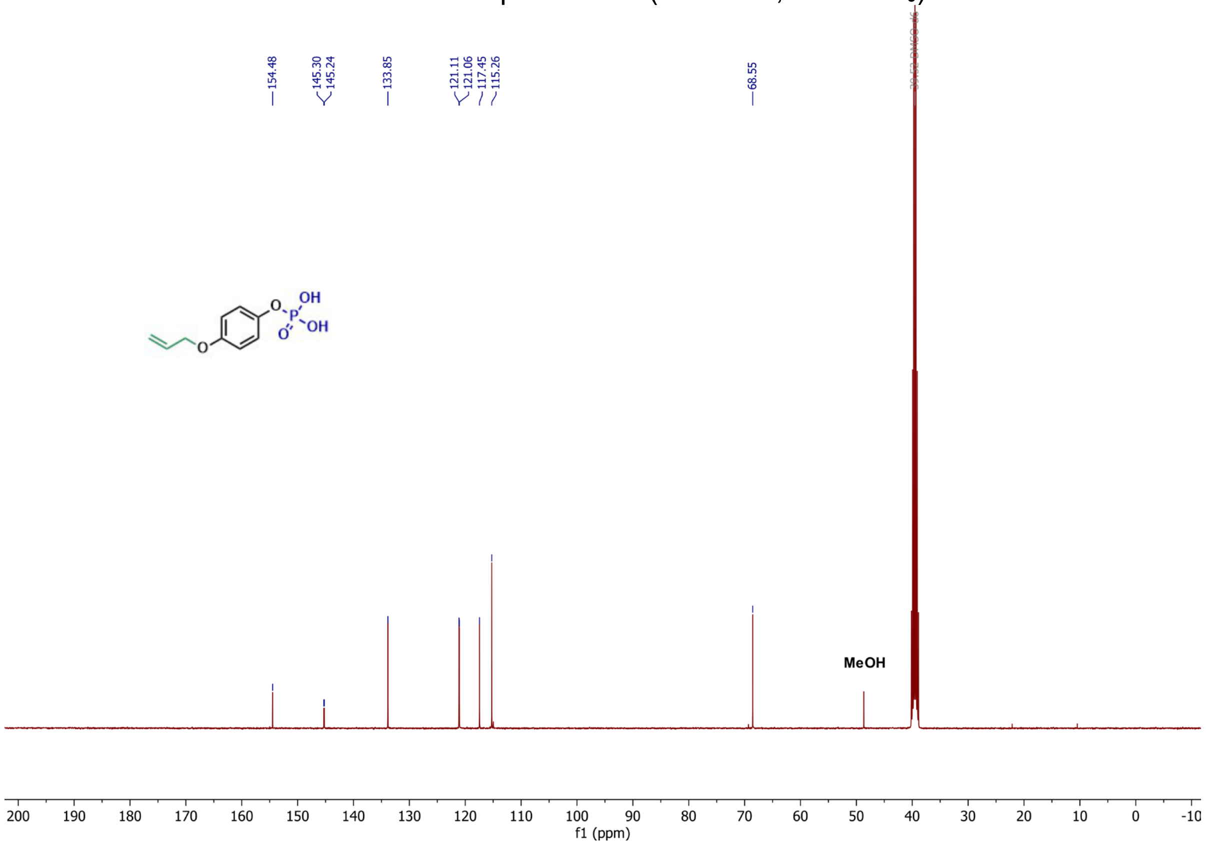

^{31}P NMR of Compound **24b** (162 MHz, DMSO-d_6)

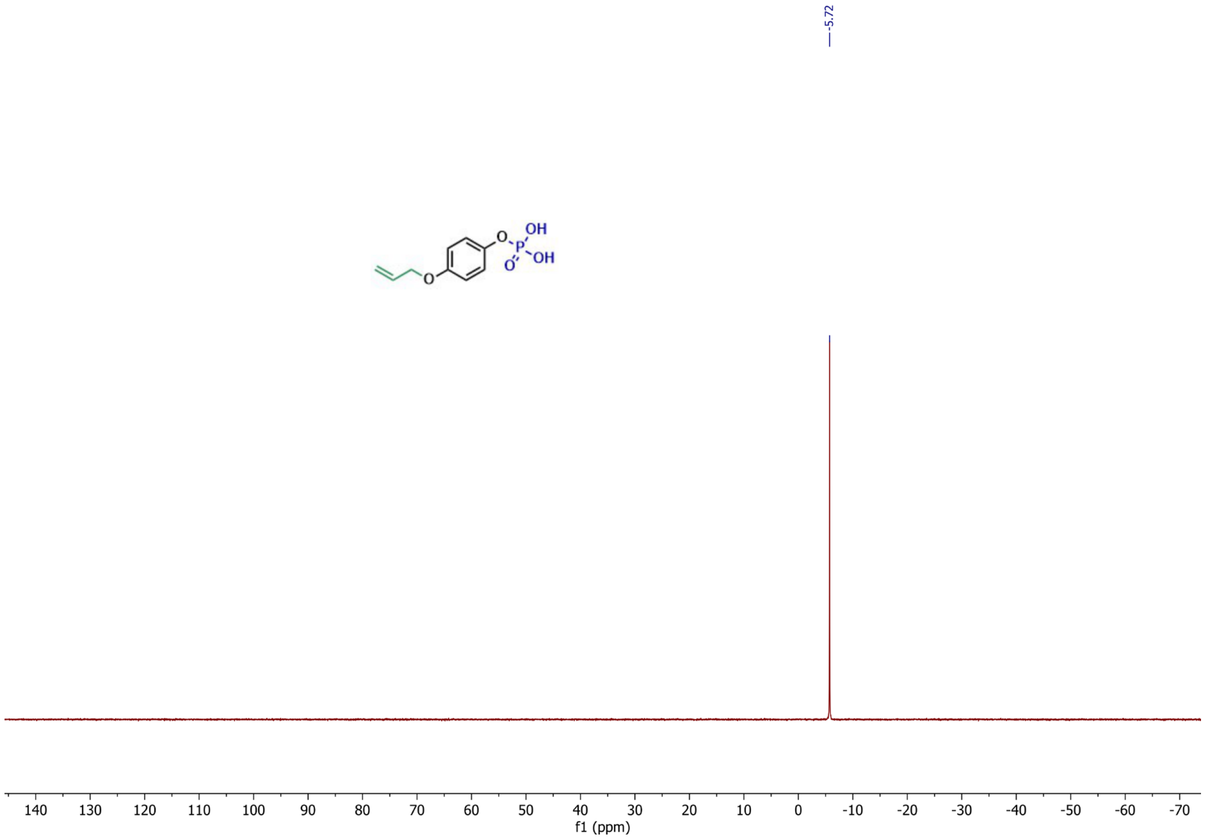

^{1}H NMR of Compound **25a** (600 MHz, $CDCl_3$)

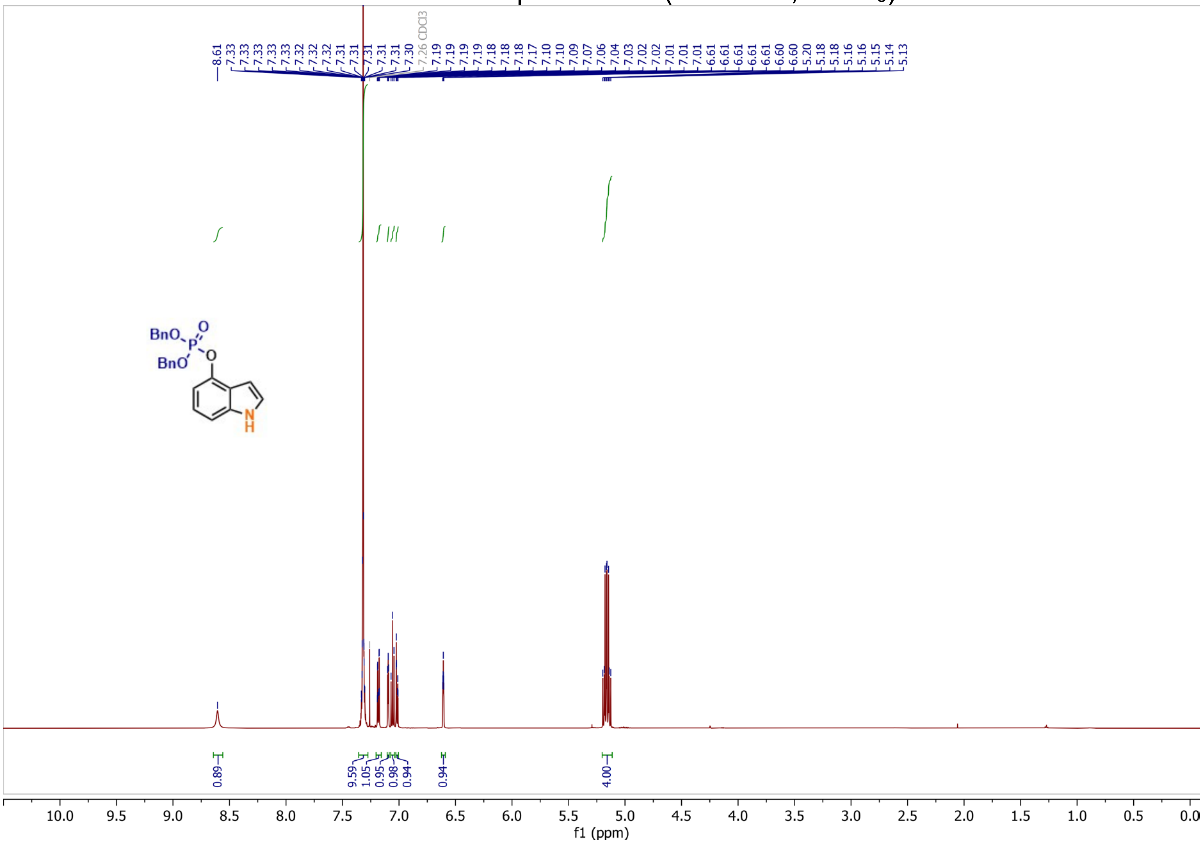

^{13}C NMR of Compound **25a** (151 MHz, $CDCl_3$)

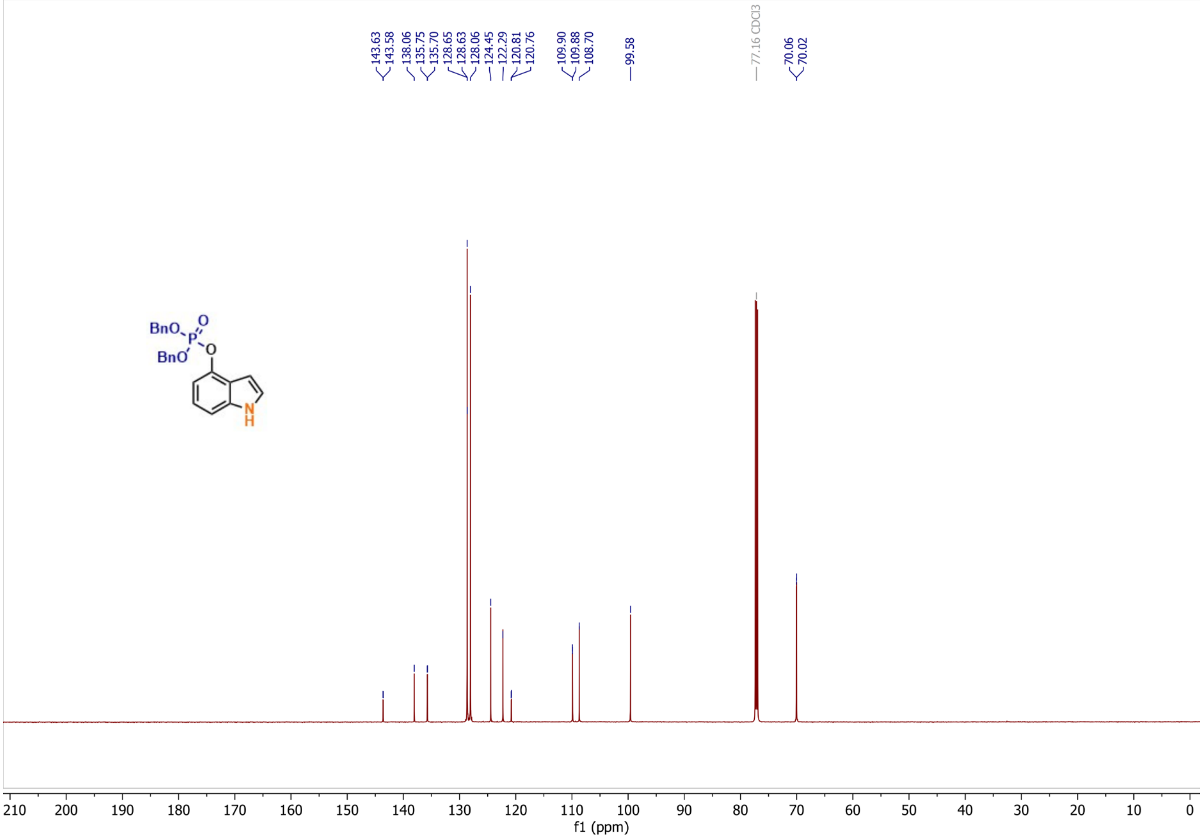

^{31}P NMR of Compound **25a** (243 MHz, $CDCl_3$)

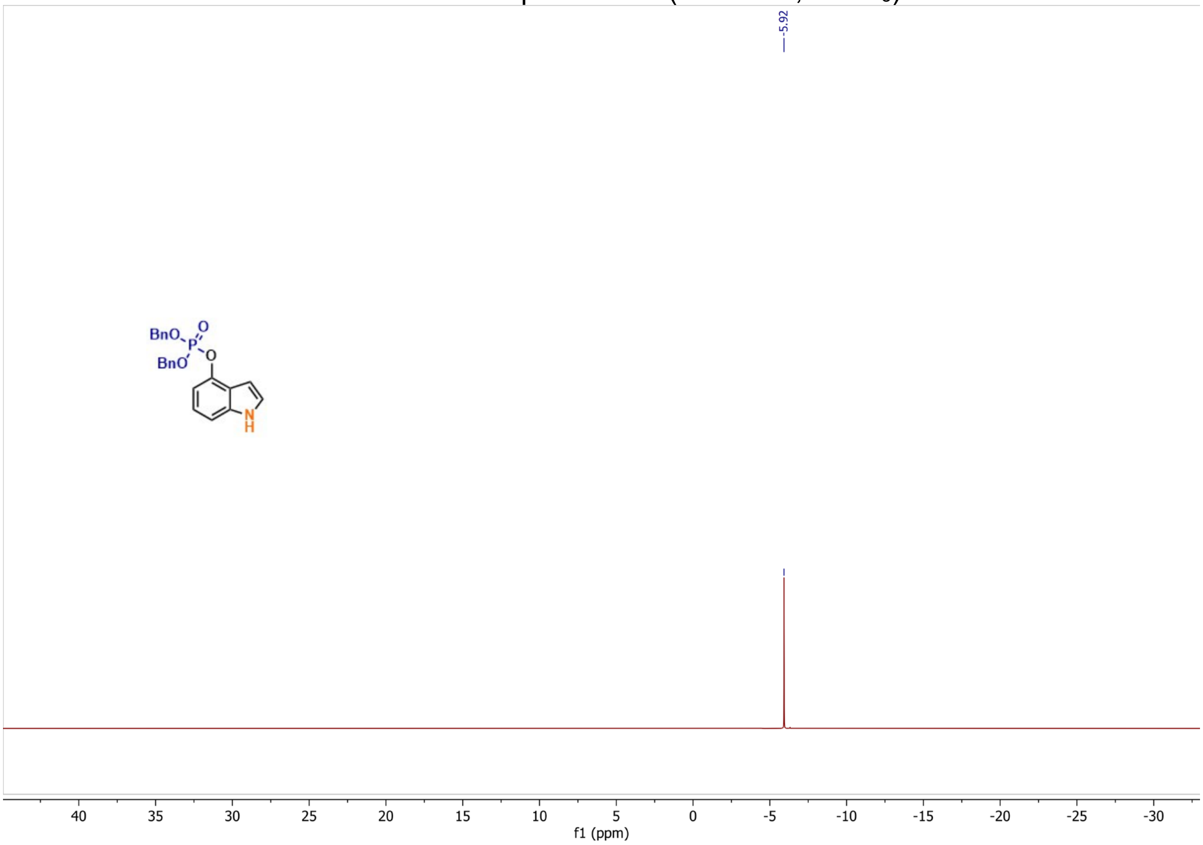

^{1}H NMR of Compound **26a** (600 MHz, $CDCl_3$)

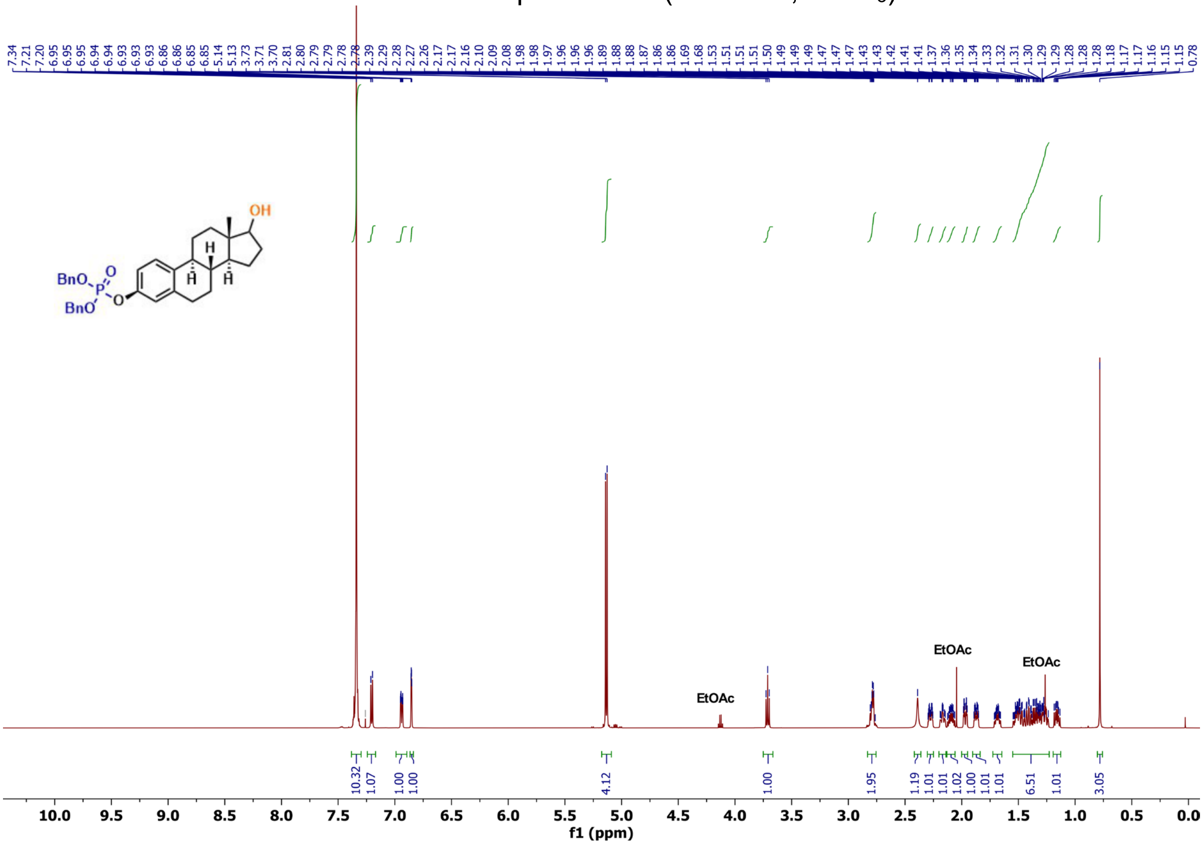

^{13}C NMR of Compound **26a** (151 MHz, $CDCl_3$)

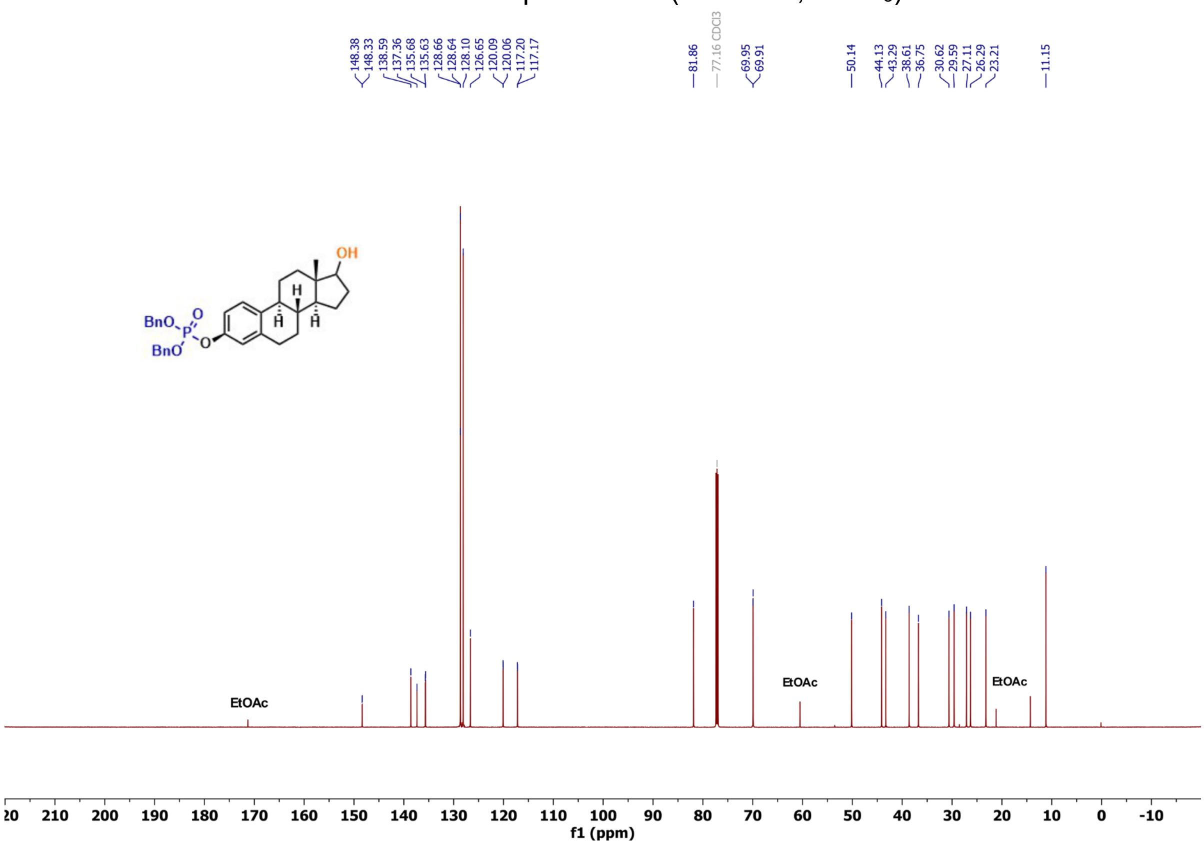

^{31}P NMR of Compound **26a** (243 MHz, $CDCl_3$)

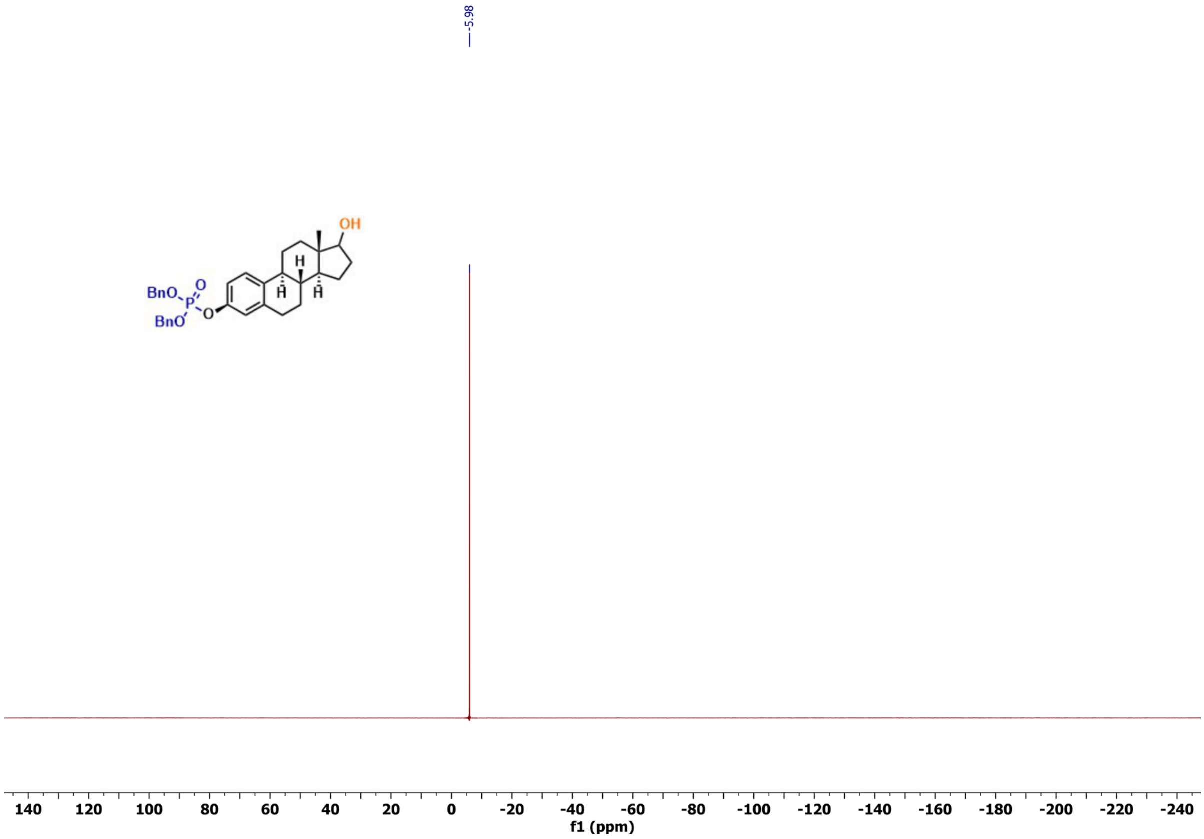

^{1}H NMR of Compound **26b** (600 MHz, DMSO-d_6)

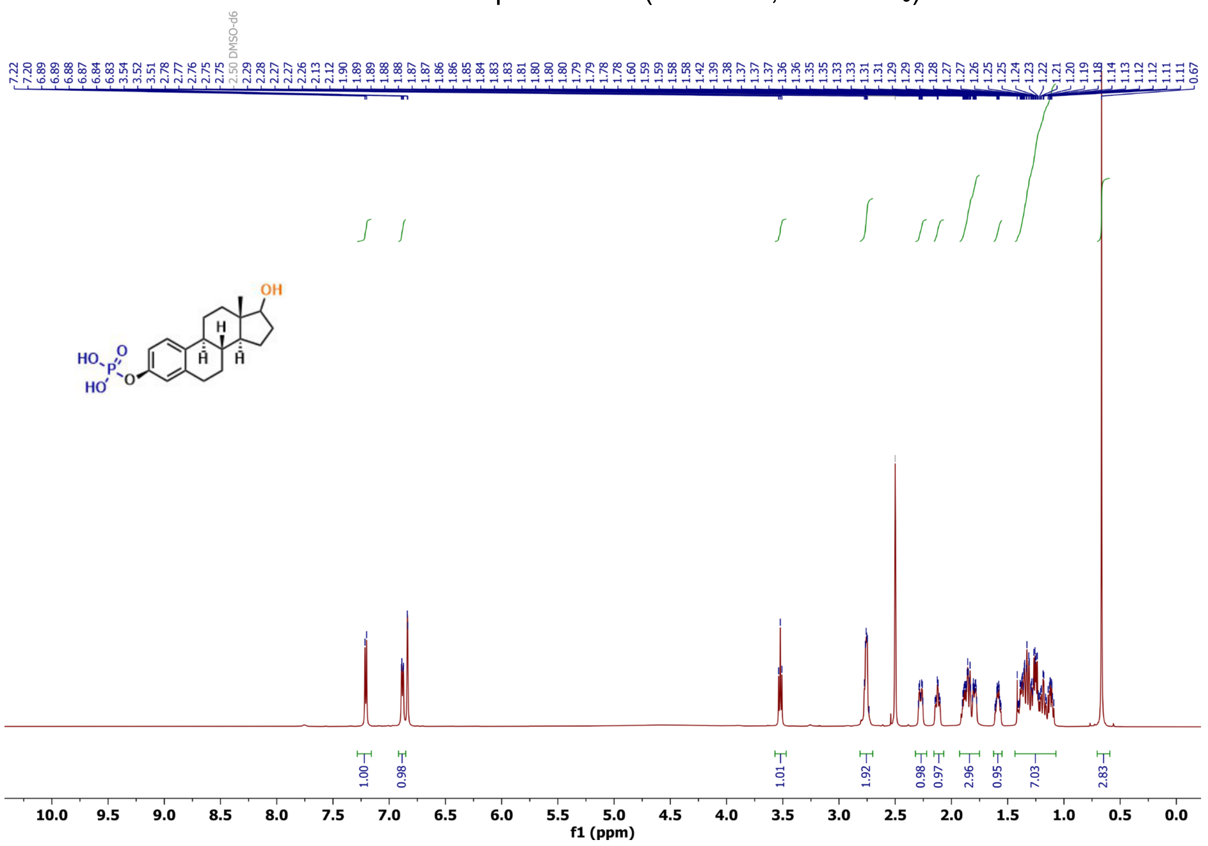

^{13}C NMR of Compound **26b** (151 MHz, DMSO-d_6)

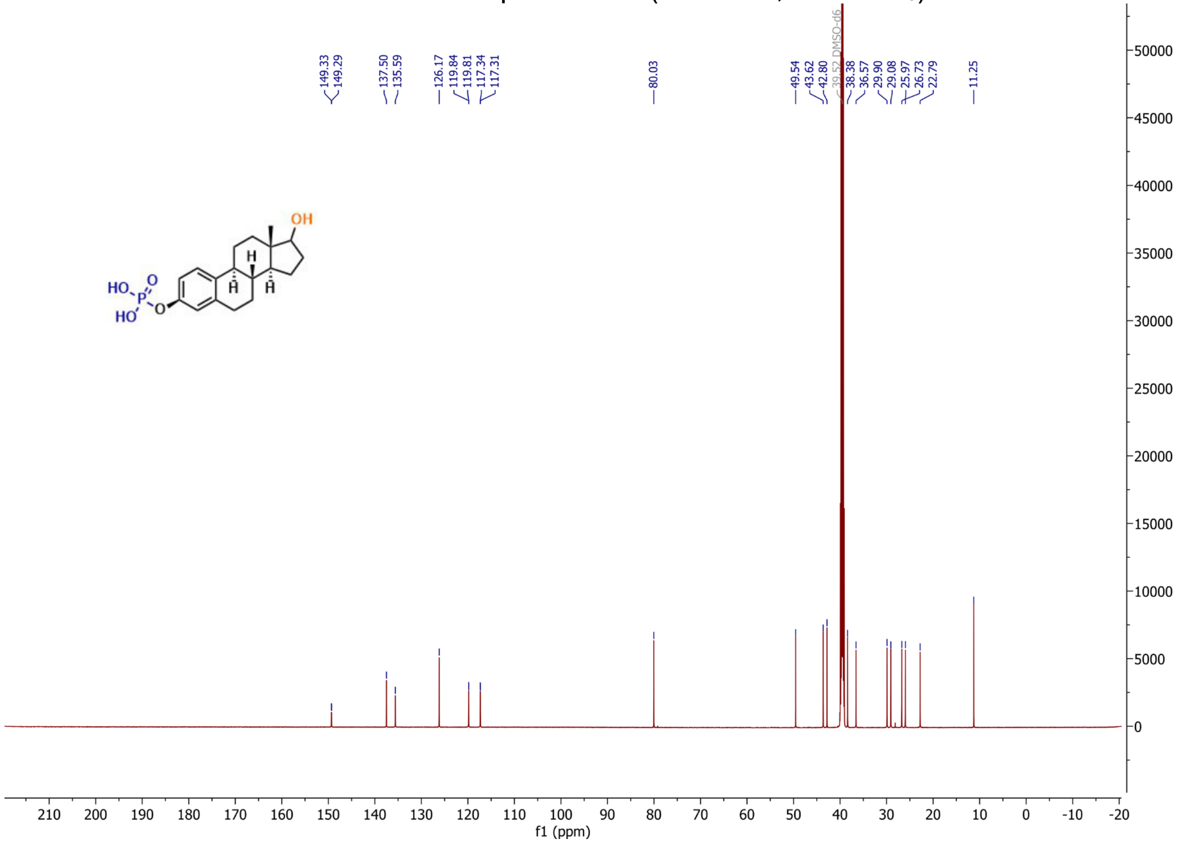

^{31}P NMR of Compound **26b** (243 MHz, $CDCl_3$)

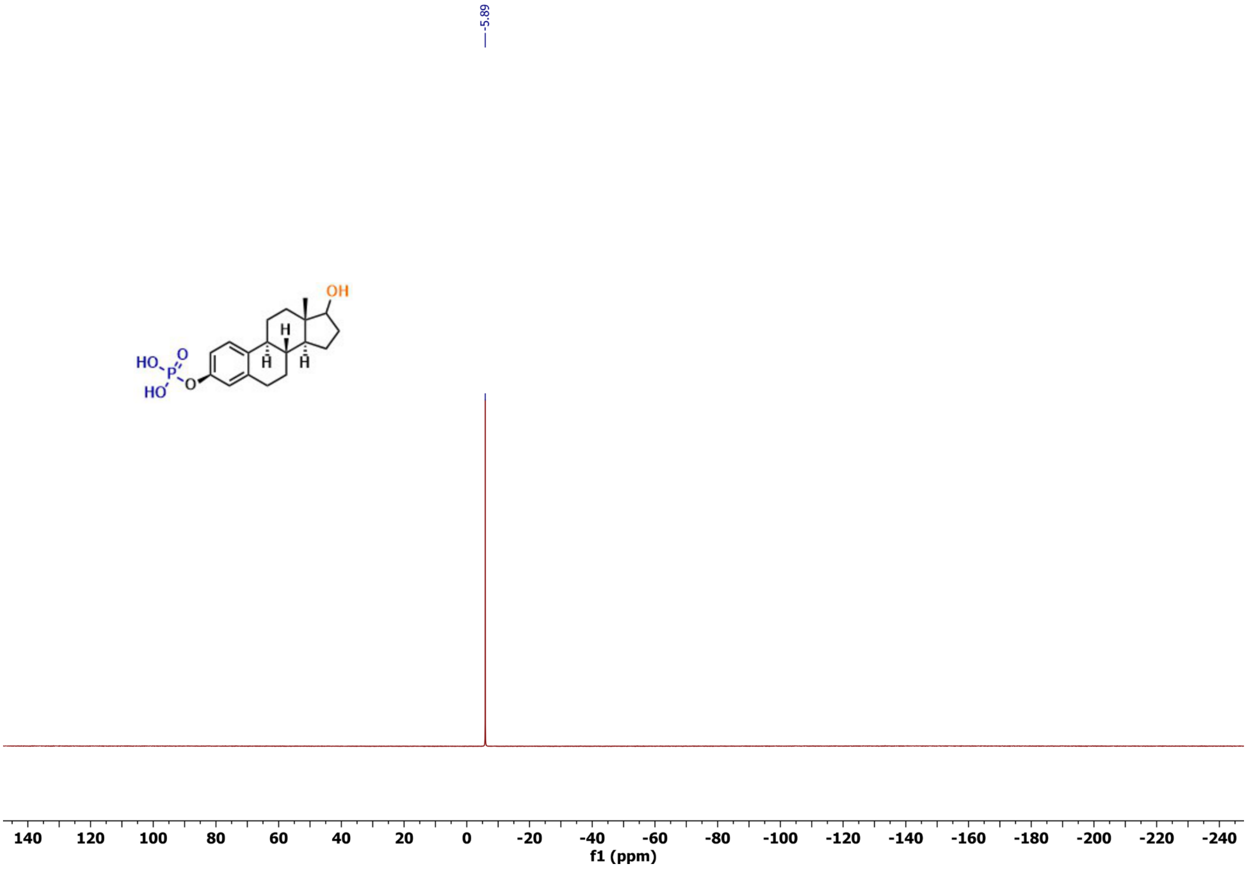

^{1}H NMR of Compound **26c** (600 MHz, DMSO-d_6)

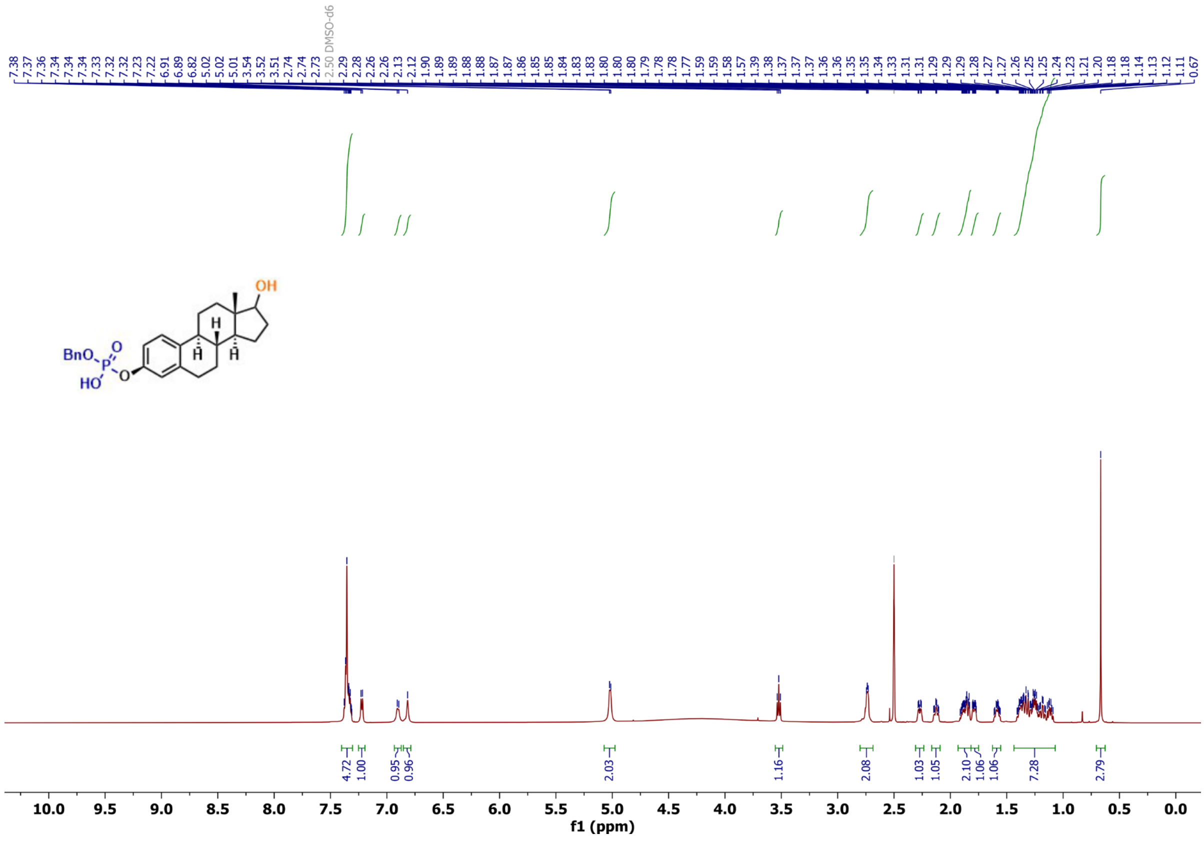

^{13}C NMR of Compound **26c** (151 MHz, DMSO-d_6)

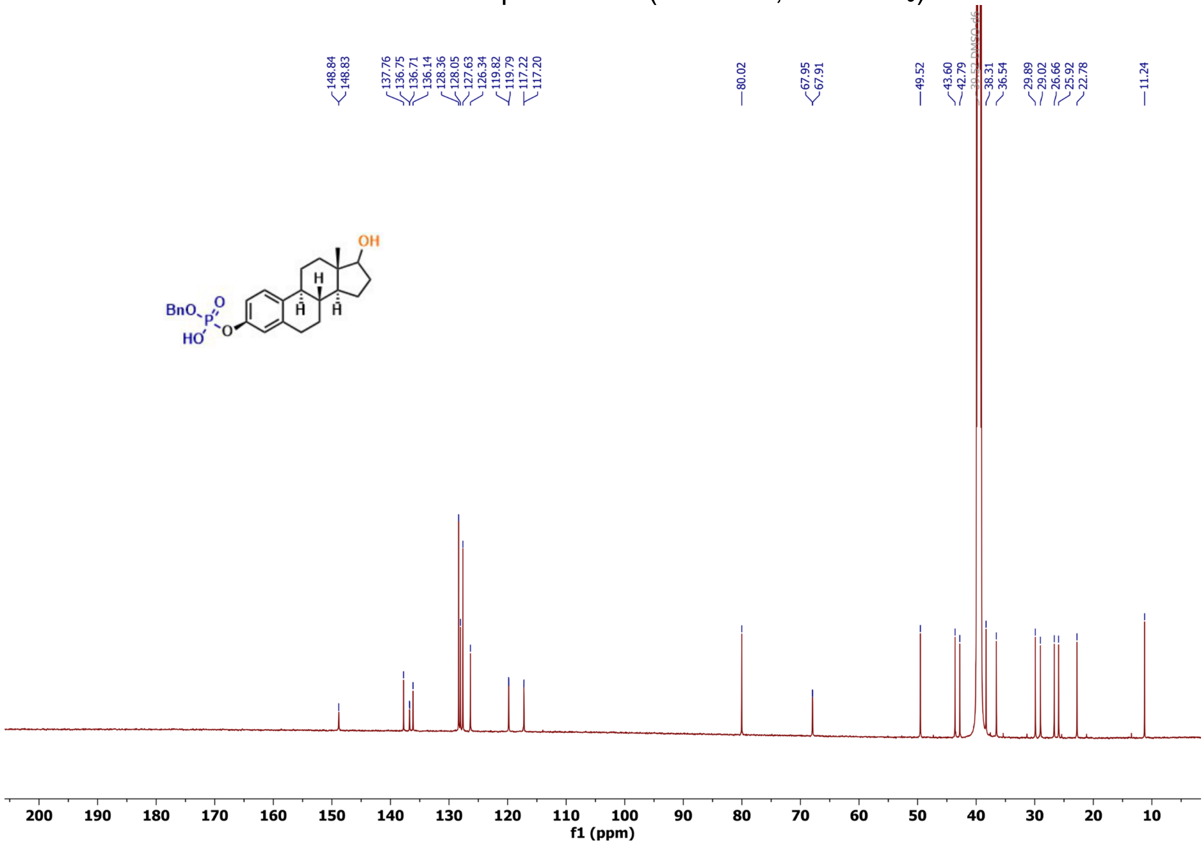

^{31}P NMR of Compound **26c** (243 MHz, DMSO-d_6)

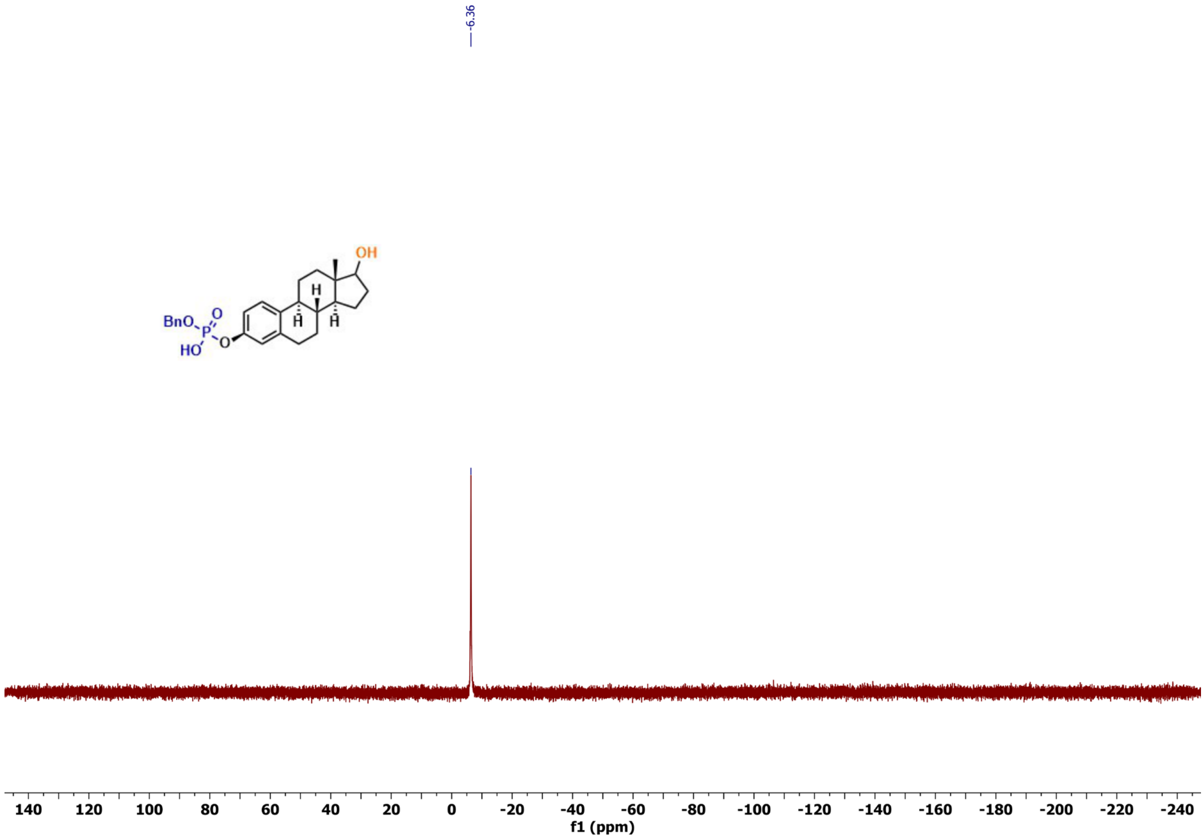

^{1}H NMR of Compound **27a** (600 MHz, $CDCl_3$)

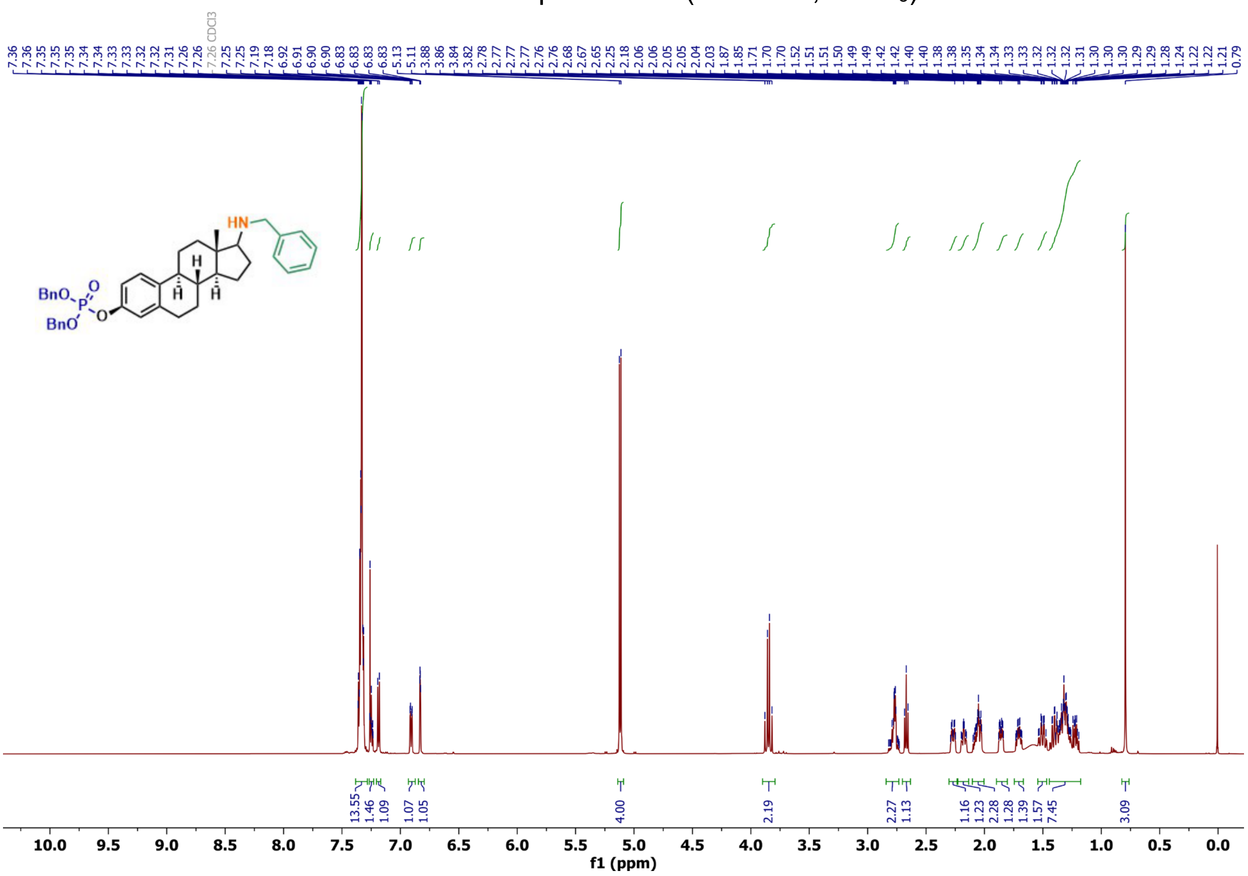

^{13}C NMR of Compound **27a** (151 MHz, $CDCl_3$)

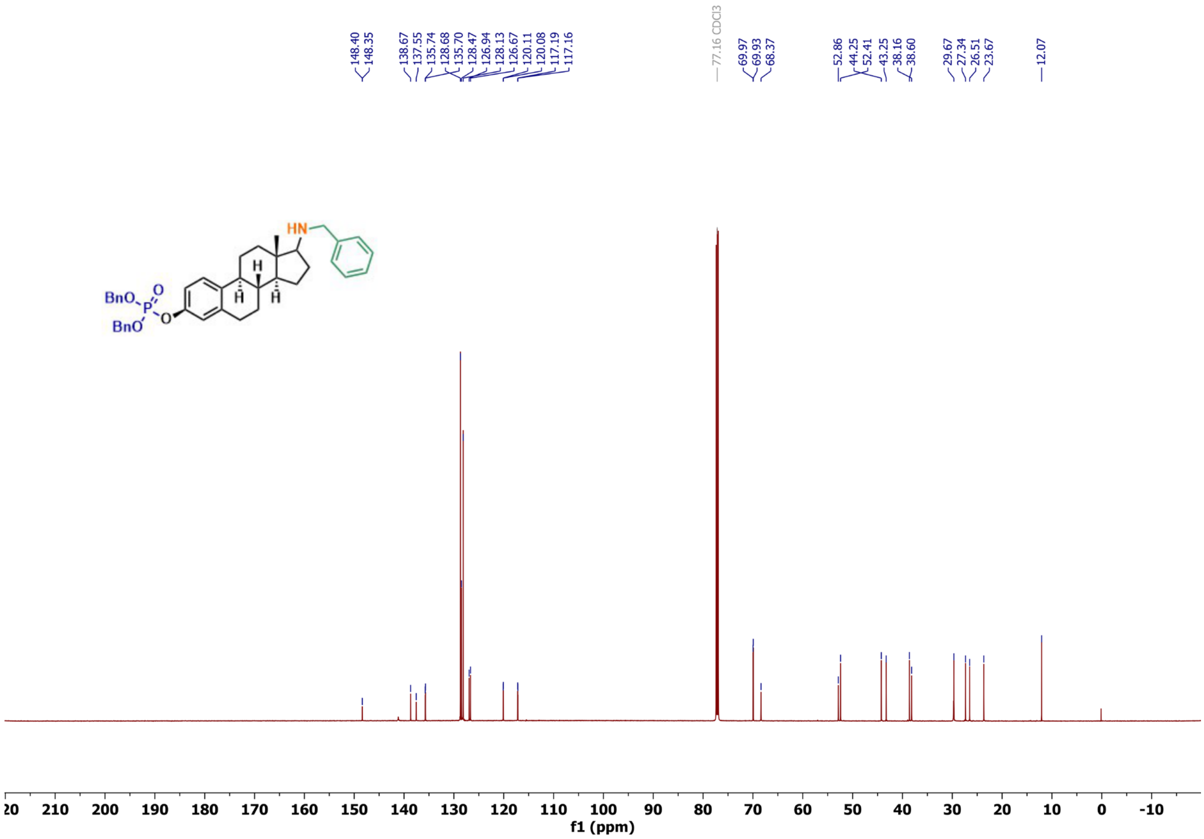

^{31}P NMR of Compound **27a** (243 MHz, $CDCl_3$)

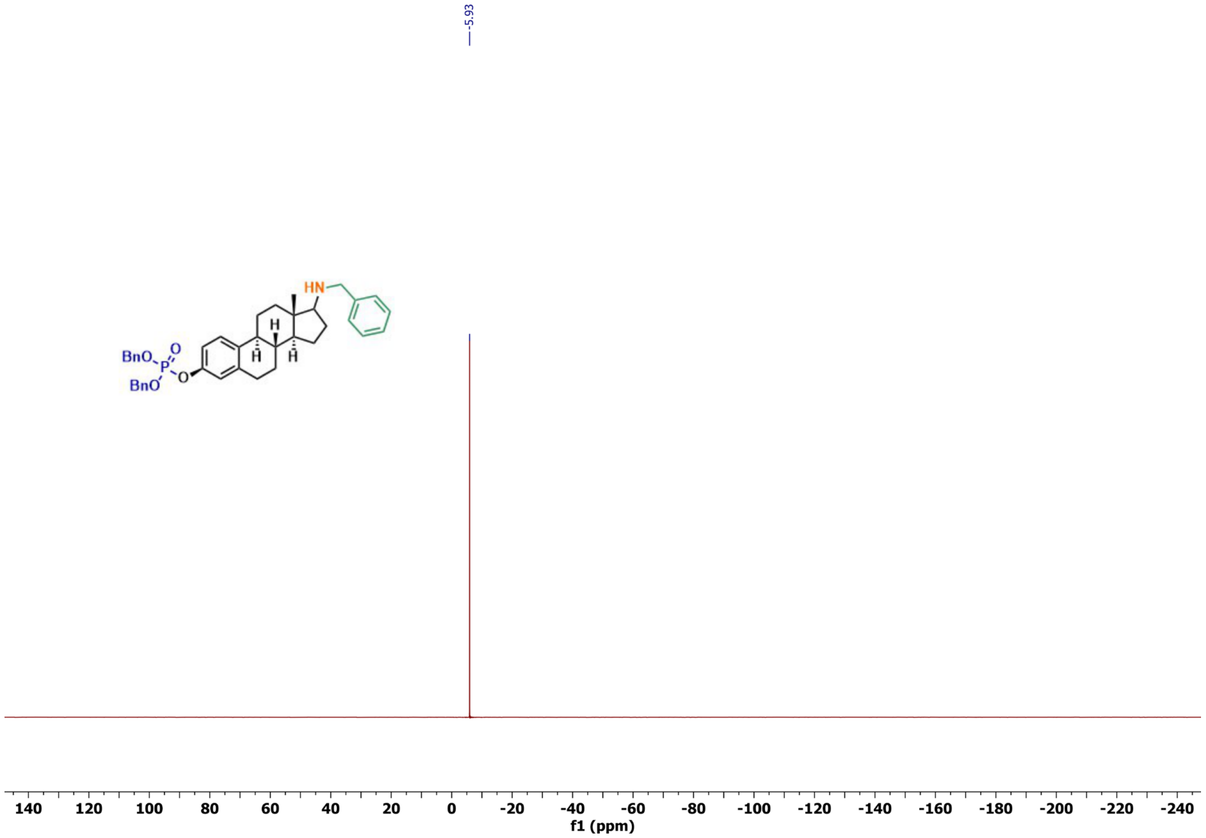

^{1}H NMR of Compound **27b** (600 MHz, DMSO-d_6)

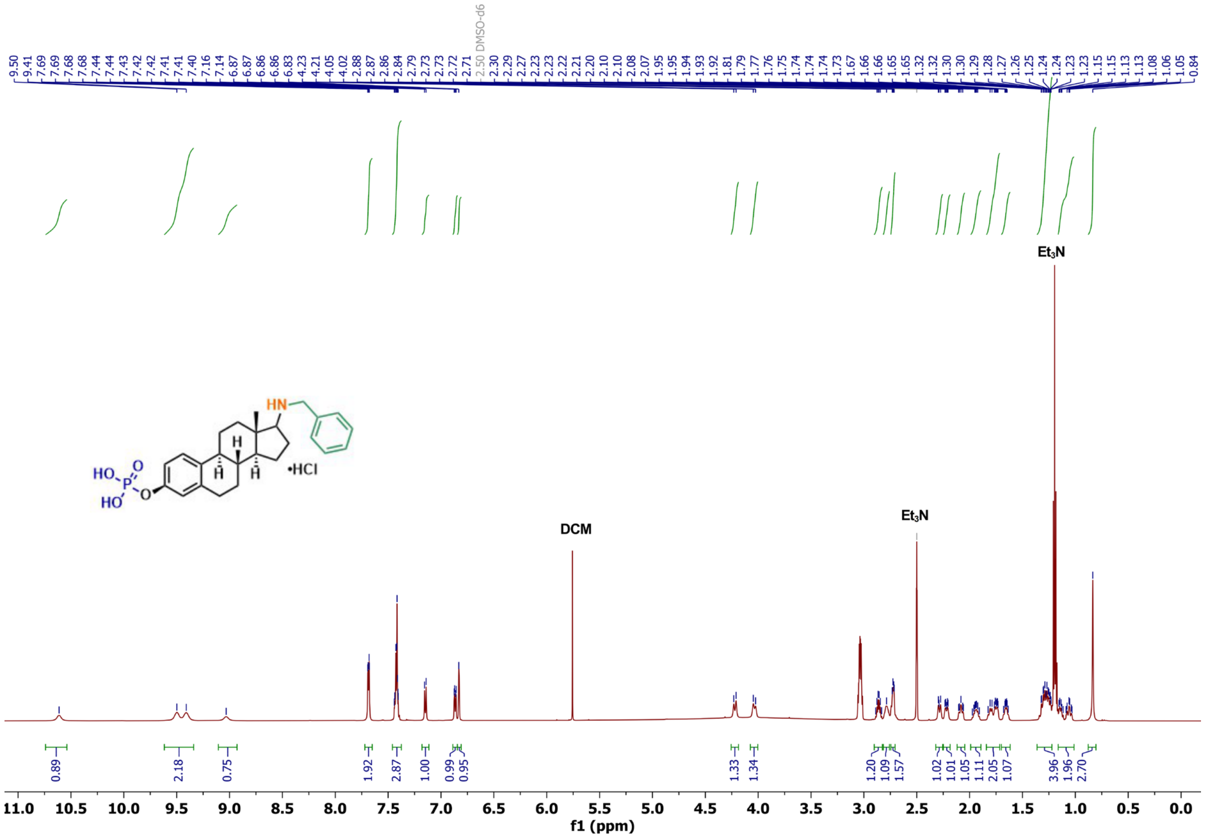

^{13}C NMR of Compound **27b** (151 MHz, DMSO-d_6)

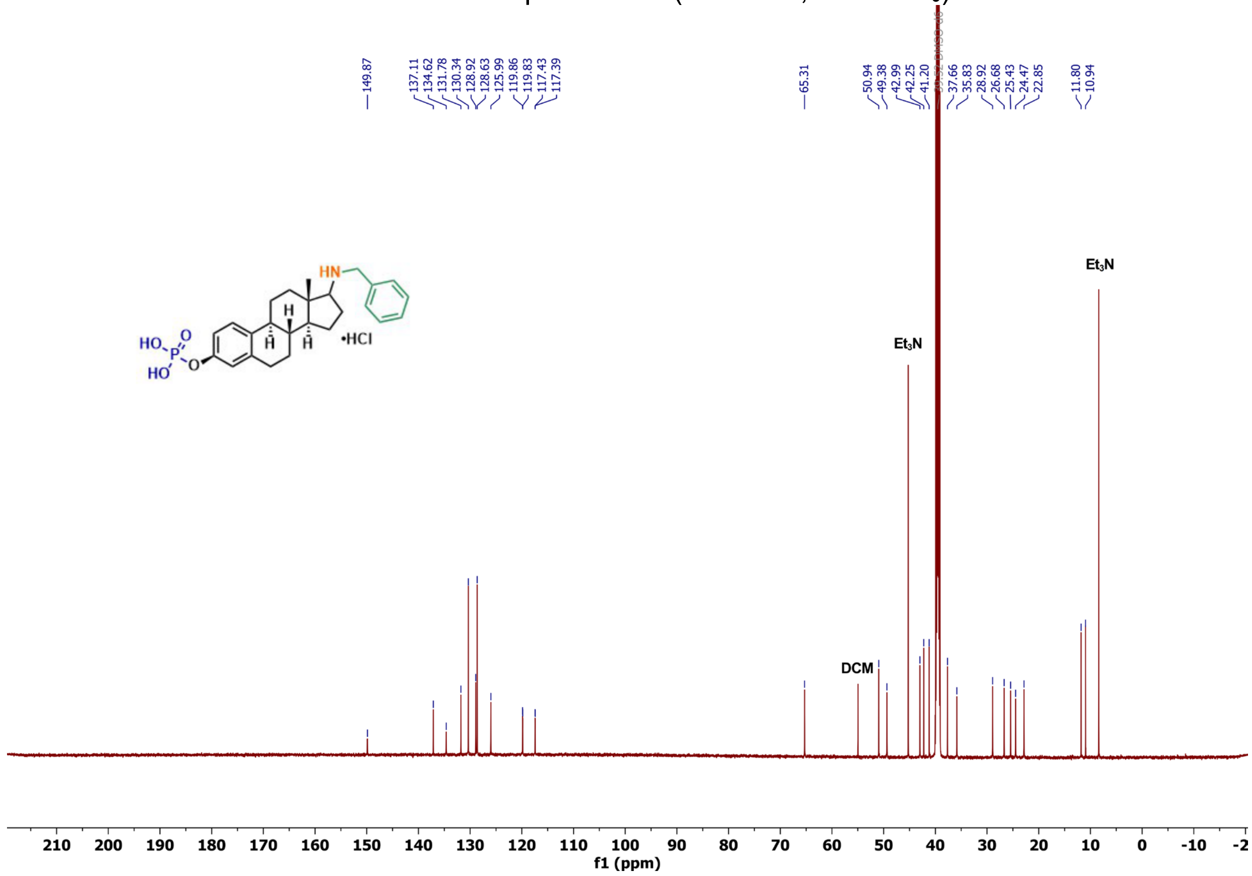

^{31}P NMR of Compound **27b** (243 MHz, DMSO-d_6)

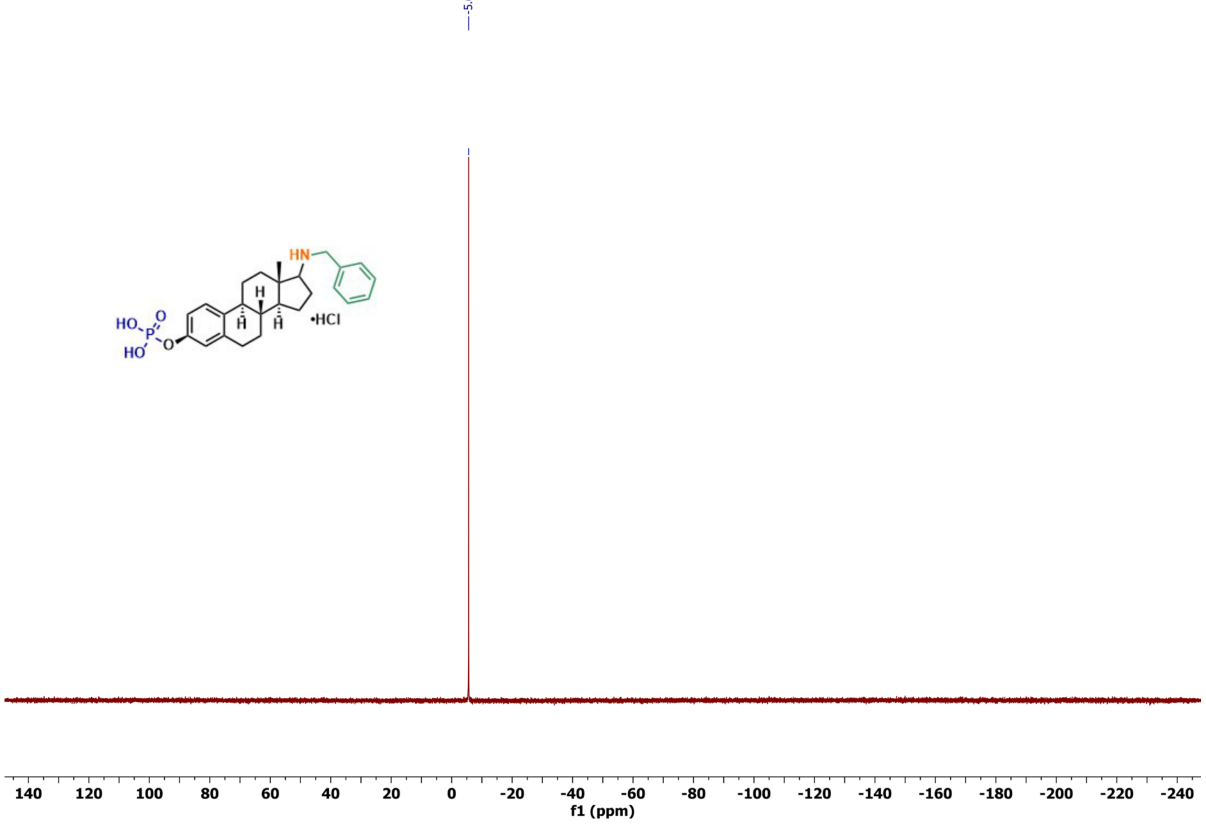

^{1}H NMR of Compound **28a** (600 MHz, $CDCl_3$)

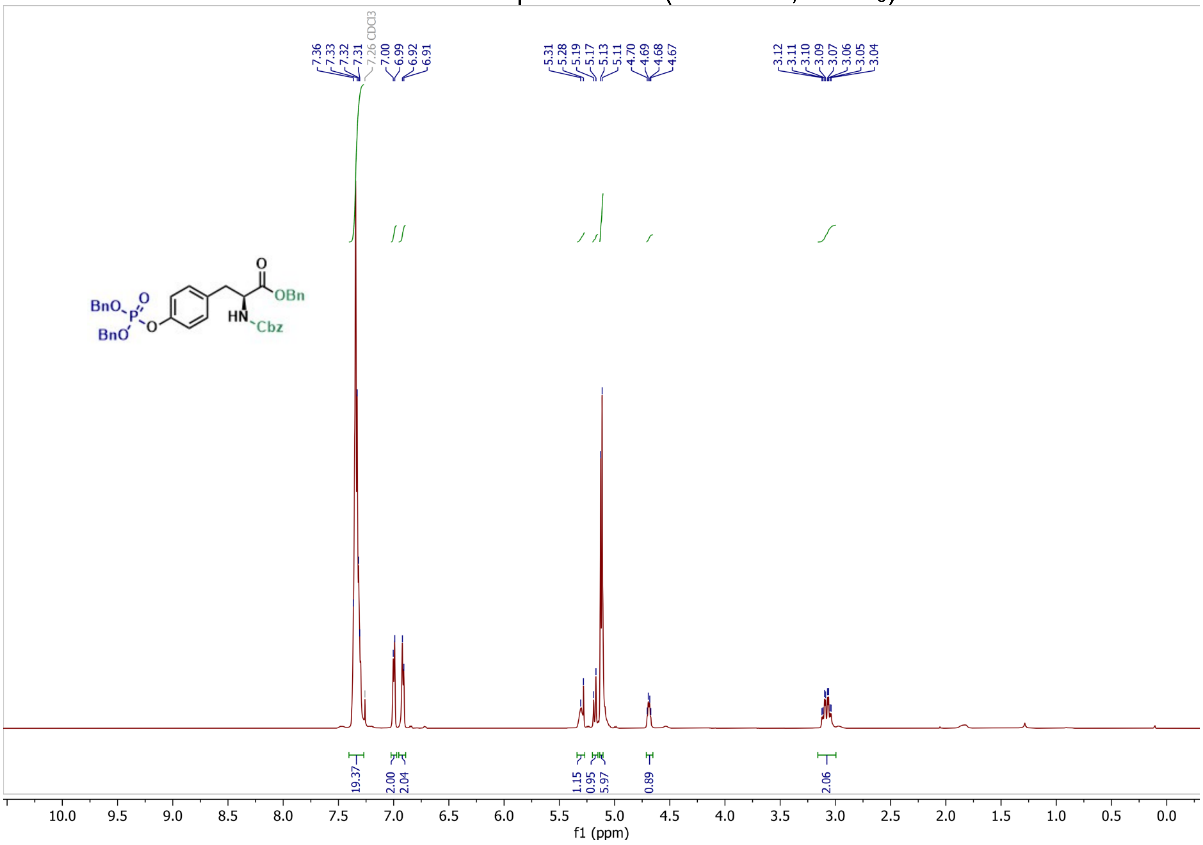

^{13}C NMR of Compound **28a** (151 MHz, $CDCl_3$)

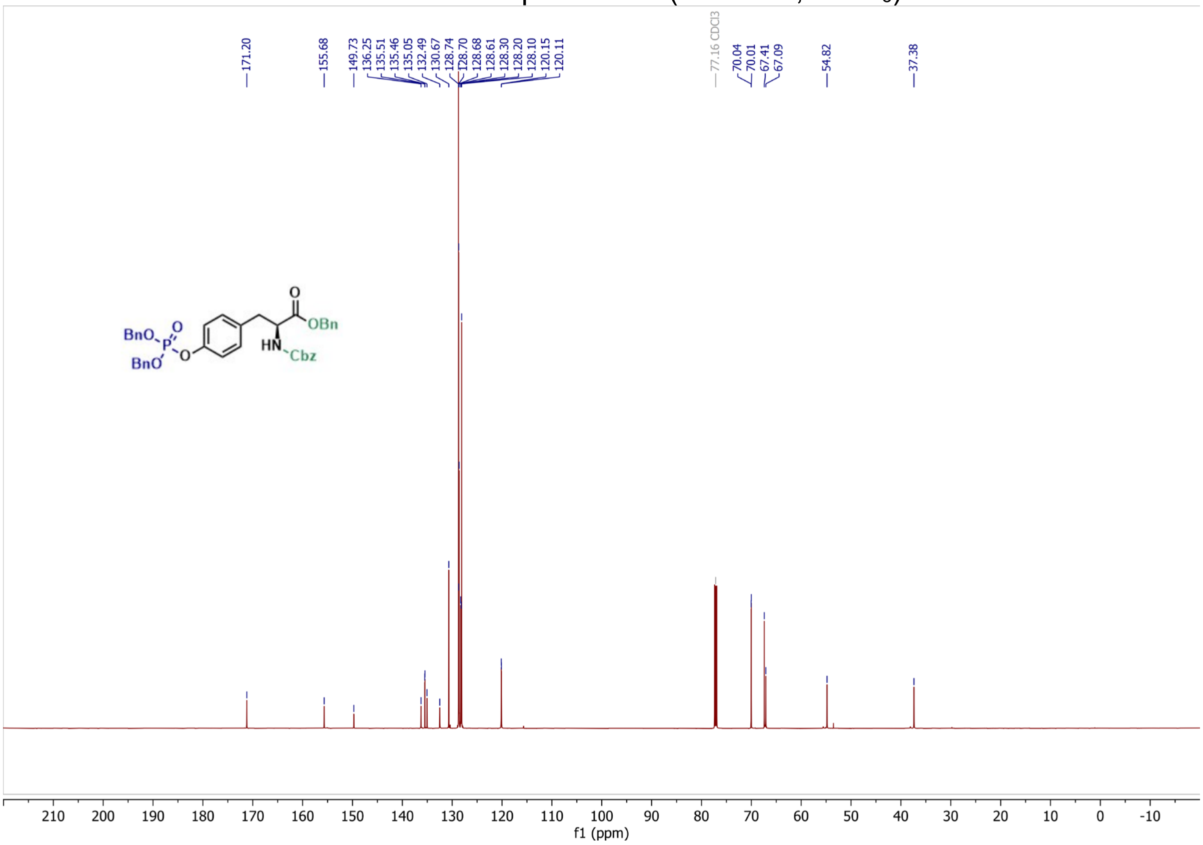

^{31}P NMR of Compound **28a** (243 MHz, $CDCl_3$)

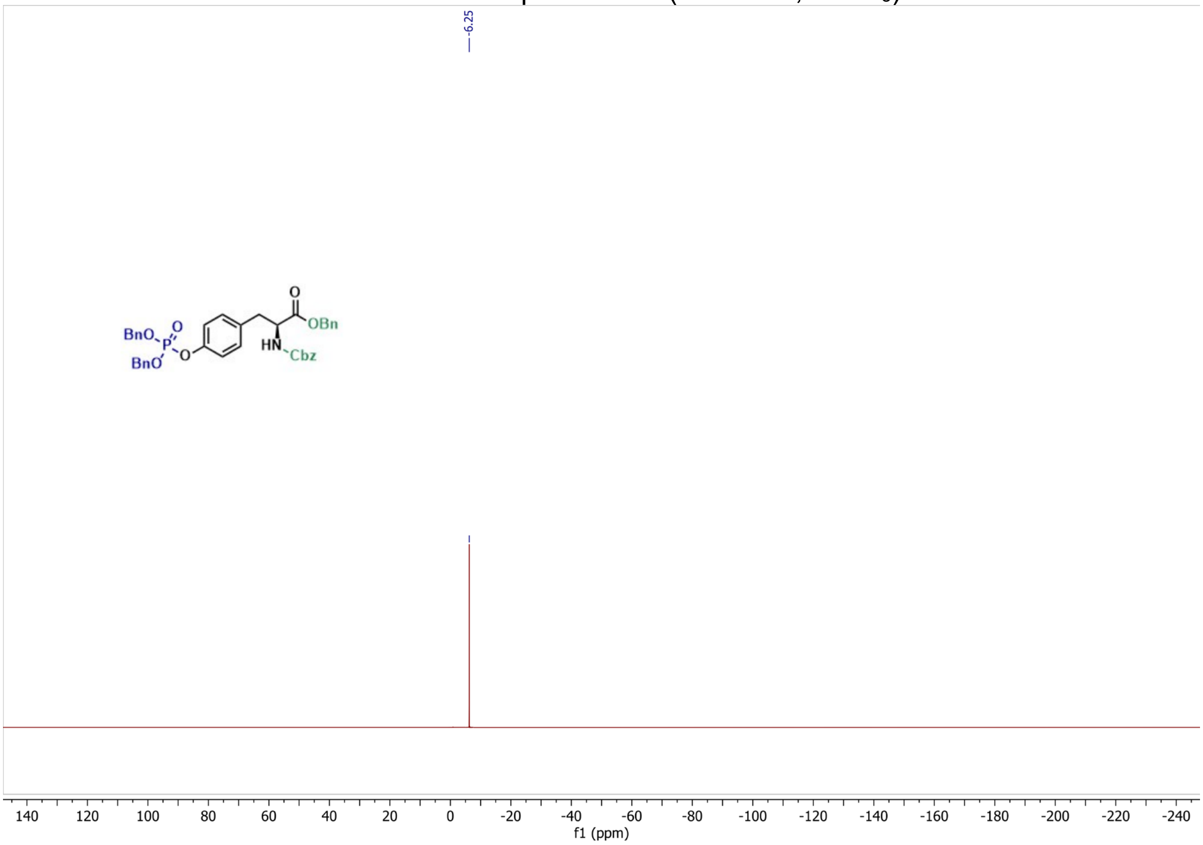

^{1}H NMR of Compound **28b** (600 MHz, $CDCl_3$)

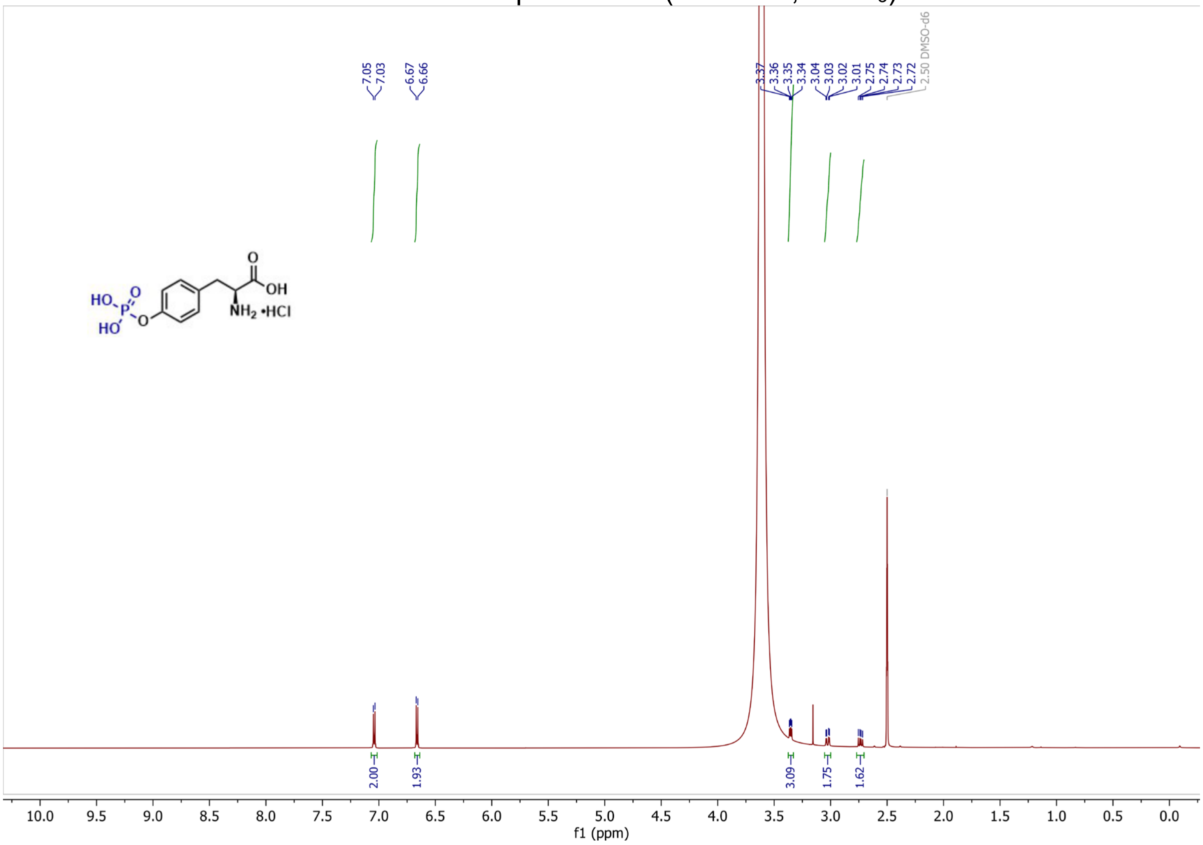

^{13}C NMR of Compound **28b** (151 MHz, $CDCl_3$)

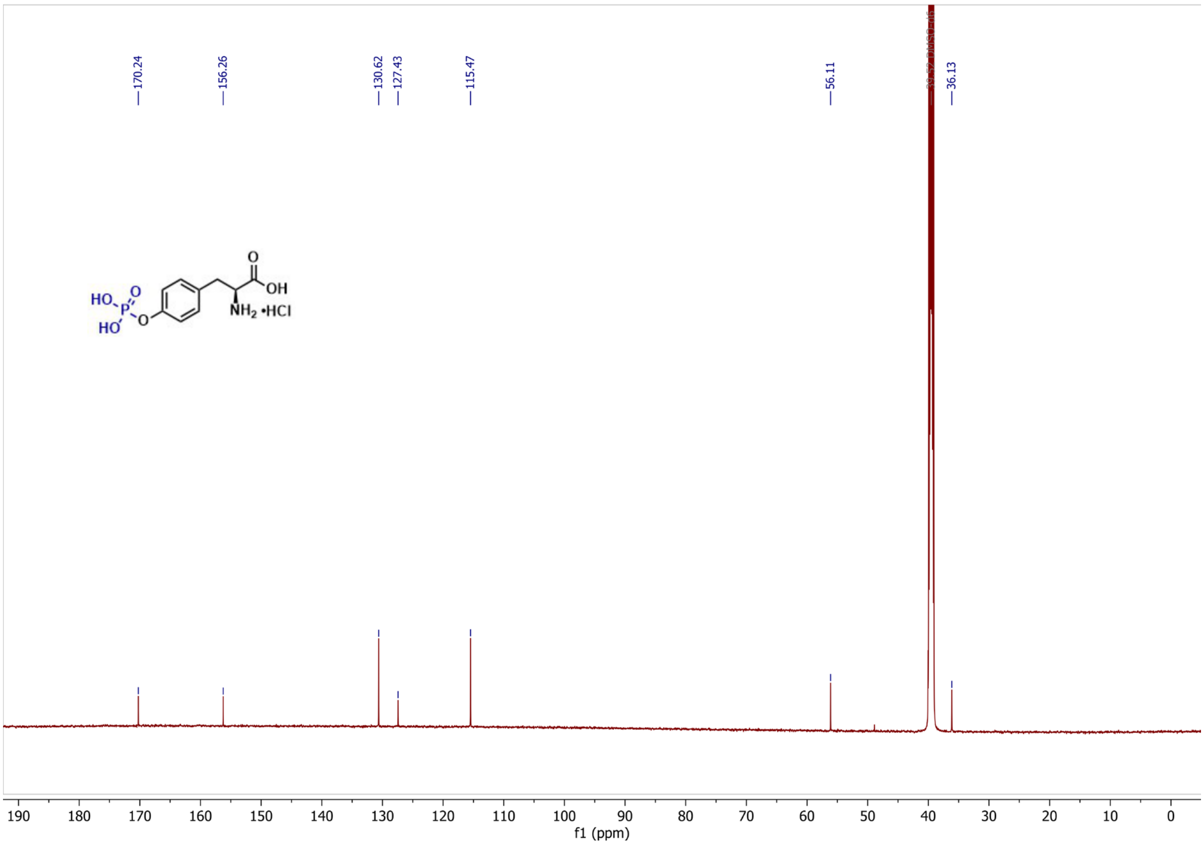

^{31}P NMR of Compound **28b** (243 MHz, $CDCl_3$)

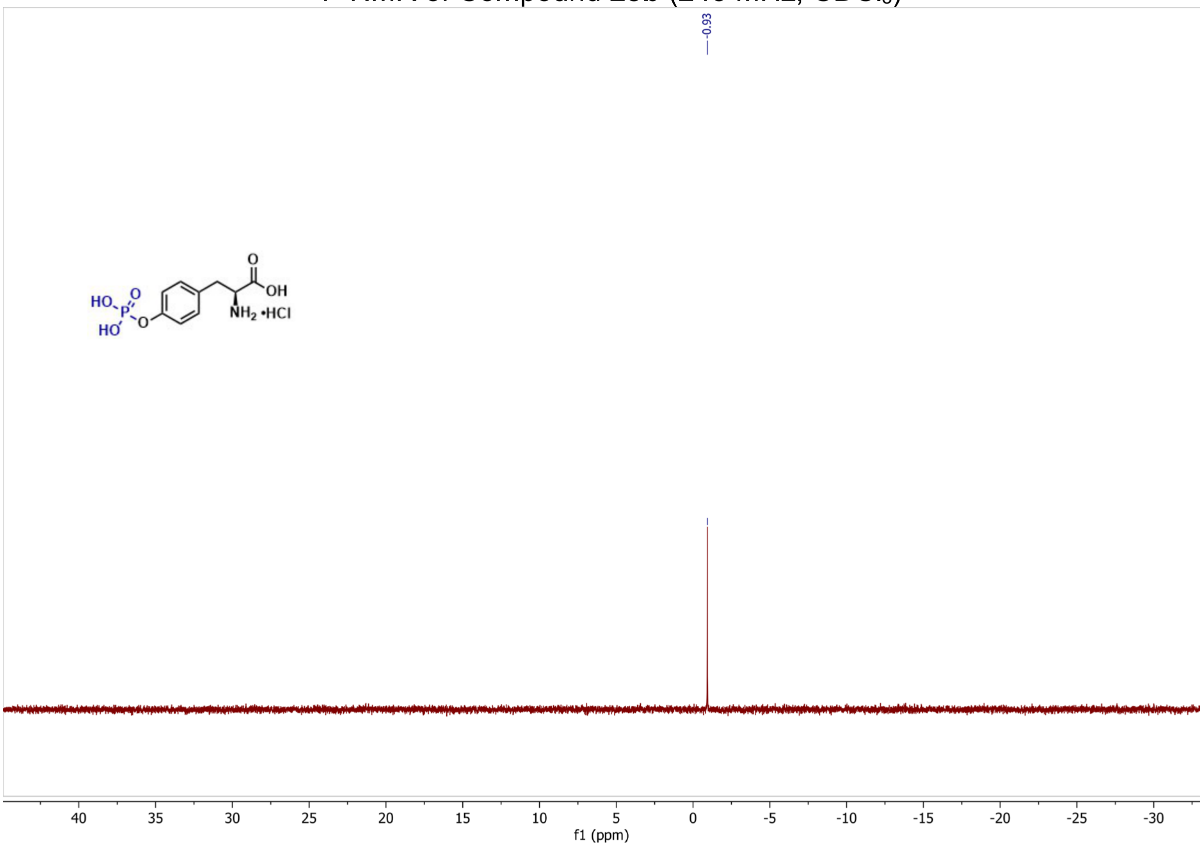

^{1}H NMR of Compound **29a** (600 MHz, $CDCl_3$)

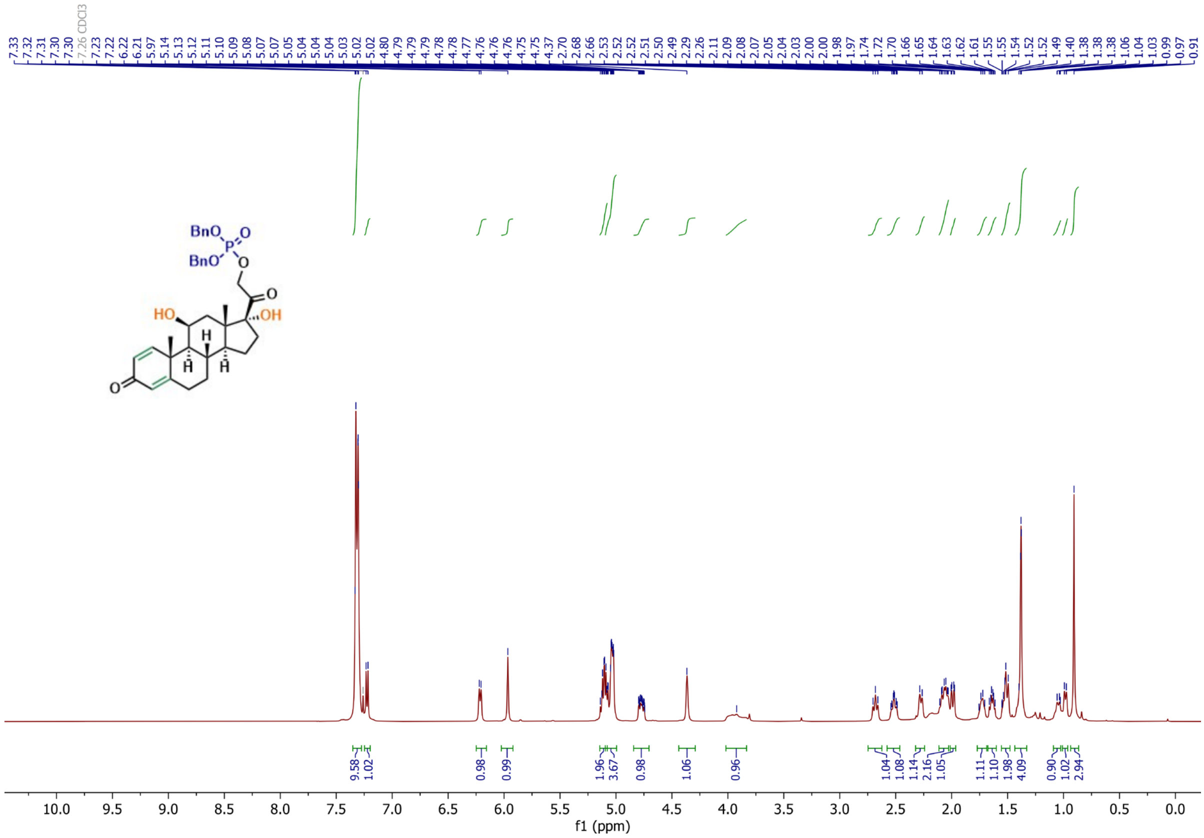

^{13}C NMR of Compound **29a** (151 MHz, $CDCl_3$)

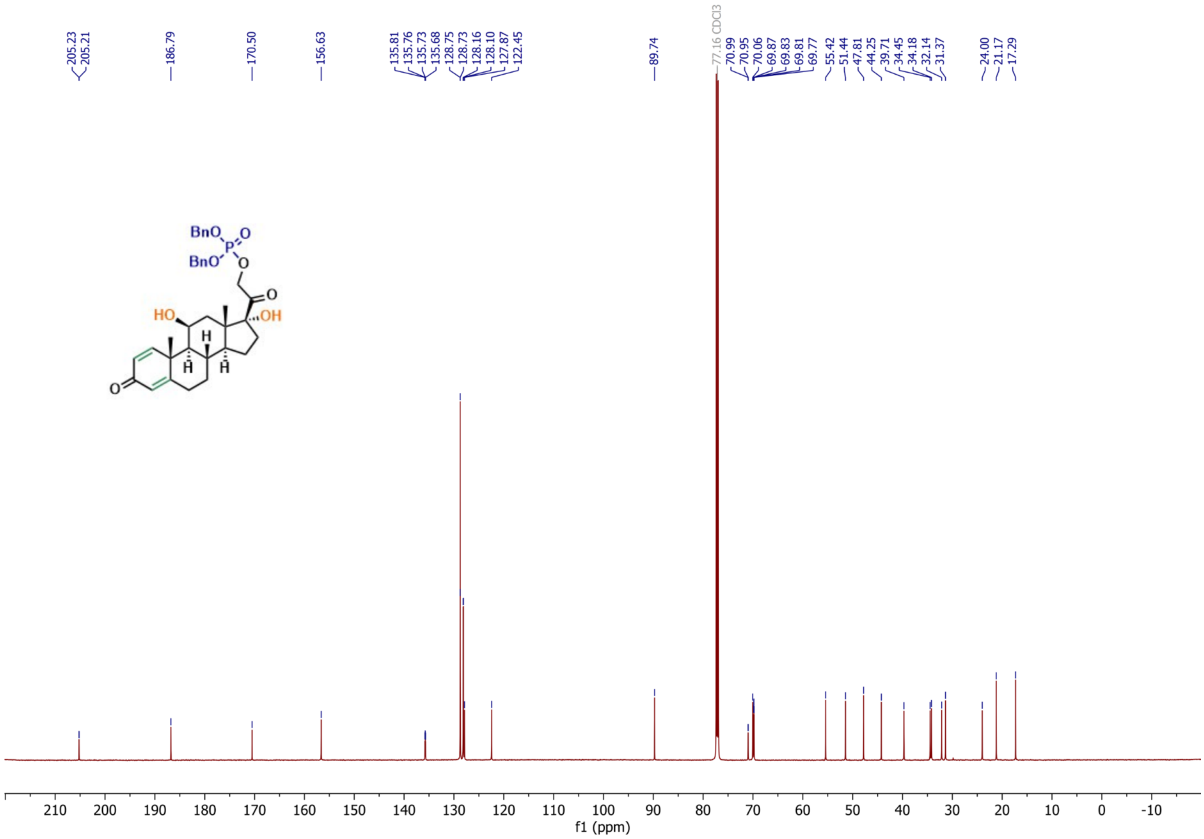

^{31}P NMR of Compound **29a** (243 MHz, $CDCl_3$)

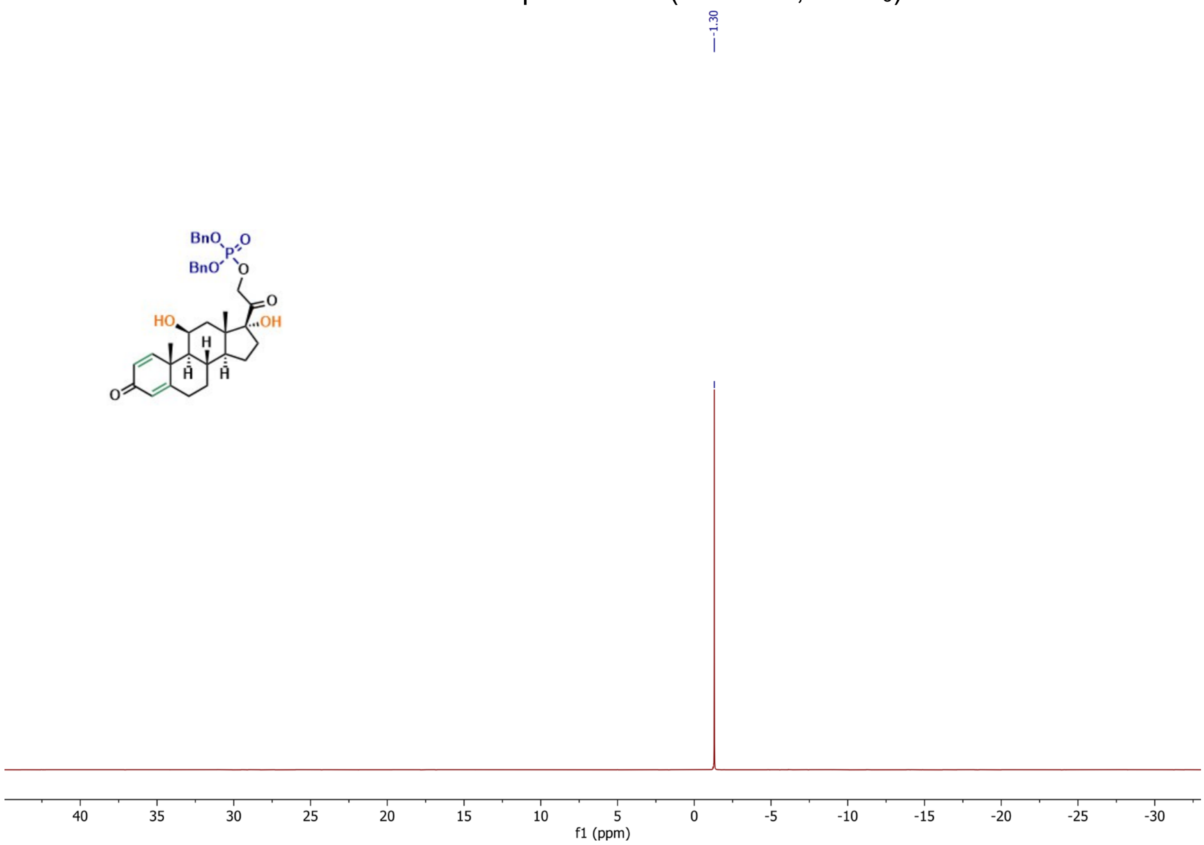

^{1}H NMR of Compound **29b** (600 MHz, $CDCl_3$)

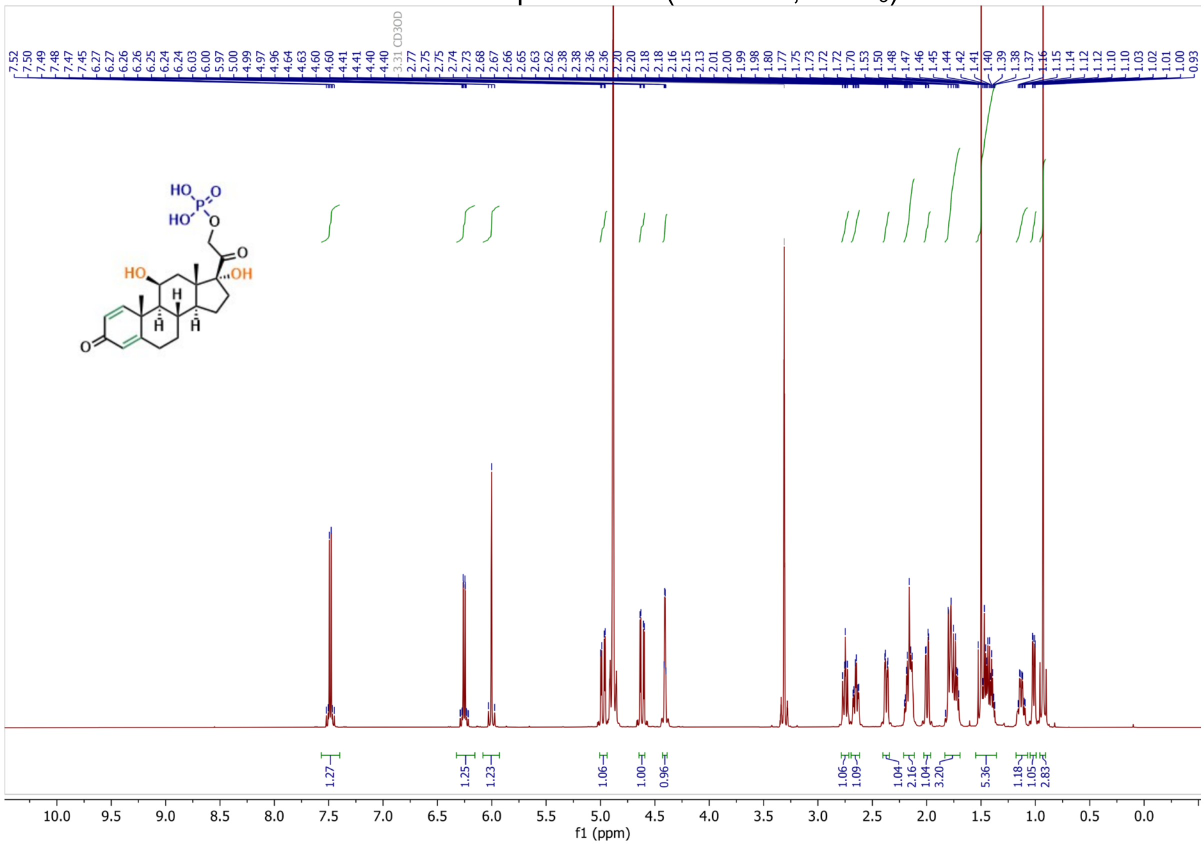

^{13}C NMR of Compound **29b** (151 MHz, $CDCl_3$)

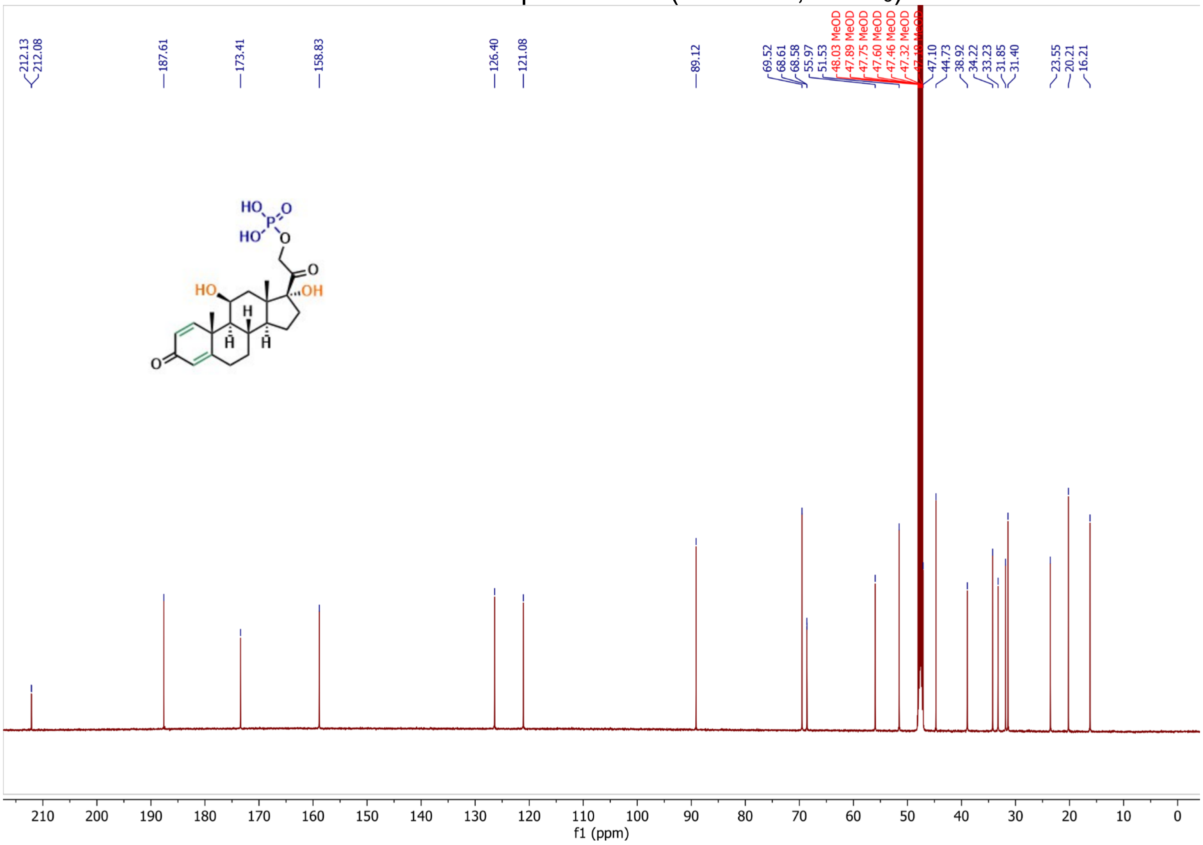

^{31}P NMR of Compound **29b** (243 MHz, $CDCl_3$)

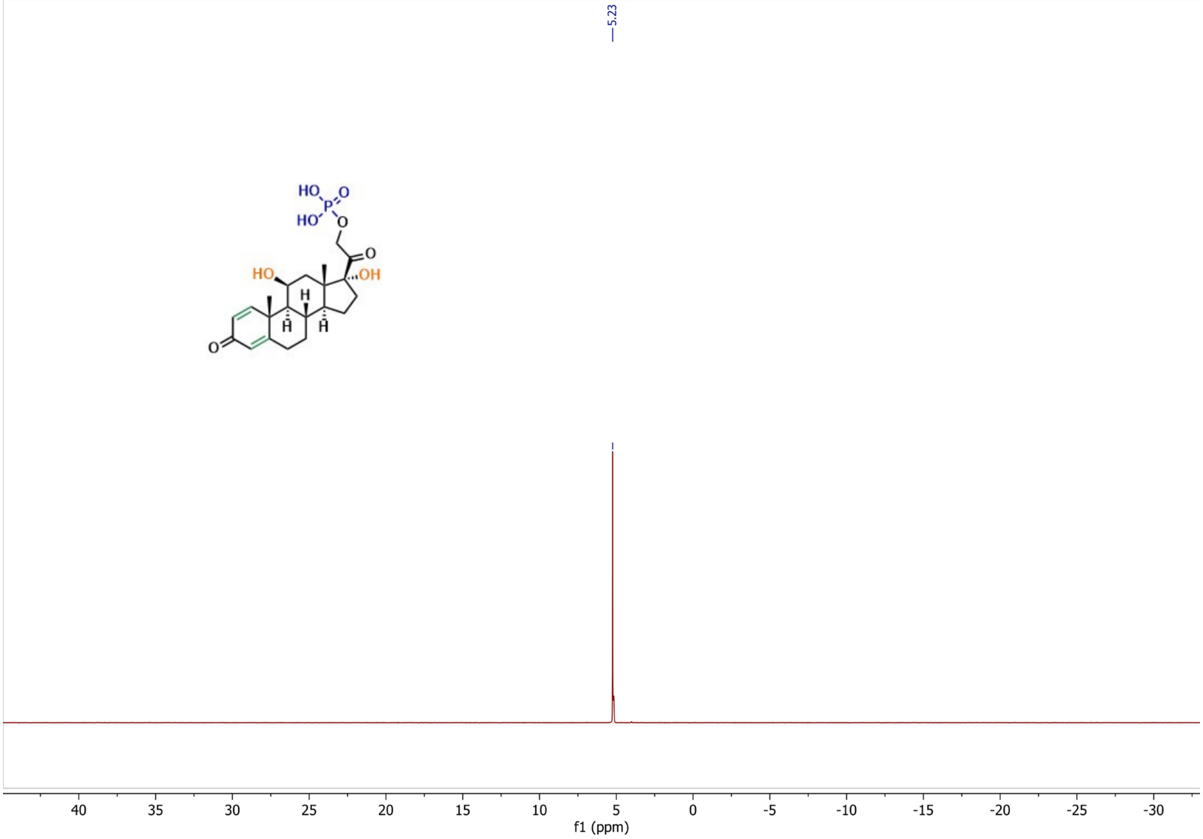

^{1}H NMR of Compound **30a** (600 MHz, MeOD)

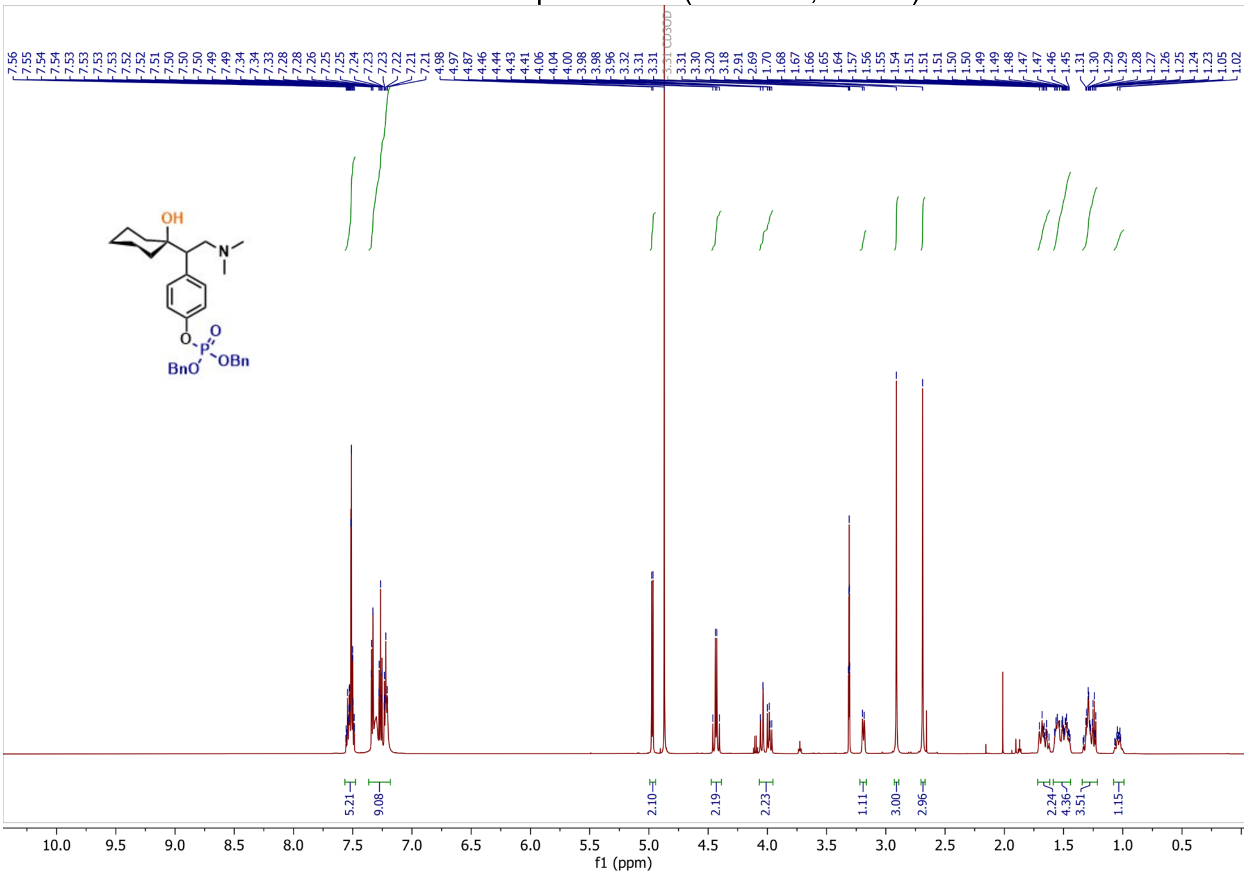

^{13}C NMR of Compound **30a** (151 MHz, MeOD)

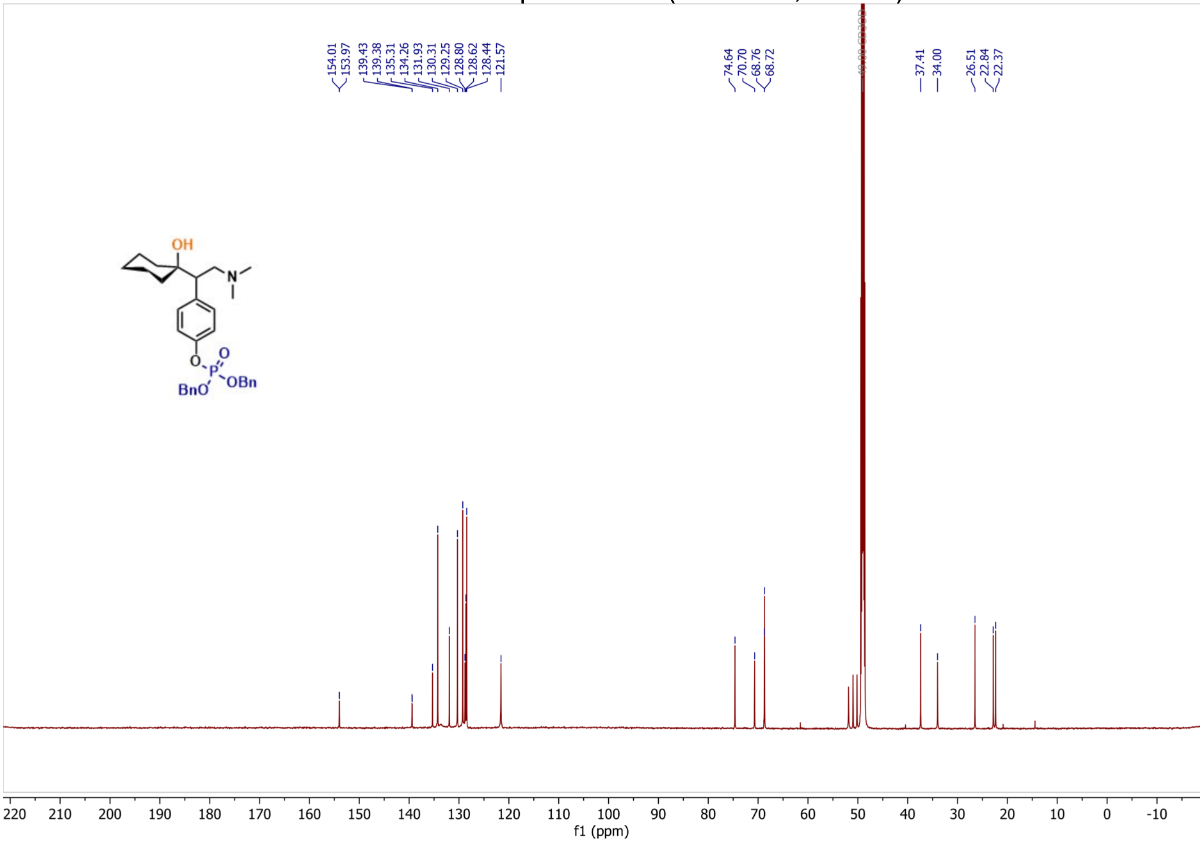

^{31}P NMR of Compound **30a** (243 MHz, MeOD)

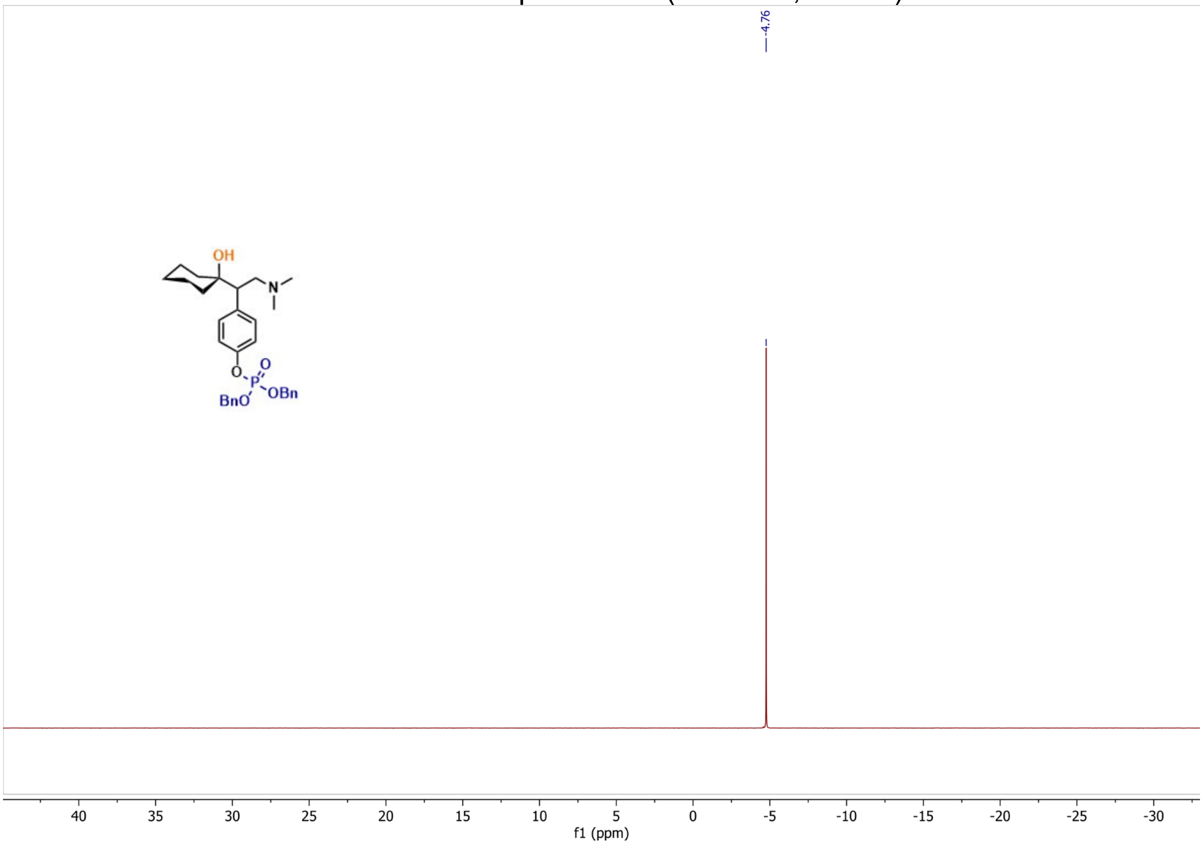

^{1}H NMR of Compound **30b** (600 MHz, MeOD)

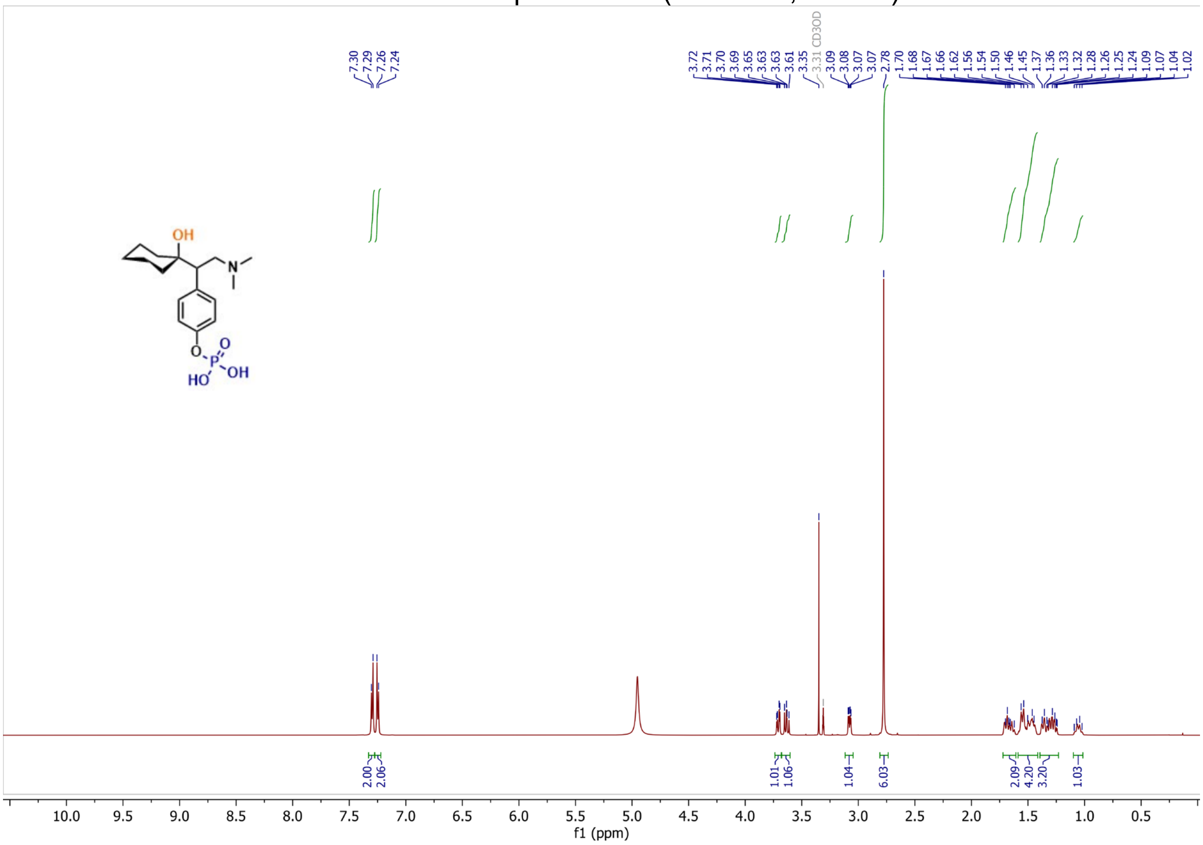

^{13}C NMR of Compound **30b** (151 MHz, MeOD)

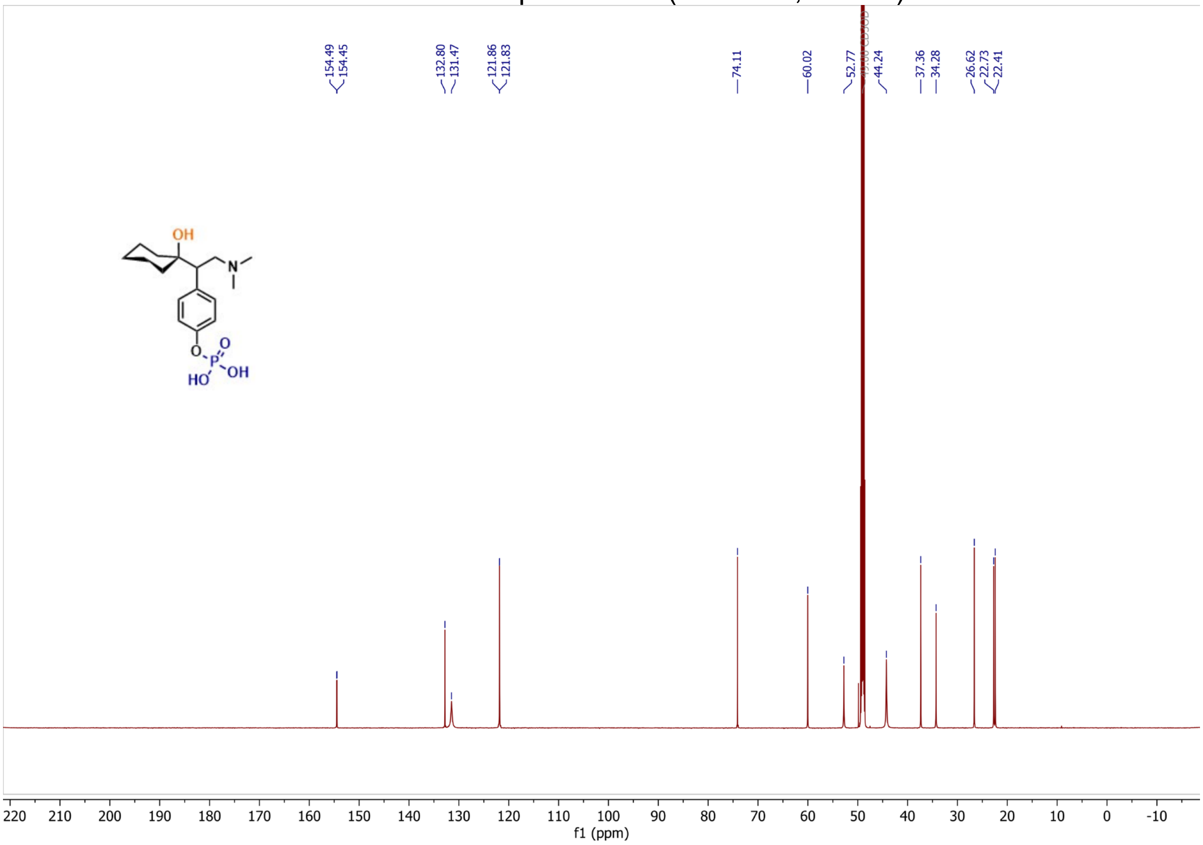

^{31}P NMR of Compound **30b** (243 MHz, MeOD)

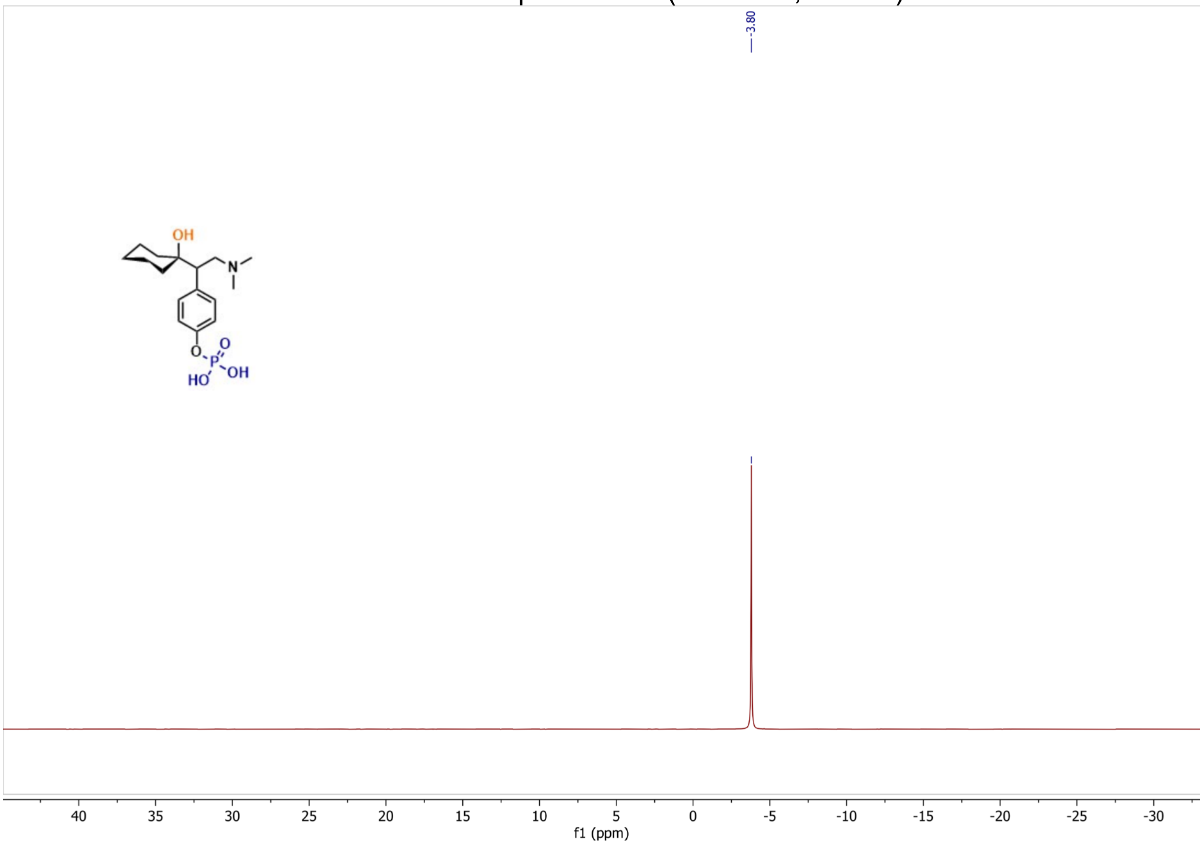

^{1}H NMR of Compound **31a** (600 MHz, $CDCl_3$:MeOD (2:1))

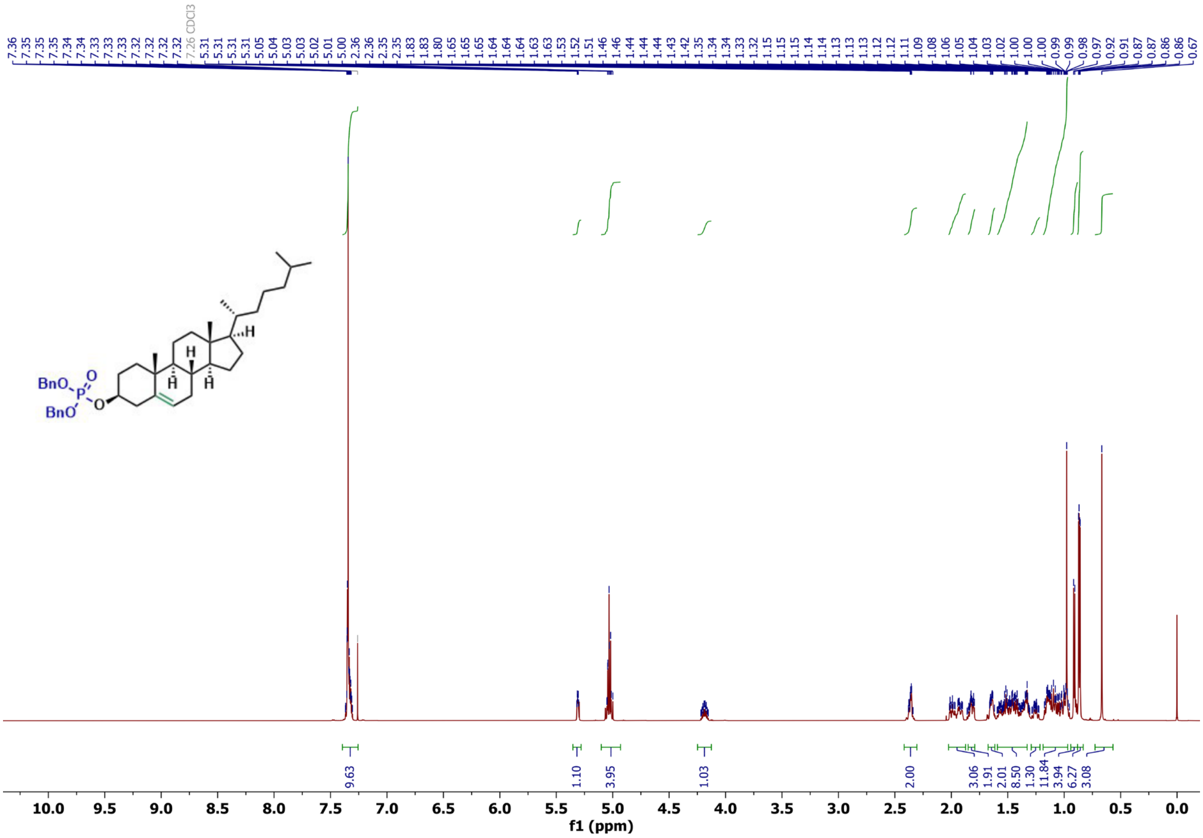

^{13}C NMR of Compound **31a** (151 MHz, $CDCl_3$:MeOD (2:1))

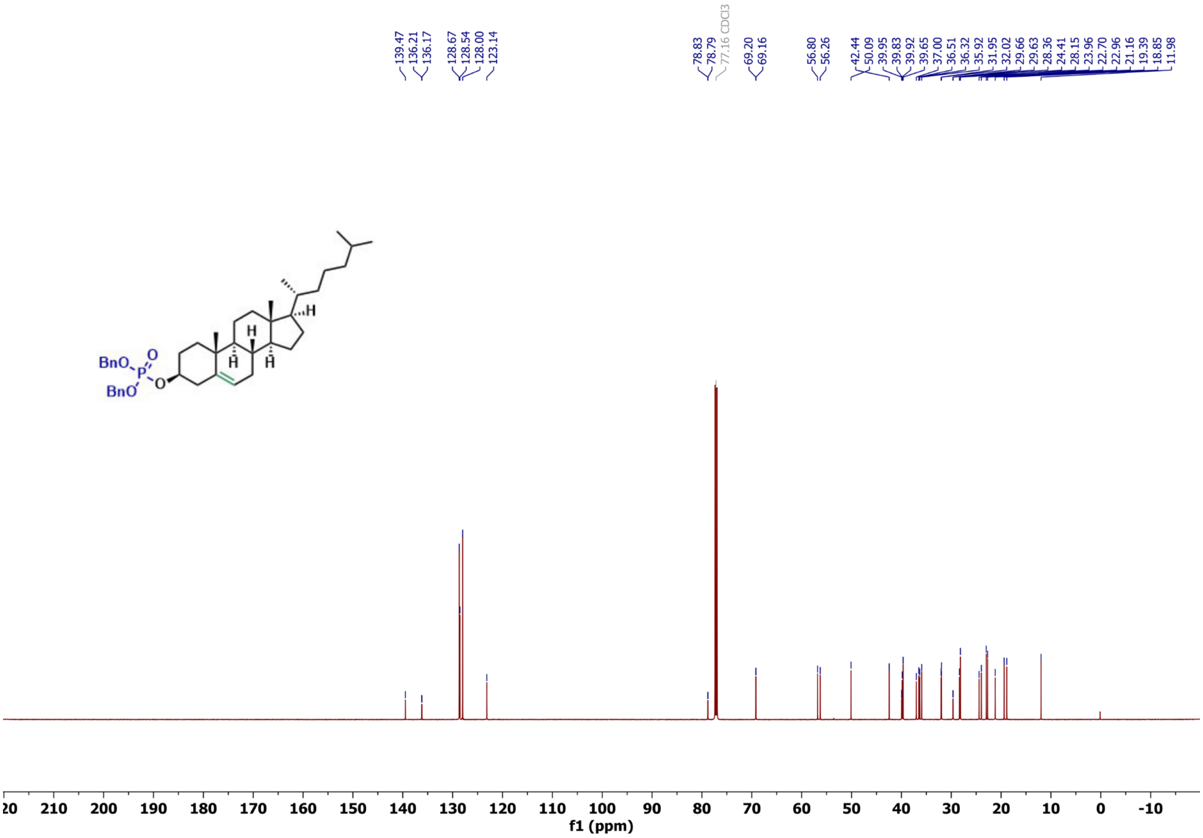

^{31}P NMR of Compound **31a** (243 MHz, $CDCl_3$:MeOD (2:1))

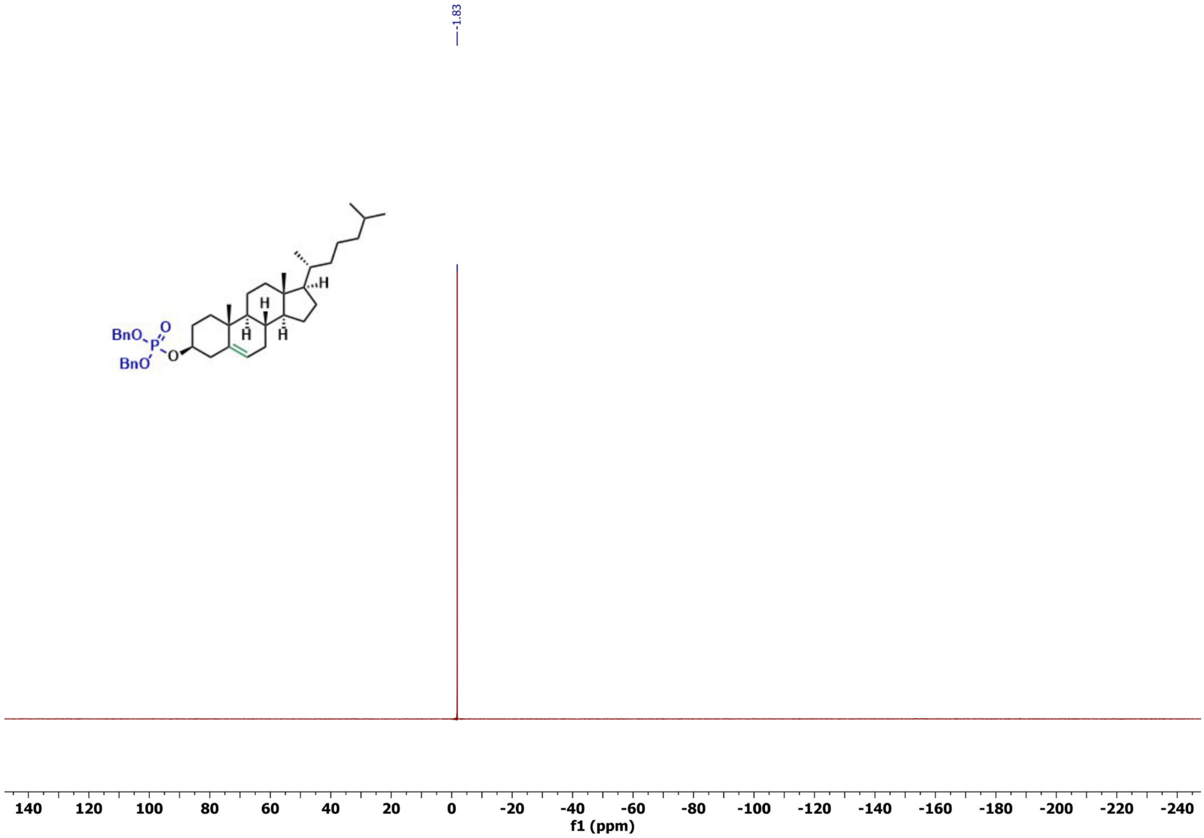

^{1}H NMR of Compound **31b** (600 MHz, $CDCl_3$:MeOD (2:1))

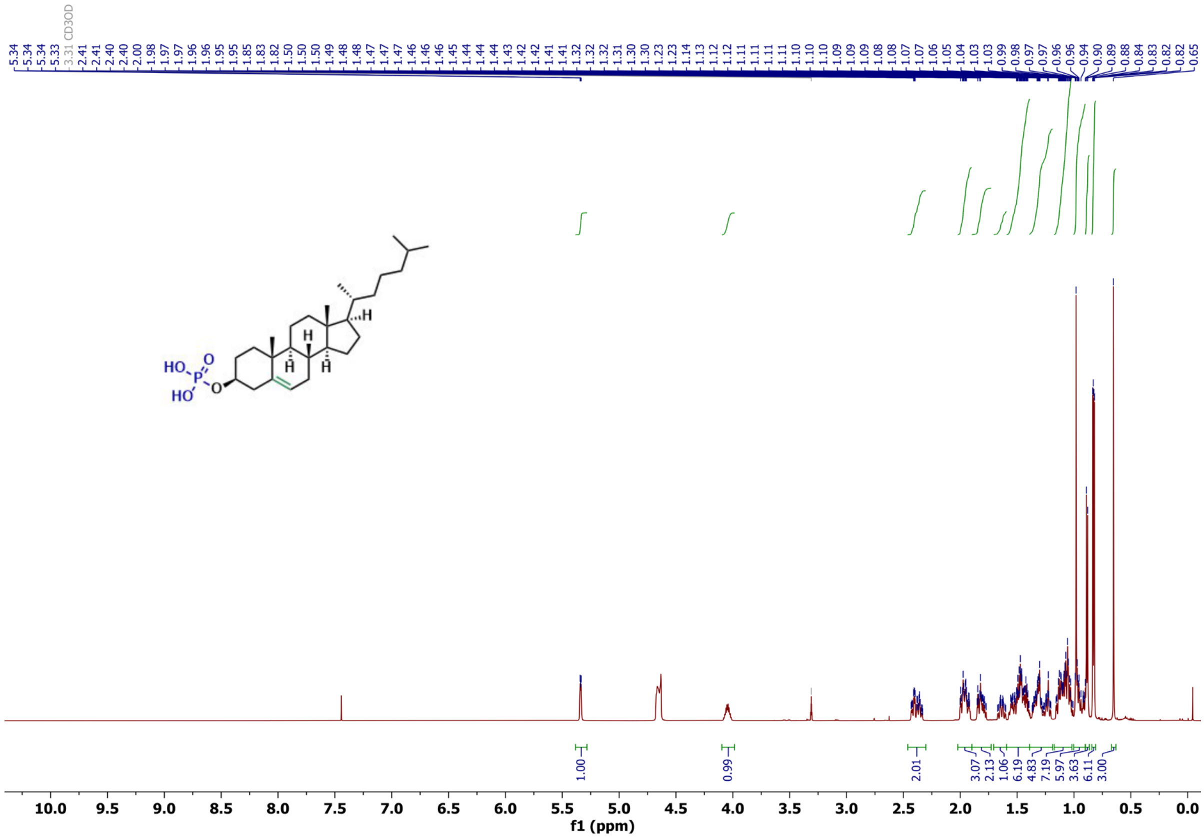

^{13}C NMR of Compound **31b** (151 MHz, $CDCl_3$:MeOD (2:1))

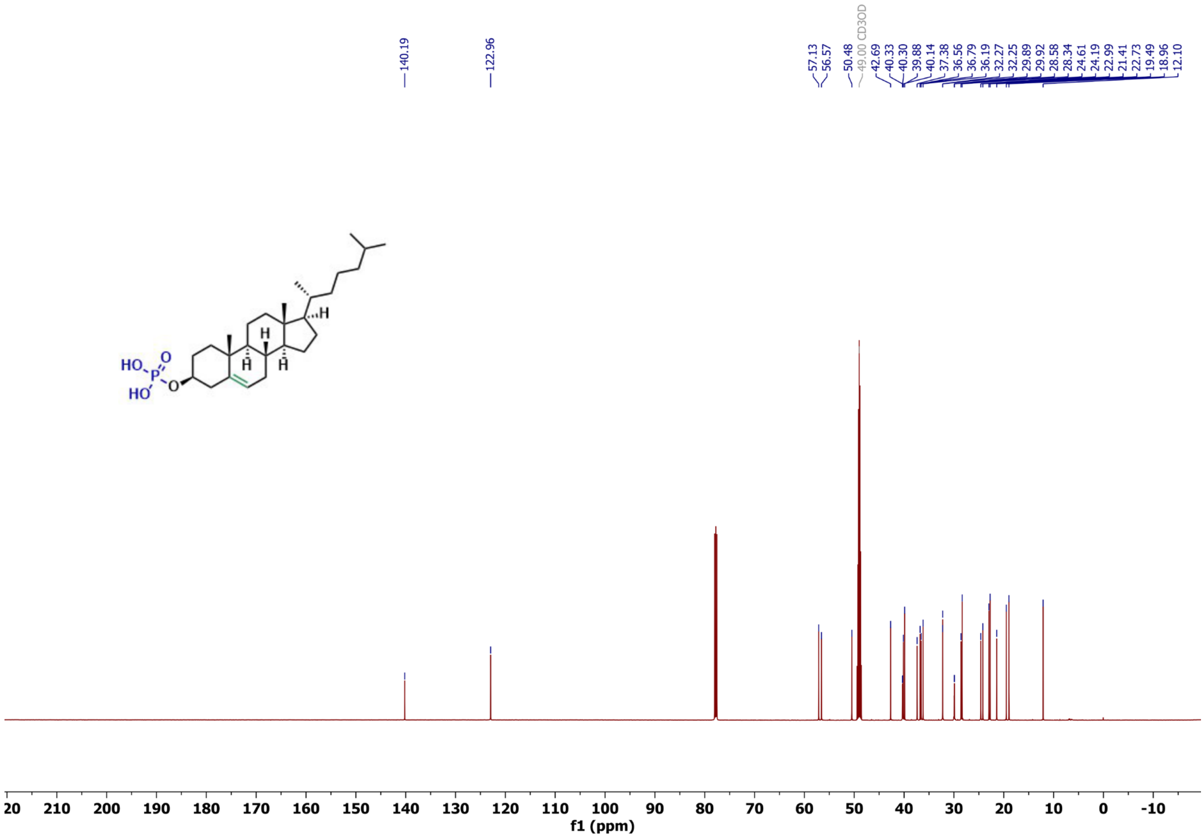

^{31}P NMR of Compound **31b** (243 MHz, $CDCl_3$:MeOD (2:1))

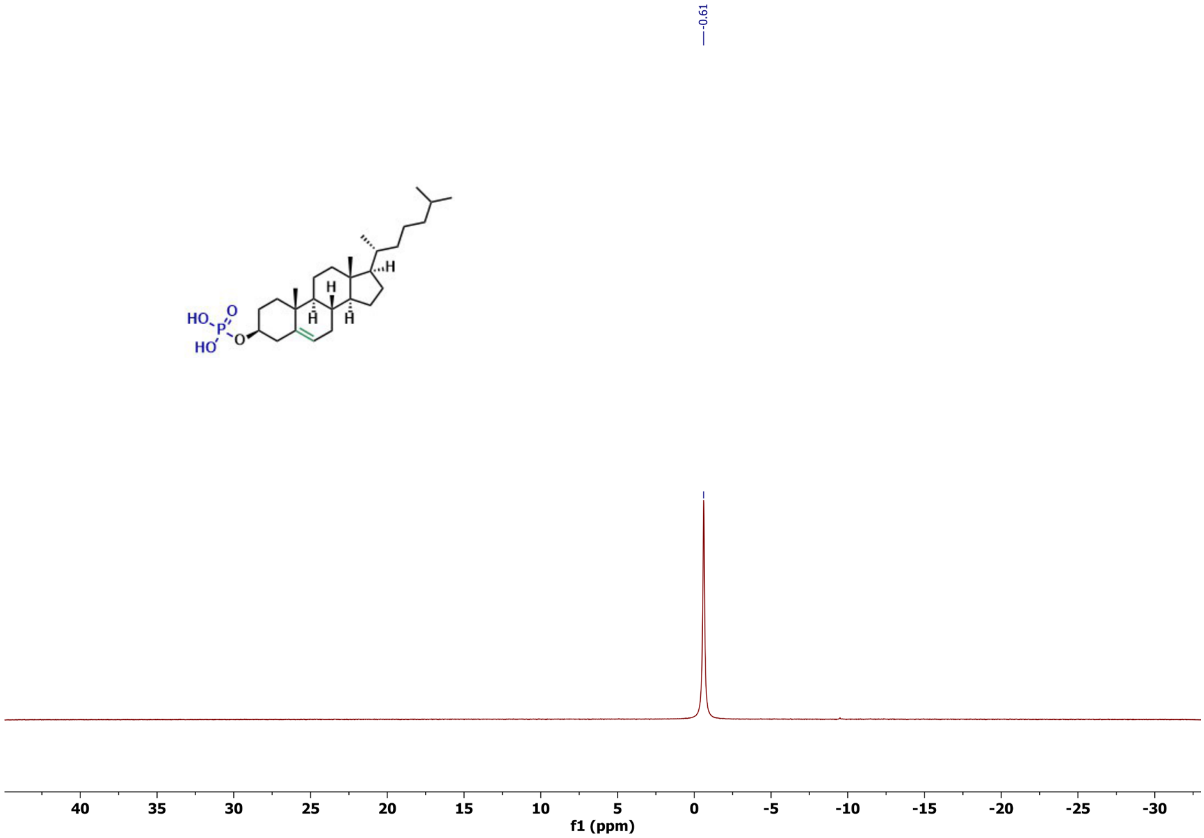

^{1}H NMR of Compound **32a** (400 MHz, MeOD)

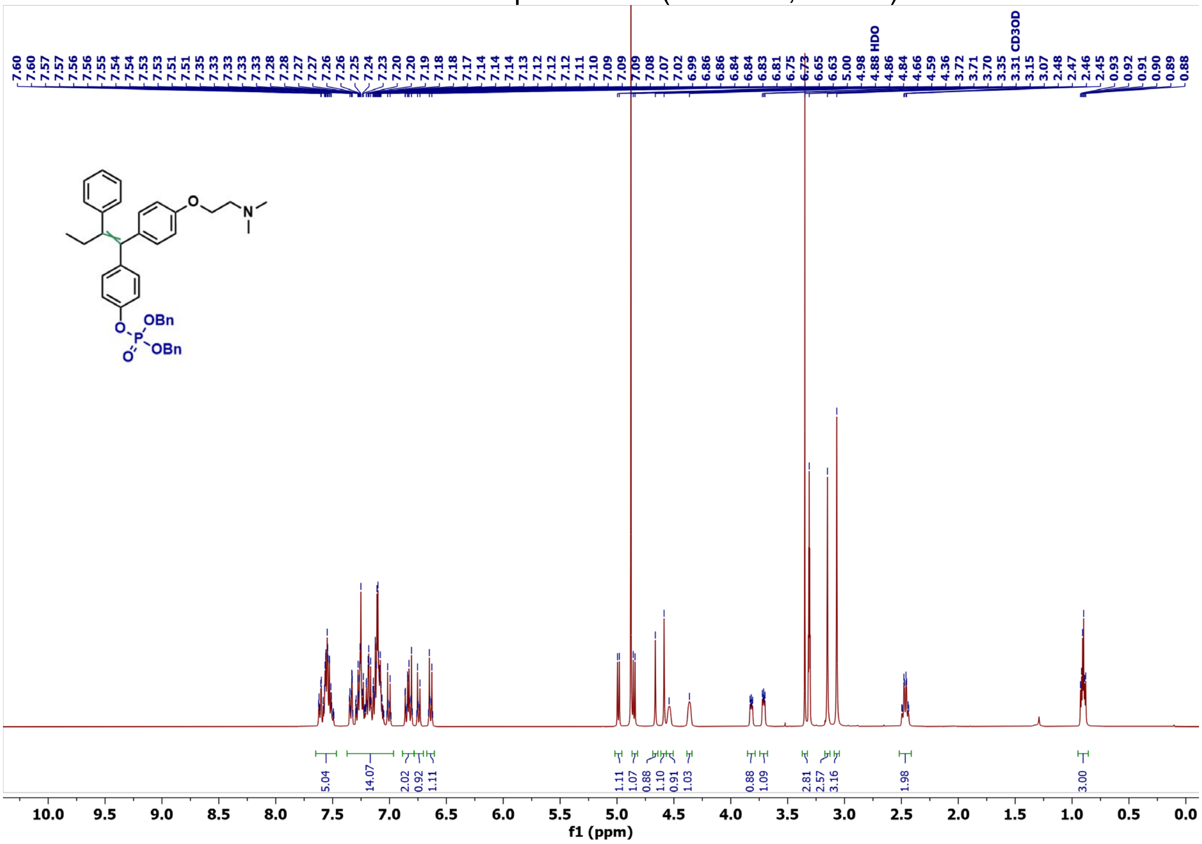

^{13}C NMR of Compound **32a** (101 MHz, MeOD)

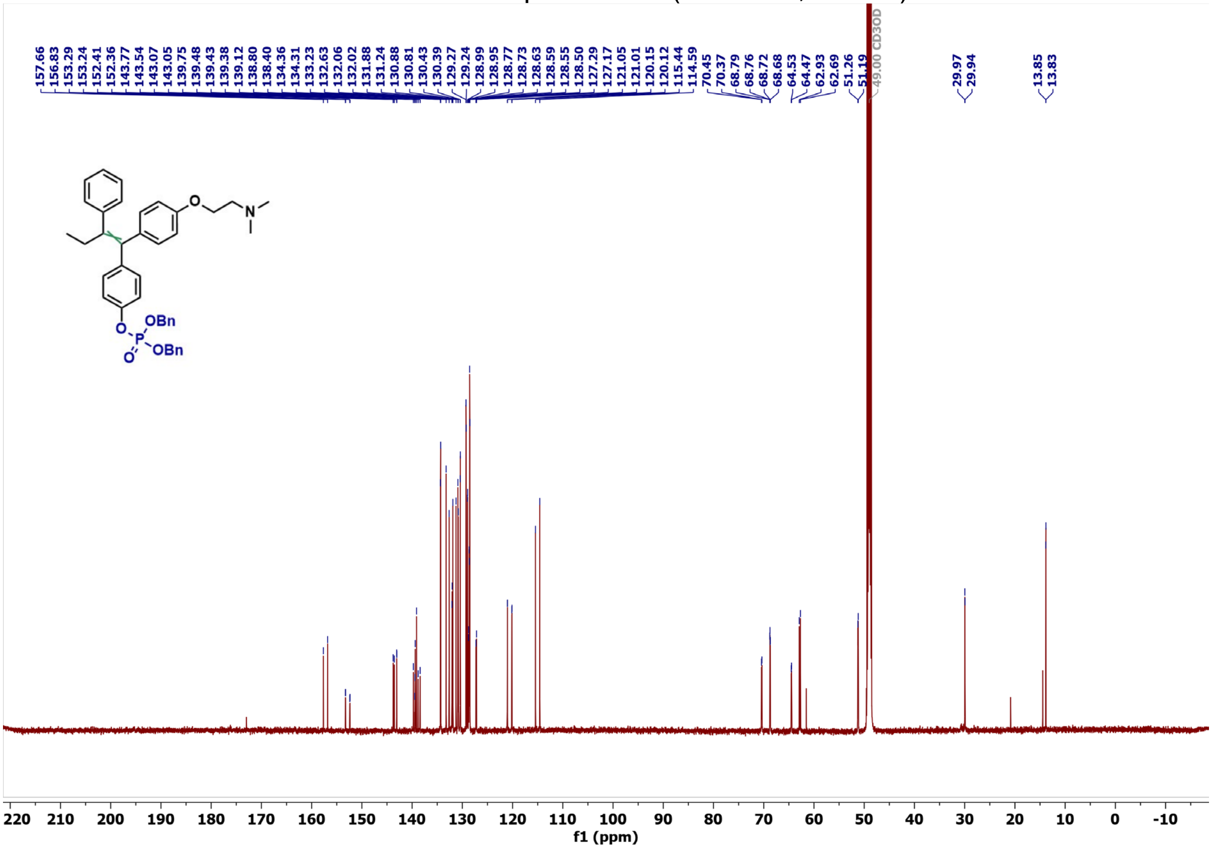

^{31}P NMR of Compound **32a** (162 MHz, MeOD)

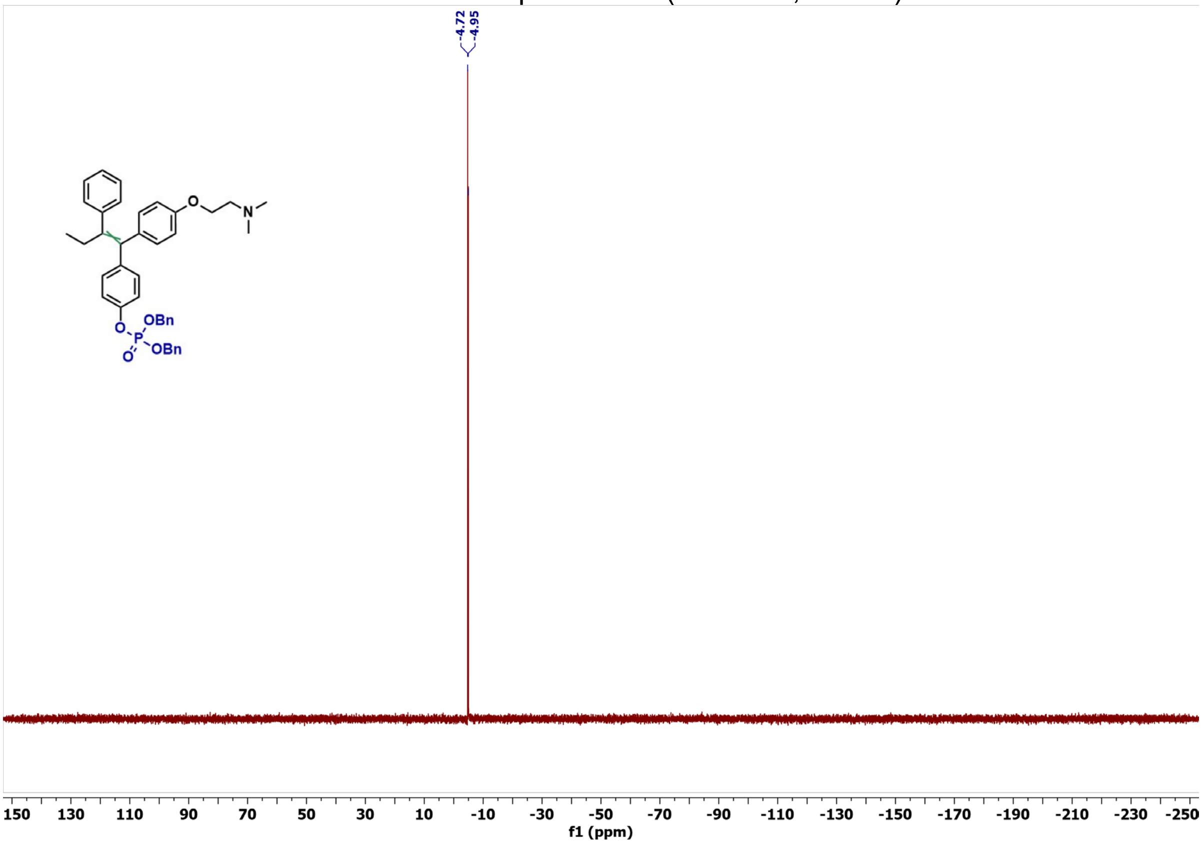

^{1}H NMR of Compound **32b** (400 MHz, DMSO-d_6)

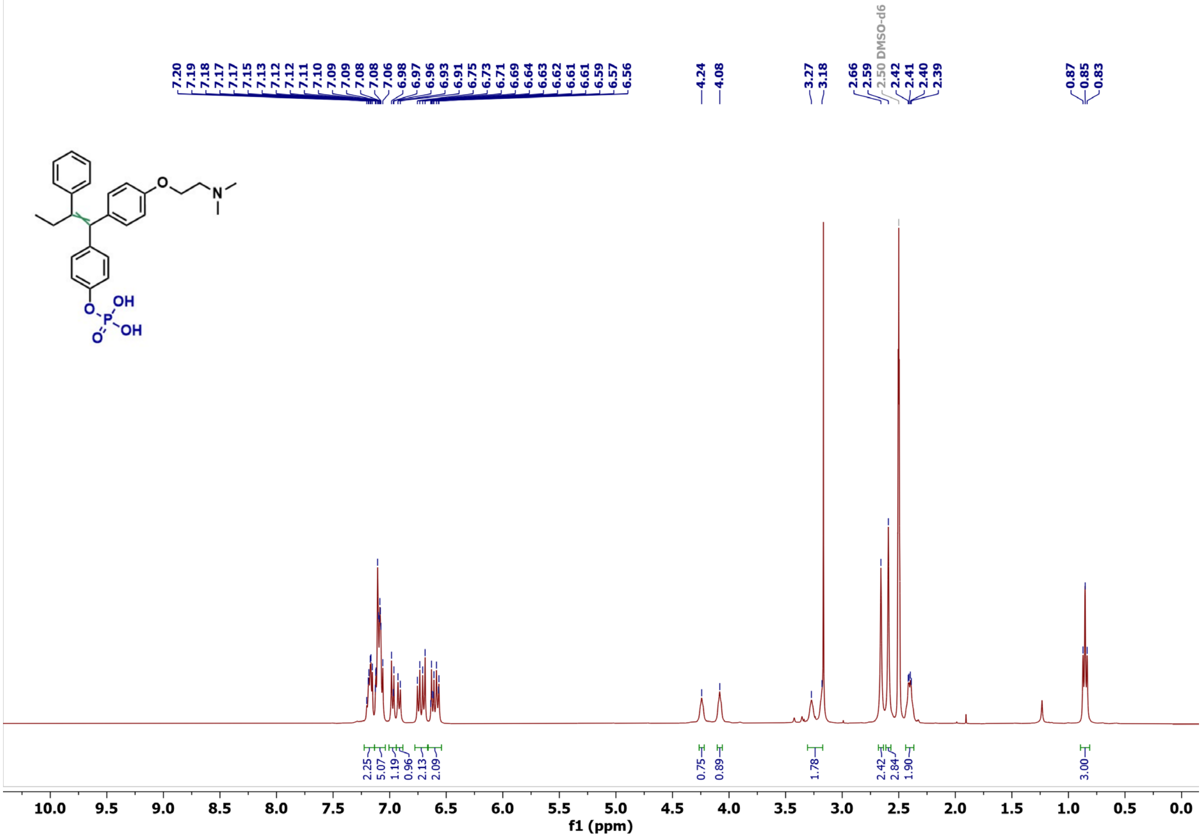

^{13}C NMR of Compound **32b** (101 MHz, DMSO-d_6)

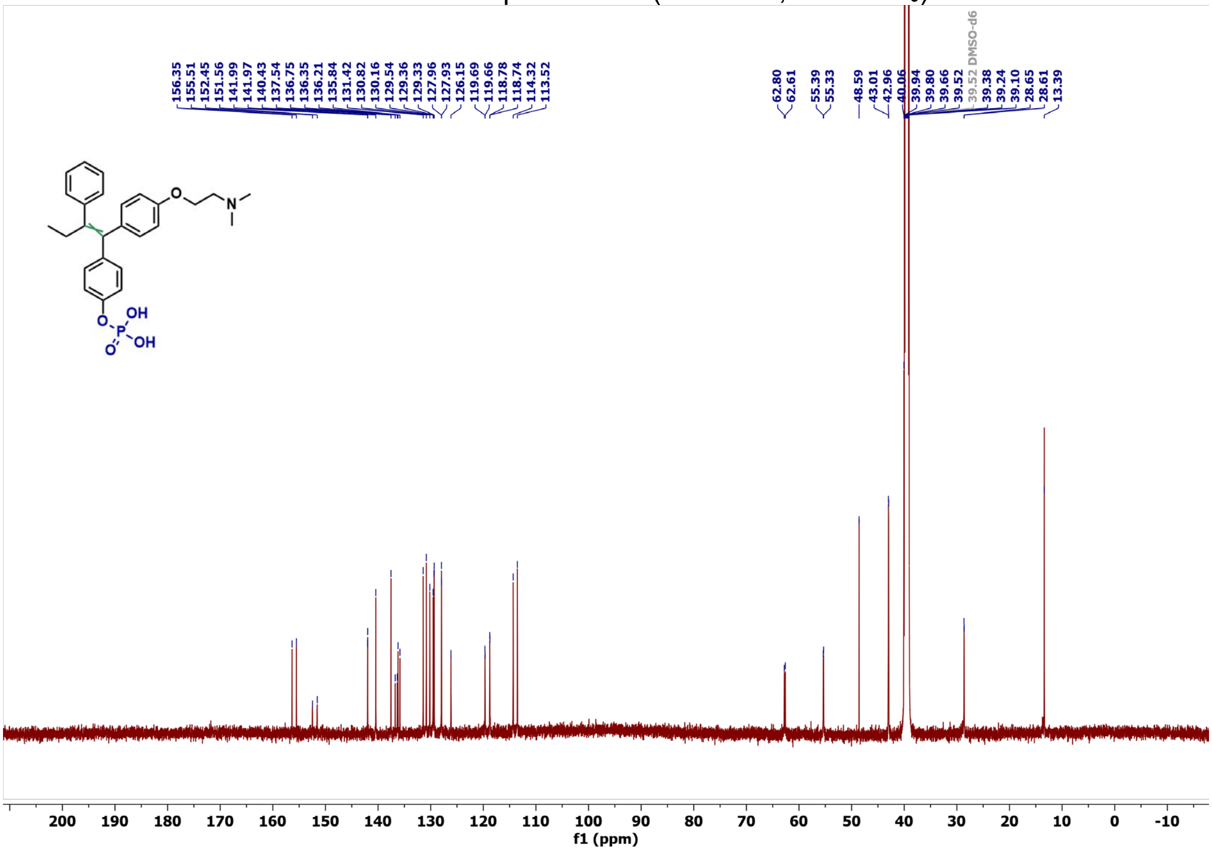

^{31}P NMR of Compound **32b** (162 MHz, DMSO-d_6)

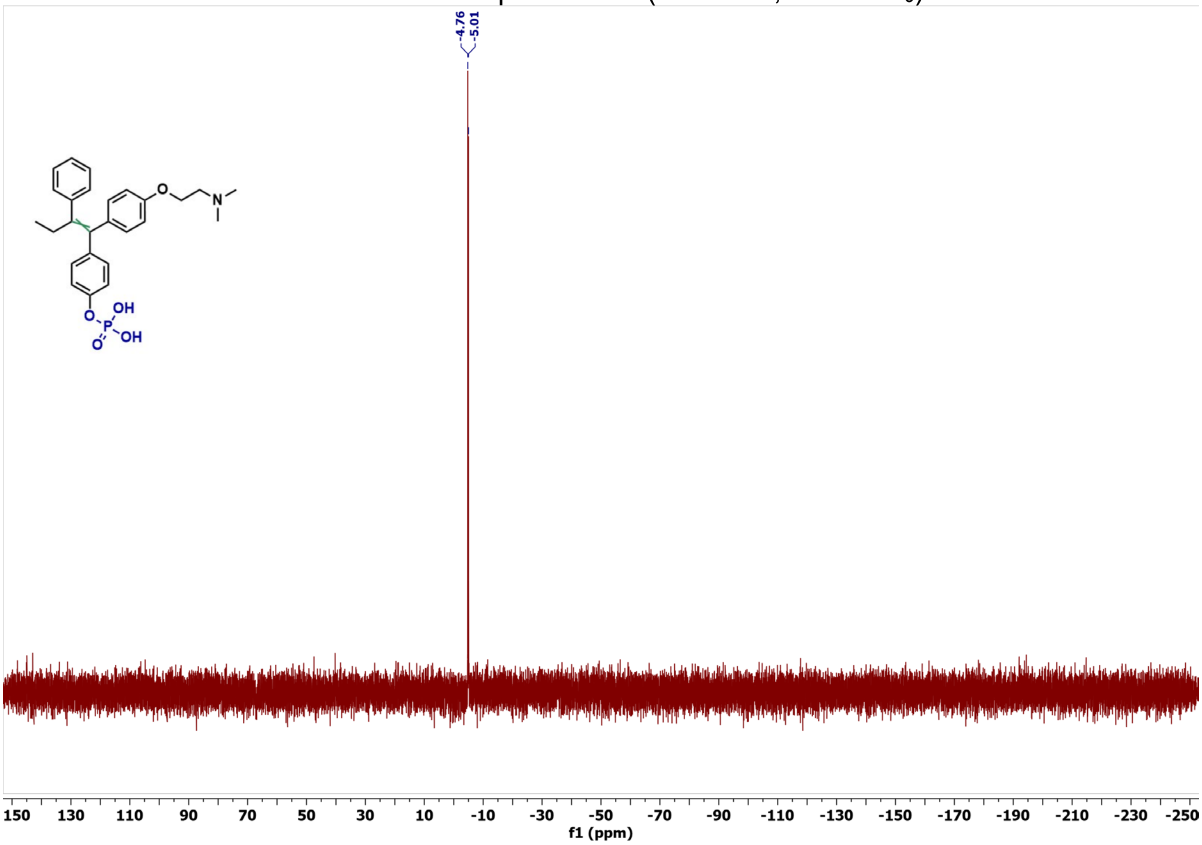

^{1}H NMR of Compound **32c** (400 MHz, MeOD)

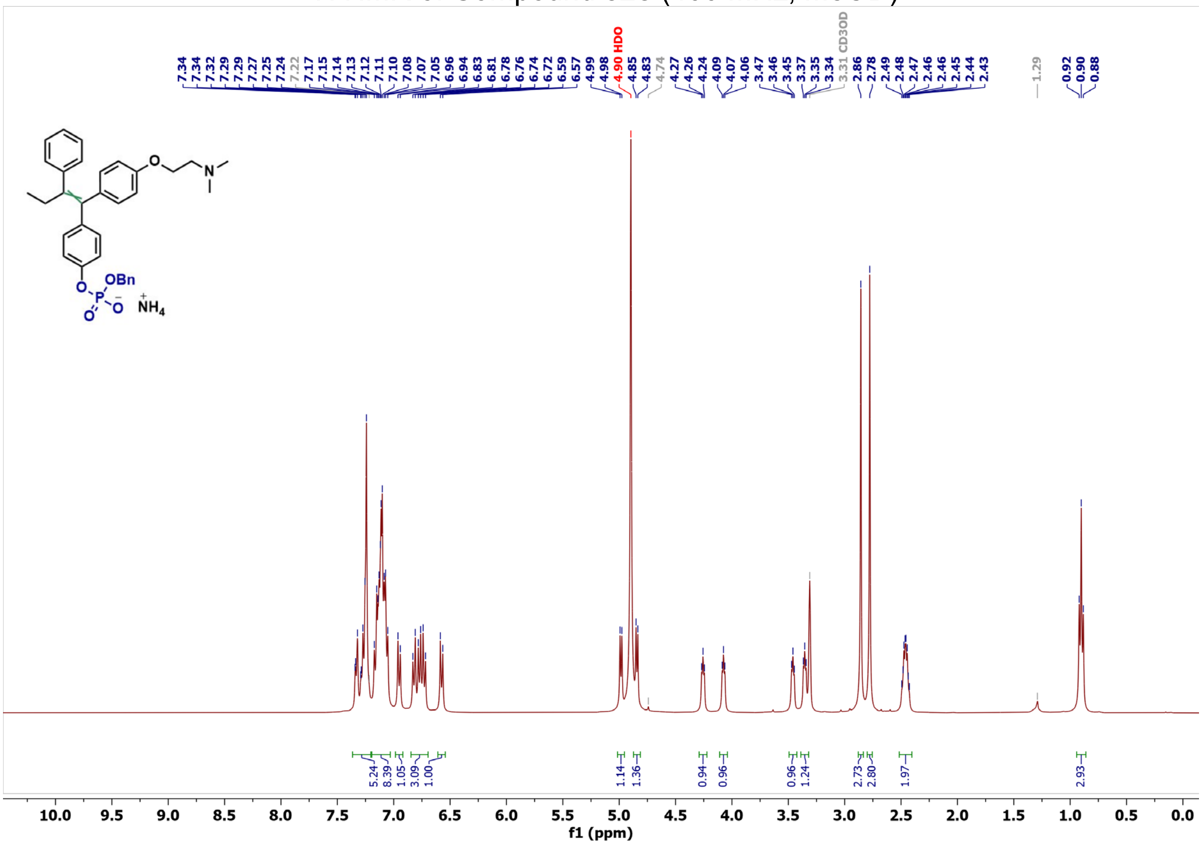

^{13}C NMR of Compound **32c** (101 MHz, MeOD)

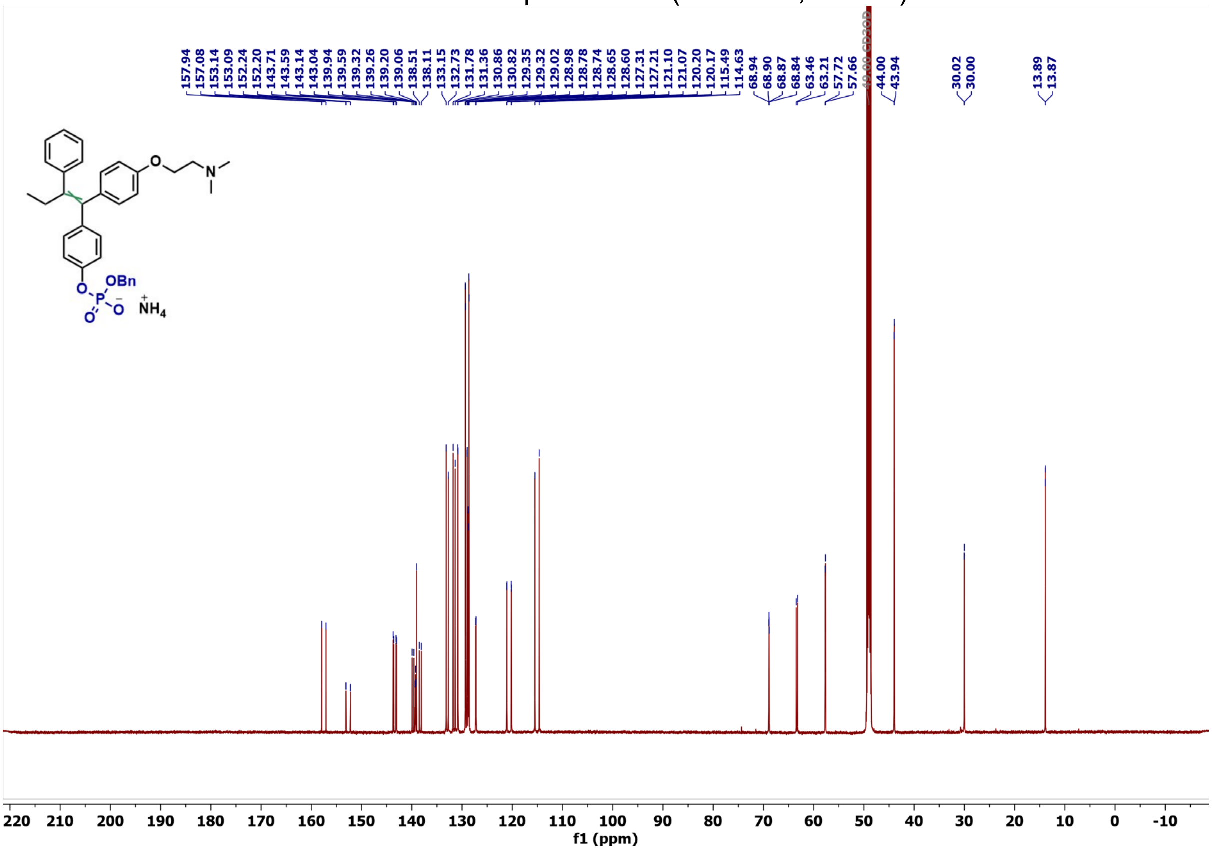

^{31}P NMR of Compound **32c** (162 MHz, MeOD)

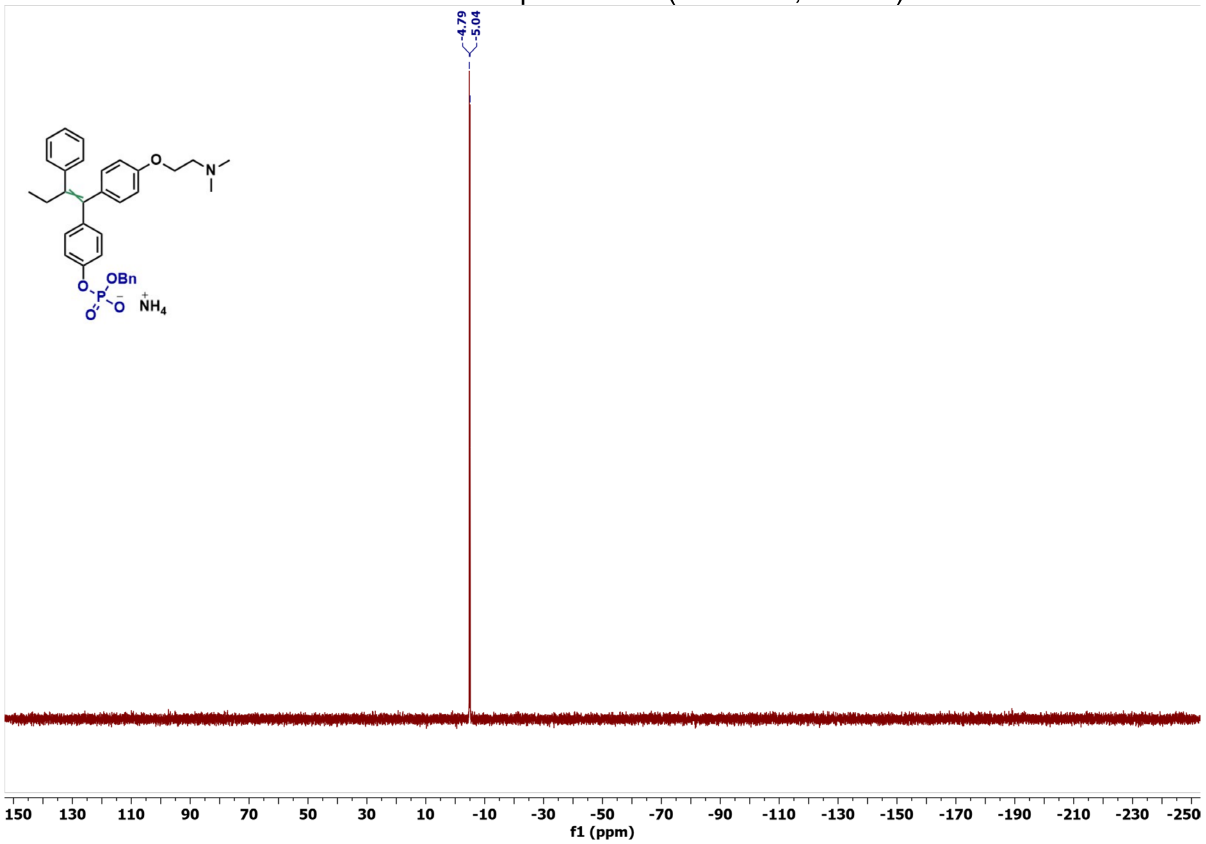

^{1}H NMR of Compound **33a** (400 MHz, $CDCl_3$)

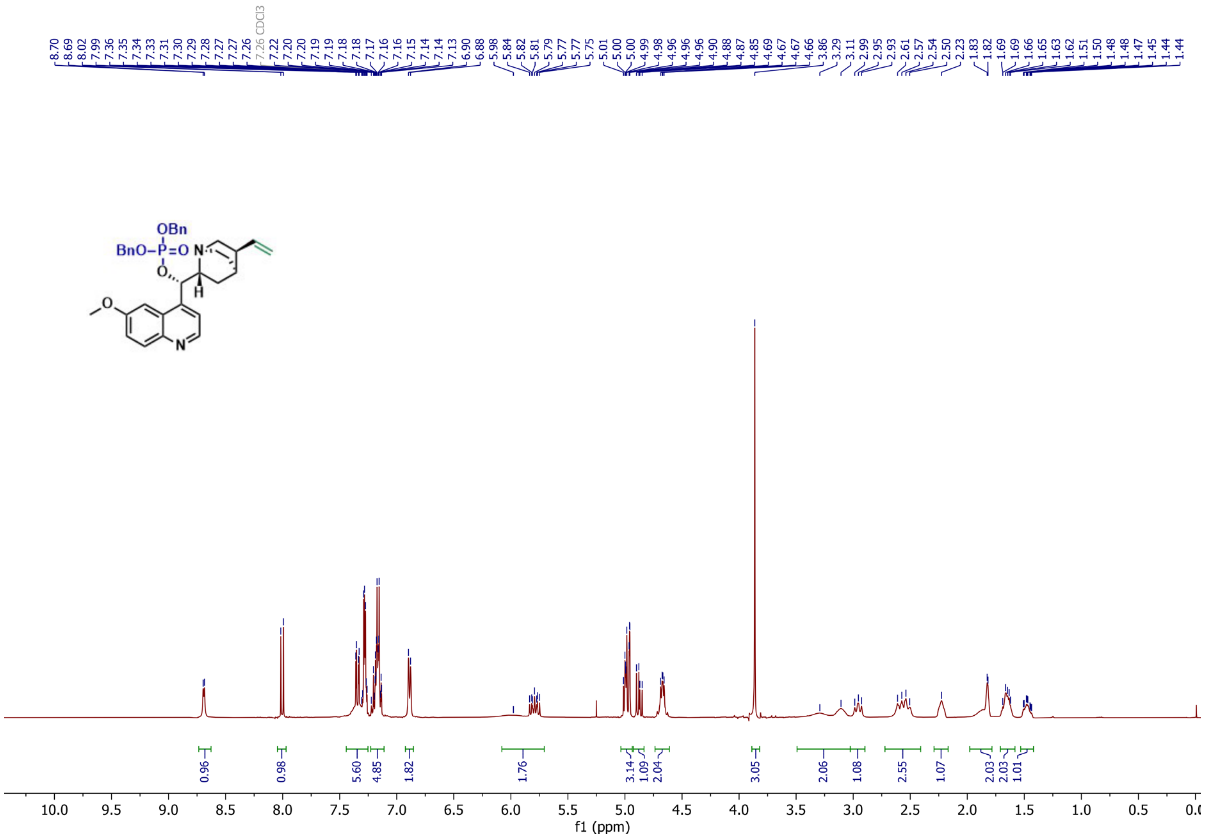

^{13}C NMR of Compound **33a** (101 MHz, $CDCl_3$)

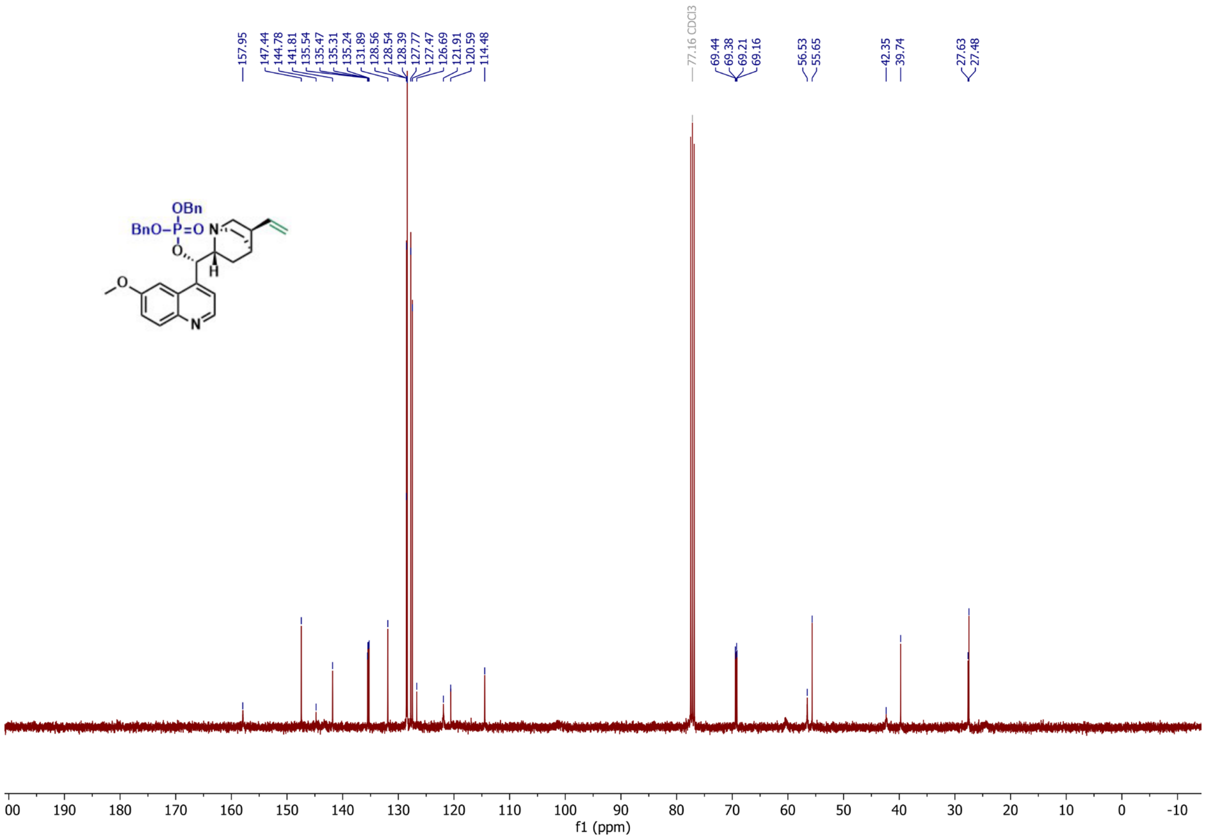

^{31}P NMR of Compound **33a** (243 MHz, $CDCl_3$)

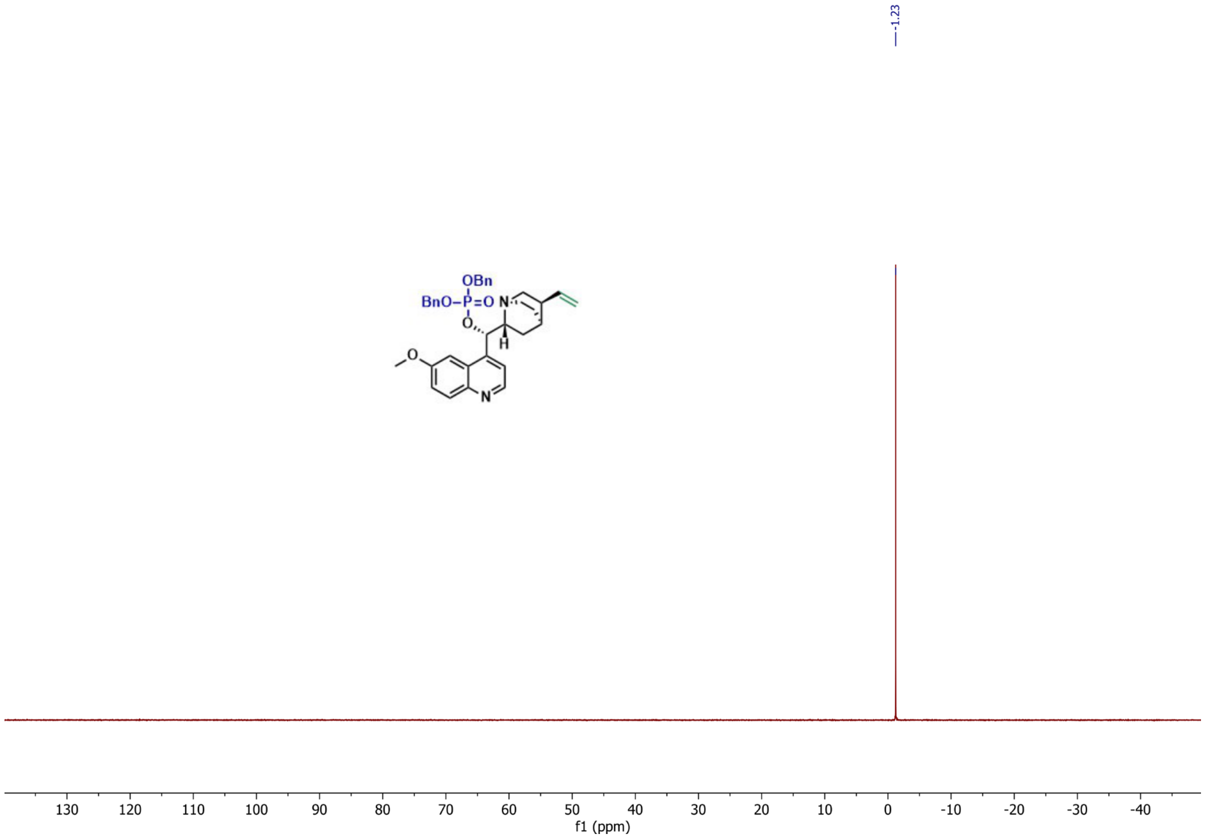

^{1}H NMR of Compound **33b** (400 MHz, $CDCl_3$)

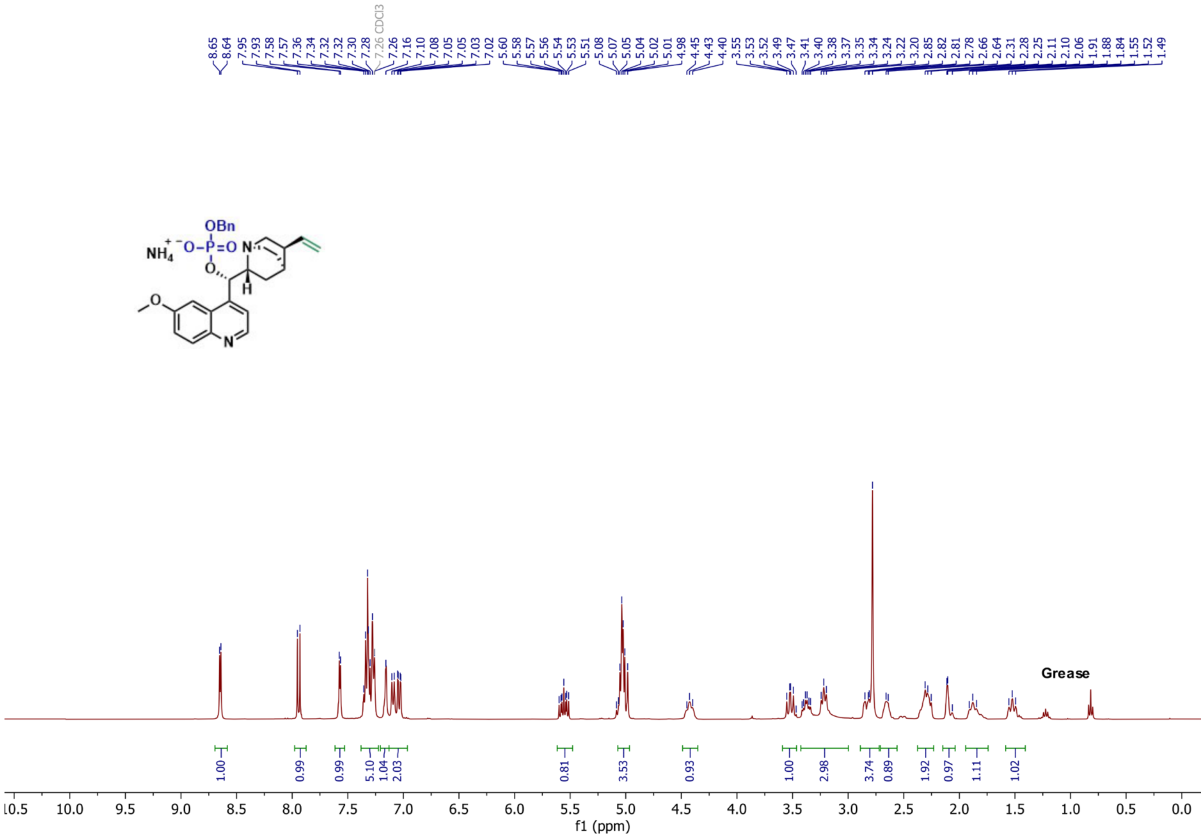

^{13}C NMR of Compound **33b** (101 MHz, $CDCl_3$)

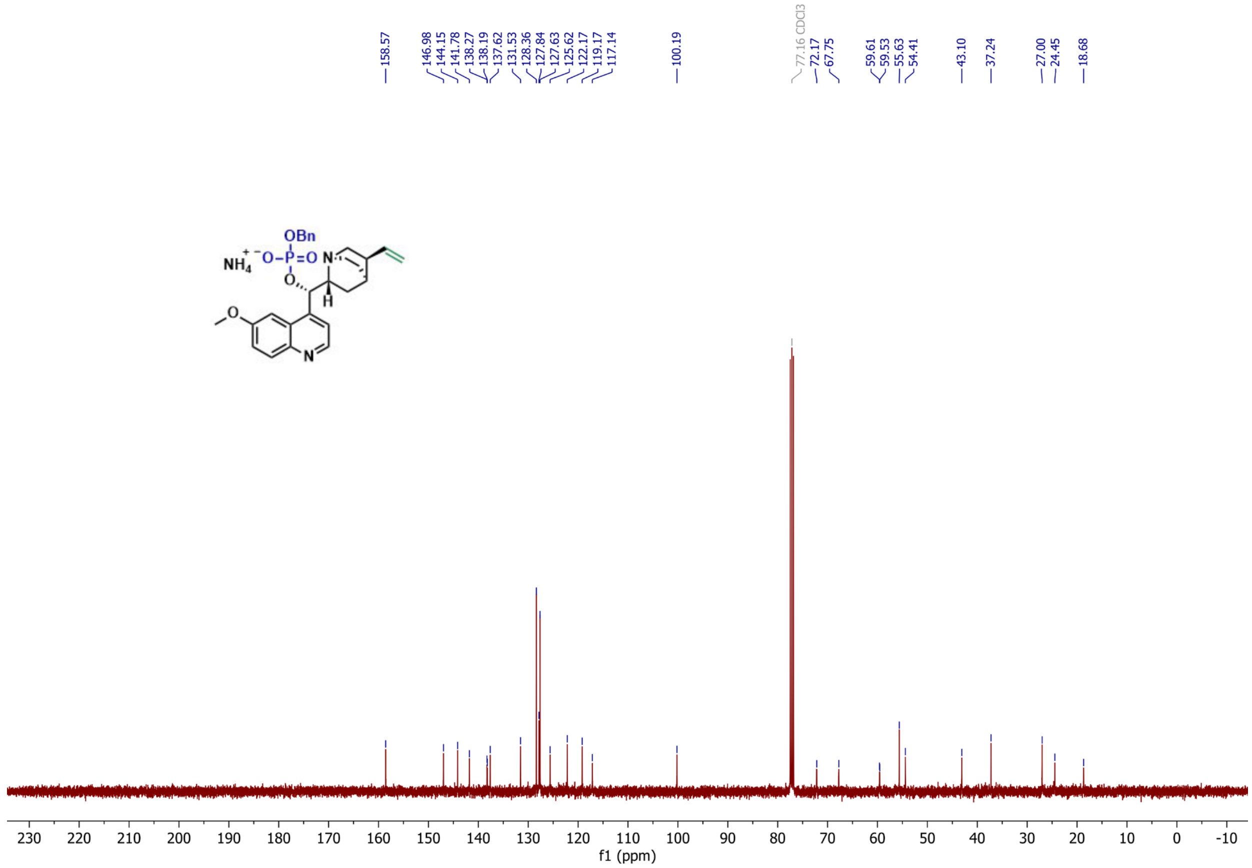

^{31}P NMR of Compound **33b** (162 MHz, $CDCl_3$)

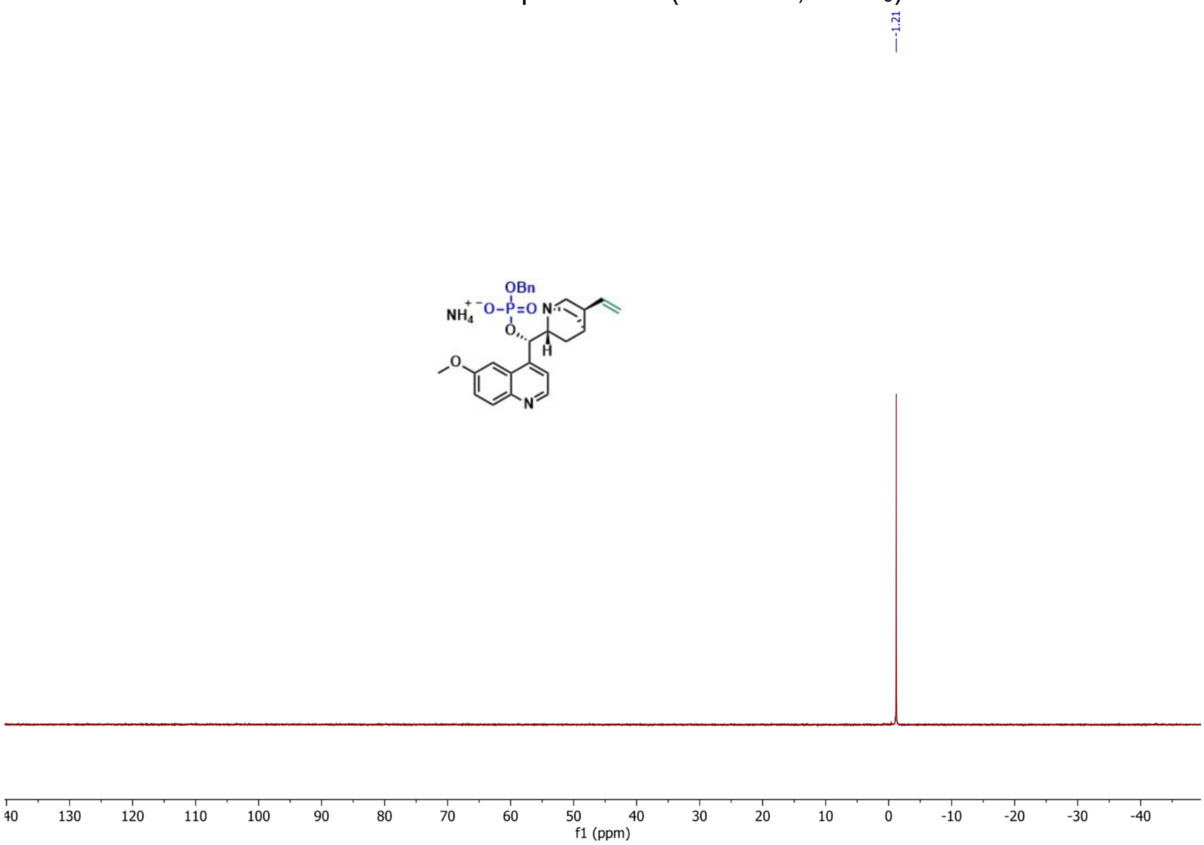

^{1}H NMR of Compound **33c** (400 MHz, $CDCl_3$)

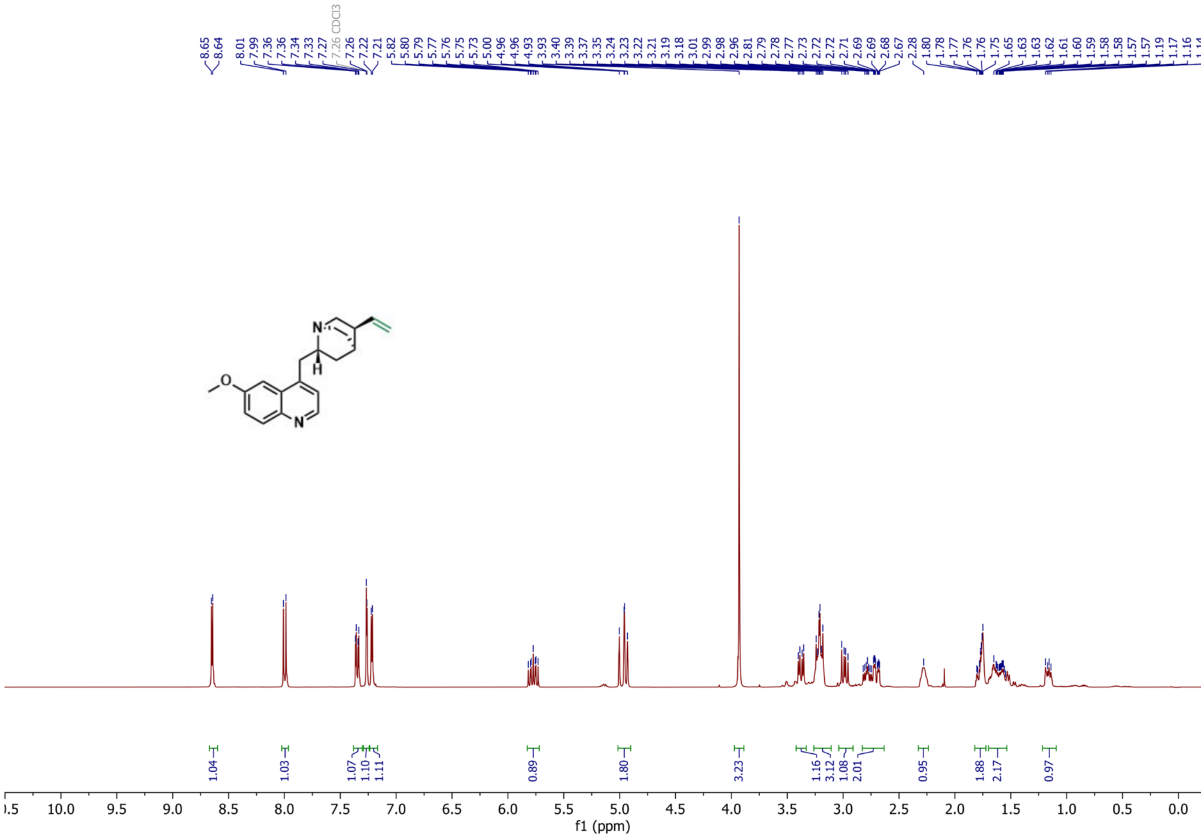

^{13}C NMR of Compound **33c** (101 MHz, $CDCl_3$)

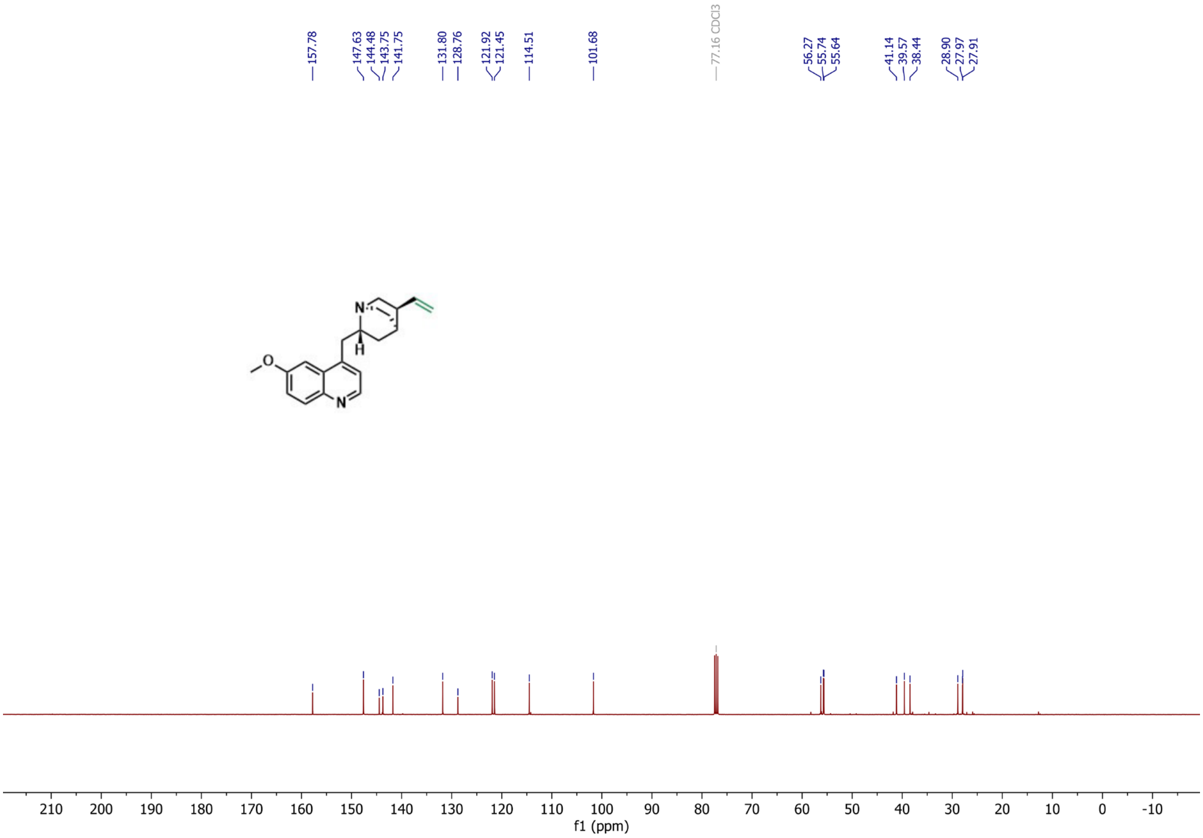

^{1}H NMR of Compound **35a** (600 MHz, $CDCl_3$)

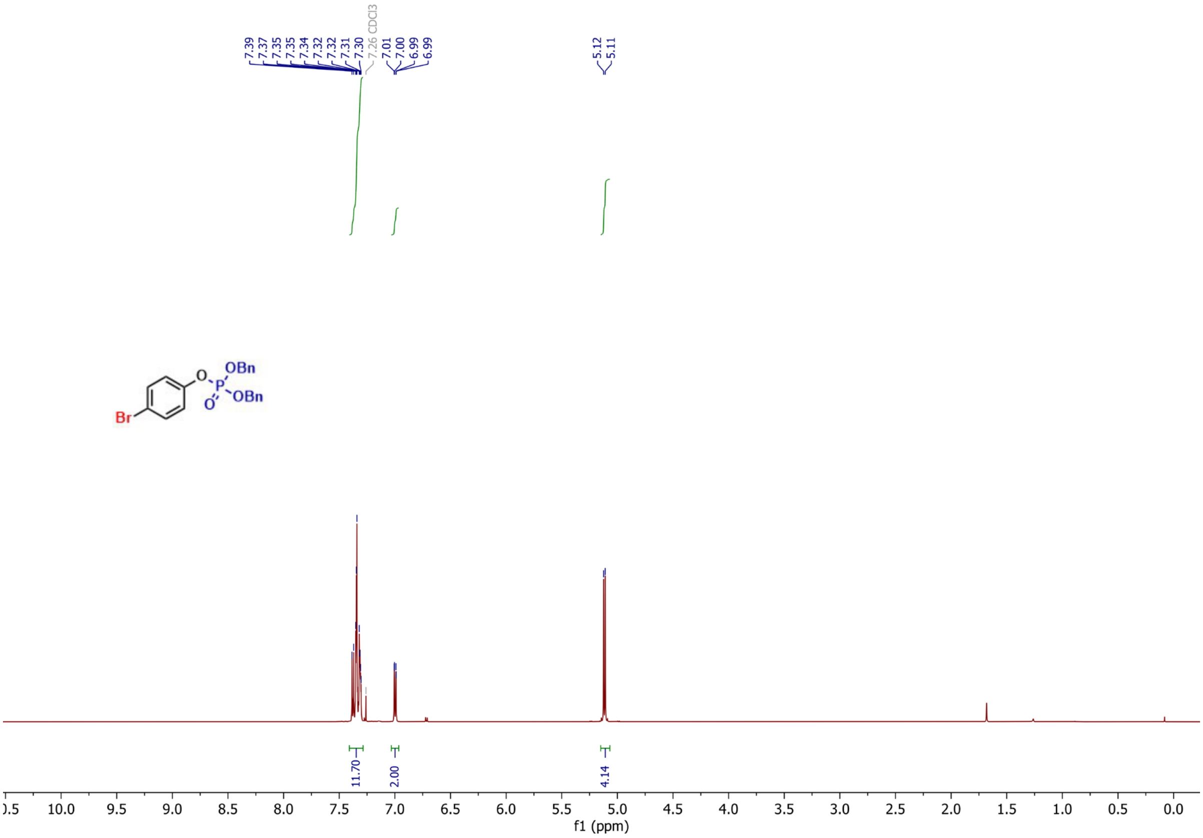

^{13}C NMR of Compound **35a** (151 MHz, $CDCl_3$)

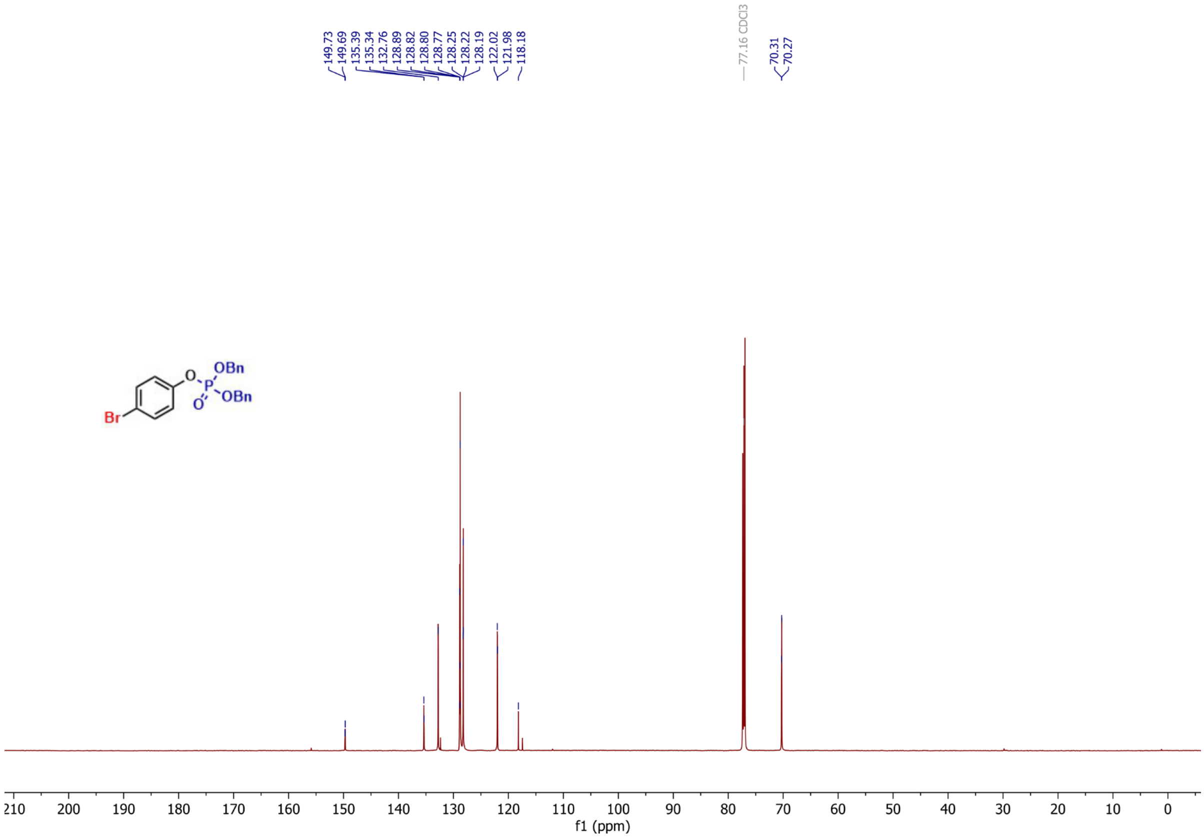

^{31}P NMR of Compound **35a** (243 MHz, $CDCl_3$)

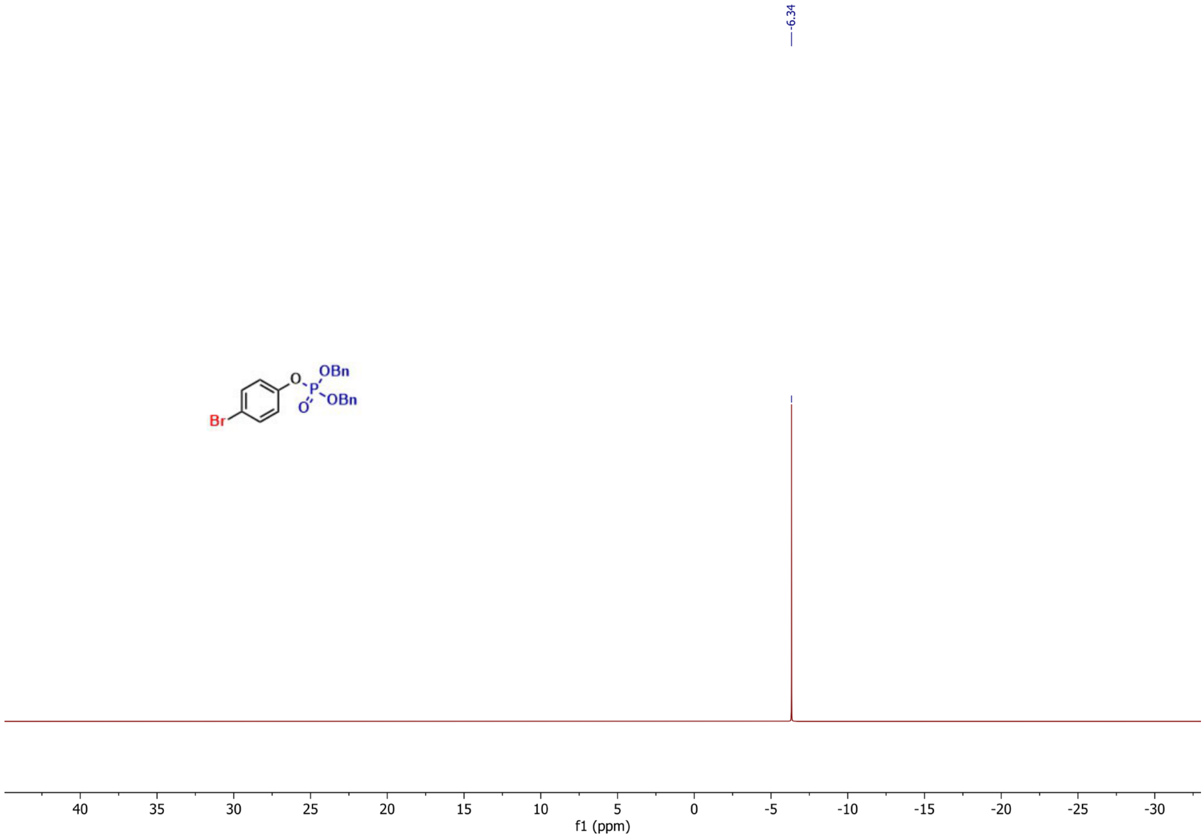

^{1}H NMR of Compound **36a** (600 MHz, $CDCl_3$)

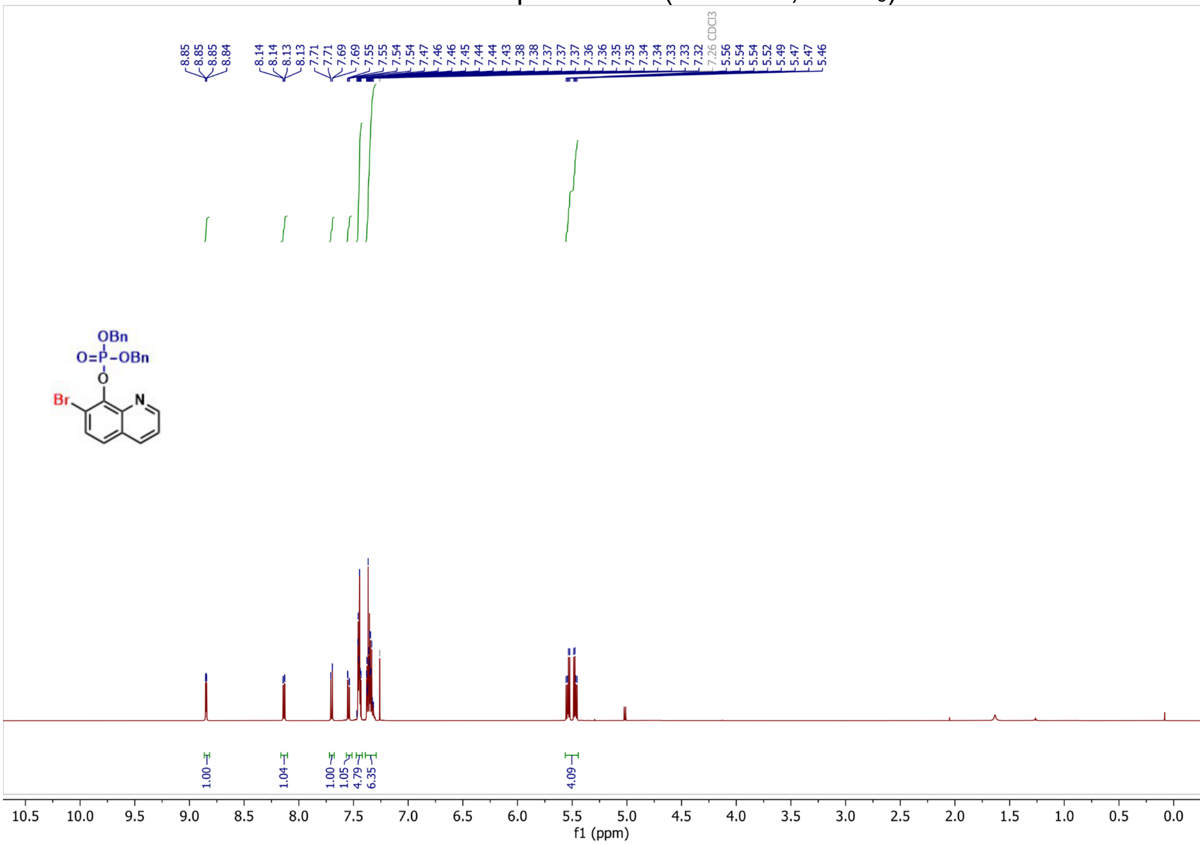

^{13}C NMR of Compound **36a** (151 MHz, $CDCl_3$)

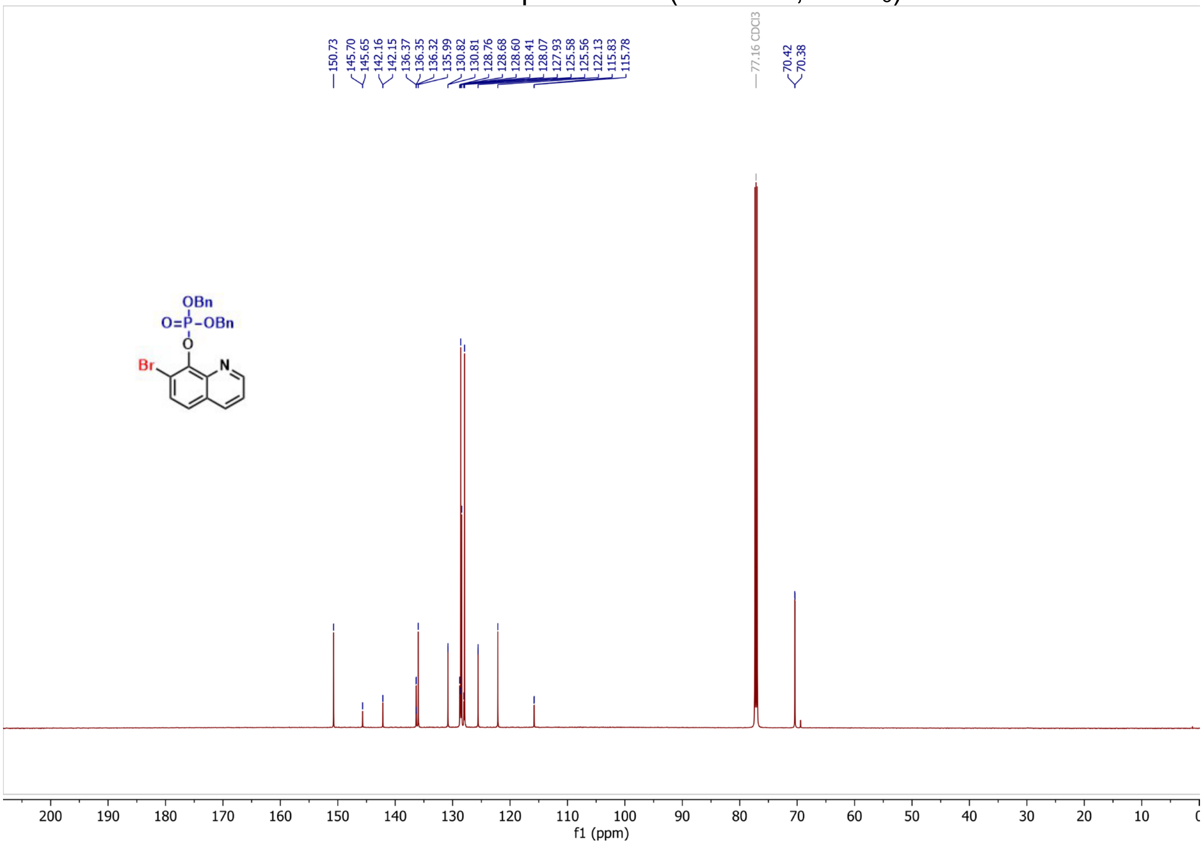

^{31}P NMR of Compound **36a** (243 MHz, $CDCl_3$)

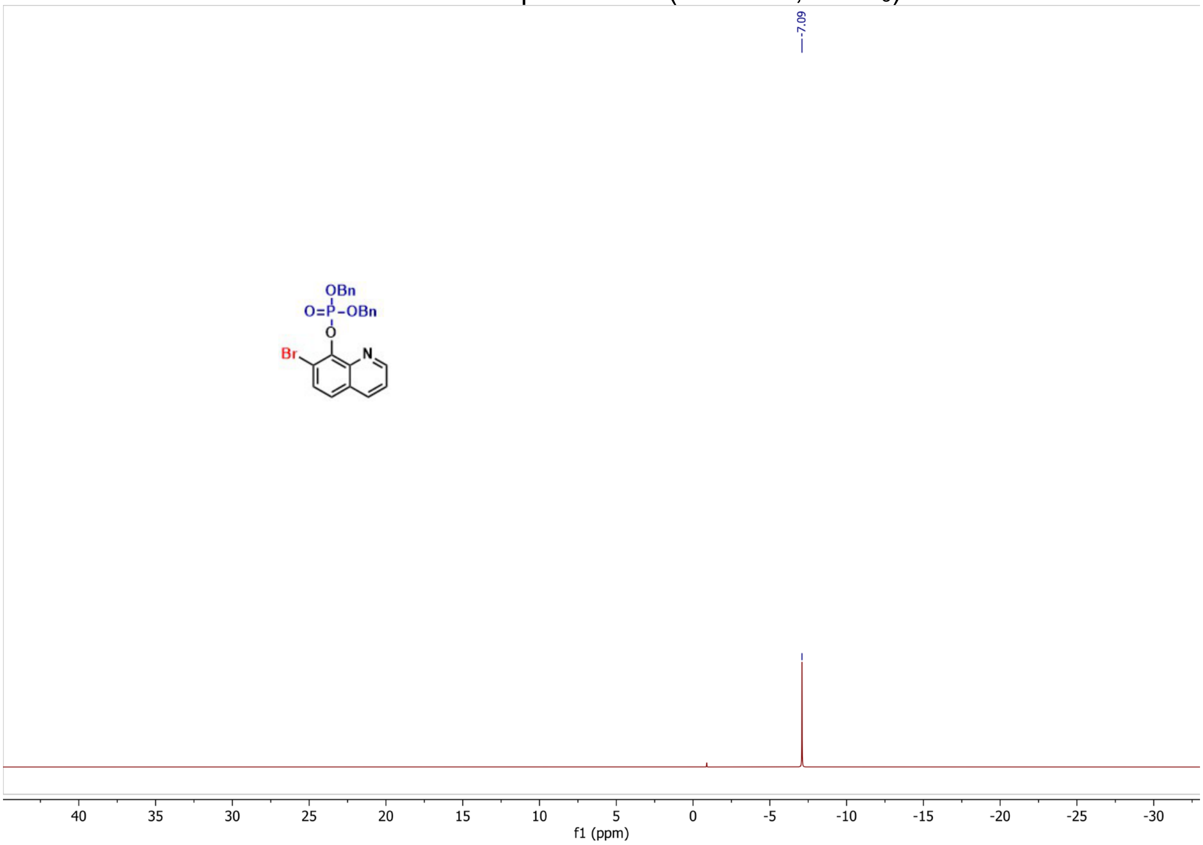

^{1}H NMR of Compound **37a** (600 MHz, $CDCl_3$)

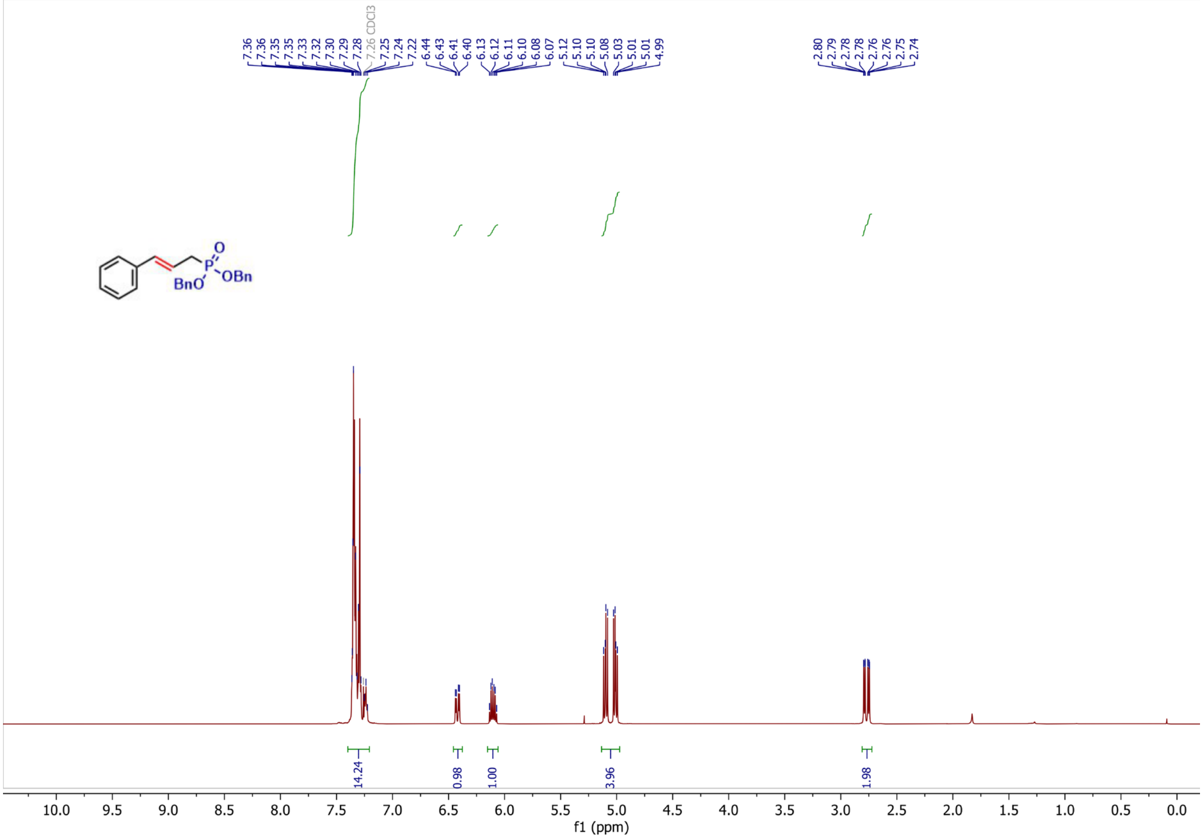

^{13}C NMR of Compound **37a** (151 MHz, $CDCl_3$)

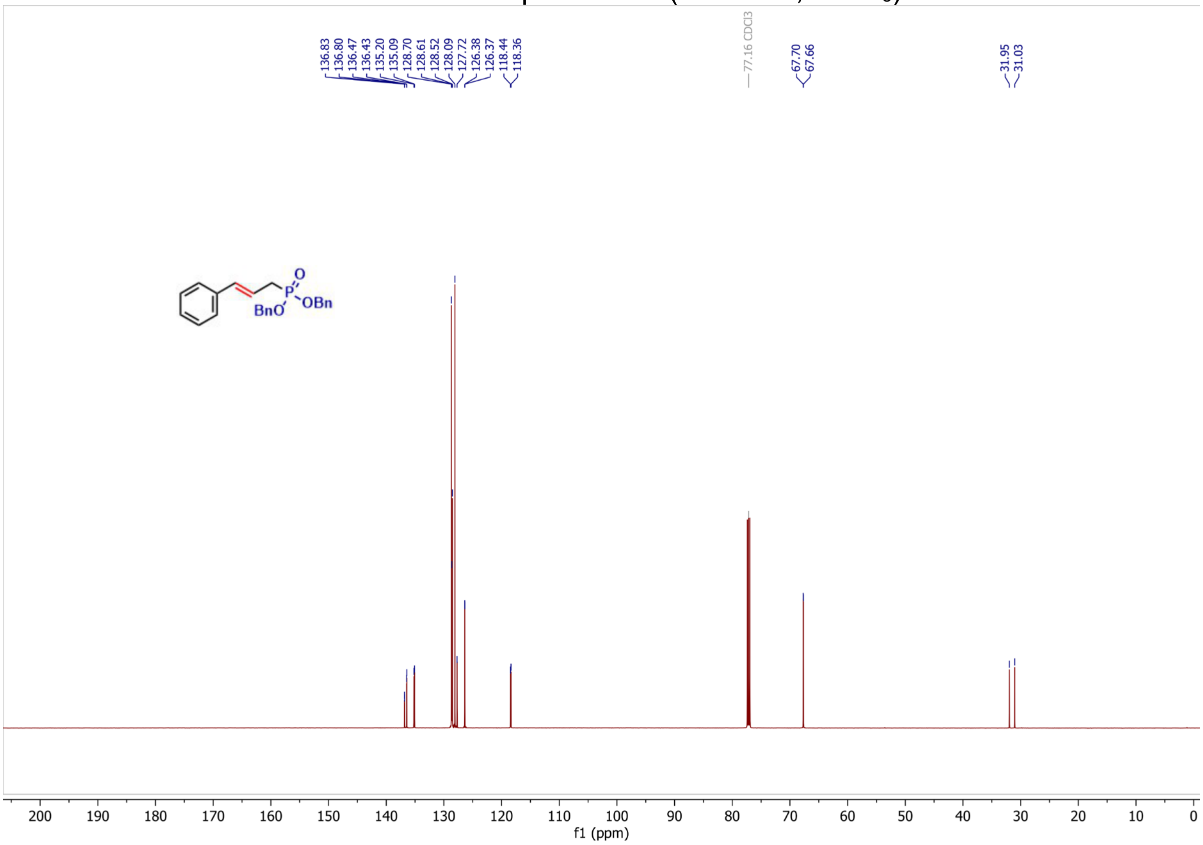

^{31}P NMR of Compound **37a** (243 MHz, $CDCl_3$)

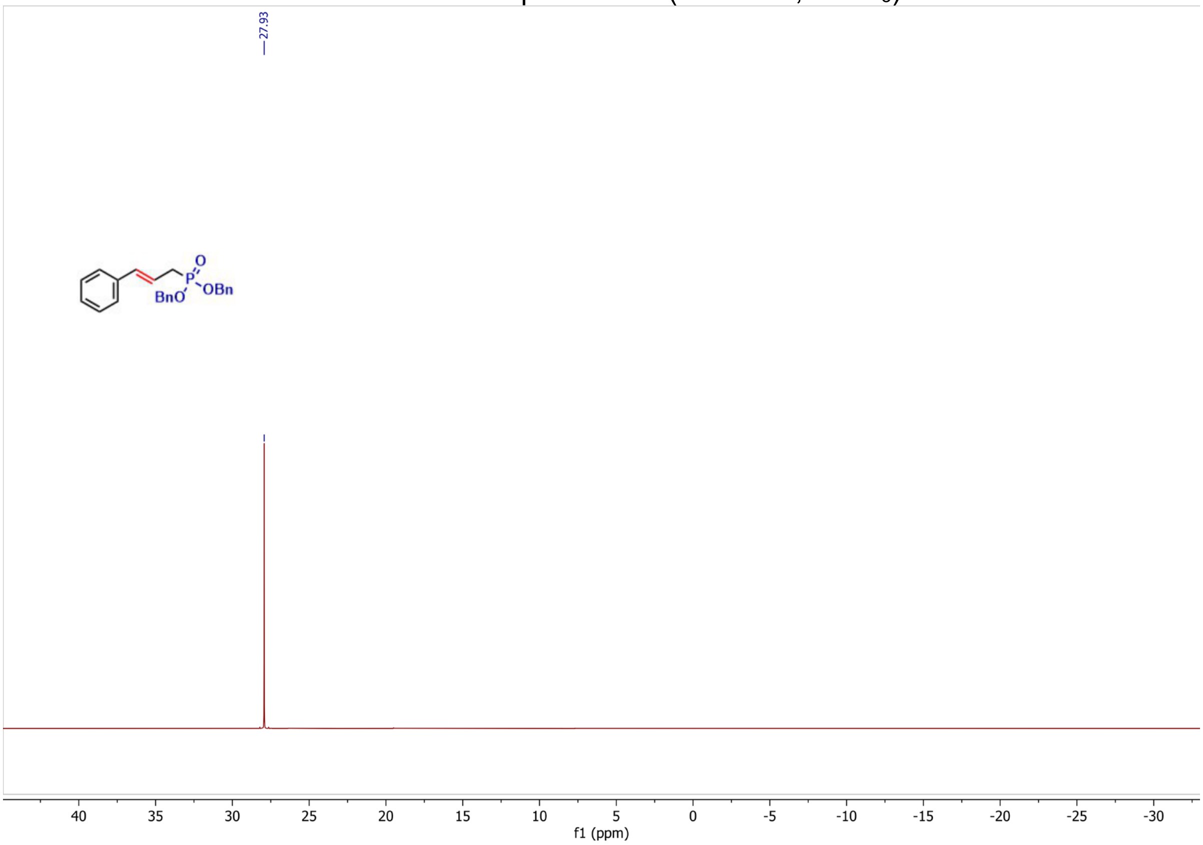

^{1}H NMR of Compound **38a** (600 MHz, $CDCl_3$)

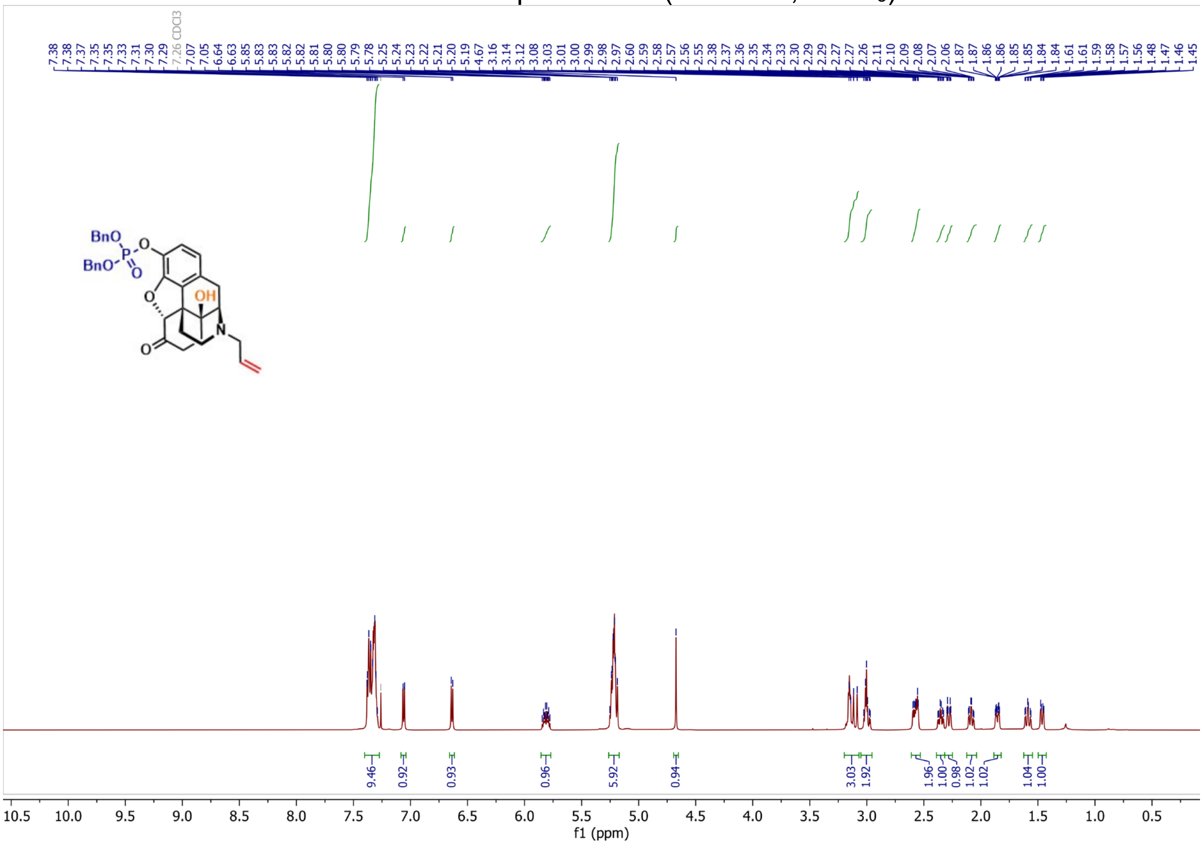

^{13}C NMR of Compound **38a** (151 MHz, $CDCl_3$)

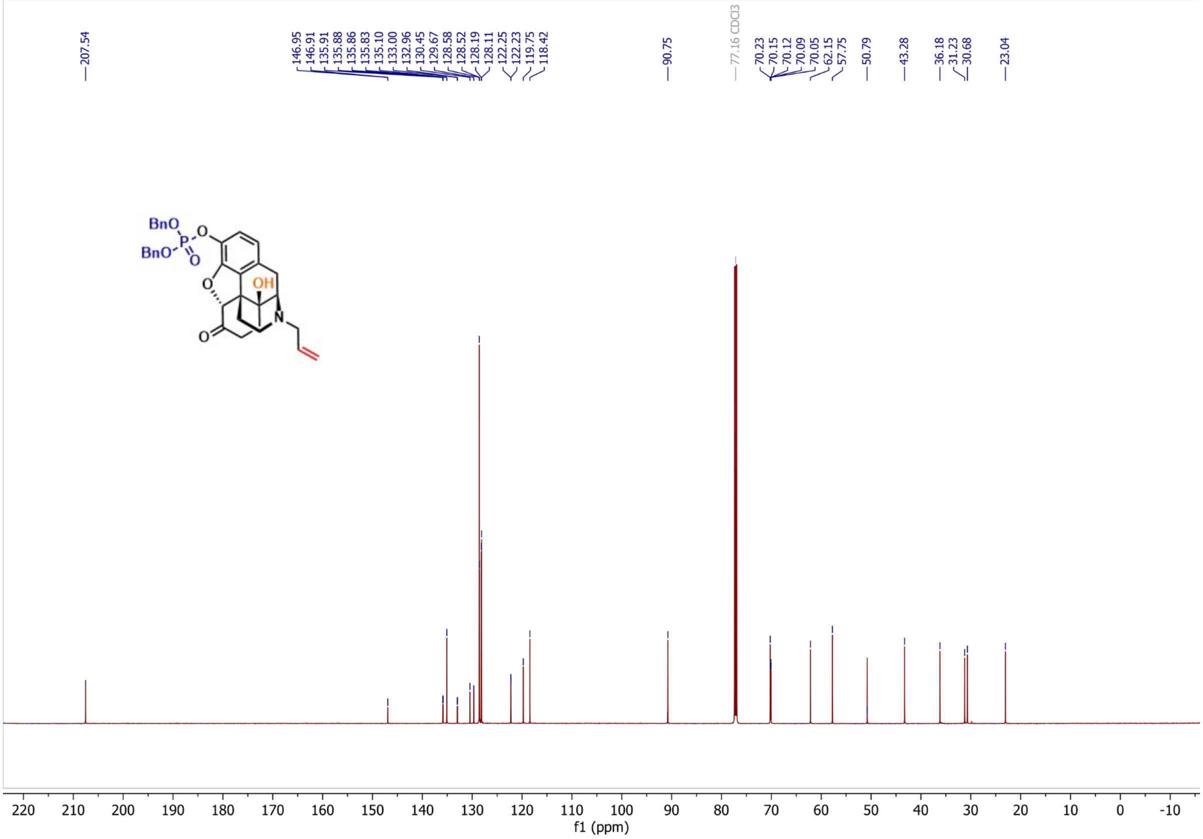

^{31}P NMR of Compound **38a** (243 MHz, $CDCl_3$)

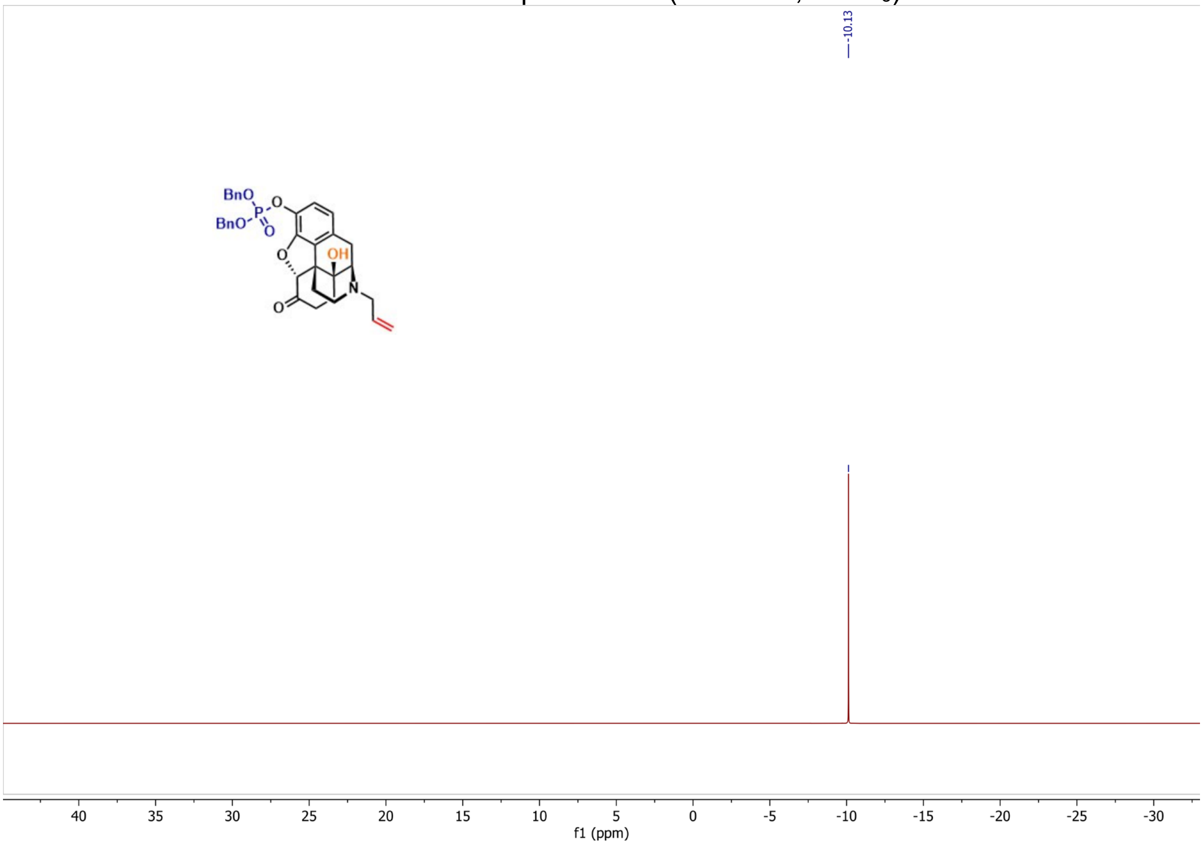

^{1}H NMR of Compound **S3** (600 MHz, $CDCl_3$)

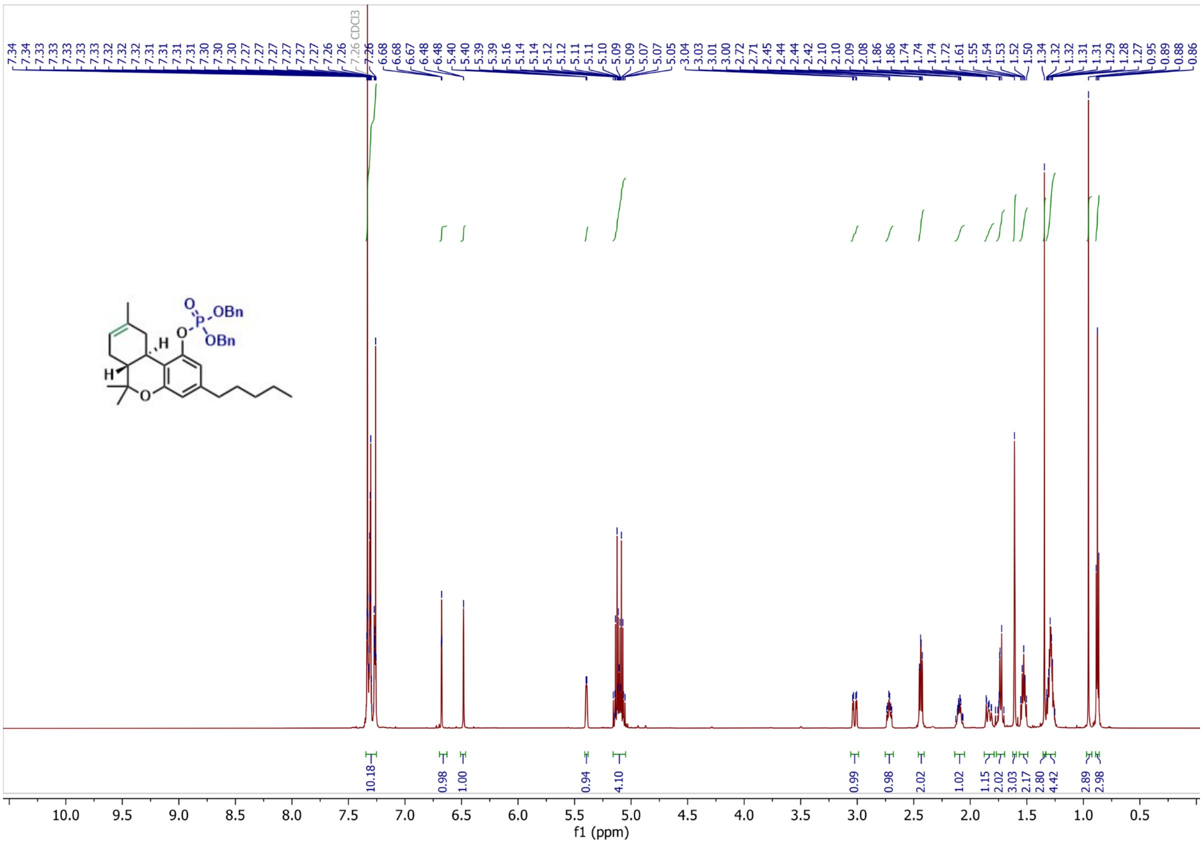

^{13}C NMR of Compound **S3** (151 MHz, $CDCl_3$)

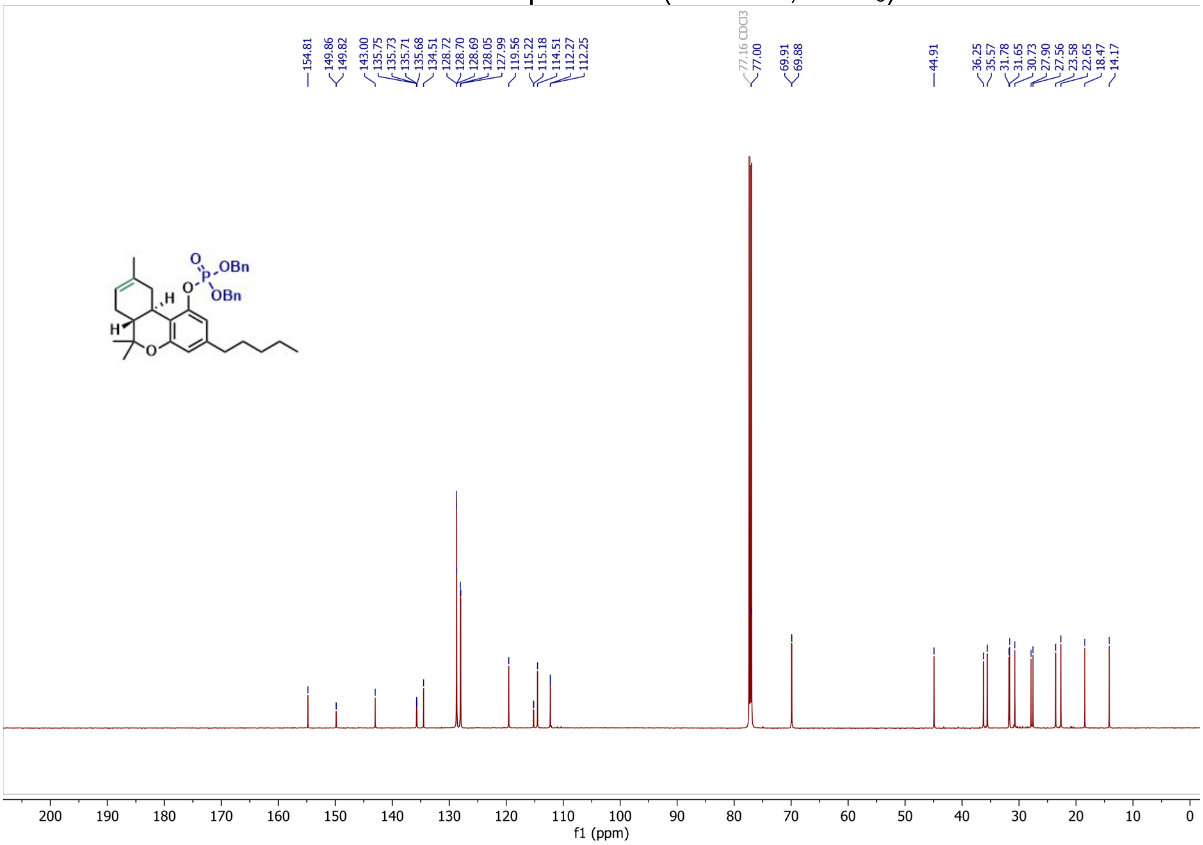

^{31}P NMR of Compound **S3** (243 MHz, $CDCl_3$)

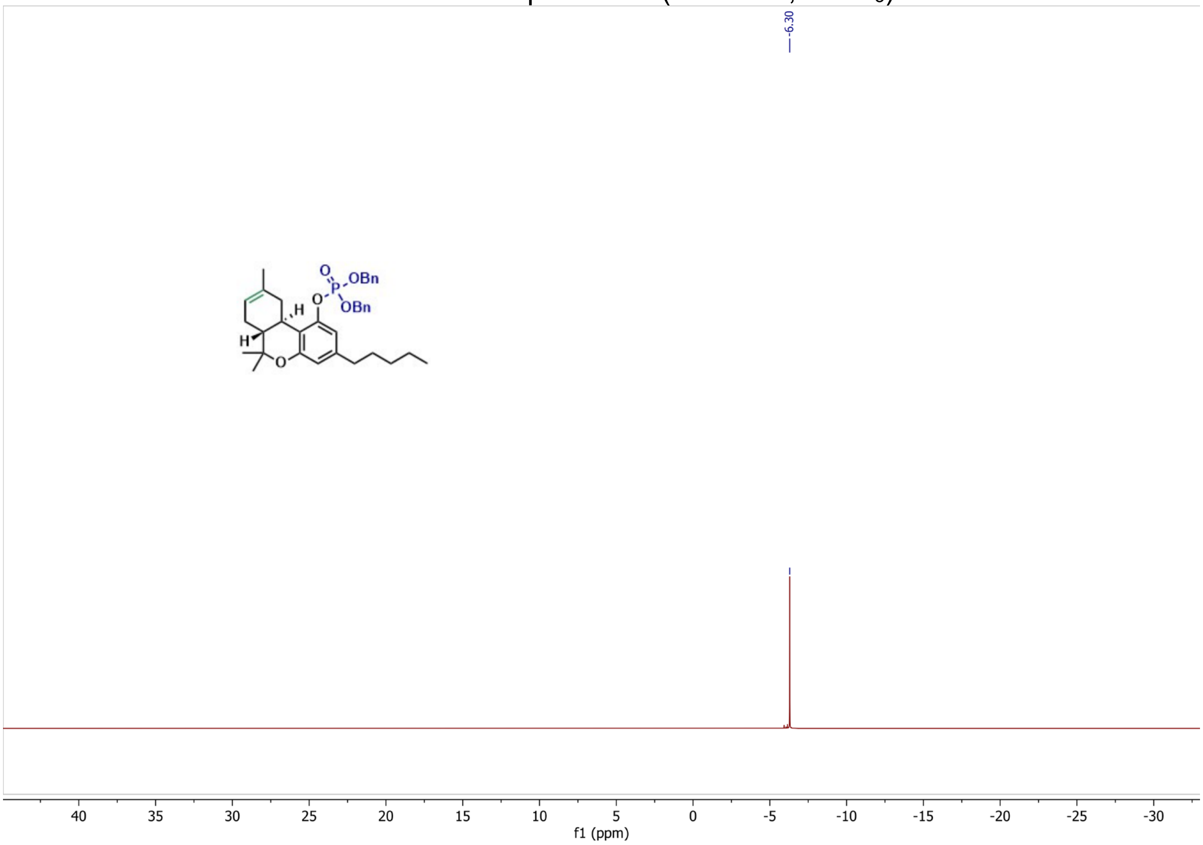

^{1}H NMR of Compound **S5** (600 MHz, $CDCl_3$)

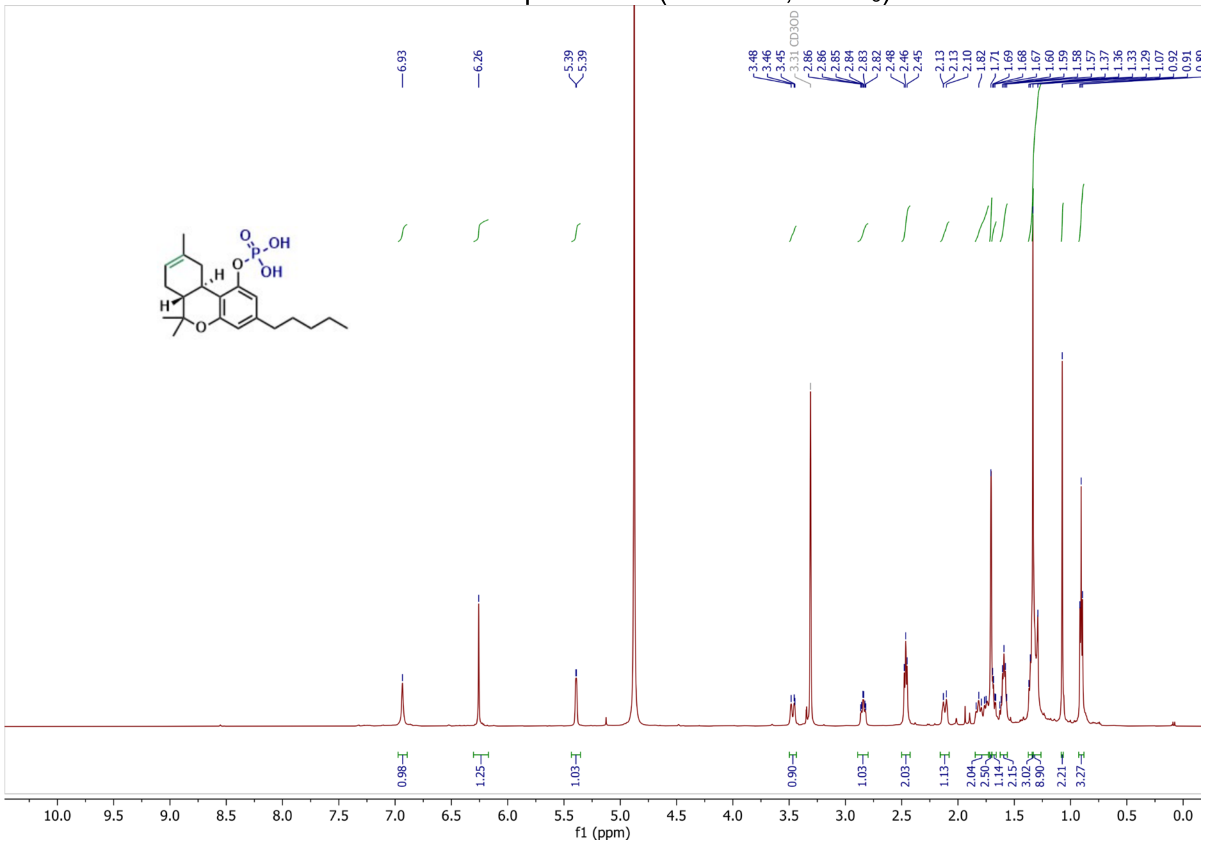

^{13}C NMR of Compound **S5** (151 MHz, $CDCl_3$)

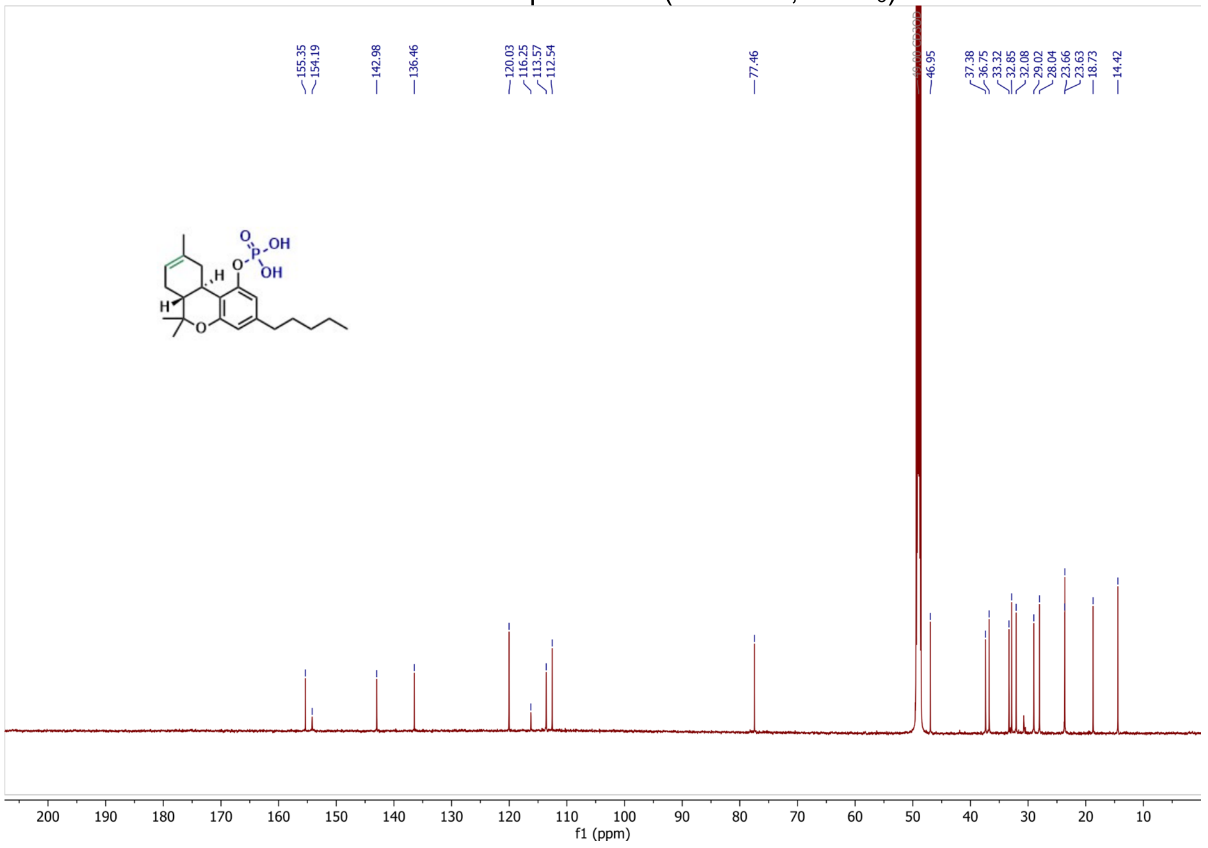

^{31}P NMR of Compound **S5** (243 MHz, $CDCl_3$)

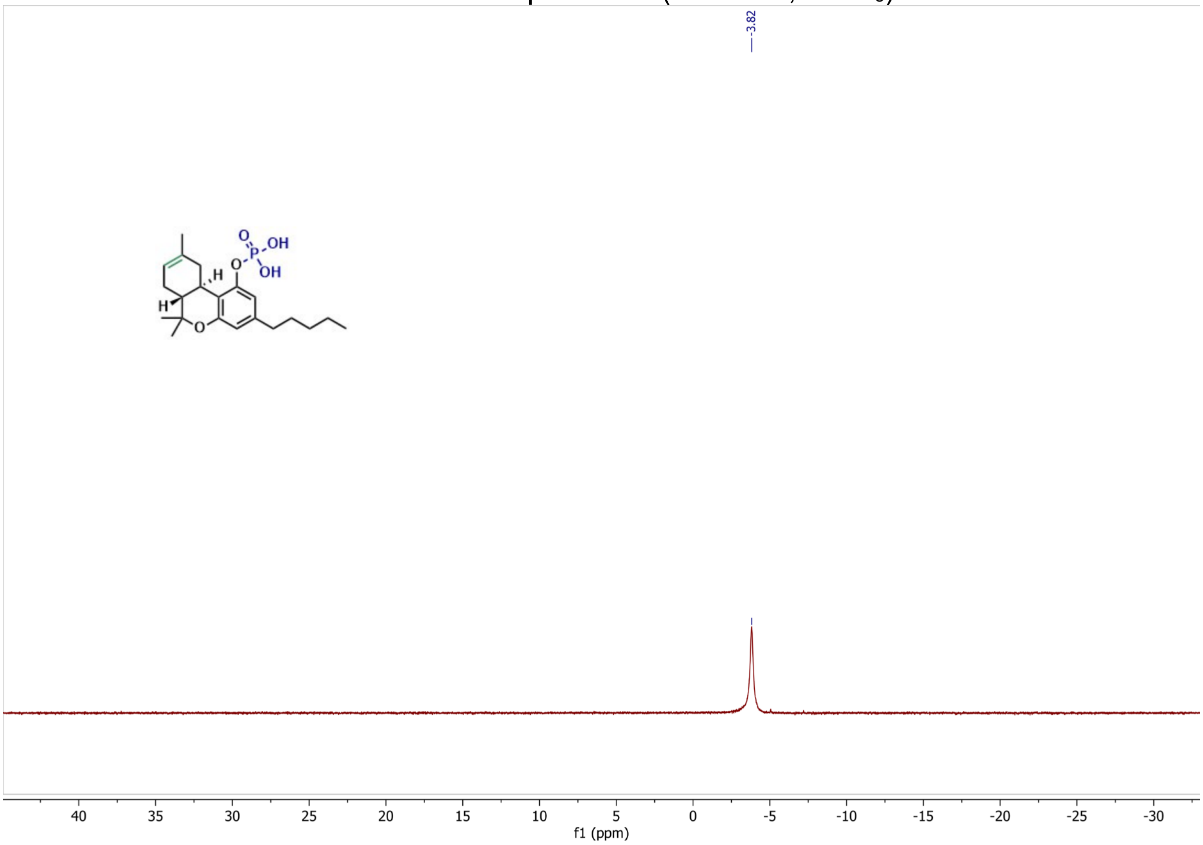

^{1}H NMR of Compound **S4** (600 MHz, $CDCl_3$)

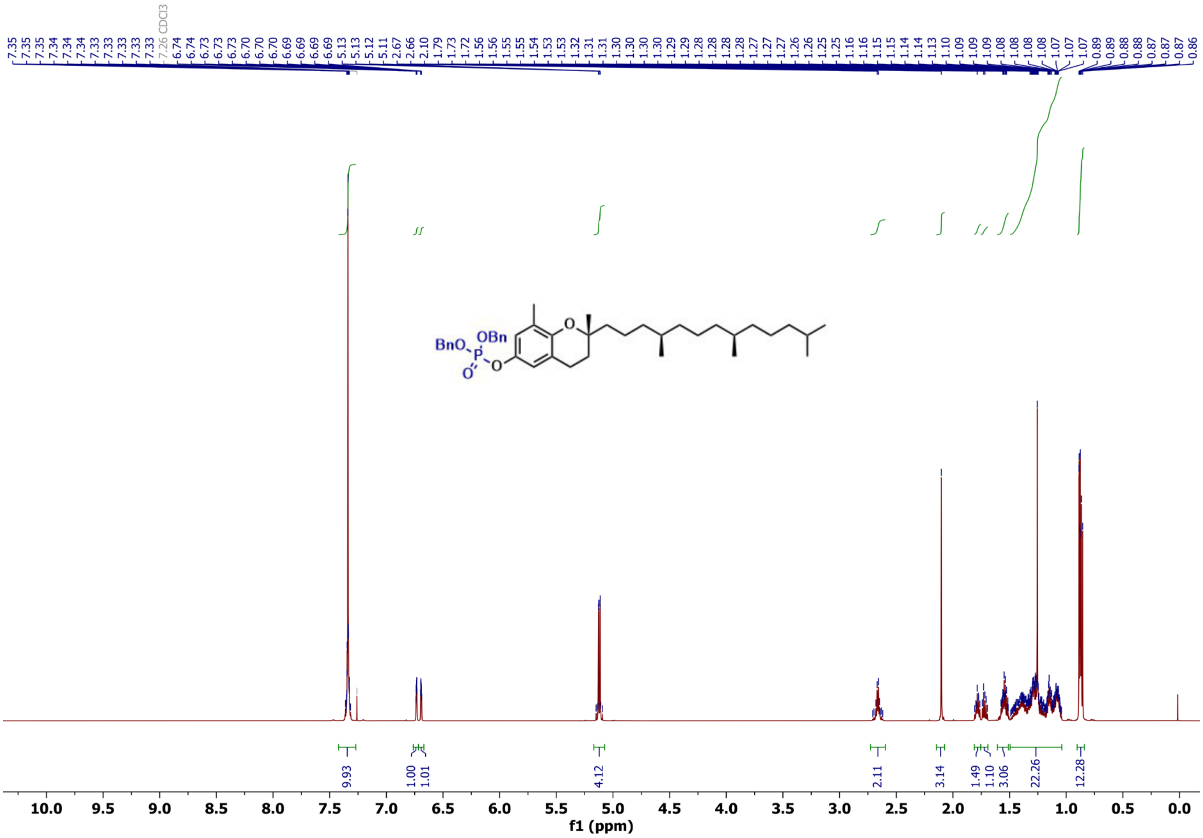

^{13}C NMR of Compound **S4** (151 MHz, $CDCl_3$)

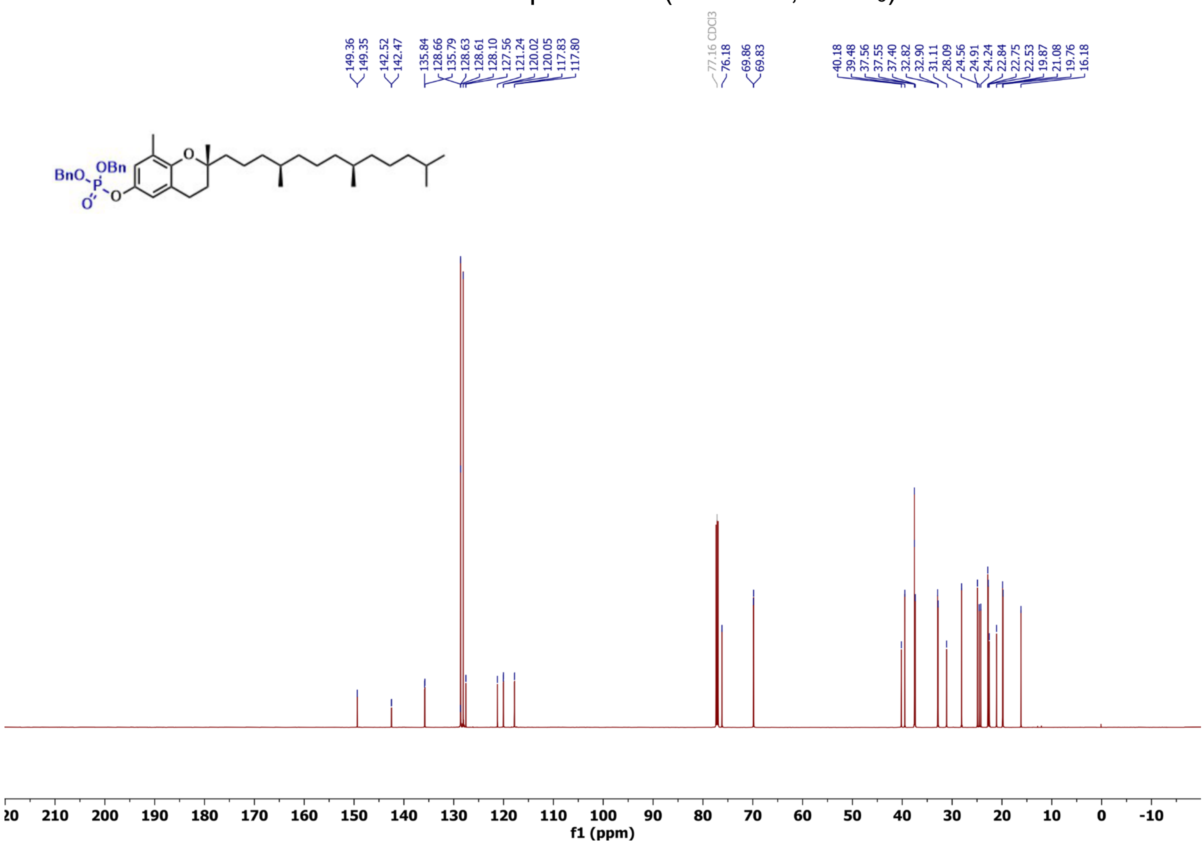

^{31}P NMR of Compound **S4** (243 MHz, $CDCl_3$)

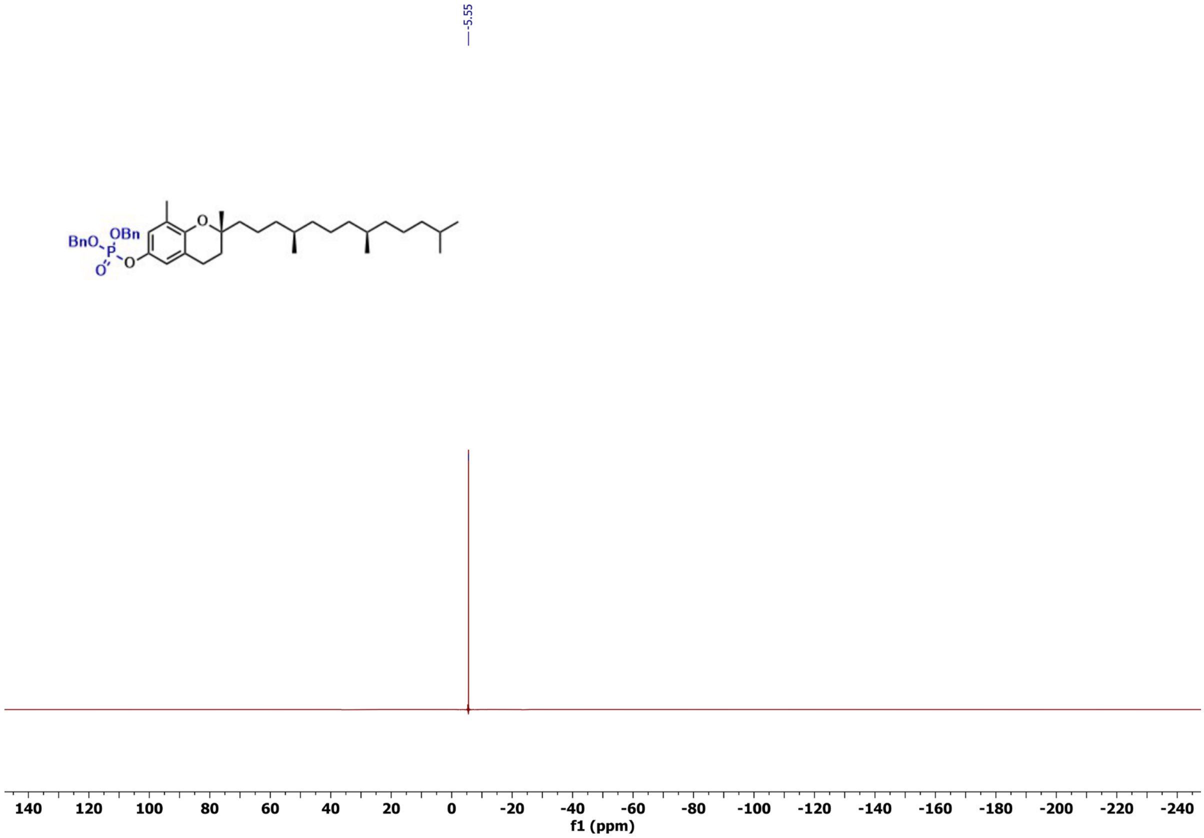

^{1}H NMR of Compound **S6** (600 MHz, $CDCl_3$)

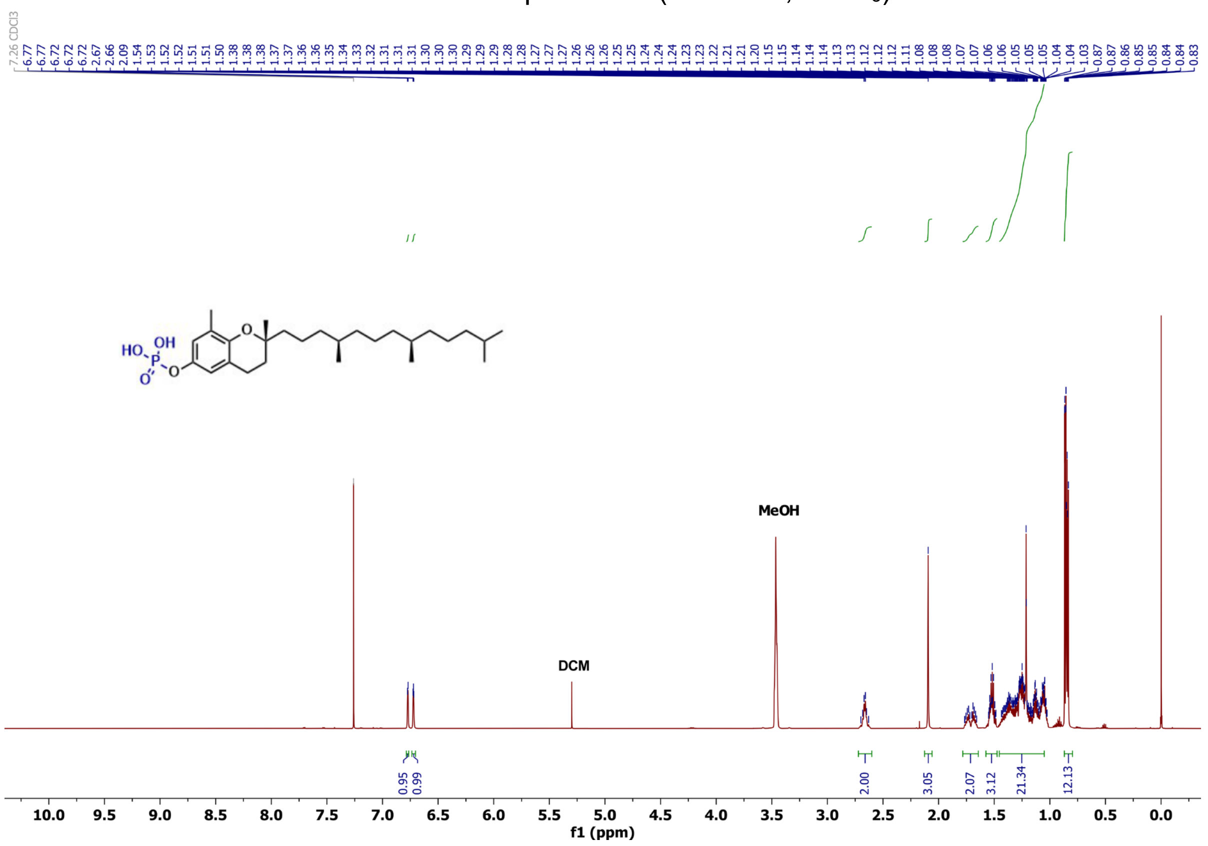

^{13}C NMR of Compound **S6** (151 MHz, $CDCl_3$)

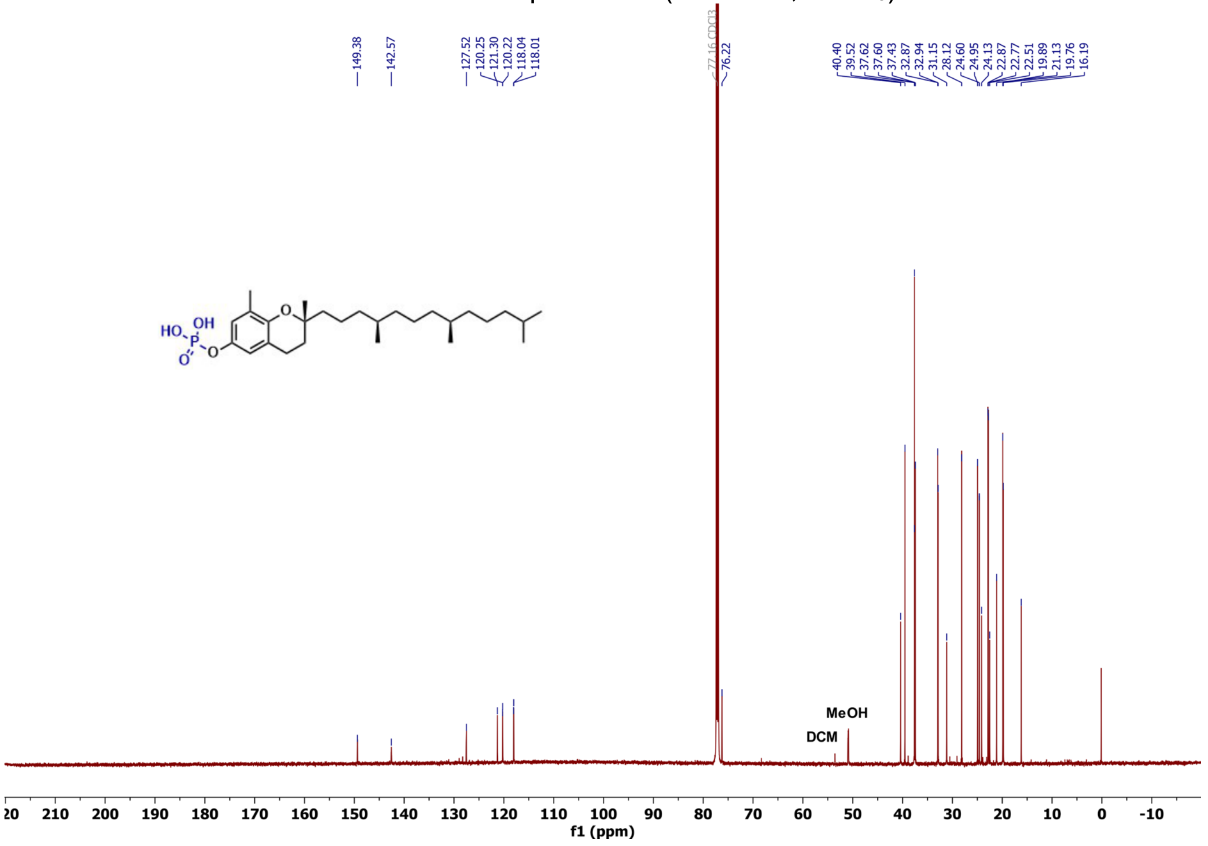

^{31}P NMR of Compound **S6** (243 MHz, $CDCl_3$)

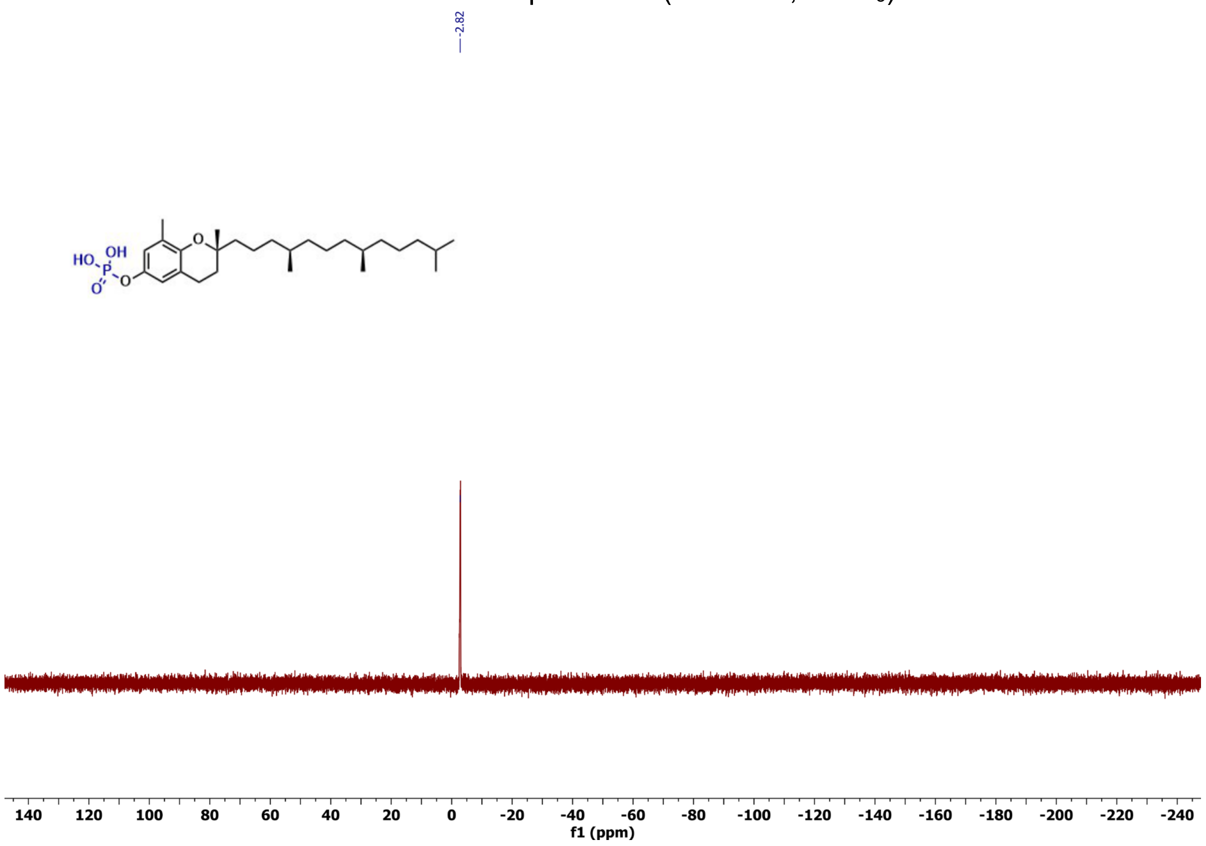

^{1}H NMR of Compound **40** (600 MHz, $CDCl_3$)

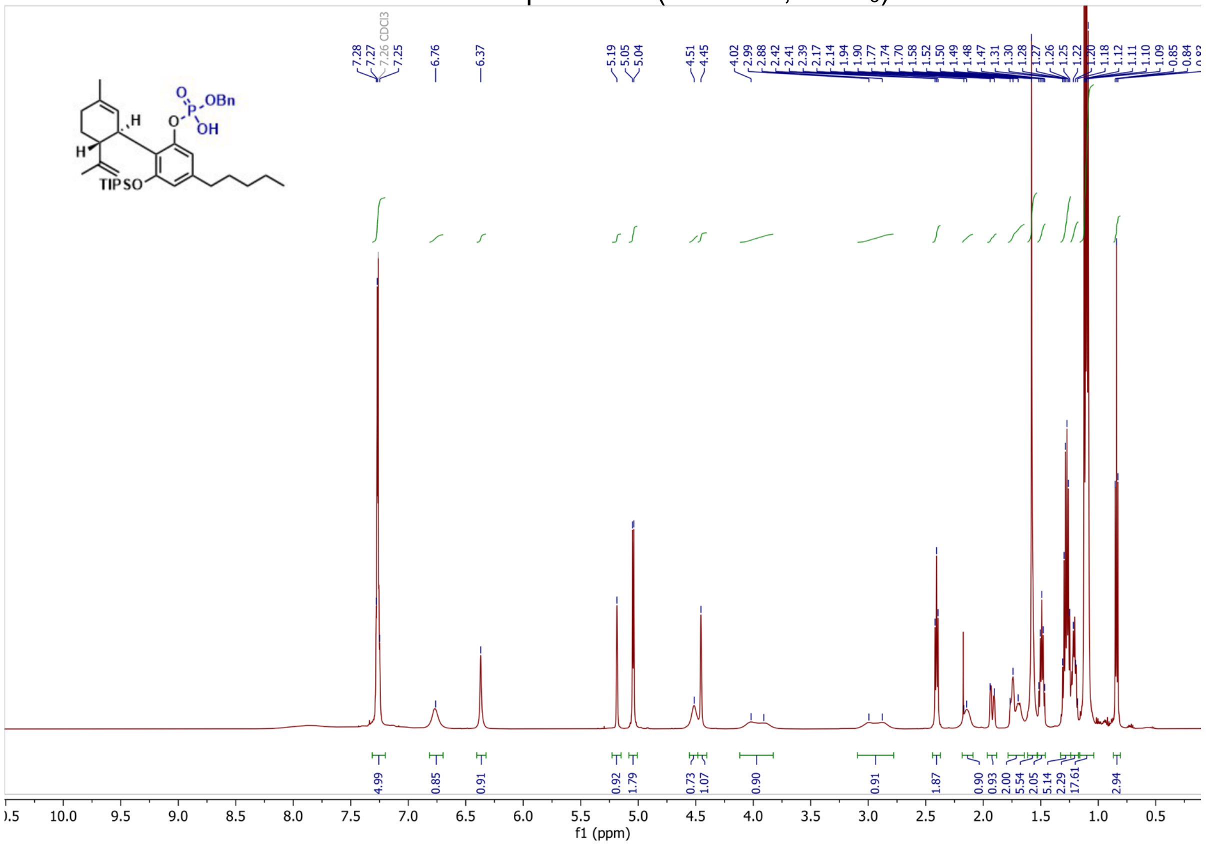

^{13}C NMR of Compound **40** (151 MHz, $CDCl_3$)

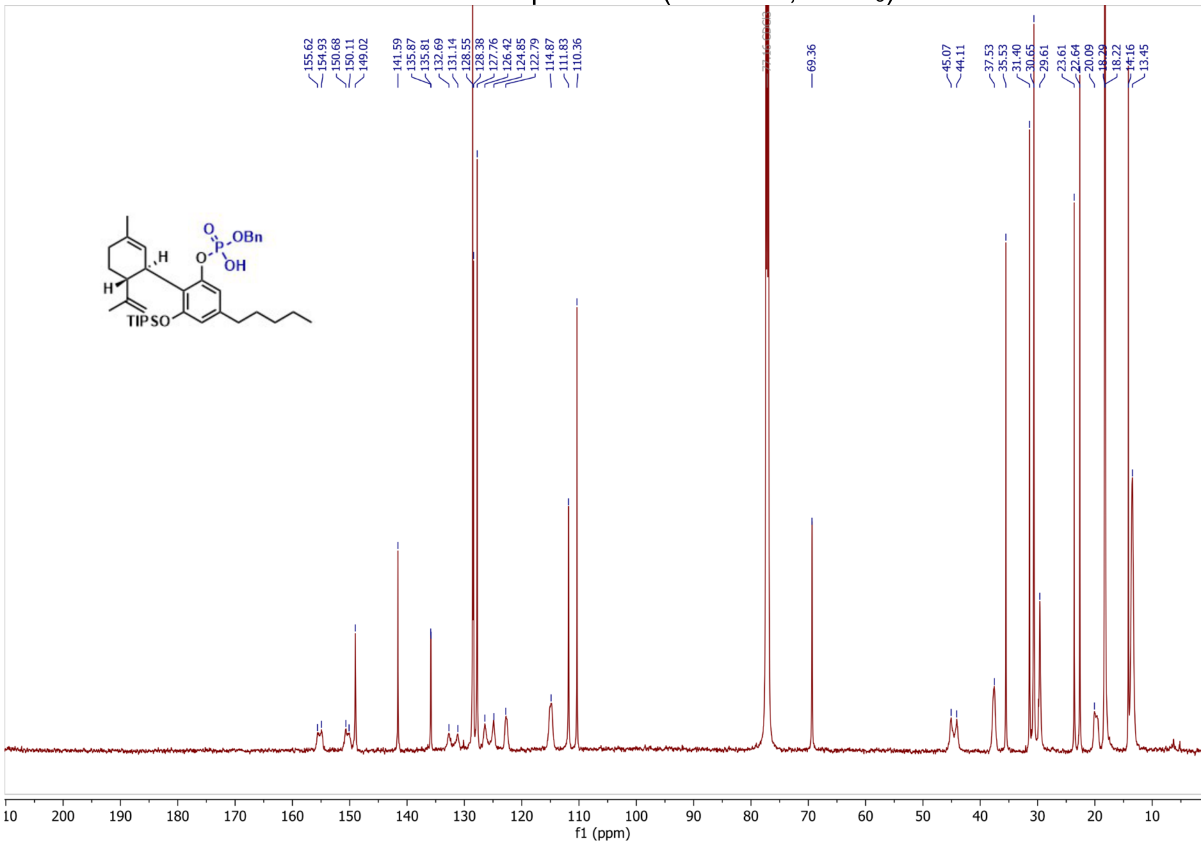

^{31}P NMR of Compound **40** (243 MHz, $CDCl_3$)

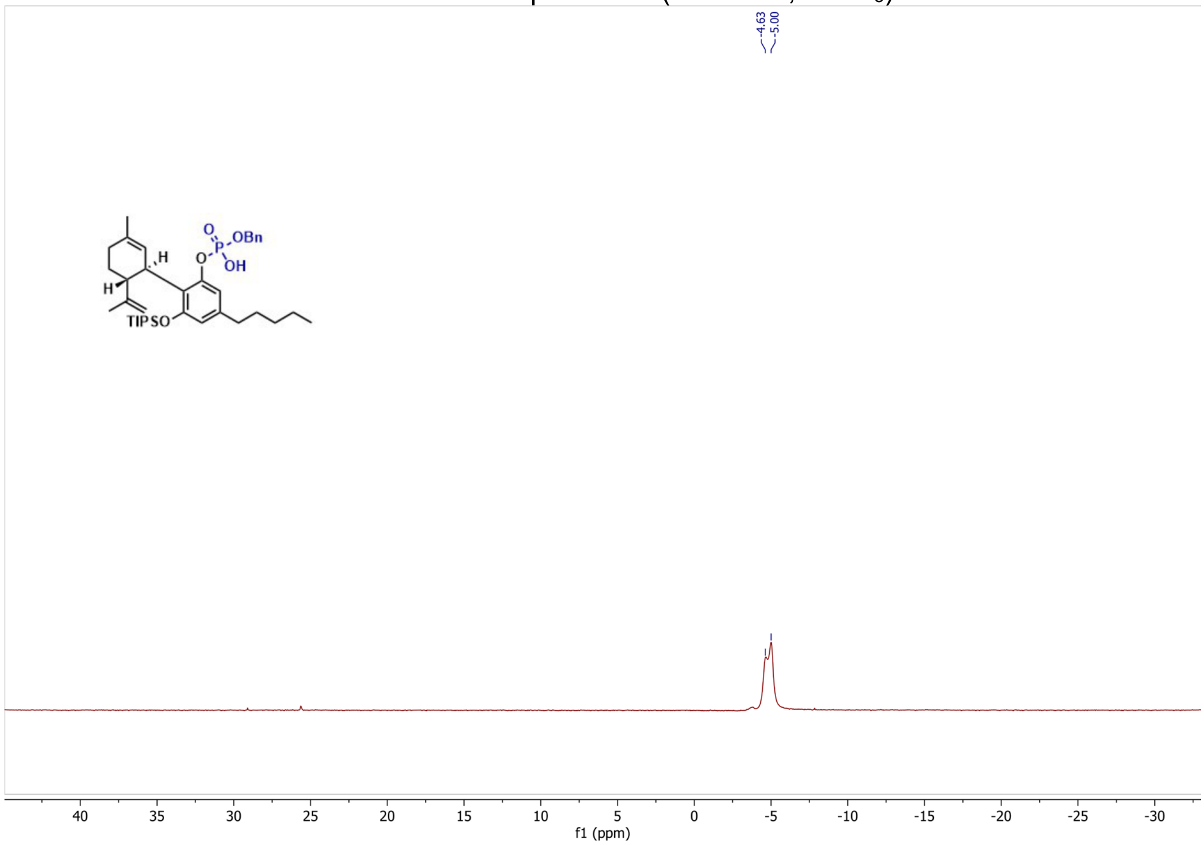

^{1}H NMR of Compound **41a** (600 MHz, $CDCl_3$)

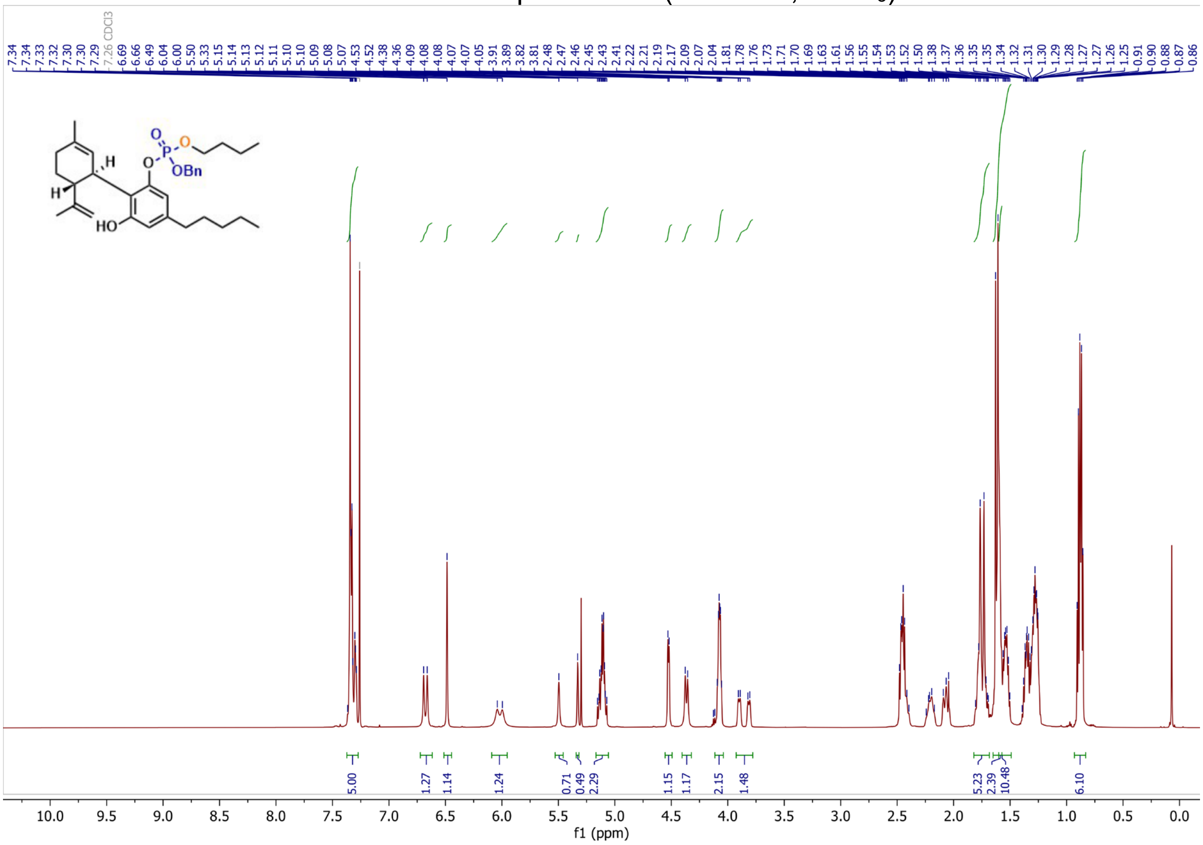

^{13}C NMR of Compound **41a** (151 MHz, $CDCl_3$)

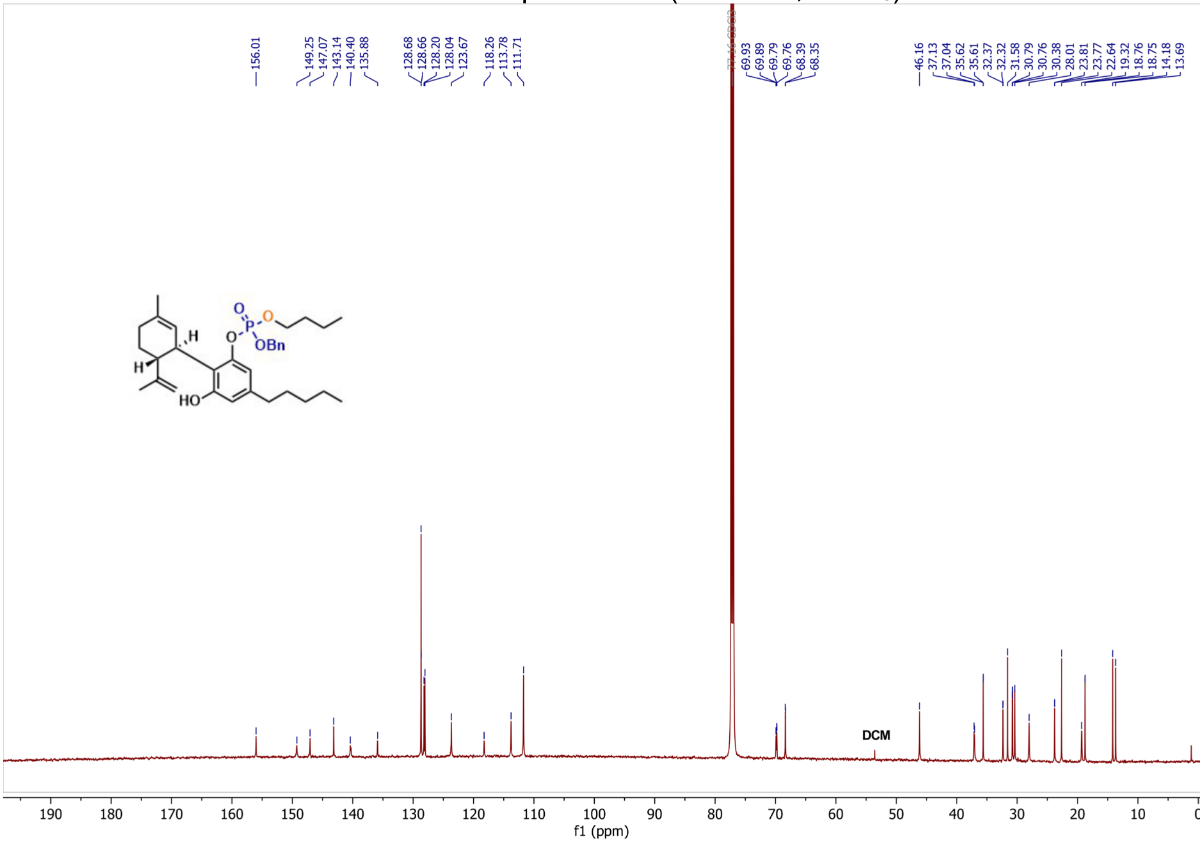

^{31}P NMR of Compound **41a** (243 MHz, $CDCl_3$)

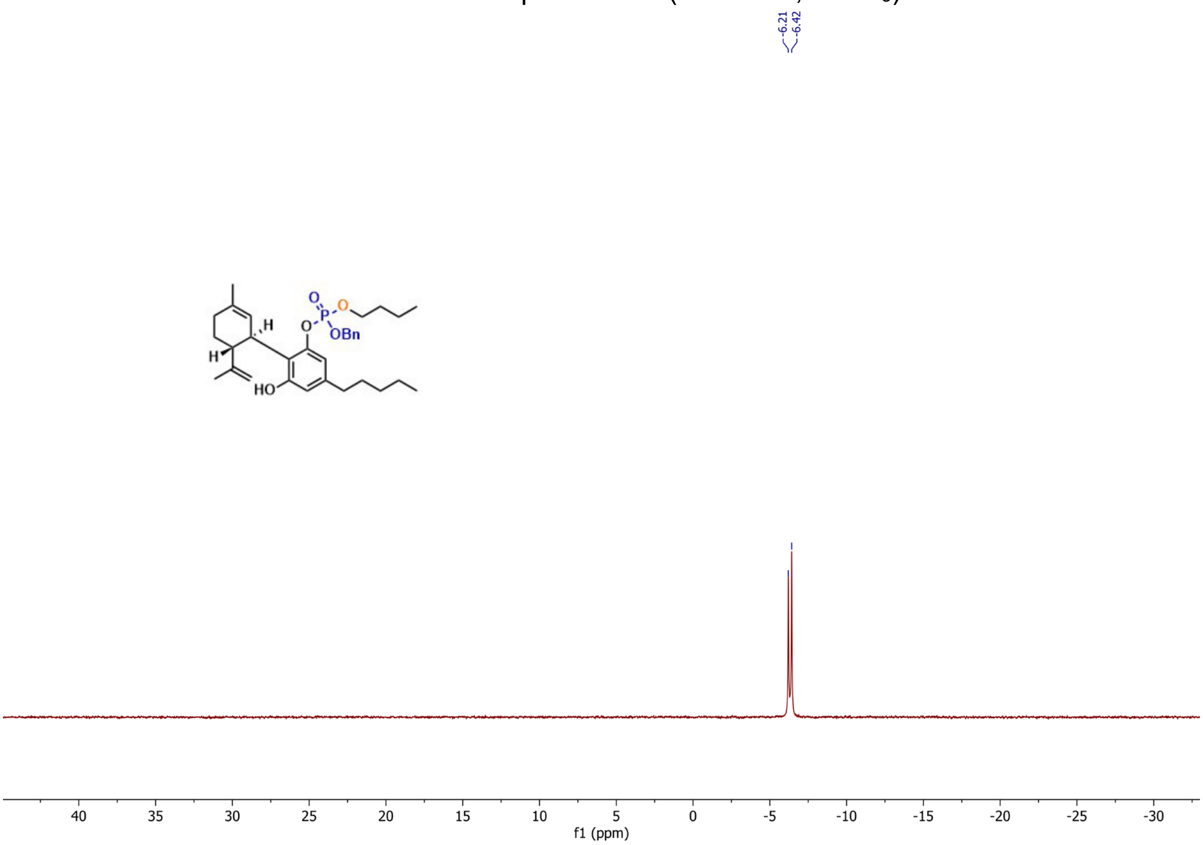

^{1}H NMR of Compound **41b** (600 MHz, $CDCl_3$)

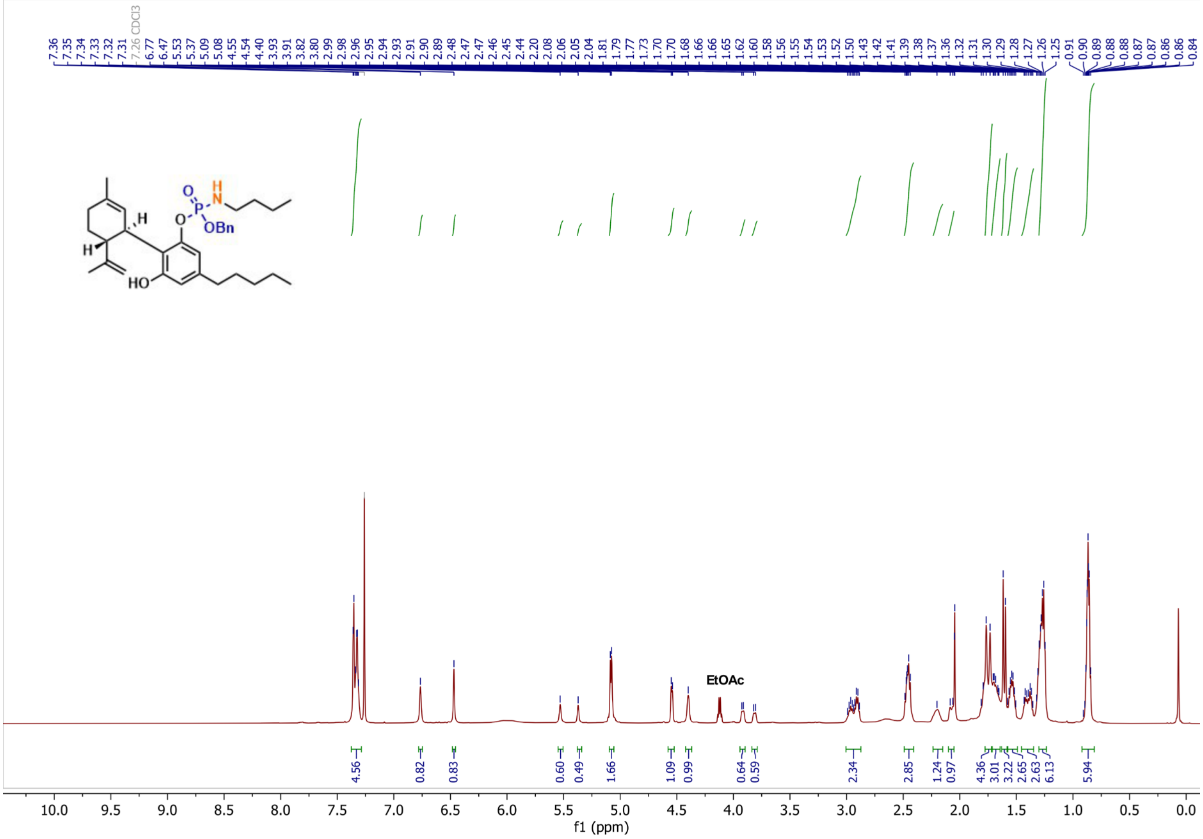

^{13}C NMR of Compound **41b** (151 MHz, $CDCl_3$)

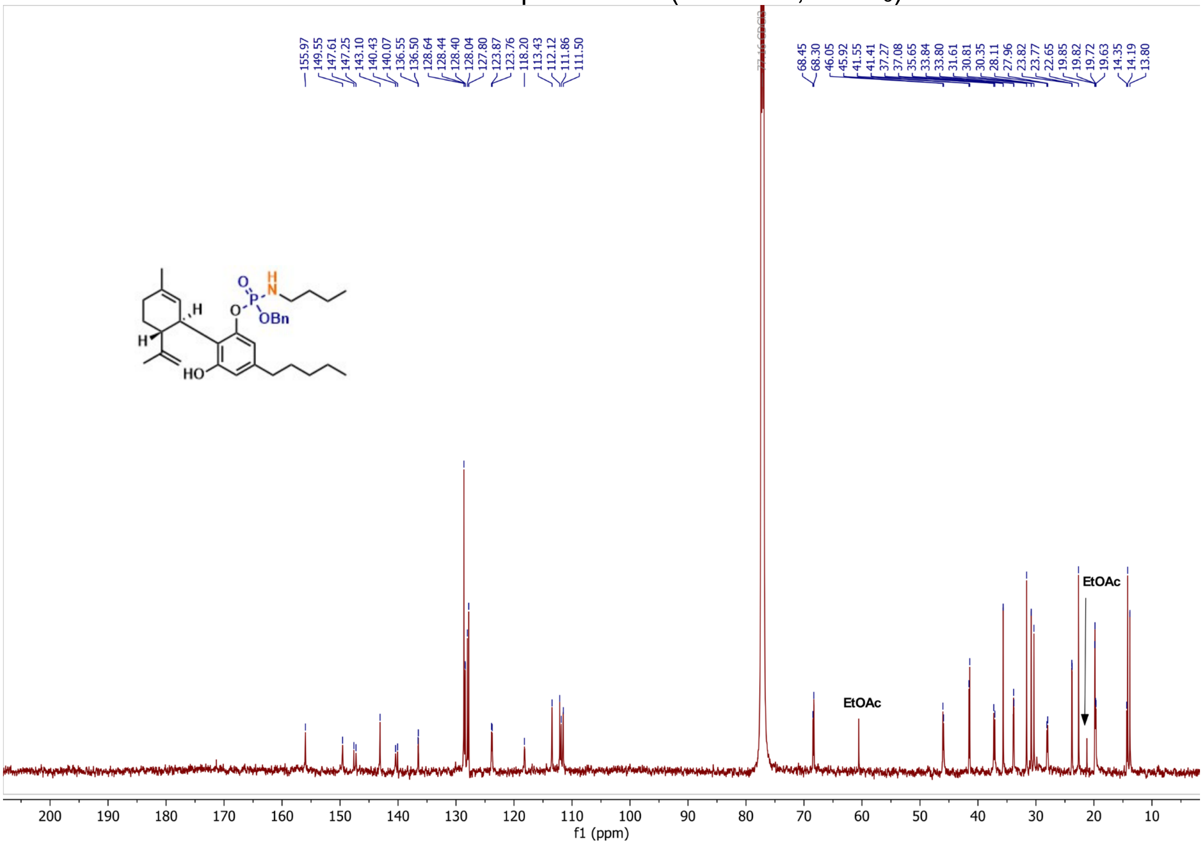

^{31}P NMR of Compound **41b** (243 MHz, $CDCl_3$)

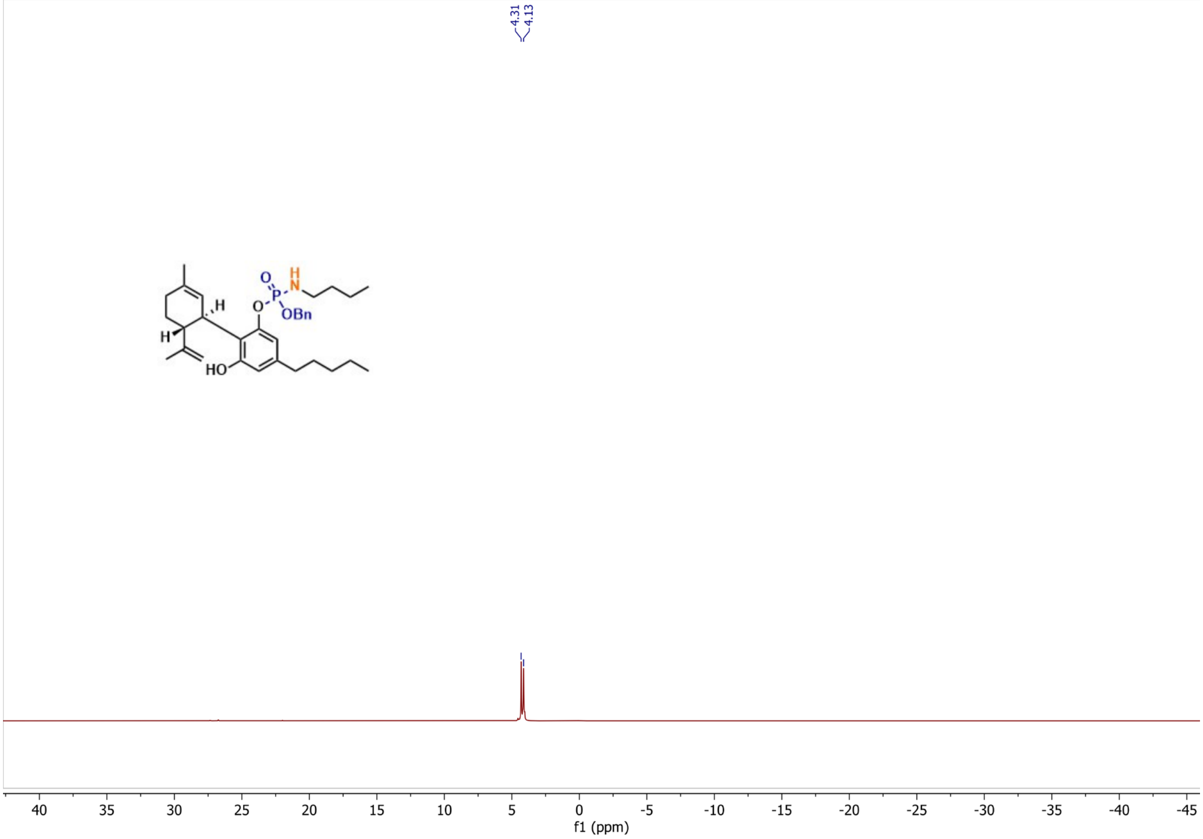

^{1}H NMR of Compound **42a** (600 MHz, MeOD)

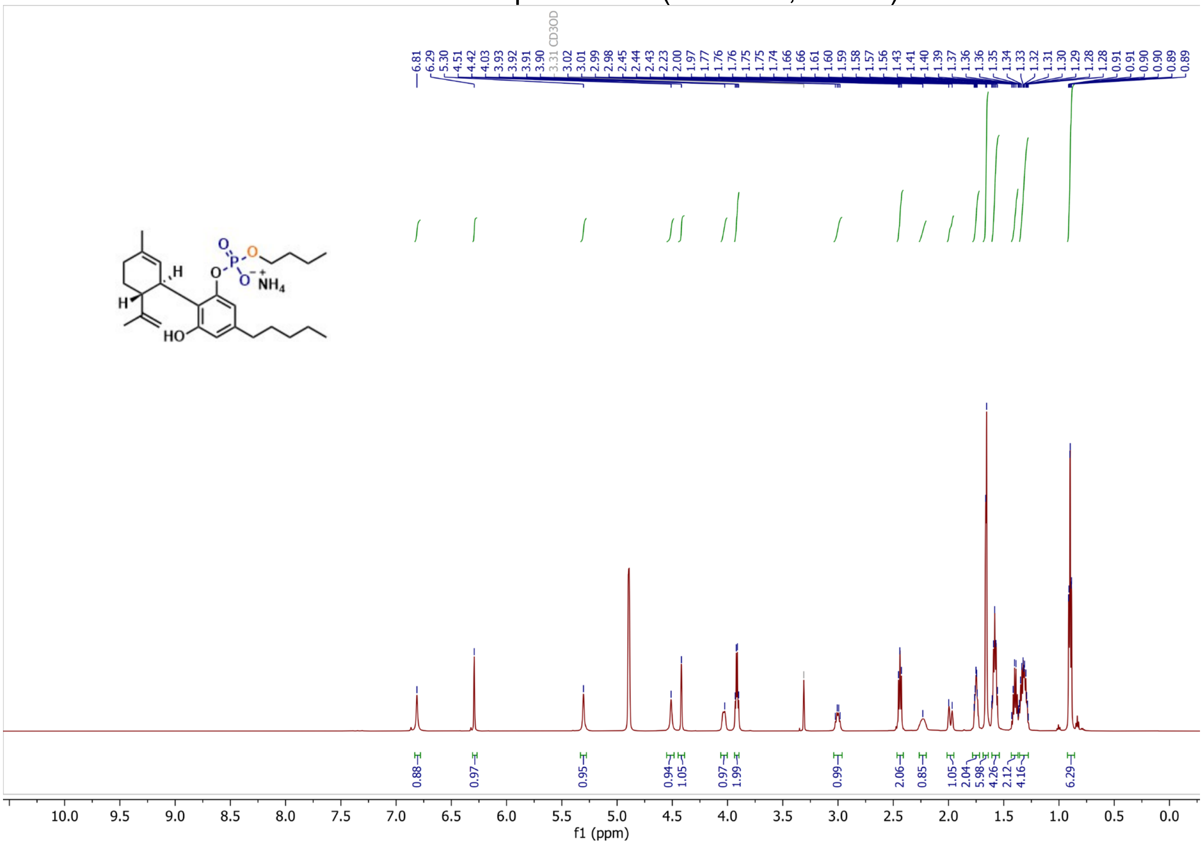

^{13}C NMR of Compound **42a** (151 MHz, MeOD)

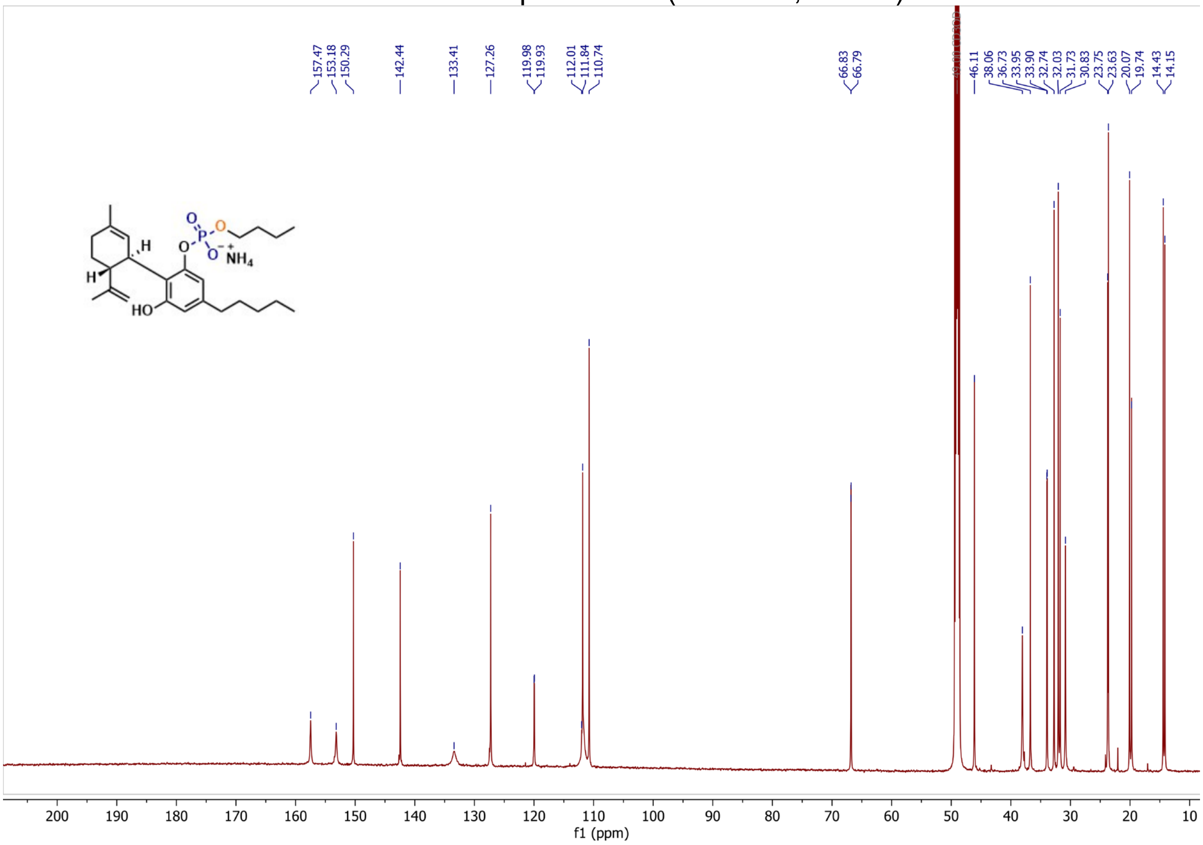

^{31}P NMR of Compound **42a** (243 MHz, MeOD)

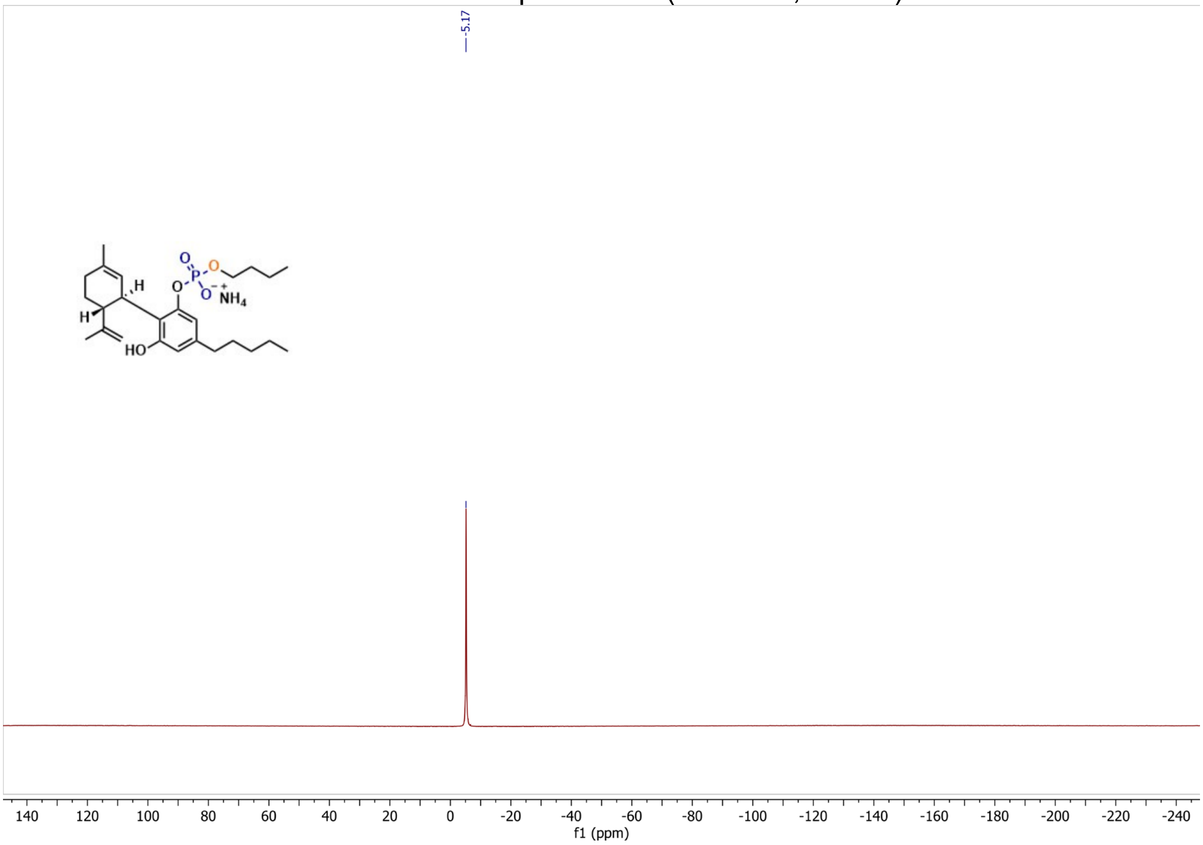

^{1}H NMR of Compound **42b** (600 MHz, MeOD)

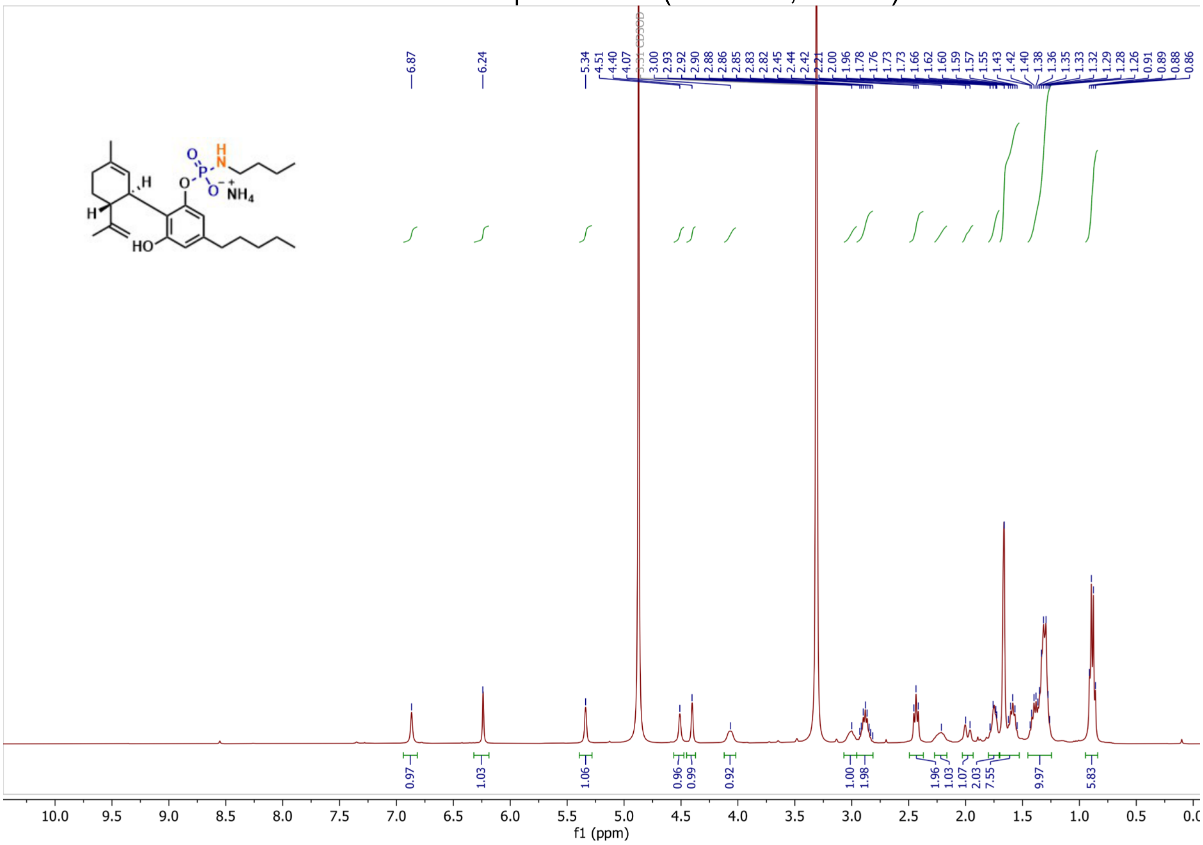

^{13}C NMR of Compound **42b** (151 MHz, MeOD)

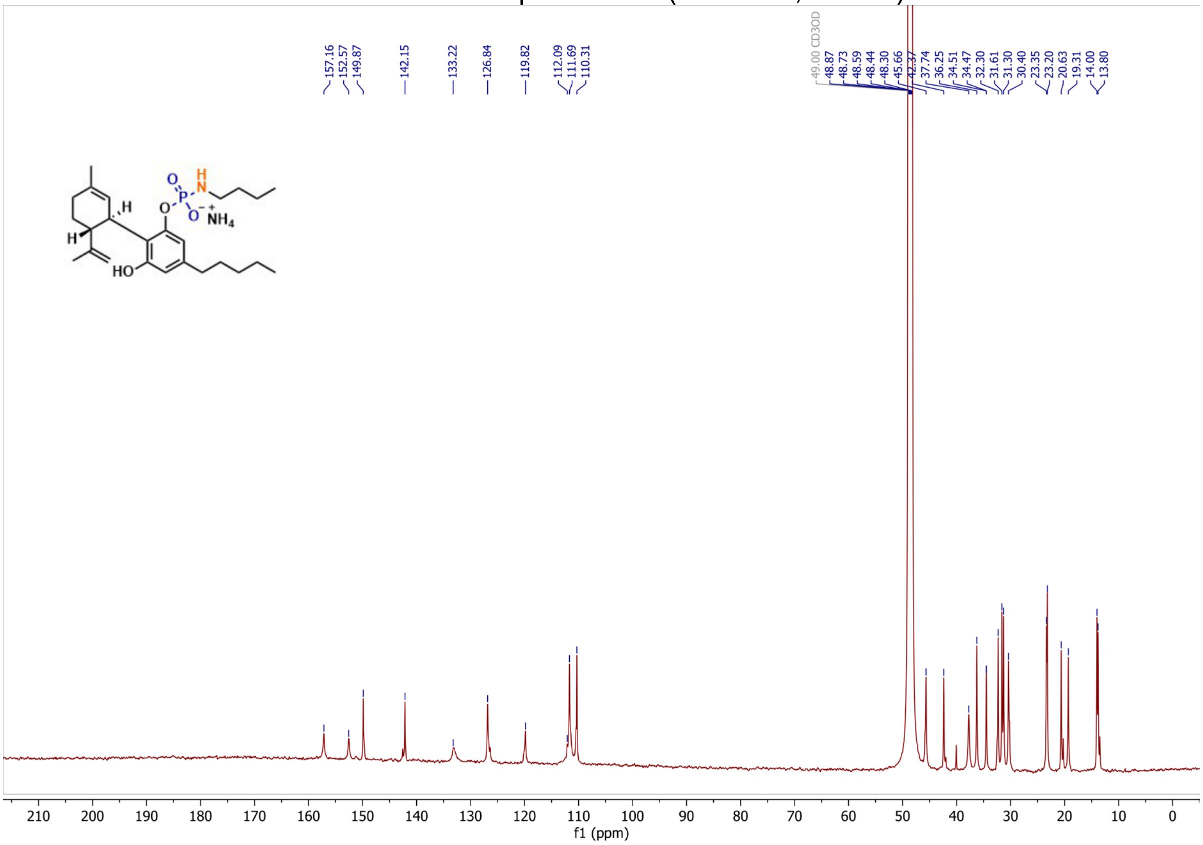

^{31}P NMR of Compound **42b** (243 MHz, MeOD)

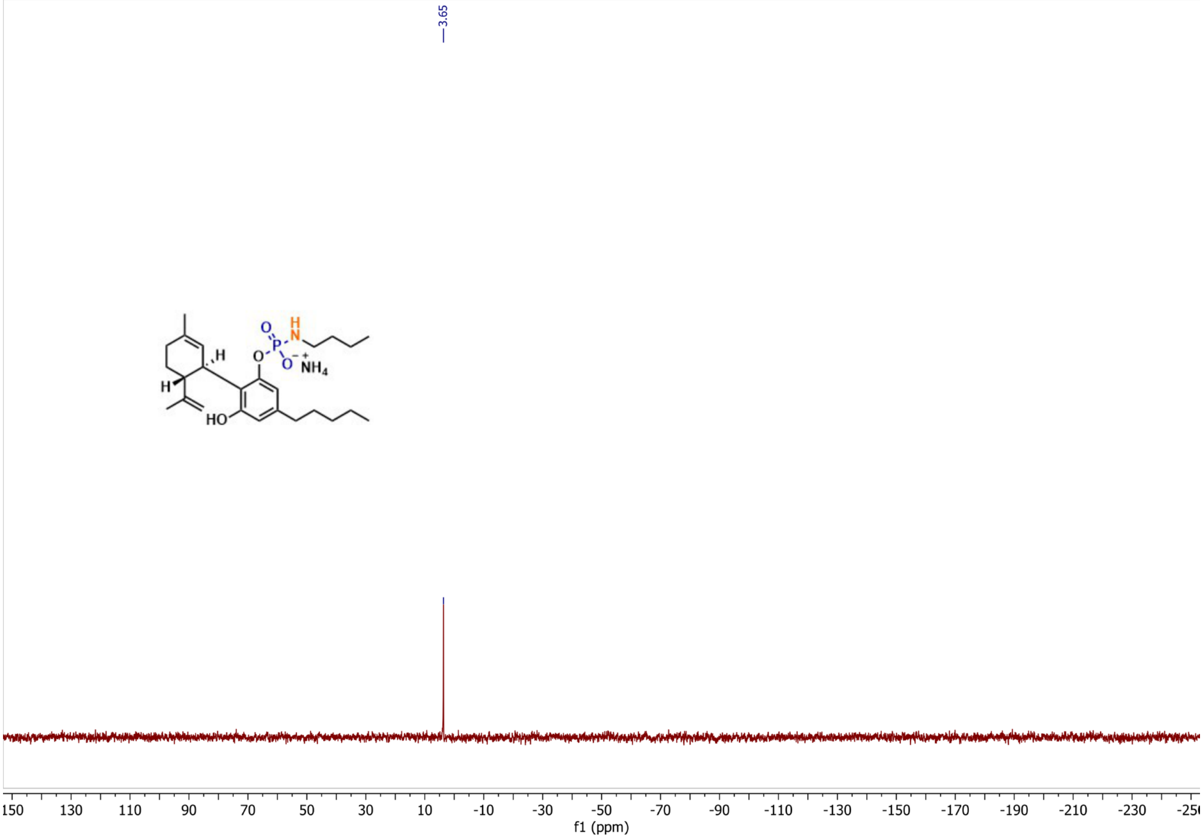

Supplement: Supplementary file 1 — ol4c04258_si_001.pdf [file ol4c04258_si_001.pdf]
