# Supplementary material for: Mild and Chemoselective Triethylsilane-Mediated Debenzylation for Phosphate Synthesis
Source: Org Lett. 2024 Dec 24;27(1):246–51. doi: 10.1021/acs.orglett.4c04258 (PMC11731398; doi:10.1021/acs.orglett.4c04258)
Supplement: Supplementary file 2 — ol4c04258_si_002.pdf [file ol4c04258_si_002.pdf]

Supporting Information

Mild and Chemoselective Triethylsilane Mediated Debenzylation for Phosphate Synthesis

Luke E. Hodson‡, Paul Joseph Tholath‡, Leon Jacobs, Nicole Pribut, Gouthami Pashikanti, Aletta E. van der Westhuyzen, David Laws III and Dennis C. Liotta*

Department of Chemistry, Emory University, 1515 Dickey Drive, Atlanta, Georgia, 30322, United States

*Correspondence to: dliotta@emory.edu

Table of Contents

1. General Experimental **S3**
2. General Procedures **S4**
General Procedure I: Mono-silylation of Cannabinoids S4
General Procedure II: Optimized Phosphorylation of Phenols Utilizing TBPP S4
General Procedure III: Silyl Deprotection of Cannabinoids S4
General Procedure IV: Optimized Phosphorylation of 1°, 2° and 3° Alcohols Utilizing TBPP S5
General Procedure V: Optimized Complete Debenzylation of Phosphates S5
General Procedure VI: Optimized Mono-debenzylation of Phosphates S6
General Procedure VII: Amidation/Esterification of Monobenzyl Phosphates S6
Guidelines S7
3. Methodology Optimization Information **S10**
General Quantitative ^{31}P NMR Experiment Information S10
TBPP Phosphorylation Optimization Information S10
Preliminary Attempted Chemoselective Phosphate Debenzylation S13
Phosphate Debenzylation Optimization Information S14
4. Experimental Procedures and Characterization Data **S16**
Preparation of Optimization Substrates and Cannabidiol Phosphate S16
Complete Scope of TBPP Phosphorylation and Triethylsilane Mediated Debenzylation. S20
Preparation of Dibenzyl Phosphate and Phosphonate Substrates S20
Debenzylation of Prepared Dibenzyl Phosphate and Phosphonate Substrates S38
Application of Mono-debenzylated Cannabidiol Phosphate S53
5. References **S56**

1. General Experimental

Chemistry. *Materials and Methods.* All chemicals were purchased from commercial vendors at the highest commercial quality and used without further purification. Cannabidiol (CBD) isolate was purchased from GVB Biopharma (purity 99.92%) and Δ-8-tetrahydrocannabinol was purchased from 3CHI (purity 96.6%). Dichloromethane (DCM), acetonitrile (MeCN), toluene, dimethylformamide (DMF), tetrahydrofuran (THF) and triethylamine (TEA) were purchased anhydrous in septum-sealed bottles from Sigma Aldrich. 1,8- Diazabicyclo[5.4.0]undec-7-ene (DBU, 33482-50ML-F), *n*-Butyllithium solution (2.5M in hexanes, 230707-100ML), lithium diisopropylamide solution (LDA, 1.0M in THF/hexanes, 774766-100ML), *tert*-Butylmagnesium chloride solution (1.0M in THF, 364649-100ML)**,** palladium(II) acetate (205869-1G) and triethylsilane 99% (230197-25G) was purchased from Sigma Aldrich. Tetrabenzyl pyrophosphate (TBPP) was purchased from ChemScene. All reactions were conducted using oven-dried glassware under an inert atmosphere of argon unless noted otherwise. Oil baths were utilized for all reactions that required heating. Isolated yields refer to chromatographically and spectroscopically (^{1}H NMR, ^{13}C NMR and ^{31}P NMR) homogeneous material, unless otherwise stated. Thin layer chromatography (TLC) was utilized to monitor reaction progress using Merck silica gel 60 F254 aluminum-backed plates. TLC spots were visualized with UV, $KMnO_4$, PMA, or ninhydrin stains. Normal and reverse phase flash chromatography was performed using a Teledyne Isco CombiFlash Rf system using RediSep® Rf silica gel disposable flash columns (60 Å pore size, 40-60 μm particle size) and RediSep Gold® C18Aq Reversed Phase Columns (20-40 μm particle size). NMR spectra were acquired using a 400 MHz Varian INOVA or 600 MHz Bruker Avance III. Chemical shifts are reported in δ ppm and referenced using residual solvent peaks ($CDCl_3$, MeOD, DMSO-d_6 or acetone-d_6). Rotamer signals are denoted with *. High resolution mass spectrometry (HRMS) was performed on a Thermo Exactive Plus Orbitrap Mass Spectrometer using APCI or ESI ionization methods. Liquid chromatography-mass spectrometry (LC-MS) was performed on an Agilent 1200 HPLC equipped with a 6120 Quadrupole mass spectrometer (ESI), Diode-array detector, and an Agilent InfinityLab Poroshell 120 EC-C18 (2.1 mm x 50 mm, 2.7 μm) column heated to 35 °C. Mobile Phase: water/MeOH (0.1% FA) or water/MeCN (0.1% FA). Purity was assessed as % of AUC_{total} at 254 nm. All final compounds were determined to be ≥95% pure unless stated otherwise.

2. General Procedures

2.1 General Procedure I: Mono-silylation of Cannabinoids

An oven dried 2-neck round bottom flask, equipped with a stir bar, was charged with cannabinoid (1 equiv.), imidazole (1.5 equiv) and anhydrous DCM (6mL/mmol cannabinoid, 0.167M). The reaction mixture was cooled to 0°C (ice-bath), and silyl triflate (1.3 equiv.) diluted in anhydrous DCM (2mL/mmol silyl triflate, 0.5M) was added dropwise. Following this addition, the ice-bath was removed, and the reaction mixture was allowed to warm to rt and stirred for 6 – 18 hr (dependent on silyl triflate utilized). Following complete consumption of the starting material as monitored by TLC, the reaction was quenched with H_2O, diluted with DCM and transferred to a separatory funnel. The organic layer was washed with H_2O (10mL/mmol cannabinoid), brine (10mL/mmol cannabinoid) and dried over anhydrous magnesium sulfate. After filtration, the crude product was evaporated in vacuo and purified by flash chromatography to afford the mono-silyl protected cannabinoid.

2.2 General Procedure II: Optimized Phosphorylation of Phenols Utilizing TBPP

An oven dried 2-neck round bottom flask, equipped with a stir bar, was charged with respective phenol (1 equiv.), TBPP (1.2 equiv.) and anhydrous MeCN (10mL/mmol phenol, 0.1M, see Guidelines regarding solubility of TBPP). The reaction mixture was cooled to 0°C (ice-bath), and DBU (1.5 equiv.) was added slowly. Following this addition, the ice-bath was removed, and the reaction mixture was allowed to warm to rt and stirred for 1 – 12 hr (substrate dependent). Following complete consumption of the starting material as monitored by TLC, the reaction mixture was either evaporated in vacuo and directly purified by flash chromatography or quenched with H_2O, diluted with EtOAc and transferred to a separatory funnel (see Guidelines). The organic layer was washed with H_2O (10mL/mmol phenol), brine (3 x 5mL/mmol phenol) and dried over anhydrous magnesium sulfate. After filtration, the crude product was evaporated in vacuo and purified by flash chromatography to afford the dibenzyl phosphate derivative.

2.3 General Procedure III: Silyl Deprotection of Cannabinoids

An oven dried 2-neck round bottom flask, equipped with a stir bar, was charged with silyl protected cannabinoid (1 equiv.), and anhydrous THF (5mL/mmol cannabinoid, 0.2M). The reaction mixture was cooled to 0°C (ice-bath), and AcOH (2 equiv.) was added, followed by the dropwise addition of 1M TBAF in THF (1.5 equiv.).

Note: Use of AcOH with cannabidiol substrates is critical to avoid deprotonation by TBAOH and

subsequent oxidation/degradation. Following this addition, the ice-bath was removed, and the reaction mixture was allowed to warm to rt and stirred for 2 hr or until complete consumption of the starting material as monitored by TLC. The reaction mixture was then quenched with H_2O, diluted with EtOAc and transferred to a separatory funnel. The organic layer was washed with H_2O (10mL/mmol cannabinoid), sat. aq. $NaHCO_3$ (3 x 5mL/mmol cannabinoid), brine (10mL/mmol cannabinoid) and dried over anhydrous magnesium sulfate. After filtration, the crude product was evaporated in vacuo and purified by flash chromatography to afford the deprotected cannabinoid derivative.

2.4 General Procedure IV: Optimized Phosphorylation of 1°, 2° and 3° Alcohols Utilizing TBPP

See Guidelines for case-specific recommendations of base selection to be employed in phosphorylation reactions. A flame-dried Schlenk flask, equipped with a stir bar, was charged with respective alcohol (1 equiv.) and anhydrous THF (5mL/mmol alcohol, 0.2M). The reaction mixture was cooled to 0°C (ice-bath), and respective base (1.2 equiv.) was added dropwise. The reaction mixture was then allowed to stir for 30 min at 0°C to ensure complete deprotonation, followed by the dropwise addition of a solution of TBPP (1.3 equiv.) in THF (3mL/mmol alcohol, 0.33M). Following this addition, the ice-bath was removed, and the reaction mixture was allowed to warm to rt and stirred for 4 – 16 hr (substrate dependent). Following complete consumption of the starting material as monitored by TLC, the reaction mixture was cooled to 0°C and carefully quenched with H_2O, diluted with EtOAc and transferred to a separatory funnel. The organic layer was washed with H_2O (10mL/mmol alcohol), brine (3 x 5mL/mmol alcohol) and dried over anhydrous magnesium sulfate. After filtration, the crude product was evaporated in vacuo and purified by flash chromatography to afford the dibenzyl phosphate derivative.

2.5 General Procedure V: Optimized Complete Debenzylation of Phosphates

An oven dried Schlenk flask, equipped with a stir bar, was charged with benzyl protected compound (1 equiv.), $Pd(OAc)_2$ (5 mol%.) and anhydrous DCM (6mL/mmol substrate, 0.17M). The reaction mixture was cooled to 0°C (ice-bath), and Et_3N (20 mol%.) was added, followed by the dropwise addition of Et_3SiH (2.5 equiv.). Following this addition, the ice-bath was removed, and the reaction mixture was allowed to warm to rt, during which time a black color developed over the course of 5 – 10 min. The reaction mixture was stirred for 30 min - 2 hr (substrate dependent) or until complete consumption of the starting material as monitored by TLC or LCMS analysis. The reaction mixture was then quenched with the addition of MeOH (10 mL/mmol substrate) and stirred for 5 min, resulting in the precipitation of palladium black which was filtered

through a plug of celite. The crude product was then evaporated in vacuo, followed by work-up and purification procedures (see Guidelines) to afford the phosphate derivative.

2.6 General Procedure VI: Optimized Mono-debenzylation of Phosphates

An oven dried schlenk flask, equipped with a stir bar, was charged with benzyl protected compound (1 equiv.), $Pd(OAc)_2$ (5 mol%.) and anhydrous DCM (6mL/mmol substrate, 0.17M). The reaction mixture was cooled to 0°C (ice-bath), and Et_3N (20 mol%.) was added, followed by the dropwise addition of Et_3SiH (1.25 equiv.). Following this addition, the reaction mixture was kept at 0°C, during which time a black color developed over the course of 5 - 20 min. The reaction mixture was stirred for 30 min - 2 hr (substrate dependent) or until complete consumption of the starting material as monitored by TLC or LCMS. The reaction mixture was then quenched with the addition of MeOH (10 mL/mmol substrate, degassed for amine containing substrates) and stirred for 5 min, resulting in the precipitation of palladium black which was filtered through a plug of celite. The crude product was then evaporated in vacuo, followed by work-up and purification procedures (see Guidelines) to afford the deprotected mono-benzyl phosphate derivative.

2.7 General Procedure VII: Amidation or Esterification of Mono-benzyl Phosphates

General procedure VII follows a modified literature procedure.[1] To an oven dried 150 mL 2-neck round bottom flask, equipped with a stir bar, was added mono-benzyl phosphate cannabinoid (1 equiv.), and anhydrous DCM (3mL/mmol cannabinoid). To this was added trichloroacetonitrile (3 equiv.), followed by a solution of triphenylphosphine (2 equiv.) in anhydrous DCM (2mL/mmol, 0.5M) and the reaction mixture was allowed to stir for 30 min. Then, a mixture of nucleophile (amine or alcohol, 1.3 equiv.) and Et_3N (3 equiv. for amine, 5.5 equiv. for alcohol) in anhydrous DCM (2mL/mmol, 0.5M) was added to the reaction mixture dropwise. The reaction mixture was then allowed to stir for 1 – 12 hr (substrate dependent) or until complete consumption of the starting material as monitored by TLC. The reaction mixture was then quenched with H_2O, diluted with DCM and transferred to a separatory funnel. The organic layer was washed with 1M HCl (10mL/mmol cannabinoid), sat. aq. $NaHCO_3$ (10mL/mmol cannabinoid), brine (10mL/mmol cannabinoid) and dried over anhydrous magnesium sulfate. After filtration, the crude product was evaporated in vacuo and purified by flash chromatography to afford the phosphodiester or phosphoramidate derivative.

2.8 Guidelines

What are the known storage and degradation issues with TBPP?

For the best results, TBPP should be stored at minimum in the fridge (2 – 8°C), but preferably at temperatures below -10°C under inert atmosphere in a sealed container. This is due to the tendency for degradation and breakdown of the pyrophosphate to dibenzyl phosphate during prolonged exposure to moisture in the air at room temperature. We have found properly stored TBPP to be viable for over a year when stored properly. Dibenzyl phosphate is less soluble in MeCN and other organic solvents and the presence of insoluble material when carrying out the phosphorylation reaction before addition of base is a clear indicator of the dibenzyl phosphate impurity. While larger volumes of MeCN can be utilized, or gentle warming of the reaction mixture to promote solvation prior to addition of base, we recommend purchase of a new bottle and proper storage and handling.

How can I decide what base will be most substrate-applicable in the phosphorylation reaction?

As has been demonstrated in these studies, for phenols and activated aliphatic alcohols (adjacent EWG) DBU serves as an effective and efficient base in reaction with TBPP. For the remaining 1°, 2° and 3° alcohols, we found that stronger bases such as n-BuLi, LDA or *t*BuMgCl were required to facilitate alkoxide formation and subsequent reaction with TBPP. n-BuLi was found to be the optimal choice of base and is recommended in most cases. However, we also found that the bulky nature of LDA can be taken advantage of when reaction of a 1° alcohol is desired over sterically congested 2° and 3° alcohols. We also found that *t*BuMgCl was only slightly less effective than n-BuLi in several examples and can serve as a viable substitute where lithium-halogen exchange poses a challenge.

Is a workup necessary following completion of the phosphorylation reaction?

While a workup is necessary when n-BuLi, LDA or *t*BuMgCl is utilized as base (excess base quench), substrates making use of DBU as base can forego the workup step and undergo direct purification by flash chromatography. We have found this to be most useful in substrates that are highly water soluble or hydrolytically unstable. While favorable and efficient, provisions must be made in large scale reactions for the excess dibenzyl phosphate byproduct (usually washed out in the aqueous layer) that is generated and will be part of the crude reaction mixture to be purified. This byproduct could co-elute with highly polar substrates, adding difficulty to the purification process.

Are there any extra precautions when undertaking debenzylation to avoid over reduction/reduction of extremely sensitive redox groups?

In this study, we have demonstrated that the stoichiometry of Et_3SiH is crucial for achieving selective and controlled debenzylation. However, for reactions with highly redox-sensitive groups (such as styrene), exact equivalent stoichiometric amounts (i.e 1 or 2 equivalents for mono-debenzylation or full debenzylation respectively) were utilized and temperatures of 0°C were maintained throughout the reaction.

Why must degassed methanol be used for quenching after debenzylation for amine containing substrates?

During method development, we observed that in rare cases, deprotection of Cbz-amine substrates led to the minor formation of a methylated amine byproduct. The source of methylation was traced to the quenching step with methanol, where residual active palladium likely generated a formaldehyde intermediate, leading to subsequent reductive amination. We found that degassing the methanol prior to its addition to the reaction mixture completely prevented methylation and byproduct formation. Although not strictly necessary as it only occurred in rare instances, this precaution should be applied in cases where Cbz deprotection is performed.

How do different substrates affect workup, purification and specific salt selection for isolation of phosphates following debenzylation?

A) For non-amine/non-protonatable, lipophilic substrates: The crude product was redissolved in DCM, and transferred to a separatory funnel, followed by the addition of 1M HCl (10mL/mmol substrate). The layers were separated, and the acidic aqueous layer was extracted with DCM (3 x 10mL/mmol cannabinoid). The combined organic layers were dried over anhydrous magnesium sulfate, filtered and evaporated in vacuo. The crude product was then purified by flash chromatography to afford the deprotected phosphate derivative. **Note:** In some cases, compounds of this class were isolated as the ammonium salt. This was particularly effective for mono debenzylated substrates. After following the workup conditions described above, and before purification, the crude product was redissolved in an excess amount of DCM:MeOH:$NH_4OH_{(aq)}$ (80:20:3) solution, stirred for 15 minutes and then evaporated in vacuo. The crude product was then purified by flash chromatography to afford the deprotected phosphate derivative as the ammonium salt. Alternatively, flash chromatography could be performed with DCM:MeOH:$NH_4OH_{(aq)}$ (80:20:3) as the polar eluent and DCM as mobile phase.

B) For amine/protonatable water-soluble substrates: The crude product was redissolved in DCM, cooled to 0°C (ice-bath) and protonated by the addition of 3 equivalents of 1M HCl in MeOH. The crude product was evaporated in vacuo and purified by flash

chromatography to afford the deprotected phosphate derivative as the HCl salt.

C) Unstable amines following protonation: In specific cases (such as with **21b**), we noticed that compounds were degrading when isolated as protonated salts. In this case, the crude product was redissolved in DCM, followed by the addition of 10 equivalents of Et_3N, to afford the anionic phosphate salt. The crude product was evaporated in vacuo and purified by flash chromatography to afford the deprotected phosphate derivative as the Et_3N salt.

What are the general guidelines for chromatography of phosphates following debenzylation?

Phosphate purification is often challenging due to the inherent polarity and the need for isolation as salts. While preparative RP-HPLC is typically optimal, we aimed to demonstrate that both normal and reverse phase flash chromatography could also be effective. The best results were achieved using reverse phase flash chromatography with RediSep Gold® C18Aq Reversed Phase Columns and methanol and H_2O as eluents. This approach allowed for an initial mobile phase of 100% H_2O, which prevented co-elution of early-eluting, highly polar substrates or phosphates with and salt byproducts from the reaction. This was particularly beneficial when workup steps were omitted, such as in the isolation of amines as hydrochloride salts. Ammonium and non-salt phosphates were easily isolated in this, typically eluting between 0 – 80% MeOH in H_2O. For non-polar phosphate substrates isolated as ammonium salts, such as **6**, normal phase column chromatography with DCM and methanol as eluents provided effective purification as well. Ammonium salt phosphates generally eluted between 5 – 80% MeOH in DCM. Alternatively, using a DCM and DCM:MeOH:$NH_4OH_{(aq)}$ (80:20:3) mixture as eluents was equally effective.

3. Methodology Optimization Information

General Quantitative ^{31}P NMR Experiment Information

^{31}P-qNMR was used for the quantification of yields directly in both the phosphorylation and debenzylation optimization reactions to avoid losses due to workups and flash purification. The quantification method was adapted from Ociepa et al. and modified to accommodate our substrates, internal standards and reagents.[2] Triphenyl phosphate (TPhP) was utilized as internal standard due to its inert nature and strong shift at -17.69 ppm, which did not overlap with any optimization substrate shifts. All optimization reactions were carried out on a 0.1 mmol scale and 2 mL of corresponding solvent. Using an accurately weighed amount of our internal standard (~1 mmol) we accurately quantified the yield of the reaction using the following equation.

$$Y_{NMR} = (I_{prod} * n_{sub2})/(I_{is} * n_{is})$$

Y_{NMR} – NMR yield [%].
I_{prod} – product signal integral.
I_{is} – internal standard signal integral.
n_{sub2} – mmol of starting material (CBD).
n_{is} – mmol of internal standard added to the reaction mixture.

TBPP Phosphorylation Optimization Information

For the initial TBPP phosphorylation reaction sequence, a Schlenk flask was charged with **3c** and TBPP under inert conditions, followed by the addition of base. After 2 hr reaction time, the internal standard was added, and the reaction mixture was allowed to stir for 10 min. Thereafter, an aliquot of 300 μL was taken, and transferred to NMR tube together with 300 μL of $CDCl_3$, followed by immediate ^{31}P-qNMR quantification. **Figure S1** illustrates a representative ^{31}P-NMR spectrum for TBPP phosphorylation optimization reactions. Signals for phosphorous containing internal standard, TBPP (-13.04 ppm) and dibenzyl phosphate (DBP) byproduct (0.70 ppm), were observed at their respective chemical shifts. However, the signal for **4c** appeared as a broad and split peak at δ -6.66 – -7.24. We hypothesized this was due to conformational locking of the sterically congested disubstituted phenol ring. To assess this, we ran successive ambient (25°C) and high temperature (60°C) ^{31}P-NMR experiments of the same sample, which led to the peak being resolved into a sharp singlet at δ -6.95 (as shown in **Figure S2**). Lastly, to ensure consistency in quantification, a comparison in the quantification of both spectra showed negligible difference in peak area. Based upon this evidence, all further ^{31}P-qNMR quantification experiments were run at 25°C. While not shown, time-course experiments revealed 2 hr as optimal reaction time. For complete optimization conditions refer to **Table S1**.

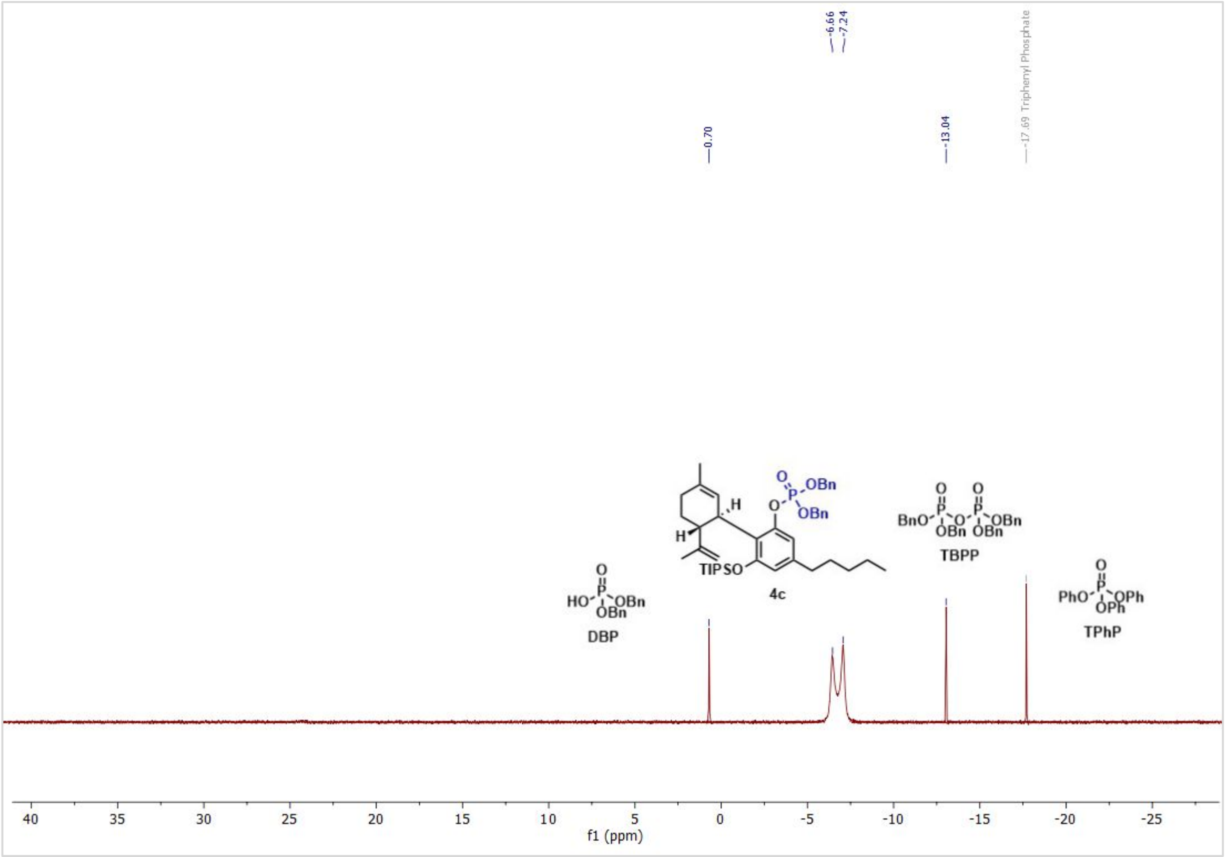

Figure S1. Representative ^{31}P-NMR spectrum (243 MHz, $CDCl_3$) for TBPP phosphorylation optimization reactions, highlighting all relevant substrates and byproducts in the reaction.

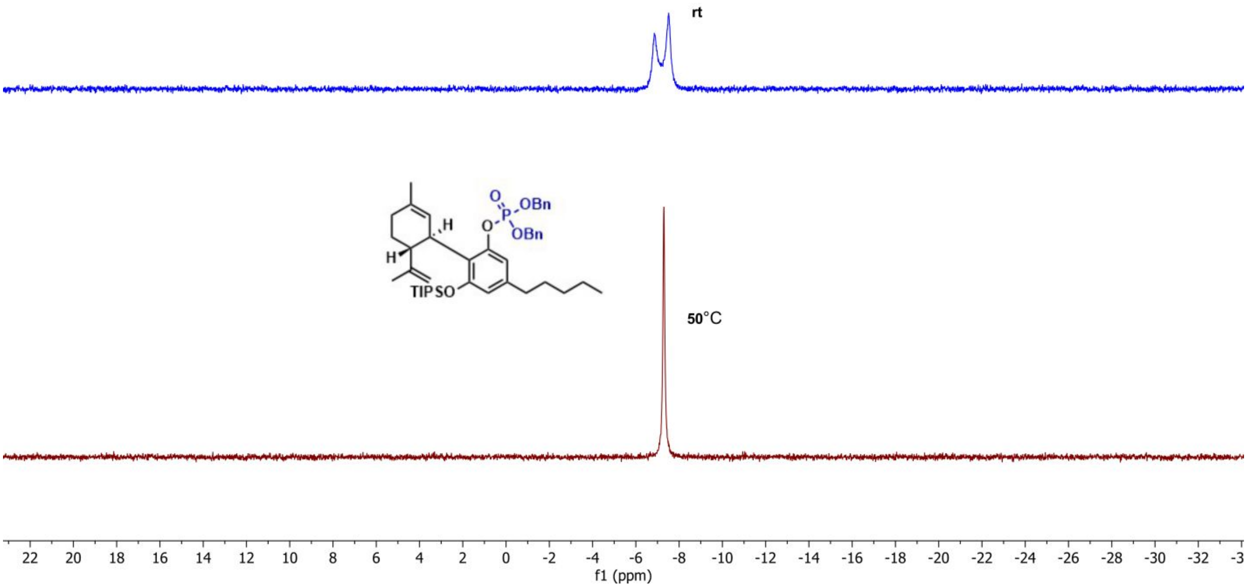

Figure S2. Comparative ambient (25°C) and high temperature (60°C) ^{31}P-NMR (243 MHz, $CDCl_3$) experiments of **4c**.

Table S1. Complete Optimization of TBPP Phosphorylation[a]

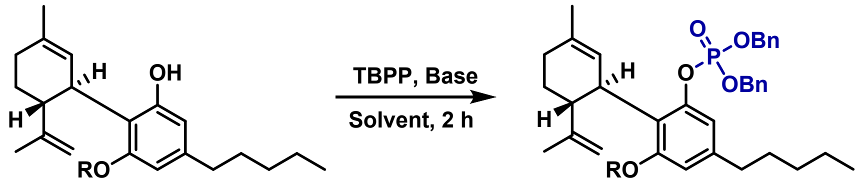

1, 3a-c 4a-c

Entry	R	TBPP (equiv.)	Base (equiv.)	Solvent	Yield (%)[b]
1	H (1)	1	DBU (1.2)	MeCN	ND
2	TES (3a)	1	DBU (1.2)	MeCN	16 (4a)
3	TBDMS (3b)	1	DBU (1.2)	MeCN	32 (4b)
4	TIPS (3c)	1	DBU (1.2)	MeCN	71 (4c)
5	TIPS	1.2	DBU (0.5)	MeCN	28
6	TIPS	1.2	DBU (1)	MeCN	52
7	TIPS	1.2	DBU (1.5)	MeCN	74
8	TIPS	1.2	DBU (2)	MeCN	67
9	TIPS	1.2	DBU (4)	MeCN	56
10	TIPS	0.5	DBU (1.5)	MeCN	37
11	TIPS	1	DBU (1.5)	MeCN	65
12	TIPS	2	DBU (1.5)	MeCN	66
13	TIPS	4	DBU (1.5)	MeCN	39
14	TIPS	1.2	Pyridine (1.5)	MeCN	0
15	TIPS	1.2	2,6-Lutidine (1.5)	MeCN	0
16	TIPS	1.2	DABCO (1.5)	MeCN	0
17	TIPS	1.2	DMAP (1.5)	MeCN	0
18	TIPS	1.2	Piperidine (1.5)	MeCN	0
19	TIPS	1.2	DIPEA (1.5)	MeCN	0
20	TIPS	1.2	Proton Sponge (1.5)	MeCN	0
21	TIPS	1.2	*t*-BuOK (1.5)	MeCN	62
22	TIPS	1.2	MTBD (1.5)	MeCN	75
23	TIPS	1.2	TBD (1.5)	MeCN	22
24	TIPS	1.2	LDA (1.5)	THF	36
25	TIPS	1.2	*n*-Buli (1.5)[c]	THF	81
26	TIPS	1.2	$Ti(OiPr)_4$ (0.1), DIPEA (1.5)	DCM	23
27	TIPS	1.2	$Ti(OBu)_4$ (0.1), DIPEA (1.5)	DCM	36
28	TIPS	1.2	*t*BuMgCl (1.2)[c]	THF	71
29	TIPS	1.2	DBU (1.2)	THF	51
30	TIPS	1.2	DBU (1.2)	Dioxane	12
31	TIPS	1.2	DBU (1.2)	DCM	50
32	TIPS	1.2	DBU (1.2)	Toluene	45
33	TIPS	1.2	DBU (1.2)	DMF	49
34	TIPS	1.2	DBU (1.5)	MeCN	76[d]

[a]Reaction conditions: **1** or **3** (0.1 mmol), TBPP in solvent (2.0 mL), then base for 2 h. [b]Yield determined by 31P-qNMR of crude mixture with triphenyl phosphate as internal standard. [c]Deprotonation first, then TBPP. [d]Isolated yield on 10g scale.

Preliminary Attempted Chemoselective Phosphate Debenzylation

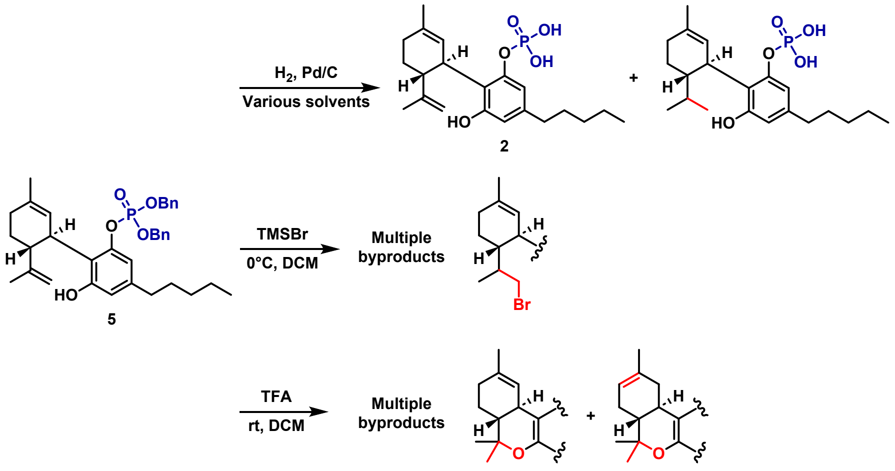

Figure S3. Preliminary attempted conditions for chemoselective phosphate debenzylation of **5**.

Several attempts towards the chemoselective phosphate debenzylation of **5** (**Figure S3**) were undertaken prior to discovery and optimization of Et_3SiH and $Pd(OAc)_2$. Initially, we employed the well-established, but non-selective, Pd/C-catalyzed hydrogenation to assess the feasibility of benzyl group removal while preserving the integrity of the terpene alkenes. Expectedly, use of catalytic Pd/C, H_2 and methanol as solvent afforded a complex mixture of products where reduction of one or both alkenes and removal of the benzyl groups had occurred. To modulate the reaction kinetics of catalytic hydrogenation and potentially promote chemoselective debenzylation, several solvents, including THF, IPA, and EtOAc, were systematically screened. At best, the use of IPA yielded a ~1:1 mixture of the desired compound **2** and a reduced product resulting from hydrogenation of the isopropylidene alkene, as determined through NMR analysis. Since these two products were chromatographically inseparable and isolating only the desired **2** would require a highly chemoselective method, this route was abandoned, and alternative strategies were explored.

Next, we explored the use of TMSBr in DCM at low temperatures, a method previously reported for the successful deprotection of pterostilbene dibenzyl phosphate.[3] While utilization of TMSBr resulted in the successful removal of the dibenzyl phosphate groups, as confirmed by LCMS and NMR analysis, this also led to the formation of numerous degradation products. We hypothesize that these unwanted side reactions arose from the inherent reactivity of the isopropylidene double bond, which may undergo an additional reaction via HBr generated *in situ*, leading to further decomposition of the compound and removal of the isopropylidene.[4]

Lastly, an attempt was made stirring **5** in a mixture of TFA in DCM. Unfortunately, this resulted in the formation of multiple, inseparable degradation byproducts, rendering this approach

unsuitable for achieving the desired transformation. As has been well documented in the literature, use of strong Lewis or Brønsted acids causes CBD to undergo intramolecular cyclization and further isomerization.[5] This is driven by the addition of a phenolic group to one of its two double bonds, of which the two main products are Δ^9-THC and Δ^8-THC. These failures underscore the inherent complexity and challenging nature of CBD's chemical reactivity and highlight the significance of the mild and chemoselective methodology developed in this work.

Phosphate Debenzylation Optimization Information

For Et_3SiH mediated debenzylation, a Schlenk flask was charged with $Pd(OAc)_2$, followed by **5** dissolved in 2 mL of DCM and cooled to 0°C. Thereafter, Et_3N and Et_3SiH were sequentially added, and the reaction was left to stir for 30 min. An aliquot of 300 µL was added to 300 µL MeOD and filtered through Whatman 0.45µ PTFE filter plug into an NMR tube, followed by immediate ^{31}P-qNMR quantification experiments. The illustrated spectrum below (**Figure S4**) represents all major products of the debenzylation reaction and internal standard, which was used to quantify both mono- and fully-debenzylated products (**6** and **2** respectively). Refer to **Table S2** for full Et_3SiH mediated debenzylation optimization parameters.

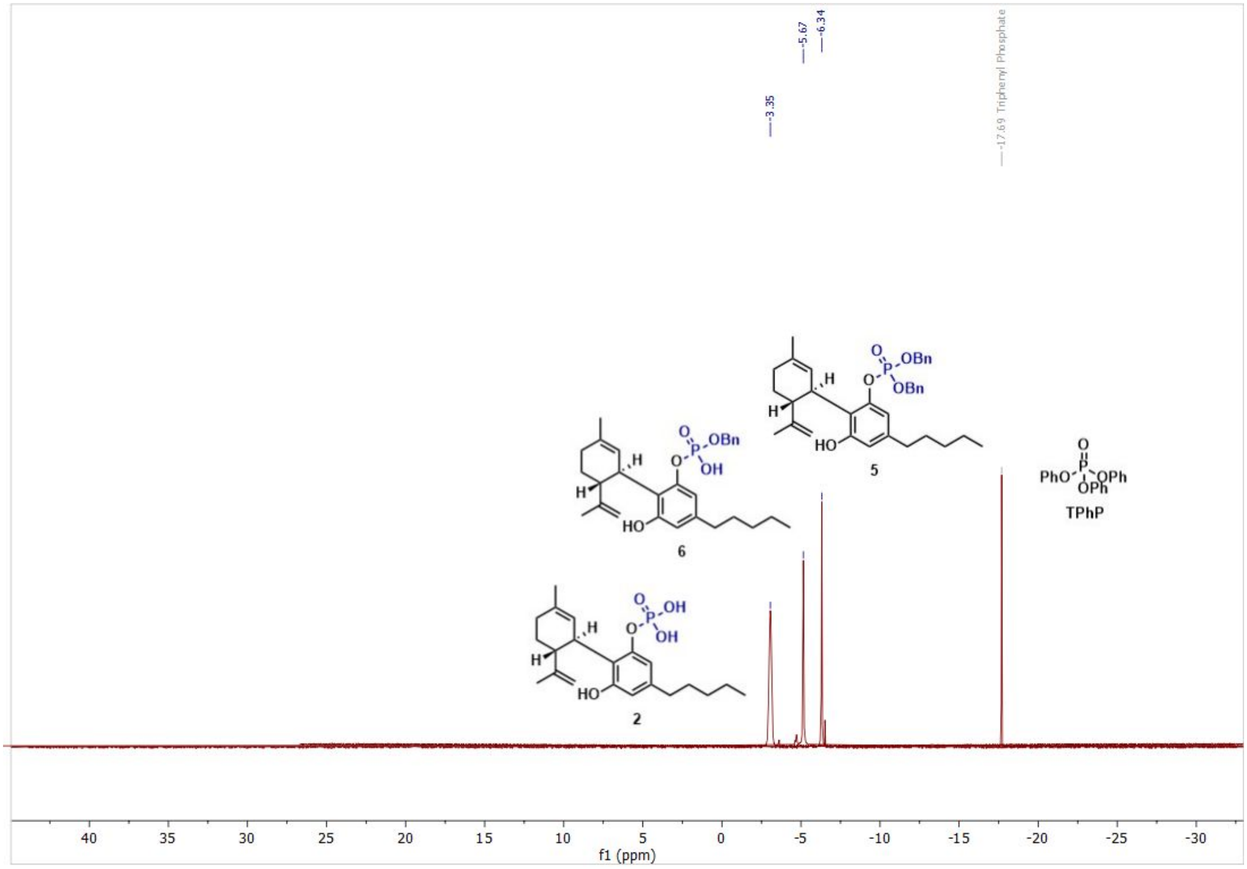

Figure S4. Representative ^{31}P-NMR spectrum (243 MHz, MeOD) for triethylsilane mediated debenzylation reactions, highlighting internal standard, starting material and both mono- and fully-debenzylated products.

Table S2. Complete Optimization of Phosphate Benzyl Deprotection[a]

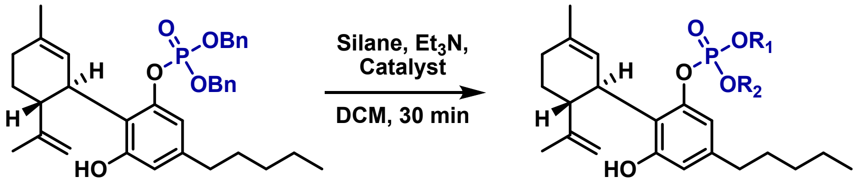

5

2: $R_1 = R_2 = OH$
6: $R_1 = Bn, R_2 = OH$

Entry	Silane (equiv.)	Et_3N (equiv.)	Catalyst (mol%.)	Yield (%) 6a	6b
1	Et_3SiH (2)	0.2	$Pd(OAc)_2$ (5)	40	16
2	Et_3SiH (0.5)	0.2	$Pd(OAc)_2$ (5)	0	20
3	Et_3SiH (1)	0.2	$Pd(OAc)_2$ (5)	0	35
4	Et_3SiH (1.25)	0.2	$Pd(OAc)_2$ (5)	2	60
5	Et_3SiH (1.5)	0.2	$Pd(OAc)_2$ (5)	10	56
6	Et_3SiH (2.5)	0.2	$Pd(OAc)_2$ (5)	67	13
7	Et_3SiH (3)	0.2	$Pd(OAc)_2$ (5)	62	14
8	Et_3SiH (4)	0.2	$Pd(OAc)_2$ (5)	58	15
13	Et_3SiH (2.5)	0	$Pd(OAc)_2$ (5)	0	0
14	Et_3SiH (1.25)	1.5	$Pd(OAc)_2$ (5)	4	52
15	Et_3SiH (1.25)	3	$Pd(OAc)_2$ (5)	2	55
16	Et_3SiH (2.5)	3	$Pd(OAc)_2$ (5)	60	2
9	Et_3SiH (1.25)	0.2	$Pd(OAc)_2$ (20)	7	33
10	Et_3SiH (1.25)	0.2	$Pd(OAc)_2$ (50)	21	36
11	Et_3SiH (2.5)	0.2	$Pd(OAc)_2$ (20)	32	8
12	Et_3SiH (2.5)	0.2	$Pd(OAc)_2$ (50)	41	0
17	Et_3SiH (2.5)	0.2	Pd/C (20)	0	0
18	Et_3SiH (2.5)	0.2	No Catalyst	0	0
19	PhSiH (2.5)	0.2	$Pd(OAc)_2$ (5)	17	30
20	EtO_3SiH (2.5)	0.2	$Pd(OAc)_2$ (5)	54	18
21	Et_3SiH (1.25)	0.2	$Pd(OAc)_2$ (5)	ND	71[c,d]
22	Et_3SiH (2.5)	0.2	$Pd(OAc)_2$ (5)	78[c]	ND

[a]Reaction conditions: **5** (0.1 mmol), $Pd(OAc)_2$ in DCM (2.0 mL), then Et_3N and then silane for 30 min. [b]Yield determined by ^{31}P-qNMR of crude mixture with triphenyl phosphate as internal standard. [c]Isolated yield on 1g scale. [d]Isolated as ammonium salt.

4. Experimental Procedures and Characterization Data

Preparation of Optimization Substrates and Cannabidiol Phosphate

(1'*R*,2'*R*)-5'-methyl-4-pentyl-2'-(prop-1-en-2-yl)-6-[(triethylsilyl)oxy]-1',2',3',4'-tetrahydro-(1,1'-biphenyl)-2-ol (3a)

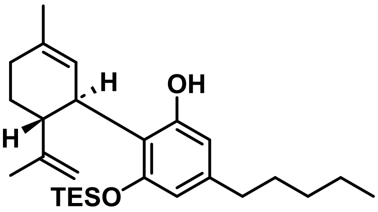

Following general procedure I, compound **3a** was obtained by reaction of cannabidiol (629 mg, 2.00 mmol) with triethylsilyl trifluoromethansulfonate. The crude product was purified by flash chromatography (0 – 5% EtOAc in hexanes) to afford the title compound (780 mg, 1.82 mmol, **91%**). **Physical state**: Clear oil; **^{1}H NMR (600 MHz, CDCl$_3$)** δ 6.28 (s, 1H), 6.16 (s, 1H), 5.90 (br s, 1H), 5.54 (br s, 1H), 4.54 (s, 1H), 4.42 (br s, 1H), 3.96 – 3.91 (m, 1H), 2.48 – 2.46 (m, 1H), 2.44 (t, *J* = 7.6 Hz, 2H), 2.25 – 2.17 (m, 1H), 2.11 – 2.04 (m, 1H), 1.83 – 1.72 (m, 5H), 1.64 (s, 3H), 1.56 (p, *J* = 7.1 Hz, 2H), 1.36 – 1.23 (m, 4H), 0.98 (t, *J* = 8.0 Hz, 9H), 0.88 (t, *J* = 7.0 Hz, 3H), 0.81 – 0.69 (m, 6H); **^{13}C NMR (151 MHz, CDCl$_3$)** δ 156.1, 154.2, 147.5, 142.4, 139.6, 124.7, 117.4, 111.2, 110.7, 109.9, 46.2, 36.6, 35.7, 31.5, 30.8, 30.5, 28.3, 23.8, 22.7, 19.7, 14.2, 6.9 (3C), 5.4 (3C); **HRMS (APCI)** m/z: [M+H]$^+$ calc. for $C_{27}H_{45}O_2Si$: 429.3183, observed: 429.3188.

(1'*R*,2'*R*)-6-[(*tert*-butyldimethylsilyl)oxy]-5'-methyl-4-pentyl-2'-(prop-1-en-2-yl)-1',2',3',4'-tetrahydro-(1,1'-biphenyl)-2-ol (3b)

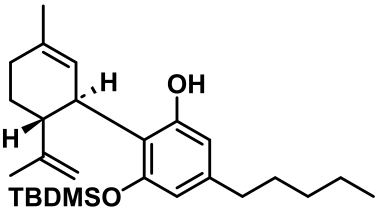

Following general procedure I, compound **3b** was obtained by reaction of cannabidiol (629 mg, 2.00 mmol) with *tert*-butyldimethylsilyl trifluoromethansulfonate. The crude product was purified by flash chromatography (0 – 5% EtOAc in hexanes) to afford the title compound (772 mg, 1.80 mmol, **90%**). **Physical state**: Clear oil; **^{1}H NMR (600 MHz, CDCl$_3$)** δ 6.29 (s, 1H), 6.19 (s, 1H), 5.87 (s, 1H), 5.52 (s, 1H), 4.56 (s, 1H), 4.47 (s, 1H), 3.93 (m, 1H), 2.49 – 2.42 (m, 3H), 2.26 – 2.17 (m, 1H), 2.08 (m, 1H), 1.84 – 1.72 (m, 5H), 1.62 (s, 3H), 1.60 – 1.51 (m, 2H), 1.37 – 1.23 (m, 4H), 0.98 (s, 9H), 0.88 (t, *J* = 7.0 Hz, 3H), 0.21 (s, 3H), 0.17 (s, 3H); **^{13}C NMR (151 MHz, CDCl$_3$)** δ 156.1, 153.9, 147.5, 142.2, 139.5, 124.5, 117.7, 111.6, 110.9, 110.0, 45.7, 36.8, 35.5, 31.4, 30.7, 30.4, 28.2, 26.0, 25.9, 23.7, 22.6, 20.1, 18.4, 14.1, -3.9, -4.1; **HRMS (APCI)** m/z: [M+H]$^+$ calc. for

$C_{27}H_{45}O_2Si$: 429.3183, observed: 429.3191.

(1'*R*,2'*R*)-5'-methyl-4-pentyl-2'-(prop-1-en-2-yl)-6-[(triisopropylsilyl)oxy]-1',2',3',4'-tetrahydro-(1,1'-biphenyl)-2-ol (3c)

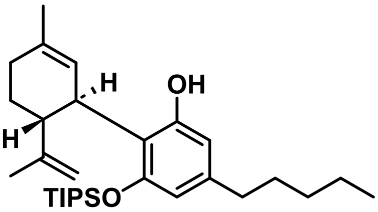

Following general procedure I, compound **3c** was obtained by reaction of cannabidiol (629 mg, 2.00 mmol) with triisopropylsilyl trifluoromethansulfonate. Synthesis of compound required heating to 40°C (oil bath) for the duration of the reaction. The crude product was purified by flash chromatography (0 – 5% EtOAc in hexanes) to afford the title compound (819 mg, 1.74 mmol, **87%**). **Physical state**: Clear oil; **^{1}H NMR (600 MHz, CDCl$_3$)** δ 6.29 (s, 1H), 6.20 (s, 1H), 5.91 (s, 1H), 5.55 (s, 1H), 4.59 (s, 1H), 4.51 (s, 1H), 4.06 (s, 1H), 2.54 – 2.47 (m, 1H), 2.46 (t, *J* = 7.6 Hz, 2H), 2.24 (m, 1H), 2.13 – 2.06 (m, 1H), 1.84 – 1.71 (m, 5H), 1.63 (s, 3H), 1.61 – 1.54 (m, 2H), 1.38 – 1.24 (m, 7H), 1.14 (d, *J* = 7.5 Hz, 9H), 1.10 (d, *J* = 7.5 Hz, 9H), 0.89 (t, *J* = 7.1 Hz, 3H); **^{13}C NMR (151 MHz, CDCl$_3$)** δ 156.1, 154.2, 147.5, 142.0, 139.3, 124.7, 117.1, 110.8, 110.6, 109.6, 45.6, 36.9, 35.5, 31.3, 30.6, 30.4, 28.1, 23.7, 22.6, 20.4, 18.2 (3C), 18.1 (2C), 14.1 (2C), 13.2 (2C). **HRMS (APCI)** m/z: $[M+H]^+$ calc. for $C_{30}H_{51}O_2Si$: 471.3653, observed: 471.3658.

Dibenzyl {(1'*R*,2'*R*)-5'-methyl-4-pentyl-2'-(prop-1-en-2-yl)-6-[(triisopropylsilyl)oxy]-1',2',3',4'-tetrahydro-(1,1'-biphenyl)-2-yl} phosphate (4c)

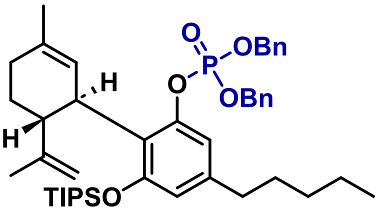

Following general procedure II, compound **4c** was obtained from phosphorylation of **3c** (10.0 g, 21.2 mmol). The crude product was purified by flash chromatography (0 – 40% EtOAc in hexanes) to afford the title compound (11.8 g, 16.1 mmol, **76%**). **Physical state**: Clear oil; **^{1}H NMR (600 MHz, CDCl$_3$)** δ 7.37 – 7.23 (m, 10H), 6.80 (br s, 1H), 6.38 (s, 1H), 5.19 (br s, 1H), 5.13 – 5.04 (m, 4H), 4.52 (br s, 1H), 4.46 (s, 1H), 4.16 – 3.71 (m, 1H), 3.13 – 2.76 (m, 1H), 2.42 (t, *J* = 7.7 Hz, 2H), 2.13 – 2.01 (m, 1H), 1.97 – 1.90 (m, 1H), 1.77 – 1.64 (m, 2H), 1.56 (s, 6H), 1.50 (p, *J* = 7.6 Hz, 2H), 1.34 – 1.20 (m, 7H), 1.14 – 1.06 (m, 18H), 0.86 (t, *J* = 7.1 Hz, 3H); **^{13}C NMR (151 MHz, CDCl$_3$)** δ 154.8, 150.9, 149.0, 141.7, 135.9 (2C), 132.2, 128.6 (4C), 128.6, 128.6, 128.0 (4C), 127.9, 125.7, 122.4, 114.7, 111.8, 110.3, 69.7, 45.3, 43.9, 37.5, 35.6, 31.4, 30.7,

29.7, 23.6, 22.6, 19.9, 18.3 (3C), 18.2 (6C), 14.2, 13.4; **^{31}P NMR (243 MHz, CDCl$_3$)** δ -6.95 (d, *J* = 142.0 Hz); **HRMS (APCI)** m/z: [M+H]$^+$ calc. for $C_{44}H_{64}O_5PSi$: 731.4255, observed: 731.4259.

Dibenzyl [(1'*R*,2'*R*)-6-hydroxy-5'-methyl-4-pentyl-2'-(prop-1-en-2-yl)-1',2',3',4'-tetrahydro-(1,1'-biphenyl)-2-yl] phosphate (5)

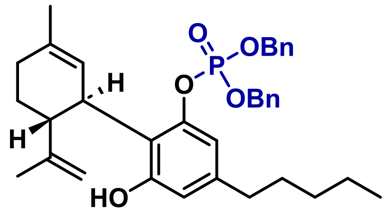

Following general procedure III, compound **5** was obtained by deprotection of **4c** (7.31 g, 10.0 mmol). The crude product was purified by flash chromatography (0 – 60% EtOAc in hexanes) to afford the title compound (5.23 g, 9.10 mmol, **91%**). **Physical state**: Clear oil; **^{1}H NMR (600 MHz, CDCl$_3$)** δ 7.35 – 7.25 (m, 10H), 6.71 (s, 1H), 6.53 (s, 1H), 6.16 (s, 1H), 5.31 (s, 1H), 5.15 – 5.05 (m, 4H), 4.53 (s, 1H), 4.39 (s, 1H), 3.86 (s, 1H), 2.52 – 2.42 (m, 3H), 2.19 (s, 1H), 2.07 – 2.01 (m, 1H), 1.81 – 1.74 (m, 1H), 1.71 (s, 3H), 1.62 (s, 3H), 1.53 (p, *J* = 6.5 Hz, 2H), 1.35 – 1.23 (m, 4H), 0.88 (t, *J* = 7.0 Hz, 3H).; **^{13}C NMR (151 MHz, CDCl$_3$)** δ 156.0, 149.2, 147.1, 143.2, 140.3, 135.7 (d, J_{CP} = 7.3 Hz) (2C), 128.7 (2C), 128.7 (4C), 128.2 (2C), 128.1 (2C), 123.6, 118.3, 113.9, 111.8, 111.7, 70.00 (d, J_{CP} = 5.5 Hz), 69.90 (d, J_{CP} = 5.5 Hz), 46.1, 37.1, 35.6, 31.6, 30.8, 30.4, 28.0, 23.8, 22.6, 19.3, 14.2; **^{31}P NMR (243 MHz, CDCl$_3$)** δ -6.33; **HRMS (APCI)** m/z: [M+H]$^+$ calc. for $C_{35}H_{44}O_5P$: 575.2921, observed: 575.2926.

(1'*R*,2'*R*)-6-hydroxy-5'-methyl-4-pentyl-2'-(prop-1-en-2-yl)-1',2',3',4'-tetrahydro-(1,1'-biphenyl)-2-yl dihydrogen phosphate (2)

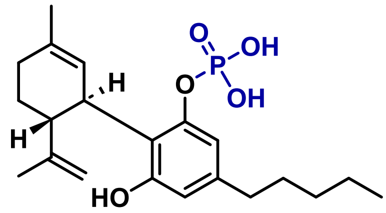

Following general procedure V, compound **2** was obtained by debenzylation of **5** (1.00 g, 1.74 mmol). The crude product was purified by reverse phase flash chromatography (0 – 80% MeOH in H_2O) to afford the title compound (535 mg, 1.36 mmol, **78%**). **Physical state**: White Solid; **^{1}H NMR (600 MHz, CDCl$_3$)** δ 6.97 (br s, 1H), 6.60 (s, 1H), 6.40 (s, 1H), 6.01 (s, 1H), 5.51 (s, 1H), 4.44 (s, 1H), 4.33 (s, 1H), 3.94 – 3.89 (m, 1H), 2.42 – 2.33 (m, 3H), 2.22 – 2.13 (m, 1H), 2.03 – 1.98 (m, 1H), 1.75– 1.70 (m, 1H), 1.69 (s, 3H), 1.58 (s, 3H), 1.48 (s, 2H), 1.34 – 1.10 (m, 4H), 0.88 – 0.78 (m, 3H); **^{13}C NMR (151 MHz, CDCl$_3$)** δ 155.9, 150.7, 147.7, 142.6, 140.0, 124.3, 118.8 (d, J_{CP} = 6.3 Hz), 112.5, 112.4, 111.4, 46.4, 36.9, 35.5, 31.7, 30.7, 30.5, 28.1, 23.7, 22.7,

19.2, 14.2; **^{31}P NMR (243 MHz, $CDCl_3$)** δ -3.99; **HRMS (APCI)** m/z: $[M+H]^+$ calc. for $C_{21}H_{32}O_5P$: 395.1982, observed: 395.1980.

Ammonium benzyl [(1'*R*,2'*R*)-6-hydroxy-5'-methyl-4-pentyl-2'-(prop-1-en-2-yl)-1',2',3',4'-tetrahydro-(1,1'-biphenyl)-2-yl] phosphate (6)

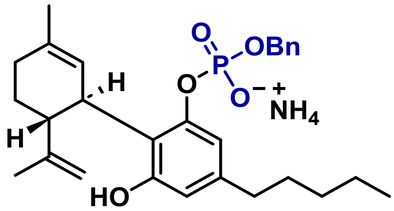

Following general procedure VI, compound **6** was obtained by mono-debenzylation of **5** (1.00 g, 1.74 mmol). Following workup, the crude product was redissolved in an excess amount of DCM:MeOH:$NH_4OH_{(aq)}$ (80:20:3) solution, stirred for 15 minutes and then evaporated in vacuo. Then, the crude product was purified by flash chromatography (0 – 80% MeOH in DCM) to afford the title compound isolated as the ammonium salt (599 mg, 1.24 mmol, **71%**). **Physical state**: White Solid; **^{1}H NMR (600 MHz, $CDCl_3$)** δ 7.21 – 7.11 (m, 5H), 6.71 (s, 1H), 6.40 (s, 1H), 5.30 (s, 1H), 4.87 – 4.82 (m, 2H), 4.44 (s, 1H), 4.35 (s, 1H), 3.96 – 3.89 (m, 1H), 2.43 – 2.38 (m, 1H), 2.38 (t, *J* = 7.8 Hz, 2H), 2.19 – 2.11 (m, 1H), 2.02 – 1.94 (m, 1H), 1.72 – 1.68 (m, 1H), 1.66 (s, 3H), 1.62 (s, 3H), 1.45 (q, *J* = 6.0 Hz, 2H), 1.28 – 1.14 (m, 4H), 0.82 (t, *J* = 7.2 Hz, 3H); **^{13}C NMR (151 MHz, $CDCl_3$)** δ 156.0, 150.8, 147.8, 142.8, 139.9, 137.4 (d, J_{CP} = 8.1 Hz), 128.5 (2C), 127.9 (2C), 127.9, 124.2, 118.2, 112.4, 111.4, 111.5, 68.44 (d, J_{CP} = 5.4 Hz), 46.3, 36.7, 35.6, 31.6, 30.9, 30.4, 28.1, 23.7, 22.7, 19.3, 14.2; **^{31}P NMR (243 MHz, $CDCl_3$)** δ -5.67; **HRMS (APCI)** m/z: $[M+H]^+$ calc. for $C_{28}H_{38}O_5P$: 485.2451, observed: 485.2454.

Complete Scope of TBPP Phosphorylation and Triethylsilane Mediated Debenzylation.

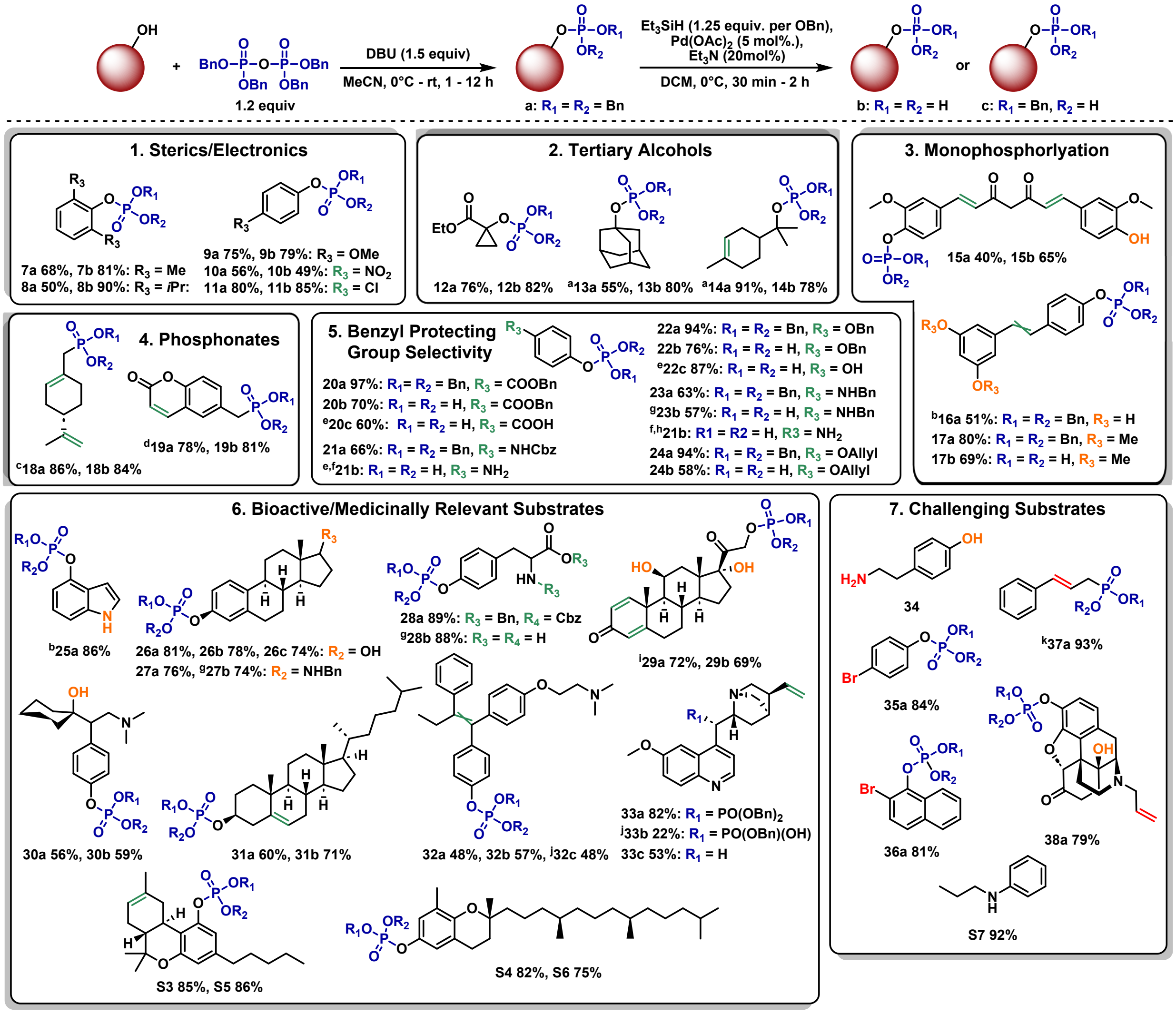

Figure S5. Scope of TBPP based phosphorylation and triethylsilane mediated debenzylation. Unless otherwise stated, a) denotes dibenzyl phosphate, b) denotes fully debenzylated phosphate, and c) denotes mono-benzyl phosphate derivative. Blue represents installed phosph(on)ates, green represents redox sensitive/benzyl protecting groups; orange represents competitive nucleophiles and red represents problematic functionalities. [a]n-Buli used as base. [b]Deprotection results in compound degradation. [c]Synthesized from (*S*)-(-)-perillyl alcohol over two steps. [d]Synthesized from 6-(bromomethyl)-2H-chromen-2-one. [e]4 equivalents of Et_3SiH. [f]Isolated as crude Et_3N salt. [g]Isolated as HCl salt. [h]8 equivalents of Et_3SiH. [i]LDA used as base. [j]Isolated as ammonium salt. [k]Synthesized from cinnamyl bromide.

Preparation of Dibenzyl Phosphate and Phosphonate Substrates

Dibenzyl (2,6-dimethylphenyl) phosphate (7a)

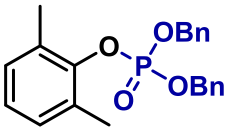

Following general procedure II, compound **7a** was obtained from phosphorylation of 2,6-dimethylphenol (206 mg, 1.69 mmol). The crude product was purified by flash chromatography

(0 – 50% EtOAc in hexanes) to afford the title compound (437 mg, 1.15 mmol, **68%**). **Physical state**: White Solid; **^{1}H NMR (400 MHz, $CDCl_3$)** δ 7.40 – 7.28 (m, 10H), 7.05 – 7.00 (m, 3H), 5.14 – 5.09 (m, 4H), 2.37 (s, 6H); **^{13}C NMR (201 MHz, $CDCl_3$)** δ 135.7 (d, J_{CP} = 7.1 Hz), 130.4 (d, J_{CP} = 3.3 Hz), 129.1 (d, J_{CP} = 1.5 Hz), 128.6, 128.6, 128.0, 125.3 (d, J_{CP} = 1.8 Hz), 69.9 (d, J_{CP} = 5.7 Hz), 17.3. **^{31}P NMR (162 MHz, $CDCl_3$)** δ -5.74; **HRMS (APCI)** m/z: $[M+H]^+$ calc. for $C_{22}H_{24}O_4P$: 383.1407, observed: 383.1407.

Dibenzyl (2,6-diisopropylphenyl) phosphate (8a)

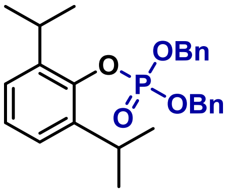

Following general procedure II, compound **8a** was obtained from phosphorylation of 2,6-diisopropylphenol (300 mg, 1.69 mmol). The crude product was purified by flash chromatography (0 – 50% hexane/EtOAc) to afford the title compound (367 mg, 0.845 mmol, **50%**). **Physical state**: White Solid; **^{1}H NMR (400 MHz, $CDCl_3$)** δ 7.38 – 7.27 (m, 10H), 7.18 – 7.12 (m, 3H), 5.12 – 5.06 (m, 4H), 3.51 (hept, J = 6.8 Hz, 2H), 1.19 (d, J = 7.1 Hz, 12H); **^{13}C NMR (125 MHz, $CDCl_3$)** δ ^{13}C NMR (201 MHz, $CDCl_3$) δ 145.7 (d, J_{CP} = 8.8 Hz), 140.6 (d, J_{CP} = 3.3 Hz), 135.7 (d, J_{CP} = 6.9 Hz), 128.6, 128.0, 125.9 (d, J_{CP} = 1.4 Hz), 124.3 (d, J_{CP} = 1.1 Hz), 69.9 (d, J_{CP} = 5.7 Hz), 27.0, 23.6; **^{31}P NMR (162 MHz, $CDCl_3$)** δ -5.68; **HRMS (APCI)** m/z: $[M+H]^+$ calc. for $C_{26}H_{32}O_4P$: 439.2033, observed: 439.2035.

Dibenzyl (4-methoxyphenyl) phosphate (9a)

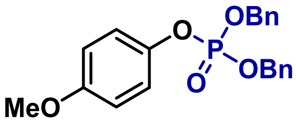

Following general procedure II, compound **9a** was obtained from phosphorylation of 4-methoxyphenol (100 mg, 0.810 mmol). The crude product was purified by flash chromatography (100% hexanes – 40% EtOAc in hexanes) to afford the title compound (233 mg, 0.606 mmol, **75%**). **Physical state**: White Solid; **^{1}H NMR (400 MHz, $CDCl_3$)** δ 7.39 – 7.27 (m, 10H), 7.11 – 7.02 (m, 2H), 6.84 – 6.75 (m, 2H), 5.11 (d, J = 8.2 Hz, 4H), 3.77 (s, 3H); **^{13}C NMR (101 MHz, $CDCl_3$)** δ 156.9 (d, J_{CP} = 1.3 Hz), 144.3 (d, J_{CP} = 7.2 Hz), 135.7 (d, J_{CP} = 7.0 Hz), 128.73, 128.71, 128.1, 121.1 (d, J_{CP} = 4.6 Hz), 114.8 (d, J_{CP} = 1.1 Hz), 70.0 (d, J_{CP} = 5.8 Hz), 55.8; **^{31}P NMR (162 MHz, $CDCl_3$)** δ -5.67; **HRMS (APCI)** m/z: $[M+H]^+$ calc. for $C_{21}H_{22}O_5P$: 385.1199, observed: 385.1194.

Dibenzyl (4-nitrophenyl) phosphate (Compound 10a)

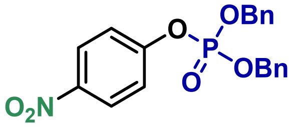

Following general procedure II, compound **10a** was obtained from phosphorylation of 4-nitrophenol (100 mg, 0.719 mmol). Workup procedure was skipped, and the crude product was purified by flash chromatography (5 – 30% EtOAC in hexanes) to afford the title compound (162 mg, 0.406 mmol, **56%**). **Physical state**: Pale Yellow Solid; **^{1}H NMR (400 MHz, $CDCl_3$)** δ 8.18 – 8.09 (m, 2H), 7.39 – 7.28 (m, 10H), 7.25 – 7.16 (m, 2H), 5.21 – 5.08 (m, 4H); **^{13}C NMR (125 MHz, $CDCl_3$)** δ 155.3 (d, J_{CP} = 6.5 Hz), 144.8, 135.1 (d, J_{CP} = 6.2 Hz), 129.1, 128.9, 128.4, 125.7, 120.7 (d, J_{CP} = 5.3 Hz), 70.7 (d, J_{CP} = 5.9 Hz); **^{31}P NMR (162 MHz, $CDCl_3$)** δ -6.90; **HRMS (APCI)** m/z: $[M+H]^+$ calc. for $C_{20}H_{19}NO_6P$: 400.0945, observed: 400.0941. ^{1}H and ^{13}C NMR spectra corresponds to the literature.[6]

Dibenzyl (4-chlorophenyl) phosphate (11a)

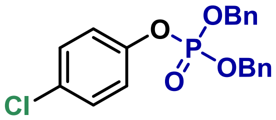

Following general procedure II, compound **11a** was obtained from phosphorylation of 4-chlorophenol (386 mg, 3.00 mmol). The crude product was purified by flash chromatography (0 – 40% EtOAc in hexanes) to afford the title compound (929 mg, 2.39 mmol, **80%**). **Physical state**: White solid; **^{1}H NMR (600 MHz, MeOD)** δ 7.39 – 7.30 (m, 12H), 7.11 – 7.05 (m, 2H), 5.13 (d, J = 9.2 Hz, 4H); **^{13}C NMR (151 MHz, MeOD)** δ 150.4 (d, J_{CP} = 6.8 Hz), 136.7 (d, J_{CP} = 6.1 Hz), 131.8, 130.8, 129.9, 129.7, 129.4, 122.8 (d, J_{CP} = 4.9 Hz), 71.7 (d, J_{CP} = 6.1 Hz); **^{31}P NMR (243 MHz, MeOD)** δ -6.68; **HRMS (APCI)** m/z: $[M+H]^+$ calc. for $C_{20}H_{19}ClO_4P$: 389.0704, observed: 389.0702.

Ethyl 1-((bis(benzyloxy)phosphoryl)oxy)cyclopropane-1-carboxylate (12a)

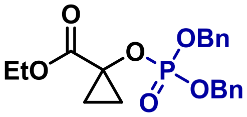

Following general procedure II, compound **12a** was obtained from phosphorylation of ethyl 1-hydroxycyclopropane-1-carboxylate (2.00 g, 15.4 mmol). The crude product was purified by flash chromatography (0 – 20% EtOAC in hexanes) to afford the title compound (4.63 g, 11.7 mmol, **76%**). **Physical state**: Clear oil; **^{1}H NMR (400 MHz, $CDCl_3$)** δ 7.39 – 7.24 (m, 10H), 5.19 – 5.05 (m, 4H), 4.17 (q, J = 7.1 Hz, 2H), 1.47 – 1.32 (m, 4H), 1.22 (t, J = 7.2 Hz, 3H); **^{13}C NMR (101 MHz, $CDCl_3$)** δ 170.9, 135.7 (d, J_{CP} = 7.3 Hz), 128.5, 128.4, 127.9, 69.5 (d, J_{CP} = 5.8 Hz), 61.7,

59.0 (d, J_{CP} = 6.7 Hz), 16.1 (d, J_{CP} = 4.4 Hz), 14.1; **^{31}P NMR (162 MHz, $CDCl_3$)** δ -2.32; **HRMS (APCI)** m/z: $[M+H]^+$ calc. for $C_{20}H_{24}O_6P$: 391.1305, observed: 391.1305.

(3*s*,5*s*,7*s*)-Adamantan-1-yl dibenzyl phosphate (13a)

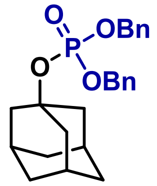

Following general procedure IV, compound **13a** was obtained from phosphorylation of adamantan-1-ol (256 mg, 1.69 mmol) using n-BuLi as base. The crude product was purified by flash chromatography (0 – 50% EtOAC in hexanes) to afford the title compound (385 mg, 0.930 mmol, **55%**). **Physical state**: White Solid; **^{1}H NMR (800 MHz, $CDCl_3$)** δ 7.40 – 7.29 (m, 10H), 5.04 – 4.97 (m, 4H), 2.17 (s, 3H), 2.07 (s, 6H), 1.61 (s, 6H); **^{13}C NMR (201 MHz, $CDCl_3$)** δ 136.3 (d, J_{CP} = 7.4 Hz), 128.5, 128.3, 127.9, 82.9 (d, J_{CP} = 7.8 Hz), 68.8 (d, J_{CP} = 5.7 Hz), 43.4 (d, J_{CP} = 3.9 Hz), 35.7, 31.1; **^{31}P NMR (162 MHz, $CDCl_3$)** δ -5.57; **HRMS (ESI-TOF)** m/z: $[M+H]^+$ calc. for $C_{24}H_{30}O_4P$: 413.1876, observed: 413.1878.

Dibenzyl (2-(4-methylcyclohex-3-en-1-yl)propan-2-yl) phosphate (14a)

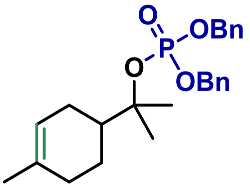

Following general procedure IV, compound **14a** was obtained from phosphorylation of 2-(4-methylcyclohex-3-en-1-yl)propan-2-ol (500 mg, 3.24 mmol) using n-BuLi as base. The crude product was purified by flash chromatography (0 – 50% EtOAc in hexanes) to afford the title compound (1227 mg, 2.95 mmol, **91%**). **Physical state**: White Solid; **^{1}H NMR (800 MHz, $CDCl_3$)** δ 7.38 – 7.28 (m, 10H), 5.34 (d, J = 3.9 Hz, 1H), 5.04 – 4.97 (m, 4H), 2.06 – 1.95 (m, 2H), 1.92 (dd, J = 17.2, 5.1 Hz, 1H), 1.89 – 1.82 (m, 1H), 1.82 – 1.72 (m, 2H), 1.64 (s, 3H), 1.51 – 1.44 (m, 6H), 1.24 (qt, J = 12.6, 6.4 Hz, 1H). **^{13}C NMR (201 MHz, $CDCl_3$)** δ 136.3 (d, J_{CP} = 7.4 Hz), 134.0, 128.5, 128.3, 127.8, 120.1, 88.2 (d, J_{CP} = 8.1 Hz), 68.8 (d, J_{CP} = 5.7 Hz), 44.6 (d, J_{CP} = 7.0 Hz), 30.8, 30.4, 26.7, 25.5 (d, J_{CP} = 1.4 Hz), 24.6 (d, J_{CP} = 2.4 Hz), 23.9, 23.4. **^{31}P NMR (162 MHz, $CDCl_3$)** δ -5.50; **HRMS (ESI-TOF)** m/z: $[M+Na]^+$ calc. for $C_{24}H_{31}O_4PNa$: 437.1852, observed: 437.1857.

Dibenzyl (4-((1*E*,6*E*)-7-(4-hydroxy-3-methoxyphenyl)-3,5-dioxohepta-1,6-dien-1-yl)-2-methoxyphenyl) phosphate (15a)

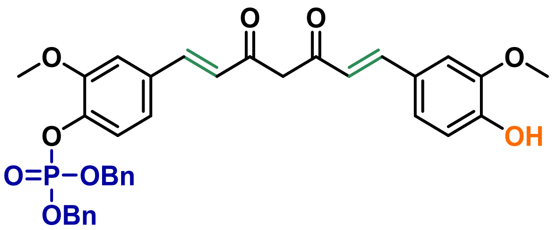

Following general procedure II, compound **15a** was obtained from phosphorylation of curcumin (500 mg, 1.35 mmol). The crude product was purified by flash chromatography (0 – 50% EtOAc in hexanes) to afford the title compound (341 mg, 0.540 mmol, **40%**). **Physical state**: Gummy Yellow Semi-solid; **1H NMR (600 MHz, DMSO-*d*$_6$)** δ 9.71 (s, 1H), 7.60 (dd, *J* = 15.8, 5.0 Hz, 2H), 7.51 (d, *J* = 1.2 Hz, 1H), 7.42 – 7.36 (m, 10H), 7.35 (d, *J* = 2.0 Hz, 1H), 7.29 (dd, *J* = 8.4, 1.9 Hz, 1H), 7.23 (dd, *J* = 8.3, 1.2 Hz, 1H), 7.18 (dd, *J* = 8.3, 2.0 Hz, 1H), 6.95 (d, *J* = 15.9 Hz, 1H), 6.84 (d, *J* = 8.2 Hz, 1H), 6.80 (d, *J* = 15.8 Hz, 1H), 6.14 (s, 1H), 5.19 (d, *J* = 8.1 Hz, 4H), 3.86 (d, *J* = 10.8 Hz, 6H); **13C NMR (151 MHz, DMSO-*d*$_6$)** δ 185.4, 181.9, 151.1 (d, J_{CP} = 4.9 Hz), 150.0, 148.5, 142.0, 140.9 (d, J_{CP} = 7.2 Hz), 139.3, 136.2 (d, J_{CP} = 7.1 Hz), 133.4, 129.0, 129.0, 128.4, 126.7, 125.0, 123.8, 121.9 (d, J_{CP} = 2.8 Hz), 121.7, 121.6, 116.2, 112.8, 111.9, 101.8, 69.8 (d, J_{CP} = 5.6 Hz), 56.5, 56.2; **^{31}P NMR (243 MHz, DMSO-*d*$_6$)** δ -6.16; **HRMS (ESI-TOF)** m/z: [M+H]+ calc for $C_{35}H_{34}O_9P$: 629.1935, observed: 629.1929. 1H NMR spectra correspond to the literature.[7]

(*E*)-Dibenzyl (4-(3,5-dihydroxystyryl)phenyl) phosphate (16a)

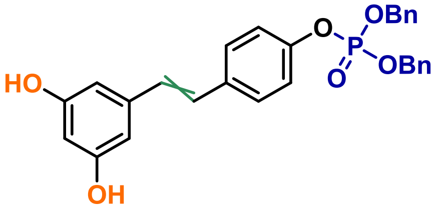

Following general procedure II, compound **GP-CBDPM-011** was obtained from phosphorylation of resveratrol (500 mg, 2.19 mmol) in DMF (10mL/mmol phenol). The crude product was purified by reverse flash chromatography (0 – 70% MeOH in H_2O) (545 mg, 1.12 mmol, **51%**). **Physical state**: Gummy Semi-solid; **^{1}H NMR (400 MHz, $CDCl_3$)** δ 7.26 (s, 10H), 7.06 (d, *J* = 8.6 Hz, 2H), 6.99 – 6.93 (m, 2H), 6.55 (d, *J* = 16.1 Hz, 1H), 6.48 (d, *J* = 16.1 Hz, 1H), 6.39 (s, 1H), 6.37 (d, *J* = 2.1 Hz, 1H), 5.07 (d, *J* = 8.5 Hz, 4H); **^{13}C NMR (151 MHz, DMSO)** δ 158.6, 149.4 (d, J_{CP} = 7.1 Hz), 138.7, 135.6 (d, J_{CP} = 6.7 Hz), 134.3, 129.1, 128.6, 128.5, 128.0, 128.0, 127.9, 126.7, 120.2 (d, J_{CP} = 4.7 Hz), 104.7, 102.4, 69.4 (d, J_{CP} = 5.6 Hz); **^{31}P NMR (162 MHz, $CDCl_3$)** δ -6.59; **HRMS (ESI-TOF)** m/z: $[M+H]^+$ calc. for $C_{28}H_{26}O_6P$: 489.1461, observed: 489.1464.

Note: While complete debenzylation of **16a** could be validated by LCMS and ^{1}H and ^{31}P NMR,

instability of the structure when exposed to light and rapid isomerization between cis and trans resulted in complex and unclear ^{13}C NMR spectra and difficulties in quantification. Therefore, the pterostilbene dibenzyl phosphate derivatives **17a** and deprotected **17b** were synthesized to demonstrate applicability of the reaction to this compound class.

(*E*)-Dibenzyl (4-(3,5-dimethoxystyryl)phenyl) phosphate (17a)

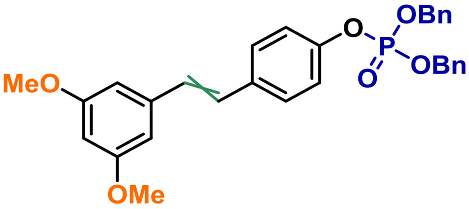

Following general procedure II, compound **17a** was obtained by methylation of pterostilbene (500 mg, 1.95 mmol). The crude product was purified by flash chromatography (0 – 40% EtOAc in hexanes) to afford the title compound (806 mg, 1.56 mmol, **80%**). **Physical state**: White Solid; **^{1}H NMR (600 MHz, CDCl$_3$)** δ 7.46 (d, J = 8.7 Hz, 2H), 7.41 – 7.33 (m, 10H), 7.22 – 7.13 (m, 2H), 7.07 (d, J = 16.3 Hz, 1H), 6.99 (d, J = 16.2 Hz, 1H), 6.70 (d, J = 2.2 Hz, 2H), 6.44 (t, J = 2.2 Hz, 1H), 5.17 (d, J = 8.4 Hz, 4H), 3.86 (s, 6H); **^{13}C NMR (151 MHz, CDCl$_3$)** δ 161.0, 150.0 (d, J_{CP} = 7.1 Hz), 139.2, 135.4 (d, J_{CP} = 6.9 Hz), 134.29 (d, J_{CP} = 1.1 Hz), 128.8, 128.7, 128.6, 128.1, 128.0, 127.8, 120.3 (d, J_{CP} = 4.9 Hz), 104.6, 100.1, 70.1 (d, J_{CP} = 5.9 Hz), 55.4; **^{31}P NMR (243 MHz, CDCl$_3$)** δ -6.20. ^{1}H NMR spectra corresponds to the literature.[3]

(*S*)-1-(chloromethyl)-4-(prop-1-en-2-yl)cyclohex-1-ene (S1)

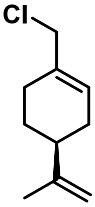

Compound **S1** was prepared according to a modified literature procedure.[4] To an oven dried 150 mL 2-neck round bottom flask, equipped with a stir bar, was added N-chlorosuccinimide (2.38 g, 17.8 mmol, 1.1 equiv.) and anhydrous DCM (32 mL, 2mL/mmol alcohol). The reaction mixture was cooled to −20°C, and dimethyl sulfide (1.4 mL, 19.7 mmol, 1.1 equiv.) was slowly added, followed by the dropwise addition of (*S*)-(-)-perillyl alcohol (2.40 g, 15.8 mmol, 1 equiv.) dissolved in 5 mL anhydrous DCM. The reaction mixture was allowed to slowly warm to 0°C and stirred for 1 hr. The reaction was then quenched with 10 mL of cold saturated brine and dried over anhydrous magnesium sulfate. After filtration, the crude product was purified by flash chromatography (0 – 5% EtOAc in hexanes) to afford the title compound (2.48 g, 14.5 mmol, **92%**). **Physical state**: Clear oil; **^{1}H NMR (600 MHz, CDCl$_3$)** δ 5.83 (s, 1H), 4.74 (br s, 1H), 4.71

(br s, 1H), 4.01 (s, 2H), 2.25 – 2.11 (m, 4H), 2.03 – 1.94 (m, 1H), 1.91 – 1.84 (m, 1H), 1.74 (s, 3H), 1.56 – 1.46 (m, 1H); **^{13}C NMR (151 MHz, $CDCl_3$)** δ 149.5, 134.3, 127.2, 109.0, 50.3, 40.8, 30.8, 27.4, 26.5, 20.9; **HRMS (APCI)** m/z: $[M+H]^+$ calc. for $C_{10}H_{16}Cl$: 171.0935, observed: 171.0936. ^{1}H and ^{13}C NMR spectra corresponds to the literature.[8]

Dibenzyl (*S*)-([4-(prop-1-en-2-yl)cyclohex-1-en-1-yl]methyl)phosphonate (18a)

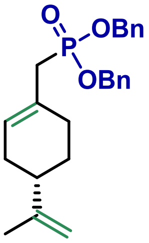

Compound **18a** was prepared according to a modified literature procedure.[4] To an oven dried 150 mL 2-neck round bottom flask, equipped with a stir bar, was added sodium hydride 60% dispersion in mineral oil (300 mg, 7.70 mmol, 1.1 equiv.) and anhydrous DMF (35 mL, 5mL/mmol **S1**). After cooling to 0°C, dibenzyl phosphonate (1.80 g, 7.00 mmol, 1 equiv.) was then added dropwise. The reaction mixture was allowed to warm to rt and stirred for 1 hr to ensure complete deprotonation. Following this, the reaction mixture was cooled to 0°C and a solution of **S1** (1.20 g, 7.00 mmol, 1 equiv.) in 5 mL anhydrous DMF was added slowly. The reaction mixture was allowed to warm to rt and stirred overnight (12 hr). Following complete consumption of the starting material as monitored by TLC, the reaction was cooled to 0°C and quenched with 20 mL of DI water and diluted with ethyl acetate (50 mL). The organic layer was washed with H_2O (50 mL), brine (3 x 50 mL) and dried over anhydrous magnesium sulfate. After filtration, the crude product was purified by flash chromatography (0 – 40% EtOAc in hexanes) to afford the title compound (2.58 g, 6.51 mmol, **93%**). **Physical state**: Clear oil; **^{1}H NMR (600 MHz, $CDCl_3$)** δ 7.40 – 7.28 (m, 10H), 5.06 (ddd, J = 11.9, 8.8, 1.2 Hz, 2H), 5.03 – 4.95 (m, 3H), 4.72 – 4.70 (m, 1H), 4.69 – 4.68 (m, 1H), 2.57 (br s, 1H), 2.54 (br s, 1H), 2.23 – 2.03 (m, 4H), 1.95 – 1.85 (m, 1H), 1.80 – 1.74 (m, 1H), 1.72 (s, 3H), 1.47 – 1.37 (m, 1H); **^{13}C NMR (151 MHz, $CDCl_3$)** δ 149.8, 136.6 (d, J_{CP} = 6.0 Hz), 128.7, 128.6 (2C), 128.6, 128.4 (2C), 128.0 (2C), 128.0 (2C), 127.8 (d, J_{CP} = 11.3 Hz),126.5 (d, J_{CP} = 12.8 Hz), 108.8, 67.4 (d, J_{CP} = 3.9 Hz), 67.4 (d, J_{CP} = 3.9 Hz), 40.6, 35.8, 34.9, 31.0 (d, J_{CP} = 2.8 Hz), 30.1 (d, J_{CP} = 2.8 Hz), 30.9, 27.8, 20.9; **^{31}P NMR (243 MHz, $CDCl_3$)** δ 28.99; **HRMS (APCI)** m/z: $[M+H]^+$ calc. for $C_{24}H_{30}O_3P$: 397.1927, observed: 397.1927. ^{1}H, ^{13}C and ^{31}P NMR spectra corresponds to the literature.[4]

Dibenzyl ((2-oxo-2H-chromen-6-yl)methyl)phosphonate (19a)

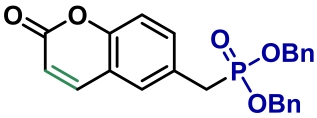

Following a modified version of general procedure II, compound **19a** was obtained by reaction of 6-(bromomethyl)-2H-chromen-2-one (717 mg, 3.00 mmol) and dibenzyl phosphonate (944 mg, 3.60 mmol). The crude product was purified by flash chromatography (0 – 40% EtOAc in DCM) to afford the title compound (984 mg, 2.34 mmol, **78%**). **Physical state**: White Solid; **^{1}H NMR (600 MHz, $CDCl_3$)** δ 7.52 (d, J = 9.5 Hz, 1H), 7.34 – 7.21 (m, 12H), 7.18 (d, J = 8.5 Hz, 1H), 6.40 (d, J = 9.5 Hz, 1H), 4.99 (dd, J = 11.8, 9.5 Hz, 2H), 4.91 (dd, J = 11.8, 8.5 Hz, 2H), 3.17 (s, 1H), 3.14 (s, 1H); **^{13}C NMR (151 MHz, $CDCl_3$)** δ 160.8, 153.2, 153.1, 143.3, 136.2 (d, J_{CP} = 5.6 Hz), 133.4 (d, J_{CP} = 6.4 Hz), 129.0 (d, J_{CP} = 7.0 Hz), 128.9, 128.7, 128.7, 128.2, 127.8 (d, J_{CP} = = 9.3 Hz), 118.9 (d, J_{CP} = = 3.1 Hz), 117.1 (d, J_{CP} = 2.7 Hz), 117.0, 68.0 (d, J_{CP} = 6.7 Hz), 33.9, 33.0; **^{31}P NMR (243 MHz, $CDCl_3$)** δ 26.59; **HRMS (APCI)** m/z: $[M+H]^+$ calc. for $C_{24}H_{22}O_5P$: 421.1199, observed: 421.1199.

Dibenzyl (4-benzyloxyphenyl) phosphate (20a)

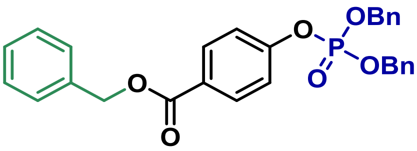

Following general procedure II, compound **20a** was obtained from phosphorylation of benzyl 4-hydroxybenzoate (384 mg, 1.69 mmol). The crude product was purified by flash chromatography (0 – 50% EtOAc in hexanes) to afford the title compound (797 mg, 1.64 mmol, **97%**). **Physical state**: White Solid; **^{1}H NMR (800 MHz, $CDCl_3$)** δ 8.02 (d, J = 8.8 Hz, 2H), 7.46 – 7.39 (m, 4H), 7.38 – 7.31 (m, 11H), 7.18 (d, J = 8.5 Hz, 2H), 5.36 (s, 2H), 5.14 (d, J = 8.7 Hz, 4H); **^{13}C NMR (201 MHz, $CDCl_3$)** δ 165.6, 154.2 (d, J_{CP} = 6.7 Hz), 136.0, 135.2 (d, J_{CP} = 6.6 Hz), 131.7, 128.9, 128.7, 128.7, 128.4, 128.3, 128.2, 127.0, 120.0 (d, J_{CP} = 5.1 Hz), 70.3 (d, J_{CP} = 5.8 Hz), 66.9; **^{31}P NMR (162 MHz, $CDCl_3$)** δ -6.76; **HRMS (ESI-TOF)** m/z: $[M+H]^+$ calc. for $C_{28}H_{26}O_6P$: 489.1467, observed: 489.1463.

Benzyl (4-(((benzyloxy)(hydroxy)phosphoryl)oxy)phenyl)carbamate (21a)

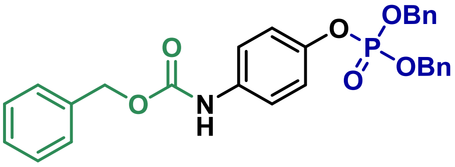

Following general procedure II, compound **21a** was obtained from phosphorylation of benzyl (4-

hydroxyphenyl)carbamate (200 mg, 0.822 mmol). The crude product was purified by flash chromatography (0 – 50% EtOAc in hexanes) to afford the title compound (272 mg, 0.540 mmol, **66%**). **Physical state**: White Solid; **^{1}H NMR (400 MHz, CDCl$_3$)** δ 7.44 – 7.27 (m, 17H), 7.10 – 7.03 (m, 2H), 6.72 (s, 1H), 5.19 (s, 2H), 5.10 (d, *J* = 8.3 Hz, 4H); **^{13}C NMR (125 MHz, CDCl$_3$)** δ 153.5, 146.4 (d, J_{CP} = 7.2 Hz), 136.1, 135.6 (d, J_{CP} = 6.8 Hz), 135.0, 128.79, 128.78, 128.7, 128.6, 128.5, 128.2, 120.8 (d, J_{CP} = 4.8 Hz), 120.0, 70.1 (d, J_{CP} = 5.8 Hz), 67.3.; **^{31}P NMR (162 MHz, CDCl$_3$)** δ -6.02; **HRMS (APCI)** m/z: [M+H]$^+$ calc. for $C_{28}H_{27}NO_6P$: 504.1570, observed: 504.1569.

Dibenzyl (4-(benzyloxy)phenyl) phosphate (22a)

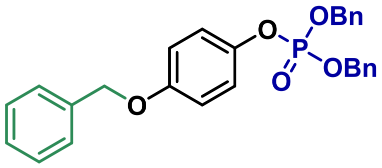

Following general procedure II, compound **22a** was obtained from phosphorylation of 4-(benzyloxy)phenol (337 mg, 1.69 mmol). The crude product was purified by flash chromatography (0 – 50% EtOAc in hexanes) to afford the title compound (725 mg, 1.59 mmol, **94%**). **Physical state**: White Solid; **^{1}H NMR (800 MHz, CDCl$_3$)** δ 7.45 – 7.39 (m, 4H), 7.35 (ddtd, *J* = 15.3, 9.7, 4.8, 1.9 Hz, 11H), 7.10 – 7.07 (m, 2H), 6.91 – 6.87 (m, 2H), 5.13 (d, *J* = 8.3 Hz, 4H), 5.03 (d, *J* = 4.3 Hz, 2H); **^{13}C NMR (201 MHz, CDCl$_3$)** δ 155.9, 144.3 (d, J_{CP} = 7.2 Hz), 136.8, 135.6 (d, J_{CP} = 6.9 Hz), 128.7 (d, J_{CP} = 1.5 Hz), 128.7, 128.1, 128.1, 128.0, 127.6, 121.1 (d, J_{CP} = 4.6 Hz), 115.7, 70.4, 70.0 (d, J_{CP} = 5.8 Hz).**^{31}P NMR (162 MHz, CDCl$_3$)** δ -6.71; **HRMS (ESI-TOF)** m/z: [M+H]$^+$ calc. for $C_{27}H_{26}O_5P$: 461.1512, observed: 461.1509.

Dibenzyl (4-(benzylamino)phenyl) phosphate (23a)

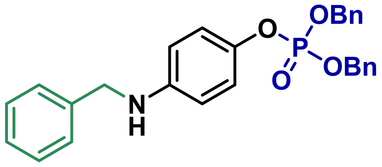

Following general procedure II, compound **23a** was obtained from phosphorylation of 4-(benzylamino)phenol (200 mg, 1.00 mmol). The crude product was purified by flash chromatography (0 – 50% EtOAc in hexanes) to afford the title compound (288 mg, 0.627 mmol; **63%**). **Physical state**: White Solid; **^{1}H NMR (400 MHz, CDCl$_3$)** δ 7.39 – 7.27 (m, 15H), 7.02 – 6.93 (m, 2H), 6.59 – 6.51 (m, 2H), 5.10 (d, J = 8.0 Hz, 4H), 4.29 (s, 2H).; **^{13}C NMR (125 MHz, CDCl$_3$)** δ 145.3, 142.5, 139.0, 135.8 (d, J_{CP} = 7.1 Hz), 128.8, 128.68, 128.65, 128.1, 127.7, 127.5, 121.0 (d, J_{CP} = 4.5 Hz), 113.9, 69.9 (d, J_{CP} = 5.8 Hz), 49.0.; **^{31}P NMR (162 MHz, CDCl$_3$)** δ -5.48; **HRMS (APCI)** m/z: [M+H]$^+$ calc. for $C_{27}H_{27}NO_4P$: 460.1672, observed: 460.1670.

(4-allyloxyphenyl) dibenzyl phosphate (24a)

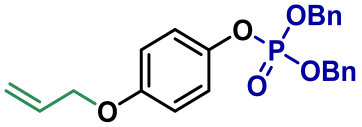

Following general procedure II, compound **24a** was obtained from phosphorylation of 4-allyloxyphenol (500 mg, 3.33 mmol). The crude product was purified by flash chromatography (0 – 50% EtOAc in hexanes) to afford the title compound (1.29 g, 3.13 mmol, **94%**). **Physical state**: Clear oil; **^{1}H NMR (400 MHz, $CDCl_3$)** δ 7.39 – 7.27 (m, 10H), 7.11 – 7.04 (m, 2H), 6.86 – 6.79 (m, 2H), 6.04 (ddt, *J* = 16.7, 10.5, 5.3 Hz, 1H), 5.45 – 5.35 (m, 1H), 5.33 – 5.24 (m, 1H), 5.12 (d, *J* = 8.2 Hz, 4H), 4.52 – 4.47 (m, 2H); **^{13}C NMR (101 MHz, $CDCl_3$)** δ 155.8, 144.3 (d, J_{CP} = 7.2 Hz), 135.6 (d, J_{CP} = 6.9 Hz), 133.1, 128.7, 128.6, 128.1, 121.0 (d, J_{CP} = 4.6 Hz), 117.8, 115.6, 69.9 (d, J_{CP} = 5.8 Hz), 69.3.; **^{31}P NMR (162 MHz, $CDCl_3$)** δ -5.68; **HRMS (ESI-TOF)** m/z: $[M+H]^+$ calc. for $C_{23}H_{24}O_5P$: 411.1356, observed: 411.1353.

Dibenzyl (1*H*-indol-4-yl) phosphate (25a)

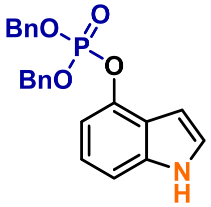

Following general procedure II, compound **25a** was obtained from phosphorylation of 4-hydroxyindole (200 mg, 1.50 mmol). The crude product was purified by flash chromatography (0 – 90% EtOAc in Hexanes) to afford the title compound (507 mg, 1.29 mmol, **86%**). **Physical state**: Light brown Solid; **^{1}H NMR (600 MHz, $CDCl_3$)** δ 8.61 (br s, 1H), 7.33 – 7.30 (m, 10H), 7.22 – 7.16 (m, 1H), 7.10 – 7.09 (m, 1H), 7.07 – 7.04 (m, 1H), 7.03 – 7.01 (m, 1H), 6.63 – 6.58 (m, 1H), 5.18 (dd, *J* = 11.7, 7.7 Hz, 2H), 5.14 (dd, *J* = 11.8, 8.2 Hz, 2H). **^{13}C NMR (151 MHz, $CDCl_3$)** δ 143.6 (d, J_{CP} = 7.2 Hz), 138.1, 135.7 (2C) (d, J_{CP} = 7.2 Hz), 128.7 (4C), 128.6 (2C), 128.1 (4C), 124.5, 122.3, 120.8 (d, J_{CP} = 6.6 Hz), 109.9 (d, J_{CP} = 3.3 Hz), 108.7, 99.6, 70.0 (2C) (d, J_{CP} = 5.5 Hz); **^{31}P NMR (243 MHz, $CDCl_3$)** δ -5.92; **HRMS (ESI-TOF)** m/z: $[M+H]^+$ calc. for $C_{22}H_{21}O_4NP$: 394.1203, observed: 394.1209.

Note: While complete debenzylation of **25a** was attempted and could be validated by LCMS, instability of the compound during isolation resulted in difficulties in quantification and spectroscopic reporting.

Dibenzyl ((13*S*)-17-hydroxy-13-methyl-7,8,9,11,12,13,14,15,16,17-decahydro-6*H*-cyclopenta[*a*]phenanthren-3-yl) phosphate (26a)

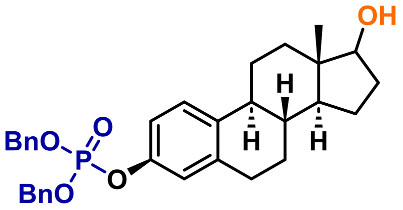

Following general procedure II, compound **26a** was obtained from phosphorylation of estradiol (200 mg, 0.732 mmol). The crude product was purified by flash chromatography (0 – 50% EtOAc in hexanes) to afford the title compound (316 mg, 0.592 mmol, **81%**). **Physical state**: White Solid; **^{1}H NMR (600 MHz, $CDCl_3$)** δ 77.34 (s, 10H), 7.20 (d, J = 8.6 Hz, 1H), 6.94 (ddd, J = 8.6, 2.5, 1.0 Hz, 1H), 6.85 (dd, J = 2.7, 1.2 Hz, 1H), 5.13 (d, J = 8.3 Hz, 4H), 3.71 (t, J = 8.6 Hz, 1H), 2.79 (td, J = 8.1, 4.2 Hz, 2H), 2.39 (s, 1H), 2.28 (dtd, J = 13.4, 4.2, 2.7 Hz, 1H), 2.19 – 2.13 (m, 1H), 2.13 – 2.05 (m, 1H), 1.97 (ddd, J = 12.6, 3.9, 2.7 Hz, 1H), 1.87 (ddt, J = 12.8, 5.8, 2.8 Hz, 1H), 1.71 – 1.63 (m, 1H), 1.55 – 1.22 (m, 6H), 1.16 (ddd, J = 12.3, 11.0, 7.3 Hz, 1H), 0.78 (s, 3H); **^{13}C NMR (151 MHz, $CDCl_3$)** δ 148.4 (d, J_{CP} = 7.1 Hz), 138.6, 137.4, 135.7 (d, J_{CP} = 6.8 Hz), 128.7, 128.6, 128.1, 126.6, 120.1 (d, J_{CP} = 4.5 Hz), 117.2 (d, J_{CP} = 4.8 Hz), 81.9, 69.9 (d, J_{CP} = 5.7 Hz), 50.1, 44.1, 43.23 38.6, 36.7, 30.6, 29.6, 27.1, 26.3, 23.2, 11.1; **^{31}P NMR (243 MHz, $CDCl_3$)** *δ* -5.98; **HRMS (ESI-TOF)** m/z: [M+H]+ calc. for $C_{32}H_{38}O_5P$: 533.2451, observed: 533.2457.

(8R,9S,13S,14S)-17-(benzylamino)-13-methyl-7,8,9,11,12,13,14,15,16,17-decahydro-6H-cyclopenta[a]phenanthren-3-ol (S2)

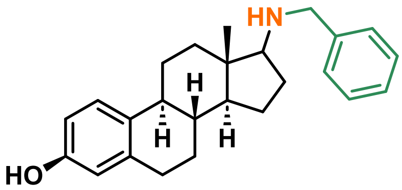

Compound **S2** was prepared according to a modified literature procedure.[9] To a flame-dried round bottom flask equipped with a magnetic stirrer was added estrone (1 g, 3.7 mmol, 1 equiv.) dissolved in dry THF (20 mL, 5mL/mmol estrone) and an equal volume of 1,2-dichloroethane (20 mL, 5mL/mmol estrone). To this solution, benzylamine (1.6 mL, 14.8 mmol, 4 equiv.), glacial acetic acid (0.84 mL, 14.8 mmol, 4 equiv.), and STAB-H (1.95 g, 9.2 mmol, 2.5 equiv.) were then added sequentially. The reaction mixture was stirred at room temperature for 48 hours. After completion, saturated aqueous Na_2CO_3 (40 mL) was added to the mixture and stirred for 10 minutes. The organic layer was extracted by adding double the volume of ethyl acetate. The organic phase was washed with saturated aqueous Na_2CO_3 (3 x 50 mL), water (10 x 50 mL), and brine (1 x 50 mL). It was then dried over anhydrous sodium sulfate, filtered, and concentrated under reduced pressure, yielding a white solid (935 mg, 2.60 mmol, **70%**), which was used

immediately without further purification.

Dibenzyl ((8*R*,9*S*,13*S*,14*S*)-17-(benzylamino)-13-methyl-7,8,9,11,12,13,14,15,16,17-decahydro-6*H*-cyclopenta[a]phenanthren-3-yl) phosphate (27a)

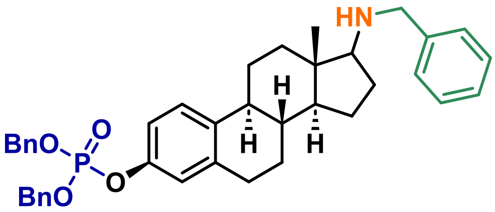

Following a modified version of general procedure II (solvent changed), compound **27a** was obtained from phosphorylation of **S2** (500 mg, 1.38 mmol) in a mixture of THF/MeCN (1:1). The crude product was purified by flash chromatography (0 – 30% EtOAc in DCM) to afford the title compound (653 mg, 1.05 mmol, **76%**). **Physical state**: White Solid; **^{1}H NMR (600 MHz, $CDCl_3$)** δ 7.37 – 7.30 (m, 14H), 7.27 – 7.23 (m, 1H), 7.18 (d, *J* = 8.6 Hz, 1H), 6.91 (dd, *J* = 8.6, 2.6 Hz, 1H), 6.83 (dd, *J* = 2.6, 1.2 Hz, 1H), 5.12 (d, *J* = 8.2 Hz, 4H), 3.98 – 3.71 (m, 2H), 2.85 – 2.70 (m, 2H), 2.67 (t, *J* = 8.6 Hz, 1H), 2.27 (dq, *J* = 13.3, 3.9 Hz, 1H), 2.23 – 2.10 (m, 1H), 2.06 (tdd, *J* = 16.2, 7.4, 3.9 Hz, 2H), 1.86 (ddt, *J* = 12.7, 5.9, 2.7 Hz, 1H), 1.78 – 1.64 (m, 1H), 1.57 – 1.45 (m, 1H), 1.46 – 1.11 (m, 7H), 0.79 (s, 3H); **^{13}C NMR (151 MHz, $CDCl_3$)** δ 148.4 (d, J_{CP} = 7.1 Hz), 138.7, 137.5, 135.7 (d, J_{CP} = 7.1 Hz), 128.7, 128.5, 128.1, 126.9, 126.7, 120.1 (d, J_{CP} = 4.6 Hz), 117.2 (d, J_{CP} = 4.5 Hz), 61.0, 69.9 (d, J_{CP} = 5.7 Hz), 68.4, 52.9, 52.4, 44.2, 43.2, 38.6, 38.2, 29.7, 27.3, 26.5, 23.7, 12.1; **^{31}P NMR (243 MHz, $CDCl_3$)** *δ* -5.93.; **HRMS (ESI-TOF)** m/z: [M+H]+ calc. for $C_{39}H_{45}O_4NP$: 622.3080, observed: 622.3080.

Benzyl (S)-2-{[(benzyloxy)carbonyl]amino}-3-(4-{[bis(benzyloxy)phosphoryl]oxy}phenyl)propanoate (28a)

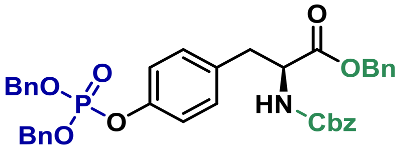

Following general procedure II, compound **28a** was obtained from phosphorylation of benzyl ((benzyloxy)carbonyl)-L-tyrosinate (810 mg, 2.00 mmol). The crude product was purified by flash chromatography (0 – 60% EtOAc in hexanes) to afford the title compound (1.18 g, 1.78 mmol, **89%**). **Physical state**: White Solid; **^{1}H NMR (600 MHz, $CDCl_3$)** δ 7.38 – 7.27 (m, 20H), 6.97 (d, *J* = 8.2 Hz, 2H), 6.89 (d, *J* = 8.2 Hz, 2H), 5.31 – 5.28 (m, 1H), 5.16 (d, *J* = 12.1 Hz, 1H), 5.12 – 5.08 (m, 6H), 4.70 – 4.64 (m, 1H), 3.12 – 3.01 (m, 2H); **^{13}C NMR (151 MHz, $CDCl_3$)** δ 171.2, 155.7, 149.8 (d, J_{CP} = 7.0 Hz), 136.3, 135.5 (2C) (d, J_{CP} = 7.0 Hz), 135.1, 132.5, 130.7 (2C), 128.8 (4C), 128.8 (2C), 128.8 (2C), 128.7 (4C), 128.7 (2C), 128.4, 128.3, 128.2 (4C), 120.2 (2C) (d, J_{CP} = 5.0 Hz), 70.1 (2C) (d, J_{CP} = 5.9 Hz), 67.5 (2C), 67.2, 37.5; **^{31}P NMR (243 MHz, $CDCl_3$)**

δ -6.33; **HRMS (APCI)** m/z: [M+H]+ calc. for $C_{38}H_{38}O_8NP$: 666.2251, observed: 666.2255.

Dibenzyl {2-[(10R,11S,13S,17R)-11,17-dihydroxy-10,13-dimethyl-3-oxo-6,7,8,9,10,11,12,13,14,15,16,17-dodecahydro-3*H*-cyclopenta[a]phenanthren-17-yl]-2-oxoethyl} phosphate (29a)

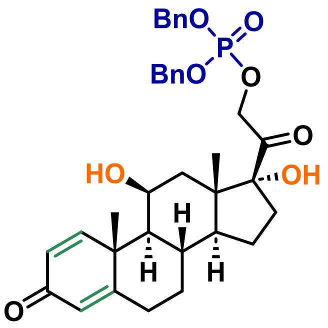

Following general procedure IV, compound **29a** was obtained from phosphorylation of prednisolone (500 mg, 1.38 mmol) using LDA as base. The crude product was purified by flash chromatography (0 – 80% EtOAc in hexanes) to afford the title compound (616 mg, 0.994 mmol, **72%**). **Physical state**: White Solid; **^{1}H NMR (600 MHz, $CDCl_3$)** δ 7.35 – 7.28 (m, 10H), 7.22 (d, *J* = 10.1 Hz, 1H), 6.21 (d, *J* = 9.6 Hz, 1H), 5.97 (s, 1H), 5.15 – 5.05 (m, 2H), 5.06 – 5.00 (m, 4H), 4.81 – 4.73 (m, 1H), 4.37 (s, 1H), 3.92 (s, 1H), 2.68 (t, *J* = 13.1 Hz, 1H), 2.56 – 2.47 (m, 1H), 2.27 (d, *J* = 13.0 Hz, 1H), 2.12 – 2.02 (m, 2H), 2.02 – 1.96 (m, 1H), 1.77 – 1.69 (m, 1H), 1.68 – 1.60 (m, 1H), 1.57 – 1.47 (m, 2H), 1.41 – 1.34 (m, 4H), 1.09 – 1.02 (m, 1H), 1.00 – 0.96 (m, 1H), 0.91 (s, 3H); **^{13}C NMR (151 MHz, $CDCl_3$)** δ 205.2 (d, J_{CP} = 3.1 Hz), 186.8, 170.5, 156.6, 135.8 (d, J_{CP} = 7.6 Hz), 135.7 (d, J_{CP} = 7.2 Hz), 128.75, 128.73, 128.1 (d, J_{CP} = 8.8 Hz), 127.9, 122.5, 89.7, 70.99, 70.95, 70.1, 69.9 (d, J_{CP} = 5.6 Hz), 69.8 (d, J_{CP} = 5.6 Hz), 55.4, 51.4, 47.8, 44.3, 39.7, 34.5, 34.2, 32.1, 31.4, 24.0, 21.2, 17.3; **^{31}P NMR (243 MHz, $CDCl_3$)** δ -1.30; **HRMS (ESI-TOF)** m/z: $[M+H]^+$ calc. for $C_{35}H_{42}O_8P$: 621.2612, observed: 621.2624.

Dibenzyl {4-[2-(dimethylamino)-1-(1-hydroxycyclohexyl)ethyl]phenyl} phosphate (30a)

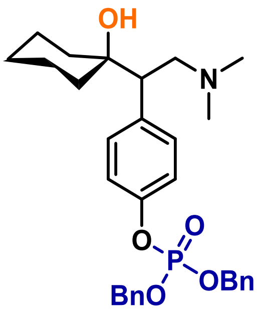

Following general procedure II, compound **30a** was obtained from phosphorylation of desvenlafaxine (500 mg, 1.90 mmol). The crude product was purified by flash chromatography (0 – 100% DCM:MeOH:$NH_4OH_{(aq)}$ (80:20:3) in DCM) and then reverse phase column

chromatography (10 – 100% MeOH in H_2O) to afford the title compound (553 mg, 1.06 mmol, **56%**). **Physical state**: White Solid; **^{1}H NMR (600 MHz, MeOD)** δ 7.56 – 7.48 (m, 5H), 7.36 – 7.19 (m, 9H), 4.97 (d, *J* = 6.4 Hz, 2H), 4.48 – 4.39 (m, 2H), 4.07 – 3.96 (m, 2H), 3.19 (d, *J* = 8.4 Hz, 1H), 2.91 (s, 3H), 2.69 (s, 3H), 1.72 – 1.61 (m, 2H), 1.60 – 1.43 (m, 4H), 1.35 – 1.21 (m, 3H),1.08 – 1.00 (m, 1H), -OH signal not observed in spectra; **^{13}C NMR (151 MHz, MeOD)** δ 154.0 (d, J_{CP} = 6.6 Hz), 139.4 (d, J_{CP} = 8.3 Hz), 135.3 (2C), 134.3 (2C), 131.9, 130.3 (2C), 129.3 (4C), 128.8, 128.6, 128.4 (4C), 121.6 (2C), 74.6, 70.7, 68.8, 68.7, 37.4, 34.0, 26.5, 22.8, 22.4. **^{31}P NMR (243 MHz, MeOD)** δ -4.76; **HRMS (APCI)** m/z: $[M+H]^+$ calc. for $C_{30}H_{39}O_5NP$: 524.2560, observed: 524.2565.

Dibenzyl [(3*S*,8*S*,9*S*,10*R*,13*R*,14*S*,17*R*)-10,13-dimethyl-17-((*R*)-6-methylheptan-2-yl)-2,3,4,7,8,9,10,11,12,13,14,15,16,17-tetradecahydro-1H-cyclopenta[a]phenanthren-3-yl] phosphate (31a)

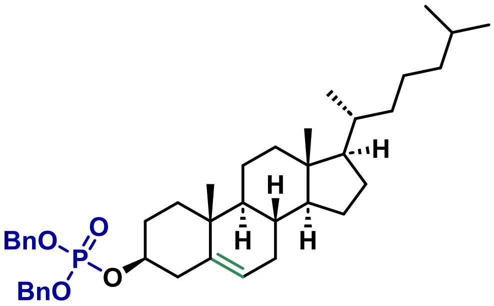

Following general procedure IV, compound **31a** was obtained from phosphorylation of cholesterol (500 mg, 1.29 mmol) using n-BuLi as base in a mixture of THF/MeCN (2:1). The crude product was purified by flash chromatography (0 – 25% EtOAc in hexanes) to afford the title compound (502 mg, 0.774 mmol, **60%**). **Physical state**: White Solid; **^{1}H NMR (600 MHz, $CDCl_3$)** δ 7.37 – 7.30 (m, 10H), 5.31 (dt, *J* = 5.7, 1.9 Hz, 1H), 5.08 – 4.97 (m, 4H), 4.19 (dddd, *J* = 17.1, 11.0, 6.4, 4.6 Hz, 1H), 2.36 (tt, *J* = 9.3, 5.0 Hz, 2H), 2.03 – 1.88 (m, 3H), 1.86 – 1.74 (m, 2H), 1.67 – 1.60 (m, 2H), 1.59 – 1.30 (m, 8H), 1.29 – 1.21 (m, 1H), 1.21 – 0.97 (m, 12H), 0.91 (d, *J* = 6.5 Hz, 4H), 0.87 (dd, *J* = 6.6, 2.8 Hz, 6H), 0.67 (s, 3H); **^{13}C NMR (151 MHz, $CDCl_3$)** δ 139.5, 136.2 (d, J_{CP} = 6.9 Hz), 128.7, 128.5, 128.0, 123.1, 78.8 (d, J_{CP} = 6.1 Hz), 69.2 (d, J_{CP} = 5.5 Hz), 56.8, 56.3, 50.1, 42.4, 339.9 (d, J_{CP} = 4.8 Hz), 39.8, 39.6, 37.0, 36.5, 36.3, 35.9, 32.0, 31.9, 29.6 (d, J_{CP} = 4.4 Hz), 28.4, 28.1, 24.4, 24.0, 23.0, 22.7, 21.2, 19.4, 18.8, 12.0; **^{31}P NMR (243 MHz, $CDCl_3$)** δ -1.83; **HRMS (ESI-TOF)** m/z: $[M+H]^+$ calc. for $C_{41}H_{60}O_4P$: 647.4223, observed: 647.4227.

(*E/Z*)-Dibenzyl (4-(1-(4-(2-(dimethylamino)ethoxy)phenyl)-2-phenylbut-1-en-1-yl)phenyl) phosphate (32a)

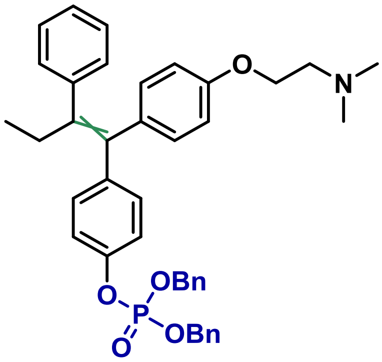

Following general procedure II, compound **32a** was obtained from phosphorylation of 4-hydroxytamoxifen (200 mg, 1.30 mmol). The crude product was purified by flash chromatography (0 – 100% DCM:MeOH:$NH_4OH_{(aq)}$ (80:20:3) in DCM) and then reverse-phase column chromatography (10 – 100% MeOH in H_2O) to afford the title compound (161 mg, 0.249 mmol; **48%, *E*/*Z* = 1/1**). **Physical state**: White Solid; **^{1}H NMR (400 MHz, MeOD)** δ 7.65 – 7.47 (m, 5H), 7.37 – 6.96 (m, 14H), 6.89 – 6.78 (m, 2H), 6.78 – 6.70 (m, 1H), 6.67 – 6.61 (m, 1H), 4.99 (d, J = 6.4 Hz, 1H), 4.85 (d, J = 6.2 Hz, 1H), 4.66 (s, 1H), 4.59 (s, 1H), 4.54 (s, 1H), 4.36 (s, 1H), 3.85 – 3.79 (m, 1H), 3.74 – 3.68 (m, 1H), 3.35 (s, 3H), 3.15 (s, 3H), 3.07 (s, 3H), 2.52 – 2.41 (m, 2H), 0.95 – 0.86 (m, 3H); **^{13}C NMR (125 MHz, MeOD)** δ 157.7, 156.8, 153.3, 153.2, 152.41, 152.36, 143.8, 143.5, 143.07, 143.05, 139.8, 139.5, 139.43, 139.38, 139.1, 138.8, 138.40, 134.36, 134.3, 133.2, 132.6, 132.1, 132.0, 131.9, 131.2, 130.9, 130.8, 130.43, 130.39, 129.3, 129.2, 128.99, 128.95, 128.8, 128.7, 128.63, 128.59, 128.55, 128.5, 127.3, 127.2, 121.1, 121.0, 120.2, 120.1, 115.4, 114.6, 70.5, 70.4, 68.79, 68.76, 68.72, 68.68, 64.53, 64.47, 62.9, 62.7, 51.3, 51.2, 30.0, 29.9, 13.9, 13.8.; **^{31}P NMR (162 MHz, MeOD)** δ -4.72, -4.95; **HRMS (APCI)** m/z: $[M+H]^+$ calc. for $C_{40}H_{43}NO_5P$: 648.2873, observed: 648.2873.

Dibenzyl ((*S*)-(6-methoxyquinolin-4-yl)((1*R*,2*R*,4*R*,5*S*)-5-vinylquinuclidin-2-yl)methyl) phosphate (33a)

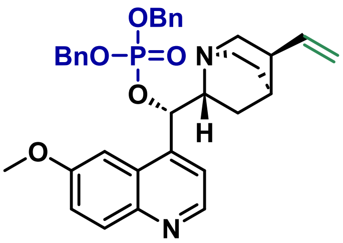

Following general procedure IV, compound **33a** was obtained from phosphorylation of quinine (1000 mg, 3.08 mmol) using n-BuLi as base. The crude product was purified by flash chromatography (0 – 100% DCM:MeOH:$NH_4OH_{(aq)}$ (80:20:3) in DCM) to afford the title compound (813 mg, 2.53 mmol, **82%**). **Physical state**: Light yellow semisolid; **^{1}H NMR (400 MHz, $CDCl_3$)** δ

8.69 (d, J = 4.3 Hz, 1H), 8.00 (d, J = 9.4 Hz, 1H), 7.44 – 7.25 (m, 6H), 7.23 – 7.11 (m, 5H), 6.89 (d, J = 7.1 Hz, 2H), 6.18 – 5.72 (m, 2H), 5.04 – 4.94 (m, 3H), 4.87 (dd, J = 11.7, 8.1 Hz, 1H), 4.74 – 4.61 (m, 2H), 3.86 (s, 3H), 3.49 – 3.03 (m, 2H), 3.03 – 2.90 (m, 1H), 2.72 – 2.41 (m, 3H), 2.23 (s, 1H), 1.83 (s, 2H), 1.71 – 1.58 (m, 2H), 1.53 – 1.42 (m, 1H); **^{13}C NMR (101 MHz, $CDCl_3$)** δ 157.9, 147.4, 144.8, 141.8, 135.5 (d, J_{CP} = 7.0 Hz), 135.3 (d, J_{CP} = 7.3 Hz), 131.9, 128.6, 128.5, 128.4, 127.8, 127.5, 126.7, 121.9, 114.5, 77.2, 69.4 (d, J_{CP} = 5.6 Hz), 69.2 (d, J_{CP} = 5.6 Hz), 56.5, 55.7, 42.4, 39.74, 27.6, 27.5. **^{31}P NMR (162 MHz, $CDCl_3$)** δ -1.23; **HRMS (APCI-TOF)** m/z: $[M+H]^+$ calc. for $C_{34}H_{38}O_5N_2P$: 585.2513, observed: 585.2512.

Dibenzyl (4-chlorophenyl) phosphate (35a)

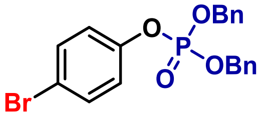

Following general procedure II, compound **35a** was obtained from phosphorylation of 4-bromophenol (519 mg, 3.00 mmol). The crude product was purified by flash chromatography (0 – 40% EtOAc in hexanes) to afford the title compound (1.09 g, 2.52 mmol, **84%**). **Physical state**: White solid; **^{1}H NMR (600 MHz, $CDCl_3$)** δ 7.40 – 7.29 (m, 12H), 7.03 – 6.97 (m, 2H), 5.12 (d, J = 8.7 Hz, 4H); **^{13}C NMR (151 MHz, $CDCl_3$)** δ 149.7 (d, J_{CP} = 6.8 Hz), 135.4 (2C) (d, J_{CP} = 6.6 Hz), 132.8 (2C), 128.9 (2C), 128.8 (4C), 128.2 (4C), 122.0 (2C) (d, J_{CP} = 4.9 Hz), 118.2, 70.3 (2C) (d, J_{CP} = 5.8 Hz); **^{31}P NMR (243 MHz, $CDCl_3$)** δ -6.34; **HRMS (ESI-TOF)** m/z: $[M+H]^+$ calc. for $C_{20}H_{19}O_4BrP$: 433.0199, observed: 433.0202.

Dibenzyl (7-bromoquinolin-8-yl) phosphate (36a)

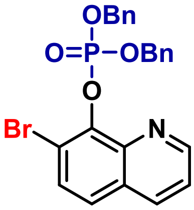

Following general procedure II, compound **36a** was obtained from phosphorylation of 7-bromoquinolin-8-ol (672 mg, 3.00 mmol). The crude product was purified by flash chromatography (0 – 80% EtOAc in hexanes) to afford the title compound (1.18 g, 2.43 mmol, **81%**). **Physical state**: White solid; **^{1}H NMR (600 MHz, $CDCl_3$)** δ 8.85 (dd, J = 4.2, 1.7 Hz, 1H), 8.14 (dd, J = 8.3, 1.7 Hz, 1H), 7.70 (dd, J = 8.8, 0.9 Hz, 1H), 7.55 (dd, J = 8.8, 1.4 Hz, 1H), 7.46 – 7.44 (m, 5H), 7.38 – 7.33 (m, 6H), 5.54 (dd, J = 11.8, 7.4 Hz, 2H), 5.47 (dd, J = 11.8, 6.7 Hz, 2H)l; **^{13}C NMR (151 MHz, $CDCl_3$)** δ 150.7, 145.7 (d, J_{CP} = 7.6 Hz), 142.2 (d, J_{CP} = 2.4 Hz), 136.3 (d, J_{CP} = 8.4 Hz), 136.0, 130.8 (d, J_{CP} = 1.9 Hz), 128.8, 128.7, 128.6 (4C), 128.4, 128.0, 127.9 (4C), 125.6 (d, J_{CP} = 2.0 Hz), 122.1, 115.8 (d, J_{CP} = 6.5 Hz), 70.4 (2C) (d, J_{CP} = 5.9 Hz); **^{31}P NMR**

(243 MHz, CDCl$_3$) δ -7.09; **HRMS (APCI)** m/z: $[M+H]^+$ calc. for $C_{23}H_{20}O_4NBrP$: 484.0308, observed: 484.0312.

Dibenzyl cinnamylphosphonate (37a)

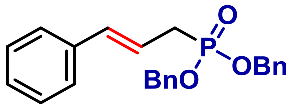

To an oven dried 150 mL 2-neck round bottom flask, equipped with a stir bar, was added sodium hydride 60% dispersion in mineral oil (224 mg, 5.61 mmol, 1.1 equiv.) and anhydrous DMF (30 mL, 5mL/mmol cinnamyl bromide). After cooling to 0°C, dibenzyl phosphonate (1.34 g, 5.10 mmol, 1 equiv.) was then added dropwise. The reaction mixture was allowed to warm to rt and stirred for 1 hr to ensure complete deprotonation. Following this, the reaction mixture was cooled to 0°C and a solution of cinnamyl bromide (1.00 g, 5.10 mmol) in 5 mL anhydrous DMF was added slowly. The reaction mixture was allowed to warm to rt and stirred overnight (12 hr). Following complete consumption of the starting material as monitored by TLC, the reaction was cooled to 0°C and quenched with 20 mL of DI water and diluted with ethyl acetate (50 mL). The organic layer was washed with H_2O (50 mL), brine (3 x 50 mL) and dried over anhydrous magnesium sulfate. After filtration, the crude product was purified by flash chromatography (0 – 40% EtOAc in hexanes) to afford the title compound (1.79 g, 4.74 mmol, **93%**). **Physical state**: White solid; **^{1}H NMR (600 MHz, CDCl$_3$)** δ 7.40 – 7.19 (m, 15H), 6.42 (dd, *J* = 15.7, 1.4 Hz, 1H), 6.10 (dt, *J* = 15.7, 7.4 Hz, 1H), 5.10 (dd, *J* = 11.9, 8.8 Hz, 2H), 5.01 (dd, *J* = 11.9, 8.8 Hz, 2H), 2.79 (dd, *J* = 7.6, 1.4 Hz, 1H), 2.75 (dd, *J* = 7.4, 1.4 Hz, 1H); **^{13}C NMR (151 MHz, CDCl$_3$)** δ 136.8 (d, J_{CP} = 3.6 Hz), 136.5 (d, J_{CP} = 5.8 Hz), 135.1 (d, J_{CP} = 15.2 Hz), 128.7, 128.6, 128.5, 128.1, 127.7, 126.4 (d, J_{CP} = 2.0 Hz), 118.4 (d, J_{CP} = 12.2 Hz), 67.7 (d, J_{CP} = 6.6 Hz), 32.0, 31.0; **^{31}P NMR (243 MHz, CDCl$_3$)** δ 27.93; **HRMS (APCI)** m/z: $[M+H]^+$ calc. for $C_{23}H_{24}O_3P$: 379.1458, observed: 379.1458.

(4*R*,4a*S*,7a*R*,12b*S*)-3-allyl-4a-hydroxy-7-oxo-2,3,4,4a,5,6,7,7a-octahydro-1*H*-4,12-methanobenzofuro[3,2-e]isoquinolin-9-yl dibenzyl phosphate (38a)

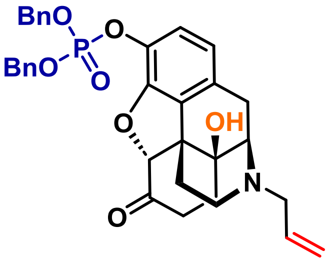

Following a modified general procedure II, compound **38a** was obtained from phosphorylation of naloxone hydrochloride (728 mg, 2.00 mmol) using 2.5 equivalent of DBU. The crude product

was purified by flash chromatography (0 – 100% EtOAc in hexanes) to afford the title compound (928 mg, 1.58 mmol, **79%**). **Physical state**: White Solid; **^{1}H NMR (600 MHz, MeOD)** δ 7.40 – 7.28 (m, 10H), 7.06 (d, J = 8.3 Hz, 1H), 6.64 (d, J = 8.3 Hz, 1H), 5.86 – 5.76 (m, 1H), 5.27 – 5.17 (m, 6H), 4.67 (s, 1H), 3.19 – 3.06 (m, 3H), 3.05 – 2.95 (m, 2H), 2.62 – 2.53 (m, 2H), 2.35 (td, J = 12.7, 5.3 Hz, 1H), 2.28 (dt, J = 14.7, 3.2 Hz, 1H), 2.08 (td, J = 12.2, 3.8 Hz, 1H), 1.89 – 1.82 (m, 1H), 1.59 (td, J = 13.9, 3.4 Hz, 1H), 1.52 – 1.43 (m, 1H), -OH signal not observed in spectra; **^{13}C NMR (151 MHz, MeOD)** δ 207.5, 146.9 (d, J_{CP} = 5.5 Hz), 135.9 (d, J_{CP} = 3.3 Hz), 135.85 (d, J_{CP} = 3.9 Hz), 135.10, 133.0 (d, J_{CP} = 7.2 Hz), 130.5, 129.8, 128.6 (4C), 128.5 (2C), 128.2 (2C), 128.1 (2C), 122.2 (d, J_{CP} = 3.3 Hz), 119.8, 118.4, 90.8, 70.2, 70.1 (d, J_{CP} = 5.5 Hz), 70.1 (d, J_{CP} = 6.1 Hz), 62.2, 57.8, 50.8, 43.3, 36.2, 31.2, 30.7, 23.0; **^{31}P NMR (243 MHz, MeOD)** δ -10.13; **HRMS (ESI-TOF)** m/z: $[M+H]^+$ calc. for $C_{33}H_{35}O_7NP$: 588.2146, observed: 588.2157. ^{1}H NMR spectra corresponds to the literature.[10]

Dibenzyl ((6aR,10aR)-6,6,9-trimethyl-3-pentyl-6a,7,10,10a-tetrahydro-6H-benzo[c]chromen-1-yl) phosphate (S3)

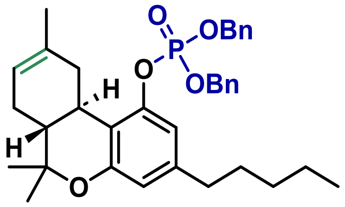

Following general procedure II, compound **S3** was obtained from phosphorylation of Δ-8-tetrahydrocannabinol (500 mg, 1.51 mmol). The crude product was purified by flash chromatography (0 – 20% EtOAc in hexanes) to afford the title compound (741 mg, 1.29 mmol, **85%**). **Physical state**: White Solid; **^{1}H NMR (600 MHz, $CDCl_3$)** δ 7.38 – 7.23 (m, 10H), 6.68 (s, 1H), 6.48 (s, 1H), 5.42 – 5.37 (m, 1H), 5.18 – 5.01 (m, 4H), 3.02 (dd, J = 16.9, 4.6 Hz, 1H), 2.72 (td, J = 11.0, 4.9 Hz, 1H), 2.44 (dd, J = 8.8, 6.3 Hz, 2H), 2.13 – 2.06 (m, 1H), 1.88 – 1.79 (m, 1H), 1.76 – 1.70 (m, 2H), 1.61 (s, 3H), 1.56 – 1.50 (m, 2H), 1.34 (s, 3H), 1.33 – 1.25 (m, 4H), 0.95 (s, 3H), 0.88 (t, J = 7.1 Hz, 3H); **^{13}C NMR (151 MHz, $CDCl_3$)** δ 154.8, 149.8 (d, J_{CP} = 6.6 Hz), 143.0, 135.7 (d, J_{CP} = 3.9 Hz), 135.7 (d, J_{CP} = 3.9 Hz), 134.5, 128.7 (4C), 128.7 (4C), 128.7 (2C), 128.1, 128.0, 119.6, 115.2 (d, J_{CP} = 7.2 Hz), 114.5, 112.3 (d, J_{CP} = 2.8 Hz), 70.0, 69.9 (2C) (d, J_{CP} = 5.5 Hz), 44.9, 36.3, 35.6, 31.8, 31.7, 30.7, 27.9, 27.6, 23.6, 22.7, 18.5, 14.2; **^{31}P NMR (243 MHz, $CDCl_3$)** δ -6.30; **HRMS (ESI-TOF)** m/z: $[M+H]^+$ calc. for $C_{35}H_{44}O_5P$: 575.2921, observed: 575.2933.

Dibenzyl ((R)-2,8-dimethyl-2-((4R,8R)-4,8,12-trimethyltridecyl)chroman-6-yl) phosphate (S4)

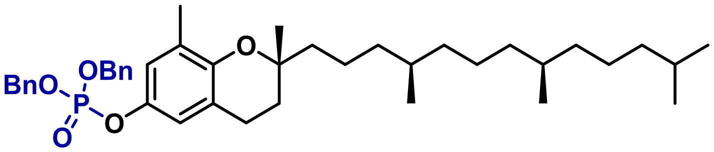

Following general procedure II, compound **S4** was obtained from phosphorylation of δ-Tocopherol (300 mg, 0.69 mmol). The crude product was purified by flash chromatography (0 – 20% EtOAc in hexanes) to afford the title compound (379 mg, 0.566 mmol, **82%**). **Physical state**: White Solid; **^{1}H NMR (600 MHz, $CDCl_3$)** δ 7.37 – 7.31 (m, 10H), 6.73 (dt, *J* = 3.0, 1.0 Hz, 1H), 6.72 – 6.68 (m, 1H), 5.16 – 5.08 (m, 4H), 2.72 – 2.60 (m, 2H), 2.10 (s, 3H), 1.78 (ddd, *J* = 13.9, 7.8, 6.4 Hz, 1H), 1.72 (dt, *J* = 13.3, 6.5 Hz, 1H), 1.61 – 1.50 (m, 3H), 1.49 – 1.03 (m, 21H), 0.90 – 0.82 (m, 12H); **^{13}C NMR (151 MHz, $CDCl_3$)** δ 149.4 (d, J_{CP} = 1.4 Hz), 142.5 (d, J_{CP} = 7.4 Hz), 135.8 (d, J_{CP} = 7.0 Hz), 128.7, 128.6, 128.6, 128.1, 127.6, 121.2, 120.0 (d, J_{CP} = 4.6 Hz), 117.8 (d, J_{CP} = 4.3 Hz), 76.2, 69.8 (d, J_{CP} = 5.7 Hz), 40.2, 39.5, 37.6, 37.5, 37.4, 32.9, 32.8, 31.1, 28.1, 24.9, 24.6, 24.2, 22.8, 22.7, 22.5, 21.1, 19.9, 19.8, 16.2; **^{31}P NMR (243 MHz, $CDCl_3$)** δ -5.55; **HRMS (ESI-TOF)** m/z: $[M+H]^+$ calc. for $C_{41}H_{60}O_4P$: 647.4223, observed: 647.4227.

Debenzylation of Prepared Dibenzyl Phosphate and Phosphonate Substrates

2,6-Dimethylphenyl phosphate (7b)

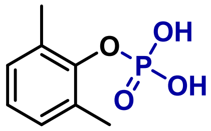

Following general procedure V, compound **7b** was obtained from debenzylation of **7a** (174 mg, 0.456 mmol). The crude product was purified by reverse flash chromatography (0 – 80% MeOH in H_2O) to afford the title compound (75 mg, 0.370 mmol, **81%**). **Physical state**: White Solid; **^{1}H NMR (400 MHz, DMSO-*d$_6$*)** δ 6.98 (d, *J* = 7.4 Hz, 2H), 6.92 – 6.86 (m, 1H), 2.26 (s, 6H); **^{13}C NMR (101 MHz, DMSO-*d$_6$*)** δ 149.4 (d, J_{CP} = 7.5 Hz), 130.4 (d, J_{CP} = 2.5 Hz), 128.4, 123.6, 17.2; **^{31}P NMR (162 MHz, DMSO-*d$_6$*)** δ -5.46; **HRMS (APCI)** m/z: $[M+H]^+$ calc. for $C_8H_{12}O_4P$: 203.0468, observed: 203.0467.

2,6-diisopropylphenyl phosphate (8b)

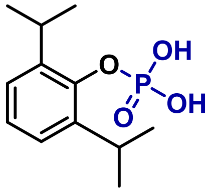

Following general procedure V, compound **8b** was obtained from debenzylation of **8a** (200 mg,

0.456 mmol). The crude product was purified by reverse flash chromatography (0 – 80% MeOH in H_2O) to afford the title compound (106 mg, 0.410 mmol, **90%**). **Physical state**: White Solid; **^{1}H NMR (400 MHz, DMSO-*d*$_6$)** δ 7.11 – 7.02 (m, 3H), 3.62 – 3.50 (m, 2H), 1.12 (d, *J* = 6.8 Hz, 12H); **^{13}C NMR (101 MHz, DMSO-*d*$_6$)** δ 146.6, 140.6 (d, J_{CP} = 2.8 Hz), 124.4, 123.6, 26.1, 23.6; **^{31}P NMR (162 MHz, DMSO-*d*$_6$)** δ -5.77; **HRMS (APCI)** m/z: $[M+H]^+$ calc. for $C_{12}H_{20}O_4P$: 259.1094, observed: 259.1093. ^{1}H NMR spectra corresponds to the literature.[11]

4-Methoxyphenyl phosphate (9b)

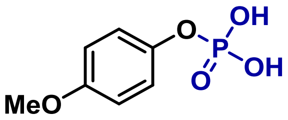

Following general procedure V, compound **9b** was obtained from debenzylation of **9a** (100 mg, 0.260 mmol). The crude product was purified by reverse phase flash chromatography (0 – 80% MeOH in H_2O) to afford the title compound (42 mg, 0.21 mmol, **79%**). **Physical state**: White Solid; **^{1}H NMR (400 MHz, DMSO-*d*$_6$)** δ 7.11 – 7.02 (m, 2H), 6.92 – 6.83 (m, 2H), 3.71 (s, 3H); **^{13}C NMR (125 MHz, DMSO-*d*$_6$)** δ 155.5, 145.2 (d, J_{CP} = 6.5 Hz), 121.0 (d, J_{CP} = 4.5 Hz), 114.3, 55.4; **^{31}P NMR (162 MHz, DMSO-*d*$_6$)** δ -5.62; **HRMS (APCI)** m/z: $[M-H]^-$ calc. for $C_7H_8O_5P$: 203.0115, observed: 203.0118.

4-Nitrophenyl phosphate (10b)

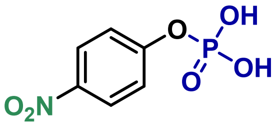

Following general procedure V, compound **10b** was obtained from debenzylation of **10a** (200 mg, 0.501 mmol). The crude product was purified by flash chromatography (0 – 80% MeOH in H_2O) to afford the title compound (56 mg, 0.240 mmol, **49%**). **Physical state**: White Solid; **^{1}H NMR (400 MHz, DMSO-*d*$_6$)** δ 8.30 – 8.21 (m, 2H), 7.44 – 7.36 (m, 2H); **^{13}C NMR (125 MHz, DMSO-*d*$_6$)** δ 157.1 (d, J_{CP} = 5.6 Hz), 143.2, 125.6, 120.6 (d, J_{CP} = 5.3 Hz); **^{31}P NMR (162 MHz, DMSO-*d*$_6$)** δ -6.80; **HRMS (APCI)** m/z: $[M-H]^-$ calc. for $C_6H_5NO_6P$: 217.9860, observed: 217.9859.

4-Chlorophenyl phosphate (11b)

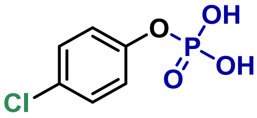

Following general procedure V, compound **11b** was obtained from debenzylation of **11a** (200 mg, 0.510 mmol). The crude product was purified by reverse phase flash chromatography (0 – 80% MeOH in H_2O) to afford the title compound (90 mg, 0.434 mmol, **85%**). **Physical state**:

White Solid; **^{1}H NMR (600 MHz, MeOD)** δ 7.33 – 7.26 (m, 2H), 7.25 – 7.15 (m, 2H); **^{31}P NMR (162 MHz, DMSO-*d*$_6$)** δ -5.87; **HRMS (APCI)** m/z: [M+H]$^+$ calc. for $C_6H_7O_4ClP$: 208.9765, observed: 208.9766. ^{1}H and ^{31}P NMR spectra corresponds to the literature.[12, 13]

Ethyl 1-(phosphonooxy)cyclopropane-1-carboxylate (12b)

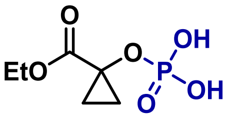

Following general procedure V, compound **12b** was obtained from debenzylation of **12a** (500 mg, 1.26 mmol). The crude product was purified by reverse phase flash chromatography (0 – 80% MeOH in H_2O) to afford the title compound (217 mg, 1.03 mmol, **82%**). **Physical state**: Sticky Clear Solid; **^{1}H NMR (600 MHz, CDCl$_3$)** δ 4.23 – 4.16 (m, 2H), 1.56 – 1.48 (m, 2H), 1.42 – 1.39 (m, 2H), 1.33 – 1.18 (m, 4H); **^{13}C NMR (151 MHz, CDCl$_3$)** δ 172.9, 62.3, 59.0, 16.3, 14.1; **^{31}P NMR (243 MHz, CDCl$_3$)** δ -0.94; **HRMS (APCI)** m/z: [M+H]$^+$ calc. for $C_6H_{12}O_6P$: 211.0366, observed: 211.0361.

(3*s*,5*s*,7*s*)-Adamantan-1-yl phosphate (13b)

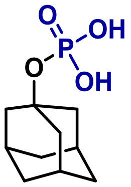

Following general procedure V, compound **13b** was obtained from debenzylation of **13a** (418 mg, 1.01 mmol). The crude product was purified by flash chromatography (0 – 50% MeOH in DCM) to afford the title compound (189 mg, 0.81 mmol, **80%**). **Physical state**: White Solid; **^{1}H NMR (400 MHz, DMSO-*d*$_6$)** δ 2.50 (s, 3H), 2.37 (s, 6H), 1.99 (s, 6H); **^{13}C NMR (125 MHz, DMSO-*d*$_6$)** δ 43.6, 36.1, 30.3; **^{31}P NMR (162 MHz, DMSO-*d*$_6$)** δ -4.92; **HRMS (APCI)** m/z: [M+H]$^+$ calc. for $C_{10}H_{17}O_4PNa$: 255.0757, observed: 255.0756.

[2-(4-Methylcyclohex-3-en-1-yl)propan-2-yl] phosphate (14b)

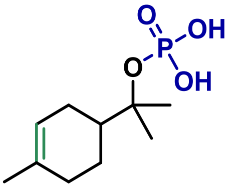

Following general procedure V, compound **14b** was obtained from debenzylation of **14a** (286 mg, 0.690 mmol). The crude product was purified by reverse phase flash chromatography (0 – 80% MeOH in H_2O) to afford the title compound (126 mg, 0.538 mmol, **78%**). **Physical state**:

White Solid; **^{1}H NMR (600 MHz, MeOD)** δ 5.39 (s, 1H), 2.12 (d, *J* = 16.7 Hz, 1H), 2.08 – 1.92 (m, 3H), 1.90 – 1.78 (m, 1H), 1.80 – 1.73 (m, 1H), 1.64 (s, 3H), 1.44 (d, *J* = 13.1 Hz, 6H), 1.36 – 1.25 (m, 1H); **^{13}C NMR (151 MHz, MeOD)** δ 133.3, 120.4, 82.0 (d, J_{CP} = 8.0 Hz), 44.9 (d, J_{CP} = 6.8 Hz), 30.6, 26.5, 24.3 (d, J_{CP} = 2.1 Hz), 23.7, 23.4 (d, J_{CP} = 2.6 Hz), 22.1; **^{31}P NMR (243 MHz, MeOD)** δ -3.37; **HRMS (ESI-TOF)** m/z: $[M-H]^+$ calc. for $C_{10}H_{18}O_4P$: 233.0948, observed: 233.0947.

{4-[(1*E*,6*E*)-7-(4-Hydroxy-3-methoxyphenyl)-3,5-dioxohepta-1,6-dien-1-yl]-2-methoxyphenyl} phosphate (15b)

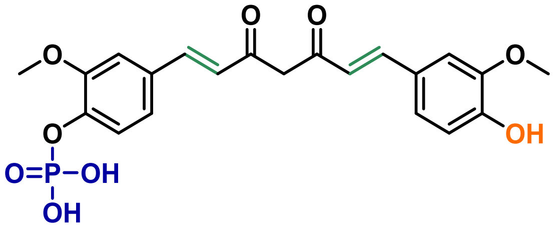

Following general procedure V, compound **15b** was obtained from debenzylation of **15a** (200 mg, 0.318 mmol). The crude product was purified by reverse phase flash chromatography (0 – 80% MeOH in H_2O) to afford the title compound (93 mg, 0.207 mmol, **65%**). **Physical state**: Yellow Solid; **^{1}H NMR (600 MHz, MeOD)** δ 7.61 (d, *J* = 6.0 Hz, 1H), 7.58 (d, *J* = 6.1 Hz, 1H), 7.35 (s, 1H), 7.31 – 7.29 (m, 1H), 7.23 (d, *J* = 1.9 Hz, 1H), 7.18 (d, *J* = 6.7 Hz, 1H), 7.12 (dd, *J* = 8.2, 1.9 Hz, 1H), 6.82 (d, *J* = 8.2 Hz, 1H), 6.74 (d, *J* = 15.8 Hz, 1H), 6.65 (d, *J* = 15.8 Hz, 1H), 3.91 (d, *J* = 6.1 Hz, 6H); **^{13}C NMR (151 MHz, MeOD)** δ 186.1, 183.2, 152.8, 150.6, 149.4, 142.7, 140.8, 133.5, 128.5, 124.6, 124.3, 122.5, 122.3 (d, J_{CP} = 3.6 Hz), 116.6, 112.9, 111.7, 56.5 (d, J_{CP} = 15.5 Hz); **^{31}P NMR (243 MHz, MeOD**) δ -4.91; **HRMS (ESI-TOF)** m/z: [M-H]+ calc. for $C_{21}H_{20}O_9P$: 447.0850, observed: 447.0846. ^{1}H, ^{13}C and ^{31}P NMR spectra corresponds to the literature.[7]

(*E*)-4-(3,5-Dimethoxystyryl)phenyl phosphate (17b)

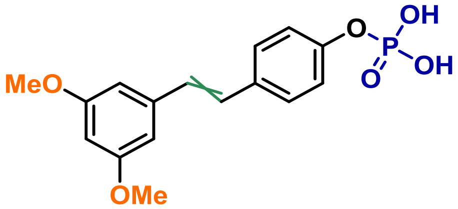

Following general procedure V, compound **17b** was obtained from debenzylation of **17a** (200 mg, 0.387 mmol). The crude product was purified by reverse phase flash chromatography (0 – 80% MeOH in H_2O) to afford the title compound (90 mg, 0.267 mmol, **69%**). **Physical state**: White Solid; **^{1}H NMR (600 MHz, $CDCl_3$)** δ ^{1}H NMR (600 MHz, $CDCl_3$) δ 7.21 (s, 2H), 7.05 (s, 2H), 6.79 (d, *J* = 16.2 Hz, 1H), 6.69 (d, *J* = 16.2 Hz, 1H), 6.46 (s, 2H), 6.27 (s, 1H), 3.68 (s, 6H); **^{13}C NMR (151 MHz, $CDCl_3$)** δ 160.9, 150.0, 139.2, 134.2, 128.7, 128.0, 127.9, 120.7, 104.6, 100.0, 55.4; **^{31}P NMR (243 MHz, $CDCl_3$)** δ -4.85; ^{1}H NMR spectra corresponds to the literature.[3]

(*S*)-{[4-(Prop-1-en-2-yl)cyclohex-1-en-1-yl]methyl}phosphonic acid (18b)

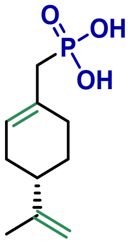

Following general procedure V, compound **18b** was obtained from phosphorylation of **18a** (500 mg, 1.26 mmol). The crude product was purified by reverse phase flash chromatography (0 – 80% MeOH in H_2O) to afford the title compound (229 mg, 1.06 mmol, **84%**). **Physical state**: White Solid; **^{1}H NMR (400 MHz, DMSO)** δ 5.43 (s, 1H), 4.71 – 4.66 (m, 2H), 2.35 – 2.21 (m, 2H), 2.20 – 2.13 (m, 2H), 2.10 – 1.99 (m, 2H), 1.92 – 1.82 (m, 1H), 1.75 – 1.67 (m, 4H), 1.48 – 1.29 (m, 1H); **^{31}P NMR (162 MHz, DMSO)** δ 20.38; **HRMS (APCI)** m/z: $[M-H]^+$ calc. for $C_{10}H_{16}O_3P$: 215.0843, observed: 215.0840. ^{1}H and ^{31}P NMR spectra corresponds to the literature.[4]

[(2-Oxo-2*H*-chromen-6-yl)methyl]phosphonic acid (19b)

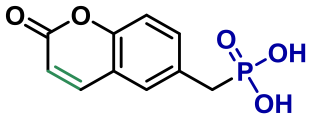

Following general procedure V, compound **19b** was obtained from phosphorylation of **19a** (280 mg, 0.660 mmol). The crude product was purified by reverse phase flash chromatography (0 – 80% MeOH in H_2O) to afford the title compound (128 mg, 0.535 mmol, **81%**). **Physical state**: White Solid; **^{1}H NMR (600 MHz, MeOD)** δ 7.95 (d, *J* = 9.5 Hz, 1H), 7.59 (s, 2H), 7.26 (d, *J* = 9.0 Hz, 1H), 6.41 (d, *J* = 9.5 Hz, 1H), 3.07 (s, 1H), 3.03 (s, 1H); **^{13}C NMR (151 MHz, MeOD)** δ 161.8 (s, 1C), 152.2 (d, J_{CP} = 2.8 Hz), 144.6 (1C), 133.6 (d, J_{CP} = 5.5 Hz), 133.4 (d, J_{CP} = 8.3 Hz), 128.7 (d, J_{CP} = 6.3 Hz), 118.6 (d, J_{CP} = 2.2 Hz), 115.6 (d, J_{CP} = 2.3 Hz), 115.3 (1C), 35.5 (d, J_{CP} = 129.5 Hz) ; **^{31}P NMR (243 MHz, MeOD)** δ 17.96; **HRMS (APCI)** m/z: $[M+H]^+$ calc. for $C_{10}H_{10}O_5P$: 241.0260, observed: 241.0258.

4-(Benzyloxy)phenyl) phosphate (20b)

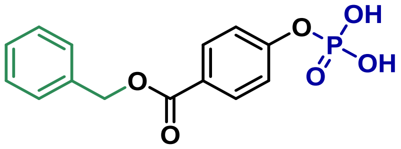

Following general procedure V, compound **20b** was obtained from debenzylation of **20a** (476 mg, 0.975 mmol). The crude product was purified by reverse flash chromatography (0 – 90% MeOH in H_2O) to afford the title compound (210 mg, 0.683 mmol, **70%**). **Physical state**: White Solid; **^{1}H NMR (400 MHz, acetone-*d*$_6$)** δ 11.46 (s, 2H), 8.03 (d, *J* = 8.2 Hz, 2H), 7.48 (d, *J* = 7.2

Hz, 2H), 7.42 – 7.28 (m, 5H), 5.35 (s, 2H); **^{13}C NMR (101 MHz, acetone-*d$_6$*)** δ 165.9, 155.9 (d, J_{CP} = 6.1 Hz), 137.3, 132.2, 129.4, 128.9, 128.9, 127.4, 121.1 (d, J_{CP} = 5.0 Hz), 67.1; **^{31}P NMR (162 MHz, acetone-*d$_6$*)** δ -6.5; **HRMS (ESI-TOF)** m/z: $[M-H]^+$ calc. for $C_{14}H_{12}O_6P$: 307.0377, observed: 307.0377.

4-Hydroxy phenyl phosphate (20c)

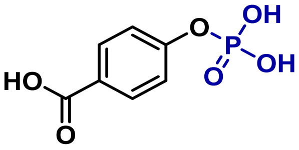

Following a modified general procedure V, compound **20c** was obtained from debenzylation of **20a** (225 mg, 0.461 mmol) using 4 equivalents of triethyl silane and stirring at rt for 2 hr. The crude product was purified by reverse phase flash chromatography (0 – 90% MeOH in H_2O) to afford the title compound (60 mg, 0.275 mmol, **60%**). **Physical state**: White Solid; **^{1}H NMR (400 MHz, DMSO-*d$_6$*)** δ 7.99 (d, J = 8.6 Hz, 2H), 7.32 (d, J = 8.5 Hz, 2H); **^{13}C NMR (125 MHz, DMSO-*d$_6$*)** δ166.8, 155.5 (d, J_{CP} = 6.0 Hz), 131.1, 126.1, 119.9 (d, J_{CP} = 5.1 Hz); **^{31}P NMR (162 MHz, DMSO-*d$_6$*)** δ -6.49; **HRMS (ESI-TOF)** $[M-H]^+$ calc. for $C_7H_6O_6P$: 216.9908, observed: 216.9908.

4-Amino phenyl phosphate (21b)

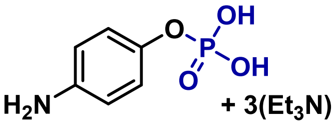

Following a modified general procedure V, compound **21b** was obtained from debenzylation of **21a** (326 mg, 0.648 mmol) using 4 equivalents of triethyl silane and stirring for 3 hr. The crude product was purified by reverse phase flash chromatography (0 – 100% MeOH in H_2O) to afford the title compound (37 mg, 0.13 mmol, **crude**). Compound **21b** can also be obtained in the same manner from debenzylation of **23a** (226 mg, 0.492 mmol), using 8 equivalents of triethyl silane and stirring for 3 hr to afford the title compound (85 mg, 0.170 mmol, **crude**). **Physical state**: Light Brown Solid; **^{1}H NMR (400 MHz, DMSO-*d$_6$*)** δ 6.82 – 6.75 (m, 2H), 6.47 – 6.38 (m, 2H), 2.94 (q, J = 7.2 Hz, 18H), 1.15 (t, J = 7.2 Hz, 27H); **^{13}C NMR (125 MHz, DMSO-*d$_6$*)** δ 144.51 (d, J_{CP} = 6.8 Hz), 143.4, 120.5 (d, J_{CP} = 4.5 Hz), 114.1, 45.2, 8.7; **^{31}P NMR (162 MHz, DMSO-*d$_6$*)** δ -4.61; **HRMS (ESI-TOF)** m/z: $[M+H]^+$ calc. for $C_6H_9O_4NP$: 190.0264, observed: 190.0267.

Note: Attempts to isolate compound **21b** as the HCl salt revealed instability. Consequently, isolation was achieved by adding excess triethylamine post reaction, allowing for recovery as the triethylamine salt. This approach provided clean spectra suitable for reporting, though an accurate yield of the debenzylation could not be determined due to the potential presence of excess

triethylamine. Nonetheless, these findings confirmed the method's compatibility with Cbz- and benzyl-protected amines.

4-(Benzyloxy)phenyl) phosphate (22b)

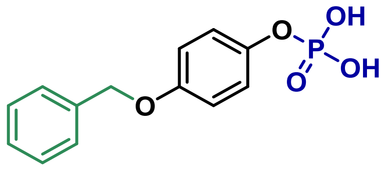

Following general procedure V, compound **22b** was obtained from debenzylation of **22a** (400 mg, 0.869 mmol). The crude product was purified by reverse flash chromatography (0 – 80% MeOH in H_2O) to afford the title compound (186 mg, 0.660 mmol, **76%**). **Physical state**: White Solid; **^{1}H NMR (400 MHz, DMSO)** δ 11.75 (s, 2H), 7.48 – 7.27 (m, 5H), 7.07 (d, *J* = 8.5 Hz, 2H), 6.97 (d, *J* = 9.0 Hz, 2H), 5.06 (s, 2H); **^{13}C NMR (125 MHz, DMSO)** δ 154.6, 145.2 (d, J_{CP} = 6.5 Hz), 137.1, 128.4, 127.8, 127.6, 121.1 (d, J_{CP} = 4.5 Hz), 115.4, 69.5; **^{31}P NMR (162 MHz, $CDCl_3$)** δ -6.3**; HRMS (APCI)** m/z: $[M+H]^+$ calc. for $C_{13}H_{14}O_5P$: 281.0573, observed: 281.0571.

4-Hydroxy phenyl phosphate (22c)

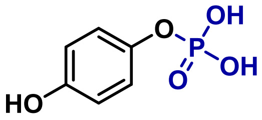

Following a modified general procedure V, compound **22c** was obtained from debenzylation of **22a** (1000 mg, 2.17 mmol) using 4 equivalents of triethyl silane and stirring for 16 hr. Workup procedure was altered by addition of 2M HCl in Et_2O (0.543 mL, 1.086 mmol), after which the organic phase was stirred with concentrated brine. The crude product was purified by reverse phase flash chromatography (0 – 90% MeOH in H_2O) to afford the title compound (358 mg, 1.89 mmol, **87%**). **Physical state**: White Solid; **^{1}H NMR (400 MHz, DMSO)** δ 9.76 (s, 3H), 6.94 (d, *J* = 8.4 Hz, 2H), 6.69 (d, *J* = 8.7 Hz, 2H); **^{13}C NMR (101 MHz, DMSO)** δ 153.7, 143.9 (d, J_{CP} = 6.6 Hz), 121.1 (d, J_{CP} = 4.2 Hz), 115.6; **^{31}P NMR (162 MHz, DMSO)** δ -5.54; **HRMS (ESI-TOF)** $[M-H]^+$ calc. for $C_6H_6O_5P$: 188.9958, observed: 188.9961.

4-(Benzylamino)phenyl phosphate hydrochloride (23b)

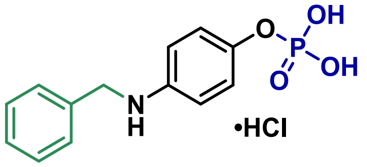

Following general procedure V, compound **23b** was obtained from debenzylation of **23a** (100 mg, 0.218 mmol). The crude product was purified by reverse phase flash chromatography (0 –

80% MeOH in H_2O) to afford the title compound (39 mg, 0.120 mmol, **57%**). **Physical state**: White Solid; **^{1}H NMR (400 MHz, DMSO-d_6)** δ 7.38 – 7.27 (m, 4H), 7.25 – 7.17 (m, 1H), 6.89 – 6.80 (m, 2H), 6.54 – 6.46 (m, 2H), 4.22 (s, 2H); **^{13}C NMR (125 MHz, DMSO-d_6)** δ 145.32, 141.9 (d, J_{CP} = 7.0 Hz), 140.3, 128.3, 127.2, 126.6, 120.7 (d, J_{CP} = 4.4 Hz), 112.6, 46.8; **^{31}P NMR (162 MHz, DMSO-d_6)** δ -5.46; **HRMS (APCI)** m/z: $[M+H]^+$ calc. for $C_{13}H_{15}NO_4P$: 280.0733, observed: 280.0727.

(4-Allyloxyphenyl) dibenzyl phosphate (24b)

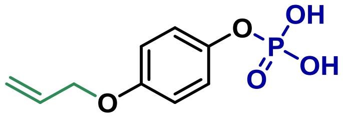

Following general procedure V, compound **24b** was obtained from debenzylation of **24a** (400 mg, 0.975 mmol). The crude product was purified by reverse flash chromatography (0 – 80% MeOH in H_2O) to afford the title compound (130 mg, 0.566 mmol, **58%**). **Physical state**: White Solid; **^{1}H NMR (400 MHz, DMSO)** δ 7.09 – 7.03 (m, 2H), 6.94 – 6.85 (m, 2H), 6.02 (ddt, J = 17.3, 10.5, 5.2 Hz, 1H), 5.38 (dq, J = 17.3, 1.7 Hz, 1H), 5.24 (dq, J = 10.5, 1.4 Hz, 1H), 4.52 (dt, J = 5.2, 1.5 Hz, 2H); **^{13}C NMR (125 MHz, DMSO)** δ 154.5, 145.3 (d, J_{CP} = 6.6 Hz), 133.9, 121.1 (d, J_{CP} = 4.5 Hz), 117.5, 115.3, 68.6; **^{31}P NMR (162 MHz, DMSO)** δ -5.72; **HRMS (APCI)** m/z: $[M+H]^+$ calc. for $C_9H_{12}O_5P$: 231.0417, observed: 231.0415.

Dibenzyl [(13*S*)-17-hydroxy-13-methyl-7,8,9,11,12,13,14,15,16,17-decahydro-6*H*-cyclopenta[*a*]phenanthren-3-yl] phosphate (26b)

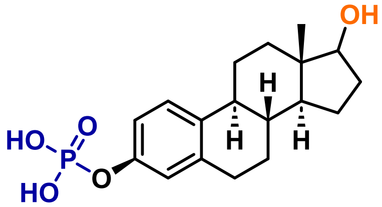

Following general procedure V, compound **26b** was obtained from debenzylation of **26a** (200 mg, 0.375 mmol). The crude product was purified by reverse phase flash chromatography (0 – 80% MeOH in H_2O) to afford the title compound (103 mg, 0.293 mmol, **78%**). **Physical state**: White Solid; **^{1}H NMR (600 MHz, DMSO-d_6)** δ 7.21 (d, J = 8.5 Hz, 1H), 6.88 (dd, J = 8.5, 2.6 Hz, 1H), 6.84 (d, J = 2.6 Hz, 1H), 3.52 (t, J = 8.5 Hz, 1H), 2.85 – 2.63 (m, 2H), 2.27 (dq, J = 11.5, 3.4 Hz, 1H), 2.12 (td, J = 11.2, 4.1 Hz, 1H), 1.93 – 1.74 (m, 3H), 1.59 (tdt, J = 9.7, 7.4, 3.4 Hz, 1H), 1.45 – 1.08 (m, 7H), 0.67 (s, 3H); **^{13}C NMR (151 MHz, DMSO-d_6)** δ 149.3 (d, J_{CP} = 6.3 Hz), 137.5, 135.6, 126.2, 119.8 (d, J_{CP} = 4.2 Hz), 117.3 (d, J_{CP} = 4.7 Hz), 80.0, 49.5, 43.6, 42.8, 38.4, 36.6, 29.9, 29.1, 26.7, 26.0, 22.8, 11.2; **^{31}P NMR (243 MHz, DMSO-d_6)** δ -5.89; **HRMS (ESI-TOF)** m/z: **HRMS (ESI-TOF)** m/z: $[M-H]^+$ calc. for $C_{18}H_{24}O_5P$: 351.1369, observed: 351.1368. ^{1}H, ^{13}C and

^{31}P NMR spectra corresponds to the literature.[14]

Monobenzyl [(13*S*)-17-hydroxy-13-methyl-7,8,9,11,12,13,14,15,16,17-decahydro-6*H*-cyclopenta[*a*]phenanthren-3-yl] phosphate (26c)

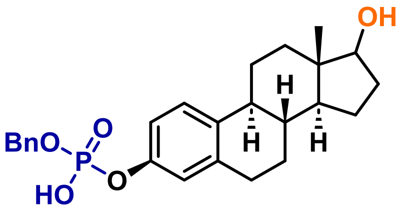

Following general procedure VI, compound **26c** was obtained from debenzylation of **26a** (100 mg, 0.187 mmol). The crude product was purified by reverse phase flash chromatography (0 – 80% MeOH in H_2O) to afford the title compound (61 mg, 0.135 mmol, **74%**). **Physical state**: White Solid; **^{1}H NMR (600 MHz, DMSO-*d*$_6$)** δ 7.40 – 7.28 (m, 5H), 7.22 (d, *J* = 8.3 Hz, 1H), 6.90 (d, *J* = 8.4 Hz, 1H), 6.82 (s, 1H), 5.10 – 4.91 (m, 2H), 3.52 (t, *J* = 8.5 Hz, 1H), 2.73 (d, *J* = 5.5 Hz, 2H), 2.27 (dd, *J* = 13.8, 3.6 Hz, 1H), 2.13 (td, *J* = 11.2, 4.0 Hz, 1H), 1.86 (ddt, *J* = 23.1, 12.8, 3.7 Hz, 2H), 1.79 (ddd, *J* = 11.6, 5.3, 2.7 Hz, 1H), 1.58 (qd, *J* = 10.5, 3.4 Hz, 1H), 1.42 – 1.07 (m, 7H), 0.67 (s, 3H; **^{13}C NMR (151 MHz, DMSO-*d*$_6$)** δ 148.8 (d, J_{CP} = 6.19 Hz), 137.8, 136.7 (d, J_{CP} = 6.5 Hz), 136.1, 128.4, 128.0, 127.6, 126.3, 119.8 (d, J_{CP} = 3.7 Hz), 117.2 (d, J_{CP} = 3.4 Hz), 80.0, 67.9 (d, J_{CP} = 4.7 Hz), 49.5, 43.6, 42.8, 38.3, 36.5, 29.9, 29.0, 26.7, 25.9, 22.8, 11.2; **^{31}P NMR (243 MHz, DMSO-*d*$_6$)** δ -6.36; **HRMS (ESI-TOF)** m/z: $[M-H]^+$ calc. for $C_{25}H_{30}O_5P$: 441.1836, observed: 441.1831.

(8*R*,9*S*,13*S*,14*S*)-17-(benzylamino)-13-methyl-7,8,9,11,12,13,14,15,16,17-decahydro-6*H*-cyclopenta[a]phenanthren-3-yl phosphate hydrochloride (27b)

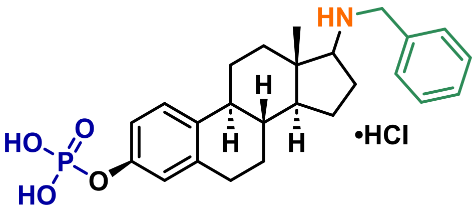

Following general procedure V, compound **27b** was obtained from debenzylation of **27a** (300 mg, 0.480 mmol). The crude product was purified by reverse phase flash chromatography (0 – 80% MeOH in H_2O) to afford the title compound (170 mg, 0.592 mmol, **74%**). **Physical state**: White Solid; **^{1}H NMR (600 MHz, DMSO)** δ 10.61 (s, 1H), 9.45 (d, *J* = 53.3 Hz, 2H), 9.03 (s, 1H), 7.71 – 7.67 (m, 2H), 7.45 – 7.38 (m, 3H), 7.15 (d, *J* = 8.6 Hz, 1H), 6.87 (dd, *J* = 8.5, 2.5 Hz, 1H), 6.83 (s, 1H), 4.22 (d, *J* = 13.1 Hz, 1H), 4.04 (d, *J* = 13.2 Hz, 1H), 2.86 (h, *J* = 7.1 Hz, 1H), 2.79 (s, 1H), 2.75 – 2.70 (m, 1H), 2.28 (d, *J* = 12.4 Hz, 1H), 2.25 – 2.18 (m, 1H), 2.12 – 2.03 (m, 1H), 1.94 (ddd, *J* = 18.3, 11.4, 7.2 Hz, 1H), 1.84 – 1.71 (m, 2H), 1.70 – 1.61 (m, 1H), 1.35 – 1.22 (m,

4H), 1.16 – 1.02 (m, 2H), 0.84 (s, 3H); **^{13}C NMR (151 MHz, DMSO)** δ 149.9, 137.1, 134.6, 131.8, 130.3, 128.9, 128.6, 126.0, 119.8 (d, J_{CP} = 4.5 Hz), 117.4 (d, J_{CP} = 4.7 Hz), 65.3, 50.9, 49.4, 43.0, 42.2, 41.2, 37.7, 35.8, 28.9, 26.7, 25.4, 24.5, 22.8, 11.8, 10.9; **^{31}P NMR (243 MHz, DMSO-*d*$_6$)** δ -5.61; **HRMS (ESI-TOF)** m/z: [M-H]$^+$ calc. for $C_{25}H_{32}O_4NClP$: 476.1763, observed: 476.1750.

(*S*)-2-Amino-3-[4-(phosphonooxy)phenyl]propanoic acid hydrochloroide (28b)

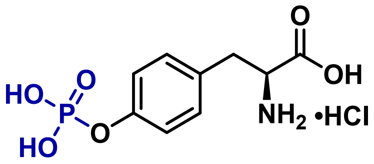

Following a modified version of general procedure V, compound **28b** was obtained from debenzylation of **28a** (222 mg, 0.333 mmol) using 5 equivalents of triethylsilane and 20 mol% $Pd(OAc)_2$. The crude product was purified by reverse phase flash chromatography (0 – 80% MeOH in H_2O) to afford the title compound (77 mg, 0.293 mmol, **88%**). **Physical state**: White Solid; **^{1}H NMR (600 MHz, DMSO-*d*$_6$)** δ 7.05 (d, *J* = 8.8 Hz, 2H), 6.67 (d, *J* = 8.8 Hz, 2H), 3.35 (dd, *J* = 8.5, 4.2 Hz, 1H), 3.03 (dd, *J* = 14.4, 4.2 Hz, 1H), 2.74 (dd, *J* = 14.4, 8.5 Hz, 1H), -NH_2 and -OH signals not observed in spectra; **^{13}C NMR (151 MHz, DMSO-*d*$_6$)** δ 170.2, 156.2, 130.6 (2C), 127.4, 115.5 (2C), 56.1, 36.1; **^{31}P NMR (243 MHz, DMSO-*d*$_6$)** δ -0.93. ^{1}H, ^{13}C and ^{31}P NMR spectra corresponds to the literature.[15]

{2-[(10*R*,11*S*,13*S*,17*R*)-11,17-dihydroxy-10,13-dimethyl-3-oxo-6,7,8,9,10,11,12,13,14,15,16,17-dodecahydro-3*H*-cyclopenta[a]phenanthren-17-yl]-2-oxoethyl} phosphate (29b)

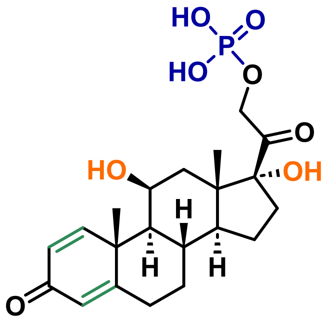

Following general procedure V, compound **29b** was obtained from debenzylation of **29a** (100 mg, 0.160 mmol). The crude product was purified by reverse phase flash chromatography (0 – 80% MeOH in H_2O) to afford the title compound (49 mg, 0.110 mmol, **69%**). **Physical state**: White Solid; **^{1}H NMR (600 MHz, MeOD)** δ 7.54 – 7.43 (m, 1H), 6.31 – 6.23 (m, 1H), 6.00 (s, 1H), 5.01 – 4.94 (m, 1H), 4.65 – 4.58 (m, 1H), 4.43 – 4.36 (m, 1H), 2.79 – 2.70 (m, 1H), 2.65 (td, *J* = 12.7, 4.7 Hz, 1H), 2.37 (dd, *J* = 13.6, 3.0 Hz, 1H), 2.22 – 2.11 (m, 2H), 2.00 (dd, *J* = 13.9, 3.8 Hz, 1H), 1.84 – 1.69 (m, 3H), 1.53 – 1.36 (m, 5H), 1.19 – 1.08 (m, 1H), 1.01 (dd, *J* = 11.1, 3.6 Hz,

1H), 0.93 (s, 3H); **^{13}C NMR (151 MHz, MeOD)** δ 212.1 (d, J_{CP} = 7.7 Hz), 187.6, 173.4, 158.8, 126.4, 121.1, 89.1, 69.5, 68.6 (d, J_{CP} = 3.7 Hz), 56.00, 51.5, 47.1 (d, J_{CP} = 2.1 Hz), 44.7, 38.92, 34.2, 33.2, 31.9, 31.4, 23.6, 20.2, 16.2; **^{31}P NMR (243 MHz, MeOD)** δ 5.23; **HRMS (ESI-TOF)** m/z: [M-H]$^+$ calc. for $C_{21}H_{28}O_8P$: 439.1527, observed: 439.1525.

{4-[2-(Dimethylamino)-1-(1-hydroxycyclohexyl)ethyl]phenyl} phosphate (30b)

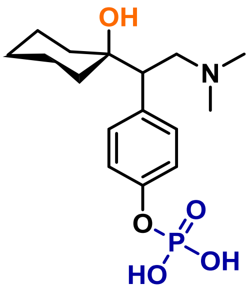

Following general procedure V, compound **30b** was obtained from debenzylation of **30a** (200 mg, 0.380 mmol). The crude product was purified by reverse phase flash chromatography (0 – 80% MeOH in H_2O) to afford the title compound (78 mg, 0.230 mmol, **59%**). **Physical state**: White Solid; **^{1}H NMR (600 MHz, MeOD)** δ 7.30 (d, *J* = 8.6 Hz, 2H), 7.25 (d, *J* = 8.6 Hz, 2H), 3.71 (dd, *J* = 13.2, 4.4 Hz, 1H), 3.63 (dd, *J* = 13.2, 10.3 Hz, 1H), 3.08 (dd, *J* = 10.2, 4.4 Hz, 1H), 2.78 (s, 6H), 1.73 – 1.60 (m, 2H), 1.57 – 1.43 (m, 4H), 1.39 – 1.23 (m, 3H), 1.11 – 1.00 (m, 1H), -OH signal not observed in spectra; **^{13}C NMR (151 MHz, MeOD)** δ 154.5 (d, J_{CP} = 6.6 Hz), 132.8 (2C), 131.5, 121.9 (2C) (d, J_{CP} = 5.0 Hz), 74.1, 60.0, 52.8, 44.2 (2C), 37.4, 34.3, 26.6, 22.7, 22.4; **^{31}P NMR (243 MHz, MeOD)** δ -3.80; **HRMS (ESI-TOF)** m/z: [M-H]$^+$ calc. for $C_{16}H_{25}O_5NP$: 342.1476, observed: 342.1470.

[(3*S*,8*S*,9*S*,10*R*,13*R*,14*S*,17*R*)-10,13-dimethyl-17-((*R*)-6-methylheptan-2-yl)-2,3,4,7,8,9,10,11,12,13,14,15,16,17-tetradecahydro-1*H*-cyclopenta[a]phenanthren-3-yl] phosphate (31b)

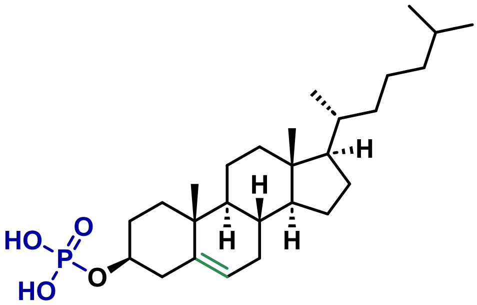

Following general procedure V, compound **31b** was obtained from debenzylation of **31a** (250 mg, 0.380 mmol). The crude product was purified by flash chromatography (0 – 60% DCM in MeOH) to afford the title compound (128 mg, 0.270 mmol, **71%**). **Physical state**: White Solid; **^{1}H NMR (600 MHz, $CDCl_3$:MeOD (2:1))** δ 5.39 – 5.28 (m, 1H), 4.05 (dddd, J = 16.4, 11.4, 7.3, 5.0

Hz, 1H), 2.46 – 2.29 (m, 2H), 2.07 – 1.90 (m, 3H), 1.81 (ddt, J = 22.7, 15.6, 4.9 Hz, 2H), 1.70 – 1.59 (m, 1H), 1.59 – 1.38 (m, 6H), 1.37 – 1.20 (m, 4H), 1.17 – 1.00 (m, 7H) (m, 7H), 1.02 – 0.91 (m, 5H), 0.89 (d, J = 6.5 Hz, 3H), 0.83 (dd, J = 6.6, 2.6 Hz, 6H), 0.65 (s, 3H); **^{13}C NMR (151 MHz, $CDCl_3$:MeOD (2:1))** δ 140.2, 123.0, 57.1, 56.6, 50.5, 42.7, 40.3 (d, J_{CP} = 4.6 Hz), 40.1, 39.9, 37.4, 36.8, 36.6, 36.2, 32.3 (d, J_{CP} = 2.7 Hz), 29.9 (d, J_{CP} = 4.2 Hz), 28.6, 28.3, 24.6, 24.2, 23.0, 22.7, 21.4, 19.5, 19.0, 12.1; **^{31}P NMR (243 MHz, $CDCl_3$:MeOD (2:1))** δ -0.61. ^{1}H NMR spectra corresponds to the literature.[2]

(*E/Z*)-[4-(1-{4-[2-(dimethylamino)ethoxy]phenyl}-2-phenylbut-1-en-1-yl)phenyl] phosphate (32b)

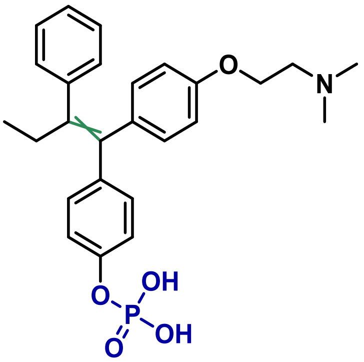

Following general procedure V, compound **32b** was obtained from debenzylation of **32a** (161 mg, 0.249 mmol). The crude product was purified by reverse phase flash chromatography (0 – 80% MeOH in H_2O) to afford the title compound (71 mg, 0.140 mmol; **57%**). **Physical state**: White Solid; **^{1}H NMR (400 MHz, DMSO-*d_6*)** δ 7.18 (td, *J* = 7.4, 5.2 Hz, 2H), 7.13 – 7.04 (m, 5H), 6.97 (d, *J* = 8.5 Hz, 1H), 6.92 (d, *J* = 8.3 Hz, 1H), 6.72 (dd, *J* = 18.7, 8.5 Hz, 2H), 6.66 – 6.54 (m, 2H), 4.24 (s, 1H), 4.08 (s, 1H), 3.30 – 3.14 (m, 2H), 2.66 (s, 3H), 2.59 (s, 3H), 2.44 – 2.37 (m, 2H), 0.85 (t, *J* = 7.3 Hz, 3H); **^{13}C NMR (125 MHz, DMSO-*d_6*)** δ 156.4, 155.5, 152.5 (d, J_{CP} = 7.1 Hz), 151.6 (d, J_{CP} = 6.9 Hz), 141.99, 141.97, 140.3, 137.5, 136.8, 136.4, 136.2, 135.8, 131.4, 130.8, 130.2, 129.5, 129.4, 129.3, 128.0, 127.9, 126.2, 126.1, 119.69, 119.66, 118.8, 118.7, 114.3, 113.5, 62.8, 62.6, 55.4, 55.3, 43.0, 42.96, 40.1, 28.7, 28.6, 13.40, 13.38; **^{31}P NMR (162 MHz, DMSO-*d_6*)** δ -4.76, -5.01; **HRMS (ESI-TOF)** m/z: $[M+H]^+$ calc. for $C_{26}H_{31}NO_5P$: 468.1934, observed: 468.1935.

Ammonium benzyl [4-(1-{4-[2-(dimethylamino)ethoxy]phenyl}-2-phenyl-but-1-enyl)phenyl] phosphate (32c)

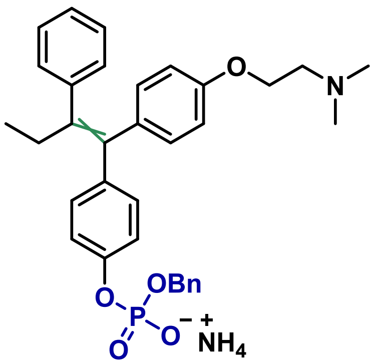

Following general procedure VI, compound **32c** was obtained by mono-debenzylation of **32a** (119 mg, 0.184 mmol). The crude product was purified by flash chromatography (0 – 100% DCM:MeOH:$NH_4OH_{(aq)}$ (80:20:3) in DCM) to afford the title compound as the ammonium salt. (51 mg, 0.089 mmol, **48%**). **Physical state**: White solid; **^{1}H NMR (400 MHz, MeOD)** δ 7.37 – 7.20 (m, 5H), 7.19 – 7.03 (m, 8H), 6.95 (d, *J* = 8.3 Hz, 1H), 6.85 – 6.70 (m, 3H), 6.58 (d, *J* = 8.4 Hz, 1H), 4.98 (d, *J* = 6.5 Hz, 1H), 4.84 (d, *J* = 6.3 Hz, 1H), 4.26 (t, *J* = 4.8 Hz, 1H), 4.11 – 4.04 (m, 1H), 3.46 (t, *J* = 4.9 Hz, 1H), 3.35 (t, *J* = 4.9 Hz, 1H), 2.86 (s, 3H), 2.78 (s, 3H), 2.52 – 2.40 (m, 2H), 0.90 (t, *J* = 7.4 Hz, 3H). **^{31}P NMR (162 MHz, MeOD)** δ -4.79, -5.04. **^{13}C NMR (151 MHz, MeOD)** δ 157.9, 157.1, 153.14, 153.09, 152.24, 152.20, 143.7, 143.6, 143.1, 143.0, 139.9, 139.6, 139.32, 139.26, 139.2, 139.1, 138.5, 138.1, 133.2, 132.7, 131.8, 131.4, 130.9, 130.8, 129.4, 129.3, 129.02, 128.98, 128.8, 128.7, 128.7, 128.6, 127.3, 127.2, 121.10, 121.07, 120.20, 120.17, 115.5, 114.6, 68.94, 68.90, 68.9, 68.8, 63.5, 63.2, 57.72, 57.66, 44.0, 43.9, 30.02, 30.00, 13.89, 13.87. **HRMS** (ESI-TOF) *m/z*: $[M+H]^+$ calc. for $C_{33}H_{37}NO_5P$: 558.2404, observed: 558.2423.

Ammonium benzyl {(S)-(6-methoxyquinolin-4-yl)[(1*R*,2*R*,4*R*,5*S*)-5-vinylquinuclidin-2-yl]methyl} phosphate (33b)

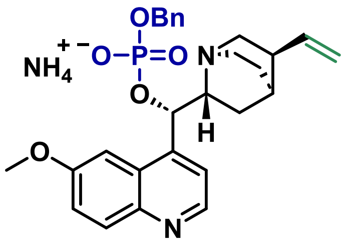

Following general procedure VI, compound **33b** was obtained from debenzylation of **33a** (770 mg, 1.37 mmol). The crude product was purified by flash chromatography (0 – 100% DCM:MeOH:$NH_4OH_{(aq)}$ (80:20:3) in DCM) to afford the title compound as the ammonium salt. (141 mg, 0.301 mmol, **22%**). **Physical state**: White Solid; **^{1}H NMR (400 MHz, $CDCl_3$)** δ 13.74 – 13.19 (m, 1H), 8.65 (d, *J* = 4.5 Hz, 1H), 7.94 (d, *J* = 9.2 Hz, 1H), 7.57 (d, *J* = 4.4 Hz, 1H), 7.38 – 7.22 (m, 5H), 7.16 (s, 1H), 7.13 – 6.96 (m, 2H), 5.56 (ddd, *J* = 17.2, 10.5, 6.8 Hz, 1H), 5.07 – 4.97 (m, 4H), 4.43 (t, *J* = 10.5 Hz, 1H), 3.52 (dd, *J* = 13.3, 10.5 Hz, 1H), 3.43 – 3.00 (m, 3H), 2.89

– 2.72 (m, 4H), 2.65 (d, *J* = 7.4 Hz, 1H), 2.38 – 2.23 (m, 2H), 2.15 – 2.04 (m, 1H), 1.94 – 1.74 (m, 1H), 1.58 – 1.41 (m, 1H).; **^{13}C NMR (101 MHz, $CDCl_3$)** δ 158.6, 147.0, 144.2, 141.8, 138.2 (d, J_{CP} = 7.6 Hz), 137.6, 131.5, 128.4, 127.8, 127.6, 125.6, 122.2, 119.2, 117.1, 100.2, 72.2, 67.8, 59.6 (d, J_{CP} = 7.5 Hz), 55.6, 54.4, 43.1, 37.2, 27.0, 24.5, 18.7; **^{31}P NMR (162 MHz, $CDCl_3$)** δ -1.21; **HRMS (ESI-TOF)** m/z: $[M+H]^+$ calc. for $C_{27}H_{31}N_2O_5P$: 495.2050, observed: 495.2050.

(1*R*,2*R*,4*R*,5*S*)-2-((6-Methoxyquinolin-4-yl)methyl)-5-vinylquinuclidine (33c)

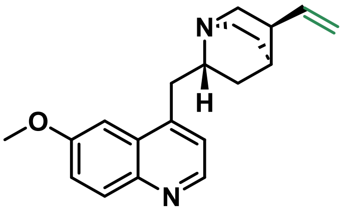

Following general procedure V, compound **33c** was obtained from deoxygenation of **33a** (770 mg, 1.37 mmol). The crude product was purified by flash chromatography (0 – 100% DCM:MeOH:$NH_4OH_{(aq)}$ (80:20:3) in DCM) to afford the title compound (224 mg, 0.730 mmol, **53%**). **Physical state**: Light Yellow Semi Solid; **^{1}H NMR (400 MHz, $CDCl_3$)** δ 8.65 (d, *J* = 4.5 Hz, 1H), 8.00 (d, *J* = 9.1 Hz, 1H), 7.35 (dd, *J* = 9.2, 2.8 Hz, 1H), 7.26 (d, *J* = 2.8 Hz, 1H), 7.22 (d, *J* = 4.4 Hz, 1H), 5.77 (ddd, *J* = 17.6, 10.3, 7.6 Hz, 1H), 5.02 – 4.90 (m, 2H), 3.93 (s, 3H), 3.38 (dd, *J* = 13.7, 5.5 Hz, 1H), 3.26 – 3.17 (m, 3H), 2.98 (dd, *J* = 13.9, 8.8 Hz, 1H), 2.83 – 2.63 (m, 2H), 2.28 (s, 1H), 1.82 – 1.72 (m, 2H), 1.70 – 1.53 (m, 2H), 1.16 (dd, *J* = 12.2, 7.1 Hz, 1H); **^{13}C NMR (101 MHz, $CDCl_3$)** δ 157.8, 147.6, 144.5, 143.8, 141.8, 131.8, 128.8, 121.9, 121.5, 114.5, 101.7, 56.3, 55.7, 55.6, 41.1, 39.6, 38.4, 28.9, 28.0, 27.9; **HRMS (ESI-TOF)** m/z: $[M+H]^+$ calc. for $C_{20}H_{25}ON_2$: 309.1961, observed: 309.1959. ^{1}H and ^{13}C NMR spectra corresponds to the literature.[16]

((6a*R*,10a*R*)-6,6,9-trimethyl-3-pentyl-6a,7,10,10a-tetrahydro-6*H*-benzo[c]chromen-1-yl) phosphate (S5)

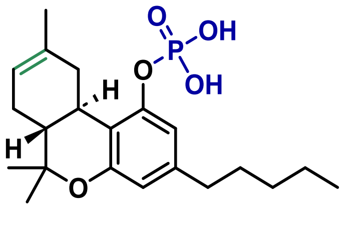

Following general procedure V, compound **S5** was obtained from debenzylation of **S3** (110 mg, 0.180 mmol). The crude product was purified by reverse phase flash chromatography (0 – 80% MeOH in H_2O) to afford the title compound (66 mg, 0.155 mmol, **86%**). **Physical state**: White Solid; **^{1}H NMR (600 MHz, MeOD)** δ 6.93 (s, 1H), 6.26 (s, 1H), 5.39 (s, 1H), 3.50 – 3.43 (m, 1H), 2.84 (td, *J* = 11.0, 4.3 Hz, 1H), 2.49 – 2.44 (m, 2H), 2.16 – 2.08 (m, 1H), 1.85 – 1.73 (m, 2H),

1.71 (s, 3H), 1.65 – 1.69 (m, 1H). 1.59 (p, J = 7.5 Hz, 2H), 1.33 (s, 3H), 1.39 – 1.26 (m, 6H), 1.07 (s, 3H). 0.91 (t, J = 7.1 Hz, 3H); **^{13}C NMR (151 MHz, MeOD)** δ 155.4, 154.2, 143.0, 136.5, 120.0, 116.3, 113.6, 112.5, 77.5, 47.0, 37.4, 36.8, 33.3, 32.9, 32.1, 29.0, 28.0, 23.7, 23.6, 18.7, 14.4; **^{31}P NMR (243 MHz, MeOD)** δ -3.82; **HRMS (ESI-TOF)** m/z: [M-H]$^+$ calc. for $C_{21}H_{30}O_5P$: 393.1836, observed: 393.1842.

(*R*)-2,8-Dimethyl-2-((4*R*,8*R*)-4,8,12-trimethyltridecyl)chroman-6-yl phosphate (S6)

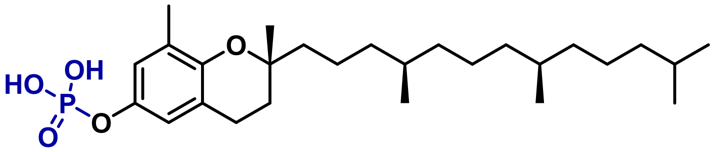

Following general procedure V, compound **S6** was obtained from debenzylation of **S4** (232 mg, 0.350 mmol). The crude product was purified by reverse phase flash chromatography (0 – 60% MeOH in DCM) to afford the title compound (127 mg, 0.263 mmol, **75%**). **Physical state**: Colorless oil; **^{1}H NMR (400 MHz, $CDCl_3$)** δ 6.77 (d, J = 2.9 Hz, 1H), 6.74 – 6.71 (m, 1H), 2.66 (q, J = 7.1 Hz, 2H), 2.09 (s, 3H), 1.71 (dq, J = 30.4, 6.9 Hz, 2H), 1.58 – 1.48 (m, 3H), 1.46 – 1.00 (m, 21H), 0.88 – 0.79 (m, 12H); **^{13}C NMR (125 MHz, $CDCl_3$)** δ 149.4, 142.6, 127.6, 121.3, 120.2 (d, J_{CP} = 4.1 Hz), 118.0 (d, J_{CP} = 3.8 Hz), 76.2, 40.4, 39.5, 37.6 (d, J_{CP} = 2.8 Hz), 37.4, 32.9, 32.9, 31.1, 28.1, 24.9, 24.6, 24.1, 22.9, 22.8, 22.5, 21.1, 19.9, 19.8, 16.2; **^{31}P NMR (162 MHz, $CDCl_3$)** δ -2.82; **HRMS (ESI-TOF)** m/z: [M+H]$^+$ calc. for $C_{27}H_{46}O_5P$: 481.3088, observed: 481.3090.

N-propylaniline (S7)

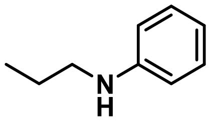

Following general procedure VI, compound **S7** was obtained from allylic reduction of allylaniline (400 mg, 3.00 mmol). The crude product was purified by flash chromatography (0 – 100% EtOAc in hexanes) to afford the title compound (373 mg, 2.76 mmol, **92%**). **Physical state**: Brown liquid; **^{1}H NMR (600 MHz, $CDCl_3$)** δ 7.40 – 7.30 (m, 1H), 6.89 – 6.83 (m, 1H), 6.77 – 6.72 (m, 2H), 3.71 (s, 1H), 3.24 – 3.18 (m, 3H), 1.82 – 1.72 (m, 2H), 1.18 – 1.12 (m, 3H); **^{13}C NMR (151 MHz, $CDCl_3$)** δ 180.0, 160.6 (2C), 148.4, 144.1 (2C), 77.16, 54.1, 43.1; **HRMS (APCI)** m/z: [M+H]$^+$ calc. for $C_9H_{14}N$: 136.1120, observed: 136.1121; ^{1}H spectra corresponds to the literature.[17]

Applications of Mono-debenzylated Cannabidiol Phosphate

Benzyl {(1'*R*,2'*R*)-5'-methyl-4-pentyl-2'-(prop-1-en-2-yl)-6-[(triisopropylsilyl)oxy]-1',2',3',4'-tetrahydro-[1,1'-biphenyl]-2-yl} phosphate (40)

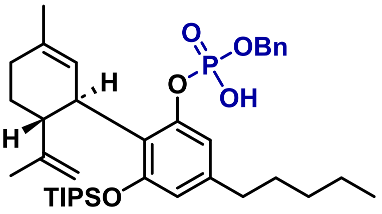

Following general procedure VI, compound **40** was obtained from debenzylation of **4c** (1.46 g, 2.00 mmol). The crude product was purified by flash chromatography (0 – 100% EtOAc in hexanes) to afford the title compound (1.05 g, 1.64 mmol, **82%**). **Physical state**: Clear oil; **^{1}H NMR (600 MHz, $CDCl_3$)** δ 7.30 – 7.19 (m, 5H), 6.77 (br s, 1H), 6.37 (s, 1H), 5.19 (s, 1H), 5.04 (d, *J* = 6.6 Hz, 2H), 4.51 (br s, 1H), 4.45 (s, 1H), 4.10 – 3.82 (m, 1H), 3.09 – 2.79 (m, 1H), 2.41 (t, *J* = 7.6 Hz, 2H), 2.17 – 2.09 (m, 1H), 1.95 – 1.89 (m, 1H), 1.78 – 1.64 (m, 2H), 1.59 – 1.55 (m, 6H), 1.49 (p, *J* = 7.6 Hz, 2H), 1.33 – 1.24 (m, 5H), 1.24 – 1.18 (m, 1H), 1.14 – 1.06 (m, 18H), 0.84 (t, *J* = 7.1 Hz, 3H); **^{13}C NMR (151 MHz, $CDCl_3$)** δ 154.9, 150.7, 149.0, 141.6, 135.9, 132.7, 128.6 (2C), 128.4 (2C), 127.8, 126.4, 122.8, 114.9, 111.8, 110.4, 69.4, 44.1, 37.5, 35.5, 31.4, 30.7, 29.6, 23.6, 22.6, 20.1, 18.3 (3C), 18.2 (6C), 14.2, 13.5; **^{31}P NMR (243 MHz, $CDCl_3$)** δ -4.82 (d, *J* = 90.7 Hz); **HRMS (APCI)** m/z: $[M+H]^+$ calc. for $C_{37}H_{58}O_5PSi$: 641.3786, observed: 641.3791.

Benzyl butyl [(1'*R*,2'*R*)-6-hydroxy-5'-methyl-4-pentyl-2'-(prop-1-en-2-yl)-1',2',3',4'-tetrahydro-[1,1'-biphenyl]-2-yl] phosphate (41a)

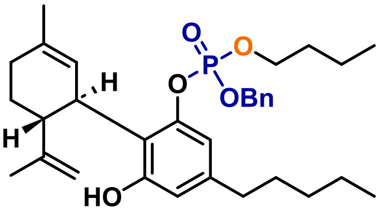

Following general procedure VII, compound **40** (461 mg, 0.72 mmol) was reacted with *n*-butanol (68 mg, 0.936 mmol) to afford the crude silyl protected phosphate diester intermediate. Without performing flash chromatography purification, this intermediate (502 mg, 0.72 mmol, assumed quantitative yield) was subjected to general procedure III. The crude product was purified by flash chromatography (0 – 70% EtOAC in hexanes) to afford the title compound **41a** as a mixture of diastereomers (284 mg, 0.526 mmol, **73%** over two steps, mixture of diastereomers). **Physical state**: Clear oil; **^{1}H NMR (600 MHz, $CDCl_3$)** δ 7.38 – 7.28 (m, 5H), 6.68 (d, *J* = 19.1 Hz, 1H), 6.49 (s, 1H), 6.07 – 5.96 (m, 1H), 5.50 (s, 0.5H), 5.33 (s, 0.5H), 5.17 – 5.06 (m, 2H), 4.52 (d, *J* = 6.5 Hz, 1H), 4.37 (d, *J* = 12.6 Hz, 1H), 4.12 – 4.04 (m, 2H), 3.93 – 3.78 (m, 1H), 2.49 – 2.38 (m, 3H), 2.25 – 2.15 (m, 1H), 2.11 – 2.01 (m, 1H), 1.82 – 1.69 (m, 2H), 1.76 (s, 3H), 1.73* (s, 3H), 1.64 –

1.58 (m, 2H), 1.63 (s, 3H), 1.61* (s, 3H), 1.57 – 1.48 (m, 2H), 1.41 – 1.21 (m, 6H), 0.93 – 0.84 (m, 6H); ^{13}C NMR (151 MHz, $CDCl_3$) δ 156.0, 149.3, 147.1, 143.1, 140.4 (broad peak splitting), 135.9 (dd splitting), 128.7 (2C), 128.7, 128.2, 128.0, 123.7, 118.3, 113.8, 111.7, 69.9(d, J_{CP} = 5.4 Hz), 69.8 (d, J_{CP} = 5.5 Hz), 68.4 (d, J_{CP} = 6.1 Hz), 46.2, 37.0 (d, J_{CP} = 13.8 Hz), 35.6 (d, J_{CP} = 2.3 Hz), 32.4 (d, J_{CP} = 7.1 Hz), 31.6, 30.8 (d, J_{CP} = 4.8 Hz), 30.4, 28.0, 23.8 (d, J_{CP} = 6.1 Hz), 22.6, 19.3, 18.8 (d, J_{CP} = 2.0 Hz), 14.2, 13.7; **^{31}P NMR (243 MHz, $CDCl_3$)** δ -6.21, -6.42; **HRMS (APCI)** m/z: $[M+H]^+$ calc. for $C_{32}H_{46}O_5P$: 541.3077, observed: 541.3079.

Benzyl [(1'*R*,2'*R*)-6-hydroxy-5'-methyl-4-pentyl-2'-(prop-1-en-2-yl)-1',2',3',4'-tetrahydro-[1,1'-biphenyl]-2-yl] butylphosphoramidate (41b)

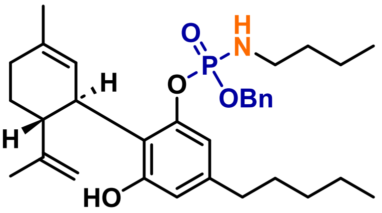

Following general procedure VII, compound **40** (461 mg, 0.72 mmol) was reacted with *n*-butylamine (68 mg, 0.936 mmol) to afford the crude silyl butylphosphoramidate intermediate. Without performing flash chromatography purification, this intermediate (502 mg, 0.72 mmol, assumed quantitative yield) was subjected to general procedure III. The crude product was purified by flash chromatography (0 – 90% EtOAc in hexanes) to afford the title compound **41b** as a mixture of diastereomers (330 mg, 0.612 mmol, **85%** over two steps, mixture of diastereomers). **Physical state**: Clear oil; **^{1}H NMR (600 MHz, $CDCl_3$)** δ 7.38 – 7.29 (m, 5H), 6.77 (s, 1H), 6.47 (s, 1H), 5.53 (s, 1H), 5.37* (s, 1H), 5.08 (d, J = 7.2 Hz, 2H), 4.56 – 4.52 (m, 1H), 4.40 (s, 1H), 3.91 (br s, 1H), 3.81* (br s, 1H), 2.99 – 2.87 (m, 2H), 2.51 – 2.42 (m, 3H), 2.24 – 2.15 (m, 1H), 2.10 – 2.05 (m, 1H), 1.82 – 1.64 (m, 2H), 1.77 (s, 3H), 1.73* (s, 3H), 1.62 (s, 3H), 1.60* (s, 3H), 1.59 – 1.49 (m, 2H), 1.45 – 1.35 (m, 2H), 1.34 – 1.23 (m, 6H), 0.91 – 0.83 (m, 6H); **^{13}C NMR (151 MHz, $CDCl_3$)** δ 156.0, 149.6, 147.6, 143.1, 140.1, 136.6, 136.5*, 128.6, 128.4, 128.4*, 128.0, 127.8, 123.8, 123.7*, 118.2, 113.4, 112.1, 111.9, 68.4 (d, J_{CP} = 5.1 Hz), 68.3* (d, J_{CP} = 5.1 Hz), 46.1, 46.0, 45.9*, 41.6, 41.4*, 37.2, 37.1*, 35.7, 33.9 (d, J_{CP} = 6.6 Hz), 33.8*, 31.6, 30.9, 30.8*, 30.4, 28.1, 28.0*, 23.9, 23.8*, 22.7, 19.9, 19.8*, 19.7, 14.2, 13.8, 13.8*; **^{31}P NMR (243 MHz, $CDCl_3$)** δ 4.31, 4.13; **HRMS (APCI)** m/z: $[M+H]^+$ calc. for $C_{32}H_{47}O_4NP$: 540.3237, observed: 540.3243.

Ammonium butyl [(1'*R*,2'*R*)-6-hydroxy-5'-methyl-4-pentyl-2'-(prop-1-en-2-yl)-1',2',3',4'-tetrahydro-[1,1'-biphenyl]-2-yl] phosphate (42a)

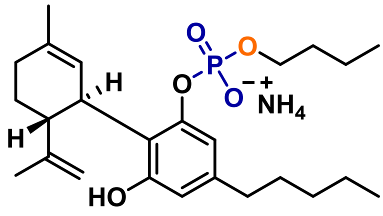

Following general procedure VI, compound **42a** was obtained from debenzylation of **41a** (200 mg, 0.370 mmol). Following workup, the crude product was redissolved in an excess amount of DCM:MeOH:$NH_4OH_{(aq)}$ (80:20:3) solution, stirred for 15 minutes and then evaporated in vacuo. Then, the crude product was purified by flash chromatography (0 – 80% MeOH in DCM) to afford the title compound isolated as the ammonium salt (143 mg, 0.318 mmol, **86%**). **Physical state**: White solid; **^{1}H NMR (600 MHz, MeOD)** δ 6.81 (s, 1H), 6.29 (s, 1H), 5.30 (s, 1H), 4.51 (s, 1H), 4.42 (s, 1H), 4.03 (s, 1H), 3.92 (q, *J* = 6.4 Hz, 2H), 3.00 (q, *J* = 8.9 Hz, 1H), 2.48 – 2.40 (m, 2H), 2.23 (s, 1H), 2.02 – 1.94 (m, 1H), 1.78 – 1.71 (m, 2H), 1.66 (d, *J* = 5.3 Hz, 6H), 1.62 – 1.54 (m, 4H), 1.43 – 1.37 (m, 2H), 1.36 – 1.23 (m, 4H), 0.90 (td, *J* = 7.3, 2.0 Hz, 6H).; **^{13}C NMR (151 MHz, MeOD)** δ 157.5, 153.2, 150.3, 142.4, 133.4, 127.3, 119.9 (d, J_{CP} = 8.3 Hz), 112.0, 111.8, 110.7, 66.8 (d, J_{CP} = 6.1 Hz), 46.1, 38.1, 36.7, 33.9 (d, J_{CP} = 7.7 Hz), 32.7, 32.0, 31.7, 30.8, 23.8, 23.6, 20.1, 19.7, 14.4, 14.2.; **^{31}P NMR (162 MHz, $CDCl_3$)** δ -5.17; **HRMS (ESI-TOF)** m/z: $[M+H]^+$ calc. for $C_{25}H_{40}O_5P$: 451.2608, observed: 451.2614.

Ammonium (1'*R*,2'*R*)-6-hydroxy-5'-methyl-4-pentyl-2'-(prop-1-en-2-yl)-1',2',3',4'-tetrahydro-[1,1'-biphenyl]-2-yl butylphosphoramidate (42b)

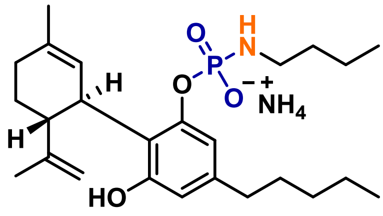

Following general procedure VI, compound **42b** was obtained from debenzylation of **41b** (200 mg, 0.371 mmol). Following workup, the crude product was redissolved in an excess amount of DCM:MeOH:$NH_4OH_{(aq)}$ (80:20:3) solution, stirred for 15 minutes and then evaporated in vacuo. Then, the crude product was purified by flash chromatography (0 – 80% MeOH in DCM) to afford the title compound isolated as the ammonium salt. (135 mg, 0.300 mmol, **81%**). **Physical state**: White solid; **^{1}H NMR (600 MHz, MeOD)** δ 6.87 (s, 1H), 6.24 (s, 1H), 5.34 (s, 1H), 4.51 (s, 1H), 4.40 (s, 1H), 4.07 (s, 1H), 3.00 (s, 1H), 2.94 – 2.81 (m, 1H), 2.43 (t, *J* = 7.8 Hz, 1H), 2.21 (s, 1H), 2.03 – 1.94 (m, 1H), 1.81 – 1.70 (m, 2H), 1.66 (s, 6H), 1.63 – 1.52 (m, 2H), 1.46 – 1.24 (m, 10H), 1.36 – 1.25 (m, 6H), 0.89 (q, *J* = 7.0 Hz, 6H); **^{13}C NMR (151 MHz, MeOD)** δ 157.2, 152.6, 149.9,

142.2, 133.2, 126.8, 119.8, 112.1, 111.7, 110.3, 48.9, 48.7, 48.6, 48.4, 48.3, 45.7, 42.4, 37.7, 36.3, 34.5, 34.5, 32.3, 31.6, 31.3, 30.4, 23.4, 23.2, 20.6, 19.3, 14.0, 13.8; **^{31}P NMR (243 MHz, MeOD)** δ 3.65; **HRMS (APCI)** m/z: $[M-H]^+$ calc. for $C_{25}H_{39}O_4NP$: 448.2622, observed: 448.2618.

5. References

(1) Jang, D. O. Efficient Amidation and Esterification of Phosphoric Acid Using Cl_3CCN/ Ph_3P. *Bulletin of the Korean Chemical Society* **2011**, *32* (9), 3486-3488. DOI: 10.5012/BKCS.2011.32.9.3486.

(2) Ociepa, M.; Knouse, K. W.; He, D.; Vantourout, J. C.; Flood, D. T.; Padial, N. M.; Chen, J. S.; Sanchez, B. B.; Sturgell, E. J.; Zheng, B.; et al. Mild and Chemoselective Phosphorylation of Alcohols Using a Ψ-Reagent. *Organic Letters* **2021**, *23* (24), 9337-9342. DOI: 10.1021/acs.orglett.1c02736.

(3) Mizuno, C. S.; Ma, G.; Khan, S.; Patny, A.; Avery, M. A.; Rimando, A. M. Design, synthesis, biological evaluation and docking studies of pterostilbene analogs inside PPARα. *Bioorganic & Medicinal Chemistry* **2008**, *16* (7), 3800-3808. DOI: 10.1016/j.bmc.2008.01.051.

(4) Eummer, J. T.; Gibbs, B. S.; Zahn, T. J.; Sebolt-Leopold, J. S.; Gibbs, R. A. Novel limonene phosphonate and farnesyl diphosphate analogues: design, synthesis, and evaluation as potential protein-farnesyl transferase inhibitors. *Bioorganic & Medicinal Chemistry* **1999**, *7* (2), 241-250. DOI: 10.1016/S0968-0896(98)00202-8.

(5) Marzullo, P.; Foschi, F.; Coppini, D. A.; Fanchini, F.; Magnani, L.; Rusconi, S.; Luzzani, M.; Passarella, D. Cannabidiol as the Substrate in Acid-Catalyzed Intramolecular Cyclization. *Journal of Natural Products* **2020**, *83* (10), 2894-2901. DOI: 10.1021/acs.jnatprod.0c00436.

(6) Belouin, A.; Simard, R. D.; Joyal, M.; Maharsy, W.; Lau, A.; Prévost, M.; Nemer, M.; Guindon, Y. Sialyl LewisX glycomimetics bearing an extended anionic chain targeting E- and P- selectin binding sites. *Bioorganic & Medicinal Chemistry* **2024**, *98*, 117553. DOI: 10.1016/j.bmc.2023.117553.

(7) Ding, L.; Ma, S.; Lou, H.; Sun, L.; Ji, M. Synthesis and Biological Evaluation of Curcumin Derivatives with Water-Soluble Groups as Potential Antitumor Agents: An in Vitro Investigation Using Tumor Cell Lines. *Molecules* **2015**, *20* (12), 21501-21514. DOI: 10.3390/molecules201219772.

(8) Longhi, M.; Arnaboldi, S.; Husanu, E.; Grecchi, S.; Buzzi, I. F.; Cirilli, R.; Rizzo, S.; Chiappe, C.; Mussini, P. R.; Guazzelli, L. A family of chiral ionic liquids from the natural pool: Relationships between structure and functional properties and electrochemical enantiodiscrimination tests. *Electrochimica Acta* **2019**, *298*, 194-209. DOI: 10.1016/j.electacta.2018.12.060.

(9) Taylor, S. D.; Harris, J. Efficient syntheses of 17-β-amino steroids. *Steroids* **2011**, *76* (10), 1098-1102. DOI: 10.1016/j.steroids.2011.04.013.

(10) Lazar, S.; Jabbouri, S.; Moisand, C.; Noël-Hocquet, S.; Meunier, J. C.; Ropars, C.; Guillaumet, G. Synthesis and biological activity of the phosphate and sulfate esters of naloxone and naltrexone. *European Journal of Medicinal Chemistry* **1994**, *29* (1), 45-53. DOI: 10.1016/0223-5234(94)90125-2.

(11) Juluri, A.; Peddikotla, P.; Repka, M. A.; Murthy, S. N. Transdermal iontophoretic delivery of propofol: A general anaesthetic in the form of its phosphate salt. *Journal of Pharmaceutical Sciences* **2013**, *102* (2), 500-507. DOI: 10.1002/jps.23373.

(12) Blackburn, G. M.; Ingleson, D. The dealkylation of phosphate and phosphonate esters by Iodotrimethylsilane : a mild and selective procedure. *Journal of the Chemical Society, Perkin Transactions 1* **1980**, (0), 1150-1153, 10.1039/P19800001150. DOI: 10.1039/P19800001150.

(13) Markowska, A.; Olejnik, J.; Młotkowska, B.; Sobańska, M. MECHANISMUS DER REAKTION VON TRIARYLPHOSPHITEN MIT PHOSPHORSÄUREN. *Phosphorus and Sulfur and the Related Elements* **1981**, *10* (2), 143-146. DOI: 10.1080/03086648108077496.

(14) Murray, J. I.; Woscholski, R.; Spivey, A. C. Organocatalytic Phosphorylation of Alcohols Using Pyridine-N-oxide. *Synlett* **2015**, *26* (07), 985-990. DOI: 10.1055/s-0034-1379993.

(15) Potrzebowski, M. J.; Assfeld, X.; Ganicz, K.; Olejniczak, S.; Cartier, A.; Gardiennet, C.; Tekely, P. An Experimental and Theoretical Study of the 13C and 31P Chemical Shielding Tensors in Solid O-Phosphorylated Amino Acids. *Journal of the American Chemical Society* **2003**, *125* (14), 4223-4232. DOI: 10.1021/ja029840z.
(16) Salehi Marzijarani, N.; Lam, Y.-h.; Wang, X.; Klapars, A.; Qi, J.; Song, Z.; Sherry, B. D.; Liu, Z.; Ji, Y. New Mechanism for Cinchona Alkaloid-Catalysis Allows for an Efficient Thiophosphorylation Reaction. *Journal of the American Chemical Society* **2020**, *142* (47), 20021-20029. DOI: 10.1021/jacs.0c09192.
(17) Liu, C.-H.; Xu, M.; Luo, Q.; Wang, Z.; Tan, W.; Zhao, X.; Jia, X. Inexpensive and mild hydrogenation condition: Raney Ni-catalyzed reduction of alkenes and alkynes using Et3SiH at ambient temperature. *Tetrahedron* **2024**, *160*, 134040. DOI: https://doi.org/10.1016/j.tet.2024.134040.
